# Disease burden attributable to intimate partner violence against females and sexual violence against children in 204 countries and territories, 1990–2023: a systematic analysis for the Global Burden of Disease Study 2023

**DOI:** 10.1016/S0140-6736(25)02503-6

**Published:** 2026-01-03

**Authors:** GBD 2023 Intimate Partner Violence and Sexual Violence against Children Collaborators, Luisa S Flor, Cory N Spencer, Jack Cagney, Gabriela Fernanda Gil, Hasan Aalruz, Samar Abd ElHafeez, Siddig Ibrahim Abdelwahab, Meriem Abdoun, Mesfin Abebe, Yonas Abebe, Armita Abedi, Roberto Ariel Abeldaño Zuñiga, Alemwork Abie, Olumide Abiodun, Richard Gyan Aboagye, Lucas Guimarães Abreu, Rana Kamal Abu Farha, Bilyaminu Abubakar, Sawsan Abuhammad, Meshack Achore, Lisa C Adams, Babatope Oluwadamilare Adebiyi, Kamoru Ademola Adedokun, Oluwatobi E Adegbile, Nurudeen A Adegoke, Olumide Thomas Adeleke, Makinde Adebayo Adeniyi, Miracle Ayomikun Adesina, Habeeb Omoponle Adewuyi, Qorinah Estiningtyas Sakilah Adnani, Leticia Akua Adzigbli, Aanuoluwapo Adeyimika Afolabi, Rotimi Felix Afolabi, Muhammad Sohail Afzal, Saira Afzal, Williams Agyemang-Duah, Bright Opoku Ahinkorah, Aqeel Ahmad, Danish Ahmad, Muayyad M Ahmad, Asma Ahmed, Ayman Ahmed, Haroon Ahmed, Mehrunnisha Sharif Ahmed, Oli Ahmed, Elizabeth Oluwatoyin Akin-Odanye, Wole Akosile, Idorenyin Ubon Akpabio, Zufishan Alam, Rasmieh Mustafa Al-Amer, Amani N Alansari, Turki M Alanzi, Shereen M Aleidi, Melaku Birhanu Alemu, Fadwa Naji Alhalaiqa, Montaha Al-Iede, Hamid Alinejad Rokny, Wesam Taher Almagharbeh, Md Al-Mamun, Joseph Uy Almazan, Mohmmad Minwer Alnaeem, Intima Alrimawi, Najim Z Alshahrani, Mohammad Sharif Ibrahim Alyahya, Tarek Tawfik Amin, Saeed Amini, Sohrab Amiri, Hubert Amu, Jimoh Amzat, David B Anderson, Boluwatife Stephen Anuoluwa, Saeid Anvari, Anayochukwu Edward Anyasodor, Aleksandr Y Aravkin, Jorge Arias de la Torre, Benedetta Armocida, Alejandra Arrieta, Deepavalli Arumuganainar, Tahira Ashraf, Bilal Aslam, Yuni Asri, Seyyed Shamsadin Athari, Prince Atorkey, Sachin R Atre, Abadi Hailay Atsbaha, Julie Alaere Atta, Madhu Sudhan Atteraya, Ahmed Y Azzam, Sheeba B, Khlood K Baghlaf, Atif Amin Baig, Wondu Feyisa Balcha, Jose Balmori-de-la-Miyar, Soham Bandyopadhyay, Manish Barik, Suzanne Lyn Barker-Collo, MD Abu Bashar, Shahid Bashir, Azadeh Bashiri, Mohammad-Mahdi Bastan, Narasimha M Beeraka, Melesse Belayneh, Gokce Belge Bilgin, Michelle L Bell, Abiye Assefa Berihun, Amiel Nazer C Bermudez, Arushee Bhatnagar, Ashmin Hari Bhattarai, Mohammad Shahangir Biswas, Espen Bjertness, Obasanjo Afolabi Bolarinwa, Paria Bolourinejad, Sri Harsha Boppana, Alejandro Botero Carvajal, Souad Bouaoud, Traolach Brugha, Danilo Buonsenso, Richard A Burns, Yasser Bustanji, Andrea Carugno, Andre F Carvalho, Felix Carvalho, Joao Mauricio Castaldelli-Maia, Sonia Cerrai, Joht Singh Chandan, Miyuru Chandradasa, Periklis Charalampous, Vijay Kumar Chattu, Anis Ahmad Chaudhary, Sirshendu Chaudhuri, Haiyan Chen, Patrick R Ching, Hitesh Chopra, Chidozie Williams Chukwu, Isaac Sunday Chukwu, Sunghyun Chung, Patricia Cullen, Alanna Gomes da Silva, Berihun Assefa Dachew, Omid Dadras, Xiaochen Dai, Koustuv Dalal, Rakhi Dandona, Lucio D'Anna, Samuel Demissie Darcho, Harsha Dayal, Erin M DeGraw, Endalkachew Dellie, Keshab Deuba, Syed Masudur Rahman Dewan, Diana Dias da Silva, Daniel Diaz, Sushil Dohare, Robert Kokou Dowou, Emeka W Dumbili, Jennifer Dunne, Ejemai Eboreime, Cynthia Edeh, Hisham Atan Edinur, Michael Ekholuenetale, Doaa Abdel Wahab El Morsi, Gilbert Eshun, Adeniyi Francis Fagbamigbe, Qiping Fan, Alireza Farahani, Andre Faro, Abidemi Omolara Fasanmi, Alireza Feizkhah, Nuno Ferreira, Richard Charles Franklin, Deborah Ann Fry, Xiang Gao, Miglas Welay Gebregergis, Mesfin Gebrehiwot, Yohannes Fikadu Geda, Miesa Gelchu, Genanew K Getahun, Shakiba Ghasemi Assl, Ehsan Gholami, Nasim Gholizadeh, Elena Ghotbi, Jaleed Ahmed Gilani, Alem Abera Girmay, Mahaveer Golechha, Pouya Goleij, Michal Grivna, Shi-Yang Guan, Damitha Asanga Gunawardane, Sapna Gupta, Pritam Halder, Hassen Mosa Halil, Asif Hanif, Nasrin Hanifi, Habtamu Endashaw Hareru, Josep Maria Haro, Eka Mishbahatul Marah Has, Ahmed I Hasaballah, Hamidreza Hasani, Simon I Hay, Molly E Herbert, Marjan Hesari, Mbuzeleni Hlongwa, Mazeda Hossain, Md Mahbub Hossain, Md Sabbir Hossain, Mohammad Bellal Hossain, Chengxi Hu, Junjie Huang, Yongsong Huang, Pulwasha Maria Iftikhar, Meesha Iqbal, Lalu Muhammad Irham, Teresa R Iskander, Md Shahinul Islam, Sheikh Mohammed Shariful Islam, Roxana Jabbarinejad, Belayneh Hamdela Jena, Ravi Prakash Jha, Nitin Joseph, Charity Ehimwenma Joshua, Jiseung Kang, Kehinde Kazeem Kanmodi, Mehrdad Karajizadeh, Jafar Karami, Faizan Zaffar Kashoo, Inn Kynn Khaing, Himanshu Khajuria, Mariam Khalil, Iqra Hamid Khan, Ramsha Mushtaq Khan, Shaghayegh Khanmohammadi, Sameer Uttamaro Khasbage, Khalid A Kheirallah, Samira Khoshvaght, Mahmood Khosrowjerdi, Jagdish Khubchandani, Jinho Kim, Kwanghyun Kim, Felicia Marie Knaul, Ann Kristin Skrindo Knudsen, Elizabeth Koomson-Yalley, Irene Akwo Kretchy, Kewal Krishan, Barthelemy Kuate Defo, Mohammed Kuddus, Ilari Kuitunen, Mukhtar Kulimbet, Dewesh Kumar, G Anil Kumar, Kamal Kumar, Manasi Kumar, Rakesh Kumar, Vijay Kumar, Abigail Kusi Amponsah, Asep Kusnali, John Paul Kuwornu, Frank Kyei-Arthur, Pallavi L C, Lucie Laflamme, Chandrakant Lahariya, Timo Lajunen, Berthold Langguth, Anne-Marie Laslett, Zohra S Lassi, Areeba Latif, Saheed Akinmayowa Lawal, Aliyu Lawan, Seung Won Lee, Cheru Tesema Leshargie, Jie Li, Zhihui Li, Jue Liu, Xuefeng Liu, Erand Llanaj, Arianna Maever Loreche, Kevin Sheng-Kai Ma, Zheng Feei Ma, Farzan Madadizadeh, Aurea Marilia Madureira-Carvalho, Nozad Hussein Mahmood, Abdelrahman M Makram, Trisha Mallick, Deborah Carvalho Malta, Lokesh Manjani, Joemer C Maravilla, Sammer Marzouk, Clara N Matei, Yasith Mathangasinghe, Khurshid A Mattoo, Pallab K Maulik, Ikechukwu Innocent Mbachu, Susan A McLaughlin, Steven M McPhail, Hadush Negash Meles, Walter Mendoza, Ritesh G Menezes, Endalkachew Worku Mengesha, Tomislav Mestrovic, Sachith Mettananda, Sandrine Donfack D Mewoabi, Andrea Michelerio, Ted R Miller, Giuseppe Minervini, Mojgan Mirghafourvand, Chaitanya Mittal, Mona Gamal Mohamed, Nouh Saad Mohamed, Khabab Abbasher Hussien Mohamed Ahmed, Sakineh Mohammad-Alizadeh-Charandabi, Abdollah Mohammadian-Hafshejani, Shafiu Mohammed, Ali H Mokdad, Hossein Molavi Vardanjani, Lorenzo Monasta, Yousef Moradi, Rafael Silveira Moreira, Rohith Motappa, Kimia Mozahheb Yousefi, Sumaira Mubarik, Oscar J Mujica, Erin C Mullany, Christopher J L Murray, Woojae Myung, Karikalan Nagarajan, Shumaila Nargus, Abdulqadir J Nashwan, Mahmoud Nassar, Samidi Nirasha Kumari Navaratna, Biswa Prakash Nayak, Shalini Ganesh Nayak, Athare Nazri-Panjaki, Anthony Wainaina Ndungu, Finiki Nearchou, Amanuel Tebabal Nega, Ionut Negoi, Cao Duy Nguyen, Cuong Tat Nguyen, Huong Lan Thi Nguyen, Long Nguyen, Syed Toukir Ahmed Noor, Mamoona Noreen, Amir Norouzy, Chisom Adaobi Nri-Ezedi, Chijindu N Nwakama, Felix Kwasi Nyande, Erin M O'Connell, Osaretin Christabel Okonji, John Olayemi Okunlola, Oluwaseyi Isaiah Olabisi, Comfort Z Olorunsaiye, Sandersan Onie, Obinna E Onwujekwe, Atakan Orscelik, Esteban Ortiz-Prado, Augustus Osborne, Uchechukwu Levi Osuagwu, Amel Ouyahia, Mahesh P A, Jagadish Rao Padubidri, Raul Felipe Palma-Alvarez, Ioannis Pantazopoulos, Romil R Parikh, Arpit Parmar, Ava Pashaei, Maja Pasovic, Jay Patel, Sangram Kishor Patel, Shankargouda Patil, Shrikant Pawar, Shubhadarshini Pawar, Prince Peprah, Ramesh Poluru, Naeimeh Pourtaheri, Jalandhar Pradhan, Elton Junio Sady Prates, Shuby Puthussery, Jagadeesh Puvvula, Ibrahim Qattea, Xiang Qi, Zhipeng Qi, Yanan Qiao, Kabir Ayobami Raheem, Vafa Rahimi-Movaghar, Md Mosfequr Rahman, Mosiur Rahman, Muhammad Aziz Rahman, Ivano Raimondo, Sathish Rajaa, Pushp Lata Rajpoot, Mahmoud Mohammed Ramadan, Sheena Ramazanu, Chhabi Lal Ranabhat, Sowmya J Rao, Devarajan Rathish, Santosh Kumar Rauniyar, Mohsen Rezaeian, Taeho Gregory Rhee, Jefferson Antonio Buendia Rodriguez, Leonardo Roever, Luca Ronfani, Moustaq Karim Khan Rony, Allen Guy Patrick Ross, Hanieh Rouzbahani, Shiva Rouzbahani, Priyanka Roy, Sharmistha Roy, Robert Rudolf, Cameron John Sabet, Mohd Saeed, Umar Saeed, Rajesh Sagar, Dominic Sagoe, Pragyan Monalisa Sahoo, S Mohammad Sajadi, Dauda Salihu, Sonia Sameen, Abdallah M Samy, Damian F Santomauro, Milena M Santric-Milicevic, Tanmay Sarkar, Gargi Sachin Sarode, Sachin C Sarode, Maheswar Satpathy, Monika Sawhney, Siddharthan Selvaraj, Yashendra Sethi, Muhammad Shahab, Samiah Shahid, Moyad Jamal Shahwan, Masood Ali Shaikh, Nafhat Shaikh, Anas Shamsi, Alfiya Shamsutdinova, Dan Shan, Mohd Shanawaz, Mohammed Shannawaz, Vishal Sharma, Mahabalesh Shetty, Premalatha K Shetty, Wenming Shi, Md Monir Hossain Shimul, Rahman Shiri, Aminu Shittu, Ivy Shiue, Seyed Afshin Shorofi, Emmanuel Edwar Siddig, Gustavo Correia Basto da Silva, Akanksha Singh, Baljinder Singh, Marco Aurelio Sousa, Muhammad Haroon Stanikzai, Caroline Stein, Dan J Stein, Brendon Stubbs, Vetriselvan Subramaniyan, Mahwish Suhaib, Mark J M Sullman, Jing Sun, Chandan Kumar Swain, Sree Sudha T Y, Rafael Tabarés-Seisdedos, Seyyed Mohammad Tabatabaei, Celine Tabche, Mircea Tampa, Minale Tareke, Sarvenaz Taridashti, Anika Tasnim, Mohamad-Hani Temsah, Azimeraw Arega Tesfu, Rekha Thapar, Muthu Thiruvengadam, Wei Tian, Marcos Roberto Tovani-Palone, Tam Quoc Minh Tran, Thang Huu Tran, Samuel Joseph Tromans, Claudia Truppa, Alexander C Tsai, Evangelia Eirini Tsermpini, Aisha Twalibu, Bhaskaran Unnikrishnan, Zahir Vally, Nadia Machado Vasconcelos, Aliscia Vieira, David Villarreal-Zegarra, Manish Vinayak, Theo Vos, Yasir Waheed, Megha Walia, Qingzhi Wang, Wei Wang, Yuan-Pang Wang, Nuwan Darshana Wickramasinghe, Martin Wiredu Agyekum, Wanqing Xie, Wanqing Xu, Saba Yahoo (Syed), Guangcan Yan, Yuqi Yang, Pengpeng Ye, Renjulal Yesodharan, Siyan Yi, Yazachew Engida Yismaw, Dong Keon Yon, Naohiro Yonemoto, Chuanhua Yu, Umar Yunusa, Siddhesh Zadey, Giulia Zamagni, Hussaini Zandam, Mohammed G M Zeariya, Alemu Birara Zemariam, Beijian Zhang, Haijun Zhang, Abzal Zhumagaliuly, Mikhail Zinchuk, Mohamed Ali Zoromba, Sa'ed H Zyoud, Emmanuela Gakidou

**Affiliations:** A Institute for Health Metrics and Evaluation, University of Washington, Seattle, WA, USA; B Department of Health Metrics Sciences, School of Medicine, University of Washington, Seattle, WA, USA; C Department of Nursing, Al Zaytoonah University of Jordan, Amman, Jordan; D Department of Epidemiology, Alexandria University, Alexandria, Egypt; E Health Research Centre, Jazan University, Jazan, Saudi Arabia; F Department of Medicine, University of Setif Algeria, Sétif, Algeria; G Department of Health, University of Setif Algeria, Sétif, Algeria; H Department of Midwifery, Dilla University, Dilla, Ethiopia; I Department of Emergency Medicine, Zanjan University of Medical Sciences, Zanjan, Iran; J Postgraduate Department, University of Sierra Sur, Miahuatlan de Porfirio Diaz, Mexico; K Yhteiskuntadatatieteen keskus (Centre for Social Data Science), University of Helsinki, Helsinki, Finland; L Department of Midwifery, Bahir Dar University, Bahir Dar, Ethiopia; M Department of Community Medicine, Babcock University, Ilishan-Remo, Nigeria; N Department of Family and Community Health, University of Health and Allied Sciences, Ho, Ghana; O School of Population Health, University of New South Wales, Sydney, NSW, Australia; P Department of Pediatric Dentistry, Federal University of Minas Gerais, Belo Horizonte, Brazil; Q Clinical Pharmacy and Therapeutics Department, Applied Science Private University, Amman, Jordan; R Department of Pharmacology and Toxicology, Usmanu Danfodiyo University, Sokoto, Sokoto, Nigeria; S Nigerian Institute of Medical Research, Lagos, Nigeria; T Department of Nursing, University of Sharjah, Sharjah, United Arab Emirates; U Maternal and Child Health Nursing, Jordan University of Science and Technology, Irbid, Jordan; V Department of Population Health, Hofstra University, Hempstead, NY, USA; W Department of Diagnostic and Interventional Radiology, Technical University of Munich, Munich, Germany; X Stanford University, Palo Alto, CA, USA; Y Department of Pediatrics, University of Calgary, Calgary, AB, Canada; Z School of Public Health, University of the Western Cape, Bellville, South Africa; AA Department of Immunology, Roswell Park Comprehensive Cancer Center, Buffalo, NY, USA; AB Graduate Program Division, University at Buffalo, Buffalo, NY, USA; AC Department of Pediatrics, East Tennessee State University, Johnson City, TN, USA; AD Center for Cardiovascular Risk Research, Center for Cardiovascular Risk Research, Johnson City, TN, USA; AE Translational Research Team, The University of Sydney, Sydney, NSW, Australia; AF Melanoma Institute Australia, The University of Sydney, Sydney, NSW, Australia; AG Department of Family Medicine, Bowen University, Iwo, Nigeria; AH Department of Family Medicine, Bowen University Teaching Hospital, Ogbomoso, Nigeria; AI Department of Community Medicine and Primary Care, Federal Medical Center Abeokuta, Abeokuta, Nigeria; AJ Slum and Rural Health Initiative Research Academy, Slum and Rural Health Initiative, Ibadan, Nigeria; AK Department of Physiotherapy, University of Ibadan, Ibadan, Nigeria; AL Department of Educational Counselling and Developmental Psychology, University of Ibadan, Ibadan, Nigeria; AM Department of Educational Psychology, University of Johannesburg, Johannesburg, South Africa; AN Department of Public Health, Universitas Padjadjaran (Padjadjaran University), Bandung, Indonesia; AO Department of Epidemiology and Biostatistics, University of Health and Allied Sciences, Ho, Ghana; AP Technical Services Directorate, MSI Nigeria Reproductive Choices, Abuja, Nigeria; AQ Department of Epidemiology and Medical Statistics, University of Ibadan, Ibadan, Nigeria; AR Department of Life Sciences, University of Management and Technology, Lahore, Pakistan; AS Department of Community Medicine, King Edward Memorial Hospital, Lahore, Pakistan; AT Department of Public Health, Public Health Institute, Lahore, Pakistan; AU Department of Public Health Sciences, Queen's University, Kingston, ON, Canada; AV School of Public Health, University of Technology Sydney, Sydney, NSW, Australia; AW College of Medicine, Shaqra University, Shaqra, Saudi Arabia; AX School of Medicine and Psychology, Australian National University, Canberra, ACT, Australia; AY Health Research Institute, University of Canberra, Canberra, NSW, Australia; AZ School of Nursing, University of Jordan, Amman, Jordan; BA Institute of Molecular Biology and Biotechnology, The University of Lahore, Lahore, Pakistan; BB Institute of Endemic Diseases, University of Khartoum, Khartoum, Sudan; BC Pan-African One Health Institute (PAOHI), Kigali, Rwanda; BD Department of Biosciences, COMSATS Institute of Information Technology, Islamabad, Pakistan; BE College of Nursing, Majmaah University, Al Majmaah, Saudi Arabia; BF Department of Psychology, University of Chittagong, Chattogram, Bangladesh; BG Clinical Psychology Department, University College Hospital, Ibadan, Ibadan, Nigeria; BH Faculty of Health and Behavioural Sciences, The University of Queensland, Brisbane, QLD, Australia; BI Department of Psychiatry and Mental Health, University of Cape Town, Cape Town, South Africa; BJ Western Cape Department of Health, Cape Town, South Africa; BK School of Health and Environmental Studies, Hamdan Bin Mohammed Smart University, Dubai, United Arab Emirates; BL School of Nursing, Yarmouk University, Irbid, Jordan; BM School of Nursing and Midwifery, Western Sydney University, Sydney, NSW, Australia; BN Department of Pediatric Surgery, Hamad Medical Corporation, Doha, Qatar; BO Department of Health Information Management and Technology, Imam Abdulrahman Bin Faisal University, Dammam, Saudi Arabia; BP College of Pharmacy, University of Sharjah, Sharjah, United Arab Emirates; BQ School of Pharmacy, The University of Jordan, Amman, Jordan; BR School of Population Health, Curtin University, Perth, WA, Australia; BS Department of Health Systems and Policy, University of Gondar, Gondar, Ethiopia; BT College of Nursing, Qatar University, Doha, Qatar; BU The School of Medicine, The University of Jordan, Amman, Jordan; BV The Graduate School of Biomedical Engineering, University of New South Wales, Sydney, NSW, Australia; BW Faculty of Nursing, University of Tabuk, Tabuk, Saudi Arabia; BX BRAC Institute of Governance and Development (BIGD), BRAC University, Dhaka, Bangladesh; BY Department of Public and Community Health, Frontier University Garowe (FUG), Puntland, Somalia; BZ Department of Medicine, Nazarbayev University, Astana, Kazakhstan; CA Department of Clinical Nursing, Al-Zaytoonah University of Jordan, Amman, Jordan; CB Department of Nursing, Georgetown University, Washington, DC, USA; CC Department of Family and Community Medicine, University of Jeddah, Jeddah, Saudi Arabia; CD Faculty of Medicine, Jordan University of Science and Technology, Irbid, Jordan; CE Public Health and Community Medicine Department, Cairo University, Cairo, Egypt; CF Department of Health and Management Sciences, Khomein University of Medical Sciences, Khomein, Iran; CG Spiritual Health Research Center, Baqiyatallah University of Medical Sciences, Tehran, Iran; CH Department of Population and Behavioural Sciences, University of Health and Allied Sciences, Ho, Ghana; CI Department of Sociology, Usmanu Danfodiyo University, Sokoto, Sokoto, Nigeria; CJ Department of Sociology, University of Johannesburg, Johannesburg, South Africa; CK Faculty of Medicine and Health, The University of Sydney, Sydney, NSW, Australia; CL Sydney Musculoskeletal Health, The University of Sydney, Sydney, NSW, Australia; CM Department of Environmental and Occupational Health, University of Medical Sciences, Ondo, Ondo, Nigeria; CN Regenerative Medicine, Organ Procurement and Transplantation Multi-disciplinary Center, Guilan University of Medical Sciences, Rasht, Iran; CO Rural Health Research Institute, Charles Sturt University, Orange, NSW, Australia; CP Department of Applied Mathematics, University of Washington, Seattle, WA, USA; CQ Care in Long Term Conditions Research Division, King's College London, London, UK; CR CIBER Epidemiology and Public Health (CIBERESP), Madrid, Spain; CS Department of Cardiovascular, Endocrine-Metabolic Diseases and Aging, Istituto Superiore di Sanità (ISS), Rome, Italy; CT Department of Periodontics, Saveetha University, Chennai, India; CU Pioneer Journal of Biostatistics and Medical Research (PJBMR), Pakistan, Pakistan; CV Department of Pediatrics, Lahore General Hospital, Lahore, Pakistan; CW Nursing Department, Institute of Technology and Health Science RS dr Soepraoen, Malang, Indonesia; CX Faculty of Health Science, Institute of Technology and Health Science RS dr Soepraoen, Malang, Indonesia; CY Department of Immunology, Zanjan University of Medical Sciences, Zanjan, Iran; CZ School of Medicine and Public Health, University of Newcastle, Newcastle, NSW, Australia; DA Discipline of Psychological Science, ACAP University College, Sydney, NSW, Australia; DB Hospital and Research Centre, Dr. D. Y. Patil Vidyapeeth Pune (Deemed to be University), Pune, India; DC Center for Clinical Global Health Education, Johns Hopkins University, Baltimore, MD, USA; DD College of Medicine and Health Sciences, Adigrat University, Adigart, Ethiopia; DE Management Policy and Community Health, University of Texas, Houston, TX, USA; DF Department of Social Welfare, Keimyung University, Daegu, South Korea; DG ASIDE Healthcare, Lewes, DE, USA; DH Faculty of Medicine, October 6 University, 6th of October City, Egypt; DI School of Public Health, JSS Academy of Higher Education and Research, Mysuru, India; DJ Pediatric Dentistry Department, King Abdulaziz University, Jeddah, Saudi Arabia; DK International Medical School, Management and Science University, Alam, Malaysia; DL Anahuac Business School, Universidad Anahuac Mexico, Mexico City, Mexico; DM Nuffield Department of Surgical Sciences, University of Oxford, Oxford, UK; DN Department of Neurosurgery, University of Southampton, Southampton, UK; DO Global Health Research Department, IMPACT Global Consulting, New Delhi, India; DP Foundation for Reproductive Health Services India, New Delhi, India; DQ School of Psychology, University of Auckland, Auckland, New Zealand; DR Department of Community and Family Medicine, All India Institute of Medical Sciences, Gorakhpur, India; DS University Institute of Food Science and Technology, The University of Lahore, Lahore, Pakistan; DT Health Information Management, Shiraz University of Medical Sciences, Shiraz, Iran; DU Non-communicable Diseases Research Center, Tehran University of Medical Sciences, Tehran, Iran; DV School of Medicine, Iran University of Medical Sciences, Tehran, Iran; DW Department of Human Anatomy and Histology, I.M. Sechenov First Moscow State Medical University, Moscow, Russia; DX Department of Public Health, Bahir Dar University, Bahir Dar, Ethiopia; DY Department of Public Health, University of South Africa, Pretoria, South Africa; DZ Department of Radiology, Mayo Clinic, Rochester, MN, USA; EA School of the Environment, Yale University, New Haven, CT, USA; EB School of Health Policy and Management, Korea University, Seoul, South Korea; EC School of Public Health, Johns Hopkins University, Baltimore, MD, USA; ED Department of Epidemiology and Biostatistics, University of the Philippines Manila, Manila, Philippines; EE Family Medicine Department, Texas Tech University, El Paso, TX, USA; EF Program Management Department, Emotional Well-Being Institute Canada, Burnaby, BC, Canada; EG Department of Biochemistry and Biotechnology, University of Science and Technology Chittagong, Chittagong, Bangladesh; EH Department of Community Medicine and Global Health, University of Oslo, Oslo, Norway; EI Department of Global Healthcare Management, York St John University, London, UK; EJ Department of Demography and Population Studies, University of the Witwatersrand, Johannesburg, South Africa; EK Isfahan University of Medical Sciences, Isfahan, Iran; EL Department of Anesthesia and Critical Care Medicine, Johns Hopkins University, Baltimore, MD, USA; EM Facultad de Salud (Faculty of Health), Universidad Santiago de Cali, Cali, Colombia; EN Department of Medicine, University Ferhat Abbas of Setif, Setif, Algeria; EO Department of Epidemiology and Preventive Medicine, University Hospital Saadna Abdenour, Setif, Algeria; EP Department of Health Sciences, University of Leicester, Leicester, UK; EQ Department of Woman and Child Health and Public Health, Fondazione Policlinico Universitario A. Gemelli IRCCS (Agostino Gemelli University Polyclinic IRCCS), Rome, Italy; ER Global Health Research Institute, Università Cattolica del Sacro Cuore (Catholic University of Sacred Heart), Rome, Italy; ES National Centre for Epidemiology and Population Health, Australian National University, Canberra, ACT, Australia; ET Department of Basic Biomedical Sciences, University of Sharjah, Sharjah, United Arab Emirates; EU Department of Medicine and Surgery, University of Insubria, Varese, Italy; EV IMPInstitute for Mental and Physical Health and Clinical Translation (IMPACT), Deakin University, Geelong, VIC, Australia; EW Research Unit on Applied Molecular Biosciences (UCIBIO), University of Porto, Porto, Portugal; EX Department of Psychiatry, University of São Paulo, São Paulo, Brazil; EY Institute of Clinical Physiology, Italian National Council of Research, Pisa, Italy; EZ Department of Applied Health Sciences, University of Birmingham, Birmingham, UK; FA Department of Psychiatry, University of Kelaniya, Ragama, Sri Lanka; FB University Psychiatry Unit, Colombo North Teaching Hospital, Ragama, Sri Lanka; FC Department of Public Health, Erasmus University Medical Center, Rotterdam, Netherlands; FD Department of Epidemiology and Biostatistics, Semey Medical University (SMU), Semey, Kazakhstan; FE Department of Community Medicine, Datta Meghe Institute of Medical Sciences, Sawangi, India; FF Department of Biology, Imam Mohammad Ibn Saud Islamic University, Riyadh, Saudi Arabia; FG Department of Public Health, Indian Institute of Public Health, Hyderabad, India; FH Clinical Project Management Office, National Clinical Research Center for Infectious Diseases, Shenzhen, Shenzhen, China; FI Division of Infectious Diseases, Virginia Commonwealth University, Richmond, VA, USA; FJ Centre for Research Impact & Outcome, Chitkara University, Rajpura, India; FK Department of Mathematical Sciences, Georgia Southern University, Statesboro, GA, USA; FL Department of Paediatric Surgery, Federal Medical Centre, Umuahia, Nigeria; FM Department of Health Behavior, Texas A&M University, College Station, TX, USA; FN School of Population Health, University of New South Wales, Kensington, NSW, Australia; FO Global Women's Health Program, The George Institute for Global Health, Newtown, NSW, Australia; FP School of Nursing, Federal University of Minas Gerais, Belo Horizonte, Brazil; FQ School of Public Health, Curtin University, Perth, WA, Australia; FR Department of Epidemiology, University of Gondar, Gondar, Ethiopia; FS Department of Health, Northern Territory Government, Darwin, SA, Australia; FT Institute for Health Sciences, Mid Sweden University, Sundsvall, Sweden; FU Public Health Foundation of India, Gurugram, India; FV Department of Brain Sciences, Imperial College London, London, UK; FW Department of Public Health, Haramaya University, Harar, Ethiopia; FX Department of Planning Monitoring and Evaluation, The Presidency Office, Pretoria, South Africa; FY Pan-African Collective for Evidence, Johannesburg, South Africa; FZ Public Health and Environment Research Centre (PERC) in Nepal, Lalitpur, Nepal; GA Global Health Epidemiology Research Group, University of Bergen, Bergen, Norway; GB Department of Pharmacy, United International University, Dhaka, Bangladesh; GC Pharmacology Division, Center for Life Sciences Research Bangladesh, Dhaka, Bangladesh; GD Escola Superior de Saúde (Higher School of Health), Instituto Politécnico do Porto (Polytechnic Institute of Porto), Porto, Portugal; GE UCIBIO Applied Molecular Biosciences Unit, University of Porto, Porto, Portugal; GF Public Health Intelligence Unit, National Institute of Public Health, Cuernavaca, Mexico; GG Department of Public Health, Jazan University, Jazan, Saudi Arabia; GH School of Sociology, University College Dublin, Dublin, Ireland; GI Department of Psychiatry, Dalhousie University, Halifax, NS, Canada; GJ Department of Psychiatry, University of Alberta, Edmonton, AB, Canada; GK School of Medicine, Johns Hopkins University, Baltimore, MD, USA; GL School of Health Sciences, Universiti Sains Malaysia (University of Science Malaysia), Kubang Kerian, Malaysia; GM Faculty of Science and Health, University of Portsmouth, Hampshire, UK; GN Department of Forensic Medicine and Clinical Toxicology, Mansoura University, Mansoura, Egypt; GO Department of Medical Education, Delta University for Science and Technology, Mansoura, Egypt; GP Wassa Amenfi East Municipal Health Directorate, Ghana Health Service, Wassa Akropong, Ghana; GQ Research Centre for Healthcare and Community, Coventry University, Coventry, UK; GR Department of Public Health Sciences, Clemson University, Clemson, SC, USA; GS Department of Medicine, Iran University of Medical Sciences, Tehran, Iran; GT Department of Psychology, Federal University of Sergipe, São Cristóvão, Brazil; GU Satcher Health Leadership Institute, Morehouse School of Medicine, Atlanta, GA, USA; GV School of Medicine, Emory University, Atlanta, GA, USA; GW Department of Social Medicine and Epidemiology, Guilan University of Medical Sciences, Rasht, Iran; GX Department of Social Sciences, University of Nicosia, Nicosia, Cyprus; GY College of Medicine, Dentistry and Public Health, James Cook University, Townsville, QLD, Australia; GZ Childlight – Global Child Safety Institute, University of Edinburgh, Edinburgh, UK; HA Department of Biostatistics, Xuzhou Medical University, Xuzhou, China; HB Key Lab of Environment and Health, Xuzhou Medical University, Xuzhou, China; HC Department of Midwifery, Adigrat University, Adigrat, Ethiopia; HD Environmental Pollution Monitoring and Study Desk, Ethiopian Environmental Protection Authority, Addis Ababa, Ethiopia; HE Department of Midwifery, Wolkite University, Wolkite, Ethiopia; HF School of Public Health, Bule Hora University, Bule Hora, Ethiopia; HG Department of Public Health, Menelik II Medical and Health Science College, Addis Ababa, Ethiopia; HH Department of Global Health Sciences, University of California San Francisco, San Francisco, CA, USA; HI Department of Electrical and Computer Engineering, University of California Davis, Davis, CA, USA; HJ Department of Dermatology, Mazandaran University of Medical Sciences, Sari, Iran; HK Obstetrics and Gynecology Department, Shahid Beheshti University of Medical Sciences, Tehran, Iran; HL Aga Khan University, Karachi, Pakistan; HM Department of Nursing, Aksum University, Aksum, Ethiopia; HN Department of Health Systems and Policy Research, Indian Institute of Public Health, Gandhinagar, India; HO Department of Genetics, Sana Institute of Higher Education, Sari, Iran; HP Universal Scientific Education and Research Network (USERN), Kermanshah University of Medical Sciences, Kermanshah, Iran; HQ Institute of Public Health, United Arab Emirates University, Al Ain, United Arab Emirates; HR Department of Public Health and Preventive Medicine, Charles University, Prague, Czech Republic; HS Department of Epidemiology and Biostatistics, Anhui Medical University, Hefei, China; HT Department of Community Medicine, University of Peradeniya, Kandy, Sri Lanka; HU Department of Toxicology, Shriram Institute for Industrial Research, Delhi, India; HV Department of Community Medicine, Post Graduate Institute of Medical Education and Research, Chandigarh, India; HW Centre for Community Medicine, All India Institute of Medical Sciences, New Delhi, India; HX Department of Midwifery, Werabe University, Werabe, Ethiopia; HY College of Medicne and Health Science, Werabe University, Werabe, Ethiopia; HZ Sakarya University, Sakarya, Turkiye; IA Department of Critical Care and Emergency Nursing, Zanjan University of Medical Sciences, Zanjan, Iran; IB School of Public Health, Dilla University, Dilla, Ethiopia; IC Research Unit, Parc Sanitari Sant Joan de Deu, Barcelona, Spain; ID Department of Mental Health, Biomedical Research Networking Center for Mental Health Network (CiberSAM), Madrid, Spain; IE Department of Advanced Nursing, Universitas Airlangga (Airlangga University), Surabaya, Indonesia; IF School of Nursing and Midwivery, La Trobe University, Bundoora, VIC, Australia; IG Department of Zoology and Entomology, Al-Azhar University, Cairo, Egypt; IH Department of Ophthalmology, Iran University of Medical Sciences, Tehran, Iran; II Department of Pediatrics, Jacobi Medical Center, New York, NY, USA; IJ School of Nursing and Public Health Medicine, University of KwaZulu-Natal, Durban, South Africa; IK Eastern Africa Centre and Institute for Health & Allied Professionals, Nottingham Trent University, Nottingham, UK; IL Department of Decision and Information Sciences, University of Houston, Houston, TX, USA; IM Public Health Research Group, Nature Study Society of Bangladesh, Khulna, Bangladesh; IN Department of Statistics, Shahjalal University of Science and Technology, Sylhet, Bangladesh; IO Department of Population Sciences, University of Dhaka, Dhaka, Bangladesh; IP Department of Psychological and Cognitive Sciences, Tsinghua University, Beijing, China; IQ Faculty of Medicine, The Chinese University of Hong Kong, Hong Kong, China; IR Advanced Institute of Convergence Knowledge Informatics, Tohoku University, Sendai, Japan; IS Graduate School of Engineering, Tohoku University, Sendai, Japan; IT Health Policy and Management Department, City University of New York, New York, NY, USA; IU Faculty of Pharmacy, Universitas Ahmad Dahlan, Yogyakarta, Indonesia; IV Independent Researcher, Cairo, Egypt; IW Faculty of Engineering and Technology, Eastern University, Dhaka, Bangladesh; IX Journal of Biosciences and Public Health (JBPH), 4-Green Research Society, Dhaka, Bangladesh; IY Department of Nutrition, Texas Tech University, Lubbock, TX, USA; IZ Department of Physical Medicine & Rehabilitation, Northwestern University, Chicago, IL, USA; JA Department of Psychiatry, Tehran University of Medical Sciences, Tehran, Iran; JB Department of Epidemiology, Wachemo University, Hossana, Ethiopia; JC Department of Community Medicine, Dr. Baba Saheb Ambedkar Medical College & Hospital, Delhi, India; JD Department of Community Medicine, Banaras Hindu University, Varanasi, India; JE Department of Community Medicine, Manipal Academy of Higher Education, Mangalore, India; JF Department of Economics, National Open University, Benin City, Nigeria; JG School of Health and Environmental Science, Korea University, Seoul, South Korea; JH Department of Anesthesia, Critical Care and Pain Medicine, Massachusetts General Hospital, Boston, MA, USA; JI Office of the Executive Director, Cephas Health Research Initiative Inc, Ibadan, Nigeria; JJ College of Health Sciences, Caleb Univeristy, Imota, Nigeria; JK Trauma Research Center, Shiraz University of Medical Sciences, Shiraz, Iran; JL Laboratory Science Department, Khomein University of Medical Sciences, Khomein, Iran; JM Department of Immunology, Tehran University of Medical Sciences, Tehran, Iran; JN Department of Physical Therapy and Health Rehabilitation, Majmaah University, Majmaah, Saudi Arabia; JO Department of Public Health and Health Policy, Hiroshima University, Hiroshima, Japan; JP Amity Institute of Forensic Sciences, Amity University, Noida, India; JQ Department of Global Health, University of Washington, Seattle, WA, USA; JR University Institute of Public Health, The University of Lahore, Lahore, Pakistan; JS Department of Community and Preventive Medicine, King Edward Medical University, Lahore, Pakistan; JT Department of Epidemiology, Non-Communicable Diseases Research Center (NCDRC), Tehran, Iran; JU School of Medicine, Tehran University of Medical Sciences, Tehran, Iran; JV Department of Pharmacology, All India Institute of Medical Sciences, Raipur, India; JW Department of Public Health, Jordan University of Science and Technology, Irbid, Jordan; JX Institute of Research and Development, Duy Tan University, Da Nang, Vietnam; JY Research Department, University of Inland Norway, Elverum, Norway; JZ Department of Public Health, New Mexico State University, Las Cruces, NM, USA; KA Department of Health Policy and Management, Korea University, Seoul, South Korea; KB School of Medicine, Creighton University, Omaha, NE, USA; KC Department of Medicine, University of California Los Angeles, Los Angeles, CA, USA; KD Escuela de Medicina y Ciencias de la Salud, Tecnológico de Monterrey, Mexico City, Mexico; KE Centre for Disease Burden, Norwegian Institute of Public Health, Bergen, Norway; KF Department of Sociology and Social Work, Kwame Nkrumah University of Science and Technology, Kumasi, Ghana; KG School of Pharmacy, University of Ghana, Legon, Ghana; KH Department of Anthropology, Panjab University, Chandigarh, India; KI Department of Demography, University of Montreal, Montreal, QC, Canada; KJ Department of Social and Preventive Medicine, University of Montreal, Montreal, QC, Canada; KK Department of Biochemistry, University of Hail, Hail, Saudi Arabia; KL Department of Pediatrics, Kuopio University Hospital, Kuopio, Finland; KM Institute of Clinical Medicine, University of Eastern Finland, Kuopio, Finland; KN Research and Publication Activity Division, Kazakh National Medical University, Almaty, Kazakhstan; KO Center of Medicine and Public Health, Asfendiyarov Kazakh National Medical University, Almaty, Kazakhstan; KP Department of Community Medicine, Rajendra Institute of Medical Sciences, Ranchi, India; KQ Department of Mathematics, Amity University Haryana, Gurugram, India; KR Institute for Excellence in Health Equity, New York University, New York, NY, USA; KS Department of Psychiatry, University of Nairobi, Nairobi, Kenya; KT Department of Health Management, University of Hail, Hail, Saudi Arabia; KU Department of Economics, Manipal University, Jaipur, India; KV Department of Public Health Nursing, Kwame Nkrumah University of Science and Technology, Kumasi, Ghana; KW National Research and Innovation Agency (BRIN), Jakarta, Indonesia; KX Australian Centre for Health Services Innovation, Queensland University of Technology, Kelvin Grove, QLD, Australia; KY Department of Environment and Public Health, University of Environment and Sustainable Development, Somanya, Ghana; KZ Kasturba Medical College, Manipal, Manipal Academy of Higher Education, Manipal, India; LA Department of Global Public Health, Karolinska Institute, Stockholm, Sweden; LB Institute for Social and Health Sciences, University of South Africa, Pretoria, South Africa; LC Division of Evidence Synthesis, Foundation for People-centric Health Systems, New Delhi, India; LD Division of Lifestyle Medicine, Centre for Health: The Specialty Practice, New Delhi, India; LE Department of Psychology, University of Helsinki, Helsinki, Finland; LF Department of Psychology, Norwegian University of Science and Technology, Trondheim, Norway; LG Department of Psychiatry and Psychotherapy, University of Regensburg, Regensburg, Germany; LH Centre for Alcohol Policy Research, La Trobe University, Melbourne, VIC, Australia; LI National Drug Research Institute, Curtin University, Perth, WA, Australia; LJ Robinson Research Institute, University of Adelaide, Adelaide, SA, Australia; LK Department of Pediatrics, Aga Khan University, Karachi, Pakistan; LL University of Management and Technology, Lahore, Pakistan; LM Health Systems, Administration and Management, Babcock University, Sagamu, Nigeria; LN Health Services Management Programme, Plasma University, Mogadishu, Somalia; LO School of Physical Therapy, The University of Western Ontario, London, ON, Canada; LP Department of Precision Medicine, Sungkyunkwan University, Suwon-si, South Korea; LQ Charles Sturt University, Haramaya University, Sydney, NSW, Australia; LR Debre Markos University, Ethiopia; LS Global Health Research Center, Guangdong Academy of Medical Sciences and General Hospital, Guangzhou, China; LT Tsinghua Vanke School of Public Health, Tsinghua University, Beijing, China; LU Department of Global Health and Population, Harvard University, Boston, MA, USA; LV Department of Epidemiology and Biostatistics, Peking University, Beijing, China; LW Lerner Research Institute, Cleveland Clinic, Cleveland, OH, USA; LX Department of Quantitative Health Science, Case Western Reserve University, Cleveland, OH, USA; LY Department of Molecular Epidemiology, German Institute of Human Nutrition Potsdam-Rehbrücke, Potsdam, Germany; LZ German Center for Diabetes Research (DZD), München-Neuherberg, Germany; MA National Institutes of Health, University of the Philippines Manila, Manila, Philippines; MB Ateneo School of Government, Ateneo De Manila University, Quezon City, Philippines; MC Center for Global Health, University of Pennsylvania, Philadelphia, PA, USA; MD Centre for Public Health and Wellbeing, University of the West of England, Bristol, UK; ME Department of Biostatistics and Epidemiology, Yazd University of Medical Sciences, Yazd, Iran; MF Associate Laboratory i4HB, University Institute of Health Sciences - CESPU, Gandra, Portugal; MG UCIBIO Research Unit on Applied Molecular Biosciences, University Institute of Health Sciences, Gandra, Portugal; MH Research Center, Cihan University-Sulaimaniya, Sulaymaniyah, Iraq; MI School of Public Health, Imperial College London, London, UK; MJ The Orthopaedic Department, October 6 University, 6th of October City, Egypt; MK Maternal and Child Health Division, International Centre for Diarrhoeal Disease Research, Bangladesh, Dhaka, Bangladesh; ML Department of Maternal-Child Nursing and Public Health, Federal University of Minas Gerais, Belo Horizonte, Brazil; MM Internal Medicine Department, MedStar Health, Washington, DC, USA; MN School of Public Health, The University of Queensland, Brisbane, QLD, Australia; MO Far Eastern University, Manila, Philippines; MP Medical Scientist Training Program, Northwestern University, Chicago, IL, USA; MQ Department of Dermatology, Carol Davila University of Medicine and Pharmacy, Bucharest, Romania; MR Board of Directors, Association of Resident Physicians, Bucharest, Romania; MS Department of Anatomy and Developmental Biology, Monash University, Clayton, VIC, Australia; MT Department of Anatomy, Genetics and Biomedical Informatics, University of Colombo, Colombo, Sri Lanka; MU Department of Prosthetic Dental Sciences, Jazan University, Jazan, Saudi Arabia; MV Research Division, The George Institute for Global Health, New Delhi, India; MW School of Medicine, University of New South Wales, Sydney, NSW, Australia; MX Department of Obstetrics and Gynaecology, Nnamdi Azikiwe University, Awka, Nigeria; MY Digital Health and Informatics Directorate, Queensland Health, Brisbane, QLD, Australia; MZ Department of Medical Laboratory Sciences, Adigrat University, Adigrat, Ethiopia; NA Dirección General de Investigación, Desarrollo e Innovación (DGIDI), Universidad Científica del Sur (University of the South), Lima, Peru; NB Department of Pathology - Forensic Medicine Division, Imam Abdulrahman Bin Faisal University, Dammam, Saudi Arabia; NC Department of Reproductive Health and Population Studies, Bahir Dar University, Bahir Dar, Ethiopia; ND University Centre Varazdin, University North, Varazdin, Croatia; NE Department of Paediatrics, University of Kelaniya, Ragama, Sri Lanka; NF University Paediatrics Unit, Colombo North Teaching Hospital, Ragama, Sri Lanka; NG Faculty of Sciences, University of Buea, Cameroon, Buea, Cameroon; NH Dermatology Unit, Fondazione IRCCS Policlinico San Matteo, Pavia, Italy; NI College of Human Medicine, Michigan State University, Flint, MI, USA; NJ Multidisciplinary Department of Medical-Surgical and Dental Specialties, University of Campania Luigi Vanvitelli, Naples, Italy; NK Saveetha Dental College and Hospitals, Saveetha University, Chennai, India; NL Faculty of Nursing and Midwifery, Tabriz University of Medical Sciences, Tabriz, Iran; NM Department of Forensic Medicine and Toxicology, All India Institute of Medical Sciences, Patna, India; NN RAK College of Nursing, RAK Medical and Health Sciences University, Ras Alkhima, United Arab Emirates; NO Nursing College, Sohag University, Sohag, Egypt; NP Molecular Biology Unit, Sirius Training and Research Centre, Khartoum, Sudan; NQ Bio-Statistical and Molecular Biology Department, Sirius Training and Research Centre, Khartoum, Sudan; NR Faculty of Medicine, University of Khartoum, Khartoum, Sudan; NS Social Determinants of Health Research Center, Tabriz University of Medical Sciences, Tabriz, Iran; NT Midwifery Department, Tabriz University of Medical Sciences, Tabriz, Iran; NU Modeling in Health Research Center, Shahrekord University of Medical Sciences, Shahrekord, Iran; NV Health Systems and Policy Research Unit, Ahmadu Bello University, Zaria, Nigeria; NW Heidelberg Institute of Global Health (HIGH), Heidelberg University, Heidelberg, Germany; NX Department of Biostatistics, Shiraz University of Medical Sciences, Shiraz, Iran; NY Clinical Epidemiology and Public Health Research Unit, Burlo Garofolo Institute for Maternal and Child Health, Trieste, Italy; NZ Department of Epidemiology and Biostatistics, Kurdistan University of Medical Sciences, Sanandaj, Iran; OA Department of Public Health, Oswaldo Cruz Foundation, Recife, Brazil; OB Department of Public Health, Federal University of Pernambuco, Recife, Brazil; OC Antimicrobial Resistance Research Center, Iran University of Medical Sciences, Tehran, Iran; OD Hazrat-e Rasool General Hospital, Iran University of Medical Sciences, Tehran, Iran; OE Unit of Pharmacotherapy, Epidemiology and Economics, University of Groningen (Rijksuniversiteit Groningen), Groningen, Netherlands; OF Department of Epidemiology and Biostatistics, Wuhan University, Wuhan, China; OG Department of Evidence and Intelligence for Action in Health, Pan American Health Organization, Washington, DC, USA; OH Department of Psychiatry, Seoul National University, Seoul, South Korea; OI Department of Neuropsychiatry, Seoul National University Bundang Hospital, Seongnam, South Korea; OJ ICMR-National Institute for Research in Tuberculosis, Indian Council of Medical Research, Chennai, India; OK Nursing & Midwifery Research Department (NMRD), Hamad Medical Corporation, Doha, Qatar; OL Division of Endocrinology and Diabetes, University of Vermont, South Burlington, VT, USA; OM Postgraduate Institute of Medicine, University of Colombo, Colombo, Sri Lanka; ON Manipal College of Nursing, Manipal Academy of Higher Education, Manipal, India; OO Department of Health Promotion, Zahedan University of Medical Sciences, Zahedan, Iran; OP Department of Management Science and Project Planning, University of Nairobi, Nairobi, Kenya; OQ School of Psychology, University College Dublin, Dublin, Ireland; OR Department of General Surgery, Carol Davila University of Medicine and Pharmacy, Bucharest, Romania; OS Department of General Surgery, Emergency University Hospital Bucharest, Bucharest, Romania; OT Institute for Global Health Innovations, Duy Tan University, Da Nang, Vietnam; OU Institute for Global Health Innovations, Duy Tan University, Hanoi, Vietnam; OV Faculty of Public Health, VNU University of Medicine and Pharmacy, Hanoi, Vietnam; OW International Institute for Training and Research (INSTAR), VNU University of Medicine and Pharmacy, Hanoi, Vietnam; OX Maternal and Child Health Division (MCHD), International Centre for Diarrhoeal Disease Research, Bangladesh, Dhaka, Bangladesh; OY Department of Microbiology and Molecular Genetics, The Women University Multan, Multan, Pakistan; OZ Bioprocess Engineering Department, National Institute of Genetic Engineering and Biotechnology, Tehran, Iran; PA Department of Paediatrics, Nnamdi Azikiwe University, Awka, Nigeria; PB Department of Nursing, University of Health and Allied Sciences, Ho, Ghana; PC School of Pharmacy, University of the Western Cape, Cape Town, South Africa; PD Department of Education Leadership and Management, University of Johannesburg, Johannesburg, South Africa; PE College of Health Sciences, Bowen University, Iwo, Nigeria; PF College of Medicine, University of Ibadan, Ibadan, Nigeria; PG Department of Public Health, Arcadia University, Glenside, PA, USA; PH Department of Global Health and Social Medicine, Harvard University, Boston, MA, USA; PI Wellspring Research, Wellspring Center Indonesia, Jakarta, Indonesia; PJ Department of Pharmacology and Therapeutics, University of Nigeria Nsukka, Enugu, Nigeria; PK Department of Neurosurgery, University of California San Francisco, San Francisco, CA, USA; PL One Health Global Research Group, Universidad de las Americas (University of the Americas), Quito, Ecuador; PM Department of Biological Sciences, Njala University, Freetown, Sierra Leone; PN School of Medicine, Western Sydney University, Bathurst, NSW, Australia; PO Department of Optometry and Vision Science, University of KwaZulu-Natal, KwaZulu-Natal, South Africa; PP Faculty of Medicine, University Ferhat Abbas of Setif, Setif, Algeria; PQ Division of Infectious Diseases, University Hospital of Setif, Setif, Algeria; PR Department of Respiratory Medicine, Jagadguru Sri Shivarathreeswara University, Mysore, India; PS Department of Forensic Medicine and Toxicology, Manipal Academy of Higher Education, Mangalore, India; PT Department of Mental Health, Hospital Universitari Vall d'Hebron (CIBERSAM), Barcelona, Spain; PU Biomedical Network Research Centre on Mental Health (CIBERSAM), Barcelona, Spain; PV Department of Emergency Medicine, University of Thessaly, Larissa, Greece; PW Department of Emergency Medicine, University of Bern, Bern, Switzerland; PX Division of Health Policy and Management, University of Minnesota, Minneapolis, MN, USA; PY Department of Psychiatry, All India Institute of Medical Sciences, Bhubaneswar, India; PZ School of Nursing, University of British Columbia, Vancouver, BC, Canada; QA Faculty of Medicine and Health, University of Leeds, Leeds, UK; QB Department of Research and Training, Population Council Institute, New Delhi, India; QC College of Dental Medicine, Roseman University of Health Sciences, South Jordan, UT, USA; QD Department of Genetics, Yale University, New Haven, CT, USA; QE Department of Interventional Cardiology, Cedars Sinai Medical Center, Los Angeles, CA, USA; QF Australian Institute of Health Innovation, Macquarie University, Sydney, NSW, Australia; QG Department of Data Management and Analysis, The INCLEN Trust International, New Delhi, India; QH Non-communicable Diseases Research Center, Bam University of Medical Sciences, Bam, Iran; QI Department of Humanities and Social Sciences, National Institute of Technology Rourkela, Rourkela, India; QJ Maternal and Child Health Research Centre, University of Bedfordshire, Luton, UK; QK Department of Biostatistics, Epidemiology, and Informatics, University of Pennsylvania, Philadelphia, PA, USA; QL Department of Neonatology, Case Western Reserve University, Akron, OH, USA; QM Rory Meyers College of Nursing, New York University, New York, NY, USA; QN School of Public Health, Xuzhou Medical University (徐州医科大学公共卫生学院), Xuzhou, China; QO Department of Epidemiology, Shandong University, Jinan, China; QP Faculty of Veterinary Medicine, University of Ilorin, Ilorin, Nigeria; QQ Sina Trauma and Surgery Research Center, Tehran University of Medical Sciences, Tehran, Iran; QR Department of Population Science and Human Resource Development, University of Rajshahi, Rajshahi, Bangladesh; QS Institute of Health and Wellbeing, Federation University Australia, Berwick, VIC, Australia; QT Department of Medical, Surgical and Experimental Sciences, University of Sassari, Sassari, Italy; QU Gynecology and Breast Care Center, Mater Olbia Hospital, Olbia, Italy; QV Department of Community Medicine, Employees' State Insurance Model Hospital, Chennai, India; QW Department of Clinical Sciences, University of Sharjah, Sharjah, United Arab Emirates; QX Department of Cardiology, Mansoura University, Mansoura, Egypt; QY School of Nursing & Health Sciences, Hong Kong Metropolitan University, Hong Kong, China; QZ Saw Swee Hock School of Public Health, National University of Singapore, Singapore, Singapore; RA Department of Research, Eastern Scientific LLC, Richmond, KY, USA; RB Planetary Health Research Centre (PHRC), Kathmandu, Nepal; RC Department of Oral Pathology, Microbiology and Forensic Odontology, Sharavathi Dental College and Hospital, Shimogga, India; RD Department of Family Medicine, Rajarata University of Sri Lanka, Anuradhapura, Sri Lanka; RE Department of Global Health Policy, University of Tokyo, Tokyo, Japan; RF Department of Epidemiology and Biostatistics, Rafsanjan University of Medical Sciences, Rafsanjan, Iran; RG Department of Public Health Sciences, University of Connecticut, Farmington, CT, USA; RH Department of Psychiatry, Yale University, New Haven, CT, USA; RI Department of Pharmacology and Toxicology, University of Antioquia, Medellin, Colombia; RJ Warwick Medical School, University of Warwick, Coventry, UK; RK Department of Clinical Research, University of Sao Paulo, Ribeirão Preto, Brazil; RL Gilbert and Rose-Marie Chagoury School of Medicine, Lebanese American University, Beirut, Lebanon; RM Miyan Research Institute, International University of Business Agriculture and Technology, Dhaka, Bangladesh; RN College of medicine, Ajman University, Ajman, United Arab Emirates; RO Isfahan University of Medical Sciences, Islamic Azad University, Isfahan, Iran; RP Department of Ophthalmology, University of Miami, Miami, FL, USA; RQ Department of Labour, Government of West Bengal, Kolkata, India; RR College of International Studies, Korea University, Seoul, South Korea; RS Faculty of Education, University of Canterbury, Christchurch, New Zealand; RT Department of Medicine, Georgetown University, Washington, DC, USA; RU Department of Biology, University of Hail, Hail, Saudi Arabia; RV Széchenyi István University, Győr, Hungary; RW Operational Research Center in Healthcare, Near East University, Cyprus, Turkiye; RX Department of Psychiatry, All India Institute of Medical Sciences, New Delhi, India; RY Department of Psychosocial Science, University of Bergen, Bergen, Norway; RZ Department of Analytical and Applied Economics, Utkal University, Bhubaneswar, India; SA College of Pharmacy, Al-Hadba University, Mosul, Iraq; SB Department of Psychiatric and Mental Health, and Community Health, Qassim University, Buraydah, Saudi Arabia; SC Department of Community Health Sciences, Aga Khan University, Karachi, Pakistan; SD Department of Entomology, Ain Shams University, Cairo, Egypt; SE Medical Ain Shams Research Institute (MASRI), Ain Shams University, Cairo, Egypt; SF Queensland Centre for Mental Health Research, Wacol, QLD, Australia; SG Faculty of Medicine, University of Belgrade, Belgrade, Serbia; SH School of Public Health and Health Management, University of Belgrade, Belgrade, Serbia; SI Department of Food Processing Technology, West Bengal State Council of Technical Education, Malda, India; SJ Department of Oral Pathology and Microbiology, Dr. D. Y. Patil Vidyapeeth, Pune (Deemed to be University), Pune, India; SK UGC Centre of Advanced Study in Psychology, Utkal University, Bhubaneswar, India; SL Udyam-Global Association for Sustainable Development, Bhubaneswar, India; SM Department of Public Health Sciences, University of North Carolina at Charlotte, Charlotte, NC, USA; SN Faculty of Dentistry, University of Puthisastra, Phnom Penh, Cambodia; SO Dr. D. Y. Patil Dental College & Hospital, Dr. D. Y. Patil Vidyapeeth, Pune (Deemed to be University), Pune, India; SP Department of Medicine, Swami Vivekanand Subharti University, Meerut, India; SQ Dongguan Key Laboratory of Computer-Aided Drug Design, Guangdong Medical University, Dongguan, China; SR State Key Laboratories of Chemical Resources Engineering, Beijing University Of Chemical Technology, Beijing, China; SS Research Centre for Health Sciences (RCHS), The University of Lahore, Lahore, Pakistan; ST Center for Medical and Bio-Allied Health Sciences Research, Ajman University, Ajman, United Arab Emirates; SU Independent Consultant, Karachi, Pakistan; SV Department of Medicine, Liaquat University Of Medical and Health Sciences, Jamshoro, Pakistan; SW Centre For Interdisciplinary Research In Basic Sciences (CIRBSc), Jamia Millia Islamia, New Delhi, India; SX Science Department, Kazakh National Medical University, Almaty, Kazakhstan; SY Lancaster University, Lancaster, UK; SZ Columbia University, New York, NY, USA; TA College of Nursing and Health Sciences, Jazan University, Jazan, Saudi Arabia; TB Amity Institute of Public Health and Hospital Administration, Amity University, Noida, India; TC Institute of Forensic Science & Criminology, Panjab University, Chandigarh, India; TD Alva's Institute of Medical Sciences & Research Centre, Rajiv Gandhi University of Health Sciences, Moodubidire, India; TE Manipal College of Dental Sciences, Mangalore, Manipal Academy of Higher Education, Manipal, India; TF School of Public Health, University of Hong Kong, Hong Kong, China; TG Department of Public Health, Daffodil International University, Dhaka, Bangladesh; TH Finnish Institute of Occupational Health, Helsinki, Finland; TI Department of Veterinary Public Health and Preventive Medicine, Usmanu Danfodiyo University, Sokoto, Sokoto, Nigeria; TJ Oulu Business School, University of Oulu, Oulu, Finland; TK Martti Ahtisaari Institute, University of Oulu, Oulu, Finland; TL Department of Medical-Surgical Nursing, Mazandaran University of Medical Sciences, Sari, Iran; TM Department of Nursing and Health Sciences, Flinders University, Adelaide, SA, Australia; TN Unit of Basic Medical Sciences, University of Khartoum, Khartoum, Sudan; TO Department of Medical Microbiology and Infectious Diseases, Erasmus University, Rotterdam, Netherlands; TP Faculty of Dentistry, Federal University of Minas Gerais, Belo Horizonte, Brazil; TQ Department of Biochemistry, Central University of Punjab, Bathinda, India; TR Universidade Federal de Minas Gerais, Federal University of Minas Gerais, Belo Horizonte, Brazil; TS Department of Public Health, Kandahar University, Kandahar, Afghanistan; TT SAMRC Unit on Risk and Resilience in Mental Disorders, University of Cape Town, Cape Town, South Africa; TU Department of Psychological Medicine, King's College London, London, UK; TV Department of Sport, University of Vienna, Vienna, Austria; TW Department of Medical Sciences, Sunway University, Subang Jaya, Malaysia; TX Hospital Administration, Sanjay Gandhi Postgraduate Institute of Medical Sciences, Lucknow, India; TY Hospital Administration, King George's Medical University, Lucknow, India; TZ Department of Life and Health Sciences, University of Nicosia, Nicosia, Cyprus; UA Institute of Integrated Intelligence and Systems, Griffith University, Brisbane, QLD, Australia; UB Department of Pharmacology, All India Institute of Medical Sciences, Deoghar, India; UC Department of Medicine, University of Valencia, Valencia, Spain; UD Carlos III Health Institute, Biomedical Research Networking Center for Mental Health Network (CiberSAM), Madrid, Spain; UE Department of Medical Informatics, Mashhad University of Medical Sciences, Mashhad, Iran; UF Applied Biomedical Research Center, Mashhad University of Medical Sciences, Mashhad, Iran; UG Department of Primary Care and Public Health, Imperial College London, London, UK; UH Department of Dermato-Venereology, Dr. Victor Babes Clinical Hospital of Infectious Diseases and Tropical Diseases, Bucharest, Romania; UI Department of Psychiatry, Bahir Dar University, Bahir Dar, Ethiopia; UJ Department of Psychology, Montclair State University, Montclair, NJ, USA; UK Department of Public Health and Informatics, Bangladesh Medical University, Dhaka, Bangladesh; UL Pediatric Intensive Care Unit, King Saud University, Riyadh, Saudi Arabia; UM College of Medicine, Alfaisal University, Riyadh, Saudi Arabia; UN Department of Applied Bioscience, Konkuk University, Seoul, South Korea; UO School of Public Health, Harbin Medical University, Harbin, China; UP John T. Milliken Department of Medicine, Washington University in St. Louis, Saint Louis, MO, USA; UQ Department of Internal Medicine, University of Medicine and Pharmacy at Ho Chi Minh City, Ho Chi Minh City, Vietnam; UR Department of Business Analytics, University of Massachusetts Dartmouth, Dartmouth, MA, USA; US Division of Public Health and Epidemiology, University of Leicester, Leicester, UK; UT Adult Learning Disability Service, Leicestershire Partnership National Health Service Trust, Leicester, UK; UU CRIMEDIM Center for Research and Training in Global Health, Humanitarian Aid and Disaster Medicine, University of Eastern Piedmont, Novara, Italy; UV Department of Primary Care, Geneva University Hospital, Geneva, Switzerland; UW Department of Psychiatry, Massachusetts General Hospital, Boston, MA, USA; UX Harvard Medical School, Harvard University, Boston, MA, USA; UY Kasturba Medical College, Mangalore, Manipal Academy of Higher Education, Manipal, India; UZ Department of Psychology, Zayed University, Abu Dhabi, United Arab Emirates; VA Department of Preventive and Social Medicine, Federal University of Minas Gerais, Belo Horizonte, Brazil; VB Department of Exact and Applied Social Sciences, Federal University of Health Science of Porto Alegre, Porto Alegre, Brazil; VC Digital Health Research Center, Instituto Peruano de Orientación Psicológica, Lima, Peru; VD Department of Biomedical Informatics, University of Utah, Salt Lake City, UT, USA; VE Department of Cardiology, Icahn School of Medicine at Mount Sinai, New York, NY, USA; VF NUST School of Health Sciences, National University of Science and Technology (NUST), Islamabad, Pakistan; VG Széchenyi István University, Gyor, Hungary; VH Department of Forensic Science, Shree Guru Gobind Singh Tricentenary University, Gurugram, India; VI School of Public Health, Xuzhou Medical University, Xuzhou, China; VJ Department of Community Medicine, Rajarata University of Sri Lanka, Anuradhapura, Sri Lanka; VK Legon Centre for Education Research and Policy, University of Ghana, Accra, Ghana; VL Department of Intelligent Medical Engineering, Anhui Medical University, Anhui, China; VM Department of Surgery, The First Affiliated Hospital of Anhui Medical University, Hefei, Anhui, China; VN Department of Nutrition, Tufts University, Boston, MA, USA; VO Department of Social and Behavioral Sciences, Harvard University, Boston, MA, USA; VP Department of Community Medicine, Apollo Institute of Medical Sciences and Research, Hyderabad, India; VQ Shanghai Institute of Infectious Disease and Biosecurity, Fudan University, Shanghai, China; VR National Center for Chronic and Noncommunicable Disease Control and Prevention, Chinese Center for Disease Control and Prevention, Beijing, China; VS The George Institute for Global Health, University of New South Wales, Sydney, NSW, Australia; VT Manipal College of Nursing, Manipal Academy of Higher Education, Udupi, India; VU KHANA Center for Population Health Research, Phnom Penh, Cambodia; VV Department of Pharmacology, Bahir Dar University, Bahir Dar, Ethiopia; VW Pharmacy Department, Alkan Health Science, Business and Technology College, Bahir Dar, Ethiopia; VX Department of Pediatrics, Kyung Hee University, Seoul, South Korea; VY Department of Biostatistics, University of Toyama, Toyama, Japan; VZ Department of Public Health, Juntendo University, Tokyo, Japan; WA Department of Nursing Science, Bayero University, Kano, Nigeria; WB Faculty of Nursing, University of Alberta, Edmonton, AB, Canada; WC Association for Socially Applicable Research (ASAR), Pune, India; WD Department of Emergency Medicine, Global Emergency Medicine Innovation and Implementation (GEMINI) Research Center, Durham, NC, USA; WE The Heller School for Social Policy and Management, Brandeis University, Waltham, MA, USA; WF Department of Public Health, University of Hail, Hail, Saudi Arabia; WG Department of Pediatrics and Child Health Nursing, Woldia University, Woldia, Ethiopia; WH Department of Cardiology, Zhongshan Hospital, Shanghai, China; WI School of Public Health, Peking University, Beijing, China; WJ Department of International Health, Johns Hopkins University, Baltimore, MD, USA; WK Atchabarov Scientific-Research Institute of Fundamental and Applied Medicine, Kazakh National Medical University, Almaty, Kazakhstan; WL Department of Suicide Research and Prevention, Moscow Research and Clinical Center for Neuropsychiatry, Moscow, Russia; WM College of Nursing, Prince Sattam bin Abdulaziz University, Al-Kharj, Saudi Arabia; WN Faculty of Nursing, Mansoura University, Mansoura, Egypt; WO Department of Clinical and Community Pharmacy, An-Najah National University, Nablus, Palestine; WP Clinical Research Centre, An-Najah National University Hospital, Nablus, Palestine

## Abstract

**Background:**

Violence against women and against children are human rights violations with lasting harms to survivors and societies at large. Intimate partner violence (IPV) and sexual violence against children (SVAC) are two major forms of such abuse. Despite their wide-reaching effects on individual and community health, these risk factors have not been adequately prioritised as key drivers of global health burden. Comprehensive x§and reliable estimates of the comparative health burden of IPV and SVAC are urgently needed to inform investments in prevention and support for survivors at both national and global levels.

**Methods:**

We estimated the prevalence and attributable burden of IPV among females and SVAC among males and females for 204 countries and territories, by age and sex, from 1990 to 2023, as part of the Global Burden of Diseases, Injuries, and Risk Factors Study 2023. We searched several global databases for data on self-reported exposure to IPV and SVAC and undertook a systematic review to identify the health outcomes associated with each of these risk factors. We modelled IPV and SVAC prevalence using spatiotemporal Gaussian process regression, applying data adjustments to account for measurement heterogeneity. We employed burden-of-proof methodology to estimate relative risks for outcomes associated with IPV and SVAC. These estimates informed the calculation of population attributable fractions, which were then used to quantify disability-adjusted life-years (DALYs) attributable to each risk factor.

**Findings:**

Globally, in 2023, we estimated that 608 million (95% uncertainty interval 518–724) females aged 15 years and older had ever been exposed to IPV, and 1·01 billion (0·764–1·48) individuals aged 15 years and older had experienced sexual violence during childhood. 18·5 million (8·74–30·0) DALYs were attributed to IPV among females and 32·2 million (16·4–52·5) DALYs were attributed to SVAC among males and females in 2023. IPV and SVAC were among the top contributors to the global disease burden in 2023, particularly among females aged 15–49 years, ranking as the fourth and fifth leading risk factors, respectively, for DALYs in this group. Among the eight health outcomes found to be associated with IPV, anxiety disorders and major depressive disorder were the leading causes of IPV-attributed DALYs, accounting for 5·43 million (–1·25 to 14·6) and 3·96 million (1·71 to 6·92) DALYs in 2023, respectively. SVAC was associated with 14 health outcomes, including mental health disorder, substance use disorder, and chronic and infectious disease outcomes. Self-harm and schizophrenia were the leading causes of SVAC-attributed burden, with SVAC accounting for 6·71 million (2·00 to 12·7) DALYs due to self-harm and 4·15 million (–1·92 to 13·1) DALYs due to schizophrenia in 2023.

**Interpretation:**

IPV and SVAC are substantial contributors to global health burden, and their health consequences span a variety of individual health outcomes. Importantly, mental health disorders account for the greatest share of disease burden among survivors. Investing in prevention of these avoidable risk factors has the potential to avert millions of DALYs and considerable premature mortality each year. Our findings represent strong evidence for global and national leaders to elevate IPV and SVAC among public health priorities. Sustained investments are needed to prevent IPV and SVAC and to implement interventions focused on supporting the complex social and health needs of survivors.

**Funding:**

Gates Foundation.

## Introduction

Violence against women and violence against children constitute individual human rights violations and, collectively, represent an underestimated global health crisis. Intimate partner violence (IPV) and sexual violence against children (SVAC) are major forms of such abuse, with both immediate and long-lasting effects on health and wellbeing. According to WHO and UNICEF, nearly one-third of ever-partnered women have experienced physical or sexual IPV,^1^ while almost one in five women and one in seven men have suffered sexual abuse before the age of 18 years.^2^, ^3^

**Research in context**

**Evidence before this study**

Numerous individual and meta-analytic studies have assessed the magnitude of intimate partner violence (IPV) and sexual violence against children (SVAC), providing essential insights into their global prevalence. WHO estimates that 27% of ever-partnered women aged 15–49 years have experienced physical or sexual IPV, while UNICEF estimates that 1 in 5 girls and women and 1 in 7 boys and men alive today have been subjected to sexual violence as children. Country-level prevalence estimates, however, remain limited by data scarcity. Furthermore, the health effects of IPV and SVAC have been mostly analysed at the individual level, with several meta-analyses finding a link between these exposures and various health outcomes. Most recently, a comprehensive systematic review and meta-analysis effort spanning data from 1970 to 2023, which used robust relative risk estimation techniques, found highly significant and consistent associations between IPV and SVAC and a wide range of adverse outcomes, including mental health conditions, physical injuries, and HIV. However, there is a notable lack of timely and detailed research on the overall population-level health effects. Previous analyses from the Global Burden of Diseases, Injuries, and Risk Factors Study (GBD) have connected IPV and SVAC with a limited number of outcomes, thereby underestimating their full health burden.

**Added value of this study**

We provide comprehensive and timely estimates of the disease burden attributable to IPV and SVAC for 204 locations. This study updates and expands previous estimates produced as part of GBD by incorporating several methodological improvements, including updated data sources, solutions to differential reporting challenges, and a systematic evaluation of new risk–outcome associations. Specifically, compared with the previous GBD cycle (GBD 2021), we incorporated 195 new sources of data to refine IPV prevalence estimates and an additional 211 sources to enhance SVAC prevalence estimates, thereby strengthening the data foundation of our analyses. We also introduced substantive methodological advances in our SVAC exposure estimation process. In particular, we revised the definition of SVAC—extending the age range of exposure from before 15 years to before 18 years—to align with international classifications of violence against children. To address a persistent challenge in violence research, we also adjusted our SVAC estimates for differential reporting across survey modes. In addition, our systematic reviews and meta-analyses enabled the assessment of a greater number of long-term health outcomes linked to IPV and SVAC, expanding upon those included in previous GBD rounds. Risk–outcome pairs were added in GBD 2023 based on data-driven determination of a risk–outcome association. For IPV, we included five additional causes of health burden, for a total of eight health outcomes. Similarly, for SVAC, the number of associated health outcomes increased from two in GBD 2021 to 14 in GBD 2023. Together, these improvements address some of the major limitations of estimating the disease burden attributable to SVAC and IPV in earlier GBD iterations and enhance our understanding of the magnitude of the health effects associated with these risks.

**Implications of all the available evidence**

Quantifying the disease burden attributable to IPV and SVAC is essential for enabling timely and effective interventions. Moreover, by leveraging the GBD comparative framework, we position IPV and SVAC alongside other major health threats, moving beyond viewing them solely as social or criminal concerns. Our results indicate that these risks contribute to a range of fatal and non-fatal outcomes and affect populations worldwide, regardless of their development status, and are particularly detrimental to young and middle-aged individuals. Given that coordinated responses can mitigate these risks, it is imperative to implement comprehensive prevention strategies to reduce the occurrence of IPV and SVAC, alongside multipronged support systems to address the complex recovery and healing needs of survivors. We strongly advocate integrating both prevention measures and survivor support into broader public health initiatives that also address mental health disorders, substance misuse, suicide, homicide, and HIV. Prioritising these risks in the global health agenda is essential for promoting global sustainability and protecting future generations. In addition, it should be acknowledged that our understanding of the scope of this problem continues to be limited by data sparsity on all forms of violence, including less studied forms, such as emotional, economic and reproductive abuse, and IPV against males. There is a continued need for further data collection and modelling efforts to more fully capture the true health impacts of violence against women and children.

Exposure to IPV and to SVAC have been linked to a wide range of health conditions—with both fatal and non-fatal consequences—including physical injuries, chronic diseases, reproductive health issues, and several mental health disorders.^4^, ^5^, ^6^, ^7^, ^8^ Moreover, the repercussions extend beyond individual survivors to affect families and communities, fostering cycles of violence and intergenerational trauma.^9^, ^10^ These adverse effects, along with corresponding social and economic costs, undermine collective wellbeing, human capital, and development potential.^11^, ^12^, ^13^

The response to violence against women and children has been a critical concern for international organisations, including WHO, the UN Entity for Gender Equality and the Empowerment of Women (UN Women), and UNICEF. Over the past decade, these organisations have developed comprehensive strategies and frameworks to assist member states in implementing evidence-based interventions that address the lifelong health needs of survivors and protect future generations.^14^, ^15^, ^16^ Interventions include, among others, establishing legal frameworks, promoting gender equitable norms, values, and relationships, and strengthening health system capacity to support survivors.^17^ However, most countries still do not have the essential resources, effective legislation, and robust enforcement mechanisms necessary to combat violence,^18^, ^19^ threatening the achievement of the UN's Sustainable Development Goals (SDGs) aimed at eliminating violence against women and girls and promoting peaceful societies.^20^ This gap underscores the urgent need for increased and accelerated advocacy, funding, and collaboration among governments, civil society, and international bodies to ensure that both prevention and intervention measures are adequately funded, implemented, and effectively integrated into national policies and practices.

Accurate and reliable estimates of violence against women and children, as well as their health consequences, are essential to guide both global and regional response efforts and for motivating investment in prevention and effective, multipronged support to survivors. Although IPV and SVAC have been incorporated into the Global Burden of Diseases, Injuries, and Risk Factors Study (GBD) since 2010, previous estimates have underestimated their health burden, primarily due to the small number of outcomes identified as being associated with these risks. For GBD 2023, we conducted systematic reviews and meta-analyses that have allowed us to improve these estimates by identifying additional health outcomes associated with IPV and SVAC and by reassessing the magnitude of these health risks.^4^ This study examines the current global patterns of IPV and SVAC and their attributable disease burdens and provides estimates for 204 countries and territories, with analyses stratified by age and sex. The research presented in this Article has contributed to the work of the *Lancet* Commission on gender-based violence and maltreatment of young people,^21^ and was produced as part of the GBD Collaborator Network and in accordance with the GBD Protocol.^22^

## Methods

### Overview

For GBD 2023, we estimated the disease burden attributable to IPV against females and to SVAC among males and females for 204 countries and territories, stratified by age and sex, from 1990 to 2023. Our IPV case definition is the lifetime experience of at least one act of physical or sexual violence by a current or former intimate partner since the age of 15 years among females. This operational definition is consistent with how WHO measures and reports on IPV. Notably, it does not encompass psychological abuse or coercive control, reflecting the lack of a global consensus on measuring and defining psychological forms of partner violence^23^, ^24^ as well as the lack of sufficient evidence to robustly quantify their health risks in the context of GBD; our assessment criteria require at least three studies linking a given exposure to a health outcome. Similarly, while IPV affects both males and females, our estimation is restricted to females due to equally insufficient evidence to quantify health risks among males.

SVAC exposure is estimated for both males and females aged 15 years and older retrospectively reporting abuse that occurred in childhood, with health burden estimates reflecting the detrimental effects of such early-life exposure to violence in adulthood. We defined SVAC as the lifetime prevalence of intercourse or other sexual contact (ie, fondling and other sexual touching) before the age of 18 years, where the contact was unwanted (ie, physically forced or coerced). In GBD 2023, we updated the case definition of SVAC by expanding the age range of exposure from before 15 years to before 18 years to ensure consistency with international standards for classifying violence against children, specifically the International Classification of Violence Against Children framework.^25^ Notably, with this change, we recognise that females aged 15–17 years might experience violence that meets the case definitions for both IPV and SVAC—for example, sexual intimate partner violence experienced at age 16 years. However, our use of the comparative risk assessment (CRA) framework^26^ allows for this overlap in case definitions and prevalence models by assessing the independent contribution of each risk factor to global health burden.

In this analysis, we focus on describing what is currently known about these health risks and summarise the following key CRA analytical steps below: (1) estimating prevalence by age-sex-year-location; (2) assessing the relative risk of health outcomes associated with exposure to each risk factor; and (3) calculating population attributable fractions (PAFs) and attributable disability-adjusted life-years (DALYs) and deaths. Appendix 1 provides more details on each of these steps. This study adheres to the GATHER statement (appendix 1 section 1).^27^ GBD 2023 is registered with the University of Washington Institutional Review Board (study number 9060).

### Data sources

To gather data on exposure to IPV or SVAC in line with GBD case definitions—or acceptable alternatives (appendix 1 section 2.1)—we searched three comprehensive databases for unique data sources: the Global Health Data Exchange (GHDx), the WHO Global Database on the Prevalence of Violence Against Women, and the UN Women Global Database on Violence Against Women. Each database used targeted and systematic search strategies to identify cross-sectional data on violence against women and children (including but not limited to IPV among women and SVAC among males and females), yielding a thorough compilation of sources that span multiple time periods, geographies, and populations. Further details on the databases and underlying search methodologies are provided in appendix 1 (section 2.1).

After searching the databases described above, we systematically identified and extracted data spanning from 1980 to 2023 that met the following inclusion criteria: (1) population-based studies; (2) samples representative of national or subnational locations modelled in GBD; and (3) measurement of self-reported instances of violence. For each country, all identified unique sources meeting these criteria were included in our models, regardless of overlap in country-age-year representation, to maximise coverage and robustness, in accordance with the standard GBD approach. Additional information on identification of sources, inclusion and exclusion criteria, and data extraction is provided in appendix 1 (section 2.1).

Ultimately, we identified and extracted information from 594 sources across 169 countries for IPV and 460 sources from 141 countries for SVAC. Of these, 195 and 211 are new sources that were added to GBD 2023 to enhance our IPV and SVAC models, respectively. These sources include major survey series like the Demographic and Health Surveys (DHS), Multiple Indicator Cluster Surveys (MICS), and Violence against Children and Youth Surveys, which provide comparable, cross-national data on the prevalence of IPV and SVAC due to their often-standardised survey instruments and methodologies. A full list of sources is provided in appendix 1 (section 2.1.4). The total number of sources and year of the most recent input data for each country are also shown in appendix 1 (section 2.1.5; figures S1–S4).

### IPV modelling strategy

For IPV prevalence, we implemented a modelling strategy similar to that used in the previously published GBD 2021 risk factors capstone Article.^28^ To maximise the use of available data and ensure comparability across all data included in our model, we implemented three data adjustment steps. First, we used the meta-regression Bayesian, regularised, trimmed (MR-BRT) tool to adjust data from sources reporting alternative, non-reference case definitions (eg, experiencing physical IPV in the past year only). Drawing upon both direct and indirect within-study comparisons, we conducted a network meta-analysis to estimate age-varying adjustment factors for non-reference definitions (appendix 1 section 2.2.1). Second, for data reported in aggregated age groups, we split them into GBD standard 5-year age groups using an age pattern derived exclusively from data reported in these standard age intervals (appendix 1 section 2.2.2). Finally, we corrected for studies reporting IPV prevalence solely among ever-partnered or currently partnered women by multiplying estimates from these studies by the age-location-year-specific fraction of women who had ever been partnered. These fractions were calculated using data from MICS and DHS within a single parameter DisMod-MR 2.1 model,^29^ a Bayesian mixed-effects meta-regression modelling tool developed specifically for GBD analyses (appendix 1 section 2.2.3).

After applying all data adjustments, we used spatiotemporal Gaussian process regression (ST-GPR),^26^ a three-stage model designed to estimate time-varying risk factors, in conjunction with Holt's exponential smoothing method, to produce IPV prevalence time series with uncertainty for females by age and location. Importantly, in areas where data are scarce, ST-GPR can create implausible trends over time by overfitting observed datapoints. To address this limitation, we introduced Holt's exponential smoothing method, which generates smoothed trends and dampened forecasts by applying a weighted moving average to past observations, with weights that decrease exponentially for older data. Further details on these modelling steps are provided in appendix 1 (section 2.2.4).

### SVAC modelling strategy

The methods used to estimate SVAC prevalence, along with detailed results, have been published elsewhere^2^ and are further elaborated in appendix 1 (section 2.3). Briefly, we applied three data adjustments before modelling. First, we used the MR-BRT tool to adjust data reported from surveys using non-reference case definitions (appendix 1 section 2.3.1). Second, recognising the impact of survey administration methods on disclosure rates, we adjusted data from face-to-face interviews using linear regression (appendix 1 section 2.3.2). This step marks an improvement over previous SVAC estimations within GBD, acknowledging the increased rates of disclosure associated with confidential self-reports compared with direct interviews. Third, we disaggregated data reported in aggregated age groups into GBD standard 5-year age categories (appendix 1 section 2.3.3). Beyond these adjustments, we introduced a cohort extrapolation step to refine our input data further (appendix 1 section 2.3.4). Upon completing all adjustments and data extrapolation, we used ST-GPR and Holt's exponential smoothing method to estimate the prevalence of SVAC by age, sex, location, and year (appendix 1 section 2.3.5).

### Relative risk estimation

Relative risk (RR) estimates were generated for outcomes associated with exposure to IPV and SVAC analysed in previous GBD rounds, as well as for additional outcomes considered for inclusion in the GBD framework. The selection of new outcomes was guided by the availability of sufficient data and evidence of association, as determined by our systematic review described below. This process followed the extensively peer-reviewed standard GBD approach established in previous rounds.^28^, ^30^

The burden-of-proof meta-analytical approach^4^, ^28^, ^30^ was used to generate RR estimates by synthesising data identified and extracted through a comprehensive systematic review of seven electronic databases (PubMed, Embase, CINAHL, PsycINFO, Global Index Medicus, Cochrane, and Web of Science Core Collection) for all relevant studies published between Jan 1, 1970 and Jan 31, 2024. This review, conducted in accordance with PRISMA guidelines,^31^ was part of a broader project aimed at identifying and synthesising all existing cohort, case–control, and longitudinal data on the health consequences of any form of violence against women, gender-based violence, and violence against children.^32^ Meta-analyses for each risk–outcome pair required data from at least three studies, with health outcomes defined according to GBD criteria.^29^ Outcomes reported in the literature as subcomponents of GBD definitions were mapped to the closest corresponding GBD outcome; for example, studies reporting risk for post-traumatic stress disorder were included under anxiety disorders. Subcomponent outcome definitions were accounted for as study-level bias covariates when possible.

For IPV, the availability of evidence on associated health risks contributed to our decision to restrict our estimation to physical or sexual IPV among females only. We identified only one health outcome (major depressive disorder) with the minimum number of studies conducted among males, and no significant association was found based on the available evidence to date. We further found fewer than three studies with data on male survivors of IPV for alcohol use disorder, anxiety, drug use disorders, HIV/AIDS, and self-harm, which are not sufficient data to inform our meta-analytical approach. We identified only four health outcomes (major depressive disorder, abortion and miscarriage, drug use disorders, and self-harm) that had sufficient evidence to assess associations with psychological IPV for male and female survivors, among which only major depressive disorder showed a significant association with psychological IPV.^33^

The methodologies used, along with previous IPV-specific and SVAC-specific results, have been published elsewhere,^4^ although we have since updated these searches and results to include newly published evidence from Feb 1, 2023 to Jan 31, 2024. These updated searches, as well as detailed systematic review methods are provided in appendix 1 (section 3.1). The burden-of-proof^30^ meta-analytical steps used to estimate RRs are also summarised in appendix 1 (section 3.2). In brief, burden-of-proof methods systematically apply covariate selection and adjustment to account for known variation in input study characteristics—such as a lack of representativeness, different strategies for ascertaining exposure, varying levels of control for confounders, and non-gold-standard outcome definitions that might bias results^34^—and quantify and incorporate remaining unexplained between-study heterogeneity into estimates of uncertainty. This incorporation of between-study heterogeneity into uncertainty around RRs results in uncertainty intervals (UIs) that are considerably wider than those calculated with conventional risk analysis methods. As delineated below, RRs and their UIs provide the foundation for calculating PAFs and risk-attributable burden, along with their associated UIs. In cases where the RR UI is below the null (ie, 0, for natural log RR), due to low RR or wide UIs, this detail will get propagated forward, potentially resulting in negative lower UI bounds for risk-attributable burden. The risk–outcome relationships used in GBD 2023, along with information on the studies included in the analyses, are available in the Burden of Proof visualisation tool.

### Attributable burden estimation

To estimate PAFs, we used three overarching strategies. First, for all associated health outcomes identified via the systematic review, excluding HIV/AIDS, we calculated PAFs using three key metrics: prevalence, RRs, and the theoretical minimum risk exposure level (TMREL; the theoretically possible level of exposure that minimises disease risk). For both IPV and SVAC, the TMREL is zero. The PAF equation for dichotomous risk factors is defined as

$${\text{PAF}}_{joasgt}=\frac{{\sum_{x}^{u}{=}_{l}\text{RR}}_{joasg}(x){\text{P}}_{jasgt}(x)-{\text{RR}}_{joasg}({\text{TMREL}}_{jas})}{{\sum_{x}^{u}{=}_{l}\text{RR}}_{joasg}(x){\text{P}}_{jasgt}(x)}$$

where PAF_joasgt_ is the PAF for cause *o* due to risk factor *j* for age group *a*, sex *s*, location *g*, and year *t*. RR_joasg_(*x*) is the RR as a function of exposure level *x* for risk factor *j* for cause *o*, age group *a*, sex *s*, and location *g* on a plausible range of exposure levels from *l* to *u*; P_jasgt_(*x*) is the proportion of the population in risk group (prevalence) for age group *a*, sex *s*, location *g*, and year *t*; and TMREL_jas_ is the TMREL for risk factor *j*, age group *a*, and sex *s*.

To measure the burden of HIV/AIDS attributable to IPV and SVAC, we adopted a cohort method whereby HIV risk associated with past exposure to IPV and SVAC is accumulated over time. This method is consistent with approaches used in GBD to estimate risk–outcome pairs with similar temporal dynamics, such as intravenous drug use and hepatitis. For PAF calculation, we estimated the history of exposure and the resulting accumulated risk of incident HIV in birth cohorts for each country from 1980 to 2023. This required a time series, spanning 1980–2023, of the following for each GBD country, year, and 5-year age group: IPV or SVAC prevalence; HIV incidence; the proportion of HIV incidence that is from sexual transmission for each GBD country; and a pooled incidence rate ratio for the risk of incident HIV, given exposure to IPV or SVAC. The denominator of the PAF was the cumulative overall incidence of HIV after age 15 years, while the numerator was the cumulative incidence of HIV attributable to IPV or SVAC, calculated as the product of the PAF on HIV incidence and the sexually transmitted HIV incidence rate (appendix 1 section 4).

Lastly, we calculated the burden of homicides and injuries attributable to IPV as direct PAFs. Although IPV is a substantial contributor to these outcomes, immediate injuries and fatalities directly resulting from violence are not adequately captured by longitudinal and cohort-based study designs, which were a primary focus of our systematic review. Further, our understanding of the association between IPV and injuries is often limited by the paucity of perpetrator information in routinely collected clinical data. Conversely, homicide datasets provide comparatively more information on perpetrator distribution. Therefore, to estimate the proportion of homicides and injuries attributable to IPV, we systematically reviewed the GHDx for data on the proportion of female homicides committed by intimate partners. Subsequently, we derived location-age-specific fractions of homicides perpetrated by intimate partners using a single-parameter DisMod-MR 2.1 model. Due to the aforementioned data limitations, these model estimates were applied as PAFs for all GBD interpersonal violence injury outcomes (deaths and injuries resulting from physical violence by firearm, sharp objects, and other means).

To arrive at measures of risk-attributable burden—ie, the disease burden attributable to IPV and to SVAC—we multiplied PAFs by the estimated DALYs and deaths associated with the outcomes linked with each risk, according to the standard GBD approach.

### Reporting of estimates

Our modelling processes generate age-sex-location-year estimates in 5-year age groups. SVAC and IPV exposure and attributable burden estimates are generated for those aged 15 years and older. To ensure comparability, we report all results for ages 15 years and over, age-standardised using GBD standard populations (appendix 1 section 5). In the main text, we also present estimates across two broader age groups (15–49 years and ≥50 years). Results are showcased globally, by seven super-regions, and for 204 countries and territories (appendix 1 section 6) for the year 2023. Estimates produced for the year 1990 are provided in appendix 2 (section 1). Mean estimates for exposure and attributable burden metrics were calculated as the average across draws from their respective distributions (1000 draws for exposure; 250 for attributable burden), with 95% UIs defined by the 2·5th and 97·5th percentiles across these draws. Uncertainty around the final burden estimates reflects the propagation of uncertainty through each modelling step: exposure, RRs (250 draws), and PAFs (250 draws).^26^ Downsampling exposure draws to 250 for PAF calculation had no effect on propagated uncertainty (appendix 1 section 7).

To aid communication and policy discussion, and taking advantage of the comparative framework of GBD, we ranked IPV and SVAC alongside other GBD Level 3 risk factors using mean estimates of DALY counts. A complete list of the risks included in the GBD Level 3 category can be found within the GBD 2023 risk factor report,^26^ as well as appendix 1 (section 8). All analyses were completed in R (version 4.2.1) and Python (version 3.10.4).

### Role of the funding source

The funder of the study had no role in study design, data collection, data analysis, data interpretation, or the writing of the report.

## Results

### Prevalence

Globally, in 2023, there were an estimated 608 million (95% UI 518–724) females aged 15 years and older who had been exposed to IPV and 1·01 billion (0·764–1·48) males and females aged 15 years and older who had been exposed to sexual violence during childhood. In 2023, global estimates of the prevalence of IPV and sexual violence experienced during childhood were highest among individuals aged 15–49 years and declined after age 50 years (appendix 2 table S3).

**Figure 1:**
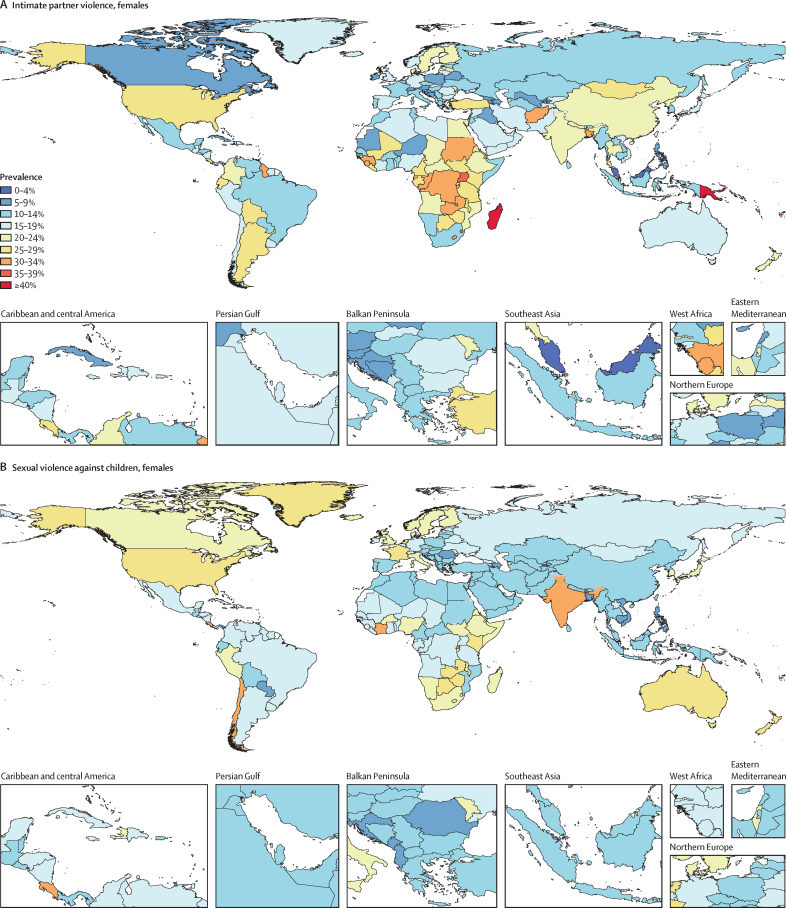

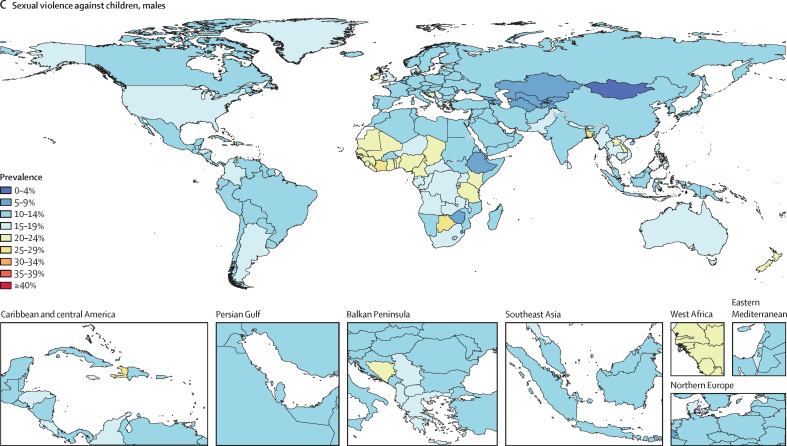
Age-standardised prevalence of intimate partner among females aged 15 years and older (A) and sexual violence against children among females (B) and males (C), in 2023 Exposure to sexual violence against children was estimated for females and males aged 15 years or older retrospectively reporting on sexual violence experienced during childhood (before age 18 years).

Countries with the highest age-standardised prevalence of IPV were primarily located within the sub-Saharan Africa and the southeast Asia, east Asia, and Oceania super-regions, while the overall highest regional prevalence was found in south Asia (figure 1A; appendix 2 table S4). For SVAC, the highest regional prevalence was observed in the south Asia and sub-Saharan Africa super-regions, followed by the high-income super-region (figure 1B–C; appendix 2 figure S1, table S4). Among females aged 15 years and older, the age-standardised prevalence of IPV in 2023 varied widely, ranging from 3·1% (95% UI 1·6–5·9) in Malaysia to 53·9% (29·3–78·3) in Kiribati (figure 1A). Prevalence was greater than 20·0% in 77 countries (appendix 2 table S4). For SVAC, the age-standardised prevalence among females aged 15 years and older ranged from 7·0% (5·1–9·5) in Montenegro to 42·0% (33·4–52·8) in Solomon Islands (figure 1B), with rates exceeding 20·0% in 54 countries (appendix 2 table S4). Among males aged 15 years and older, the age-standardised prevalence of SVAC ranged from 4·2% (1·6–9·1) in Mongolia to 28·6% (14·8–48·2) in Côte d'Ivoire (figure 1C), with rates exceeding 20·0% in 25 countries (appendix 2 table S4).

### Relative risks

Eight health outcomes were linked to IPV in GBD 2023. Beyond the three outcomes previously estimated in GBD studies (HIV/AIDS, major depressive disorder, and interpersonal violence homicide and injuries), we examined five more outcomes with sufficient data for burden-of-proof analyses and found evidence of associations between IPV exposure and all five: self-harm, maternal abortion and miscarriage, maternal haemorrhage, anxiety disorders, and drug use disorders (appendix 2 table S5).

Similarly, we examined 14 new health conditions in relation to SVAC and identified 12 associations with exposure to SVAC that were not included in previous iterations of the GBD: type 2 diabetes, HIV/AIDS, self-harm, abortion and miscarriage, drug use disorders, anxiety disorders, bipolar disorder, conduct disorder, bulimia nervosa, schizophrenia, asthma, and sexually transmitted infections excluding HIV. Alongside updated evidence for two conditions previously linked to SVAC in GBD studies—alcohol use disorder and major depressive disorder—this resulted in a total of 14 health outcomes associated with SVAC in GBD 2023. Estimated RRs used in the calculation of PAFs and additional estimated metrics for each risk–outcome combination are presented in appendix 2 (table S6).

### Disease burden attributable to IPV and SVAC

**Figure 2:**
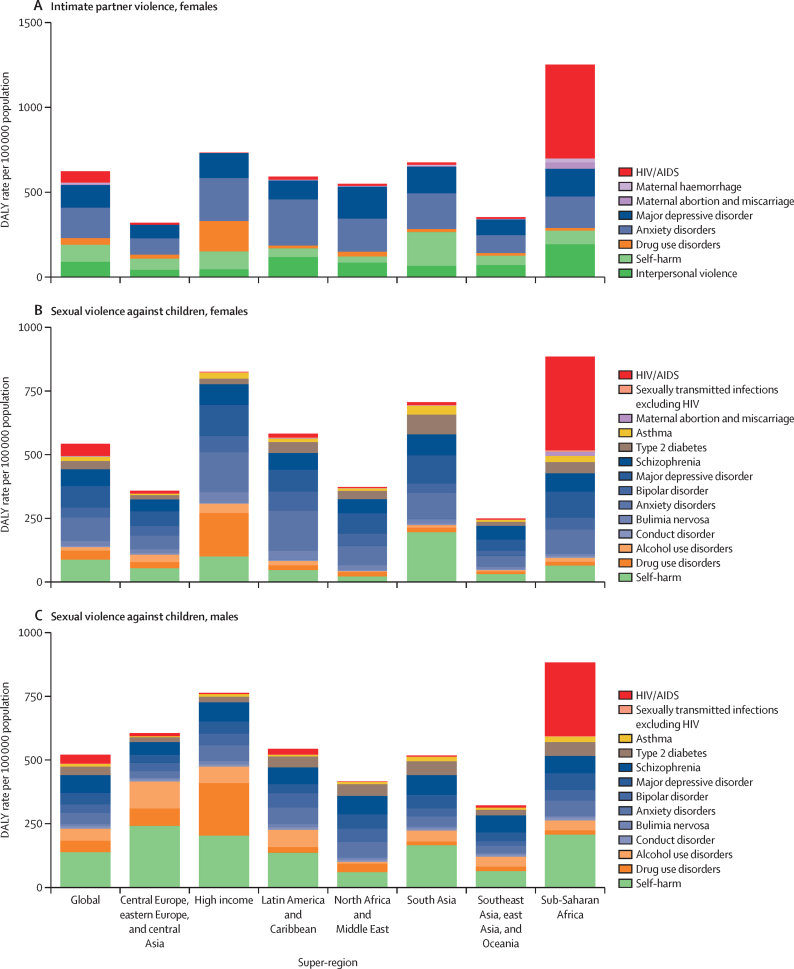
Age-standardised rates of cause-specific DALYs attributable to intimate partner violence among females (A) and to sexual violence against children among females (B) and males (C) aged 15 years and older, globally and by super-region, 2023 Bar heights represent all-cause DALY rates attributed to the respective risk factor and among the respective population globally and in each of the seven GBD super-regions. Colours indicate cause groupings, while shading within each colour category denotes specific Level 3 causes in the GBD cause hierarchy. DALY=disability-adjusted life-year. GBD=Global Burden of Diseases, Injuries, and Risk Factors Study.

**Figure 3:**
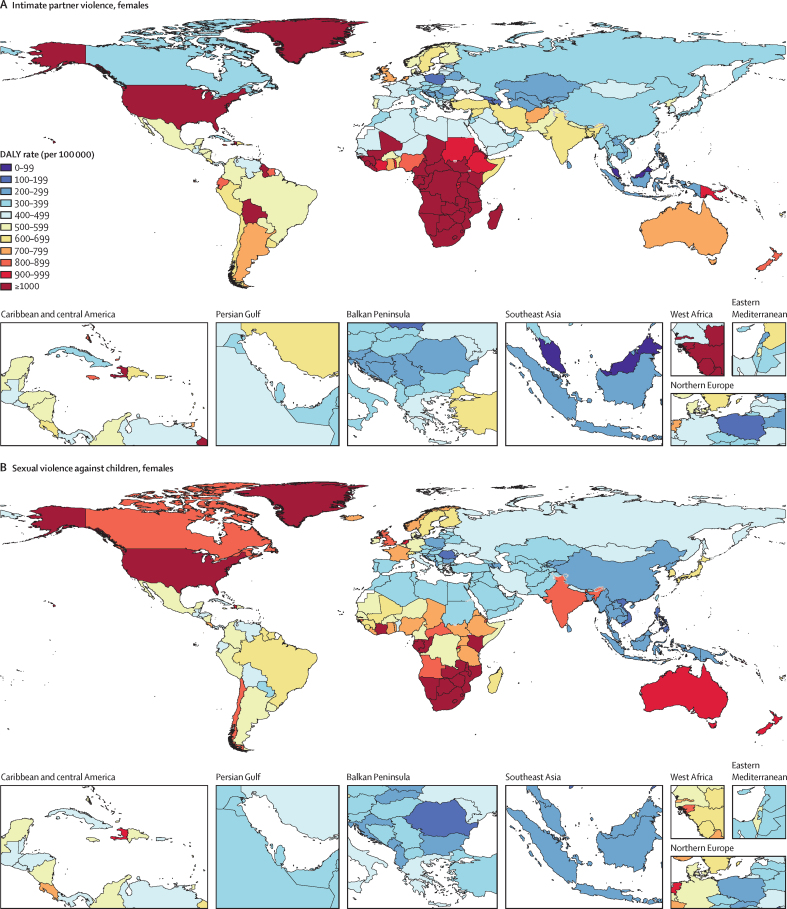

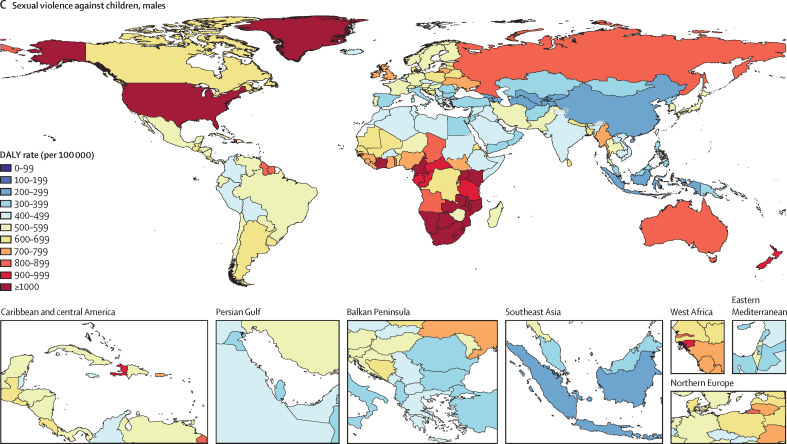
Age-standardised DALY rates attributable to intimate partner violence among females (A) and to sexual violence against children among females (B) and males (C) aged 15 years and older, 2023 DALY rates are presented per 100 000 people. DALY=disability-adjusted life-year.

In 2023, 145 thousand (95% UI 9·18–301) deaths and 18·5 million (8·74–30·0) DALYs were attributable to IPV among females aged 15 years and older. Additionally, 290 thousand (119–537) deaths and 32·2 million (16·4–52·5) DALYs were attributable to sexual violence experienced during childhood among individuals aged 15 years and older (appendix 2 tables S1–S2). Globally, the age-standardised rate of all-cause DALYs due to IPV among females aged 15 years and older was 624·4 DALYs (294·7–1015·6) per 100 000 females. At the super-region level the rate of DALYs due to IPV was highest in sub-Saharan Africa (1252·5 [540·8–2160·8] per 100 000) and lowest in central Europe, eastern Europe, and central Asia (321·3 [158·0–542·1] per 100 000; figure 2A; appendix 2 table S2). In 2023, across all countries, the highest age-standardised rates of DALYs due to IPV were found in Eswatini (6472·8 [2117·4–10 847·1] per 100 000), Lesotho (5778·7 [1624·2–9841·3] per 100 000), and Equatorial Guinea (3619·0 [892·8–6968·4] per 100 000), while the lowest rates were found in Malaysia (82·7 [45·0–144·5] per 100 000), Singapore (102·7 [36·5–231·1] per 100 000), and Georgia (135·8 [61·4–254·9] per 100 000; figure 3A; appendix 2 table S2).

Globally in 2023, age-standardised rates of cause-specific DALYs attributed to IPV were highest for anxiety disorders (181·1 [95% UI –41·8 to 484·4] per 100 000) followed by major depressive disorder (132·0 [56·9 to 230·7] per 100 000) and self-harm (100·8 [–71·0 to 286·6 per 100 000]). Cause-specific rates were lowest for maternal haemorrhage (6·4 [–3·9 to 17·6] per 100 000), abortion and miscarriage (7·2 [1·6 to 16·0] per 100 000), and drug use disorders (39·3 [–2·8 to 89·1] per 100 000; figure 2A). Anxiety disorders and major depressive disorder consistently had among the highest DALY rates attributable to IPV across all super-regions in 2023 (figure 2A; appendix 2 table S7). For the other causes, geographical variations in DALY rates were more pronounced. In sub-Saharan Africa, rates of IPV-attributed DALYs for HIV/AIDS (552·1 [89·7 to 1087·4] per 100 000), maternal haemorrhage (25·5 [–16·0 to 71·9] per 100 000), abortion and miscarriage (37·1 [8·2 to 83·2] per 100 000), and interpersonal violence homicide and injuries (192·5 [115·1 to 295·5] per 100 000) far exceeded global rates and those of any other super-region. Rates of IPV-attributable DALYs for self-harm in south Asia (197·5 [–146·6 to 563·7] per 100 000) and for drug use disorders in the high-income super-region (179·0 [–14·1 to 406·0] per 100 000) also surpassed global rates and those of other super-regions (figure 2A; appendix 2 table S7).

The global age-standardised rate of all-cause DALYs due to SVAC was 545·4 (95% UI 284·1–866·5) per 100 000 females aged 15 years and older and 520·7 (270·1–882·8) per 100 000 males in the same age range. The rate of DALYs attributable to SVAC was highest in sub-Saharan Africa, at 884·8 (260·4–1722·7) per 100 000 females and 882·1 (349·0–1577·1) per 100 000 males, and was lowest in southeast Asia, east Asia, and Oceania, at 246·8 (115·3–411·9) per 100 000 females and 323·3 (159·3–496·1) per 100 000 males (figure 2B–C; appendix 2 table S2, figure S2). In 2023, the countries with the highest age-standardised rates of SVAC-attributable DALYs were largely the same for both males and females. Botswana, Eswatini, Greenland, Lesotho, Malawi, South Africa, Zambia, and Equatorial Guinea consistently ranked among the ten countries with the highest rates for both sexes. For females, Namibia (1335·7 [96·0 to 3058·7] per 100 000) and Zimbabwe (1748·6 [–104·3 to 4066·6] per 100 000) also had notably high rates, while for males, Mozambique (1297·2 [165·1 to 3415·3] per 100 000) and the USA (1212·2 [422·0 to 2181·2] per 100 000) were also ranked within the ten highest rates (figure 3B–C; appendix 2 table S2).

Cause-specific rates of SVAC-attributed DALYs differed by sex. Among females in 2023, age-standardised rates of cause-specific DALYs due to SVAC were highest for anxiety disorders (92·4 [95% UI –41·1 to 292·7] per 100 000), self-harm (87·1 [28·2 to 151·6] per 100 000), and major depressive disorder (85·2 [9·1 to 192·3] per 100 000), whereas among males, rates were highest for self-harm (138·8 [38·0 to 284·4] per 100 000), schizophrenia (69·2 [–27·7 to 225·2] per 100 000), and alcohol use disorders (46·1 [19·3 to 83·6] per 100 000; figure 2B–C; appendix 2 table S8). Rates for substance use disorders, conduct disorder, and self-harm were higher for males than for females in all super-regions except south Asia, where rates of SVAC-attributable DALYs for drug use disorders and self-harm were higher among females than among males. Conversely, rates of SVAC-attributed DALYs for major depressive disorder, anxiety disorders, sexually transmitted infections (excluding HIV/AIDS), and bulimia nervosa were higher among females than among males in all super-regions. The rate of SVAC-attributed DALYs for drug use disorders in the high-income super-region (204·7 [–50·5 to 540·6] per 100 000 males; 170·9 [–62·4 to 435·1] per 100 000 females) were notably higher than in any other super-region. Likewise, the DALY rate due to HIV/AIDS was highest in sub-Saharan Africa (289·8 [–96·2 to 843·6] per 100 000 males; 368·7 [–135·0 to 1047·0] per 100 000 females) when compared with all other super-regions (figure 2B–C; appendix 2 table S8). Among females, south Asia had particularly high rates of SVAC-attributed DALYs due to type 2 diabetes (78·1 [0·1–196·4] per 100 000) and self-harm (194·7 [63·1–345·0] per 100 000) when compared with females in other super-regions. Among males, rates of SVAC-attributed DALYs associated with self-harm (240·8 [47·4–550·8] per 100 000) and alcohol use disorders (105·9 [33·6–221·8] per 100 000) were comparatively high in central Europe, eastern Europe, and central Asia (figure 2B–C; appendix 2 table S8).

**Figure 4:**
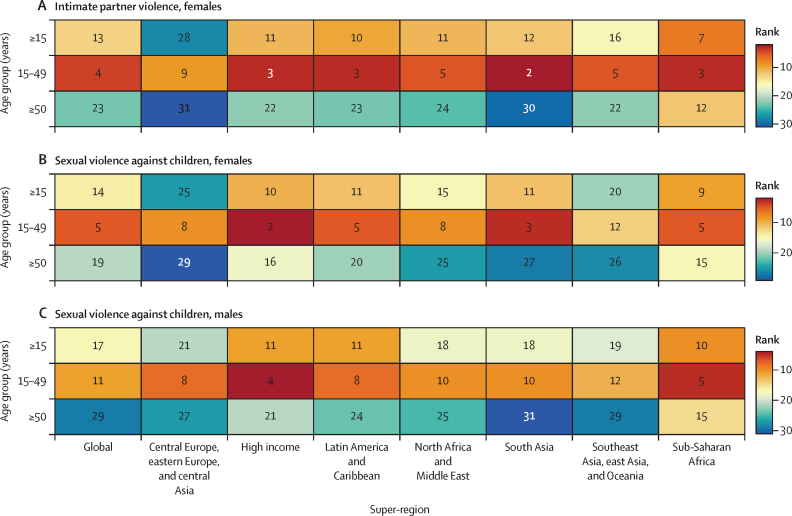
Rankings of DALY counts attributable to intimate partner violence among females (A) and to sexual violence against children among females (B) and males (C), compared with other Level 3 risk factors included in GBD 2023, globally and by super-region Rows display DALY count rankings for all individuals aged 15 years and older, as well as for specific age strata. Columns show rankings globally and across each of the seven GBD super-regions. Numbers within each cell indicate the ranking of the respective risk factor among GBD Level 3 risk factors (see full list in appendix 1 section 8) within each population group and geographical region in 2023. Cell colours represent the relative rankings, ranging from red (lower ranking, higher DALYs) to blue (higher ranking, lower DALYs). Detailed risk factor rankings for ages 15 years and older and 15–49 years are available in appendix 2 (figures S3–S6). DALY=disability-adjusted life-year. GBD=Global Burden of Diseases, Injuries, and Risk Factors Study.

When comparing mean estimates of risk-attributable DALY counts among other risks analysed in GBD 2023, IPV and SVAC emerged as the 13th and 14th leading risks, respectively, for global DALYs among females aged 15 years and older (figure 4A–B; appendix 2 figure S3). Notably, among females aged 15–49 years, IPV and SVAC ranked fourth and fifth (figure 4A–B; appendix 2 figure S4). In the south Asia and high-income super-regions, both IPV and SVAC ranked among the top three leading risk factors for females aged 15–49 years (figure 4A–B). Among males, SVAC was the 17th leading risk globally for those aged 15 years and older and reached the 11th position among those aged 15–49 years (figure 4C; appendix 2 figures S5–S6). For males aged 15–49 years, SVAC ranked within the top 10 risks contributing to DALYs in six out of seven super-regions, ranking among the top five risks in the high-income and sub-Saharan Africa super-regions (fourth and fifth leading risks, respectively; figure 4C).

In 2023, IPV accounted for 1·8% (95% UI 0·8–2·9) of all-cause DALYs among females aged 15 years and older globally. SVAC was responsible for 1·6% (0·8–2·4) of all-cause DALYs among females and 1·4% (0·7–2·4) among males aged 15 years and older. The proportion of global DALYs attributable to IPV was highest among females aged 25–29 years, at 4·3% (2·1–6·9). For females, the highest percentage of all-cause global DALYs due to SVAC was seen among those aged 15–19 years (3·1% [1·6–5·2]), whereas for males, it was highest among those aged 30–34 years (2·9% [1·4–4·7]; appendix 2 figure S7).

**Figure 5:**
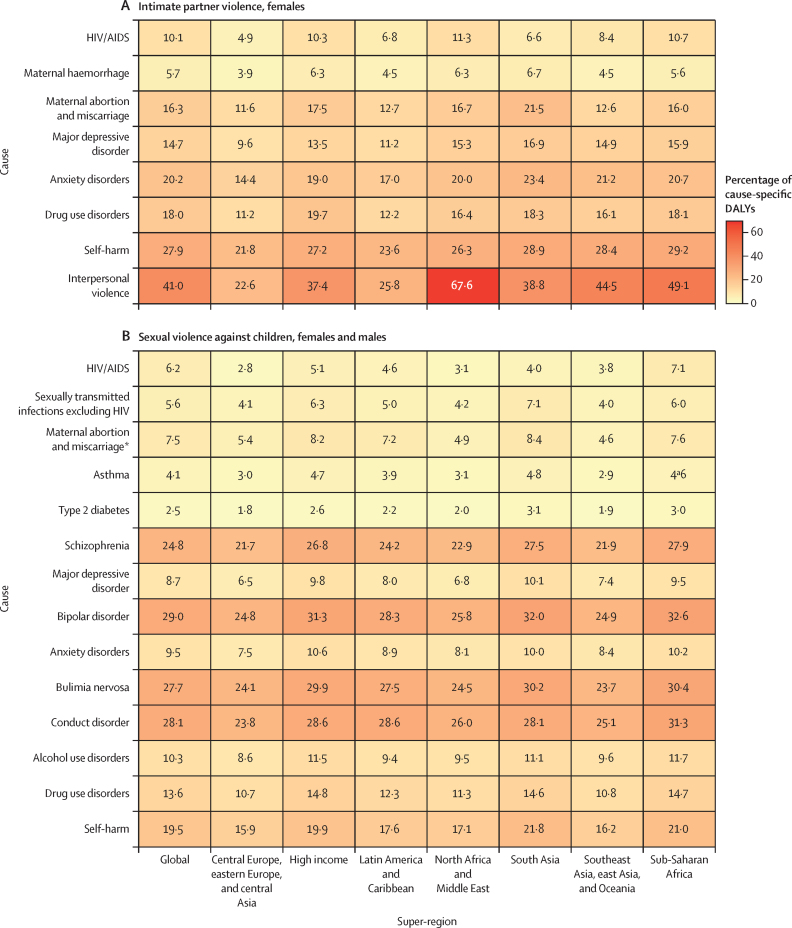
Percentage of cause-specific health burden attributable to intimate partner violence among females (A) and to sexual violence against children among females and males combined (B) aged 15 years and older, globally and by super-region, 2023 Percentages represent the proportion of cause-specific DALYs among individuals aged 15 years and older attributed to intimate partner violence or sexual violence against children globally or within a specific super-region. Numbers within each cell are estimated mean percentages, while cell colours represent the relative level of the mean percentage, ranging from yellow (lower percentage) to red (higher percentage). Causes are presented at Level 3 of the GBD cause hierarchy. DALY=disability-adjusted life-year. GBD=Global Burden of Diseases, Injuries, and Risk Factors Study. *Female-specific condition.

Among the eight health outcomes associated with IPV, the largest contributors to DALYs attributable to IPV exposure among females aged 15 years and older were anxiety disorders (5·43 million [95% UI –1·25 to 14·6] DALYs), major depressive disorder (3·96 million [1·71 to 6·92]), and self-harm (2·97 million [–2·08 to 8·40]; appendix 2 table S9). Among females aged 15 years and older in 2023, IPV accounted for 20·2% (–3·3 to 49·9), 14·7% (7·0 to 23·7), and 27·9% (–18·6 to 78·0) of the global DALYs due to these conditions, respectively (appendix 2 table S10). IPV exposure contributed to more than 10·0% of the cause-specific global DALYs for seven out of the eight outcomes it was associated with. The exception was maternal haemorrhage, for which IPV accounted for 5·7% (–3·1 to 15·0) of DALYs (figure 5A). The highest number of deaths attributable to exposure to IPV resulted from self-harm (60·6 thousand [–39·9 to 173] deaths), HIV/AIDS (40·7 thousand [6·40 to 80·6]), and interpersonal violence homicides (28·2 thousand [18·4 to 40·5]; appendix 2 table S11). IPV accounted for a particularly large share of the total health burden from interpersonal violence homicide and injuries among females, with 2·60 million (1·71–3·67) DALYs due to this cause attributable to IPV in 2023, representing 41·0% (28·5–53·3) of this outcome's DALYs and roughly 1 death per 100 000 females aged 15 years and older (appendix 2 tables S9–S13). At the super-regional level, IPV against females accounted for more than half of interpersonal violence DALYs in north Africa and the Middle East (67·6% [49·8–84·8]) and nearly half of those in sub-Saharan Africa (49·1% [32·1–65·4]; figure 5A; appendix 2 table S10).

Among the 14 health outcomes linked to SVAC, the leading causes contributing to DALYs attributable to SVAC were self-harm (6·71 million [95% UI 2·00 to 12·7] DALYs), schizophrenia (4·15 million [–1·92 to 13·1]), and anxiety disorders (4·13 million [–1·71 to 13·3]; appendix 2 table S14). SVAC accounted for 19·5% (5·6 to 37·5), 24·8% (–10·7 to 74·2), and 9·5% (–3·6 to 28·1) of the global DALYs due to these causes, respectively (figure 5B; appendix 2 table S15). In 2023, SVAC exposure was responsible for more than 20% of cause-specific DALYs for several mental health outcomes, including bipolar disorder (29·0% [–0·8 to 67·3]), conduct disorder (28·1% [–6·9 to 77·5]), schizophrenia (24·8% [–10·7 to 74·2]), and bulimia nervosa (27·7% [–12·5 to 78·4]; figure 5B). Among substance use disorders, more than 10% of cause-specific global DALYs were also attributable to SVAC for alcohol use disorders (10·3% [4·6 to 17·9]) and drug use disorders (13·6% [–3·6 to 35·6]; figure 5B). Six of the 14 health outcomes associated with SVAC are mental health conditions, which do not carry fatal burden in GBD. Thus, while mental health conditions were leading causes of DALYs due to SVAC, the primary causes of death attributable to SVAC were self-harm (141 thousand [40·6 to 273] deaths), HIV/AIDS (48·6 thousand [–17·1 to 137]), and type 2 diabetes (42·2 thousand [0·0333 to 109]; appendix 2 table S16). For both HIV/AIDS and type 2 diabetes, exposure to SVAC accounted for a relatively small proportion of total DALYs (6·2% [–2·1 to 17·1] for HIV/AIDS and 2·5% [0·0 to 6·0] for type 2 diabetes; figure 5B; appendix 2 table S15). The cause-specific proportion of DALYs attributable to SVAC did not vary substantially by sex. These results, as well as the rates and proportions of deaths attributable to SVAC, are provided in appendix 2 (tables S17–S18, figures S8–S9).

## Discussion

Our study offers an extended and updated examination of the disease burden attributable to IPV and SVAC across 204 countries and territories. Our results capture a broad range of negative health outcomes associated with both forms of violence—from maternal disorders and sexually transmitted diseases to chronic diseases and physical injuries—and notably expand upon the outcomes assessed in previous iterations of GBD. Despite widespread recognition of the profound individual and societal threats posed by IPV and SVAC, the statistics remain alarmingly high. For 2023, we estimated that 608 million (95% UI 518–724) girls and women had experienced physical or sexual violence from their intimate partners, and 1·01 billion (0·764–1·48) individuals aged 15 years and older had experienced sexual violence during their childhood. The health burden of these acts manifests as a substantial loss of health for those exposed, both due to premature mortality and long-term disability. These findings paint a stark picture of the enduring impacts of IPV and SVAC and affirm their roles as substantial drivers of global health burden.

The GBD comparative risk assessment framework is instrumental in contextualising IPV and SVAC among a wide range of health threats.^26^ The high prevalence rates of these risks across the world, combined with their association with numerous adverse health outcomes—including some major causes of disease burden such as depressive and anxiety disorders—underlies the magnitude of the contributions of IPV and SVAC to early mortality and disability throughout the life course. These exposures are particularly consequential for young and middle-aged individuals, and the loss of health and productivity at these key stages has far-reaching economic and social implications, potentially affecting national development. Notably, in 2023, IPV and SVAC emerged as the fourth and fifth leading risk factors for DALYs among females aged 15–49 years, respectively, while SVAC ranked 11th among males in the same age group. Given their social and health toll, violence against women and children must also be featured prominently in global calls for healthier lives, as achieving a truly healthy society requires striving not only for physical wellbeing but also for a violence-free world where every individual can thrive in safety and dignity.

Differences in exposure rates and the overall disease burden profile across locations resulted in notable geographical disparities in the health burden of IPV and SVAC. Sub-Saharan Africa had the highest rates of DALYs attributable to both SVAC and IPV, a pattern largely driven by high prevalence rates and the vastly debated substantial burden of HIV in this super-region.^35^ Exposure to violence against women and children in low-income and middle-income countries have been linked to socioeconomic inequalities, weak governance, entrenched gender norms, forced migration, structural violence, and inadequate access to health care and other support systems.^36^, ^37^, ^38^ However, DALY rates for IPV and SVAC were also elevated across the high-income super-region compared with other super-regions, reflecting the large burden of substance use disorders and non-communicable diseases in these settings.^29^ Notably, among males aged 15–49 years in the high-income super-region, the burden of SVAC ranked fourth. This finding underscores the need for nations in development to monitor and address these risks and associated outcomes early, as reducing other major health threats could increase the relative importance of IPV and SVAC in the overall disease burden.

While our results show that the detrimental effects of violence against women and children are ubiquitous and span societies across all levels of economic development, it is important to acknowledge that geographical variations in our estimates might stem not solely from actual differences in violence exposure or burden profiles but also potentially from discrepancies in reporting practices and disclosure rates. Cultural norms, gender dynamics, and the availability of reliable data collection all influence these factors.^39^, ^40^ Furthermore, the varying capacities of local health systems to diagnose and record health outcomes linked to IPV and SVAC are likely to have contributed to the regional disparities observed in our attributable burden estimates. For example, a comprehensive assessment of the state of the diabetes cascade of care globally showed that underdiagnosis remains a major challenge, particularly in low-income and middle-income countries,^41^ with similar variations in health system capacity also evident for other conditions, such as mental health disorders.^42^ These gaps limit our ability to accurately ascertain the true prevalence of these conditions and, consequently, impair the assessment of the contribution of IPV and SVAC to their overall health burden, highlighting the need to strengthen health systems to improve data quality and inform effective context-specific interventions.

Recognising the magnitude of the health loss associated with IPV and SVAC, it is imperative to increase and amplify violence-prevention measures and targeted response strategies, including the integration of effective, multipronged support for survivors into women's health programmes as well as broader public health initiatives. Such efforts will require robust funding and sustained political commitment at the international, national, and community levels. Currently, less than 1% of aid spending targets gender-based violence—an indication that there is a substantial gap in prioritising this important issue within global health agendas.^43^ Notably, given the widespread health burden associated with these risks, there is an urgent need for increased prioritisation and funding—not only through international aid and development support, but also as a core component of national social and health agendas. Recent international efforts have established frameworks and resources to guide action plans for violence prevention and responses.^15^, ^16^ Examples of comprehensive strategies include promoting child-protective and gender-equality norms, strengthening legal frameworks, integrating responses to IPV and SVAC within mental health and social support services, and fostering community mobilisation initiatives that create safe spaces for survivors.^14^, ^44^, ^45^ Effective interventions must also recognise the cyclical nature of violence—whereby those who have experienced violence might go on to perpetuate violence across generations and throughout communities—and seek to intervene in this cycle by promoting safer and healthier family environments and supports.^13^, ^46^ Moreover, intervention strategies should be inclusive and consider gender dynamics, ensuring that the unique needs of boys and men, as well as girls and women, are addressed through gender-responsive approaches.

By prioritising the prevention of IPV and SVAC, countries will be better positioned to tackle multiple health challenges simultaneously, thereby alleviating pressure on their health-care systems. Our results suggest that eliminating exposure to these risks could avert millions of DALYs annually, primarily through reductions in the burden of mental health disorders, self-harm, interpersonal violence, substance use, and infectious diseases such as HIV. Notably, the substantial contributions of IPV and SVAC to the burden of mental disorders—a leading cause of health loss for which effective preventive measures remain limited^47^—underscores the need to incorporate these risks into strategic plans targeting this cluster of conditions. Such integration is likely to yield further global health gains, particularly among women, who disproportionately experience mental health burden over their life course.^48^ Furthermore, IPV emerged as a crucial driver of the burden attributable to homicide and injuries among women, accounting for over 40% of the associated DALYs worldwide and nearly 70% in North Africa and the Middle East. Alarmingly, over half of these DALYs globally are linked to premature mortality from partner-perpetrated killings, with nearly 30 000 women estimated to have been killed by their partners in 2023, highlighting the urgent need for enhanced protective measures for at-risk individuals. At the same time, it is notable that IPV-attributable deaths from self-harm and HIV exceeded those from direct homicide in 2023. The substantial proportion of self-harm-related deaths associated with both IPV and SVAC is especially concerning, as these incidents disproportionately affect young people, whose loss carries grave implications for national development and societal resilience, pointing to the importance of prioritising self-harm prevention within violence response strategies to safeguard not only individual wellbeing, but also the future strength of communities and nations.

Importantly, while the associations of IPV and SVAC with mental disorders and self-harm have been relatively well documented, our findings also underscore the less commonly discussed relationship between SVAC and non-communicable diseases such as diabetes and asthma. Emerging evidence suggests that exposure to violence during childhood might contribute to the development of these conditions through mechanisms including chronic stress and inflammation, pathways known to influence metabolic and immune function.^49^ Although the current evidence base for these associations remains limited and further investigation is warranted to clarify causal links and underlying biological mechanisms, the effects of these associations on the overall burden attributable to SVAC is notable. For example, given the magnitude of its global burden,^50^ type 2 diabetes stood out as one of the major contributors to deaths attributable to SVAC, with over 42 000 SVAC-associated deaths in 2023. It is particularly important to invest in an evidence base that could be used to explore whether similar associations exist for IPV.

Considering the wide array of health conditions associated with IPV and SVAC, survivors will continue to require both immediate and long-term care from health services worldwide. While the health sector is positioned at the forefront of efforts to identify, protect, rehabilitate, and support survivors, comprehensive and specialised services to address violence are often lacking. Considerable barriers, such as insufficient access to affordable care, pervasive stigma within health-care settings, and systemic inequities, further challenge the ability of health systems to effectively meet the critical needs of survivors.^18^, ^51^, ^52^ Therefore, it is essential to design and implement targeted interventions that address variations in exposure while ensuring universal access for all survivors. The cause-specific evidence from this analysis could inform efforts from within the health-care system—eg, by assessing for violence exposure when treating individuals with health conditions shown to be associated with such exposures to improve both quality of care and connection to further resources.^53^ Indeed, emerging evidence suggests that early referrals to trauma-informed care, shared decision-making processes, and comprehensive support can greatly improve treatment quality and recovery trajectories for survivors.^54^ Support and interventions have traditionally been framed within the legal and criminal justice systems; however, our results firmly position IPV and SVAC as important health risks. Thus, fostering intersectoral collaboration among health-care providers, law enforcement, social services, and community organisations will be crucial for developing coordinated, sustainable interventions that help survivors to heal and find safety.^51^

Our study provides an extended overview of the disease burden associated with IPV and SVAC; however, several limitations should be noted. First, the prevalence data for IPV and SVAC in our models were self-reported and are therefore prone to under-reporting, given the sensitive nature of these topics and the associated stigma. Although we applied correction factors to account for SVAC under-reporting by survey method,^2^ a similar approach could not be used for IPV due to data constraints. Most data available for SVAC surveys adults who recall experiences during childhood, which could also introduce recall bias that was uncontrolled for in this study. Additionally, our analysis leveraged data from 1990–2023 and, although we undertook several data adjustment steps to account for differences in survey instruments, we were unable to control for how disclosure of exposure to violence has potentially changed over time. Moreover, while the number of high-quality, nationally representative prevalence surveys for IPV and SVAC has increased substantially in recent decades, substantial gaps remain across geographical regions and time periods. In areas with sparse data, such as the North Africa and Middle East super-region, our estimates rely heavily on the predictive validity of the modelling techniques employed, which might affect accuracy. The wide uncertainty intervals for some of our exposure estimates highlight the considerable statistical uncertainty that persists, particularly in data-sparse settings, and underscore the need for cautious interpretation, especially when informing policy decisions. Notably, several countries with sparse data correspond to those with the lowest observed exposure and DALY rates, and there is potential for these estimates to shift as new data emerge. Although persistent data gaps have posed substantial challenges to maintaining effective surveillance for decades, recent cuts to foreign assistance risk exacerbating existing limitations and further undermine the capacity of many countries to monitor and address violence against women and children. Sustained investment in research and surveillance remains crucial to closing these gaps and preventing setbacks in progress. Future data collection efforts should adhere to best practices in study design to ensure confidentiality, and interviewers should be trained to create a safe and supportive environment to enhance disclosure.^55^, ^56^

A second limitation is that our analysis focused exclusively on the health effects of IPV, defined as acts of physical or sexual violence perpetrated by an intimate partner, and SVAC. Notably, due to limitations in the available evidence to date,^23^ our operational definition of IPV, similar to those adopted by the WHO and UN SDG monitoring frameworks,^57^ does not encompass acts within an intimate relationship that induce fear or emotional distress. Additionally, our study does not address other forms of violence against children and women, such as physical or psychological abuse against children, child neglect, female genital mutilation, or violence perpetrated by individuals other than intimate partners. Analyses of the health effects of other forms of gender-based violence and violence against children, based on the same systematic review described in our Methods, have been published elsewhere.^33^, ^58^ It is essential for future work to consider the overlapping and unique health effects of additional forms of IPV, as well as violence beyond IPV and SVAC, as the most effective policies and intervention strategies might differ for these forms of violence.

Third, although the currently available evidence supported the inclusion of 12 additional health outcomes linked to SVAC and five linked to IPV in GBD 2023, these outcomes might still not fully capture the total health loss associated with these risks. The number of outcomes associated with each risk reflects the totality of available evidence, as well as study design and follow-up time, all of which are greater for SVAC than for IPV. Cohorts assessing childhood exposures such as SVAC often include longer and repeated follow-up, enabling the detection of more long-term health effects, whereas IPV cohorts often have shorter follow-up or limited data, and a smaller number of health outcomes assessed. In future iterations of GBD, we plan to continue to evaluate the evidence supporting associations between additional health outcomes and IPV and SVAC. Through regular updates of our systematic reviews, we will also evaluate evidence supporting the inclusion of additional forms of violence and their associated outcomes in GBD, contingent on factors such as the availability of evidence and the robustness of risk–outcome associations. To advance this work, high-quality prospective cohort studies are needed to clarify links between exposure to violence and long-term health outcomes, as well as to reduce uncertainty in future burden estimates. As detailed elsewhere,^4^ the strength of evidence supporting some of the associations presented here between IPV and SVAC and specific health outcomes is weak; thus, additional rigorous studies are required, and current findings should be interpreted with caution. In addition, health system databases offer a promising avenue to quantify immediate health effects. Future research should also aim to establish consensus guidelines for addressing confounding and mediation, to strengthen causal inference. Notably, although our burden-of-proof meta-analysis synthesises the available evidence—accounting for confounding to the extent possible by coding for and adjusting for bias covariates representing known variation in input study characteristics—we acknowledge that there is no consensus on the level of evidence necessary to establish causality, especially relative to the observational studies which form the bulk of the available literature in this area.

Lastly, our study conceptualises IPV and SVAC as independent, not mutually exclusive risks, and does not estimate DALYs or deaths for individuals who have been exposed to both forms of violence. Additionally, the GBD framework does not currently account for potential correlations or clustering of these risks with other health threats. Furthermore, by treating IPV and SVAC as binary risks and using prevalence to measure exposure, our estimates do not account for the complexities related to the timing, frequency, and severity of exposure to violence, each of which could influence risk of future violence and negative health outcomes. For example, the experience of repeated acts of IPV might indicate greater risk for fatality.^59^ Thus, future research should leverage longitudinal and linked datasets to more accurately capture the co-occurrence of violence and provide a nuanced, cumulative analysis of its health effects over the life course.

Substantial progress has been made in understanding the global health burden of IPV and SVAC, and these pervasive forms of violence remain substantial contributors to DALYs worldwide. Our findings highlight the far-reaching implications of these forms of violence, not only for individual health outcomes (such as mental disorders, chronic diseases, and premature mortality), but also for entire populations, spanning socioeconomic and geographical divides. The magnitude of health loss associated with these risks, even when compared with other widely recognised health threats, indicates that these risks require robust recognition, and the socially created burdens of violence against women and children must be treated as public health priorities. Concerted efforts are required across multiple sectors to implement prevention strategies, enhance services for survivors, and eliminate structural barriers to care. Investing in sustainable, gender-sensitive violence prevention and support strategies holds the potential to avert millions of DALYs annually while promoting equity and improving health outcomes globally. The findings presented in this study serve as an urgent call to action to combat IPV and SVAC as avoidable risks for global disability and premature mortality.

### GBD 2023 Intimate Partner Violence and Sexual Violence against Children Collaborators

### Affiliations

### Contributors

### Data sharing

For detailed information on data sources and estimates in this Article, please visit the GHDx at https://ghdx.healthdata.org/record/ihme-data/gbd-2023-ipv-svac-1990-2023.

## Declaration of interests

S Afzal reports support for the present manuscript from the Institute of Public Health Lahore; payment or honoraria for lectures, presentations, speakers bureaus, manuscript writing or educational seminars provided by the Dean Institute of Public Health Lahore; support for attending meetings and travel provided by the Dean Institute of Public Health, Lahore Pakistan; participation on a data and safety monitoring board or advisory board as member of the Pakistan National Bioethics Committee, member of the Institutional Review Board of Fatima Jinnah Medical University, member of the Ethical Review Board and Data Monitoring Board Institute of Public Health Lahore Pakistan, and in-charge Clinical Research Organization King Edward Medical University; leadership or fiduciary roles in other board, society, committee or advocacy groups (paid or unpaid) as member of the Pakistan Higher Education Commission Research Committee, member of the Pakistan Medical and Dental Commission Research and Journals Committee, member of the Pakistan Society of Internal Medicine, member of the Pakistan Association of Medical Editors, member of the Medical Microbiology and Infectious Diseases Society, Fellow of Leads International, Fellow of Faculty of Public Health UK, and as a Fellow of the College of Physicians and Surgeons Pakistan; receipt of computer software and equipment from Bergen University Norway for research writing; other financial or non-financial support from the Dean Public Health and Preventive Medicine King Edward Medical University; outside the submitted work. J S Chandan reports support for the present manuscript from the National Institute for Health and Care Research; grants or contracts from the National Institute for Health and Care Research, Youth Endowment Fund, College of Policing, University of Birmingham, Birmingham City Council; consulting fees from Dexter AI (Chief Medical Officer); support for attending meetings and/or travel from the University of Miami and the University of Washington; outside the submitted work. K Deuba reports grants or contracts from the Research Council of Norway (project no 335495); outside the submitted work. D Dias da Silva reports grants or contracts from Fundação para a Ciência e a Tecnologia, project 2024.06933 RESTART (payment made to institution) and E2S|P.Porto—Escola Superior de Saúde do Politécnico do Porto (tenure position as an Adjunct Professor), LAQV-Requimte PT national funds (FCT/MECI, Fundação para a Ciência e Tecnologia and Ministério da Educação, Ciência e Inovação) through the project UID/50006—Laboratório Associado para a Química Verde—Tecnologias e Processos Limpos; payment or honoraria for lectures, presentations, speakers bureaus, manuscript writing or educational events from Faculdade de Farmácia da Universidade do Porto (Portugal) payment made to institution for lectures, Faculdade de Medicina da Universidade do Porto (Portugal) payment made to institution for lectures; support for attending meetings and/or travel from E2S|P.Porto—Escola Superior de Saúde do Politécnico do Porto, Portugal, and travel and meetings expenses from Research Unit LAQV-REQUIMTE, Portugal; outside the submitted work. A Faro reports support for the present manuscript from National Council for Scientific and Technological Development (CNPq), Brazil, CNPq Researcher (PQ B). R C Franklin reports support for attending meetings and/or travel from Australasian College of Tropical Medicine Annual Conference 2022-2025; leadership or fiduciary roles in other board, society, committee or advocacy groups (paid or unpaid) as President of Australasian College of Tropical Medicine, President of Kidsafe Australia, Board Member of the Royal Life Saving Society Australia, and Board Member of Auschem Trainingas; outside the submitted work. D Fry reports support for attending meetings and/or travel from the Institute for Health Metrics and Evaluation for attending an advisory group meeting related to this work; leadership or fiduciary roles in other board, society, committee or advocacy groups (paid or unpaid) on the Childlight's Index Technical Sub-Committee which is also around the topic of child sexual abuse data. F M Knaul reports grants or contracts with Merck KGaA/EMD Serono (research grant paid to the University of Miami), Tides Foundation via the Oak Foundation (two research grants paid to the University of Miami), Fondation Botnar, and the Finker-Frenkel Family Foundation. FMK also reports gifts to the University of Miami from the Wellcome Trust, Mena Catering, the Gloria Estefan Foundation, and the Jose Milton Foundation to support the Lancet Commission on Gender-based Violence and Maltreatment of Young People; consulting fees from Tecnológico de Monterrey; provided strategic guidance on research priorities and lectures for the Institute for Obesity Research at the Tecnólogico de Monterrey (university); payment or honoraria for lectures, presentations, speakers bureaus, manuscript writing or educational events from Cannon Medical Systems—FMK received honoraria for participation in two webinars related reducing disparities in women's health; leadership or fiduciary roles as Founding President of Tómatelo a Pecho, a Mexican non-profit organisation that promotes research, advocacy, awareness, and early detection of breast cancer and has expanded its mission to promote women's health more broadly; Senior Economist (unpaid) with the Mexican Health Foundation; Member of the Board of Directors (unpaid) for Esperanza United; and Member of the Board of Directors for Kristi House Children's Advocacy Center; outside the submitted work. K Krishan acknowledges non-financial support from the University Grants Commission (UGC) Centre of Advanced Study, CAS II, awarded to the Department of Anthropology, and RUSA 2.O grant awarded to Panjab University, Chandigarh, India, outside the submitted work. A-M L Laslett reports support for the present manuscript from the National Health and Medical Research Council of Australia (GNT 2016706; fellowship/salary); support for attending meetings and/or travel from the University of Agder, Norway for the Visiting Travel Award—local airfares and accommodation provided for visit (no conflict of interest); leadership or fiduciary roles in other board, society, committee or advocacy groups (paid or unpaid) as a member of the International Society for Addiction Journal Editors and as Secretary of the Kettil Bruun Society for Social and Epidemiological Research on Alcohol (La Trobe University Academic Board); outside the submitted work. J Liu reports support for the present manuscript from the National Natural Science Foundation (72474005) and Beijing Youth Scholar Program (081); grants or contracts from the National Natural Science Foundation (72474005) and Beijing Youth Scholar Program (081); outside the submitted work. K S-K Ma reports grants or contracts from a research grant from the International Team for Implantology; outside the submitted work. L Monasta reports support for the present manuscript from the Italian Ministry of Health to the Institute of Maternal and Child Health—IRCCS Burlo Garofol. LM received funds from the Italian Ministry of Health payments made to the Institute for Maternal and Child Health—IRCCS Burlo Garofolo under project RC 34/2017. R S Moreira reports grants or contracts from CNPq Research Productivity Scholarship (National Council for Scientific and Technological Development); scholarship registration number 308986/2025-3, outside the submitted work. R F Palma-Alvarez reports payment or honoraria for lectures, presentations, speakers bureaus, manuscript writing, or educational events from Angelini, Casen Recordati, Lundbeck, Neuraxpharm, Rubió, Servier, Takeda; support for attending meetings and/or travel from Angelini, Italfarmaco, Advanz Pharma, Takeda, Lundbeck, Camurus; outside the submitted work. L Ronfani reports support for the present manuscript from the Italian Ministry of Health (Ricerca Corrente 34/2017), payments made to the Institute for Maternal and Child Health IRCCS Burlo Garofolo. V Sharma acknowledges support from the Directorate of Forensic Science Services (DFSS) Ministry of Home Affairs (MHA) research project (DFSS28(1)2019/EMR/6) at Institute of Forensic Science & Criminology, Panjab University, Chandigarh, India, and RUSA grant to Panjab University by Ministry of Education, Govt. of India, outside the submitted work. D J Stein reports support from consultancy honoraria from Discovery Vitality, Kanna, L'Oreal, Lundbeck, Orion, Servier, Seaport Therapeutics, Takeda, and Wellcome; outside the submitted work. R Tabares-Seisdedos reports grants or contracts from the Valencian Regional Government's Ministry of Education (PROMETEO/CIPROM/2022/58) and the Spanish Ministry of Science, Innovation and Universities (PID2021-129099OB-I00); these funders were not involved in the design of the manuscript or decision to submit the manuscript for publication, nor will they be involved in any aspect of the study's conduct, outside the submitted work. S J Tromans reports grants or contracts from the 2023/4 Adult Psychiatric Morbidity Survey team, collecting epidemiological data on community-based adults living in England (a contracted study from NHS Digital, via the Department of Health and Social Care); contributed to multiple chapters of the 2023/4 Adult Psychiatric Morbidity Survey report (payments made to University of Leicester); grants or contracts as lead on a study funded by the National Institute for Health and Care Research Clinical Research Network, on optimising survey design for people with learning disability and autistic people (payments made to University of Leicester); served as the lead on a study from the National Institute for Health and Care Research related to evaluating a national training programme for health and social care professionals relating to learning disability and autism (payments made to University of Leicester); was co-applicant on a study funded by the National Institute for Health and Care Research related to identification, recording, and reasonable adjustments for people with a learning disability and autistic people in NHS electronic clinical record systems (payments made to University of Leicester); was co-applicant on a study funded by the National Institute for Health and Care Research related to medication support interventions and strategies for people with learning disabilities (payments made to University of Leicester); served as lead applicant on a study funded by the Baily Thomas Charitable Fund investigating barriers, enablers and interventions to facilitate de-prescribing for people with intellectual disability (payments made to University of Leicester); has received support for attending meetings and/or travel from the Royal College of Psychiatrists for accommodation and travel to conference events due to academic secretary role in the faculty of the Psychiatry of Intellectual Disability, including conference fees waived for Royal College of Psychiatrists events; reports leadership or fiduciary roles in other board, society, committee or advocacy groups (unpaid) as Academic Secretary and Executive Committee Member for the Neurodevelopmental Psychiatry Special Interest Group and Psychiatry of Intellectual Disability Faculty at the Royal College of Psychiatrists; is Associate Editor for the *Journal of Mental Health Research in Intellectual Disabilities;* is Editorial Board Member for *Progress in Neurology and Psychiatry, Advances in Mental Health and Intellectual Disability, Advances in Autism, BMC Psychiatry*, and *BJPsych Open*; and received royalties as Editor of *Psychiatry of Intellectual Disability Across Cultures* (Oxford University Press); all outside the submitted work. A C Tsai reports support for the present manuscript from the US National Institutes of Health (K24DA061696); leadership or fiduciary roles in other board, society, committee or advocacy groups (paid) with Elsevier, receipt of financial honorarium for work as Co-Editor in Chief of the Elsevier-owned journal *SSM—Mental Health*, and from BMJ Publishing Group, receipt of financial honorarium for work as Clinical Editorial Advisor for the BMJ Publishing-owned journal *The BMJ*, outside the submitted work. A Vieira reports leadership or fiduciary roles in other board, society, committee or advocacy groups (unpaid) with the American Academy of Sleep Medicine as the ambassador 2025; outside the submitted work. S Zadey reports payment or honoraria for lectures, presentations, speakers bureaus, manuscript writing or educational events from Hindu; leadership or fiduciary roles in other board, society, committee or advocacy groups (paid or unpaid) as Co-founder and Board Member of The Association for Socially Applicable Research, an Advisor with Nivarana, a Permanent Council Member for G4 Alliance and Chair of the Asia Working Group, a Drafting Committee Member for the Maharashtra State Mental Health Policy, a Fellow for a *Lancet* Commission, a Fellow for the Blood DESERT Coalition, and an Adjunct Appointment at Dr D Y Patil Vidyapeeth (deemed to be university); outside the submitted work. G Zamagni reports support for the present manuscript from the Italian Ministry of Health (Ricerca Corrente 34/2017), payments made to the Institute for Maternal and Child Health IRCCS Burlo Garofolo.
