# Supplementary material for: Disease burden attributable to intimate partner violence against females and sexual violence against children in 204 countries and territories, 1990–2023: a systematic analysis for the Global Burden of Disease Study 2023
Source: Lancet. 2026 Jan 3;407(10523):31–52. doi: 10.1016/S0140-6736(25)02503-6 (PMC12775558; doi:10.1016/S0140-6736(25)02503-6)
Supplement: Supplementary appendix 1 [file mmc1.pdf]

THE LANCET

Supplementary appendix 1

This appendix formed part of the original submission and has been peer reviewed. We post it as supplied by the authors.

Supplement to: GBD 2023 Intimate Partner Violence and Sexual Violence against Children Collaborators. Disease burden attributable to intimate partner violence against females and sexual violence against children in 204 countries and territories, 1990–2023: a systematic analysis for the Global Burden of Disease Study 2023. *Lancet* 2025; published online Dec 9. https://doi.org/10.1016/S0140-6736(25)02503-6.

Supplementary Methods

Disease burden attributable to intimate partner violence against females and sexual violence against children in 204 countries and territories, 1990–2023: a systematic analysis from the Global Burden of Disease Study 2023

GBD 2023 Intimate Partner Violence and Sexual Violence against Children collaborators

Contents

Section 1: GATHER Checklist 4

Section 2: Exposure modelling 6

Section 2.1: Exposure data 6

Section 2.1.1: Data identification 6

Section 2.1.2: Inclusion criteria 6

Section 2.1.3 Case definitions 7

Section 2.1.4: Exposure sources lists 8

Section 2.1.5: Exposure data coverage 107

Section 2.2: Intimate partner violence exposure estimation 110

Model flowchart 110

Section 2.2.1: Adjustment for non-standard case definitions 110

Section 2.2.2: Age-splitting 120

Section 2.2.3: Adjustment for estimates among ever-partnered women only 120

Section 2.2.4: Prevalence modelling 120

Section 2.3: Sexual violence against children exposure estimation 122

Model flowchart 122

Section 2.3.1: Adjustment for non-standard case definitions 122

Section 2.3.2: Adjustment for non-disclosure by mode of survey delivery 123

Section 2.3.3: Age-splitting 124

Section 2.3.4: Cohort extrapolation 125

Section 2.3.5: Prevalence modelling 125

Section 3: Risk outcome pair identification and modelling 127

Section 3.1: Systematic review of scientific literature 127

Section 3.1.1: Search parameters 127

Section 3.1.2: Screening process 127

Section 3.1.3: Inclusion criteria 128

Section 3.1.4: Exclusion criteria 128

Section 3.1.5: PRISMA diagram 129

Section 3.2: Relative risk estimation 130
Section 3.2.1: Burden of Proof Risk Factor methodology 130
Section 4: Population attributable fraction calculations 132
Section 4.1: Cumulative risk approach for HIV/AIDS 132
Section 5: Demographics and age-standardisation 133
Section 6: Locations estimated 134
Section 7: GBD estimation draws 140
Section 8: Risk factors hierarchy 141
References 143

Section 1: GATHER Checklist

Table S1: GATHER checklist

Item #	Checklist item	Reported on page #
Objectives and funding		
1	Define the indicator(s), populations (including age, sex, and geographic entities), and time period(s) for which estimates were made.	Main text - Methods overview
2	List the funding sources for the work.	Main text - Summary
Data Inputs		
For all data inputs from multiple sources that are synthesized as part of the study:		
3	Describe how the data were identified and how the data were accessed.	Main text - Methods; Appendix 1 - Sections 1 and 2
4	Specify the inclusion and exclusion criteria. Identify all ad-hoc exclusions.	Main text - Methods; Appendix 1 Section 2.1
5	Provide information on all included data sources and their main characteristics. For each data source used, report reference information or contact name/institution, population represented, data collection method, year(s) of data collection, sex and age range, diagnostic criteria or measurement method, and sample size, as relevant.	Main text - Data Sharing
6	Identify and describe any categories of input data that have potentially important biases (e.g., based on characteristics listed in item 5).	Appendix 1 - Sections 1.2 and 1.3; Appendix 2 – Tables S3-S4
For data inputs that contribute to the analysis but were not synthesized as part of the study:		
7	Describe and give sources for any other data inputs.	N/A
For all data inputs:		
8	Provide all data inputs in a file format from which data can be efficiently extracted (e.g., a spreadsheet rather than a PDF), including all relevant meta-data listed in item 5. For any data inputs that cannot be shared because of ethical or legal reasons, such as third-party ownership, provide a contact name or the name of the institution that retains the right to the data.	Main text - Data Sharing
Data analysis		
9	Provide a conceptual overview of the data analysis method. A diagram may be helpful.	Main text - Methods; Appendix 1 - Sections 1, 2, and 3
10	Provide a detailed description of all steps of the analysis, including mathematical formulae. This description should cover, as relevant, data cleaning, data pre-processing, data adjustments and weighting of data sources, and mathematical or statistical model(s).	Main text - Methods; Appendix 1 - Sections 1, 2, and 3
11	Describe how candidate models were evaluated and how the final model(s) were selected.	N/A
12	Provide the results of an evaluation of model performance, if done, as well as the results of any relevant sensitivity analysis.	N/A
13	Describe methods for calculating uncertainty of the estimates. State which sources of uncertainty were, and were not, accounted for in the uncertainty analysis.	Main text – Methods; Appendix 1 – Sections 1 and 2
14	State how analytic or statistical source code used to generate estimates can be accessed.	Main text - Data Sharing

Results and Discussion		
15	Provide published estimates in a file format from which data can be efficiently extracted.	Main text - Data sharing
16	Report a quantitative measure of the uncertainty of the estimates (e.g. uncertainty intervals).	Main text - Results; Supplementary Information 2
17	Interpret results in light of existing evidence. If updating a previous set of estimates, describe the reasons for changes in estimates.	Main text - Discussion
18	Discuss limitations of the estimates. Include a discussion of any modelling assumptions or data limitations that affect interpretation of the estimates.	Main text - Discussion

Section 2: Exposure modelling

Section 2.1: Exposure data

Section 2.1.1: Data identification

Data sources were identified through the Global Health Data Exchange (GHDx)[1], the GBD Collaborator Network, the World Health Organization (WHO) Global Database on the Prevalence of Violence against Women[2], and the United Nations Entity for Gender Equality and the Empowerment of Women (UN Women) Global Database on Violence against Women[3].

The GHDx is a catalogue of datasets from nearly every country in the world, and the most common types of data include demographic and health surveys, censuses, disease registries and other epidemiological surveillance systems, statistical yearbooks, and scientific literature. GHDx sources are identified through systematic reviews, expert knowledge, and targeted data seeking efforts by the Institute for Health Metrics and Evaluation and its individual and institutional collaborators. Currently, the GBD Collaborator network includes over 13,000 individuals in 163 countries who contribute to the data.

WHO's Global Database on Prevalence of Violence Against Women includes representative prevalence studies of sexual violence by any perpetrator. The database was created via a systematic review of six electronic databases by the WHO, the London School of Hygiene and Tropical Medicine, and the South Africa Medical Research Council. The WHO database also includes sources identified by manual searches for reports and surveys published and/or administered by local, national, and international governments and agencies (e.g., national violence studies, Demographic and Health Surveys).

Lastly, the UN Women's Global Database on Violence against Women contains relevant surveillance systems, reports, surveys, laws, and legislations submitted by United Nations Member States.

We reviewed and cross-referenced sources identified from each of these three databases. All data sources related to interpersonal violence and sexual violence against children were reviewed. Sources meeting inclusion criteria were extracted to be used in prevalence analyses.

Section 2.1.2: Inclusion criteria

We systematically extracted data from all sources from 1980 to 2023 that met the following inclusion criteria: were population-based; (2) reported on a sample representative of a national or subnational (i.e., first administrative level) location; and (3) measured self-reported instances of violence according to an

accepted definition of Intimate Partner Violence (IPV) and/or Sexual Violence against Children (SVAC) (described in next section).

Section 2.1.3 Case definitions

Our reference case definitions for IPV and SVAC were defined in alignment with GBD risk factor definitions. The GBD case definition for IPV is having ever experienced one or more acts of physical and/or sexual violence by a current or former intimate partner since the age of 15 years. IPV is estimated in females only because the existing evidence of risk-outcomes for males does not meet our inclusion criteria.

- Physical violence is defined as "being slapped or having something thrown at you that could hurt you, being pushed or shoved, being hit with a fist or something else that could hurt, being kicked, dragged, or beaten up, being choked or burnt on purpose, and/or being threatened with or actually having a gun, knife, or other weapon used on you."
- Sexual violence is defined as "being physically forced to have intercourse when you did not want to, having sexual intercourse because you were afraid of what your partner might do, and/or being forced to do something that you found humiliating or degrading." The definition of humiliating and degrading may vary across studies depending on the regional and cultural setting.
- Intimate partner is defined as "a partner to whom you are married or with whom you cohabit." In countries where people date, dating partners will also be considered (a partner with whom you have an intimate [sexual] relationship with but are not married to or cohabiting).

The GBD case definition for SVAC is having ever experienced intercourse or other contact violence (i.e., fondling and other sexual touching) before the age of 18 in which the contact was unwanted (i.e., physically forced or coerced).

For both IPV and SVAC, we additionally considered data using accepted alternate case definitions, which are presented in Table S1. Data using alternate case definitions were adjusted using adjustment factors derived from within-study comparisons of reference versus alternate case definitions (see more detail in Appendix sections 1.2.1 and 1.3.1).

Table S2: Reference and alternate case definitions used for Intimate Partner Violence and Sexual Violence against Children.

**Risk Factor**	**Reference Case Definition**	**Accepted Alternate Case Definitions**
Intimate Partner Violence	Lifetime prevalence of physical or sexual violence	• Lifetime prevalence of:<br>○ physical violence by a current or former intimate partner

	by a current or former intimate partner since age 15.	◦ sexual violence by a current or former intimate partner<br>• Past-year prevalence of:<br>◦ physical or sexual violence by a current or former intimate partner<br>◦ physical violence by a current or former intimate partner<br>◦ sexual violence by a current or former intimate partner<br>• Any of the above case definitions (or reference case definition), measured in samples of currently or ever-married females only.
Sexual Violence against Children	Lifetime prevalence of intercourse or other sexual contact (i.e., fondling and other sexual touching) before the age of 18 in which the contact was unwanted (i.e., physically forced or coerced).	• Lifetime prevalence of intercourse-only SVAC<br>• Lifetime prevalence of contact or non-contact SVAC<br>• Lifetime prevalence of SVAC in which the definition of perpetrator is restricted (e.g., sexual violence perpetrated by a caregiver)<br>• Lifetime prevalence of SVAC, defined as abuse before some age less than 18 (such as before age 12 or 15)<br>• Proportion of individuals whose first sexual debut was SVAC (i.e., before age 18 and forced, coerced, or unwanted)<br>• Proportion of individuals who experienced SVAC, measured from a student population

Section 2.1.4: Exposure sources lists

Table S3: Data sources used to estimate the prevalence of intimate partner violence

GHDx Record ID	GHDx Record Title	Geography	Suggested Citation
27321	Albania Reproductive Health Survey 2002	Albania	Albania Institute of Public Health (IPH), Ministry of Health (Albania), National Institute of Statistics (Albania), and Centers for Disease Control and Prevention. (2005) Albania Reproductive Health Survey 2001. Atlanta, United States: Centers for Disease Control and Prevention (CDC).
157018	Afghanistan Demographic and Health Survey 2015-2016	Afghanistan	Central Statistics Organization (Afghanistan), ICF International, Ministry of Public Health (Afghanistan). Afghanistan Demographic and Health Survey 2015-2016. Fairfax, United States of America: ICF International, 2017.

137276	A determination of the prevalence of gender-based violence among conflict-affected populations in East Timor	Timor-Leste	Hynes M, Robertson K, Ward J, Crouse C. A determination of the prevalence of gender-based violence among conflict-affected populations in East Timor. Disasters. 2004; 28(3): 294-321.
393876	Albania Demographic and Health Survey 2017-2018	Albania	Albania Institute of Public Health (IPH), Albania Institute of Statistics (INSTAT), ICF International. Albania Demographic and Health Survey 2017-2018. Fairfax, United States of America: ICF International, 2019.
150541	A Survey on Violence Against Female Partners in Québec, Canada	Canada	Rinfret-Raynor M, Riou A, Cantin S, Drouin C, Dubé M. A Survey on Violence Against Female Partners in Québec, Canada. Violence Against Women. 2004; 10(7): 709-28.
474217	Albania OSCE-Led Survey on the Well-Being and Safety of Women 2018	Albania	Ipsos, Organization for Security and Co-operation in Europe (OSCE). Albania OSCE-Led Survey on the Well-Being and Safety of Women 2018. 2019.
506103	Albania National Violence Against Women Survey 2018	Albania	Albania Institute of Statistics (INSTAT), United Nations Development Programme (UNDP), United Nations Entity for Gender Equality and the Empowerment of Women (UN Women). Albania National Violence Against Women Survey 2018.
506577	Abused, Battered, or Stalked: Violence in Intimate Partner Relations Gendered	Czechia	Buria?nek, J., Pikalkova, S., & Podana, Z. (2015). Abused, battered, or stalked?: violence in intimate partner relations gendered. Karolinum Press.
507509	Albania National Domestic Violence Survey 2013	Albania	Albania Institute of Statistics (INSTAT), United Nations Development Programme (UNDP). Albania National Domestic Violence Survey 2013.
218563	Armenia Demographic and Health Survey 2015-2016	Armenia	ICF International, Ministry of Health (Armenia), National Statistical Service of the Republic of Armenia. Armenia Demographic and Health Survey 2015-2016. Fairfax, United States of America: ICF International, 2017.
168024	Argentina - Buenos Aires Gender, Alcohol and Culture: An International Study (GENACIS) 2003	Argentina	Aarhus University, Addiction Switzerland Research Institute, Alcohol Research Group, Public Health Institute, Centre for Addiction and Mental Health (Canada), Centre for Alcohol Policy Research, Turning Point Alcohol and Drug Centre (Australia), Kettil Bruun Society for Social and Epidemiological Research on Alcohol, University of North Dakota. Argentina - Buenos Aires Gender, Alcohol and Culture: An International Study (GENACIS) 2003.
218555	Angola Demographic and Health Survey 2015-2016	Angola	ICF International, Ministry of Health (Angola), National Institute of Statistics (Angola), United Nations Children's Fund (UNICEF). Angola Demographic and Health Survey 2015-2016. Fairfax, United States of America: ICF International, 2017.

506916	Argentina National Study on Violence Against Women 2018	Argentina	Ministry of Justice and Human Rights (Argentina). Argentina National Study on Violence Against Women 2018. Argentina: Ministry of Justice and Human Rights (Argentina).
543717	An Analysis of Surveys on Domestic Violence by Japans Cabinet Office (1999-2017)	Japan	Suga T. An Analysis of Surveys on Domestic Violence by Japans Cabinet Office (1999-2017). Open J Soc Sci. 2018; 6(7): 56-66.
126029	Association of exposure to intimate-partner physical violence and potentially traumatic war-related events with mental health in Liberia	Liberia	Vinck P, Pham PN. Association of exposure to intimate-partner physical violence and potentially traumatic war-related events with mental health in Liberia. Soc Sci Med. 2013; 77: 41-9.
150452	Association of sexual violence and human rights violations with physical and mental health in territories of the Eastern Democratic Republic of the Congo	Democratic Republic of the Congo	Johnson K, Scott J, Rughita B, Kisielewski M, Asher J, Ong R, Lawry L. Association of sexual violence and human rights violations with physical and mental health in territories of the Eastern Democratic Republic of the Congo. JAMA. 2010; 304(5): 533-62.
150546	Association of Combatant Status and Sexual Violence With Health and Mental Health Outcomes in Postconflict Liberia	Liberia	Johnson K, Asher J, Rosborough S, Raja A, Panjabi R, Beadling C, Lawry L. Association of Combatant Status and Sexual Violence With Health and Mental Health Outcomes in Postconflict Liberia. JAMA. 2008; 300(6): 676-90.
506154	Armenia Nationwide Survey on Violence Against Women 2008	Armenia	National Statistical Service of the Republic of Armenia, United Nations Population Fund (UNFPA). Armenia Nationwide Survey on Violence Against Women 2008.
18865	Azerbaijan Demographic and Health Survey 2006	Azerbaijan	Macro International, Inc, State Statistical Committee of Azerbaijan. Azerbaijan Demographic and Health Survey 2006. Fairfax, United States of America: ICF International.
137014	Australia Personal Safety Survey 2005	Australia	Australian Bureau of Statistics. Australia Personal Safety Survey 2005.
341276	Australia Personal Safety Survey 2016-2017	Australia	Australian Bureau of Statistics, Department of Social Services (Australia). Australia Personal Safety Survey 2016-2017.
27338	Azerbaijan Reproductive Health Survey 2001	Azerbaijan	Adventist Development and Relief Agency (ADRA), Azerbaijan Ministry of Health, State Statistical Committee of Azerbaijan, and Centers for Disease Control and Prevention (CDC). (2003) Azerbaijan Reproductive Health Survey 2001. Atlanta, United States: Centers for Disease Control and Prevention (CDC).
133434	Australia Women's Safety Survey 1996	Australia	Australian Bureau of Statistics. Australia Women's Safety Survey 1996.
504375	Azerbaijan International Men and Gender Equality Study 2016	Azerbaijan	International Center for Research on Women, International Center for Social Research (ICSR) (Azerbaijan), Promundo, State Committee for Family, Women and Children's

			Affairs (Azerbaijan), United Nations Population Fund (UNFPA). Azerbaijan International Men and Gender Equality Study 2016.
516408	Australian Longitudinal Study on Women's Health 1989-1995 Cohort Summary Surveys 1-5 (2013-2017)	Australia	University of Newcastle (Australia), University of Queensland (Australia). Australian Longitudinal Study on Women's Health 1989-1995 Cohort Summary Surveys 1-5 (2013-2017). Australia: Australian Longitudinal Study on Women's Health, 2019.
18913	Bangladesh Demographic and Health Survey 2007	Bangladesh	Macro International, Inc, Mitra and Associates, National Institute of Population Research and Training (NIPORT). Bangladesh Demographic and Health Survey 2007. Fairfax, United States of America: ICF International, 2009.
95474	Bangladesh Urban Health Survey 2006	Bangladesh	Associates for Community and Population Research (ACPR), International Centre for Diarrhoeal Disease Research, Bangladesh (ICDDR,B), MEASURE Evaluation Project, Carolina Population Center, University of North Carolina, National Institute of Population Research and Training (NIPORT). Bangladesh Urban Health Survey 2006.
150513	Bangladesh WHO Multi-country Study on Women's Health and Domestic Violence Against Women 2001	Bangladesh	International Centre for Diarrhoeal Disease Research, Bangladesh (ICDDR,B), Naripokkho, Uppsala University, World Health Organization (WHO). Bangladesh WHO Multi-country Study on Women's Health and Domestic Violence Against Women 2001.
218565	Benin Demographic and Health Survey 2017-2018	Benin	Hubert Koutoukou Maga National University Hospital Center (CNHU-HKM)(Benin), ICF International, National Institute of Statistics and Economic Analysis (INSAE) (Benin), National Malaria Control Program, Ministry of Health (Benin), Permanent Secretariat of the Food Council and Nutrition (SP-CAN)(Benin). Benin Demographic and Health Survey 2017-2018. Fairfax, United States of America: ICF International, 2018.
80733	Belarus Multiple Indicator Cluster Survey 2012	Belarus	National Statistical Committee of the Republic of Belarus, United Nations Children's Fund (UNICEF). Belarus Multiple Indicator Cluster Survey 2012. New York, United States of America: United Nations Children's Fund (UNICEF), 2013.
169718	Belize Gender, Alcohol and Culture: An International Study (GENACIS) 2005	Belize	Aarhus University, Addiction Switzerland Research Institute, Alcohol Research Group, Public Health Institute, Centre for Addiction and Mental Health (Canada), Centre for Alcohol Policy Research, Turning Point Alcohol and Drug Centre (Australia), Kettil Bruun Society for Social and Epidemiological Research on Alcohol, University of North

			Dakota. Belize Gender, Alcohol and Culture: An International Study (GENACIS) 2005.
240312	Bermuda Adults Health Survey 2011	United Kingdom	Bermuda Health Council (BHeC), Department of Health (Bermuda), Mindmaps (Bermuda). Bermuda Adults Health Survey 2011.
506157	Belgium Emotional, Physical and Sexual Abuse Study 2009	Belgium	Ghent University, Institute for the Equality of Women and Men (Belgium), University of Liège (Belgium). Belgium Emotional, Physical and Sexual Abuse Study 2009.
539788	Benin Multiple Indicator Cluster Survey 2021-2022	Benin	National Institute of Statistics and Demography (INStaD) (Benin), United Nations Children's Fund (UNICEF). Benin Multiple Indicator Cluster Survey 2021-2022. New York, United States of America: United Nations Children's Fund (UNICEF), 2023.
286787	Botswana Gender Based Violence Indicators Study 2011	Botswana	Gender Links (South Africa), Women's Affairs Department, Ministry of Labour and Home Affairs (Botswana). Botswana Gender Based Violence Indicators Study 2011.
19001	Bolivia Demographic and Health Survey 2003-2004	Bolivia	Macro International, Inc, Ministry of Health and Sports (Bolivia), National Institute of Statistics (Bolivia). Bolivia Demographic and Health Survey 2003-2004. Fairfax, United States of America: ICF International.
134753	Botswana AIDS Impact Survey 2013	Botswana	Ministry of Health (Botswana), National AIDS Coordinating Agency (Botswana), Statistics Botswana. Botswana AIDS Impact Survey 2013. Gaborone, Botswana: Statistics Botswana, 2015.
19016	Bolivia Demographic and Health Survey 2008	Bolivia	Macro International, Inc, Ministry of Health and Sports (Bolivia), National Institute of Statistics (Bolivia). Bolivia Demographic and Health Survey 2008. Fairfax, United States of America: ICF International.
165290	Bhutan Health Survey 2012-2013	Bhutan	Ministry of Health (Bhutan), National Statistics Bureau (Bhutan), United Nations Population Fund (UNFPA). Bhutan Health Survey 2012-2013.
474219	Bosnia and Herzegovina OSCE-Led Survey on the Well-Being and Safety of Women 2018	Bosnia and Herzegovina	Ipsos, Organization for Security and Co-operation in Europe (OSCE). Bosnia and Herzegovina OSCE-Led Survey on the Well-Being and Safety of Women 2018.
504377	Bosnia and Herzegovina International Men and Gender Equality Study 2011	Bosnia and Herzegovina	CARE International, International Center for Research on Women, Perpetuum Mobile (Bosnia and Herzegovina), Promundo. Bosnia and Herzegovina International Men and Gender Equality Study 2011.
504378	Bolivia International Men and Gender Equality Study 2019	Bolivia	Equimundo, International Center for Research on Women, Private University of Bolivia. Bolivia International Men and Gender Equality Study 2019.
506160	Bhutan National Study on Women's Health and Life Experiences 2017	Bhutan	National Commission for Women and Children (NCWC) (Bhutan). Bhutan National Study on Women's Health and Life Experiences 2017.

506199	Bolivia Situation of Violence against Women, Trafficking and Smuggling 2015	Bolivia	Community for Social Studies and Political Action - Ciudadanía (Bolivia), Free Alliance Without Violence (Bolivia). Bolivia Situation of Violence against Women, Trafficking and Smuggling 2015.
506223	Bosnia and Herzegovina Prevalence and Characteristics of Violence Against Women 2012	Bosnia and Herzegovina	Agency for Gender Equality (Bosnia and Herzegovina), Gender Center of Republika Srpska, Gender Center of the Federation of Bosnia and Herzegovina, United Nations Entity for Gender Equality and the Empowerment of Women (UN Women), United Nations Population Fund (UNFPA). Bosnia and Herzegovina Prevalence and Characteristics of Violence Against Women 2012.
508129	Bolivia Survey on the Prevalence and Characteristics of Violence Against Women 2016	Bolivia	National Institute of Statistics (Bolivia). Bolivia Survey on the Prevalence and Characteristics of Violence Against Women 2016.
142941	Brazil National Alcohol and Drugs Survey 2011-2012	Brazil	Ipsos, National Institute of Public Policy for Alcohol and Other Drugs (INPAD) (Brazil), University of São Paulo. Brazil National Alcohol and Drugs Survey 2011-2012.
195010	Brazil National Survey of Health 2013	Brazil	Brazilian Institute of Geography and Statistics (IBGE), Ministry of Health (Brazil), Ministry of Planning, Budget, and Management (Brazil). Brazil National Survey of Health 2013. Rio de Janeiro, Brazil: Brazilian Institute of Geography and Statistics (IBGE).
281642	Brazil National Survey of School Health 2015	Brazil	Brazilian Institute of Geography and Statistics (IBGE). Brazil National Survey of School Health 2015. Rio de Janeiro, Brazil: Brazilian Institute of Geography and Statistics (IBGE), 2016.
169719	Brazil - Botucatu Gender, Alcohol and Culture: An International Study (GENACIS) 2001-2002	Brazil	Aarhus University, Addiction Switzerland Research Institute, Alcohol Research Group, Public Health Institute, Centre for Addiction and Mental Health (Canada), Centre for Alcohol Policy Research, Turning Point Alcohol and Drug Centre (Australia), Kettil Bruun Society for Social and Epidemiological Research on Alcohol, University of North Dakota. Brazil - Botucatu Gender, Alcohol and Culture: An International Study (GENACIS) 2001-2002.
466849	Brazil National Health Survey 2019	Brazil	Brazilian Institute of Geography and Statistics (IBGE), Ministry of Economy (Brazil), Ministry of Health (Brazil). Brazil National Health Survey 2019. Rio de Janeiro, Brazil: Brazilian Institute of Geography and Statistics (IBGE).
238854	Cambodia WHO Multi-country Study on Women's Health and Domestic	Cambodia	Ministry of Women's Affairs (Cambodia), National Institute of Statistics (Cambodia), World Health Organization (WHO). Cambodia WHO Multi-country Study on

	Violence Against Women 2015		Women's Health and Domestic Violence Against Women 2015. Phnom Penh, Cambodia: National Institute of Statistics (Cambodia).
286766	Burundi Demographic and Health Survey 2016-2017	Burundi	Burundi Institute of Statistics and Economic Studies, ICF International, Ministry of Public Health and the Fight Against AIDS (Burundi). Burundi Demographic and Health Survey 2016-2017. Fairfax, United States of America: ICF International, 2018.
19133	Burkina Faso Demographic and Health Survey 2010-2011	Burkina Faso	ICF Macro, Ministry of Health (Burkina Faso), National Institute of Statistics and Demography (Burkina Faso). Burkina Faso Demographic and Health Survey 2010-2011. Fairfax, United States of America: ICF International.
150514	Brazil WHO Multi-country Study on Women's Health and Domestic Violence Against Women 2000-2001	Brazil	Federal University of Pernambuco, Feminist Collective for Health and Sexuality (São Paulo), University of São Paulo, World Health Organization (WHO). Brazil WHO Multi-country Study on Women's Health and Domestic Violence Against Women 2000-2001.
493322	Burkina Faso Performance Monitoring and Action Phase 2 Household and Female Survey 2020-2021	Burkina Faso	Bill and Melinda Gates Institute for Population and Reproductive Health, Johns Hopkins Bloomberg School of Public Health, Higher Institute of Population Sciences (ISSP) (Burkina Faso), Jhpiego. Burkina Faso Performance Monitoring and Action Phase 2 Household and Female Survey 2020-2021. Baltimore, United States of America: Johns Hopkins Bloomberg School of Public Health, 2021.
527622	Burkina Faso Demographic and Health Survey 2021	Burkina Faso	ICF International, National Institute of Public Health (INSP) (Burkina Faso), National Institute of Statistics and Demography (Burkina Faso). Burkina Faso Demographic and Health Survey 2021.
157024	Cambodia Demographic and Health Survey 2014	Cambodia	ICF International, Ministry of Health (Cambodia), National Institute of Statistics (Cambodia). Cambodia Demographic and Health Survey 2014. Fairfax, United States of America: ICF International, 2017.
231764	Cambodia Violence Against Children Survey 2013	Cambodia	Centers for Disease Control and Prevention (CDC), Ministry of Social Affairs, Veterans and Youth Rehabilitation (MoSVY) (Cambodia), Ministry of Women's Affairs (Cambodia), National Institute of Statistics (Cambodia), Together for Girls, United Nations Children's Fund (UNICEF). Cambodia Violence Against Children Survey 2013. Washington, DC, United States of America: Together for Girls.
19274	Cameroon Demographic and Health Survey 2011	Cameroon	ICF International, Ministry of Economy, Planning and Regional Development

			(Cameroon), Ministry of Public Health (Cameroon), National Institute of Statistics (Cameroon), Pasteur Center of Cameroon. Cameroon Demographic and Health Survey 2011. Fairfax, United States of America: ICF International.
413167	Cameroon Demographic and Health Survey 2018-2019	Cameroon	ICF International, Ministry of Public Health (Cameroon), National Institute of Statistics (Cameroon). Cameroon Demographic and Health Survey 2018-2019. Fairfax, United States of America: ICF International.
19167	Cambodia Demographic and Health Survey 2005-2006	Cambodia	Macro International, Inc, National Institute of Public Health (Cambodia), National Institute of Statistics (Cambodia). Cambodia Demographic and Health Survey 2005-2006. Fairfax, United States of America: ICF International.
19156	Cambodia Demographic and Health Survey 2000	Cambodia	Macro International, Inc, Ministry of Health (Cambodia), National Institute of Statistics (Cambodia). Cambodia Demographic and Health Survey 2000. Fairfax, United States of America: ICF International.
19211	Cameroon Demographic and Health Survey 2004	Cameroon	Macro International, Inc, National Institute of Statistics (Cameroon). Cameroon Demographic and Health Survey 2004. Fairfax, United States of America: ICF International.
358376	Cameroon Population-Based HIV Impact Assessment 2017-2018	Cameroon	Centers for Disease Control and Prevention (CDC), Global Health System Solutions (GHSS) (Cameroon), ICAP, Columbia University Mailman School of Public Health, International Reference Center ""Chantal Biya"" (CIRCB) (Cameroon), Ministry of Public Health (Cameroon), National AIDS Control Committee (CNLS) (Cameroon), National Institute of Statistics (Cameroon), National Public Health Laboratory (Cameroon), Pasteur Center of Cameroon. Cameroon Population-Based HIV Impact Assessment 2017-2018. New York, New York: ICAP, Columbia University Mailman School of Public Health, 2021.
503522	Cambodia United Nations Multi-country Study on Men and Violence 2012 - Equimundo	Cambodia	Partners for Prevention, United Nations Development Programme (UNDP), United Nations Entity for Gender Equality and the Empowerment of Women (UN Women), United Nations Population Fund (UNFPA), United Nations Volunteers (UNV). Cambodia United Nations Multi-country Study on Men and Violence 2012 - Equimundo.
507744	Cambodia Violence Against Women Survey 2005	Cambodia	Indochina Research Ltd. (IRL), Ministry of Women's Affairs (Cambodia). Cambodia Violence Against Women Survey 2005.
523643	Cambodia Demographic and Health Survey 2021-2022	Cambodia	ICF International, Ministry of Health (Cambodia), National Institute of Statistics

			(Cambodia). Cambodia Demographic and Health Survey 2021-2022. Fairfax, United States of America: ICF International, 2023.
540286	Cambodia Violence Against Women Survey 2009	Cambodia	German Technical Cooperation Agency (GTZ), Indochina Research Ltd. (IRL), Ministry of Women's Affairs (Cambodia). Cambodia Violence Against Women Survey 2009.
157025	Chad Demographic and Health Survey 2014-2015	Chad	ICF International, National Institute for Statistics, Economic and Demographic Studies (INSEED) (Chad). Chad Demographic and Health Survey 2014-2015. Fairfax, United States of America: ICF International, 2016.
76701	Chad Multiple Indicator Cluster Survey 2010	Chad	Ministry of Planning, Economy, and International Cooperation (Chad), National Institute for Statistics, Economic and Demographic Studies (INSEED) (Chad), United Nations Children's Fund (UNICEF). Chad Multiple Indicator Cluster Survey 2010. New York, United States of America: United Nations Children's Fund (UNICEF), 2014.
2223	Central African Republic Multiple Indicator Cluster Survey 2006	Central African Republic	United Nations Children's Fund (UNICEF). Central African Republic Multiple Indicator Cluster Survey 2006. New York, United States: United Nations Children's Fund (UNICEF).
169720	Canada Gender, Alcohol and Culture: An International Study (GENACIS) 2004	Canada	Aarhus University, Addiction Switzerland Research Institute, Alcohol Research Group, Public Health Institute, Centre for Addiction and Mental Health (Canada), Centre for Alcohol Policy Research, Turning Point Alcohol and Drug Centre (Australia), Kettil Bruun Society for Social and Epidemiological Research on Alcohol, University of North Dakota. Canada Gender, Alcohol and Culture: An International Study (GENACIS) 2004.
21442	Cape Verde Demographic and Health Survey 2005	Cabo Verde	Macro International, Inc, Ministry of Health (Cape Verde), National Institute of Statistics (Cape Verde). Cape Verde Demographic and Health Survey 2005.
137211	Canada General Social Survey on Personal Risk 1993	Canada	Statistics Canada (StatCan). Canada General Social Survey on Personal Risk 1993. Ottawa, Canada: Statistics Canada (StatCan), 1995.
137213	Canada General Social Survey on Victimization 1999	Canada	Statistics Canada (StatCan). Canada General Social Survey on Victimization 1999. Ottawa, Canada: Statistics Canada (StatCan), 2000.
137214	Canada General Social Survey on Victimization 2004	Canada	Statistics Canada (StatCan). Canada General Social Survey on Victimization 2004. Ottawa, Canada: Statistics Canada (StatCan), 2005.
137217	Canada General Social Survey on Victimization 2014	Canada	Statistics Canada (StatCan). Canada General Social Survey on Victimization 2014. 2015.

507764	Canada Violence Against Women Survey 1993	Canada	Health Canada, Statistics Canada (StatCan). Canada Violence Against Women Survey 1993.
524155	Cape Verde Demographic and Reproductive Health Survey 2018	Cabo Verde	National Institute of Statistics (Cape Verde), Utica International. Cape Verde Demographic and Reproductive Health Survey 2018. Praia, Cabo Verde: National Institute of Statistics (Cape Verde), 2020.
137181	Childhood Abuse as a Risk Factor for Adolescent Pregnancy in El Salvador	El Salvador	Pallitto CC, Murillo V. Childhood Abuse as a Risk Factor for Adolescent Pregnancy in El Salvador. J Adolesc Health. 2008; 42(6): 580-6.
139804	Child sexual abuse revisited: a population-based cross-sectional study among swiss adolescents	Switzerland	Mohler-Kuo M, Landolt MA, Maier T, Meidert U, Schönbucher V, Schnyder U. Child sexual abuse revisited: a population-based cross-sectional study among swiss adolescents. J Adolesc Health. 2014; 54(3): 304?311e1.
31903	China Health and Family Life Survey 1999-2000	China	National Opinion Research Center, University of Chicago (NORC), Renmin University, Beijing, Peking Union Medical College, Beijing, University of North Carolina. China Health and Family Life Survey 1999-2000. Chicago, USA: Population Research Center, University of Chicago.
19324	Colombia Demographic and Health Survey 2004-2005	Colombia	Macro International, Inc, Profamilia (Colombia). Colombia Demographic and Health Survey 2004-2005. Fairfax, United States of America: ICF International, 2005.
21281	Colombia Demographic and Health Survey 2009-2010	Colombia	ICF Macro, Profamilia (Colombia). Colombia Demographic and Health Survey 2009-2010. Fairfax, United States of America: ICF International, 2011.
218566	Colombia Demographic and Health Survey 2015-2016	Colombia	ICF International, Ministry of Health (Colombia), Profamilia (Colombia). Colombia Demographic and Health Survey 2015-2016. Fairfax, United States of America: ICF International, 2017.
19359	Colombia Demographic and Health Survey 2000	Colombia	Macro International, Inc, Profamilia (Colombia). Colombia Demographic and Health Survey 2000. Fairfax, United States of America: ICF International, 2000.
437431	Chile National Survey of Victimization for Domestic Violence and Sexual Crimes 2008	Chile	Center for the Study and Analysis of Crime (CEAD) (Chile), Directorate of Social Studies UC (DESUC), Pontifical Catholic University of Chile (Chile). Chile National Survey of Victimization for Domestic Violence and Sexual Crimes 2008. Chile: Center for the Study and Analysis of Crime (CEAD) (Chile).
437432	Chile National Survey of Victimization for Domestic Violence and Sexual Crimes 2012	Chile	Center for the Study and Analysis of Crime (CEAD) (Chile), GfK Adimark (Chile). Chile National Survey of Victimization for Domestic Violence and Sexual Crimes 2012. Chile: Center for the Study and Analysis of Crime (CEAD) (Chile).

437433	Chile National Survey of Victimization for Domestic Violence and Sexual Crimes 2016-2017	Chile	Center for the Study and Analysis of Crime (CEAD) (Chile). Chile National Survey of Victimization for Domestic Violence and Sexual Crimes 2016-2017. Chile: Center for the Study and Analysis of Crime (CEAD) (Chile).
503500	China United Nations Multi-country Study on Men and Violence 2011 - Equimundo	China	Anti-Domestic Violence Network of China, Partners for Prevention, United Nations Population Fund (UNFPA). China United Nations Multi-country Study on Men and Violence 2011 - Equimundo.
504539	Chile International Men and Gender Equality Study 2009	Chile	EME Masculinities and Gender Equity (Chile), International Center for Research on Women, Promundo. Chile International Men and Gender Equality Study 2009.
522151	Chile National Survey on Violence Against Women in the Field of Intrafamily Violence and in Other Spaces 2019-2020	Chile	Center for the Study and Analysis of Crime (CEAD) (Chile), Ministry of the Interior and Public Security (Chile). Chile National Survey on Violence Against Women in the Field of Intrafamily Violence and in Other Spaces 2019-2020. Chile: Center for the Study and Analysis of Crime (CEAD) (Chile).
76850	Comoros Demographic and Health Survey 2012-2013	Comoros	General Directorate of Statistics and Forecasting (Comoros), ICF International. Comoros Demographic and Health Survey 2012-2013. Fairfax, United States of America: ICF International.
150590	Comparative Reanalysis of Prevalence of Violence Against Women and Health Impact Data in Europe - Obstacles and Possible Solutions	Finland; France; Germany; Lithuania; Sweden	Co-ordination Action on Human Rights Violations (CAHRV). Comparative Reanalysis of Prevalence of Violence Against Women and Health Impact Data in Europe - Obstacles and Possible Solutions. Osnabrück, Germany: Co-ordination Action on Human Rights Violations (CAHRV), 2006. (CAHRV-Report 2006. Co-ordination Action on Human Rights Violations funded through the European Commission, 6th Framework Programme, Project No. 506348).
465288	Colombia Violence Against Children and Youth Survey 2018	Colombia	Centers for Disease Control and Prevention (CDC), International Organization for Migration (IOM), Ministry of Health and Social Protection (Colombia), Together for Girls. Colombia Violence Against Children and Youth Survey 2018. Bogotá, Colombia: Ministry of Health and Social Protection (Colombia).
541281	Comoros Multiple Indicator Cluster Survey 2022	Comoros	National Institute of Statistics and Economic and Demographic Studies (INSEED) (Comoros), United Nations Children's Fund (UNICEF). Comoros Multiple Indicator Cluster Survey 2022. New York, United States of America: United Nations Children's Fund (UNICEF), 2023.

18533	Côte d'Ivoire Demographic and Health Survey 2011-2012	Côte d'Ivoire	ICF International, Ministry of the Fight Against AIDS (Côte d'Ivoire), National Institute of Statistics (Côte d'Ivoire). Côte d'Ivoire Demographic and Health Survey 2011-2012. Fairfax, United States of America: ICF International.
169723	Costa Rica Gender, Alcohol and Culture: An International Study (GENACIS) 2003	Costa Rica	Aarhus University, Addiction Switzerland Research Institute, Alcohol Research Group, Public Health Institute, Centre for Addiction and Mental Health (Canada), Centre for Alcohol Policy Research, Turning Point Alcohol and Drug Centre (Australia), Kettil Bruun Society for Social and Epidemiological Research on Alcohol, University of North Dakota. Costa Rica Gender, Alcohol and Culture: An International Study (GENACIS) 2003.
500885	Cook Islands Family Health and Safety Study 2012-2013	New Zealand	Cook Islands Statistics Office, Ministry of Health (Cook Islands), National Council of Women (Cook Islands). Cook Islands Family Health and Safety Study 2012-2013. 2014.
513249	Cote d'Ivoire Performance Monitoring and Action Phase 2 Household and Female Survey 2021-2022	Côte d'Ivoire	Bill and Melinda Gates Institute for Population and Reproductive Health, Johns Hopkins Bloomberg School of Public Health, Jhpiego, National School for Statistics and Economics Applied (ENSEA). Cote d'Ivoire Performance Monitoring and Action Phase 2 Household and Female Survey 2021-2022. Baltimore, United States of America: Johns Hopkins Bloomberg School of Public Health, 2024.
515016	Costa Rica National Survey of Sexual and Reproductive Health 2015	Costa Rica	Central American Population Center, University of Costa Rica, Costa Rican Demographic Association, Costa Rican Social Security Fund (CCCS), Ministry of Health (Costa Rica), National Institute of Statistics and Censuses (Costa Rica), United Nations Population Fund (UNFPA). Costa Rica National Survey of Sexual and Reproductive Health 2015. San José, Costa Rica: Central American Population Center, University of Costa Rica, 2016.
531039	Cote d'Ivoire Performance Monitoring and Action Phase 3 Household and Female Survey 2022	Côte d'Ivoire	Bill and Melinda Gates Institute for Population and Reproductive Health, Johns Hopkins Bloomberg School of Public Health, Jhpiego, National School for Statistics and Economics Applied (ENSEA). Cote d'Ivoire Performance Monitoring and Action Phase 3 Household and Female Survey 2022. Baltimore, United States of America: Johns Hopkins Bloomberg School of Public Health, 2024.
535004	Côte d'Ivoire Demographic and Health Survey 2021	Côte d'Ivoire	ICF International, Ministry of Health and Public Hygiene (MSHP) (Côte d'Ivoire), National Institute of Statistics (Côte d'Ivoire). Côte d'Ivoire Demographic and Health Survey

			2021. Fairfax, United States of America: ICF International, 2023.
169721	Czech Republic Gender, Alcohol and Culture: An International Study (GENACIS) 2002	Czechia	Aarhus University, Addiction Switzerland Research Institute, Alcohol Research Group, Public Health Institute, Centre for Addiction and Mental Health (Canada), Centre for Alcohol Policy Research, Turning Point Alcohol and Drug Centre (Australia), Kettil Bruun Society for Social and Epidemiological Research on Alcohol, University of North Dakota. Czech Republic Gender, Alcohol and Culture: An International Study (GENACIS) 2002.
506251	Cuba National Survey on Gender Equality 2016	Cuba	Federation of Cuban Women, National Office of Statistics and Information (Cuba). Cuba National Survey on Gender Equality 2016.
506259	Cyprus Extent, Frequency, Nature and Consequences of Domestic Violence Against Women 2012	Cyprus	Advisory Committee for the Prevention and Combating of Violence in the Family (Cyprus). Cyprus Extent, Frequency, Nature and Consequences of Domestic Violence Against Women 2012.
524832	Cross-lagged relationships between substance use and intimate partner violence among a sample of young adult women	United States of America	Martino SC, Collins RL, Ellickson PL. Cross-lagged relationships between substance use and intimate partner violence among a sample of young adult women. J Stud Alcohol. 2005; 66(1): 139-48.
19381	Democratic Republic of the Congo Demographic and Health Survey 2007	Democratic Republic of the Congo	Macro International, Inc, Ministry of Planning (Congo, DR). Democratic Republic of the Congo Demographic and Health Survey 2007. Fairfax, United States of America: ICF International.
76878	Democratic Republic of the Congo Demographic and Health Survey 2013-2014	Democratic Republic of the Congo	ICF International, Ministry of Planning and Monitoring Implementation of the Revolution of Modernity (Congo, DR), Ministry of Public Health (Congo, DR), National Institute of Statistics (Congo, DR). Democratic Republic of the Congo Demographic and Health Survey 2013-2014. Fairfax, United States of America: ICF International, 2014.
137280	Dating violence against adolescent girls and associated substance use, unhealthy weight control, sexual risk behavior, pregnancy, and suicidality	United States of America	Silverman JG, Raj A, Mucci LA, Hathaway JE. Dating violence against adolescent girls and associated substance use, unhealthy weight control, sexual risk behavior, pregnancy, and suicidality. JAMA. 2001; 286(5): 572-9.
128664	Domestic violence in India: insights from the 2005-2006 national family health survey	India	Kimuna SR, Djamba YK, Ciciurkaite G, Cherukuri S. Domestic violence in India: insights from the 2005-2006 national family health survey. J Interpers Violence. 2013; 28(4): 773-807.
150545	Domestic violence and women?s mental health in Japan	Japan	Weingourt R, Maruyama T, Sawada I, Yoshino J. Domestic violence and women?s mental health in Japan. Int Nurs Rev. 2001; 48(2): 102-8.

150617	Descriptive epidemiology of intimate partner aggression in Ukraine	Ukraine	O'Leary KD, Tintle N, Bromet EJ, Gluzman SF. Descriptive epidemiology of intimate partner aggression in Ukraine. Soc Psychiatry Psychiatr Epidemiol. 2008; 43(8): 619-26.
238505	Domestic violence against women in Eastern Sudan	Sudan	Ali AA, Yassin K, Omer R. Domestic violence against women in Eastern Sudan. BMC Public Health. 2014; 14: 1136.
505706	Domestic violence and its predictors among married women in reproductive age in Fagitalekoma Woreda, Awi zone, Amhara regional state, North Western Ethiopia	Ethiopia	Semahegn A, Belachew T, Abdulahi M. Domestic violence and its predictors among married women in reproductive age in Fagitalekoma Woreda, Awi zone, Amhara regional state, North Western Ethiopia. Reprod Health. 2013; 10: 63.
505930	Domestic Violence and Womens Well-being in Malaysia: Issues and Challenges Conducting a National Study Using the WHO Multi-country Questionnaire on Womens Health and Domestic Violence Against Women	Malaysia	Shuib R, Endut N, Ali SH, Osman I, Abdullah S, Oon SW, Ghani PA, Prabakaran G, Hussin NS, Shahrudin SSH. Domestic Violence and Womens Well-being in Malaysia: Issues and Challenges Conducting a National Study Using the WHO Multi-country Questionnaire on Womens Health and Domestic Violence Against Women. Procedia Soc Behav Sci. 2013; 91: 475-88.
77819	Dominican Republic Demographic and Health Survey 2013	Dominican Republic	Center for Social and Demographic Studies (Dominican Republic) (CESDEM), ICF International, Ministry of Public Health and Social Assistance (Dominican Republic). Dominican Republic Demographic and Health Survey 2013. Fairfax, United States of America: ICF International, 2014.
153700	Ecuador National Survey of Family Relationships and Gender Violence Against Women 2011	Ecuador	Ministry of the Interior (Ecuador), National Institute of Statistics and Censuses (Ecuador), Transition Committee to the Council on Women and Gender Equality (Ecuador). Ecuador National Survey of Family Relationships and Gender Violence Against Women 2011.
19444	Dominican Republic Demographic and Health Survey 2002	Dominican Republic	Center for Social and Demographic Studies (Dominican Republic) (CESDEM), Macro International, Inc. Dominican Republic Demographic and Health Survey 2002. Fairfax, United States of America: ICF International.
19456	Dominican Republic Demographic and Health Survey 2007	Dominican Republic	Center for Social and Demographic Studies (Dominican Republic) (CESDEM), Macro International, Inc. Dominican Republic Demographic and Health Survey 2007. Fairfax, United States of America: ICF International.
27621	Ecuador Reproductive Health Survey 1999	Ecuador	Center for Studies of Population and Social Development (CEPAR) (Ecuador), Division of Reproductive Health-Centers for Disease Control and Prevention (CDC). Ecuador Reproductive Health Survey 1999. Atlanta,

			United States: Centers for Disease Control and Prevention (CDC), 2001.
19431	Dominican Republic Experimental Demographic and Health Survey 1999	Dominican Republic	Center for Social and Demographic Studies (Dominican Republic) (CESDEM), Macro International, Inc. Dominican Republic Experimental Demographic and Health Survey 1999. Fairfax, United States of America: ICF International.
27630	Ecuador Reproductive Health Survey 2004	Ecuador	Center for Studies of Population and Social Development (CEPAR) (Ecuador) and Division of Reproductive Health-Centers for Disease Control and Prevention (CDC). (2005) Ecuador Reproductive Health Survey 2004. Quito, Ecuador: CEPAR.
137258	Domestic violence in South Australia: a population survey of males and females	Australia	Grande ED, Hickling J, Taylor A, Woollacott T. Domestic violence in South Australia: a population survey of males and females. Aust N Z J Public Health. 2003; 27(5): 543-50.
150472	Domestic violence in rural Uganda : evidence from a community-based study	Uganda	Koenig MA, Lutalo T, Zhao F, Nalugoda F, Wabwire-Mangen F, Kiwanuka N, Wagman J, Serwadda D, Wawer M, Gray R. Domestic violence in rural Uganda : evidence from a community-based study. Bull World Health Organ. 2003; 81(1): 53-60.
408852	El Salvador Violence Against Children, Girls and Adolescents Survey 2017	El Salvador	Centers for Disease Control and Prevention (CDC), General Directorate of Statistics and Census (DIGESTYC) (El Salvador), International Organization for Migration (IOM), Ministry of Justice and Public Safety (El Salvador), Together for Girls. El Salvador Violence Against Children, Girls and Adolescents Survey 2017.
19521	Egypt Demographic and Health Survey 2005	Egypt	El-Zanaty and Associates, Macro International, Inc, Ministry of Health and Population (Egypt), Population Council (Egypt). Egypt Demographic and Health Survey 2005. Fairfax, United States of America: ICF International.
27606	El Salvador Reproductive Health Survey 2008	El Salvador	Asociación Demográfica Salvadoreña (ADS), Division of Reproductive Health-Centers for Disease Control and Prevention (CDC). (2009) El Salvador Reproductive Health Survey 2008. San Salvador, El Salvador: ADS.
154897	Egypt Demographic and Health Survey 2014	Egypt	El-Zanaty and Associates, ICF International, Ministry of Health and Population (Egypt). Egypt Demographic and Health Survey 2014. Fairfax, United States of America: ICF International.
27599	El Salvador Reproductive Health Survey 2002-2003	El Salvador	Asociación Demográfica Salvadoreña (ADS), Division of Reproductive Health-Centers for Disease Control and Prevention (CDC). (2004) El Salvador Reproductive Health Survey 2002-2003. San Salvador, El Salvador: ADS.

76884	Equatorial Guinea Demographic and Health Survey 2011	Equatorial Guinea	ICF International, Ministry of Health and Social Welfare (Equatorial Guinea), Ministry of Planning, Economic Development and Public Investment (Equatorial Guinea). Equatorial Guinea Demographic and Health Survey 2011. Fairfax, United States of America: ICF International, 2012.
151544	Epidemiological Survey of Spousal Abuse in South Korea	Republic of Korea	Kim KI, Cho YG. Epidemiological Survey of Spousal Abuse in Korea. In: Intimate Violence: Interdisciplinary Perspectives. Viano E, ed. Washington, D.C., United States: Hemisphere Publishing Corporation, 1992. p. 277-82.
506323	Egypt Economic Gender-Based Violence Survey 2015	Egypt	Central Agency for Public Mobilization and Statistics (CAPMAS) (Egypt), United Nations Population Fund (UNFPA). Egypt Economic Gender-Based Violence Survey 2015.
506722	El Salvador Violence Against Women Study 2014	El Salvador	Pan American Health Organization (PAHO), Technological University of El Salvador, University of Granada (Spain), World Health Organization (WHO). El Salvador Violence Against Women Study 2014.
218568	Ethiopia Demographic and Health Survey 2016	Ethiopia	Central Statistics Agency (Ethiopia), ICF International. Ethiopia Demographic and Health Survey 2016. Fairfax, United States of America: ICF International, 2017.
135626	European Union Violence Against Women Study 2012	Austria; Belgium; Bulgaria; Croatia; Cyprus; Czechia; Denmark; Estonia; Finland; France; Germany; Greece; Hungary; Ireland; Italy; Latvia; Lithuania; Luxembourg; Malta; Netherlands; Poland; Portugal; Romania; Slovakia; Slovenia; Spain; Sweden; United Kingdom	European Union Agency for Fundamental Rights. European Union Violence Against Women Study 2012.
272644	Ethiopia STEPS Noncommunicable Disease Risk Factors Survey 2015	Ethiopia	Ethiopian Public Health Institute (EPHI), World Health Organization (WHO). Ethiopia STEPS Noncommunicable Disease Risk Factors Survey 2015.
137165	Exploring the associations between intimate partner violence and women's mental health: Evidence from a population-based study in Paraguay	Paraguay	Ishida K, Stupp P, Melian M, Serbanescu F, Goodwin M. Exploring the associations between intimate partner violence and women's mental health: Evidence from a population-based study in Paraguay. Soc Sci Med. 2010; 71(9): 1653-61.
137264	Experiences with sexual aggression within the	Czechia	Weiss P, Zverina J. Experiences with sexual aggression within the general population in

	general population in the Czech Republic		the Czech Republic. Arch Sex Behav. 1999; 28(3): 265-9.
500887	Federated States of Micronesia Family Health and Safety Study 2014	Micronesia (Federated States of)	Department of Health and Social Affairs (Micronesia). Federated States of Micronesia Family Health and Safety Study 2014. 2014.
519436	Eswatini Violence Against Children and Youth Survey 2021	Eswatini	Centers for Disease Control and Prevention (CDC), Central Statistical Office (Eswatini), ICAP, Columbia University Mailman School of Public Health, Ministry of Health (Eswatini), President's Emergency Plan for AIDS Relief (PEPFAR). Eswatini Violence Against Children and Youth Survey 2021.
137218	Finland Women's Safety Survey 1997-1998	Finland	Statistics Finland. Finland Women's Safety Survey 1997-1998.
150574	France National Survey of Violence Against Women 2000	France	French Institute of Health and Medical Research (INSERM), National Center for Scientific Research (CNRS) (France), National Institute for Demographic Studies (France), Paris Demography Institute (IDUP). France National Survey of Violence Against Women 2000.
506328	Fiji National Research on Women's Health and Life Experiences 2010-2011	Fiji	Fiji Women's Crisis Centre. Fiji National Research on Women's Health and Life Experiences 2010-2011.
506329	Finland Violence Against Women Survey 2005-2006	Finland	Statistics Finland. Finland Violence Against Women Survey 2005-2006.
508288	France Violence and Gender Relations Survey 2015	France	National Institute of Demographic Studies (INED) (France). France Violence and Gender Relations Survey 2015.
27494	Georgia Reproductive Health Survey 2005	Georgia	Georgia Center for Disease Control (NCDC), Georgian Ministry of Labor Health and Social Affairs (MOLHSA), Division of Reproductive Health, Centers for Disease Control and Prevention (CDC). Georgia Reproductive Health Survey 2005. Atlanta, United States: Centers for Disease Control and Prevention (CDC).
27486	Georgia Reproductive Health Survey 1999-2000	Georgia	Georgia Center for Disease Control (NCDC), Georgia Ministry of Labor, Health and Social Affairs (MOLHSA), Division of Reproductive Health-Centers for Disease Control and Prevention (CDC). (2001) Georgia Reproductive Health Survey 1999-2000. Atlanta, United States: Centers for Disease Control and Prevention (CDC).
77384	Gambia Demographic and Health Survey 2013	Gambia	Gambia Bureau of Statistics (GBOS), ICF International, Ministry of Health and Social Welfare (The Gambia). Gambia Demographic and Health Survey 2013. Fairfax, United States of America: ICF International, 2015.
95336	Georgia Reproductive Health Survey 2010-2011	Georgia	Division of Reproductive Health, Centers for Disease Control and Prevention (CDC), Georgia Ministry of Labor, Health and Social Affairs, National Center for Disease Control and Public Health (Georgia), National

			Statistics Office of Georgia (GeoStat). Georgia Reproductive Health Survey 2010-2011.
76706	Gabon Demographic and Health Survey 2012	Gabon	General Directorate of Statistics (Gabon), ICF International, Ministry of Economy, Employment and Sustainable Development (Gabon), Ministry of Health and Social Affairs (Gabon). Gabon Demographic and Health Survey 2012. Fairfax, United States of America: ICF International, 2013.
459854	Gambia Demographic and Health Survey 2019-2020	Gambia	Gambia Bureau of Statistics (GBOS), ICF International, Ministry of Health and Social Welfare (The Gambia). Gambia Demographic and Health Survey 2019-2020. Fairfax, United States of America: ICF International, 2021.
505891	Gender-based violence and sexual and reproductive health among low-income youth in three Brazilian cities	Brazil	Chacham AS, Simão ABS, Caetano AJ. Gender-based violence and sexual and reproductive health among low-income youth in three Brazilian cities. Reprod Health Matters. 2016; 24(47): 141-52.
507796	Georgia National Research on Domestic Violence Against Women 2009	Georgia	ACT Research (Georgia), Ivane Javakhishvili Tbilisi State University (Georgia). Georgia National Research on Domestic Violence Against Women 2009.
529525	Gabon Demographic and Health Survey 2019-2021	Gabon	General Directorate of Statistics (Gabon), ICF International, Ministry of Economy, Employment and Sustainable Development (Gabon), Ministry of Health and Social Affairs (Gabon), Ministry of Higher Education and Scientific Research (Gabon). Gabon Demographic and Health Survey 2019-2021. Fairfax, United States of America: ICF International, 2023.
540292	Gabon National Survey on Gender-Based Violence 2016	Gabon	Ministry of Health and Social Affairs (Gabon), United Nations Population Fund (UNFPA). Gabon National Survey on Gender-Based Violence 2016.
21188	Ghana Demographic and Health Survey 2008	Ghana	Ghana Statistical Service, Macro International, Inc, Ministry of Health (Ghana). Ghana Demographic and Health Survey 2008. Fairfax, United States of America: ICF International.
150478	Germany Health, Well-Being and Personal Safety of Women 2003	Germany	Bielefeld University, Federal Ministry of Family Affairs, Senior Citizens, Women and Youth (Germany), Institute for Applied Social Sciences (INFAS). Germany Health, Well-Being and Personal Safety of Women 2003.
507907	Greece Domestic Violence Against Women First Nationwide Epidemiological Survey 2002-2003	Greece	Research Centre for Gender Equality (??TH?) (Greece). Greece Domestic Violence Against Women First Nationwide Epidemiological Survey 2002-2003.
527726	Ghana Demographic and Health Survey 2022-2023	Ghana	Ghana Statistical Service, ICF International. Ghana Demographic and Health Survey 2022-2023. 2024.

4779	Guatemala Reproductive Health Survey 2008-2009	Guatemala	Guatemala Ministry of Health and Social Assistance, University of Valle and Division of Reproductive Health-Centers for Disease Control and Prevention (CDC). Guatemala Reproductive Health Survey 2008-2009. Atlanta, United States: Centers for Disease Control and Prevention (CDC).
157031	Guatemala Demographic and Health Survey 2014-2015	Guatemala	ICF International, Institute of Nutrition of Central America and Panama, Ministry of Public Health and Social Assistance (Guatemala), National Statistics Institute (Guatemala), Secretary of Planning and Programming of the Presidency (Segeplán) (Guatemala). Guatemala Demographic and Health Survey 2014-2015. Fairfax, United States of America: ICF International, 2017.
27563	Guatemala Reproductive Health Survey 2002	Guatemala	Guatemala Ministry of Health and Social Assistance, University of Valle, Division of Reproductive Health-Centers for Disease Control and Prevention (CDC). (2003) Guatemala Reproductive Health Survey 2002. Atlanta, United States: Centers for Disease Control and Prevention (CDC).
474478	Grenada Women's Health and Life Experiences Survey 2018	Grenada	Caribbean Development Bank, Central Statistical Office (Grenada), Global Women?s Institute, George Washington University, United Nations Entity for Gender Equality and the Empowerment of Women (UN Women). Grenada Women's Health and Life Experiences Survey 2018.
507051	Guinea National Survey on Gender Based Violence 2016	Guinea	Ministry of Social Action, Women's Advancement and Childhood (Guinea). Guinea National Survey on Gender Based Violence 2016.
19720	Haiti Demographic and Health Survey 2005-2006	Haiti	Haitian Institute of Childhood (IHE), Haitian Institute of Statistics and Informatics, Macro International, Inc. Haiti Demographic and Health Survey 2005-2006. Fairfax, United States of America: ICF International.
408933	Honduras Violence Against Children, Girls and Adolescents Survey 2017	Honduras	Centers for Disease Control and Prevention (CDC), International Organization for Migration (IOM), Ministry of Security (Honduras), Together for Girls, United Nations Children's Fund (UNICEF). Honduras Violence Against Children, Girls and Adolescents Survey 2017.
19728	Honduras Demographic and Health Survey 2005-2006	Honduras	Macro International, Inc, National Institute of Statistics (Honduras), Secretary of Health (Honduras). Honduras Demographic and Health Survey 2005-2006. Fairfax, United States of America: ICF International.
65118	Haiti Demographic and Health Survey 2012	Haiti	Centers for Disease Control and Prevention (CDC), Haitian Institute of Childhood (IHE), Haitian Institute of Statistics and Informatics, Macro International, Inc. Haiti Demographic

			and Health Survey 2012. Fairfax, United States of America: ICF International.
95440	Honduras Demographic and Health Survey 2011-2012	Honduras	ICF Macro, National Institute of Statistics (Honduras). Honduras Demographic and Health Survey 2011-2012. Fairfax, United States of America: ICF International.
27551	Honduras Reproductive Health Survey 2001	Honduras	Honduras Family Planning Association (ASHONPLAFA), Ministry of Health (Honduras), and Division of Reproductive Health-Centers for Disease Control and Prevention (CDC). Honduras Reproductive Health Survey 2001. Tegucigalpa, Honduras: Honduras Family Planning Association (ASHONPLAFA).
231763	Haiti Violence Against Children Survey 2012	Haiti	Centers for Disease Control and Prevention (CDC), Government of Haiti, Interuniversity Institute for Research and Development (INURED), Multi-Sector Coordination Committee (CCMHAITI) (Haiti), Together for Girls. Haiti Violence Against Children Survey 2012. Washington, DC, United States of America: Together for Girls.
19708	Haiti Demographic and Health Survey 2000	Haiti	Haitian Institute of Childhood (IHE), Macro International, Inc. Haiti Demographic and Health Survey 2000. Fairfax, United States of America: ICF International.
27215	Guinea-Bissau Multiple Indicator Cluster Survey 2010	Guinea-Bissau	Centers for Disease Control and Prevention (CDC), National Statistics Institute (Guinea-Bissau), United Nations Children's Fund (UNICEF). Guinea-Bissau Multiple Indicator Cluster Survey 2010. New York, United States of America: United Nations Children's Fund (UNICEF), 2018.
4949	Haiti Living Condition Survey 2001	Haiti	Haitian Institute of Statistics and Informatics, The Fafo Research Foundation. Haiti Living Condition Survey 2001, Haiti: Haitian Institute of Statistics and Informatics.
137192	Health status and health care use of Massachusetts women reporting partner abuse	United States of America	Hathaway JE, Mucci LA, Silverman JG, Brooks DR, Mathews R, Pavlos CA. Health status and health care use of Massachusetts women reporting partner abuse. Am J Prev Med. 2000; 19(4): 302-7.
218574	Haiti Demographic and Health Survey 2016-2017	Haiti	Haitian Institute of Childhood (IHE), Haitian Institute of Statistics and Informatics, ICF International, Ministry of Public Health and Population (Haiti). Haiti Demographic and Health Survey 2016-2017. Fairfax, United States of America: ICF International.
474276	Guyana Women's Health and Life Experiences Survey 2018	Guyana	Bureau of Statistics (Guyana), Global Women?s Institute, George Washington University, United Nations Entity for Gender Equality and the Empowerment of Women (UN Women). Guyana Women's Health and Life Experiences Survey 2018.

490966	Honduras Multiple Indicator Cluster Survey 2019	Honduras	European Union (EU), Government of Canada, Government of Honduras, National Institute of Statistics (Honduras), Secretary of Health (Honduras), United Nations Children's Fund (UNICEF), United Nations Population Fund (UNFPA). Honduras Multiple Indicator Cluster Survey 2019. New York, United States of America: United Nations Children's Fund (UNICEF), 2021.
169728	Hungary Gender, Alcohol and Culture: An International Study (GENACIS) 2001	Hungary	Aarhus University, Addiction Switzerland Research Institute, Alcohol Research Group, Public Health Institute, Centre for Addiction and Mental Health (Canada), Centre for Alcohol Policy Research, Turning Point Alcohol and Drug Centre (Australia), Kettil Bruun Society for Social and Epidemiological Research on Alcohol, University of North Dakota. Hungary Gender, Alcohol and Culture: An International Study (GENACIS) 2001.
150606	Human rights abuses and concerns about women's health and human rights in southern Iraq	Iraq	Amowitz LL, Kim G, Reis C, Asher JL, Iacopino V. Human rights abuses and concerns about women's health and human rights in southern Iraq. JAMA. 2004; 291(12): 1471-9.
508294	Iceland Men's Violence Against Women Study 2008-2010	Iceland	Ministry of Social Affairs and Insurance (Iceland). Iceland Men's Violence Against Women Study 2008-2010.
19963	India Demographic and Health Survey 2005-2006	India	International Institute for Population Sciences (India), Macro International, Inc. India Demographic and Health Survey 2005-2006. Fairfax, United States of America: ICF International.
157050	India Demographic and Health Survey 2015-2016	India	ICF International, International Institute for Population Sciences (India), Ministry of Health and Family Welfare (India). India Demographic and Health Survey 2015-2016. Fairfax, United States of America: ICF International, 2018.
19950	India Demographic and Health Survey 1998-1999	India	International Institute for Population Sciences (India), Macro International, Inc. India Demographic and Health Survey 1998-1999. Calverton, United States of America: Macro International, Inc.
169730	India - Karnataka Gender, Alcohol and Culture: An International Study (GENACIS) 2003	India	Aarhus University, Addiction Switzerland Research Institute, Alcohol Research Group, Public Health Institute, Centre for Addiction and Mental Health (Canada), Centre for Alcohol Policy Research, Turning Point Alcohol and Drug Centre (Australia), Kettil Bruun Society for Social and Epidemiological Research on Alcohol, University of North Dakota. India - Karnataka Gender, Alcohol and Culture: An International Study (GENACIS) 2003.

228081	India - Goa Alcohol Use Study 2004-2008	India	Alcohol Research Group, Public Health Institute, Sangath. India - Goa Alcohol Use Study 2004-2008.
501406	India - Bihar and Uttar Pradesh Understanding the Lives of Adolescents and Young Adults 2015-2016, Wave 1	India	Population Council (India). India - Bihar and Uttar Pradesh Understanding the Lives of Adolescents and Young Adults 2015-2016, Wave 1. New Delhi, India: Population Council (India), 2017.
501530	India - Bihar and Uttar Pradesh Understanding the Lives of Adolescents and Young Adults 2018-2019, Wave 2	India	Population Council (India). India - Bihar and Uttar Pradesh Understanding the Lives of Adolescents and Young Adults 2018-2019, Wave 2. New Delhi, India: Population Council (India), 2020.
506335	Indonesia - Papua and West Papua Study on Women's and Men's Life Experiences 2016	Indonesia	Rifka Annisa Women's Crisis Center (Indonesia), Statistics Indonesia, United Nations Development Programme (UNDP), United States Agency for International Development (USAID). Indonesia - Papua and West Papua Study on Women's and Men's Life Experiences 2016.
512518	India Masculinity, Intimate Partner Violence and Son Preference 2014	India	International Center for Research on Women, United Nations Population Fund (UNFPA). India Masculinity, Intimate Partner Violence and Son Preference 2014.
137189	Intimate partner violence and health outcomes in mid-life women: a population-based cohort study	Australia	Schei B, Guthrie JR, Dennerstein L, Alford S. Intimate partner violence and health outcomes in mid-life women: a population-based cohort study. Arch Womens Ment Health. 2006; 9(6): 317-24.
150747	International Violence Against Women Surveys Data 2002-2005	Australia; China; Costa Rica; Czechia; Denmark; Mozambique; Philippines; Poland; Switzerland	European Institute for Crime Prevention and Control, affiliated with the United Nations (HEUNI), United Nations Office on Drugs and Crime (UNODC), Statistics Canada, United Nations Interregional Crime and Justice Research Institute (UNICRI). International Violence Against Women Surveys (IVAWS) Data 2002-2005. As provided by the Global Burden of Disease Child Sexual Abuse and Intimate Partner Violence Expert Group. [Unpublished].
270601	Intimate partner violence and sexual health outcomes: a population-based study among 16-44-year-old women in Estonia	Estonia	Laanpere M, Ringmets I, Part K, Karro H. Intimate partner violence and sexual health outcomes: a population-based study among 16-44-year-old women in Estonia. Eur J Public Health. 2013; 23(4): 688-93.
128662	Intimate partner violence against women in the capital province of Sri Lanka: prevalence, risk factors, and help seeking	Sri Lanka	Jayasuriya V, Wijewardena K, Axemo P. Intimate partner violence against women in the capital province of Sri Lanka: prevalence, risk factors, and help seeking. Violence Against Women. 2011; 17(8): 1086-102.
137261	Intimate partner violence and alcohol consumption	Brazil	Zaleski M, Pinsky I, Laranjeira R, Ramisetty-Mikler S, Caetano R. Intimate partner violence and alcohol consumption. Rev Saude Publica. 2010; 44(1): 53-9.

150454	Intimate partner abuse factors associated with women?s health: a general population study	Iceland	Svavarsdottir EK, Orlygsdottir B. Intimate partner abuse factors associated with women?s health: a general population study. J Adv Nurs. 2009; 65(7): 1452-62.
150537	Intimate Partner Violence in Spain: Findings from a National Survey	Spain	Medina-Ariza J, Barberet R. Intimate Partner Violence in Spain: Findings from a National Survey. Violence Against Women. 2003; 9(3): 302-22.
163087	Intimate partner violence, relationship power inequity, and incidence of HIV infection in young women in South Africa: a cohort study	South Africa	Jewkes RK, Dunkle K, Nduna M, Shai N. Intimate partner violence, relationship power inequity, and incidence of HIV infection in young women in South Africa: a cohort study. Lancet. 2010; 376(9734): 41?8.
425294	Intimate partner violence and incidence of depression in married women: A longitudinal study of a nationally representative sample	Republic of Korea	Han KM, Jee HJ, An H, Shin C, Yoon HK, Ko YH, Ham BJ, Kim YK, Han C. Intimate partner violence and incidence of depression in married women: A longitudinal study of a nationally representative sample. J Affect Disord. 2019; 245: 305-311.
499625	Integrated Survey of Social Conditions in Iraq for a Year and Women's Health in 2021	Iraq	Central Statistical Organization (Iraq), Kurdistan Regional Statistics Office, Ministry of Health (Iraq), United Nations Population Fund (UNFPA). Integrated Survey of Social Conditions in Iraq for a Year and Women's Health in 2021.
505730	Intimate partner violence in southwestern Nigeria: are there rural-urban differences?	Nigeria	Balogun MO, Owoaje ET, Fawole OI. Intimate partner violence in southwestern Nigeria: are there rural-urban differences?. Women Health. 2012; 52(7): 627-45.
505932	Intimate Partner Violence among Women of Child Bearing Age in Alimosho LGA of Lagos State, Nigeria	Nigeria	Adegbite OB, Ajuwon AJ. Intimate Partner Violence among Women of Child Bearing Age in Alimosho LGA of Lagos State, Nigeria. Afr J Biomed Res. 2015; 18(2): 135-46.
540294	Indonesia Violence Against Women Survey 2016	Indonesia	United Nations Population Fund (UNFPA). Indonesia Violence Against Women Survey 2016 .
151535	Iraq Women Integrated Social and Health Survey 2011	Iraq	Central Statistical Organization (Iraq), Kurdistan Regional Statistics Office, Ministry of Health (Iraq), United Nations Population Fund (UNFPA). Iraq Women Integrated Social and Health Survey 2011.
273012	Ireland Sexual Abuse and Violence Study 2001	Ireland	Department of Health and Children (Ireland), Department of Justice, Equality and Law Reform (Ireland), Dublin Rape Crisis Center, Royal College of Surgeons in Ireland (RCSI). Ireland Sexual Abuse and Violence Study 2001.
23429	Iraq Family Health Survey 2006-2007	Iraq	Ministry of Health (Iraq), Central Organization for Statistics and Information Technology (Iraq), Kurdistan Regional Statistics Office, World Health Organization (WHO), Ministry of Health (Kurdistan). Iraq Family Health Survey 2006-2007.

137219	Ireland National Study of Domestic Abuse 2003	Ireland	Economic and Social Research Institute (ESRI) (Ireland), National Crime Council (Ireland). Ireland National Study of Domestic Abuse 2003.
137269	Is Elder Abuse and Neglect a Social Phenomenon? Data from the First National Prevalence Survey in Israel	Israel	Lowenstein A, Eisikovits Z, Band-Winterstein T, Enosh G. Is Elder Abuse and Neglect a Social Phenomenon? Data from the First National Prevalence Survey in Israel. J Elder Abuse Negl. 2009; 21(3): 253-77.
150620	Ireland Making the Links Study 1995	Ireland	Economic and Social Research Institute (ESRI) (Ireland), Women's Aid (Ireland). Ireland Making the Links Study 1995.
7163	Jamaica Reproductive Health Survey 2008-2009	Jamaica	Jamaica Family Planning Board, Jamaica Division of Reproductive Health-Centers for Disease Control and Prevention (CDC). Jamaica Reproductive Health Survey 2008. Atlanta, United States: Centers for Disease Control and Prevention (CDC).
169735	Japan Gender, Alcohol and Culture: An International Study (GENACIS) 2001	Japan	Aarhus University, Addiction Switzerland Research Institute, Alcohol Research Group, Public Health Institute, Centre for Addiction and Mental Health (Canada), Centre for Alcohol Policy Research, Turning Point Alcohol and Drug Centre (Australia), Kettil Bruun Society for Social and Epidemiological Research on Alcohol, University of North Dakota. Japan Gender, Alcohol and Culture: An International Study (GENACIS) 2001.
415455	Jamaica Women?s Health Survey 2016	Jamaica	Global Women?s Institute, George Washington University, Statistical Institute of Jamaica. Jamaica Women?s Health Survey 2016. Inter-American Development Bank (IDB), 2018.
7161	Jamaica Reproductive Health Survey 2002-2003	Jamaica	Division of Reproductive Health, Centers for Disease Control and Prevention (CDC), Jamaica National Family Planning Board, Statistical Institute of Jamaica. Jamaica Reproductive Health Survey 2002-2003. Kingston, Jamaica: Derek Gordon Databank, University of the West Indies.
437818	Jamaica HIV/AIDS Knowledge, Attitudes, Behavior, and Practices Survey 2012	Jamaica	Hope Enterprises Ltd, Ministry of Health (Jamaica). Jamaica HIV/AIDS Knowledge, Attitudes, Behavior, and Practices Survey 2012.
506358	Italy Violence Against Women Survey 2014	Italy	National Institute of Statistics (Italy). Italy Violence Against Women Survey 2014. Rome, Italy: National Institute of Statistics (Italy).
506367	Italy Violence Against Women Survey 2006	Italy	Department for Equal Opportunities (Italy), National Institute of Statistics (Italy). Italy Violence Against Women Survey 2006.
557684	Jamaica Reproductive Health Survey 2021-2022	Jamaica	Jamaica National Family Planning Board, Statistical Institute of Jamaica. Jamaica Reproductive Health Survey 2021-2022. 2023.

20083	Jordan Demographic and Health Survey 2007	Jordan	Department of Statistics (Jordan), Macro International, Inc. Jordan Demographic and Health Survey 2007. Fairfax, United States of America: ICF International.
76702	Kazakhstan Multiple Indicator Cluster Survey 2010-2011	Kazakhstan	Agency of the Republic of Kazakhstan on Statistics, United Nations Children's Fund (UNICEF). Kazakhstan Multiple Indicator Cluster Survey 2010-2011. New York, United States of America: United Nations Children's Fund (UNICEF), 2013.
77517	Jordan Demographic and Health Survey 2012	Jordan	Department of Statistics (Jordan), ICF International. Jordan Demographic and Health Survey 2012. Fairfax, United States of America: ICF International.
169736	Kazakhstan Gender, Alcohol and Culture: An International Study (GENACIS) 2002-2003	Kazakhstan	Aarhus University, Addiction Switzerland Research Institute, Alcohol Research Group, Public Health Institute, Centre for Addiction and Mental Health (Canada), Centre for Alcohol Policy Research, Turning Point Alcohol and Drug Centre (Australia), Kettil Bruun Society for Social and Epidemiological Research on Alcohol, University of North Dakota. Kazakhstan Gender, Alcohol and Culture: An International Study (GENACIS) 2002-2003.
356955	Jordan Demographic and Health Survey 2017-2018	Jordan	Department of Statistics (Jordan), ICF International. Jordan Demographic and Health Survey 2017-2018. Fairfax, United States of America: ICF International, 2019.
508296	Japan Violence Between Men and Women Survey 2008	Japan	Gender Equality Cabinet Bureau Office (Japan). Japan Violence Between Men and Women Survey 2008.
508297	Japan Violence Between Men and Women Survey 2005	Japan	Gender Equality Cabinet Bureau Office (Japan). Japan Violence Between Men and Women Survey 2005.
508298	Japan Violence Between Men and Women Survey 2002	Japan	Gender Equality Cabinet Bureau Office (Japan). Japan Violence Between Men and Women Survey 2002.
508299	Japan Violence Between Men and Women Survey 1999	Japan	Gender Equality Cabinet Bureau Office (Japan). Japan Violence Between Men and Women Survey 1999.
540297	Kazakhstan Sample Survey on Violence Against Women 2015	Kazakhstan	Ministry of National Economy (Kazakhstan). Kazakhstan Sample Survey on Violence Against Women 2015.
126420	Kenya Violence Against Children Study 2010	Kenya	Centers for Disease Control and Prevention (CDC), Kenya National Bureau of Statistics, United Nations Children's Fund (UNICEF). Kenya Violence Against Children Study 2010. Nairobi, Kenya: Kenya National Bureau of Statistics.
454583	Kenya Violence Against Children Study 2018-2019	Kenya	Centers for Disease Control and Prevention (CDC), Kenya National Bureau of Statistics, Ministry of Labour and Social Protection (Kenya), Population Council, Together for Girls, United Nations Children's Fund

			(UNICEF). Kenya Violence Against Children Study 2018-2019. Washington, DC, United States of America: Together for Girls.
21365	Kenya Demographic and Health Survey 2008-2009	Kenya	ICF Macro, Kenya Medical Research Institute (KEMRI), Kenya National Bureau of Statistics, Ministry of Public Health and Sanitation (Kenya), National AIDS and STI Control Programme (NASCOP) (Kenya), National Association of County and City Health Officials (NACCHO) (United States), National Coordinating Agency for Population and Development (Kenya). Kenya Demographic and Health Survey 2008-2009. Fairfax, United States of America: ICF International.
39427	Kenya - Coast and Rift Valley Behavioral Surveillance Survey 2007	Kenya	Family Health International, Federation of Kenya Employers, International Center for Reproductive Health (Kenya), Matatu Welfare Association. Kenya - Coast and Rift Valley Behavioral Surveillance Survey 2007. Durham, North Carolina: Family Health International.
157057	Kenya Demographic and Health Survey 2014	Kenya	ICF International, Kenya Medical Research Institute (KEMRI), Kenya National Bureau of Statistics, Ministry of Health (Kenya), National AIDS Control Council (Kenya), National Council for Population and Development (Kenya). Kenya Demographic and Health Survey 2014. Fairfax, United States of America: ICF International.
20145	Kenya Demographic and Health Survey 2003	Kenya	Centers for Disease Control and Prevention (CDC), Central Bureau of Statistics (Kenya), Macro International, Inc, Ministry of Health (Kenya), National Council for Population and Development (Kenya). Kenya Demographic and Health Survey 2003. Fairfax, United States of America: ICF International.
515657	Kenya Performance Monitoring and Action Phase 3 Household and Female Survey 2021-2022	Kenya	Bill and Melinda Gates Institute for Population and Reproductive Health, Johns Hopkins Bloomberg School of Public Health, International Center for Reproductive Health (Kenya), Jhpiego. Kenya Performance Monitoring and Action Phase 3 Household and Female Survey 2021-2022. Baltimore, United States of America: Johns Hopkins Research Data Repository, 2024.
515915	Kenya Nairobi Performance Monitoring and Action Agile Youth Respondent Driven Sampling Survey Round 3 2021	Kenya	Bill and Melinda Gates Institute for Population and Reproductive Health, Johns Hopkins Bloomberg School of Public Health, International Center for Reproductive Health (Kenya). Kenya Nairobi Performance Monitoring and Action Agile Youth Respondent Driven Sampling Survey Round 3 2021. Baltimore, United States of America: Johns Hopkins Bloomberg School of Public Health, 2021.

515791	Kenya Nairobi Performance Monitoring and Action Agile Youth Respondent Driven Sampling Survey Round 1 2019	Kenya	Bill and Melinda Gates Institute for Population and Reproductive Health, Johns Hopkins Bloomberg School of Public Health, International Center for Reproductive Health (Kenya). Kenya Nairobi Performance Monitoring and Action Agile Youth Respondent Driven Sampling Survey Round 1 2019. Baltimore, United States of America: Johns Hopkins Bloomberg School of Public Health, 2021.
529017	Kenya Demographic and Health Survey 2022	Kenya	ICF International, Kenya National Bureau of Statistics, Ministry of Health (Kenya). Kenya Demographic and Health Survey 2022. Fairfax, United States of America: ICF International, 2023.
126428	Kiribati Family Health and Support Study 2008	Kiribati	Kiribati National Statistics Office, Ministry of Internal and Social Affairs (Kiribati), Secretariat of the Pacific Community (SPC), United Nations Children's Fund (UNICEF), World Health Organization (WHO). Kiribati Family Health and Support Study 2008.
77518	Kyrgyzstan Demographic and Health Survey 2012	Kyrgyzstan	ICF International, Ministry of Health (Kyrgyzstan), National Statistical Committee of the Kyrgyz Republic. Kyrgyzstan Demographic and Health Survey 2012. Fairfax, United States of America: ICF International.
438014	Kiribati Multiple Indicator Cluster Survey 2018-2019	Kiribati	Kiribati National Statistics Office, Ministry of Health and Medical Services (Kiribati), Secretariat of the Pacific Community (SPC), United Nations Children's Fund (UNICEF), United Nations Population Fund (UNFPA). Kiribati Multiple Indicator Cluster Survey 2018-2019. New York, United States of America: United Nations Children's Fund (UNICEF), 2020.
20191	Liberia Demographic and Health Survey 2006-2007	Liberia	Liberia Institute for Statistics and Geo-information Services (LISGIS), Macro International, Inc. Liberia Demographic and Health Survey 2006-2007. Fairfax, United States of America: ICF International.
338550	Lesotho Gender Based Violence Indicators Study 2013	Lesotho	Bureau of Statistics (Lesotho), Gender Links (South Africa). Lesotho Gender Based Violence Indicators Study 2013.
465218	Lesotho Violence Against Children and Youth Survey 2018	Lesotho	Centers for Disease Control and Prevention (CDC), ICAP, Columbia University Mailman School of Public Health, Ministry of Social Development (Lesotho), Together for Girls. Lesotho Violence Against Children and Youth Survey 2018. Lesotho: Ministry of Social Development (Lesotho).
459845	Liberia Demographic and Health Survey 2019-2020	Liberia	ICF International, Liberia Institute for Statistics and Geo-information Services (LISGIS), Ministry of Health and Social

			Welfare (Liberia). Liberia Demographic and Health Survey 2019-2020. 2021.
327582	Lesotho Population-Based HIV Impact Assessment 2016-2017	Lesotho	Bureau of Statistics (Lesotho), Centers for Disease Control and Prevention (CDC), ICAP, Columbia University Mailman School of Public Health, Ministry of Health (Lesotho). Lesotho Population-Based HIV Impact Assessment 2016-2017. New York, New York: ICAP, Columbia University Mailman School of Public Health, 2021.
391388	Laos National Survey on Women?s Health and Life Experiences 2013-2014	Lao People's Democratic Republic	Lao Statistics Bureau, Lao Women's Union, Ministry of Health (Laos), Ministry of Justice (Laos), Ministry of Public Security (Laos), National Commission for the Advancement of Women (NCAW) (Laos), United Nations Entity for Gender Equality and the Empowerment of Women (UN Women), United Nations Population Fund (UNFPA), World Health Organization (WHO). Laos National Survey on Women?s Health and Life Experiences 2013-2014.
137285	Lifetime interpersonal violence and self-reported chlamydia trachomatis diagnosis among California women	United States of America	Alvarez J, Pavao J, Mack KP, Chow JM, Baumrind N, Kimerling R. Lifetime interpersonal violence and self-reported chlamydia trachomatis diagnosis among California women. J Womens Health (Larchmt). 2009; 18(1): 57-63.
508301	Lithuania Domestic Violence Against Women and Assessment of the Status of Victims of Domestic Violence 2008	Lithuania	BGI Consulting (Lithuania), Ministry of Social Security and Labor (Lithuania). Lithuania Domestic Violence Against Women and Assessment of the Status of Victims of Domestic Violence 2008.
21393	Malawi Demographic and Health Survey 2010	Malawi	ICF Macro, National Statistical Office of Malawi. Malawi Demographic and Health Survey 2010. Fairfax, United States of America: ICF International.
218581	Malawi Demographic and Health Survey 2015-2016	Malawi	Emory University and Centers for Disease Control & Prevention Collaboration, ICF International, Ministry of Health (Malawi), National Statistical Office of Malawi. Malawi Demographic and Health Survey 2015-2016. Fairfax, United States of America: ICF International, 2017.
231758	Malawi Violence Against Children and Young Women Survey 2013	Malawi	Center for Social Research, University of Malawi, Centers for Disease Control and Prevention (CDC), Ministry of Gender, Children, Disability and Social Welfare (Malawi), President's Emergency Plan for AIDS Relief (PEPFAR), Together for Girls, United Nations Children's Fund (UNICEF). Malawi Violence Against Children and Young Women Survey 2013. Washington, DC, United States of America: Together for Girls.

20263	Malawi Demographic and Health Survey 2004-2005	Malawi	Macro International, Inc, National Statistical Office of Malawi. Malawi Demographic and Health Survey 2004-2005. Fairfax, United States of America: ICF International.
287629	Malawi Population-Based HIV Impact Assessment 2015-2016	Malawi	Centers for Disease Control and Prevention (CDC), ICAP, Columbia University Mailman School of Public Health, Johns Hopkins University, Ministry of Health (Malawi), Statistical Center for HIV/AIDS Research and Prevention (SCHARP), University of Malawi College of Medicine, Westat. Malawi Population-Based HIV Impact Assessment 2015-2016.
150619	Malawi National Gender-Based Violence Study 2005	Malawi	National Statistical Office of Malawi. Malawi National Gender-Based Violence Study 2005.
399853	Madagascar Multiple Indicator Cluster Survey 2018	Madagascar	National Institute of Statistics (Madagascar), United Nations Children's Fund (UNICEF). Madagascar Multiple Indicator Cluster Survey 2018. 2019.
550704	Malaysia National Health and Morbidity Survey 2022	Malaysia	Department of Statistics (Malaysia), Institute for Public Health, Ministry of Health (Malaysia). Malaysia National Health and Morbidity Survey 2022.
218582	Maldives Demographic and Health Survey 2016-2017	Maldives	ICF International, Ministry of Health (Maldives). Maldives Demographic and Health Survey 2016-2017. Fairfax, United States of America: ICF International, 2019.
398033	Mali Demographic and Health Survey 2018	Mali	ICF International, National Institute of Statistics (INSTAT) (Mali), Sector Planning and Statistics Unit Health-Social Development and Family Promotion (CPS / SS-DS-PF) (Mali). Mali Demographic and Health Survey 2018. Fairfax, United States of America: ICF International, 2019.
20274	Mali Demographic and Health Survey 2006	Mali	Macro International, Inc, Ministry of Health (Mali), National Directorate of Statistics and Informatics (DNSI) (Mali). Mali Demographic and Health Survey 2006. Fairfax, United States of America: ICF International.
77388	Mali Demographic and Health Survey 2012-2013	Mali	ICF International, INFO-STAT (Mali), Ministry of Health (Mali), National Institute of Statistics (INSTAT) (Mali), Planning and Statistics Unit, Ministry of Health (Mali). Mali Demographic and Health Survey 2012-2013. Fairfax, United States of America: ICF International, 2014.
150604	Marital Violence Among Korean Elderly Couples: A Cultural Residue	Republic of Korea	Kim JY, Sung K. Marital Violence Among Korean Elderly Couples: A Cultural Residue. J Elder Abuse Negl. 2001; 13(4): 73-89.
500889	Marshall Islands Family Health and Safety Study 2012	Marshall Islands	Ministry of Internal Affairs (Marshall Islands), Women United Together Marshall Islands (WUTMI). Marshall Islands Family Health and Safety Study 2012.

506383	Maldives Study on Women?s Health and Life Experiences 2006	Maldives	Ministry of Gender, Family and Social Services (Maldives). Maldives Study on Women?s Health and Life Experiences 2006.
506512	Malta Nationwide Research Study on the Prevalence of Domestic Violence Against Women 2010	Malta	Commission on Gender-Based Violence and Domestic Violence (Malta), M. Fsadni & Associates (Malta). Malta Nationwide Research Study on the Prevalence of Domestic Violence Against Women 2010.
124873	Male responsibility and maternal morbidity: a cross-sectional study in two Nigerian states	Nigeria	Andersson N, Omer K, Caldwell D, Dambam MM, Maikudi AY, Effiong B, Ikpi E, Udofia E, Khan A, Ansari U, Ansari N, Hamel C. Male responsibility and maternal morbidity: a cross-sectional study in two Nigerian states. BMC Health Serv Res. 2011; S7.
550624	Malta Survey on Safety and Well-Being 2022	Malta	Eurostat, National Statistics Office (Malta). Malta Survey on Safety and Well-Being 2022. Lascaris, Valletta, Malta: National Statistics Office (Malta).
338551	Mauritius Gender Based Violence Indicators Study 2010-2011	Mauritius	Central Statistics Office (Mauritius), Gender Links (South Africa). Mauritius Gender Based Violence Indicators Study 2010-2011.
137151	Men's and women's childhood sexual abuse and victimization in adult partner relationships: A study of risk factors	Canada	Daigneault I, Hébert M, McDuff P. Men's and women's childhood sexual abuse and victimization in adult partner relationships: A study of risk factors. Child Abuse Negl. 2009; 33(9): 638-47.
105291	Mexico National Addiction Survey 2011	Mexico	National Center for the Prevention and Control of Addictions (Mexico), National Council Against Addictions (Mexico), National Institute of Psychiatry Ramón de la Fuente Muñiz (Mexico), National Institute of Public Health (Mexico). Mexico National Addiction Survey 2011.
238502	Men?s and women?s exposure and perpetration of partner violence: an epidemiological study from Sweden	Sweden	Lövestad S, Krantz G. Men?s and women?s exposure and perpetration of partner violence: an epidemiological study from Sweden. BMC Public Health. 2012; 12: 945.
470667	Mauritania Demographic and Health Survey 2019-2020	Mauritania	National Office of Statistics (Mauritania). Mauritania Demographic and Health Survey 2019-2020. Fairfax, United States of America: ICF International, 2022.
188837	Mexico National Survey on Violence Against Women 2002-2003	Mexico	Institute for Social Security and Services for State Workers (ISSSTE) (Mexico), Mexican Social Security Institute (IMSS), National Institute of Public Health (Mexico), Secretariat of Health (Mexico). Mexico National Survey on Violence Against Women 2002-2003.
429109	Mexico National Survey of Health and Nutrition (ENSANUT) 2018-2019 100K	Mexico	Ministry of Health (Mexico), National Institute of Public Health (Mexico). Mexico National Survey of Health and Nutrition (ENSANUT) 2018-2019 100K. Cuernavaca, Mexico: National Institute of Public Health (Mexico).

20339	Moldova Demographic and Health Survey 2005	Republic of Moldova	Macro International, Inc, National Scientific and Applied Center for Preventive Medicine (Moldova). Moldova Demographic and Health Survey 2005. Fairfax, United States of America: ICF International.
432257	Mongolia Gender Based Violence Survey 2017	Mongolia	National Statistical Office of Mongolia. Mongolia Gender Based Violence Survey 2017. Ulaanbaatar, Mongolia: National Statistical Office of Mongolia, 2018.
151334	Mexico National Survey on the Dynamics of Household Relationships 2003	Mexico	National Institute of Statistics and Geography (INEGI) (Mexico), National Institute of Women (Mexico), United Nations Women's Fund (UNIFEM). Mexico National Survey on the Dynamics of Household Relationships 2003. Mexico City, Mexico: National Institute of Statistics and Geography (INEGI) (Mexico).
151335	Mexico National Survey on the Dynamics of Household Relationships 2006	Mexico	National Institute of Statistics and Geography (INEGI) (Mexico), National Institute of Women (Mexico), Special Prosecutor for Violence Related Crimes Against Women in the Country (FEVIM), Attonery General's Office (Mexico), United Nations Women's Fund (UNIFEM). Mexico National Survey on the Dynamics of Household Relationships 2006. Mexico City, Mexico: National Institute of Statistics and Geography (INEGI) (Mexico).
151336	Mexico National Survey on the Dynamics of Household Relationships 2011	Mexico	National Commission for the Development of Indigenous Peoples (Mexico), National Institute of Statistics and Geography (INEGI) (Mexico), National Institute of Women (Mexico), United Nations Women's Fund (UNIFEM). Mexico National Survey on the Dynamics of Household Relationships 2011. Mexico City, Mexico: National Institute of Statistics and Geography (INEGI) (Mexico).
317752	Mexico National Survey on the Dynamics of Household Relationships 2016	Mexico	National Institute of Statistics and Geography (INEGI) (Mexico), National Institute of Women (Mexico), United Nations Women's Fund (UNIFEM). Mexico National Survey on the Dynamics of Household Relationships 2016. Mexico City, Mexico: National Institute of Statistics and Geography (INEGI) (Mexico).
8750	Moldova Reproductive Health Survey 1997	Republic of Moldova	Division of Reproductive Health-Centers for Disease Control and Prevention (CDC) and Moldova Ministry of Health. (1998) Moldova Reproductive Health Survey 1997. Atlanta, United States: Centers for Disease Control and Prevention (CDC).
81748	Mexico National Survey of Health and Nutrition 2011-2012	Mexico	National Institute of Public Health (Mexico). Mexico National Survey of Health and Nutrition 2011-2012. Cuernavaca, Mexico: National Institute of Public Health (Mexico).

474222	Moldova OSCE-Led Survey on the Well-Being and Safety of Women 2018	Republic of Moldova	IMAS (Moldova), Organization for Security and Co-operation in Europe (OSCE). Moldova OSCE-Led Survey on the Well-Being and Safety of Women 2018. 2019.
506504	Moldova Violence Against Women in the Family 2010	Republic of Moldova	National Office of Statistics (Moldova), United Nations Development Programme (UNDP), United Nations Entity for Gender Equality and the Empowerment of Women (UN Women), United Nations Population Fund (UNFPA). Moldova Violence Against Women in the Family 2010.
553181	Mexico National Survey on the Dynamics of Household Relationships 2021	Mexico	National Institute of Statistics and Geography (INEGI) (Mexico), National Institute of Women (Mexico), United Nations Women's Fund (UNIFEM). Mexico National Survey on the Dynamics of Household Relationships 2021. Mexico City, Mexico: National Institute of Statistics and Geography (INEGI) (Mexico).
157061	Myanmar Demographic and Health Survey 2015-2016	Myanmar	ICF International, Ministry of Health and Sports (Myanmar). Myanmar Demographic and Health Survey 2015-2016. Fairfax, United States of America: ICF International, 2017.
55975	Mozambique Demographic and Health Survey 2011	Mozambique	ICF Macro, Manhica Health Research Center (CISM), Ministry of Health (Mozambique), National Institute of Statistics (INE) (Mozambique). Mozambique Demographic and Health Survey 2011. Fairfax, United States of America: ICF International.
157060	Mozambique AIDS Indicator Survey 2015	Mozambique	Centers for Disease Control and Prevention (CDC), ICF International, Ministry of Health (Mozambique), National Institute of Health (Mozambique), National Institute of Statistics (INE) (Mozambique). Mozambique AIDS Indicator Survey 2015. Fairfax, United States of America: ICF International, 2018.
27519	Mozambique Young Adult Reproductive Health Survey 2001	Mozambique	Mozambique National Institute of Statistics, Division of Reproductive Health-Centers for Disease Control and Prevention (CDC). (2003) Mozambique Young Adult Reproductive Health Survey 2001. Atlanta, United States: Centers for Disease Control and Prevention (CDC).
423654	Morocco National Survey on Population and Family Health 2017-2018	Morocco	Ministry of Health (Morocco), Pan Arab Project for Family Health (PAPFAM), United Nations Children's Fund (UNICEF), United Nations Population Fund (UNFPA), World Health Organization (WHO). Morocco National Survey on Population and Family Health 2017-2018.
474225	Montenegro OSCE-Led Survey on the Well-Being and Safety of Women 2018	Montenegro	Ipsos, Organization for Security and Co-operation in Europe (OSCE). Montenegro OSCE-Led Survey on the Well-Being and Safety of Women 2018. 2019.

507058	Morocco National Survey on the Prevalence of Violence Against Women 2009-2010	Morocco	High Commission for Planning (Morocco). Morocco National Survey on the Prevalence of Violence Against Women 2009-2010.
508380	Morocco National Survey on Violence Against Women and Men 2019	Morocco	High Commission for Planning (Morocco). Morocco National Survey on Violence Against Women and Men 2019.
553572	Mozambique Demographic and Health Survey 2022-2023	Mozambique	ICF International, National Institute of Statistics (INE) (Mozambique). Mozambique Demographic and Health Survey 2022-2023. Fairfax, United States of America: ICF International, 2024.
286782	Nepal Demographic and Health Survey 2016-2017	Nepal	ICF International, Ministry of Health (Nepal), New ERA. Nepal Demographic and Health Survey 2016-2017. Fairfax, United States of America: ICF International, 2017.
359089	Namibia Population-Based HIV Impact Assessment 2017	Namibia	Centers for Disease Control and Prevention (CDC), ICAP, Columbia University Mailman School of Public Health, Ministry of Health and Social Services (Namibia), Namibia Institute of Pathology, Namibia Statistics Agency, University of California, San Francisco. Namibia Population-Based HIV Impact Assessment 2017. New York, New York: ICAP, Columbia University Mailman School of Public Health, 2021.
21240	Nepal Demographic and Health Survey 2011	Nepal	ICF Macro, Ministry of Health and Population (Nepal), New ERA. Nepal Demographic and Health Survey 2011. Fairfax, United States of America: ICF International.
150382	Namibia Demographic and Health Survey 2013	Namibia	ICF International, Ministry of Health and Social Services (Namibia), Namibia Institute of Pathology, Namibia Statistics Agency. Namibia Demographic and Health Survey 2013. Fairfax, United States of America: ICF International.
452911	National Survey on Social Relations: Main Results ENARES 2013 and 2015	Peru	National Institute of Statistics and Informatics (Peru). National Survey on Social Relations: Main Results ENARES 2013 and 2015 . Lima, Peru: National Institute of Statistics and Informatics (Peru).
472783	Nauru Family Health and Support Study 2013	Nauru	Department of Women's Affairs (Nauru), Ministry of Home Affairs (Nauru). Nauru Family Health and Support Study 2013. Nauru: Ministry of Home Affairs (Nauru).
506334	National Study on Violence Against Women in Georgia 2017	Georgia	European Union (EU), National Statistics Office of Georgia (GeoStat), United Nations Entity for Gender Equality and the Empowerment of Women (UN Women). National Study on Violence Against Women in Georgia 2017. 2018.
528571	Nepal Demographic and Health Survey 2022	Nepal	ICF International, Ministry of Health and Population (Nepal), New ERA. Nepal Demographic and Health Survey 2022.

			Fairfax, United States of America: ICF International, 2023.
554207	National Study on Violence Against Women in Georgia 2022	Georgia	European Union (EU), National Statistics Office of Georgia (GeoStat), United Nations Entity for Gender Equality and the Empowerment of Women (UN Women). National Study on Violence Against Women in Georgia 2022. 2023.
169739	New Zealand Gender, Alcohol and Culture: An International Study (GENACIS) 2007	New Zealand	Aarhus University, Addiction Switzerland Research Institute, Alcohol Research Group, Public Health Institute, Centre for Addiction and Mental Health (Canada), Centre for Alcohol Policy Research, Turning Point Alcohol and Drug Centre (Australia), Kettil Bruun Society for Social and Epidemiological Research on Alcohol, University of North Dakota. New Zealand Gender, Alcohol and Culture: An International Study (GENACIS) 2007.
141635	New Zealand Youth2012 Health and Wellbeing of Secondary School Students 2012	New Zealand	Adolescent Health Research Group, University of Auckland, Auckland UniServices, University of Auckland (New Zealand), Faculty of Medical and Health Sciences, University of Auckland. New Zealand Youth2012 Health and Wellbeing of Secondary School Students 2012.
454488	New Zealand Youth 2019 Rangatahi Smart Survey 2019	New Zealand	University of Auckland (New Zealand), University of Otago (New Zealand), Victoria University of Wellington (New Zealand). New Zealand Youth 2019 Rangatahi Smart Survey 2019.
9270	Nicaragua Reproductive Health Survey 2006-2007	Nicaragua	Division of Reproductive Health, Centers for Disease Control and Prevention (CDC), National Institute for Development Information (Nicaragua). Nicaragua Reproductive Health Survey 2006-2007. Managua, Nicaragua: National Institute for Development Information (Nicaragua).
150424	New Zealand Youth2000 Health and Wellbeing of Secondary School Students 2001	New Zealand	Adolescent Health Research Group, University of Auckland, Faculty of Medical and Health Sciences, University of Auckland. New Zealand Youth2000 Health and Wellbeing of Secondary School Students 2001.
169738	Nicaragua Gender, Alcohol and Culture: An International Study (GENACIS) 2005	Nicaragua	Aarhus University, Addiction Switzerland Research Institute, Alcohol Research Group, Public Health Institute, Centre for Addiction and Mental Health (Canada), Centre for Alcohol Policy Research, Turning Point Alcohol and Drug Centre (Australia), Kettil Bruun Society for Social and Epidemiological Research on Alcohol, University of North Dakota. Nicaragua Gender, Alcohol and Culture: An International Study (GENACIS) 2005.

150525	New Zealand WHO Multi-country Study on Women's Health and Domestic Violence Against Women 2003	New Zealand	School of Population Health, University of Auckland, World Health Organization (WHO). New Zealand WHO Multi-country Study on Women's Health and Domestic Violence Against Women 2003.
20478	Nicaragua Demographic and Health Survey 1997-1998	Nicaragua	Macro International, Inc, Ministry of Health (Nicaragua), National Institute of Statistics and Censuses (Nicaragua). Nicaragua Demographic and Health Survey 1997-1998. Fairfax, United States of America: ICF International.
126952	Nicaragua National Demographic and Health Survey 2011-2012	Nicaragua	Ministry of Health (Nicaragua), National Institute for Development Information (Nicaragua). Nicaragua National Demographic and Health Survey 2011-2012. Managua, Nicaragua: National Institute for Development Information (Nicaragua).
516219	Niger Performance Monitoring and Action Phase 2 Household and Female Survey 2022	Niger	Bill and Melinda Gates Institute for Population and Reproductive Health, Johns Hopkins Bloomberg School of Public Health, Jhpiego, National Institute of Statistics (Niger). Niger Performance Monitoring and Action Phase 2 Household and Female Survey 2022. Baltimore, United States of America: Johns Hopkins Bloomberg School of Public Health, 2022.
21433	Nigeria Demographic and Health Survey 2008	Nigeria	Macro International, Inc, National Population Commission of Nigeria. Nigeria Demographic and Health Survey 2008. Fairfax, United States of America: ICF International, 2009.
231760	Nigeria Violence Against Children Survey 2014	Nigeria	Centers for Disease Control and Prevention (CDC), Government of Nigeria, Together for Girls, United Nations Children's Fund (UNICEF). Nigeria Violence Against Children Survey 2014.
408484	Nigeria Demographic and Health Survey 2018	Nigeria	Federal Ministry of Health (Nigeria), ICF International, National Population Commission (NPC). Nigeria Demographic and Health Survey 2018. Fairfax, United States of America: ICF International, 2020.
77390	Nigeria Demographic and Health Survey 2013	Nigeria	ICF International, National Population Commission of Nigeria. Nigeria Demographic and Health Survey 2013. Fairfax, United States of America: ICF International.
336233	Norway Domestic Violence Survey 2003-2004	Norway	Norwegian Institute for Urban and Regional Research (NIBR), Norwegian University of Science and Technology (NTNU), Statistics Norway. Norway Domestic Violence Survey 2003-2004.
169740	Nigeria Gender, Alcohol and Culture: An International Study (GENACIS) 2003	Nigeria	Aarhus University, Addiction Switzerland Research Institute, Alcohol Research Group, Public Health Institute, Centre for Addiction and Mental Health (Canada), Centre for Alcohol Policy Research, Turning Point Alcohol and Drug Centre (Australia), Kettil

			Bruun Society for Social and Epidemiological Research on Alcohol, University of North Dakota. Nigeria Gender, Alcohol and Culture: An International Study (GENACIS) 2003.
354770	Norway Safety, Violence and Quality of Life Survey 2013	Norway	Norwegian Centre for Violence and Traumatic Stress Studies (NKVTS). Norway Safety, Violence and Quality of Life Survey 2013.
449727	Norway Violence and Rape: A National Prevalence Study of Violence in a Lifespan Perspective 2013	Norway	Ipsos MMI (Norway), Norwegian Centre for Violence and Traumatic Stress Studies (NKVTS). Norway Violence and Rape: A National Prevalence Study of Violence in a Lifespan Perspective 2013.
512760	Nigeria International Men and Gender Equality Study 2015	Nigeria	Promundo. Nigeria International Men and Gender Equality Study 2015.
518895	Nigeria - Lagos Performance Monitoring and Action Agile Youth Respondent Driven Sampling Survey Round 1 2020	Nigeria	Bill and Melinda Gates Institute for Population and Reproductive Health, Johns Hopkins Bloomberg School of Public Health, University of Ibadan (Nigeria). Nigeria - Lagos Performance Monitoring and Action Agile Youth Respondent Driven Sampling Survey Round 1 2020. Baltimore, United States of America: Johns Hopkins Bloomberg School of Public Health, 2021.
9975	Palestine Domestic Violence Survey 2005-2006	Palestine	Palestinian Central Bureau of Statistics. Palestine Domestic Violence Survey 2005-2006.
286783	Pakistan Demographic and Health Survey 2017-2018	Pakistan	ICF International, Ministry of National Health Services, Regulations & Coordination (Pakistan), National Institute of Population Studies (Pakistan). Pakistan Demographic and Health Survey 2017-2018. Fairfax, United States of America: ICF International, 2018.
77521	Pakistan Demographic and Health Survey 2012-2013	Pakistan	ICF International, National Institute of Population Studies (Pakistan), Pakistan Bureau of Statistics. Pakistan Demographic and Health Survey 2012-2013. Fairfax, United States of America: ICF International.
151539	Palestine - Wife Abuse and Battering In the West Bank and Gaza: Results of Two National Surveys	Palestine	Haj-Yahia MM, Bisan Center for Research and Development. Palestine - Wife Abuse and Battering In the West Bank and Gaza: Results of Two National Surveys. Ramallah, Palestine: Bisan Center for Research and Development, 1999.
500888	Palau Family Health and Safety Study 2013	Palau	Ministry of Health (Palau). Palau Family Health and Safety Study 2013.
510667	Palestine International Men and Gender Equality Study 2016-2017	Palestine	Institute of Women's Studies, Birzeit University (Palestine), Promundo, United Nations Entity for Gender Equality and the Empowerment of Women (UN Women). Palestine International Men and Gender Equality Study 2016-2017.

512974	Pakistan International Men and Gender Equality Study 2017	Pakistan	International Center for Research on Women, Ministry of Human Rights (Pakistan), Population Council, Promundo, Rozan (Pakistan), Rutgers (Netherlands). Pakistan International Men and Gender Equality Study 2017.
24826	Pakistan Status of Women, Reproductive Health, and Family Planning Survey 2003	Pakistan	Federal Bureau of Statistics (Pakistan), National Institute of Population Studies (Pakistan). Pakistan Status of Women, Reproductive Health, and Family Planning Survey 2003.
426238	Papua New Guinea Demographic and Health Survey 2016-2018	Papua New Guinea	ICF International, National Statistical Office (Papua New Guinea). Papua New Guinea Demographic and Health Survey 2016-2018. Fairfax, United States of America: ICF International, 2019.
137256	Palestine Violence Survey 2011	Palestine	Palestinian Central Bureau of Statistics, United Nations Children's Fund (UNICEF), United Nations Development Programme, Programme of Assistance to the Palestinian People (UNDP/PAPP), United Nations Population Fund (UNFPA). Palestine Violence Survey 2011.
10370	Paraguay Reproductive Health Survey 2004	Paraguay	Division of Reproductive Health-Centers for Disease Control and Prevention (CDC). (2005): Paraguay Reproductive Health Survey 2004. Asunción, Paraguay, Paraguayan Center for Population Studies (CEPEP).
27525	Paraguay Reproductive Health Survey 2008	Paraguay	Paraguay Center for Population Studies (CEPEP). Paraguay Reproductive Health Survey 2008. Asunción, Paraguay: Paraguayan Center for Population Studies (CEPEP).
10326	Paraguay Contraceptive Prevalence Survey 1998	Paraguay	Division of Reproductive Health-Centers for Disease Control and Prevention (CDC). Paraguay Contraceptive Prevalence Survey 1998. Atlanta, United States: Centers for Disease Control and Prevention (CDC).
10364	Paraguay Reproductive Health Survey 1995-1996	Paraguay	Division of Reproductive Health-Centers for Disease Control and Prevention (CDC). Paraguay Reproductive Health Survey 1995-1996. Asunción, Paraguay, Paraguayan Center for Population Studies (CEPEP).
137274	Partner violence and health: results from the first national study on violence against women in Norway	Norway	Neroien AI, Schei B. Partner violence and health: results from the first national study on violence against women in Norway. Scand J Public Health. 2008; 36(2): 161-8.
417528	Panama Sexual and Reproductive Health Survey 2014-2015	Panama	Gorgas Memorial Institute for Health Studies, Ministry of Health (Panama), National Institute of Statistics and Census (Panama), United Nations Population Fund (UNFPA). Panama Sexual and Reproductive Health Survey 2014-2015.

506588	Panama Sexual and Reproductive Health Survey 2009	Panama	Gorgas Memorial Institute for Health Studies. Panama Sexual and Reproductive Health Survey 2009. 2011.
531275	Palestine Violence Survey 2019	Palestine	Palestinian Central Bureau of Statistics, Violence Survey in the Palestinian Society, 2019 Version 1.0 (2022). Ramallah - Palestine.
539932	Papua New Guinea - Western Highlands and West Sepik Family Wellbeing Survey 2014	Papua New Guinea	Australian Government Department of Foreign Affairs and Trade (DFAT), FHI 360. Papua New Guinea - Western Highlands and West Sepik Family Wellbeing Survey 2014.
358824	Peru Demographic and Family Health Survey 2017	Peru	National Center for Food and Nutrition, National Institute of Health (Peru), National Institute of Statistics and Informatics (Peru), National Police of Peru (PNP). Peru Demographic and Family Health Survey 2017. Lima, Peru: National Institute of Statistics and Informatics (Peru).
270404	Peru Continuous Demographic and Health Survey 2009	Peru	National Institute of Statistics and Informatics (Peru), ORC Macro. Peru Continuous Demographic and Health Survey 2009. Fairfax, United States of America: ICF International.
270469	Peru Continuous Demographic and Health Survey 2010	Peru	National Institute of Statistics and Informatics (Peru). Peru Continuous Demographic and Health Survey 2010. Fairfax, United States of America: ICF International.
270470	Peru Continuous Demographic and Health Survey 2011	Peru	Macro International, Inc, National Institute of Statistics and Informatics (Peru). Peru Continuous Demographic and Health Survey 2011. Fairfax, United States of America: ICF International.
270471	Peru Continuous Demographic and Health Survey 2012	Peru	Macro International, Inc, National Institute of Statistics and Informatics (Peru). Peru Continuous Demographic and Health Survey 2012. Fairfax, United States of America: ICF International.
275090	Peru Continuous Demographic and Health Survey 2003-2008	Peru	Ministry of Economy and Finance (Peru), National Institute of Statistics and Informatics (Peru), ORC Macro. Peru Continuous Demographic and Health Survey 2003-2008. Fairfax, United States of America: ICF International.
169742	Peru - Lima and Ayacucho Gender, Alcohol and Culture: An International Study (GENACIS) 2005	Peru	Aarhus University, Addiction Switzerland Research Institute, Alcohol Research Group, Public Health Institute, Centre for Addiction and Mental Health (Canada), Centre for Alcohol Policy Research, Turning Point Alcohol and Drug Centre (Australia), Kettil Bruun Society for Social and Epidemiological Research on Alcohol, University of North Dakota. Peru - Lima and Ayacucho Gender, Alcohol and Culture: An International Study (GENACIS) 2005.

210231	Peru Continuous Demographic and Health Survey 2013 - INEI	Peru	ICF International, National Institute of Statistics and Informatics (Peru). Peru Continuous Demographic and Health Survey 2013 - INEI. Lima, Peru: National Institute of Statistics and Informatics (Peru), 2014.
303663	Peru Demographic and Family Health Survey 2015	Peru	National Institute of Statistics and Informatics (Peru). Peru Demographic and Family Health Survey 2015. Lima, Peru: National Institute of Statistics and Informatics (Peru), 2017.
303664	Peru Demographic and Family Health Survey 2016	Peru	National Institute of Statistics and Informatics (Peru). Peru Demographic and Family Health Survey 2016. Lima, Peru: National Institute of Statistics and Informatics (Peru), 2017.
210182	Peru Continuous Demographic and Health Survey 2014 - INEI	Peru	ICF International, Ministry of Health (Peru), National Institute of Statistics and Informatics (Peru), National Police of Peru (PNP). Peru Continuous Demographic and Health Survey 2014 - INEI. Lima, Peru: National Institute of Statistics and Informatics (Peru), 2015.
150518	Peru WHO Multi-country Study on Women's Health and Domestic Violence Against Women 2000	Peru	Cayetano Heredia University, Flora Tristan Center of Peruvian Women, World Health Organization (WHO). Peru WHO Multi-country Study on Women's Health and Domestic Violence Against Women 2000.
20649	Peru Demographic and Health Survey 2000	Peru	Macro International, Inc, National Institute of Statistics (Peru). Peru Demographic and Health Survey 2000. Fairfax, United States of America: ICF International.
407869	Peru Demographic and Family Health Survey 2018	Peru	National Institute of Statistics and Informatics (Peru). Peru Demographic and Family Health Survey 2018. Lima, Peru: National Institute of Statistics and Informatics (Peru), 2019.
452894	Peru Demographic and Family Health Survey 2019	Peru	National Institute of Statistics and Informatics (Peru). Peru Demographic and Family Health Survey 2019. Lima, Peru: National Institute of Statistics and Informatics (Peru).
511269	Peru Demographic and Family Health Survey 2020	Peru	National Institute of Statistics and Informatics (Peru). Peru Demographic and Family Health Survey 2020. Lima, Peru: National Institute of Statistics and Informatics (Peru), 2021.
511759	Peru Demographic and Family Health Survey 2021	Peru	National Institute of Statistics and Informatics (Peru). Peru Demographic and Family Health Survey 2021. Lima, Peru: National Institute of Statistics and Informatics (Peru), 2022.
539146	Peru Demographic and Family Health Survey 2022	Peru	National Institute of Statistics and Informatics (Peru). Peru Demographic and Family Health Survey 2022. Digital Government Secretariat, Presidency of the Council of Ministers (Peru), 2023.
142943	Philippines Demographic and Health Survey 2013	Philippines	ICF International, Philippines Statistics Authority. Philippines Demographic and Health Survey 2013. Fairfax, United States of America: ICF International, 2014.
21421	Philippines Demographic and Health Survey 2008	Philippines	Macro International, Inc, National Statistics Office (Philippines). Philippines Demographic

			and Health Survey 2008. Fairfax, United States of America: ICF International, 2010.
337877	Philippines Demographic and Health Survey 2017	Philippines	ICF International, Philippines Statistics Authority, United States Agency for International Development (USAID). Philippines Demographic and Health Survey 2017. Fairfax, United States of America: ICF International, 2018.
137263	Physical and sexual abuse of women before, during, and after pregnancy	China	Guo SF, Wu JL, Qu CY, Yan RY. Physical and sexual abuse of women before, during, and after pregnancy. Int J Gynaecol Obstet. 2004; 84(3): 281-6.
150538	Physical and mental health effects of intimate partner violence for men and women	United States of America	Coker AL, Davis KE, Arias I, Desai S, Sanderson M, Brandt HM, Smith PH. Physical and mental health effects of intimate partner violence for men and women. Am J Prev Med. 2002; 23(4): 260-8.
150618	Physical intimate partner violence in Chile, Egypt, India and the Philippines	Chile; Egypt; India; Philippines	Hassan F, Sadowski LS, Bangdiwala SI, Vizcarra B, Ramiro L, De Paula CS, Bordin IA, Mitra MK. Physical intimate partner violence in Chile, Egypt, India and the Philippines. Inj Control Saf Promot. 2004; 11(2): 111-6.
521982	Philippines Young Adult Fertility and Sexuality Study 2012-2013	Philippines	Demographic Research and Development Foundation Inc. (Philippines), University of the Philippines Population Institute (UPPI). Philippines Young Adult Fertility and Sexuality Study 2012-2013. Manila, Philippines: University of the Philippines, Manila, 2016.
527435	Philippines Demographic and Health Survey 2022	Philippines	ICF International, Philippine Statistics Authority, United States Agency for International Development (USAID). Philippines Demographic and Health Survey 2022. Fairfax, United States of America: ICF International, 2023.
150611	Prevalence of emotional, physical and sexual abuse of women in three South African provinces	South Africa	Jewkes R, Penn-Kekana L, Levin J, Ratsaka M, Schrieber M. Prevalence of emotional, physical and sexual abuse of women in three South African provinces. S Afr Med J. 2001; 91(5): 421-8.
126440	Prevalence and characteristics of sexual violence in the Netherlands, the risk of revictimization and pregnancy: results from a national population survey	Netherlands	De Haas S, van Berlo W, Bakker F, Vanwesenbeeck I. Prevalence and characteristics of sexual violence in the Netherlands, the risk of revictimization and pregnancy: results from a national population survey. Violence Vict. 2012; 27(4): 592-608.
14486	Puerto Rico Reproductive Health Survey 1995-1996	United States of America	University of Puerto Rico. Puerto Rico Reproductive Health Survey 1995-1996. San Juan, Puerto Rico: University of Puerto Rico, 1998.
137273	Psychotropic drug use among women exposed to intimate partner violence: A population-based study	Norway	Stene LE, Dyb G, Jacobsen GW, Schei B. Psychotropic drug use among women exposed to intimate partner violence: A population-

			based study. Scand J Public Health. 2010; 38(5 Suppl): 88-95.
137286	Prevalence of wife rape and other intimate partner sexual coercion in a nationally representative sample of women	United States of America	Basile KC. Prevalence of wife rape and other intimate partner sexual coercion in a nationally representative sample of women. Violence Vict. 2002; 17(5): 511-24.
150547	Prevalence, antecedent causes and consequences of domestic violence in Myanmar	Myanmar	Kyu N, Kanai A. Prevalence, antecedent causes and consequences of domestic violence in Myanmar. Asian J Soc Psychol. 2005; 8(3): 244-71.
150549	Prevalence of Wife Abuse in the Netherlands: Combining Quantitative and Qualitative Methods in Survey Research	Netherlands	Romkens R. Prevalence of Wife Abuse in the Netherlands: Combining Quantitative and Qualitative Methods in Survey Research. J Interpers Violence. 1997; 12(1): 99-125.
222877	Prevalence and Factors Associated with Intimate Partner Violence Among Married Women in an Urban Community in Lagos State, Nigeria	Nigeria	Onigbogi MO, Odeyemi KA, Onigbogi OO. Prevalence and Factors Associated with Intimate Partner Violence Among Married Women in an Urban Community in Lagos State, Nigeria. Afr J Reprod Health. 2015; 19(1): 91-100.
505724	Preconceptional health: risks of adverse pregnancy outcomes by reproductive life stage in the Central Pennsylvania Women's Health Study (CePAWHS)	United States of America	Weisman CS, Hillemeier MM, Chase GA, Dyer AM, Baker SA, Feinberg M, Symons Downs D, Parrott RL, Cecil HK, Botti JJ, MacNeill C, Chuang CH, Yost B. Preconceptional health: risks of adverse pregnancy outcomes by reproductive life stage in the Central Pennsylvania Women's Health Study (CePAWHS). Womens Health Issues. 2006; 16(4): 216-24.
530940	Prevalence of interpersonal violence against women and men in New Zealand: results of a cross-sectional study	New Zealand	Fanslow JL, Malihi Z, Hashemi L, Gulliver P, McIntosh T. Prevalence of interpersonal violence against women and men in New Zealand: results of a cross-sectional study. Aust N Z J Public Health. 2022; 46(2): 117-126.
454625	Republic of Moldova Violence Against Children and Youth Survey 2018-2019	Republic of Moldova	Centers for Disease Control and Prevention (CDC), International Organization for Migration (IOM), Ministry of Health (Moldova). Republic of Moldova Violence Against Children and Youth Survey 2018-2019. Washington, DC, United States of America: Together for Girls.
162535	Russia Reproductive Health Survey 2011	Russian Federation	Centers for Disease Control and Prevention (CDC), Federal State Statistics Service (Russia), Ministry of Health (Russian Federation). Russia Reproductive Health Survey 2011.
243047	Republic of Moldova Progress Report on HIV/AIDS 2008-2009	Republic of Moldova	Joint United Nations Program on HIV/AIDS (UNAIDS), Ministry of Health (Moldova). Republic of Moldova Progress Report on HIV/AIDS 2008-2009. Republic of Moldova: Ministry of Health (Moldova), 2010.

11128	Romania Reproductive Health Survey 1999	Romania	Division of Reproductive Health-Centers for Disease Control and Prevention (CDC) and Romanian Association of Public Health and Health Management. (2001) Romania Reproductive Health Survey 1999. Atlanta, United States: Centers for Disease Control and Prevention (CDC).
139160	Romania Reproductive Health Survey 2004	Romania	Alfred Rusescu Institute for Mother and Child Care, John Snow, Inc., Ministry of Health (Romania), Swiss Agency for Development and Cooperation, United Nations Children's Fund (UNICEF), United Nations Population Fund (UNFPA), United States Agency for International Development (USAID), World Health Organization (WHO). Romania Reproductive Health Survey 2004.
128661	Racial Differences in the Prevalence of Intimate Partner Violence Against Women and Associated Factors	United States of America	Cho H. Racial Differences in the Prevalence of Intimate Partner Violence Against Women and Associated Factors. J Interpers Violence. 2012; 27(2): 344-63.
137262	Risk factors for domestic physical violence: national cross-sectional household surveys in eight southern African countries	Botswana; Lesotho; Malawi; Mozambique; Namibia; Eswatini; Zambia; Zimbabwe	Andersson N, Ho-Foster A, Mitchell S, Scheepers E, Goldstein S. Risk factors for domestic physical violence: national cross-sectional household surveys in eight southern African countries. BMC Womens Health. 2007; 7: 11.
137278	Rape against Women: The Magnitude, Perpetrators and Patterns of Disclosure of Events in Dar es Salaam, Tanzania	United Republic of Tanzania	Muganyizi PS, Kilewo C, Moshiro C. Rape against Women: The Magnitude, Perpetrators and Patterns of Disclosure of Events in Dar es Salaam, Tanzania. Afr J Reprod Health. 2004; 8(3): 137-46.
151542	Russia Reproductive Health Survey 1999	Russian Federation	Division of Reproductive Health, Centers for Disease Control and Prevention (CDC), Russian Public Opinion Research Center. Russia Reproductive Health Survey 1999.
511672	Romania Reproductive Health Survey 2016	Romania	Alfred Rusescu Institute for Mother and Child Care. Romania Reproductive Health Survey 2016.
20740	Rwanda Demographic and Health Survey 2005	Rwanda	Macro International, Inc, National Institute of Statistics of Rwanda. Rwanda Demographic and Health Survey 2005. Fairfax, United States of America: ICF International.
26866	Sao Tome and Principe Demographic and Health Survey 2008-2009	Sao Tome and Principe	ICF Macro, Ministry of Health (Sao Tome and Principe), National Institute of Statistics (Sao Tome and Principe). Sao Tome and Principe Demographic and Health Survey 2008-2009. Fairfax, United States of America: ICF International.
157063	Rwanda Demographic and Health Survey 2014-2015	Rwanda	ICF International, Ministry of Health (Rwanda), National Institute of Statistics of Rwanda. Rwanda Demographic and Health Survey 2014-2015. Fairfax, United States of America: ICF International, 2016.

126427	Samoa Family Health and Safety Study 2000	Samoa	Ministry of Women Affairs (Samoa), Secretariat of the Pacific Community (SPC). Samoa Family Health and Safety Study 2000.
56040	Rwanda Demographic and Health Survey 2010-2011	Rwanda	ICF Macro, Ministry of Health (Rwanda), National Institute of Statistics of Rwanda. Rwanda Demographic and Health Survey 2010-2011. Fairfax, United States of America: ICF International.
462482	Rwanda Demographic and Health Survey 2019-2020	Rwanda	Ministry of Health (Rwanda), National Institute of Statistics (Rwanda), Rwanda Biomedical Center (RBC). Rwanda Demographic and Health Survey 2019-2020. Fairfax, United States of America: ICF International, 2021.
353526	Senegal Continuous Demographic and Health Survey 2017	Senegal	ICF International, Ministry of Health and Social Action (Senegal), National Agency of Statistics and Demography (Senegal), Unit for the Fight Against Malnutrition (Senegal). Senegal Continuous Demographic and Health Survey 2017. Fairfax, United States of America: ICF International, 2018.
238503	Self-reported exposure to intimate partner violence among women and men in Sweden: results from a population-based survey	Sweden	Nybergh L, Taft C, Enander V, Krantz G. Self-reported exposure to intimate partner violence among women and men in Sweden: results from a population-based survey. BMC Public Health. 2013; 13: 845.
460813	Senegal Continuous Demographic and Health Survey 2019	Senegal	ICF International, Ministry of Health and Social Action (Senegal), National Agency of Statistics and Demography (Senegal), United States Agency for International Development (USAID). Senegal Continuous Demographic and Health Survey 2019. Fairfax, United States of America: ICF International, 2020.
450419	Senegal Continuous Demographic and Health Survey 2018	Senegal	Directorate of Forecasting and Statistics, Ministry of the Economy, Finance and Planning (Senegal), ICF International, Ministry of Health and Social Action (Senegal), United States Agency for International Development (USAID). Senegal Continuous Demographic and Health Survey 2018. Fairfax, United States of America: ICF International, 2020.
474226	Serbia OSCE-Led Survey on the Well-Being and Safety of Women 2018	Serbia	Ipsos, Organization for Security and Co-operation in Europe (OSCE). Serbia OSCE-Led Survey on the Well-Being and Safety of Women 2018. 2019.
513143	Serbia International Men and and Gender Equality Survey 2017	Serbia	CARE International, Centar E8, Ministry of Youth and Sport (Serbia), Oak Foundation, Promundo, United Nations Population Fund (UNFPA). Serbia International Men and and Gender Equality Survey 2017.
131467	Sierra Leone Demographic and Health Survey 2013	Sierra Leone	ICF International, Ministry of Health and Sanitation (Sierra Leone), Statistics Sierra Leone. Sierra Leone Demographic and Health

			Survey 2013. Fairfax, United States of America: ICF International, 2014.
425283	Sierra Leone Demographic and Health Survey 2019	Sierra Leone	ICF International, Ministry of Health and Sanitation (Sierra Leone), Statistics Sierra Leone. Sierra Leone Demographic and Health Survey 2019. Fairfax, United States of America: ICF International, 2020.
506505	Singapore International Violence Against Women Survey 2009	Singapore	Nielsen Company. Singapore International Violence Against Women Survey 2009. 2013.
508068	Seychelles National Survey on Domestic Violence 2006	Seychelles	Gender Secretariat (Seychelles). Seychelles National Survey on Domestic Violence 2006.
508069	Slovakia Representative Research on Prevalence and Experience of Women with Violence Against Women 2008	Slovakia	Ministry of Labor, Social Affairs and Family (Slovakia). Slovakia Representative Research on Prevalence and Experience of Women with Violence Against Women 2008.
157064	South Africa Demographic and Health Survey 2016	South Africa	Department of Health (South Africa), ICF International, South African Medical Research Council, Statistics South Africa. South Africa Demographic and Health Survey 2016. Fairfax, United States of America: ICF International, 2019.
20796	South Africa Demographic and Health Survey 1998	South Africa	Department of Health (South Africa), Macro International, Inc, South African Medical Research Council. South Africa Demographic and Health Survey 1998. Fairfax, United States of America: ICF International.
454381	Somalia Health and Demographic Survey 2018-2019	Somalia	Directorate of National Statistics (Somalia), Federal Government of Somalia, United Nations Population Fund (UNFPA). Somalia Health and Demographic Survey 2018-2019.
12102	South Africa HIV/AIDS Behavioral Risks, Sero-Status, and Mass Media Impact Survey 2002	South Africa	Center for AIDS Development, Research and Evaluation (CADRE) (South Africa), Department of Social Development (South Africa), Family Health International, Geospace International (South Africa), Human Sciences Research Council, Joint United Nations Program on HIV/AIDS (UNAIDS), Medical University of Southern Africa (MEDUNSA), National Agency for AIDS Research (ANRS) (France), National Health Laboratory Service (NHLS) (South Africa), South African Medical Research Council, University of Natal, Wits Health Consortium. South Africa HIV/AIDS Behavioral Risks, Sero-Status, and Mass Media Impact Survey 2002. Pretoria, South Africa: Human Sciences Research Council, 2011.
344441	South Africa Gender Based Violence Indicators Study 2010-2013	South Africa	Gender Links (South Africa). South Africa Gender Based Violence Indicators Study 2010-2013.

313076	South Africa National HIV Prevalence, Incidence, and Behavior Survey 2011-2012	South Africa	Centers for Disease Control and Prevention (CDC), Global Clinical and Viral Laboratory (South Africa), Human Sciences Research Council, National Institute for Communicable Diseases (South Africa), South African Medical Research Council, University of Cape Town. South Africa National HIV Prevalence, Incidence, and Behavior Survey 2011-2012. Pretoria, South Africa: Human Sciences Research Council, 2016.
126426	Solomon Islands Family Health and Safety Study 2008	Solomon Islands	Solomon Islands National Statistics Office (SINSO). Solomon Islands Family Health and Safety Study 2008.
137275	Socioeconomic resources, gender traditionalism, and wife abuse in urban Russian couples	Russian Federation	Cubbins LA, Vannoy D. Socioeconomic resources, gender traditionalism, and wife abuse in urban Russian couples. J Marriage Fam. 2005; 67(1): 37-52.
169743	Spain Gender, Alcohol and Culture: An International Study (GENACIS) 2003	Spain	Aarhus University, Addiction Switzerland Research Institute, Alcohol Research Group, Public Health Institute, Centre for Addiction and Mental Health (Canada), Centre for Alcohol Policy Research, Turning Point Alcohol and Drug Centre (Australia), Kettil Bruun Society for Social and Epidemiological Research on Alcohol, University of North Dakota. Spain Gender, Alcohol and Culture: An International Study (GENACIS) 2003.
305577	South Korea National Survey on Domestic Violence 2013	Republic of Korea	Korean Women's Development Institute, Ministry of Gender Equality and Family (South Korea). South Korea National Survey on Domestic Violence 2013.
305583	South Korea National Survey on Domestic Violence 2016	Republic of Korea	Korean Women's Development Institute, Ministry of Gender Equality and Family (South Korea). South Korea National Survey on Domestic Violence 2016.
300508	South Korea National Survey on Domestic Violence 2007	Republic of Korea	Korea Family Law Counseling Center, Korea Institute for Health and Social Affairs (KIHASA), Korean Criminal Policy Institute. South Korea National Survey on Domestic Violence 2007.
507099	Spain Gender Violence Macro Survey 2010-2011	Spain	Government Delegation for Gender Violence (Spain), Ministry of Health, Social Services and Equality (Spain), Sociological Research Center (Spain). Spain Gender Violence Macro Survey 2010-2011.
169744	Sri Lanka Gender, Alcohol and Culture: An International Study (GENACIS) 2002	Sri Lanka	Aarhus University, Addiction Switzerland Research Institute, Alcohol Research Group, Public Health Institute, Centre for Addiction and Mental Health (Canada), Centre for Alcohol Policy Research, Turning Point Alcohol and Drug Centre (Australia), Kettil Bruun Society for Social and Epidemiological Research on Alcohol, University of North Dakota. Sri Lanka Gender, Alcohol and

			Culture: An International Study (GENACIS) 2002.
326837	Sri Lanka Demographic and Health Survey 2016	Sri Lanka	Department of Census and Statistics (Sri Lanka), ICF International. Sri Lanka Demographic and Health Survey 2016.
474283	Suriname National Women's Health Survey 2018	Suriname	General Bureau of Statistics (Suriname), Global Women?s Institute, George Washington University, Government of the Republic of Suriname, Inter-American Development Bank (IDB), Ministry of Health (Suriname), QURE (Trinidad and Tobago). Suriname National Women's Health Survey 2018.
503525	Sri Lanka United Nations Multi-country Study on Men and Violence 2012-2013 - Equimundo	Sri Lanka	CARE International, Partners for Prevention, United Nations Development Programme (UNDP), United Nations Entity for Gender Equality and the Empowerment of Women (UN Women), United Nations Population Fund (UNFPA), United Nations Volunteers (UNV). Sri Lanka United Nations Multi-country Study on Men and Violence 2012-2013 - Equimundo.
507100	Spain Violence Against Women Macro Survey 2014	Spain	Government Delegation for Gender Violence (Spain), Ministry of Health, Social Services and Equality (Spain), Sociological Research Center (Spain). Spain Violence Against Women Macro Survey 2014.
508070	Sri Lanka Women?s Wellbeing Survey 2019	Sri Lanka	Department of Census and Statistics (Sri Lanka). Sri Lanka Women?s Wellbeing Survey 2019.
539964	Spain Violence Against Women Macro Survey 2019	Spain	Government Delegation for Gender Violence (Spain), Ministry of Health, Social Services and Equality (Spain), Sociological Research Center (Spain). Spain Violence Against Women Macro Survey 2019.
137221	Sweden Prevalence Study on Men´s Violence Against Women in ""Equal"" Sweden 1999-2000	Sweden	Statistics Sweden, Uppsala University. Sweden Prevalence Study on Men´s Violence Against Women in ""Equal"" Sweden 1999-2000.
225621	Sweden National Prevalence Study on Exposure to Violence 2012	Sweden	National Board of Health and Welfare (Sweden), National Center for Knowledge on Men's Violence Against Women, Uppsala University, Statistics Sweden. Sweden National Prevalence Study on Exposure to Violence 2012.
126418	Swaziland National Study on Violence Against Children and Young Women 2007	Eswatini	Centers for Disease Control and Prevention (CDC), United Nations Children's Fund (UNICEF). Swaziland National Study on Violence Against Children and Young Women 2007.
229690	Sweden National Survey of Crime in Close Relationships 2012	Sweden	Government of Sweden, National Council for Crime Prevention (BRA, Sweden). Sweden National Survey of Crime in Close Relationships 2012.

169745	Sweden Gender, Alcohol and Culture: An International Study (GENACIS) 2002	Sweden	Aarhus University, Addiction Switzerland Research Institute, Alcohol Research Group, Public Health Institute, Centre for Addiction and Mental Health (Canada), Centre for Alcohol Policy Research, Turning Point Alcohol and Drug Centre (Australia), Kettil Bruun Society for Social and Epidemiological Research on Alcohol, University of North Dakota. Sweden Gender, Alcohol and Culture: An International Study (GENACIS) 2002.
21331	Tanzania Demographic and Health Survey 2009-2010	United Republic of Tanzania	ICF Macro, National Bureau of Statistics (Tanzania). Tanzania Demographic and Health Survey 2009-2010. Fairfax, United States of America: ICF International.
74460	Tajikistan Demographic and Health Survey 2012	Tajikistan	ICF International, Ministry of Health (Tajikistan), Statistical Agency under the President of the Republic of Tajikistan. Tajikistan Demographic and Health Survey 2012. Fairfax, United States of America: ICF International, 2013.
218593	Tanzania Demographic and Health Survey 2015-2016	United Republic of Tanzania	ICF International, Ministry of Health (Zanzibar), Ministry of Health, Community Development, Gender, Elderly and Children (MoHCDEC) (Tanzania), National Bureau of Statistics (Tanzania), Office of the Chief Government Statistician (OCGS) (Zanzibar). Tanzania Demographic and Health Survey 2015-2016. Fairfax, United States of America: ICF International, 2016.
341838	Tajikistan Demographic and Health Survey 2017	Tajikistan	ICF International, Statistical Agency under the President of the Republic of Tajikistan. Tajikistan Demographic and Health Survey 2017. Fairfax, United States of America: ICF International, 2018.
27297	Tanzania National Panel Survey 2008-2009	United Republic of Tanzania	National Bureau of Statistics (Tanzania). Tanzania Living Standards Measurement Study - Integrated Survey on Agriculture 2008-2009. Dar es Salaam, Tanzania: National Bureau of Statistics (Tanzania).
152098	Switzerland International Violence Against Women Survey 2003	Switzerland	European Institute for Crime Prevention and Control, affiliated with the United Nations (HEUNI), United Nations Interregional Crime and Justice Research Institute (UNICRI), University of Lausanne. Switzerland International Violence Against Women Survey 2003.
538795	Tanzania Demographic and Health Survey 2022	United Republic of Tanzania	Bureau of Statistics (Tanzania), ICF International, Ministry of Health (Tanzania), Ministry of Health (Zanzibar), Office of the Chief Government Statistician (OCGS) (Zanzibar), Tanzania Food and Nutrition Centre. Tanzania Demographic and Health Survey 2022. Fairfax, United States of America: ICF International, 2023.

126419	Tanzania Violence Against Children Study 2009	United Republic of Tanzania	Ministry of Community Development, Gender and Children (MCDGC) (Tanzania), Muhimbili University of Health and Allied Sciences (Tanzania), National Center for Injury Prevention and Control, Centers for Disease Control and Prevention (CDC), United Nations Children's Fund (UNICEF). Tanzania Violence Against Children Study 2009.
150524	Thailand WHO Multi-country Study on Women's Health and Domestic Violence Against Women 2000	Thailand	Foundation for Women (Thailand), Institute for Population and Social Research, Mahidol University (Thailand), World Health Organization (WHO). Thailand WHO Multi-country Study on Women's Health and Domestic Violence Against Women 2000.
150523	Tanzania WHO Multi-country Study on Women's Health and Domestic Violence Against Women 2001-2002	United Republic of Tanzania	Muhimbili University of Health and Allied Sciences (Tanzania), University of Dar es Salaam, World Health Organization (WHO). Tanzania WHO Multi-country Study on Women's Health and Domestic Violence Against Women 2001-2002.
58575	Thailand Reproductive Health Survey 2009	Thailand	Chulalongkorn University, National Statistical Office (Thailand), United Nations Population Fund (UNFPA). Thailand Reproductive Health Survey 2009. 2010.
128657	The effect of intimate partner violence and other forms of violence against women on health	Spain	Vives-Cases C, Ruiz-Cantero MT, Escribà-Agüir V, Miralles JJ. The effect of intimate partner violence and other forms of violence against women on health. J Public Health (Oxf). 2011; 33(1): 15-21.
150544	The First Israeli National Survey on Domestic Violence	Israel	Eisikovits Z, Winstok Z, Fishman G. The First Israeli National Survey on Domestic Violence. Violence Against Women. 2004; 10(7): 729-48.
222884	Teen Dating Violence (Physical and Sexual) Among US High School Students: Findings From the 2013 National Youth Risk Behavior Survey	United States of America	Vagi KJ, O'Malley Olsen E, Basile KC, Vivolo-Kantor AM. Teen Dating Violence (Physical and Sexual) Among US High School Students: Findings From the 2013 National Youth Risk Behavior Survey. JAMA Pediatr. 2015; 169(5): 474-82.
21274	Timor-Leste Demographic and Health Survey 2009-2010	Timor-Leste	ICF Macro, Ministry of Finance (Timor-Leste), National Statistics Directorate (Timor-Leste). Timor-Leste Demographic and Health Survey 2009-2010. Fairfax, United States of America: ICF International.
286785	Timor-Leste Demographic and Health Survey 2016	Timor-Leste	ICF International, National Statistics Directorate (Timor-Leste). Timor-Leste Demographic and Health Survey 2016. Fairfax, United States of America: ICF International, 2018.
126425	Tonga National Study on Domestic Violence Against Women 2008-2009	Tonga	Ma?a Fafine mo e Famili (Tonga). Tonga National Study on Domestic Violence Against Women 2008-2009.
77515	Togo Demographic and Health Survey 2013-2014	Togo	Directorate General of Statistics and National Accounts (Togo), ICF International, Ministry

			of Health (Togo), Ministry of Planning, Development and Zoning (Togo). Togo Demographic and Health Survey 2013-2014. Fairfax, United States of America: ICF International, 2015.
264603	Togo Core Welfare Indicator Questionnaire Survey 2015	Togo	European Union (EU), Government of Togo, National Institute of Statistics and Economic and Demographic Studies (INSEED) (Togo), United Nations Children's Fund (UNICEF), United Nations Development Programme (UNDP), World Bank. Togo Core Welfare Indicator Questionnaire Survey 2015.
453346	Tonga Multiple Indicator Cluster Survey 2019	Tonga	Government of Tonga, Pacific Community Statistics for Development Division, Tonga Department of Statistics, United Nations Children's Fund (UNICEF), United Nations Population Fund (UNFPA). Tonga Multiple Indicator Cluster Survey 2019. New York, United States of America: United Nations Children's Fund (UNICEF), 2020.
151631	Togo Core Welfare Indicator Questionnaire Survey 2011	Togo	African Capacity Building Foundation (ACBF), Directorate General of Statistics and National Accounts (Togo), European Union (EU), Government of Togo, United Nations Children's Fund (UNICEF), United Nations Development Programme (UNDP), World Bank. Togo Core Welfare Indicator Questionnaire Survey 2011.
467160	Timor-Leste Nabilan Health and Life Experiences Baseline Study 2015	Timor-Leste	The Asia Foundation. Timor-Leste Nabilan Health and Life Experiences Baseline Study 2015.
474476	Trinidad and Tobago National Women's Health Survey 2017	Trinidad and Tobago	Global Women?s Institute, George Washington University, Inter-American Development Bank (IDB), QURE (Trinidad and Tobago). Trinidad and Tobago National Women's Health Survey 2017.
507142	Tunisia National Survey on Violence Against Women 2010	Tunisia	National Office for Family and Population, Ministry of Public Health (Tunisia). Tunisia National Survey on Violence Against Women 2010.
126423	Turkey National Research on Domestic Violence Against Women 2008	Türkiye	BNB Consulting (Turkey), ICON-INSTITUTE Consulting Group, Institute of Population Studies, Hacettepe University. Turkey National Research on Domestic Violence Against Women 2008.
21014	Uganda Demographic and Health Survey 2006	Uganda	Macro International, Inc, Uganda Bureau of Statistics. Uganda Demographic and Health Survey 2006. Fairfax, United States of America: ICF International.
239733	Turkey National Research on Domestic Violence Against Women 2014	Türkiye	Institute of Population Studies, Hacettepe University, Ministry of Family and Social Policies (Turkey). Turkey National Research on Domestic Violence Against Women 2014.
152669	Uganda Gender Based Violence Survey 2009	Uganda	Uganda Bureau of Statistics, Northern Uganda Social Action Fund - Youth Opportunites

			Baseline Survey 2008 (NUSAF - YOP 2008), Version 1.0 of the public use dataset (February 2009), provided by the Uganda National Data Archive. www.ubos.org/nada
56021	Uganda Demographic and Health Survey 2011	Uganda	ICF Macro, Uganda Bureau of Statistics. Uganda Demographic and Health Survey 2011. Fairfax, United States of America: ICF International.
286780	Uganda Demographic and Health Survey 2016	Uganda	ICF International, Uganda Bureau of Statistics. Uganda Demographic and Health Survey 2016. Fairfax, United States of America: ICF International, 2018.
169748	Uganda Gender, Alcohol and Culture: An International Study (GENACIS) 2003	Uganda	Aarhus University, Addiction Switzerland Research Institute, Alcohol Research Group, Public Health Institute, Centre for Addiction and Mental Health (Canada), Centre for Alcohol Policy Research, Turning Point Alcohol and Drug Centre (Australia), Kettil Bruun Society for Social and Epidemiological Research on Alcohol, University of North Dakota. Uganda Gender, Alcohol and Culture: An International Study (GENACIS) 2003.
23289	Uganda Child Verbal Autopsy Study 2007	Uganda	MEASURE Evaluation Project, Carolina Population Center, University of North Carolina, Macro International, Inc, Ministry of Health (Uganda), Uganda Bureau of Statistics. Uganda Child Verbal Autopsy Study 2007. Calverton, United States of America: Macro International, Inc.
18818	Tuvalu Demographic and Health Survey 2007	Tuvalu	Macro International, Inc, Secretariat of the Pacific Community (SPC), Tuvalu Central Statistics Division. Tuvalu Demographic and Health Survey 2007.
489863	Tuvalu Multiple Indicator Cluster Survey 2019-2020	Tuvalu	Tuvalu Central Statistics Division, United Nations Children's Fund (UNICEF). Tuvalu Multiple Indicator Cluster Survey 2019-2020. New York, United States of America: United Nations Children's Fund (UNICEF), 2021.
508087	Turkmenistan Health and Status of a Woman in the Family 2020	Turkmenistan	Ministry of Health and Medical Industry (Turkmenistan), United Nations Population Fund (UNFPA). Turkmenistan Health and Status of a Woman in the Family 2020.
21024	Ukraine Demographic and Health Survey 2007	Ukraine	Macro International, Inc, State Statistical Committee (Ukraine), Ukrainian Center for Social Reforms (UCSR). Ukraine Demographic and Health Survey 2007. Fairfax, United States of America: ICF International.
327593	Uganda Population-Based HIV Impact Assessment 2016-2017	Uganda	Centers for Disease Control and Prevention (CDC), ICAP, Columbia University Mailman School of Public Health, Joint United Nations Program on HIV/AIDS (UNAIDS), Ministry of Health (Uganda), Uganda Bureau of Statistics, Uganda Virus Research Institute, World Health Organization (WHO). Uganda

			Population-Based HIV Impact Assessment 2016-2017. New York, New York: ICAP, Columbia University Mailman School of Public Health, 2021.
13218	Ukraine Reproductive Health Survey 1999	Ukraine	Division of Reproductive Health-Centers for Disease Control and Prevention (CDC) and Kiev International Institute of Sociology. (2001) Ukraine Reproductive Health Survey 1999. Atlanta, United States: Centers for Disease Control and Prevention (CDC).
474227	Ukraine OSCE-Led Survey on the Well-Being and Safety of Women 2018	Ukraine	Ipsos, Organization for Security and Co-operation in Europe (OSCE). Ukraine OSCE-Led Survey on the Well-Being and Safety of Women 2018. 2019.
505020	Understanding Masculinities: Results from the International Men and Gender Equality Survey (IMAGES) - Middle East and North Africa	Egypt; Lebanon; Morocco; Palestine	Promundo, United Nations Entity for Gender Equality and the Empowerment of Women (UN Women). Understanding Masculinities: Results from the International Men and Gender Equality Survey (IMAGES) - Middle East and North Africa. Promundo, 2017.
506506	Ukraine Prevalence of Violence Against Women and Girls 2014	Ukraine	GfK Ukraine. Ukraine Prevalence of Violence Against Women and Girls 2014.
554627	Uganda National Survey on Violence 2020	Uganda	Uganda Bureau of Statistics. Uganda National Survey on Violence 2020.
196210	United Kingdom - Scottish Crime and Justice Survey 2008-2009 - UK Data Service	United Kingdom	TNS-BMRB Scotland, Scottish Government. (2010). Scottish Crime and Justice Survey, 2008-2009. [data collection]. UK Data Service. SN: 6362, http://dx.doi.org/10.5255/UKDA-SN-6362-1.
196211	United Kingdom - Scottish Crime and Justice Survey 2009-2010 - UK Data Service	United Kingdom	TNS-BMRB Scotland and Scottish Government, Scottish Crime and Justice Survey, 2009-2010 [computer file]. Colchester, Essex: UK Data Archive [distributor], August 2011. SN: 6685, http://dx.doi.org/10.5255/UKDA-SN-6685-1.
196212	United Kingdom - Scottish Crime and Justice Survey 2010-2011 - UK Data Service	United Kingdom	TNS BMRB Scotland and Scottish Government, Scottish Crime and Justice Survey, 2010-2011 [computer file]. Colchester, Essex: UK Data Archive [distributor], March 2013. SN: 7229, http://dx.doi.org/10.5255/UKDA-SN-7229-1.
283814	United Kingdom - Scottish Crime and Justice Survey 2012-2013 - Scottish Government	United Kingdom	Scottish Government. United Kingdom - Scottish Crime and Justice Survey 2012-2013 - Scottish Government.
283817	United Kingdom - Scottish Crime and Justice Survey 2014-2015 - Scottish Government	United Kingdom	Scottish Government. United Kingdom - Scottish Crime and Justice Survey 2014-2015 - Scottish Government. Edinburgh, Scotland: Scottish Government, 2016.
126610	United Kingdom - England Adult Psychiatric Morbidity Survey 2006-2007 - UK Data Service	United Kingdom	National Centre for Social Research and University of Leicester, Adult Psychiatric Morbidity Survey, 2007 [computer file]. 3rd Edition. Colchester, Essex: UK Data Archive

			[distributor], January 2011. SN: 6379, http://dx.doi.org/10.5255/UKDA-SN-6379-1
150469	United Kingdom - Homicides, Firearm Offences and Intimate Violence 2006/07: Supplementary Volume 2 to Crime in England and Wales 2006/07	United Kingdom	Home Office (United Kingdom). Homicides, Firearm Offences and Intimate Violence 2006/07: Supplementary Volume 2 to Crime in England and Wales 2006/07. London, United Kingdom: Home Office (United Kingdom), 2008.
150592	United Kingdom - Domestic Violence: Findings From a New British Crime Survey Self-completion Questionnaire	United Kingdom	Home Office (United Kingdom). Domestic Violence: Findings From a New British Crime Survey Self-completion Questionnaire. London, United Kingdom: Home Office (United Kingdom), 1999.
169749	United Kingdom Gender, Alcohol and Culture: An International Study (GENACIS) 2000	United Kingdom	Aarhus University, Addiction Switzerland Research Institute, Alcohol Research Group, Public Health Institute, Centre for Addiction and Mental Health (Canada), Centre for Alcohol Policy Research, Turning Point Alcohol and Drug Centre (Australia), Kettil Bruun Society for Social and Epidemiological Research on Alcohol, University of North Dakota. United Kingdom Gender, Alcohol and Culture: An International Study (GENACIS) 2000.
124529	United Kingdom - Scottish Crime Survey 2000 - UK Data Service	United Kingdom	MVA Limited and Scottish Government, Scottish Crime Survey, 2000 [computer file]. Colchester, Essex: UK Data Archive [distributor], July 2002. SN: 4542, http://dx.doi.org/10.5255/UKDA-SN-4542-1.
148648	United States Commonwealth Fund Survey of Women's Health 1998	United States of America	Louis Harris and Associates. United States Commonwealth Fund Survey of Women's Health 1998. New York, United States of America: Commonwealth Fund, 1999.
169752	United States Gender, Alcohol and Culture: An International Study (GENACIS) 1995-1996	United States of America	Aarhus University, Addiction Switzerland Research Institute, Alcohol Research Group, Public Health Institute, Centre for Addiction and Mental Health (Canada), Centre for Alcohol Policy Research, Turning Point Alcohol and Drug Centre (Australia), Kettil Bruun Society for Social and Epidemiological Research on Alcohol, University of North Dakota. United States Gender, Alcohol and Culture: An International Study (GENACIS) 1995-1996.
283356	United States High School Youth Risk Behavior Survey 1991-2015	United States of America	Centers for Disease Control and Prevention (CDC). United States High School Youth Risk Behavior Survey 1991-2015. Atlanta, United States of America: Centers for Disease Control and Prevention (CDC).
169751	United States Gender, Alcohol and Culture: An International Study (GENACIS) 2001	United States of America	Aarhus University, Addiction Switzerland Research Institute, Alcohol Research Group, Public Health Institute, Centre for Addiction and Mental Health (Canada), Centre for Alcohol Policy Research, Turning Point

			Alcohol and Drug Centre (Australia), Kettil Bruun Society for Social and Epidemiological Research on Alcohol, University of North Dakota. United States Gender, Alcohol and Culture: An International Study (GENACIS) 2001.
29983	United States Behavioral Risk Factor Surveillance System 2005	United States of America	Centers for Disease Control and Prevention (CDC). United States Behavioral Risk Factor Surveillance System 2005. Atlanta, Georgia: CDC, US Department of Health and Human Services.
30000	United States Behavioral Risk Factor Surveillance System 2007	United States of America	Centers for Disease Control and Prevention (CDC). United States Behavioral Risk Factor Surveillance System 2007. Atlanta, Georgia: CDC, US Department of Health and Human Services.
212526	United States Collaborative Psychiatric Epidemiology Surveys 2001-2003	United States of America	Margarita Algeria, James S. Jackson, Ronald C. Kessler, and David Takeuchi. Collaborative Psychiatric Epidemiology Surveys (CPES), 2001-2003 [United States]. ICPSR20240-v7. Ann Arbor, MI: Inter-university Consortium for Political and Social Research [distributor], 2015-05-28. http://doi.org/10.3886/ICPSR20240.v7
124091	United States National Epidemiologic Survey on Alcohol and Related Conditions 2004-2005	United States of America	National Institute on Alcohol Abuse and Alcoholism (NIAAA), National Institutes of Health (NIH), U.S. Department of Health and Human Services. United States National Epidemiologic Survey on Alcohol and Related Conditions 2004-2005.
126439	United States National Intimate Partner and Sexual Violence Survey 2010	United States of America	National Center for Injury Prevention and Control, Centers for Disease Control and Prevention (CDC), Research Triangle Institute, Inc. (RTI). United States National Intimate Partner and Sexual Violence Survey 2010.
509789	United States National Intimate Partner and Sexual Violence Survey 2016-2018	United States of America	National Center for Injury Prevention and Control, Centers for Disease Control and Prevention (CDC), RTI International. United States National Intimate Partner and Sexual Violence Survey 2016-2018.
150625	United States National Violence Against Women Survey 1995-1996	United States of America	Centers for Disease Control and Prevention (CDC), National Institute of Justice (United States), Schulman, Ronca and Bucuvalas Inc. (SRBI). United States National Violence Against Women Survey 1995-1996.
205347	United States National Youth Risk Behavior Survey 2015	United States of America	Centers for Disease Control and Prevention (CDC). United States National Youth Risk Behavior Survey 2015. Atlanta, United States of America: Centers for Disease Control and Prevention (CDC), 2016.
318061	United States National Youth Risk Behavior Survey 2017	United States of America	Centers for Disease Control and Prevention (CDC). United States National Youth Risk Behavior Survey 2017. Atlanta, United States

			of America: Centers for Disease Control and Prevention (CDC).
558877	United States National Youth Risk Behavior Survey 2021	United States of America	Centers for Disease Control and Prevention (CDC). United States National Youth Risk Behavior Survey 2021. Atlanta, United States of America: Centers for Disease Control and Prevention (CDC).
354800	Uruguay National Prevalence Survey on Violence Based on Gender and Generations 2013	Uruguay	Ministry of Public Health (Uruguay), Ministry of Social Development (Uruguay), National Institute of Statistics (Uruguay), National Institute of Women (Uruguay). Uruguay National Prevalence Survey on Violence Based on Gender and Generations 2013. Montevideo, Uruguay: National Institute of Statistics (Uruguay).
169750	Uruguay Gender, Alcohol and Culture: An International Study (GENACIS) 2004	Uruguay	Aarhus University, Addiction Switzerland Research Institute, Alcohol Research Group, Public Health Institute, Centre for Addiction and Mental Health (Canada), Centre for Alcohol Policy Research, Turning Point Alcohol and Drug Centre (Australia), Kettil Bruun Society for Social and Epidemiological Research on Alcohol, University of North Dakota. Uruguay Gender, Alcohol and Culture: An International Study (GENACIS) 2004.
474232	Vanuatu National Survey on Women's Lives and Family Relationships 2009	Vanuatu	National Statistics Office (Vanuatu), Vanuatu Women's Centre. Vanuatu National Survey on Women's Lives and Family Relationships 2009.
507443	Venezuela Demographic Survey 2010	Venezuela (Bolivarian Republic of)	National Institute of Statistics (Venezuela). Venezuela Demographic Survey 2010.
540927	United States Virgin Islands Youth Risk Behavior Survey 2017	United States of America	Department of Health (United States Virgin Islands), University of the Virgin Islands (United States). United States Virgin Islands Youth Risk Behavior Survey 2017.
126424	Vietnam National Study of Domestic Violence Against Women 2009-2010	Viet Nam	General Statistics Office (Vietnam), World Health Organization (WHO). Vietnam National Study of Domestic Violence Against Women 2009-2010.
355055	Violence against children, later victimisation, and mental health: a cross-sectional study of the general Norwegian population	Norway	Thoresen S, Myhre M, Wentzel-Larsen T, Aakvaag HF, Hjemdal OK. Violence against children, later victimisation, and mental health: a cross-sectional study of the general Norwegian population. Eur J Psychotraumatol. 2015; 6(1): 26259.
137206	Violence Against Women: An International Perspective	Australia; Canada; China; Costa Rica; Czechia; Denmark; Greece; Italy; Mozambique; Philippines; Poland; Switzerland	Johnson H, Ollus N, Nevala S. Violence Against Women: An International Perspective. New York City, United States: Springer; 2008.

137259	Violence against young Australian women and association with reproductive events: a cross-sectional analysis of a national population sample	Australia	Taft AJ, Watson LF, Lee C. Violence against young Australian women and association with reproductive events: a cross-sectional analysis of a national population sample. Aust N Z J Public Health. 2004; 28(4): 324-9.
137272	Violence and PTSD in Mexico: gender and regional differences	Mexico	Baker CK, Norris FH, Diaz DMV, Perilla JL, Murphy AD, Hill EG. Violence and PTSD in Mexico: gender and regional differences. Soc Psychiatry Psychiatr Epidemiol. 2005; 40(7): 519-28.
150543	Wife abuse in Esfahan, Islamic Republic of Iran, 2002	Iran (Islamic Republic of)	Mousavi SM, Eshagian A. Wife abuse in Esfahan, Islamic Republic of Iran, 2002. East Mediterr Health J. 2005; 11(5-6): 860-9.
150605	Wife abuse in Hong Kong Chinese Families: A Community Survey	China	Tang CS. Wife abuse in Hong Kong Chinese Families: A Community Survey. J Fam Violence. 1999; 14(2): 173-191.
150629	Violence against women in India: evidence from rural Maharashtra, India	India	Jain D, Sanon S, Sadowski L, Hunter W. Violence against women in India: evidence from rural Maharashtra, India. Rural Remote Health. 2004; 4(4): 304.
508124	Viet Nam National Study on Violence Against Women 2018-2019	Viet Nam	General Statistics Office (Vietnam), Ministry of Labour, Invalids and Social Affairs (MOLISA) (Vietnam), United Nations Population Fund (UNFPA). Viet Nam National Study on Violence Against Women 2018-2019.
508246	Violence Against Women in Intimate Partnerships: First Findings of Follow-up Research to IVAWS 2003	Czechia	Pikálková S. Violence Against Women in Intimate Partnerships: First Findings of Follow-up Research to IVAWS 2003. Acta Univ Carol Philos Hist. 2015; 2012(2): 85-100.
21117	Zambia Demographic and Health Survey 2007	Zambia	Central Statistical Office (Zambia), Macro International, Inc. Zambia Demographic and Health Survey 2007. Fairfax, United States of America: ICF International.
408815	Zambia Violence Against Children Survey (VACS) 2014	Zambia	Centers for Disease Control and Prevention (CDC), Central Statistical Office (Zambia), Ministry of Community Development and Social Services (MCDSS) (Zambia), Ministry of Youth Sport and Child Development (Zambia), Save the Children International, Together for Girls, United Nations Children's Fund (UNICEF), University of Zambia. Zambia Violence Against Children Survey (VACS) 2014.
21163	Zimbabwe Demographic and Health Survey 2005-2006	Zimbabwe	Central Statistical Office (Zimbabwe), Macro International, Inc. Zimbabwe Demographic and Health Survey 2005-2006. Fairfax, United States of America: ICF International.
21102	Zambia Demographic and Health Survey 2001-2002	Zambia	Central Board of Health (Zambia), Central Statistical Office (Zambia), Macro International, Inc. Zambia Demographic and Health Survey 2001-2002. Fairfax, United States of America: ICF International.

338552	Zimbabwe Violence Against Women Baseline Survey 2012	Zimbabwe	Gender Links (South Africa), Ministry of Women's Affairs, Gender and Community Development (Zimbabwe), Zimbabwe National Statistics Agency. Zimbabwe Violence Against Women Baseline Survey 2012.
77516	Zambia Demographic and Health Survey 2013-2014	Zambia	Central Statistical Office (Zambia), ICF International, Ministry of Health (Zambia), Tropical Diseases Research Centre, University Teaching Hospital (Zambia), University of Zambia. Zambia Demographic and Health Survey 2013-2014. Fairfax, United States of America: ICF International.
157066	Zimbabwe Demographic and Health Survey 2015	Zimbabwe	ICF International, National Microbiology Reference Laboratory, Harare Central Hospital (NMRL) (Zimbabwe), Zimbabwe National Statistics Agency. Zimbabwe Demographic and Health Survey 2015. Fairfax, United States of America: ICF International, 2016.
55992	Zimbabwe Demographic and Health Survey 2010-2011	Zimbabwe	ICF Macro, Zimbabwe National Statistics Agency. Zimbabwe Demographic and Health Survey 2010-2011. Calverton, United States of America: ICF Macro, 2012.
128660	Women's Status and Violence against Young Married Women in Rural Nepal	Nepal	Lamichhane P, Puri M, Tamang J, Dulal B. Women's Status and Violence against Young Married Women in Rural Nepal. BMC Womens Health. 2011; 19.
238506	Women are considerably more exposed to intimate partner violence than men in Rwanda: results from a population-based, cross-sectional study	Rwanda	Umubyeyi A, Mogren I, Ntaganira J, Krantz G. Women are considerably more exposed to intimate partner violence than men in Rwanda: results from a population-based, cross-sectional study. BMC Womens Health. 2014; 14: 99.
287630	Zambia Population-Based HIV Impact Assessment 2016	Zambia	Centers for Disease Control and Prevention (CDC), ICAP, Columbia University Mailman School of Public Health, Ministry of Health (Zambia), Statistical Center for HIV/AIDS Research and Prevention (SCHARP), Tropical Diseases Research Centre, University of Zambia, Westat. Zambia Population-Based HIV Impact Assessment 2016. New York, New York: ICAP, Columbia University Mailman School of Public Health.
431951	Zimbabwe Multiple Indicator Cluster Survey 2019	Zimbabwe	United Nations Children's Fund (UNICEF), Zimbabwe National Statistics Agency. Zimbabwe Multiple Indicator Cluster Survey 2019. New York, United States of America: United Nations Children's Fund (UNICEF), 2020.
411301	Zambia Demographic and Health Survey 2018-2019	Zambia	Central Statistical Office (Zambia), ICF International, Ministry of Health (Zambia), University Teaching Hospital (Zambia), University of Zambia. Zambia Demographic

			and Health Survey 2018-2019. Fairfax, United States of America: ICF International, 2020.
287631	Zimbabwe Population-Based HIV Impact Assessment 2015-2016	Zimbabwe	Biomedical Research and Training Institute (Zimbabwe), Centers for Disease Control and Prevention (CDC), ICAP, Columbia University Mailman School of Public Health, Ministry of Health and Child Welfare (Zimbabwe), National AIDS Council (Zimbabwe), Statistical Center for HIV/AIDS Research and Prevention (SCHARP), Westat, Zimbabwe National Statistics Agency. Zimbabwe Population-Based HIV Impact Assessment 2015-2016. New York, New York: ICAP, Columbia University Mailman School of Public Health, 2021.
137187	[Prevalence of sexual abuse in students and its relation with drug abuse]	Mexico	Ramos-Lira L, Saldívar-Hernández G, Medina-Mora ME, Rojas-Guiot E, Villatoro-Velázquez J. [Prevalence of sexual abuse in students and its relation with drug abuse]. Salud Publica Mex. 1998; 40(3): 221-33.
137260	[Intimate partner sexual violence among men and women in urban Brazil, 2005]	Brazil	Schraiber LB, D'Oliveira AFPL, França Junior I. [Intimate partner sexual violence among men and women in urban Brazil, 2005]. Rev Saude Publica. 2008; 127-37.
150631	[Prevalence of violent events and post-traumatic stress disorder in the Mexican population]	Mexico	Medina-Mora Icaza ME, Borges-Guimaraes G, Lara C, Ramos-Lira L, Zambrano J, Fleiz-Bautista C. [Prevalence of violent events and post-traumatic stress disorder in the Mexican population]. Salud Publica Mex. 2005; 47(1): 8-22.

Table S4: Data sources on the prevalence of sexual violence against children

GHDx Record ID	GHDx Record Title	Geography	Suggested Citation
27321	Albania Reproductive Health Survey 2002	Albania	Albania Institute of Public Health (IPH), Ministry of Health (Albania), National Institute of Statistics (Albania), and Centers for Disease Control and Prevention. (2005) Albania Reproductive Health Survey 2001. Atlanta, United States: Centers for Disease Control and Prevention (CDC).
157018	Afghanistan Demographic and Health Survey 2015-2016	Afghanistan	Central Statistics Organization (Afghanistan), ICF International, Ministry of Public Health (Afghanistan). Afghanistan Demographic and Health Survey 2015-2016. Fairfax, United States of America: ICF International, 2017.
137276	A determination of the prevalence of gender-based violence among conflict-affected populations in East Timor	Timor-Leste	Hynes M, Robertson K, Ward J, Crouse C. A determination of the prevalence of gender-based violence among conflict-affected populations in East Timor. Disasters. 2004; 28(3): 294-321.

126437	Albania Balkan Epidemiological Study on Child Abuse and Neglect 2011	Albania	Children?s Human Rights Centre of Albania (CRCA), Institute of Child Health (Greece). Albania Balkan Epidemiological Study on Child Abuse and Neglect 2011.
137159	Adverse childhood events are associated with obesity and disordered eating: Results from a U.S. population-based survey of young adults	United States of America	Fuemmeler BF, Dedert E, McClernon FJ, Beckham JC. Adverse childhood events are associated with obesity and disordered eating: Results from a U.S. population-based survey of young adults. J Trauma Stress. 2009; 22(4): 329-33.
137173	Adolescent sexuality in Saint Petersburg, Russia	Russian Federation	Lunin I, Hall TL, Mandel JS, Kay J, Hearst N. Adolescent sexuality in Saint Petersburg, Russia. AIDS. 1995; 9(Suppl 1): S53-60.
137174	A population-based study of childhood sexual contact in China: prevalence and long-term consequences	China	Luo Y, Parish WL, Laumann EO. A population-based study of childhood sexual contact in China: prevalence and long-term consequences. Child Abuse Negl. 2008; 32(7): 721-31.
137183	Alcohol and sexual victimization: a longitudinal study of Norwegian girls	Norway	Pedersen W, Skrondal A. Alcohol and sexual victimization: a longitudinal study of Norwegian girls. Addiction. 1996; 91(4): 565-81.
148644	Adolescent Violence Perpetration: Associations With Multiple Types of Adverse Childhood Experiences	United States of America	Duke NN, Pettingell SL, McMorris BJ, Borowsky IW. Adolescent Violence Perpetration: Associations With Multiple Types of Adverse Childhood Experiences. Pediatrics. 2010; 125(4): e778?e786.
506103	Albania National Violence Against Women Survey 2018	Albania	Albania Institute of Statistics (INSTAT), United Nations Development Programme (UNDP), United Nations Entity for Gender Equality and the Empowerment of Women (UN Women). Albania National Violence Against Women Survey 2018.
218563	Armenia Demographic and Health Survey 2015-2016	Armenia	ICF International, Ministry of Health (Armenia), National Statistical Service of the Republic of Armenia. Armenia Demographic and Health Survey 2015-2016. Fairfax, United States of America: ICF International, 2017.
168024	Argentina - Buenos Aires Gender, Alcohol and Culture: An International Study (GENACIS) 2003	Argentina	Aarhus University, Addiction Switzerland Research Institute, Alcohol Research Group, Public Health Institute, Centre for Addiction and Mental Health (Canada), Centre for Alcohol Policy Research, Turning Point Alcohol and Drug Centre (Australia), Kettil Bruun Society for Social and Epidemiological Research on Alcohol, University of North Dakota. Argentina - Buenos Aires Gender, Alcohol and Culture: An International Study (GENACIS) 2003.
530321	American Samoa Adult Hybrid Survey 2017-2018	United States of America	American Samoa Department of Human and Social Services, Department of Health (American Samoa), Pacific Islands Health Officers Association (PIHOA). American Samoa Adult Hybrid Survey 2017-2018.

137160	Attempted and completed incidents of stranger-perpetrated child sexual abuse and abduction	United Kingdom	Gallagher B, Bradford M, Pease K. Attempted and completed incidents of stranger-perpetrated child sexual abuse and abduction. Child Abuse Negl. 2008; 32(5): 517-28.
150429	Associations between sexual abuse and family conflict/violence, self-injurious behavior, and substance use: the mediating role of depressed mood and anger	Iceland	Asgeirsdottir BB, Sigfusdottir ID, Gudjonsson GH, Sigurdsson JF. Associations between sexual abuse and family conflict/violence, self-injurious behavior, and substance use: the mediating role of depressed mood and anger. Child Abuse Negl. 2011; 35(3): 210-9.
156681	Assessment of first sexual intercourse in young women with a history of childhood sexual abuse	Australia	Moore EE, Jayasinghe Y, Olsson CA, Romaniuk H, Sasongko V, Patton GC, Garland SM. Assessment of first sexual intercourse in young women with a history of childhood sexual abuse. Aust N Z J Obstet Gynaecol. 2011; 51(3): 276-79.
506154	Armenia Nationwide Survey on Violence Against Women 2008	Armenia	National Statistical Service of the Republic of Armenia, United Nations Population Fund (UNFPA). Armenia Nationwide Survey on Violence Against Women 2008.
527998	Armenia Survey on Domestic Violence Against Women 2021	Armenia	International Center for Human Development (ICHD), Statistical Committee of the Republic of Armenia. Armenia Survey on Domestic Violence Against Women 2021.
18865	Azerbaijan Demographic and Health Survey 2006	Azerbaijan	Macro International, Inc, State Statistical Committee of Azerbaijan. Azerbaijan Demographic and Health Survey 2006. Fairfax, United States of America: ICF International.
137014	Australia Personal Safety Survey 2005	Australia	Australian Bureau of Statistics. Australia Personal Safety Survey 2005.
341276	Australia Personal Safety Survey 2016-2017	Australia	Australian Bureau of Statistics, Department of Social Services (Australia). Australia Personal Safety Survey 2016-2017.
504375	Azerbaijan International Men and Gender Equality Study 2016	Azerbaijan	International Center for Research on Women, International Center for Social Research (ICSR) (Azerbaijan), Promundo, State Committee for Family, Women and Children's Affairs (Azerbaijan), United Nations Population Fund (UNFPA). Azerbaijan International Men and Gender Equality Study 2016.
516408	Australian Longitudinal Study on Women's Health 1989-1995 Cohort Summary Surveys 1-5 (2013-2017)	Australia	University of Newcastle (Australia), University of Queensland (Australia). Australian Longitudinal Study on Women's Health 1989-1995 Cohort Summary Surveys 1-5 (2013-2017). Australia: Australian Longitudinal Study on Women's Health, 2019.
18913	Bangladesh Demographic and Health Survey 2007	Bangladesh	Macro International, Inc, Mitra and Associates, National Institute of Population Research and Training (NIPORT). Bangladesh Demographic and Health Survey 2007. Fairfax, United States of America: ICF International, 2009.

95474	Bangladesh Urban Health Survey 2006	Bangladesh	Associates for Community and Population Research (ACPR), International Centre for Diarrhoeal Disease Research, Bangladesh (ICDDR,B), MEASURE Evaluation Project, Carolina Population Center, University of North Carolina, National Institute of Population Research and Training (NIPORT). Bangladesh Urban Health Survey 2006.
150513	Bangladesh WHO Multi-country Study on Women's Health and Domestic Violence Against Women 2001	Bangladesh	International Centre for Diarrhoeal Disease Research, Bangladesh (ICDDR,B), Naripokkho, Uppsala University, World Health Organization (WHO). Bangladesh WHO Multi-country Study on Women's Health and Domestic Violence Against Women 2001.
437407	Bangladesh Violence Against Women Survey 2015	Bangladesh	Bangladesh Bureau of Statistics (BBS). Bangladesh Violence Against Women Survey 2015. Dhaka, Bangladesh: Bangladesh Bureau of Statistics (BBS).
437416	Bangladesh Violence Against Women Survey 2011	Bangladesh	Bangladesh Bureau of Statistics (BBS). Bangladesh Violence Against Women Survey 2011. Dhaka, Bangladesh: Bangladesh Bureau of Statistics (BBS).
218565	Benin Demographic and Health Survey 2017-2018	Benin	Hubert Koutoukou Maga National University Hospital Center (CNHU-HKM)(Benin), ICF International, National Institute of Statistics and Economic Analysis (INSAE) (Benin), National Malaria Control Program, Ministry of Health (Benin), Permanent Secretariat of the Food Council and Nutrition (SP-CAN)(Benin). Benin Demographic and Health Survey 2017-2018. Fairfax, United States of America: ICF International, 2018.
169718	Belize Gender, Alcohol and Culture: An International Study (GENACIS) 2005	Belize	Aarhus University, Addiction Switzerland Research Institute, Alcohol Research Group, Public Health Institute, Centre for Addiction and Mental Health (Canada), Centre for Alcohol Policy Research, Turning Point Alcohol and Drug Centre (Australia), Kettil Bruun Society for Social and Epidemiological Research on Alcohol, University of North Dakota. Belize Gender, Alcohol and Culture: An International Study (GENACIS) 2005.
506157	Belgium Emotional, Physical and Sexual Abuse Study 2009	Belgium	Ghent University, Institute for the Equality of Women and Men (Belgium), University of Liège (Belgium). Belgium Emotional, Physical and Sexual Abuse Study 2009.
539788	Benin Multiple Indicator Cluster Survey 2021-2022	Benin	National Institute of Statistics and Demography (INStaD) (Benin), United Nations Children's Fund (UNICEF). Benin Multiple Indicator Cluster Survey 2021-2022. New York, United States of America: United Nations Children's Fund (UNICEF), 2023.

286787	Botswana Gender Based Violence Indicators Study 2011	Botswana	Gender Links (South Africa), Women's Affairs Department, Ministry of Labour and Home Affairs (Botswana). Botswana Gender Based Violence Indicators Study 2011.
134753	Botswana AIDS Impact Survey 2013	Botswana	Ministry of Health (Botswana), National AIDS Coordinating Agency (Botswana), Statistics Botswana. Botswana AIDS Impact Survey 2013. Gaborone, Botswana: Statistics Botswana, 2015.
165290	Bhutan Health Survey 2012-2013	Bhutan	Ministry of Health (Bhutan), National Statistics Bureau (Bhutan), United Nations Population Fund (UNFPA). Bhutan Health Survey 2012-2013.
126436	Bosnia and Herzegovina Balkan Epidemiological Study on Child Abuse and Neglect 2011	Bosnia and Herzegovina	Institute of Child Health (Greece), University of Sarajevo. Bosnia and Herzegovina Balkan Epidemiological Study on Child Abuse and Neglect 2011.
22116	Botswana AIDS Impact Survey 2008	Botswana	Central Statistics Office (Botswana), National AIDS Coordinating Agency (Botswana). Botswana AIDS Impact Survey 2008. Gaborone, Botswana: Statistics Botswana, 2015.
506160	Bhutan National Study on Women's Health and Life Experiences 2017	Bhutan	National Commission for Women and Children (NCWC) (Bhutan). Bhutan National Study on Women's Health and Life Experiences 2017.
142941	Brazil National Alcohol and Drugs Survey 2011-2012	Brazil	Ipsos, National Institute of Public Policy for Alcohol and Other Drugs (INPAD) (Brazil), University of São Paulo. Brazil National Alcohol and Drugs Survey 2011-2012.
432810	Botswana Violence Against Children Survey 2016	Botswana	5 AM Holdings (PTY) LTD (Botswana), Centers for Disease Control and Prevention (CDC), Department of Social Protection (Botswana), Together for Girls, United Nations Children's Fund (UNICEF). Botswana Violence Against Children Survey 2016. Washington, DC, United States of America: Together for Girls.
281642	Brazil National Survey of School Health 2015	Brazil	Brazilian Institute of Geography and Statistics (IBGE). Brazil National Survey of School Health 2015. Rio de Janeiro, Brazil: Brazilian Institute of Geography and Statistics (IBGE), 2016.
169719	Brazil - Botucatu Gender, Alcohol and Culture: An International Study (GENACIS) 2001-2002	Brazil	Aarhus University, Addiction Switzerland Research Institute, Alcohol Research Group, Public Health Institute, Centre for Addiction and Mental Health (Canada), Centre for Alcohol Policy Research, Turning Point Alcohol and Drug Centre (Australia), Kettil Bruun Society for Social and Epidemiological Research on Alcohol, University of North Dakota. Brazil - Botucatu Gender, Alcohol and Culture: An International Study (GENACIS) 2001-2002.

238854	Cambodia WHO Multi-country Study on Women's Health and Domestic Violence Against Women 2015	Cambodia	Ministry of Women's Affairs (Cambodia), National Institute of Statistics (Cambodia), World Health Organization (WHO). Cambodia WHO Multi-country Study on Women's Health and Domestic Violence Against Women 2015. Phnom Penh, Cambodia: National Institute of Statistics (Cambodia).
286766	Burundi Demographic and Health Survey 2016-2017	Burundi	Burundi Institute of Statistics and Economic Studies, ICF International, Ministry of Public Health and the Fight Against AIDS (Burundi). Burundi Demographic and Health Survey 2016-2017. Fairfax, United States of America: ICF International, 2018.
19133	Burkina Faso Demographic and Health Survey 2010-2011	Burkina Faso	ICF Macro, Ministry of Health (Burkina Faso), National Institute of Statistics and Demography (Burkina Faso). Burkina Faso Demographic and Health Survey 2010-2011. Fairfax, United States of America: ICF International.
150514	Brazil WHO Multi-country Study on Women's Health and Domestic Violence Against Women 2000-2001	Brazil	Federal University of Pernambuco, Feminist Collective for Health and Sexuality (São Paulo), University of São Paulo, World Health Organization (WHO). Brazil WHO Multi-country Study on Women's Health and Domestic Violence Against Women 2000-2001.
126429	Bulgaria Balkan Epidemiological Study on Child Abuse and Neglect 2011	Bulgaria	Institute of Child Health (Greece), South-West University ""Neofit Rilski"". Bulgaria Balkan Epidemiological Study on Child Abuse and Neglect 2011.
493322	Burkina Faso Performance Monitoring and Action Phase 2 Household and Female Survey 2020-2021	Burkina Faso	Bill and Melinda Gates Institute for Population and Reproductive Health, Johns Hopkins Bloomberg School of Public Health, Higher Institute of Population Sciences (ISSP) (Burkina Faso), Jhpiego. Burkina Faso Performance Monitoring and Action Phase 2 Household and Female Survey 2020-2021. Baltimore, United States of America: Johns Hopkins Bloomberg School of Public Health, 2021.
539968	Bulgaria Violence Against Children Study 2019	Bulgaria	Coram International, United Nations Children's Fund (UNICEF). Bulgaria Violence Against Children Study 2019.
540012	Burkina Faso National Study on Violence Against Children 2018	Burkina Faso	Ministry of Women, National Solidarity, Family and Humanitarian Action (Burkina Faso). Burkina Faso National Study on Violence Against Children 2018.
527622	Burkina Faso Demographic and Health Survey 2021	Burkina Faso	ICF International, National Institute of Public Health (INSP) (Burkina Faso), National Institute of Statistics and Demography (Burkina Faso). Burkina Faso Demographic and Health Survey 2021.
157024	Cambodia Demographic and Health Survey 2014	Cambodia	ICF International, Ministry of Health (Cambodia), National Institute of Statistics

			(Cambodia). Cambodia Demographic and Health Survey 2014. Fairfax, United States of America: ICF International, 2017.
231764	Cambodia Violence Against Children Survey 2013	Cambodia	Centers for Disease Control and Prevention (CDC), Ministry of Social Affairs, Veterans and Youth Rehabilitation (MoSVY) (Cambodia), Ministry of Women's Affairs (Cambodia), National Institute of Statistics (Cambodia), Together for Girls, United Nations Children's Fund (UNICEF). Cambodia Violence Against Children Survey 2013. Washington, DC, United States of America: Together for Girls.
19274	Cameroon Demographic and Health Survey 2011	Cameroon	ICF International, Ministry of Economy, Planning and Regional Development (Cameroon), Ministry of Public Health (Cameroon), National Institute of Statistics (Cameroon), Pasteur Center of Cameroon. Cameroon Demographic and Health Survey 2011. Fairfax, United States of America: ICF International.
413167	Cameroon Demographic and Health Survey 2018-2019	Cameroon	ICF International, Ministry of Public Health (Cameroon), National Institute of Statistics (Cameroon). Cameroon Demographic and Health Survey 2018-2019. Fairfax, United States of America: ICF International.
19167	Cambodia Demographic and Health Survey 2005-2006	Cambodia	Macro International, Inc, National Institute of Public Health (Cambodia), National Institute of Statistics (Cambodia). Cambodia Demographic and Health Survey 2005-2006. Fairfax, United States of America: ICF International.
19211	Cameroon Demographic and Health Survey 2004	Cameroon	Macro International, Inc, National Institute of Statistics (Cameroon). Cameroon Demographic and Health Survey 2004. Fairfax, United States of America: ICF International.
358376	Cameroon Population-Based HIV Impact Assessment 2017-2018	Cameroon	Centers for Disease Control and Prevention (CDC), Global Health System Solutions (GHSS) (Cameroon), ICAP, Columbia University Mailman School of Public Health, International Reference Center ""Chantal Biya"" (CIRCB) (Cameroon), Ministry of Public Health (Cameroon), National AIDS Control Committee (CNLS) (Cameroon), National Institute of Statistics (Cameroon), National Public Health Laboratory (Cameroon), Pasteur Center of Cameroon. Cameroon Population-Based HIV Impact Assessment 2017-2018. New York, New York: ICAP, Columbia University Mailman School of Public Health, 2021.
503522	Cambodia United Nations Multi-country Study on	Cambodia	Partners for Prevention, United Nations Development Programme (UNDP), United Nations Entity for Gender Equality and the

	Men and Violence 2012 - Equimundo		Empowerment of Women (UN Women), United Nations Population Fund (UNFPA), United Nations Volunteers (UNV). Cambodia United Nations Multi-country Study on Men and Violence 2012 - Equimundo.
523643	Cambodia Demographic and Health Survey 2021-2022	Cambodia	ICF International, Ministry of Health (Cambodia), National Institute of Statistics (Cambodia). Cambodia Demographic and Health Survey 2021-2022. Fairfax, United States of America: ICF International, 2023.
157025	Chad Demographic and Health Survey 2014-2015	Chad	ICF International, National Institute for Statistics, Economic and Demographic Studies (INSEED) (Chad). Chad Demographic and Health Survey 2014-2015. Fairfax, United States of America: ICF International, 2016.
2223	Central African Republic Multiple Indicator Cluster Survey 2006	Central African Republic	United Nations Children's Fund (UNICEF). Central African Republic Multiple Indicator Cluster Survey 2006. New York, United States: United Nations Children's Fund (UNICEF).
169720	Canada Gender, Alcohol and Culture: An International Study (GENACIS) 2004	Canada	Aarhus University, Addiction Switzerland Research Institute, Alcohol Research Group, Public Health Institute, Centre for Addiction and Mental Health (Canada), Centre for Alcohol Policy Research, Turning Point Alcohol and Drug Centre (Australia), Kettil Bruun Society for Social and Epidemiological Research on Alcohol, University of North Dakota. Canada Gender, Alcohol and Culture: An International Study (GENACIS) 2004.
137193	Child abuse and AIDS-related knowledge, attitudes and behavior among adolescents in Zambia	Zambia	Slonim-Nevo V, Mukuka L. Child abuse and AIDS-related knowledge, attitudes and behavior among adolescents in Zambia. Child Abuse Negl. 2007; 31(2): 143-59.
137217	Canada General Social Survey on Victimization 2014	Canada	Statistics Canada (StatCan). Canada General Social Survey on Victimization 2014. 2015.
524155	Cape Verde Demographic and Reproductive Health Survey 2018	Cabo Verde	National Institute of Statistics (Cape Verde), Utica International. Cape Verde Demographic and Reproductive Health Survey 2018. Praia, Cabo Verde: National Institute of Statistics (Cape Verde), 2020.
137139	Child sexual abuse: A study of prevalence in Great Britain	United Kingdom	Baker AW, Duncan SP. Child sexual abuse: A study of prevalence in Great Britain. Child Abuse Negl. 1985; 9(4): 457-67.
137142	Child sexual abuse in southern Brazil and associated factors: a population-based study	Brazil	Bassani DG, Palazzo LS, Béria JU, Gigante LP, Figueiredo AC, Aerts DR, Raymann BC. Child sexual abuse in southern Brazil and associated factors: a population-based study. BMC Public Health. 2009; 9(1): 133.
137156	Children as Victims of Violence: A National Survey	United States of America	Finkelhor D, Dziuba-Leatherman J. Children as Victims of Violence: A National Survey. Pediatrics. 1994; 94(4): 413-20.

137181	Childhood Abuse as a Risk Factor for Adolescent Pregnancy in El Salvador	El Salvador	Pallitto CC, Murillo V. Childhood Abuse as a Risk Factor for Adolescent Pregnancy in El Salvador. J Adolesc Health. 2008; 42(6): 580-6.
137199	Child sexual abuse and persistence of risky sexual behaviors and negative sexual outcomes over adulthood: Findings from a birth cohort	New Zealand	Van Roode T, Dickson N, Herbison P, Paul C. Child sexual abuse and persistence of risky sexual behaviors and negative sexual outcomes over adulthood: Findings from a birth cohort. Child Abuse Negl. 2009; 33(3): 161-72.
137202	Child sexual abuse and its outcomes among high school students in southwest Ethiopia	Ethiopia	Worku D, Gebremariam A, Jayalakshmi S. Child sexual abuse and its outcomes among high school students in southwest Ethiopia. Trop Doct. 2006; 36(3): 137-40.
139804	Child sexual abuse revisited: a population-based cross-sectional study among swiss adolescents	Switzerland	Mohler-Kuo M, Landolt MA, Maier T, Meidert U, Schönbucher V, Schnyder U. Child sexual abuse revisited: a population-based cross-sectional study among swiss adolescents. J Adolesc Health. 2014; 54(3): 304?311e1.
148641	Childhood Sexual Abuse, An Investigation among 239 Male High School Students	China	Chen J, Dunne M, Wang X. Childhood Sexual Abuse, An Investigation among 239 Male High School Students. Chin Ment Health J. 2003; 17(5): 345?7.
148643	Child sexual abuse in China: a study of adolescents in four provinces	China	Chen J, Dunne MP, Han P. Child sexual abuse in China: a study of adolescents in four provinces. Child Abuse Negl. 2004; 28(11): 1171?86.
163098	Childhood maltreatment and early alcohol use among high-risk adolescents	United States of America	Hamburger ME, Leeb RT, Swahn MH. Childhood maltreatment and early alcohol use among high-risk adolescents. J Stud Alcohol Drugs. 2008; 69(2): 291?5.
163100	Childhood abuse and lifetime psychopathology in a community sample	Canada	MacMillan HL, Fleming JE, Streiner DL, Lin E, Boyle MH, Jamieson E, Duku EK, Walsh CA, Wong MY, Beardslee WR. Childhood abuse and lifetime psychopathology in a community sample. Am J Psychiatry. 2001; 158(11): 1878?83.
163103	Childhood sexual abuse and women?s substance abuse: national survey findings	United States of America	Wilsnack SC, Vogeltanz ND, Klassen AD, Harris TR. Childhood sexual abuse and women?s substance abuse: national survey findings. J Stud Alcohol. 1997; 58(3): 264?71.
283129	Childhood sexual abuse experiences and its associated factors among adolescent female high school students in Arbaminch town, Gammo Goffa zone, Southern Ethiopia: a mixed method study	Ethiopia	Mekuria A, Nigussie A, Abera M. Childhood sexual abuse experiences and its associated factors among adolescent female high school students in Arbaminch town, Gammo Goffa zone, Southern Ethiopia: a mixed method study. BMC Int Health Hum Rights. 2015; 15: 21.
31903	China Health and Family Life Survey 1999-2000	China	National Opinion Research Center, University of Chicago (NORC), Renmin University, Beijing, Peking Union Medical College, Beijing, University of North Carolina. China

			Health and Family Life Survey 1999-2000. Chicago, USA: Population Research Center, University of Chicago.
19324	Colombia Demographic and Health Survey 2004-2005	Colombia	Macro International, Inc, Profamilia (Colombia). Colombia Demographic and Health Survey 2004-2005. Fairfax, United States of America: ICF International, 2005.
21281	Colombia Demographic and Health Survey 2009-2010	Colombia	ICF Macro, Profamilia (Colombia). Colombia Demographic and Health Survey 2009-2010. Fairfax, United States of America: ICF International, 2011.
218566	Colombia Demographic and Health Survey 2015-2016	Colombia	ICF International, Ministry of Health (Colombia), Profamilia (Colombia). Colombia Demographic and Health Survey 2015-2016. Fairfax, United States of America: ICF International, 2017.
19359	Colombia Demographic and Health Survey 2000	Colombia	Macro International, Inc, Profamilia (Colombia). Colombia Demographic and Health Survey 2000. Fairfax, United States of America: ICF International, 2000.
126421	China Optimus Study 2009-2010	China	UBS Optimus Foundation, University of Hong Kong. China Optimus Study 2009-2010.
437431	Chile National Survey of Victimization for Domestic Violence and Sexual Crimes 2008	Chile	Center for the Study and Analysis of Crime (CEAD) (Chile), Directorate of Social Studies UC (DESUC), Pontifical Catholic University of Chile (Chile). Chile National Survey of Victimization for Domestic Violence and Sexual Crimes 2008. Chile: Center for the Study and Analysis of Crime (CEAD) (Chile).
437432	Chile National Survey of Victimization for Domestic Violence and Sexual Crimes 2012	Chile	Center for the Study and Analysis of Crime (CEAD) (Chile), GfK Adimark (Chile). Chile National Survey of Victimization for Domestic Violence and Sexual Crimes 2012. Chile: Center for the Study and Analysis of Crime (CEAD) (Chile).
504539	Chile International Men and Gender Equality Study 2009	Chile	EME Masculinities and Gender Equity (Chile), International Center for Research on Women, Promundo. Chile International Men and Gender Equality Study 2009.
76850	Comoros Demographic and Health Survey 2012-2013	Comoros	General Directorate of Statistics and Forecasting (Comoros), ICF International. Comoros Demographic and Health Survey 2012-2013. Fairfax, United States of America: ICF International.
465288	Colombia Violence Against Children and Youth Survey 2018	Colombia	Centers for Disease Control and Prevention (CDC), International Organization for Migration (IOM), Ministry of Health and Social Protection (Colombia), Together for Girls. Colombia Violence Against Children and Youth Survey 2018. Bogotá, Colombia: Ministry of Health and Social Protection (Colombia).
541281	Comoros Multiple Indicator Cluster Survey 2022	Comoros	National Institute of Statistics and Economic and Demographic Studies (INSEED)

			(Comoros), United Nations Children's Fund (UNICEF). Comoros Multiple Indicator Cluster Survey 2022. New York, United States of America: United Nations Children's Fund (UNICEF), 2023.
56148	Côte d'Ivoire AIDS Indicator Survey 2005	Côte d'Ivoire	CDC Retro-CI, Ministry of the Fight Against AIDS (Côte d'Ivoire), National Institute of Statistics (Côte d'Ivoire), ORC Macro. Côte d'Ivoire AIDS Indicator Survey 2005. Fairfax, United States of America: ICF International.
169723	Costa Rica Gender, Alcohol and Culture: An International Study (GENACIS) 2003	Costa Rica	Aarhus University, Addiction Switzerland Research Institute, Alcohol Research Group, Public Health Institute, Centre for Addiction and Mental Health (Canada), Centre for Alcohol Policy Research, Turning Point Alcohol and Drug Centre (Australia), Kettil Bruun Society for Social and Epidemiological Research on Alcohol, University of North Dakota. Costa Rica Gender, Alcohol and Culture: An International Study (GENACIS) 2003.
126435	Croatia Balkan Epidemiological Study on Child Abuse and Neglect 2011	Croatia	Institute of Child Health (Greece), University of Zagreb. Croatia Balkan Epidemiological Study on Child Abuse and Neglect 2011.
465293	Côte d?Ivoire Violence Against Children and Youth Survey 2018	Côte d'Ivoire	Centers for Disease Control and Prevention (CDC), Ministry of Women, Family and Children (Côte d?Ivoire), National Institute of Statistics (Côte d'Ivoire), National Program for the Care of Orphans and Other Children Made Vulnerable by HIV/AIDS (PNOEV) (Côte d?Ivoire), Together for Girls. Côte d?Ivoire Violence Against Children and Youth Survey 2018. Côte d'Ivoire: Ministry of Women, Family and Children (Côte d?Ivoire).
500885	Cook Islands Family Health and Safety Study 2012-2013	New Zealand	Cook Islands Statistics Office, Ministry of Health (Cook Islands), National Council of Women (Cook Islands). Cook Islands Family Health and Safety Study 2012-2013. 2014.
535004	Côte d'Ivoire Demographic and Health Survey 2021	Côte d'Ivoire	ICF International, Ministry of Health and Public Hygiene (MSHP) (Côte d'Ivoire), National Institute of Statistics (Côte d'Ivoire). Côte d'Ivoire Demographic and Health Survey 2021. Fairfax, United States of America: ICF International, 2023.
169721	Czech Republic Gender, Alcohol and Culture: An International Study (GENACIS) 2002	Czechia	Aarhus University, Addiction Switzerland Research Institute, Alcohol Research Group, Public Health Institute, Centre for Addiction and Mental Health (Canada), Centre for Alcohol Policy Research, Turning Point Alcohol and Drug Centre (Australia), Kettil Bruun Society for Social and Epidemiological Research on Alcohol, University of North Dakota. Czech Republic Gender, Alcohol and

			Culture: An International Study (GENACIS) 2002.
259194	Denmark Youth Welfare Survey 2002	Denmark	National Institute of Public Health (Denmark). Denmark Youth Welfare Survey 2002.
259197	Denmark Youth Welfare Survey 2008	Denmark	National Institute of Public Health (Denmark), University of Copenhagen, University of Southern Denmark. Denmark Youth Welfare Survey 2008.
19381	Democratic Republic of the Congo Demographic and Health Survey 2007	Democratic Republic of the Congo	Macro International, Inc, Ministry of Planning (Congo, DR). Democratic Republic of the Congo Demographic and Health Survey 2007. Fairfax, United States of America: ICF International.
76878	Democratic Republic of the Congo Demographic and Health Survey 2013-2014	Democratic Republic of the Congo	ICF International, Ministry of Planning and Monitoring Implementation of the Revolution of Modernity (Congo, DR), Ministry of Public Health (Congo, DR), National Institute of Statistics (Congo, DR). Democratic Republic of the Congo Demographic and Health Survey 2013-2014. Fairfax, United States of America: ICF International, 2014.
137280	Dating violence against adolescent girls and associated substance use, unhealthy weight control, sexual risk behavior, pregnancy, and suicidality	United States of America	Silverman JG, Raj A, Mucci LA, Hathaway JE. Dating violence against adolescent girls and associated substance use, unhealthy weight control, sexual risk behavior, pregnancy, and suicidality. JAMA. 2001; 286(5): 572-9.
137143	Descriptive Epidemiology of Chronic Childhood Adversity in Mexican Adolescents	Mexico	Benjet C, Borges G, Medina-Mora ME, Zambrano J, Cruz C, Méndez E. Descriptive Epidemiology of Chronic Childhood Adversity in Mexican Adolescents. J Adolesc Health. 2009; 45(5): 483-9.
137194	Dimensions of child sexual abuse before age 15 in three Central American countries: Honduras, El Salvador, and Guatemala	El Salvador; Guatemala; Honduras	Speizer IS, Goodwin M, Whittle L, Clyde M, Rogers J. Dimensions of child sexual abuse before age 15 in three Central American countries: Honduras, El Salvador, and Guatemala. Child Abuse Negl. 2008; 32(4): 455-62.
77819	Dominican Republic Demographic and Health Survey 2013	Dominican Republic	Center for Social and Demographic Studies (Dominican Republic) (CESDEM), ICF International, Ministry of Public Health and Social Assistance (Dominican Republic). Dominican Republic Demographic and Health Survey 2013. Fairfax, United States of America: ICF International, 2014.
153700	Ecuador National Survey of Family Relationships and Gender Violence Against Women 2011	Ecuador	Ministry of the Interior (Ecuador), National Institute of Statistics and Censuses (Ecuador), Transition Committee to the Council on Women and Gender Equality (Ecuador). Ecuador National Survey of Family Relationships and Gender Violence Against Women 2011.
19444	Dominican Republic Demographic and Health Survey 2002	Dominican Republic	Center for Social and Demographic Studies (Dominican Republic) (CESDEM), Macro International, Inc. Dominican Republic

			Demographic and Health Survey 2002. Fairfax, United States of America: ICF International.
19456	Dominican Republic Demographic and Health Survey 2007	Dominican Republic	Center for Social and Demographic Studies (Dominican Republic) (CESDEM), Macro International, Inc. Dominican Republic Demographic and Health Survey 2007. Fairfax, United States of America: ICF International.
27621	Ecuador Reproductive Health Survey 1999	Ecuador	Center for Studies of Population and Social Development (CEPAR) (Ecuador), Division of Reproductive Health-Centers for Disease Control and Prevention (CDC). Ecuador Reproductive Health Survey 1999. Atlanta, United States: Centers for Disease Control and Prevention (CDC), 2001.
19431	Dominican Republic Experimental Demographic and Health Survey 1999	Dominican Republic	Center for Social and Demographic Studies (Dominican Republic) (CESDEM), Macro International, Inc. Dominican Republic Experimental Demographic and Health Survey 1999. Fairfax, United States of America: ICF International.
27630	Ecuador Reproductive Health Survey 2004	Ecuador	Center for Studies of Population and Social Development (CEPAR) (Ecuador) and Division of Reproductive Health-Centers for Disease Control and Prevention (CDC). (2005) Ecuador Reproductive Health Survey 2004. Quito, Ecuador: CEPAR.
408852	El Salvador Violence Against Children, Girls and Adolescents Survey 2017	El Salvador	Centers for Disease Control and Prevention (CDC), General Directorate of Statistics and Census (DIGESTYC) (El Salvador), International Organization for Migration (IOM), Ministry of Justice and Public Safety (El Salvador), Together for Girls. El Salvador Violence Against Children, Girls and Adolescents Survey 2017.
27606	El Salvador Reproductive Health Survey 2008	El Salvador	Asociación Demográfica Salvadoreña (ADS), Division of Reproductive Health-Centers for Disease Control and Prevention (CDC). (2009) El Salvador Reproductive Health Survey 2008. San Salvador, El Salvador: ADS.
27599	El Salvador Reproductive Health Survey 2002-2003	El Salvador	Asociación Demográfica Salvadoreña (ADS), Division of Reproductive Health-Centers for Disease Control and Prevention (CDC). (2004) El Salvador Reproductive Health Survey 2002-2003. San Salvador, El Salvador: ADS.
506323	Egypt Economic Gender-Based Violence Survey 2015	Egypt	Central Agency for Public Mobilization and Statistics (CAPMAS) (Egypt), United Nations Population Fund (UNFPA). Egypt Economic Gender-Based Violence Survey 2015.
506722	El Salvador Violence Against Women Study 2014	El Salvador	Pan American Health Organization (PAHO), Technological University of El Salvador, University of Granada (Spain), World Health

			Organization (WHO). El Salvador Violence Against Women Study 2014.
218568	Ethiopia Demographic and Health Survey 2016	Ethiopia	Central Statistics Agency (Ethiopia), ICF International. Ethiopia Demographic and Health Survey 2016. Fairfax, United States of America: ICF International, 2017.
135626	European Union Violence Against Women Study 2012	Austria; Belgium; Bulgaria; Croatia; Cyprus; Czechia; Denmark; Estonia; Finland; France; Germany; Greece; Hungary; Ireland; Italy; Latvia; Lithuania; Luxembourg; Malta; Netherlands; Poland; Portugal; Romania; Slovakia; Slovenia; Spain; Sweden; United Kingdom	European Union Agency for Fundamental Rights. European Union Violence Against Women Study 2012.
272644	Ethiopia STEPS Noncommunicable Disease Risk Factors Survey 2015	Ethiopia	Ethiopian Public Health Institute (EPHI), World Health Organization (WHO). Ethiopia STEPS Noncommunicable Disease Risk Factors Survey 2015.
137165	Exploring the associations between intimate partner violence and women's mental health: Evidence from a population-based study in Paraguay	Paraguay	Ishida K, Stupp P, Melian M, Serbanescu F, Goodwin M. Exploring the associations between intimate partner violence and women's mental health: Evidence from a population-based study in Paraguay. Soc Sci Med. 2010; 71(9): 1653-61.
137264	Experiences with sexual aggression within the general population in the Czech Republic	Czechia	Weiss P, Zverina J. Experiences with sexual aggression within the general population in the Czech Republic. Arch Sex Behav. 1999; 28(3): 265-9.
414568	Ethiopia Population-Based HIV Impact Assessment 2017-2018	Ethiopia	Centers for Disease Control and Prevention (CDC), Central Statistics Agency (Ethiopia), Ethiopian Public Health Institute (EPHI), Government of Ethiopia, ICAP, Columbia University Mailman School of Public Health, Ministry of Health (Ethiopia). Ethiopia Population-Based HIV Impact Assessment 2017-2018. New York, New York: ICAP, Columbia University Mailman School of Public Health, 2021.
500887	Federated States of Micronesia Family Health and Safety Study 2014	Micronesia (Federated States of)	Department of Health and Social Affairs (Micronesia). Federated States of Micronesia Family Health and Safety Study 2014. 2014.
519436	Eswatini Violence Against Children and Youth Survey 2021	Eswatini	Centers for Disease Control and Prevention (CDC), Central Statistical Office (Eswatini), ICAP, Columbia University Mailman School of Public Health, Ministry of Health (Eswatini), President's Emergency Plan for

			AIDS Relief (PEPFAR). Eswatini Violence Against Children and Youth Survey 2021.
543406	Eswatini Multiple Indicator Cluster Survey 2021-2022	Eswatini	Central Statistical Office (Eswatini), United Nations Children's Fund (UNICEF). Eswatini Multiple Indicator Cluster Survey 2021-2022. 2023.
474268	Finland Sex Survey 1991-1992	Finland	Haavio-Mannila, Elina (University of Helsinki) & Kontula, Osmo (University of Helsinki): Finnish Sex Survey 1992 [dataset]. Version 2.0 (2018-08-10). Finnish Social Science Data Archive [distributor]. http://urn.fi/urn:nbn:fi:fsd:T-FSD1243
506328	Fiji National Research on Women's Health and Life Experiences 2010-2011	Fiji	Fiji Women's Crisis Centre. Fiji National Research on Women's Health and Life Experiences 2010-2011.
27494	Georgia Reproductive Health Survey 2005	Georgia	Georgia Center for Disease Control (NCDC), Georgian Ministry of Labor Health and Social Affairs (MOLHSA), Division of Reproductive Health, Centers for Disease Control and Prevention (CDC). Georgia Reproductive Health Survey 2005. Atlanta, United States: Centers for Disease Control and Prevention (CDC).
27486	Georgia Reproductive Health Survey 1999-2000	Georgia	Georgia Center for Disease Control (NCDC), Georgia Ministry of Labor, Health and Social Affairs (MOLHSA), Division of Reproductive Health-Centers for Disease Control and Prevention (CDC). (2001) Georgia Reproductive Health Survey 1999-2000. Atlanta, United States: Centers for Disease Control and Prevention (CDC).
77384	Gambia Demographic and Health Survey 2013	Gambia	Gambia Bureau of Statistics (GBOS), ICF International, Ministry of Health and Social Welfare (The Gambia). Gambia Demographic and Health Survey 2013. Fairfax, United States of America: ICF International, 2015.
95336	Georgia Reproductive Health Survey 2010-2011	Georgia	Division of Reproductive Health, Centers for Disease Control and Prevention (CDC), Georgia Ministry of Labor, Health and Social Affairs, National Center for Disease Control and Public Health (Georgia), National Statistics Office of Georgia (GeoStat). Georgia Reproductive Health Survey 2010-2011.
76706	Gabon Demographic and Health Survey 2012	Gabon	General Directorate of Statistics (Gabon), ICF International, Ministry of Economy, Employment and Sustainable Development (Gabon), Ministry of Health and Social Affairs (Gabon). Gabon Demographic and Health Survey 2012. Fairfax, United States of America: ICF International, 2013.
137191	Gender and the associated impairments of childhood sexual abuse: A national study of Icelandic youth	Iceland	Gault-Sherman M, Silver E, Sigfúsdóttir ID. Gender and the associated impairments of childhood sexual abuse: A national study of

			Icelandic youth. Soc Sci Med. 2009; 69(10): 1515-22.
150444	Gender, sexual abuse and risk behaviours in adolescents: a cross-sectional survey in schools in Goa	India	Patel V, Andrew G. Gender, sexual abuse and risk behaviours in adolescents: a cross-sectional survey in schools in Goa. Natl Med J India. 2001; 14(5): 263-7.
459854	Gambia Demographic and Health Survey 2019-2020	Gambia	Gambia Bureau of Statistics (GBOS), ICF International, Ministry of Health and Social Welfare (The Gambia). Gambia Demographic and Health Survey 2019-2020. Fairfax, United States of America: ICF International, 2021.
507796	Georgia National Research on Domestic Violence Against Women 2009	Georgia	ACT Research (Georgia), Ivane Javakhishvili Tbilisi State University (Georgia). Georgia National Research on Domestic Violence Against Women 2009.
511588	Georgia International Men and Gender Equality Study 2019	Georgia	National Center for Disease Control and Public Health (Georgia), Promundo, United Nations Development Programme (UNDP), United Nations Entity for Gender Equality and the Empowerment of Women (UN Women), United Nations Population Fund (UNFPA). Georgia International Men and Gender Equality Study 2019.
21188	Ghana Demographic and Health Survey 2008	Ghana	Ghana Statistical Service, Macro International, Inc, Ministry of Health (Ghana). Ghana Demographic and Health Survey 2008. Fairfax, United States of America: ICF International.
126431	Greece Balkan Epidemiological Study on Child Abuse and Neglect 2010-2012	Greece	Institute of Child Health (Greece). Greece Balkan Epidemiological Study on Child Abuse and Neglect 2010-2012.
4779	Guatemala Reproductive Health Survey 2008-2009	Guatemala	Guatemala Ministry of Health and Social Assistance, University of Valle and Division of Reproductive Health-Centers for Disease Control and Prevention (CDC). Guatemala Reproductive Health Survey 2008-2009. Atlanta, United States: Centers for Disease Control and Prevention (CDC).
157031	Guatemala Demographic and Health Survey 2014-2015	Guatemala	ICF International, Institute of Nutrition of Central America and Panama, Ministry of Public Health and Social Assistance (Guatemala), National Statistics Institute (Guatemala), Secretary of Planning and Programming of the Presidency (Segeplán) (Guatemala). Guatemala Demographic and Health Survey 2014-2015. Fairfax, United States of America: ICF International, 2017.
27563	Guatemala Reproductive Health Survey 2002	Guatemala	Guatemala Ministry of Health and Social Assistance, University of Valle, Division of Reproductive Health-Centers for Disease Control and Prevention (CDC). (2003) Guatemala Reproductive Health Survey 2002.

			Atlanta, United States: Centers for Disease Control and Prevention (CDC).
259184	Greenland Youth Welfare 2011	Greenland	Department of Family, Culture, Church and Gender Equality (Greenland), National Institute of Public Health (Denmark). Greenland Youth Welfare 2011.
259190	Greenland Youth Welfare 2004-2005	Greenland	National Institute of Public Health (Denmark). Greenland Youth Welfare 2004-2005.
474478	Grenada Women's Health and Life Experiences Survey 2018	Grenada	Caribbean Development Bank, Central Statistical Office (Grenada), Global Women?s Institute, George Washington University, United Nations Entity for Gender Equality and the Empowerment of Women (UN Women). Grenada Women's Health and Life Experiences Survey 2018.
19720	Haiti Demographic and Health Survey 2005-2006	Haiti	Haitian Institute of Childhood (IHE), Haitian Institute of Statistics and Informatics, Macro International, Inc. Haiti Demographic and Health Survey 2005-2006. Fairfax, United States of America: ICF International.
408933	Honduras Violence Against Children, Girls and Adolescents Survey 2017	Honduras	Centers for Disease Control and Prevention (CDC), International Organization for Migration (IOM), Ministry of Security (Honduras), Together for Girls, United Nations Children's Fund (UNICEF). Honduras Violence Against Children, Girls and Adolescents Survey 2017.
19728	Honduras Demographic and Health Survey 2005-2006	Honduras	Macro International, Inc, National Institute of Statistics (Honduras), Secretary of Health (Honduras). Honduras Demographic and Health Survey 2005-2006. Fairfax, United States of America: ICF International.
65118	Haiti Demographic and Health Survey 2012	Haiti	Centers for Disease Control and Prevention (CDC), Haitian Institute of Childhood (IHE), Haitian Institute of Statistics and Informatics, Macro International, Inc. Haiti Demographic and Health Survey 2012. Fairfax, United States of America: ICF International.
95440	Honduras Demographic and Health Survey 2011-2012	Honduras	ICF Macro, National Institute of Statistics (Honduras). Honduras Demographic and Health Survey 2011-2012. Fairfax, United States of America: ICF International.
27551	Honduras Reproductive Health Survey 2001	Honduras	Honduras Family Planning Association (ASHONPLAFA), Ministry of Health (Honduras), and Division of Reproductive Health-Centers for Disease Control and Prevention (CDC). Honduras Reproductive Health Survey 2001. Tegucigalpa, Honduras: Honduras Family Planning Association (ASHONPLAFA).
231763	Haiti Violence Against Children Survey 2012	Haiti	Centers for Disease Control and Prevention (CDC), Government of Haiti, Interuniversity Institute for Research and Development (INURED), Multi-Sector Coordination Committee (CCMHAITI) (Haiti), Together

			for Girls. Haiti Violence Against Children Survey 2012. Washington, DC, United States of America: Together for Girls.
19708	Haiti Demographic and Health Survey 2000	Haiti	Haitian Institute of Childhood (IHE), Macro International, Inc. Haiti Demographic and Health Survey 2000. Fairfax, United States of America: ICF International.
27215	Guinea-Bissau Multiple Indicator Cluster Survey 2010	Guinea-Bissau	Centers for Disease Control and Prevention (CDC), National Statistics Institute (Guinea-Bissau), United Nations Children's Fund (UNICEF). Guinea-Bissau Multiple Indicator Cluster Survey 2010. New York, United States of America: United Nations Children's Fund (UNICEF), 2018.
137192	Health status and health care use of Massachusetts women reporting partner abuse	United States of America	Hathaway JE, Mucci LA, Silverman JG, Brooks DR, Mathews R, Pavlos CA. Health status and health care use of Massachusetts women reporting partner abuse. Am J Prev Med. 2000; 19(4): 302-7.
218574	Haiti Demographic and Health Survey 2016-2017	Haiti	Haitian Institute of Childhood (IHE), Haitian Institute of Statistics and Informatics, ICF International, Ministry of Public Health and Population (Haiti). Haiti Demographic and Health Survey 2016-2017. Fairfax, United States of America: ICF International.
490966	Honduras Multiple Indicator Cluster Survey 2019	Honduras	European Union (EU), Government of Canada, Government of Honduras, National Institute of Statistics (Honduras), Secretary of Health (Honduras), United Nations Children's Fund (UNICEF), United Nations Population Fund (UNFPA). Honduras Multiple Indicator Cluster Survey 2019. New York, United States of America: United Nations Children's Fund (UNICEF), 2021.
19963	India Demographic and Health Survey 2005-2006	India	International Institute for Population Sciences (India), Macro International, Inc. India Demographic and Health Survey 2005-2006. Fairfax, United States of America: ICF International.
157050	India Demographic and Health Survey 2015-2016	India	ICF International, International Institute for Population Sciences (India), Ministry of Health and Family Welfare (India). India Demographic and Health Survey 2015-2016. Fairfax, United States of America: ICF International, 2018.
169730	India - Karnataka Gender, Alcohol and Culture: An International Study (GENACIS) 2003	India	Aarhus University, Addiction Switzerland Research Institute, Alcohol Research Group, Public Health Institute, Centre for Addiction and Mental Health (Canada), Centre for Alcohol Policy Research, Turning Point Alcohol and Drug Centre (Australia), Kettil Bruun Society for Social and Epidemiological Research on Alcohol, University of North Dakota. India - Karnataka Gender, Alcohol

			and Culture: An International Study (GENACIS) 2003.
166274	Iceland Youth Survey 2004	Iceland	Icelandic Centre for Social Research and Analysis (ICSRA). Iceland Youth Survey 2004.
166277	Iceland Youth Survey 2007	Iceland	Icelandic Centre for Social Research and Analysis (ICSRA). Iceland Youth Survey 2007.
166279	Iceland Youth Survey 2009	Iceland	Icelandic Centre for Social Research and Analysis (ICSRA). Iceland Youth Survey 2009.
166283	Iceland Youth Survey 2013	Iceland	Icelandic Centre for Social Research and Analysis (ICSRA). Iceland Youth Survey 2013.
166273	Iceland Youth Survey 2003	Iceland	Icelandic Centre for Social Research and Analysis (ICSRA). Iceland Youth Survey 2003.
166276	Iceland Youth Survey 2006	Iceland	Icelandic Centre for Social Research and Analysis (ICSRA). Iceland Youth Survey 2006.
166280	Iceland Youth Survey 2010	Iceland	Icelandic Centre for Social Research and Analysis (ICSRA). Iceland Youth Survey 2010.
166282	Iceland Youth Survey 2012	Iceland	Icelandic Centre for Social Research and Analysis (ICSRA). Iceland Youth Survey 2012.
166284	Iceland Youth Survey 2014	Iceland	Icelandic Centre for Social Research and Analysis (ICSRA). Iceland Youth Survey 2014.
501406	India - Bihar and Uttar Pradesh Understanding the Lives of Adolescents and Young Adults 2015-2016, Wave 1	India	Population Council (India). India - Bihar and Uttar Pradesh Understanding the Lives of Adolescents and Young Adults 2015-2016, Wave 1. New Delhi, India: Population Council (India), 2017.
506335	Indonesia - Papua and West Papua Study on Women's and Men's Life Experiences 2016	Indonesia	Rifka Annisa Women's Crisis Center (Indonesia), Statistics Indonesia, United Nations Development Programme (UNDP), United States Agency for International Development (USAID). Indonesia - Papua and West Papua Study on Women's and Men's Life Experiences 2016.
512518	India Masculinity, Intimate Partner Violence and Son Preference 2014	India	International Center for Research on Women, United Nations Population Fund (UNFPA). India Masculinity, Intimate Partner Violence and Son Preference 2014.
137189	Intimate partner violence and health outcomes in mid-life women: a population-based cohort study	Australia	Schei B, Guthrie JR, Dennerstein L, Alford S. Intimate partner violence and health outcomes in mid-life women: a population-based cohort study. Arch Womens Ment Health. 2006; 9(6): 317-24.
150747	International Violence Against Women Surveys Data 2002-2005	Australia; China; Costa Rica; Czechia; Denmark; Mozambique;	European Institute for Crime Prevention and Control, affiliated with the United Nations (HEUNI), United Nations Office on Drugs and Crime (UNODC), Statistics Canada, United Nations Interregional Crime and

		Philippines; Poland; Switzerland	Justice Research Institute (UNICRI). International Violence Against Women Surveys (IVAWS) Data 2002-2005. As provided by the Global Burden of Disease Child Sexual Abuse and Intimate Partner Violence Expert Group. [Unpublished].
503521	Indonesia United Nations Multi-country Study on Men and Violence 2012 - Equimundo	Indonesia	Partners for Prevention, Rifka Annisa Women's Crisis Center (Indonesia), United Nations Development Programme (UNDP), United Nations Entity for Gender Equality and the Empowerment of Women (UN Women), United Nations Population Fund (UNFPA), United Nations Volunteers (UNV). Indonesia United Nations Multi-country Study on Men and Violence 2012 - Equimundo.
273012	Ireland Sexual Abuse and Violence Study 2001	Ireland	Department of Health and Children (Ireland), Department of Justice, Equality and Law Reform (Ireland), Dublin Rape Crisis Center, Royal College of Surgeons in Ireland (RCSI). Ireland Sexual Abuse and Violence Study 2001.
137152	Is child sexual abuse declining? Evidence from a population-based survey of men and women in Australia	Australia	Dunne MP, Purdie DM, Cook MD, Boyle FM, Najman JM. Is child sexual abuse declining? Evidence from a population-based survey of men and women in Australia. Child Abuse Negl. 2003; 27(2): 141-52.
7163	Jamaica Reproductive Health Survey 2008-2009	Jamaica	Jamaica Family Planning Board, Jamaica Division of Reproductive Health-Centers for Disease Control and Prevention (CDC). Jamaica Reproductive Health Survey 2008. Atlanta, United States: Centers for Disease Control and Prevention (CDC).
415455	Jamaica Women?s Health Survey 2016	Jamaica	Global Women?s Institute, George Washington University, Statistical Institute of Jamaica. Jamaica Women?s Health Survey 2016. Inter-American Development Bank (IDB), 2018.
7161	Jamaica Reproductive Health Survey 2002-2003	Jamaica	Division of Reproductive Health, Centers for Disease Control and Prevention (CDC), Jamaica National Family Planning Board, Statistical Institute of Jamaica. Jamaica Reproductive Health Survey 2002-2003. Kingston, Jamaica: Derek Gordon Databank, University of the West Indies.
506358	Italy Violence Against Women Survey 2014	Italy	National Institute of Statistics (Italy). Italy Violence Against Women Survey 2014. Rome, Italy: National Institute of Statistics (Italy).
506367	Italy Violence Against Women Survey 2006	Italy	Department for Equal Opportunities (Italy), National Institute of Statistics (Italy). Italy Violence Against Women Survey 2006.
76702	Kazakhstan Multiple Indicator Cluster Survey 2010-2011	Kazakhstan	Agency of the Republic of Kazakhstan on Statistics, United Nations Children's Fund (UNICEF). Kazakhstan Multiple Indicator Cluster Survey 2010-2011. New York, United

			States of America: United Nations Children's Fund (UNICEF), 2013.
169736	Kazakhstan Gender, Alcohol and Culture: An International Study (GENACIS) 2002-2003	Kazakhstan	Aarhus University, Addiction Switzerland Research Institute, Alcohol Research Group, Public Health Institute, Centre for Addiction and Mental Health (Canada), Centre for Alcohol Policy Research, Turning Point Alcohol and Drug Centre (Australia), Kettil Bruun Society for Social and Epidemiological Research on Alcohol, University of North Dakota. Kazakhstan Gender, Alcohol and Culture: An International Study (GENACIS) 2002-2003.
126420	Kenya Violence Against Children Study 2010	Kenya	Centers for Disease Control and Prevention (CDC), Kenya National Bureau of Statistics, United Nations Children's Fund (UNICEF). Kenya Violence Against Children Study 2010. Nairobi, Kenya: Kenya National Bureau of Statistics.
454583	Kenya Violence Against Children Study 2018-2019	Kenya	Centers for Disease Control and Prevention (CDC), Kenya National Bureau of Statistics, Ministry of Labour and Social Protection (Kenya), Population Council, Together for Girls, United Nations Children's Fund (UNICEF). Kenya Violence Against Children Study 2018-2019. Washington, DC, United States of America: Together for Girls.
21365	Kenya Demographic and Health Survey 2008-2009	Kenya	ICF Macro, Kenya Medical Research Institute (KEMRI), Kenya National Bureau of Statistics, Ministry of Public Health and Sanitation (Kenya), National AIDS and STI Control Programme (NASCOP) (Kenya), National Association of County and City Health Officials (NACCHO) (United States), National Coordinating Agency for Population and Development (Kenya). Kenya Demographic and Health Survey 2008-2009. Fairfax, United States of America: ICF International.
157057	Kenya Demographic and Health Survey 2014	Kenya	ICF International, Kenya Medical Research Institute (KEMRI), Kenya National Bureau of Statistics, Ministry of Health (Kenya), National AIDS Control Council (Kenya), National Council for Population and Development (Kenya). Kenya Demographic and Health Survey 2014. Fairfax, United States of America: ICF International.
20145	Kenya Demographic and Health Survey 2003	Kenya	Centers for Disease Control and Prevention (CDC), Central Bureau of Statistics (Kenya), Macro International, Inc, Ministry of Health (Kenya), National Council for Population and Development (Kenya). Kenya Demographic and Health Survey 2003. Fairfax, United States of America: ICF International.

529017	Kenya Demographic and Health Survey 2022	Kenya	ICF International, Kenya National Bureau of Statistics, Ministry of Health (Kenya). Kenya Demographic and Health Survey 2022. Fairfax, United States of America: ICF International, 2023.
126428	Kiribati Family Health and Support Study 2008	Kiribati	Kiribati National Statistics Office, Ministry of Internal and Social Affairs (Kiribati), Secretariat of the Pacific Community (SPC), United Nations Children's Fund (UNICEF), World Health Organization (WHO). Kiribati Family Health and Support Study 2008.
77518	Kyrgyzstan Demographic and Health Survey 2012	Kyrgyzstan	ICF International, Ministry of Health (Kyrgyzstan), National Statistical Committee of the Kyrgyz Republic. Kyrgyzstan Demographic and Health Survey 2012. Fairfax, United States of America: ICF International.
20191	Liberia Demographic and Health Survey 2006-2007	Liberia	Liberia Institute for Statistics and Geo-information Services (LISGIS), Macro International, Inc. Liberia Demographic and Health Survey 2006-2007. Fairfax, United States of America: ICF International.
408930	Laos Violence Against Children Survey 2014	Lao People's Democratic Republic	Centers for Disease Control and Prevention (CDC), Lao Statistics Bureau, Lao Women's Union, Ministry of Labour and Social Welfare (Laos), National Commission for Mothers and Children (Laos), Together for Girls, United Nations Children's Fund (UNICEF). Laos Violence Against Children Survey 2014.
465218	Lesotho Violence Against Children and Youth Survey 2018	Lesotho	Centers for Disease Control and Prevention (CDC), ICAP, Columbia University Mailman School of Public Health, Ministry of Social Development (Lesotho), Together for Girls. Lesotho Violence Against Children and Youth Survey 2018. Lesotho: Ministry of Social Development (Lesotho).
391388	Laos National Survey on Women?s Health and Life Experiences 2013-2014	Lao People's Democratic Republic	Lao Statistics Bureau, Lao Women's Union, Ministry of Health (Laos), Ministry of Justice (Laos), Ministry of Public Security (Laos), National Commission for the Advancement of Women (NCAW) (Laos), United Nations Entity for Gender Equality and the Empowerment of Women (UN Women), United Nations Population Fund (UNFPA), World Health Organization (WHO). Laos National Survey on Women?s Health and Life Experiences 2013-2014.
126430	Macedonia Balkan Epidemiological Study on Child Abuse and Neglect 2011	North Macedonia	Institute of Child Health (Greece), Saints Cyril and Methodius University of Skopje. Macedonia Balkan Epidemiological Study on Child Abuse and Neglect 2011.
137285	Lifetime interpersonal violence and self-reported chlamydia trachomatis	United States of America	Alvarez J, Pavao J, Mack KP, Chow JM, Baumrind N, Kimerling R. Lifetime interpersonal violence and self-reported chlamydia trachomatis diagnosis among

	diagnosis among California women		California women. J Womens Health (Larchmt). 2009; 18(1): 57-63.
21393	Malawi Demographic and Health Survey 2010	Malawi	ICF Macro, National Statistical Office of Malawi. Malawi Demographic and Health Survey 2010. Fairfax, United States of America: ICF International.
218581	Malawi Demographic and Health Survey 2015-2016	Malawi	Emory University and Centers for Disease Control & Prevention Collaboration, ICF International, Ministry of Health (Malawi), National Statistical Office of Malawi. Malawi Demographic and Health Survey 2015-2016. Fairfax, United States of America: ICF International, 2017.
231758	Malawi Violence Against Children and Young Women Survey 2013	Malawi	Center for Social Research, University of Malawi, Centers for Disease Control and Prevention (CDC), Ministry of Gender, Children, Disability and Social Welfare (Malawi), President's Emergency Plan for AIDS Relief (PEPFAR), Together for Girls, United Nations Children's Fund (UNICEF). Malawi Violence Against Children and Young Women Survey 2013. Washington, DC, United States of America: Together for Girls.
20263	Malawi Demographic and Health Survey 2004-2005	Malawi	Macro International, Inc, National Statistical Office of Malawi. Malawi Demographic and Health Survey 2004-2005. Fairfax, United States of America: ICF International.
287629	Malawi Population-Based HIV Impact Assessment 2015-2016	Malawi	Centers for Disease Control and Prevention (CDC), ICAP, Columbia University Mailman School of Public Health, Johns Hopkins University, Ministry of Health (Malawi), Statistical Center for HIV/AIDS Research and Prevention (SCHARP), University of Malawi College of Medicine, Westat. Malawi Population-Based HIV Impact Assessment 2015-2016.
542420	Macedonia Survey of Adverse Childhood Experiences Among Young People 2010	North Macedonia	World Health Organization Regional Office for Europe (WHO/Europe). Macedonia Survey of Adverse Childhood Experiences Among Young People 2010.
218582	Maldives Demographic and Health Survey 2016-2017	Maldives	ICF International, Ministry of Health (Maldives). Maldives Demographic and Health Survey 2016-2017. Fairfax, United States of America: ICF International, 2019.
398033	Mali Demographic and Health Survey 2018	Mali	ICF International, National Institute of Statistics (INSTAT) (Mali), Sector Planning and Statistics Unit Health-Social Development and Family Promotion (CPS / SS-DS-PF) (Mali). Mali Demographic and Health Survey 2018. Fairfax, United States of America: ICF International, 2019.
20274	Mali Demographic and Health Survey 2006	Mali	Macro International, Inc, Ministry of Health (Mali), National Directorate of Statistics and Informatics (DNSI) (Mali). Mali

			Demographic and Health Survey 2006. Fairfax, United States of America: ICF International.
77388	Mali Demographic and Health Survey 2012-2013	Mali	ICF International, INFO-STAT (Mali), Ministry of Health (Mali), National Institute of Statistics (INSTAT) (Mali), Planning and Statistics Unit, Ministry of Health (Mali). Mali Demographic and Health Survey 2012-2013. Fairfax, United States of America: ICF International, 2014.
500889	Marshall Islands Family Health and Safety Study 2012	Marshall Islands	Ministry of Internal Affairs (Marshall Islands), Women United Together Marshall Islands (WUTMI). Marshall Islands Family Health and Safety Study 2012.
506383	Maldives Study on Women?s Health and Life Experiences 2006	Maldives	Ministry of Gender, Family and Social Services (Maldives). Maldives Study on Women?s Health and Life Experiences 2006.
506512	Malta Nationwide Research Study on the Prevalence of Domestic Violence Against Women 2010	Malta	Commission on Gender-Based Violence and Domestic Violence (Malta), M. Fsadni & Associates (Malta). Malta Nationwide Research Study on the Prevalence of Domestic Violence Against Women 2010.
137151	Men's and women's childhood sexual abuse and victimization in adult partner relationships: A study of risk factors	Canada	Daigneault I, Hébert M, McDuff P. Men's and women's childhood sexual abuse and victimization in adult partner relationships: A study of risk factors. Child Abuse Negl. 2009; 33(9): 638-47.
137176	Measuring child maltreatment in the United Kingdom: a study of the prevalence of child abuse and neglect	United Kingdom	May-Chahal C, Cawson P. Measuring child maltreatment in the United Kingdom: a study of the prevalence of child abuse and neglect. Child Abuse Negl. 2005; 29(9): 969-84.
188837	Mexico National Survey on Violence Against Women 2002-2003	Mexico	Institute for Social Security and Services for State Workers (ISSSTE) (Mexico), Mexican Social Security Institute (IMSS), National Institute of Public Health (Mexico), Secretariat of Health (Mexico). Mexico National Survey on Violence Against Women 2002-2003.
20339	Moldova Demographic and Health Survey 2005	Republic of Moldova	Macro International, Inc, National Scientific and Applied Center for Preventive Medicine (Moldova). Moldova Demographic and Health Survey 2005. Fairfax, United States of America: ICF International.
432257	Mongolia Gender Based Violence Survey 2017	Mongolia	National Statistical Office of Mongolia. Mongolia Gender Based Violence Survey 2017. Ulaanbaatar, Mongolia: National Statistical Office of Mongolia, 2018.
317752	Mexico National Survey on the Dynamics of Household Relationships 2016	Mexico	National Institute of Statistics and Geography (INEGI) (Mexico), National Institute of Women (Mexico), United Nations Women's Fund (UNIFEM). Mexico National Survey on the Dynamics of Household Relationships 2016. Mexico City, Mexico: National Institute

			of Statistics and Geography (INEGI) (Mexico).
8750	Moldova Reproductive Health Survey 1997	Republic of Moldova	Division of Reproductive Health-Centers for Disease Control and Prevention (CDC) and Moldova Ministry of Health. (1998) Moldova Reproductive Health Survey 1997. Atlanta, United States: Centers for Disease Control and Prevention (CDC).
55975	Mozambique Demographic and Health Survey 2011	Mozambique	ICF Macro, Manhica Health Research Center (CISM), Ministry of Health (Mozambique), National Institute of Statistics (INE) (Mozambique). Mozambique Demographic and Health Survey 2011. Fairfax, United States of America: ICF International.
157060	Mozambique AIDS Indicator Survey 2015	Mozambique	Centers for Disease Control and Prevention (CDC), ICF International, Ministry of Health (Mozambique), National Institute of Health (Mozambique), National Institute of Statistics (INE) (Mozambique). Mozambique AIDS Indicator Survey 2015. Fairfax, United States of America: ICF International, 2018.
27519	Mozambique Young Adult Reproductive Health Survey 2001	Mozambique	Mozambique National Institute of Statistics, Division of Reproductive Health-Centers for Disease Control and Prevention (CDC). (2003) Mozambique Young Adult Reproductive Health Survey 2001. Atlanta, United States: Centers for Disease Control and Prevention (CDC).
474225	Montenegro OSCE-Led Survey on the Well-Being and Safety of Women 2018	Montenegro	Ipsos, Organization for Security and Co-operation in Europe (OSCE). Montenegro OSCE-Led Survey on the Well-Being and Safety of Women 2018. 2019.
497122	Mozambique Violence Against Children and Youth Survey 2019	Mozambique	Centers for Disease Control and Prevention (CDC), Ministry of Gender, Children and Social Action (Mozambique), Ministry of Health (Mozambique), National Institute of Health (Mozambique), National Institute of Statistics (INE) (Mozambique), United Nations Children's Fund (UNICEF). Mozambique Violence Against Children and Youth Survey 2019.
286782	Nepal Demographic and Health Survey 2016-2017	Nepal	ICF International, Ministry of Health (Nepal), New ERA. Nepal Demographic and Health Survey 2016-2017. Fairfax, United States of America: ICF International, 2017.
21240	Nepal Demographic and Health Survey 2011	Nepal	ICF Macro, Ministry of Health and Population (Nepal), New ERA. Nepal Demographic and Health Survey 2011. Fairfax, United States of America: ICF International.
150382	Namibia Demographic and Health Survey 2013	Namibia	ICF International, Ministry of Health and Social Services (Namibia), Namibia Institute of Pathology, Namibia Statistics Agency. Namibia Demographic and Health Survey 2013. Fairfax, United States of America: ICF International.

472783	Nauru Family Health and Support Study 2013	Nauru	Department of Women's Affairs (Nauru), Ministry of Home Affairs (Nauru). Nauru Family Health and Support Study 2013. Nauru: Ministry of Home Affairs (Nauru).
490343	Namibia Violence Against Children and Youth Survey 2019	Namibia	Centers for Disease Control and Prevention (CDC), International Training and Education Center for Health (I-TECH), University of Washington, Ministry of Gender Equality, Poverty Eradication and Social Welfare (Namibia), Namibia Statistics Agency, United Nations Children's Fund (UNICEF). Namibia Violence Against Children and Youth Survey 2019.
506334	National Study on Violence Against Women in Georgia 2017	Georgia	European Union (EU), National Statistics Office of Georgia (GeoStat), United Nations Entity for Gender Equality and the Empowerment of Women (UN Women). National Study on Violence Against Women in Georgia 2017. 2018.
528571	Nepal Demographic and Health Survey 2022	Nepal	ICF International, Ministry of Health and Population (Nepal), New ERA. Nepal Demographic and Health Survey 2022. Fairfax, United States of America: ICF International, 2023.
554207	National Study on Violence Against Women in Georgia 2022	Georgia	European Union (EU), National Statistics Office of Georgia (GeoStat), United Nations Entity for Gender Equality and the Empowerment of Women (UN Women). National Study on Violence Against Women in Georgia 2022. 2023.
169739	New Zealand Gender, Alcohol and Culture: An International Study (GENACIS) 2007	New Zealand	Aarhus University, Addiction Switzerland Research Institute, Alcohol Research Group, Public Health Institute, Centre for Addiction and Mental Health (Canada), Centre for Alcohol Policy Research, Turning Point Alcohol and Drug Centre (Australia), Kettil Bruun Society for Social and Epidemiological Research on Alcohol, University of North Dakota. New Zealand Gender, Alcohol and Culture: An International Study (GENACIS) 2007.
411338	Netherlands Sexual Health Survey 2017	Netherlands	Rutgers Nisso Group. Netherlands Sexual Health Survey 2017.
141635	New Zealand Youth2012 Health and Wellbeing of Secondary School Students 2012	New Zealand	Adolescent Health Research Group, University of Auckland, Auckland UniServices, University of Auckland (New Zealand), Faculty of Medical and Health Sciences, University of Auckland. New Zealand Youth2012 Health and Wellbeing of Secondary School Students 2012.
454488	New Zealand Youth 2019 Rangatahi Smart Survey 2019	New Zealand	University of Auckland (New Zealand), University of Otago (New Zealand), Victoria University of Wellington (New Zealand). New Zealand Youth 2019 Rangatahi Smart Survey 2019.

9270	Nicaragua Reproductive Health Survey 2006-2007	Nicaragua	Division of Reproductive Health, Centers for Disease Control and Prevention (CDC), National Institute for Development Information (Nicaragua). Nicaragua Reproductive Health Survey 2006-2007. Managua, Nicaragua: National Institute for Development Information (Nicaragua).
150424	New Zealand Youth2000 Health and Wellbeing of Secondary School Students 2001	New Zealand	Adolescent Health Research Group, University of Auckland, Faculty of Medical and Health Sciences, University of Auckland. New Zealand Youth2000 Health and Wellbeing of Secondary School Students 2001.
150426	New Zealand Youth2007 Health and Wellbeing of Secondary School Students 2007	New Zealand	Adolescent Health Research Group, University of Auckland, Faculty of Medical and Health Sciences, University of Auckland. New Zealand Youth2007 Health and Wellbeing of Secondary School Students 2007.
169738	Nicaragua Gender, Alcohol and Culture: An International Study (GENACIS) 2005	Nicaragua	Aarhus University, Addiction Switzerland Research Institute, Alcohol Research Group, Public Health Institute, Centre for Addiction and Mental Health (Canada), Centre for Alcohol Policy Research, Turning Point Alcohol and Drug Centre (Australia), Kettil Bruun Society for Social and Epidemiological Research on Alcohol, University of North Dakota. Nicaragua Gender, Alcohol and Culture: An International Study (GENACIS) 2005.
20478	Nicaragua Demographic and Health Survey 1997-1998	Nicaragua	Macro International, Inc, Ministry of Health (Nicaragua), National Institute of Statistics and Censuses (Nicaragua). Nicaragua Demographic and Health Survey 1997-1998. Fairfax, United States of America: ICF International.
126952	Nicaragua National Demographic and Health Survey 2011-2012	Nicaragua	Ministry of Health (Nicaragua), National Institute for Development Information (Nicaragua). Nicaragua National Demographic and Health Survey 2011-2012. Managua, Nicaragua: National Institute for Development Information (Nicaragua).
506911	Niger Extent and Determinants of Gender-Based Violence 2015	Niger	Ministry for the Promotion of Women and the Protection of Children (Niger). Niger Extent and Determinants of Gender-Based Violence 2015.
21433	Nigeria Demographic and Health Survey 2008	Nigeria	Macro International, Inc, National Population Commission of Nigeria. Nigeria Demographic and Health Survey 2008. Fairfax, United States of America: ICF International, 2009.
231760	Nigeria Violence Against Children Survey 2014	Nigeria	Centers for Disease Control and Prevention (CDC), Government of Nigeria, Together for Girls, United Nations Children's Fund (UNICEF). Nigeria Violence Against Children Survey 2014.

408484	Nigeria Demographic and Health Survey 2018	Nigeria	Federal Ministry of Health (Nigeria), ICF International, National Population Commission (NPC). Nigeria Demographic and Health Survey 2018. Fairfax, United States of America: ICF International, 2020.
77390	Nigeria Demographic and Health Survey 2013	Nigeria	ICF International, National Population Commission of Nigeria. Nigeria Demographic and Health Survey 2013. Fairfax, United States of America: ICF International.
169740	Nigeria Gender, Alcohol and Culture: An International Study (GENACIS) 2003	Nigeria	Aarhus University, Addiction Switzerland Research Institute, Alcohol Research Group, Public Health Institute, Centre for Addiction and Mental Health (Canada), Centre for Alcohol Policy Research, Turning Point Alcohol and Drug Centre (Australia), Kettil Bruun Society for Social and Epidemiological Research on Alcohol, University of North Dakota. Nigeria Gender, Alcohol and Culture: An International Study (GENACIS) 2003.
354770	Norway Safety, Violence and Quality of Life Survey 2013	Norway	Norwegian Centre for Violence and Traumatic Stress Studies (NKVTS). Norway Safety, Violence and Quality of Life Survey 2013.
137166	Nonconsensual Sexual Experiences of Adolescents in Urban India	India	Jaya J, Hindin MJ. Nonconsensual Sexual Experiences of Adolescents in Urban India. J Adolesc Health. 2007; 40(6): 573e7?573e.
449727	Norway Violence and Rape: A National Prevalence Study of Violence in a Lifespan Perspective 2013	Norway	Ipsos MMI (Norway), Norwegian Centre for Violence and Traumatic Stress Studies (NKVTS). Norway Violence and Rape: A National Prevalence Study of Violence in a Lifespan Perspective 2013.
512760	Nigeria International Men and Gender Equality Study 2015	Nigeria	Promundo. Nigeria International Men and Gender Equality Study 2015.
500888	Palau Family Health and Safety Study 2013	Palau	Ministry of Health (Palau). Palau Family Health and Safety Study 2013.
426238	Papua New Guinea Demographic and Health Survey 2016-2018	Papua New Guinea	ICF International, National Statistical Office (Papua New Guinea). Papua New Guinea Demographic and Health Survey 2016-2018. Fairfax, United States of America: ICF International, 2019.
10370	Paraguay Reproductive Health Survey 2004	Paraguay	Division of Reproductive Health-Centers for Disease Control and Prevention (CDC). (2005): Paraguay Reproductive Health Survey 2004. Asunción, Paraguay, Paraguayan Center for Population Studies (CEPEP).
357074	Pathways between childhood trauma, intimate partner violence, and harsh parenting: findings from the UN Multi-country Study on Men and Violence in Asia and the Pacific	Bangladesh; Cambodia; China; Indonesia; Papua New Guinea; Sri Lanka	Fulu E, Miedema S, Roselli T, McCook S, Chan KL, Haardörfer R, Jewkes R, UN Multi-country Study on Men and Violence study team. Pathways between childhood trauma, intimate partner violence, and harsh parenting: findings from the UN Multi-country Study on Men and Violence in Asia and the Pacific. Lancet Glob Health. 2017; 5(5): e512?22.

27525	Paraguay Reproductive Health Survey 2008	Paraguay	Paraguay Center for Population Studies (CEPEP). Paraguay Reproductive Health Survey 2008. Asunción, Paraguay: Paraguayan Center for Population Studies (CEPEP).
10326	Paraguay Contraceptive Prevalence Survey 1998	Paraguay	Division of Reproductive Health-Centers for Disease Control and Prevention (CDC). Paraguay Contraceptive Prevalence Survey 1998. Atlanta, United States: Centers for Disease Control and Prevention (CDC).
506588	Panama Sexual and Reproductive Health Survey 2009	Panama	Gorgas Memorial Institute for Health Studies. Panama Sexual and Reproductive Health Survey 2009. 2011.
539932	Papua New Guinea - Western Highlands and West Sepik Family Wellbeing Survey 2014	Papua New Guinea	Australian Government Department of Foreign Affairs and Trade (DFAT), FHI 360. Papua New Guinea - Western Highlands and West Sepik Family Wellbeing Survey 2014.
358824	Peru Demographic and Family Health Survey 2017	Peru	National Center for Food and Nutrition, National Institute of Health (Peru), National Institute of Statistics and Informatics (Peru), National Police of Peru (PNP). Peru Demographic and Family Health Survey 2017. Lima, Peru: National Institute of Statistics and Informatics (Peru).
270404	Peru Continuous Demographic and Health Survey 2009	Peru	National Institute of Statistics and Informatics (Peru), ORC Macro. Peru Continuous Demographic and Health Survey 2009. Fairfax, United States of America: ICF International.
270469	Peru Continuous Demographic and Health Survey 2010	Peru	National Institute of Statistics and Informatics (Peru). Peru Continuous Demographic and Health Survey 2010. Fairfax, United States of America: ICF International.
270470	Peru Continuous Demographic and Health Survey 2011	Peru	Macro International, Inc, National Institute of Statistics and Informatics (Peru). Peru Continuous Demographic and Health Survey 2011. Fairfax, United States of America: ICF International.
270471	Peru Continuous Demographic and Health Survey 2012	Peru	Macro International, Inc, National Institute of Statistics and Informatics (Peru). Peru Continuous Demographic and Health Survey 2012. Fairfax, United States of America: ICF International.
275090	Peru Continuous Demographic and Health Survey 2003-2008	Peru	Ministry of Economy and Finance (Peru), National Institute of Statistics and Informatics (Peru), ORC Macro. Peru Continuous Demographic and Health Survey 2003-2008. Fairfax, United States of America: ICF International.
169742	Peru - Lima and Ayacucho Gender, Alcohol and Culture: An International Study (GENACIS) 2005	Peru	Aarhus University, Addiction Switzerland Research Institute, Alcohol Research Group, Public Health Institute, Centre for Addiction and Mental Health (Canada), Centre for Alcohol Policy Research, Turning Point Alcohol and Drug Centre (Australia), Kettil

			Bruun Society for Social and Epidemiological Research on Alcohol, University of North Dakota. Peru - Lima and Ayacucho Gender, Alcohol and Culture: An International Study (GENACIS) 2005.
210231	Peru Continuous Demographic and Health Survey 2013 - INEI	Peru	ICF International, National Institute of Statistics and Informatics (Peru). Peru Continuous Demographic and Health Survey 2013 - INEI. Lima, Peru: National Institute of Statistics and Informatics (Peru), 2014.
210182	Peru Continuous Demographic and Health Survey 2014 - INEI	Peru	ICF International, Ministry of Health (Peru), National Institute of Statistics and Informatics (Peru), National Police of Peru (PNP). Peru Continuous Demographic and Health Survey 2014 - INEI. Lima, Peru: National Institute of Statistics and Informatics (Peru), 2015.
150518	Peru WHO Multi-country Study on Women's Health and Domestic Violence Against Women 2000	Peru	Cayetano Heredia University, Flora Tristan Center of Peruvian Women, World Health Organization (WHO). Peru WHO Multi-country Study on Women's Health and Domestic Violence Against Women 2000.
452894	Peru Demographic and Family Health Survey 2019	Peru	National Institute of Statistics and Informatics (Peru). Peru Demographic and Family Health Survey 2019. Lima, Peru: National Institute of Statistics and Informatics (Peru).
142943	Philippines Demographic and Health Survey 2013	Philippines	ICF International, Philippines Statistics Authority. Philippines Demographic and Health Survey 2013. Fairfax, United States of America: ICF International, 2014.
21421	Philippines Demographic and Health Survey 2008	Philippines	Macro International, Inc, National Statistics Office (Philippines). Philippines Demographic and Health Survey 2008. Fairfax, United States of America: ICF International, 2010.
337877	Philippines Demographic and Health Survey 2017	Philippines	ICF International, Philippines Statistics Authority, United States Agency for International Development (USAID). Philippines Demographic and Health Survey 2017. Fairfax, United States of America: ICF International, 2018.
527435	Philippines Demographic and Health Survey 2022	Philippines	ICF International, Philippine Statistics Authority, United States Agency for International Development (USAID). Philippines Demographic and Health Survey 2022. Fairfax, United States of America: ICF International, 2023.
126440	Prevalence and characteristics of sexual violence in the Netherlands, the risk of revictimization and pregnancy: results from a national population survey	Netherlands	De Haas S, van Berlo W, Bakker F, Vanwesenbeeck I. Prevalence and characteristics of sexual violence in the Netherlands, the risk of revictimization and pregnancy: results from a national population survey. Violence Vict. 2012; 27(4): 592-608.
137137	Prevalence of Childhood Sexual Abuse Experiences in a Community Sample of Women	New Zealand	Anderson J, Martin J, Mullen P, Romans S, Herbison P. Prevalence of Childhood Sexual Abuse Experiences in a Community Sample

			of Women. J Am Acad Child Adolesc Psychiatry. 1993; 32(5): 911-9.
137145	Psychosocial sequelae of violent victimization in a national youth sample	United States of America	Boney-McCoy S, Finkelhor D. Psychosocial sequelae of violent victimization in a national youth sample. J Consult Clin Psychol. 1995; 63(5): 726-36.
137153	Prevalence and characteristics of sexual abuse in a national sample of Swedish seventeen-year-old boys and girls	Sweden	Edgardh K, Ormstad K. Prevalence and characteristics of sexual abuse in a national sample of Swedish seventeen-year-old boys and girls. Acta Paediatr. 2000; 89(3): 310-9.
137155	Prevalence of child sexual abuse reported by a cross-sectional sample of New Zealand women	New Zealand	Fanslow JL, Robinson EM, Crengle S, Perese L. Prevalence of child sexual abuse reported by a cross-sectional sample of New Zealand women. Child Abuse Negl. 2007; 31(9): 935-45.
137184	Prevalence and correlates of sexual abuse reported by late adolescent school children in Sri Lanka	Sri Lanka	Perera B, Ostbye T. Prevalence and correlates of sexual abuse reported by late adolescent school children in Sri Lanka. Int J Adolesc Med Health. 2009; 21(2): 203-11.
137185	Prevalence of childhood sexual abuse among Mexican adolescents	Mexico	Pineda-Lucatero AG, Trujillo-Hernández B, Millán-Guerrero RO, Vásquez C. Prevalence of childhood sexual abuse among Mexican adolescents. Child Care Health Dev. 2009; 35(2): 184-9.
137200	Prevalence and risk factors for childhood sexual abuse in women: national survey findings	United States of America	Vogeltanz ND, Wilsnack SC, Harris TR, Wilsnack RW, Wonderlich SA, Kristjanson AF. Prevalence and risk factors for childhood sexual abuse in women: national survey findings. Child Abuse Negl. 1999; 23(6): 579-92.
148642	Prevalence of childhood sexual abuse among female students in a senior high school	China	Chen J. Prevalence of childhood sexual abuse among female students in a senior high school. Chin J Sch Health. 2002; 23: 108?10.
150446	Prevalence and co-occurrence of violence against children in the Quebec population	Canada	Tourigny M, Hébert M, Joly J, Cyr M, Baril K. Prevalence and co-occurrence of violence against children in the Quebec population. Aust N Z J Public Health. 2008; 32(4): 331-5.
150613	Prevalence and correlates of drug/alcohol-facilitated and incapacitated sexual assault in a nationally representative sample of adolescent girls	United States of America	McCauley JL, Conoscenti LM, Ruggiero KJ, Resnick HS, Saunders BE, Kilpatrick DG. Prevalence and correlates of drug/alcohol-facilitated and incapacitated sexual assault in a nationally representative sample of adolescent girls. J Clin Child Adolesc Psychol. 2009; 38(2): 295-300.
14486	Puerto Rico Reproductive Health Survey 1995-1996	United States of America	University of Puerto Rico. Puerto Rico Reproductive Health Survey 1995-1996. San Juan, Puerto Rico: University of Puerto Rico, 1998.
454625	Republic of Moldova Violence Against Children and Youth Survey 2018-2019	Republic of Moldova	Centers for Disease Control and Prevention (CDC), International Organization for Migration (IOM), Ministry of Health (Moldova). Republic of Moldova Violence Against Children and Youth Survey 2018-

			2019. Washington, DC, United States of America: Together for Girls.
126434	Romania Balkan Epidemiological Study on Child Abuse and Neglect 2011	Romania	Babe?-Bolyai University, Institute of Child Health (Greece). Romania Balkan Epidemiological Study on Child Abuse and Neglect 2011.
137133	Reported childhood trauma, attempted suicide and self-mutilative behavior among women in the general population	Türkiye	Akyuz G, Sar V, Kugu N, Dogan O. Reported childhood trauma, attempted suicide and self-mutilative behavior among women in the general population. Eur Psychiatry. 2005; 20(3): 268-73.
282352	Relationship between child abuse exposure and reported contact with child protection organizations: results from the Canadian Community Health Survey	Canada	Afifi TO, MacMillan HL, Taillieu T, Cheung K, Turner S, Tonmyr L, Hovdestad W. Relationship between child abuse exposure and reported contact with child protection organizations: results from the Canadian Community Health Survey. Child Abuse Negl. 2015; 46: 198?206.
408923	Rwanda Violence Against Children and Youth Survey 2015	Rwanda	Centers for Disease Control and Prevention (CDC), Ministry of Education (Rwanda), Ministry of Gender and Family Promotion (Rwanda), Ministry of Health (Rwanda), National Commission for Children (NCC) (Rwanda), National Institute of Statistics (Rwanda), Rwanda National Police (RNP), Together for Girls, United Nations Children's Fund (UNICEF). Rwanda Violence Against Children and Youth Survey 2015.
20740	Rwanda Demographic and Health Survey 2005	Rwanda	Macro International, Inc, National Institute of Statistics of Rwanda. Rwanda Demographic and Health Survey 2005. Fairfax, United States of America: ICF International.
26866	Sao Tome and Principe Demographic and Health Survey 2008-2009	Sao Tome and Principe	ICF Macro, Ministry of Health (Sao Tome and Principe), National Institute of Statistics (Sao Tome and Principe). Sao Tome and Principe Demographic and Health Survey 2008-2009. Fairfax, United States of America: ICF International.
157063	Rwanda Demographic and Health Survey 2014-2015	Rwanda	ICF International, Ministry of Health (Rwanda), National Institute of Statistics of Rwanda. Rwanda Demographic and Health Survey 2014-2015. Fairfax, United States of America: ICF International, 2016.
126427	Samoa Family Health and Safety Study 2000	Samoa	Ministry of Women Affairs (Samoa), Secretariat of the Pacific Community (SPC). Samoa Family Health and Safety Study 2000.
56040	Rwanda Demographic and Health Survey 2010-2011	Rwanda	ICF Macro, Ministry of Health (Rwanda), National Institute of Statistics of Rwanda. Rwanda Demographic and Health Survey 2010-2011. Fairfax, United States of America: ICF International.
462482	Rwanda Demographic and Health Survey 2019-2020	Rwanda	Ministry of Health (Rwanda), National Institute of Statistics (Rwanda), Rwanda Biomedical Center (RBC). Rwanda Demographic and Health Survey 2019-2020.

			Fairfax, United States of America: ICF International, 2021.
497190	São Tomé and Príncipe STEPS Noncommunicable Disease Risk Factors Survey 2019	Sao Tome and Principe	Ministry of Health (Sao Tome and Principe), World Health Organization (WHO). São Tomé and Príncipe STEPS Noncommunicable Disease Risk Factors Survey 2019. WHO NCD Microdata Repository, 2022.
513133	Rwanda Masculinity and Gender Based Violence Survey 2010	Rwanda	International Center for Research on Women, Rwanda Men's Resource Centre (RWAMREC), Rwanda MenEngage Network, United Nations Development Programme (UNDP). Rwanda Masculinity and Gender Based Violence Survey 2010.
353526	Senegal Continuous Demographic and Health Survey 2017	Senegal	ICF International, Ministry of Health and Social Action (Senegal), National Agency of Statistics and Demography (Senegal), Unit for the Fight Against Malnutrition (Senegal). Senegal Continuous Demographic and Health Survey 2017. Fairfax, United States of America: ICF International, 2018.
126432	Serbia Balkan Epidemiological Study on Child Abuse and Neglect 2011	Serbia	Institute of Child Health (Greece), University of Belgrade. Serbia Balkan Epidemiological Study on Child Abuse and Neglect 2011.
137150	Self-reported health and behavioral problems among adolescent victims of rape in France: Results of a cross-sectional survey	France	Choquet M, Darves-Bornoz J-M, Ledoux S, Manfredi R, Hassler C. Self-reported health and behavioral problems among adolescent victims of rape in France: Results of a cross-sectional survey. Child Abuse Negl. 1997; 21(9): 823-32.
137158	Self-reported suicide attempts and associated risk and protective factors among secondary school students in New Zealand	New Zealand	Fleming TM, Merry SN, Robinson EM, Denny SJ, Watson PD. Self-reported suicide attempts and associated risk and protective factors among secondary school students in New Zealand. Aust N Z J Psychiatry. 2007; 41(3): 213-21.
137177	Secular trends in child and adult sexual violence - one decreasing and the other increasing: a population survey in Ireland	Ireland	McGee H, Garavan R, Byrne J, O'Higgins M, Conroy RM. Secular trends in child and adult sexual violence - one decreasing and the other increasing: a population survey in Ireland. Eur J Public Health. 2011; 21(1): 98-103.
460813	Senegal Continuous Demographic and Health Survey 2019	Senegal	ICF International, Ministry of Health and Social Action (Senegal), National Agency of Statistics and Demography (Senegal), United States Agency for International Development (USAID). Senegal Continuous Demographic and Health Survey 2019. Fairfax, United States of America: ICF International, 2020.
450419	Senegal Continuous Demographic and Health Survey 2018	Senegal	Directorate of Forecasting and Statistics, Ministry of the Economy, Finance and Planning (Senegal), ICF International, Ministry of Health and Social Action (Senegal), United States Agency for International Development (USAID). Senegal Continuous Demographic and Health Survey

			2018. Fairfax, United States of America: ICF International, 2020.
131467	Sierra Leone Demographic and Health Survey 2013	Sierra Leone	ICF International, Ministry of Health and Sanitation (Sierra Leone), Statistics Sierra Leone. Sierra Leone Demographic and Health Survey 2013. Fairfax, United States of America: ICF International, 2014.
137138	Shaming experiences and the association between adolescent depression and psychosocial risk factors	Sweden	Åslund C, Nilsson K, Starrin B, Sjöberg R. Shaming experiences and the association between adolescent depression and psychosocial risk factors. Eur Child Adolesc Psychiatry. 2007; 16(5): 298-304.
137144	Sexual Abuse and Suicidal Behavior: A Model Constructed From a Large Community Sample of Adolescents	Australia	Bergen HA, Martin G, Richardson AS, Allison S, Roeger L. Sexual Abuse and Suicidal Behavior: A Model Constructed From a Large Community Sample of Adolescents. J Am Acad Child Adolesc Psychiatry. 2003; 42(11): 1301-9.
137198	Sexual victimization in adolescent girls (age 15-20 years) enrolled in post-mandatory schools or professional training programmes in Switzerland	Switzerland	Tschumper A, Narring F, Meier C, Michaud P. Sexual victimization in adolescent girls (age 15-20 years) enrolled in post-mandatory schools or professional training programmes in Switzerland. Acta Paediatr. 1998; 87(2): 212-7.
148647	Sexual Violence and Reproductive Health Among Young People in Three Communities in Jamaica	Jamaica	Geary CW, Wedderburn M, McCarraher D, Cuthbertson C, Pottinger A. Sexual Violence and Reproductive Health Among Young People in Three Communities in Jamaica. J Interpers Violence. 2006; 21(11): 1512?33.
150416	Sexual abuse in a national survey of adult men and women: prevalence, characteristics, and risk factors	United States of America	Finkelhor D, Hotaling G, Lewis IA, Smith C. Sexual abuse in a national survey of adult men and women: prevalence, characteristics, and risk factors. Child Abuse Negl. 1990; 14(1): 19-28.
156677	Sex in Australia: experiences of sexual coercion among a representative sample of adults	Australia	de Visser RO, Smith AM, Rissel CE, Richters J, Grulich AE. Sex in Australia: experiences of sexual coercion among a representative sample of adults. Aust N Z J Public Health. 2003; 27(2): 198-203.
157064	South Africa Demographic and Health Survey 2016	South Africa	Department of Health (South Africa), ICF International, South African Medical Research Council, Statistics South Africa. South Africa Demographic and Health Survey 2016. Fairfax, United States of America: ICF International, 2019.
20796	South Africa Demographic and Health Survey 1998	South Africa	Department of Health (South Africa), Macro International, Inc, South African Medical Research Council. South Africa Demographic and Health Survey 1998. Fairfax, United States of America: ICF International.
284975	South Africa Optimus Study 2014	South Africa	Centre for Justice and Crime Prevention (South Africa), UBS Optimus Foundation, University of Cape Town. South Africa Optimus Study 2014. 2016.

344441	South Africa Gender Based Violence Indicators Study 2010-2013	South Africa	Gender Links (South Africa). South Africa Gender Based Violence Indicators Study 2010-2013.
126426	Solomon Islands Family Health and Safety Study 2008	Solomon Islands	Solomon Islands National Statistics Office (SINSO). Solomon Islands Family Health and Safety Study 2008.
169743	Spain Gender, Alcohol and Culture: An International Study (GENACIS) 2003	Spain	Aarhus University, Addiction Switzerland Research Institute, Alcohol Research Group, Public Health Institute, Centre for Addiction and Mental Health (Canada), Centre for Alcohol Policy Research, Turning Point Alcohol and Drug Centre (Australia), Kettil Bruun Society for Social and Epidemiological Research on Alcohol, University of North Dakota. Spain Gender, Alcohol and Culture: An International Study (GENACIS) 2003.
169744	Sri Lanka Gender, Alcohol and Culture: An International Study (GENACIS) 2002	Sri Lanka	Aarhus University, Addiction Switzerland Research Institute, Alcohol Research Group, Public Health Institute, Centre for Addiction and Mental Health (Canada), Centre for Alcohol Policy Research, Turning Point Alcohol and Drug Centre (Australia), Kettil Bruun Society for Social and Epidemiological Research on Alcohol, University of North Dakota. Sri Lanka Gender, Alcohol and Culture: An International Study (GENACIS) 2002.
137170	Student victimization by educational staff in Israel	Israel	Khoury-Kassabri M. Student victimization by educational staff in Israel. Child Abuse Negl. 2006; 30(6): 691-707.
137171	Substance abuse and behavioral correlates of sexual assault among South African adolescents	South Africa	King G, Flisher AJ, Noubary F, Reece R, Marais A, Lombard C. Substance abuse and behavioral correlates of sexual assault among South African adolescents. Child Abuse Negl. 2004; 28(6): 683-96.
503525	Sri Lanka United Nations Multi-country Study on Men and Violence 2012-2013 - Equimundo	Sri Lanka	CARE International, Partners for Prevention, United Nations Development Programme (UNDP), United Nations Entity for Gender Equality and the Empowerment of Women (UN Women), United Nations Population Fund (UNFPA), United Nations Volunteers (UNV). Sri Lanka United Nations Multi-country Study on Men and Violence 2012-2013 - Equimundo.
507100	Spain Violence Against Women Macro Survey 2014	Spain	Government Delegation for Gender Violence (Spain), Ministry of Health, Social Services and Equality (Spain), Sociological Research Center (Spain). Spain Violence Against Women Macro Survey 2014.
508070	Sri Lanka Women?s Wellbeing Survey 2019	Sri Lanka	Department of Census and Statistics (Sri Lanka). Sri Lanka Women?s Wellbeing Survey 2019.
539964	Spain Violence Against Women Macro Survey 2019	Spain	Government Delegation for Gender Violence (Spain), Ministry of Health, Social Services and Equality (Spain), Sociological Research

			Center (Spain). Spain Violence Against Women Macro Survey 2019.
225621	Sweden National Prevalence Study on Exposure to Violence 2012	Sweden	National Board of Health and Welfare (Sweden), National Center for Knowledge on Men's Violence Against Women, Uppsala University, Statistics Sweden. Sweden National Prevalence Study on Exposure to Violence 2012.
126418	Swaziland National Study on Violence Against Children and Young Women 2007	Eswatini	Centers for Disease Control and Prevention (CDC), United Nations Children's Fund (UNICEF). Swaziland National Study on Violence Against Children and Young Women 2007.
250033	Swaziland STEPS Noncommunicable Disease Risk Factors Survey 2014	Eswatini	Ministry of Health (Swaziland), World Health Organization (WHO). Swaziland STEPS Noncommunicable Disease Risk Factors Survey 2014.
21331	Tanzania Demographic and Health Survey 2009-2010	United Republic of Tanzania	ICF Macro, National Bureau of Statistics (Tanzania). Tanzania Demographic and Health Survey 2009-2010. Fairfax, United States of America: ICF International.
74460	Tajikistan Demographic and Health Survey 2012	Tajikistan	ICF International, Ministry of Health (Tajikistan), Statistical Agency under the President of the Republic of Tajikistan. Tajikistan Demographic and Health Survey 2012. Fairfax, United States of America: ICF International, 2013.
218593	Tanzania Demographic and Health Survey 2015-2016	United Republic of Tanzania	ICF International, Ministry of Health (Zanzibar), Ministry of Health, Community Development, Gender, Elderly and Children (MoHCDEC) (Tanzania), National Bureau of Statistics (Tanzania), Office of the Chief Government Statistician (OCGS) (Zanzibar). Tanzania Demographic and Health Survey 2015-2016. Fairfax, United States of America: ICF International, 2016.
341838	Tajikistan Demographic and Health Survey 2017	Tajikistan	ICF International, Statistical Agency under the President of the Republic of Tajikistan. Tajikistan Demographic and Health Survey 2017. Fairfax, United States of America: ICF International, 2018.
126422	Switzerland Optimus Study 2009-2010	Switzerland	UBS Optimus Foundation, University of Zurich. Switzerland Optimus Study 2009-2010. 2012.
152098	Switzerland International Violence Against Women Survey 2003	Switzerland	European Institute for Crime Prevention and Control, affiliated with the United Nations (HEUNI), United Nations Interregional Crime and Justice Research Institute (UNICRI), University of Lausanne. Switzerland International Violence Against Women Survey 2003.
538795	Tanzania Demographic and Health Survey 2022	United Republic of Tanzania	Bureau of Statistics (Tanzania), ICF International, Ministry of Health (Tanzania), Ministry of Health (Zanzibar), Office of the Chief Government Statistician (OCGS)

			(Zanzibar), Tanzania Food and Nutrition Centre. Tanzania Demographic and Health Survey 2022. Fairfax, United States of America: ICF International, 2023.
126419	Tanzania Violence Against Children Study 2009	United Republic of Tanzania	Ministry of Community Development, Gender and Children (MCDGC) (Tanzania), Muhimbili University of Health and Allied Sciences (Tanzania), National Center for Injury Prevention and Control, Centers for Disease Control and Prevention (CDC), United Nations Children's Fund (UNICEF). Tanzania Violence Against Children Study 2009.
150524	Thailand WHO Multi-country Study on Women's Health and Domestic Violence Against Women 2000	Thailand	Foundation for Women (Thailand), Institute for Population and Social Research, Mahidol University (Thailand), World Health Organization (WHO). Thailand WHO Multi-country Study on Women's Health and Domestic Violence Against Women 2000.
150523	Tanzania WHO Multi-country Study on Women's Health and Domestic Violence Against Women 2001-2002	United Republic of Tanzania	Muhimbili University of Health and Allied Sciences (Tanzania), University of Dar es Salaam, World Health Organization (WHO). Tanzania WHO Multi-country Study on Women's Health and Domestic Violence Against Women 2001-2002.
137154	The Experience of Sexual Coercion among Young People in Kenya	Kenya	Erulkar AS. The Experience of Sexual Coercion among Young People in Kenya. Int Fam Plan Perspect. 2004; 30(4): 182-9.
137196	The association between childhood and adolescent sexual abuse and proxies for sexual risk behavior: a random sample of the general population of Sweden	Sweden	Steel JL, Herlitz CA. The association between childhood and adolescent sexual abuse and proxies for sexual risk behavior: a random sample of the general population of Sweden. Child Abuse Negl. 2005; 29(10): 1141-53.
21274	Timor-Leste Demographic and Health Survey 2009-2010	Timor-Leste	ICF Macro, Ministry of Finance (Timor-Leste), National Statistics Directorate (Timor-Leste). Timor-Leste Demographic and Health Survey 2009-2010. Fairfax, United States of America: ICF International.
286785	Timor-Leste Demographic and Health Survey 2016	Timor-Leste	ICF International, National Statistics Directorate (Timor-Leste). Timor-Leste Demographic and Health Survey 2016. Fairfax, United States of America: ICF International, 2018.
126425	Tonga National Study on Domestic Violence Against Women 2008-2009	Tonga	Ma?a Fafine mo e Famili (Tonga). Tonga National Study on Domestic Violence Against Women 2008-2009.
77515	Togo Demographic and Health Survey 2013-2014	Togo	Directorate General of Statistics and National Accounts (Togo), ICF International, Ministry of Health (Togo), Ministry of Planning, Development and Zoning (Togo). Togo Demographic and Health Survey 2013-2014. Fairfax, United States of America: ICF International, 2015.

120028	Trauma exposure and post-traumatic stress symptoms in urban African schools Survey in CapeTown and Nairobi	Kenya; South Africa	Seedat S, Nyamai C, Njenga F, Vythilingum B, Stein DJ. Trauma exposure and post-traumatic stress symptoms in urban African schools Survey in CapeTown and Nairobi. Br J Psychiatry. 2004; 184(2): 169-75.
137136	The Relationship Between Child Abuse and Adult Obesity Among California Women	United States of America	Alvarez J, Pavao J, Baumrind N, Kimerling R. The Relationship Between Child Abuse and Adult Obesity Among California Women. Am J Prev Med. 2007; 33(1): 28-33.
137164	The prevalence of unwanted and unlawful sexual experiences reported by Danish adolescents: Results from a national youth survey in 2002	Denmark	Helweg-Larsen K, Larsen HB. The prevalence of unwanted and unlawful sexual experiences reported by Danish adolescents: Results from a national youth survey in 2002. Acta Paediatr. 2006; 95(10): 1270-6.
137179	The prevalence of childhood sexual abuse and adolescent unwanted sexual contact among boys and girls living in Victoria, Australia	Australia	Moore EE, Romaniuk H, Olsson CA, Jayasinghe Y, Carlin JB, Patton GC. The prevalence of childhood sexual abuse and adolescent unwanted sexual contact among boys and girls living in Victoria, Australia. Child Abuse Negl. 2010; 34(5): 379-85.
137188	The prevalence of child sexual abuse in Finland	Finland	Sariola H, Uutela A. The prevalence of child sexual abuse in Finland. Child Abuse Negl. 1994; 18(10): 827-35.
150445	To unfold a hidden epidemic: prevalence of child maltreatment and its health implications among high school students in Guangzhou, China	China	Wong WCW, Leung PWS, Tang CSK, Chen W-Q, Lee A, Ling DC. To unfold a hidden epidemic: prevalence of child maltreatment and its health implications among high school students in Guangzhou, China. Child Abuse Negl. 2009; 33(7): 441-50.
467160	Timor-Leste Nabilan Health and Life Experiences Baseline Study 2015	Timor-Leste	The Asia Foundation. Timor-Leste Nabilan Health and Life Experiences Baseline Study 2015.
554411	The prevalence of child maltreatment in Australia: findings from a national survey	Australia	Mathews B, Pacella R, Scott JG, Finkelhor D, Meinck F, Higgins DJ, Erskine HE, Thomas HJ, Lawrence DM, Haslam DM, Malacova E, Dunne MP. The prevalence of child maltreatment in Australia: findings from a national survey. Med J Aust. 2023; 218 Suppl 6(Suppl 6): S13-S18.
126423	Turkey National Research on Domestic Violence Against Women 2008	Türkiye	BNB Consulting (Turkey), ICON-INSTITUTE Consulting Group, Institute of Population Studies, Hacettepe University. Turkey National Research on Domestic Violence Against Women 2008.
21014	Uganda Demographic and Health Survey 2006	Uganda	Macro International, Inc, Uganda Bureau of Statistics. Uganda Demographic and Health Survey 2006. Fairfax, United States of America: ICF International.
239733	Turkey National Research on Domestic Violence Against Women 2014	Türkiye	Institute of Population Studies, Hacettepe University, Ministry of Family and Social Policies (Turkey). Turkey National Research on Domestic Violence Against Women 2014.
56021	Uganda Demographic and Health Survey 2011	Uganda	ICF Macro, Uganda Bureau of Statistics. Uganda Demographic and Health Survey

			2011. Fairfax, United States of America: ICF International.
286780	Uganda Demographic and Health Survey 2016	Uganda	ICF International, Uganda Bureau of Statistics. Uganda Demographic and Health Survey 2016. Fairfax, United States of America: ICF International, 2018.
169748	Uganda Gender, Alcohol and Culture: An International Study (GENACIS) 2003	Uganda	Aarhus University, Addiction Switzerland Research Institute, Alcohol Research Group, Public Health Institute, Centre for Addiction and Mental Health (Canada), Centre for Alcohol Policy Research, Turning Point Alcohol and Drug Centre (Australia), Kettil Bruun Society for Social and Epidemiological Research on Alcohol, University of North Dakota. Uganda Gender, Alcohol and Culture: An International Study (GENACIS) 2003.
23289	Uganda Child Verbal Autopsy Study 2007	Uganda	MEASURE Evaluation Project, Carolina Population Center, University of North Carolina, Macro International, Inc, Ministry of Health (Uganda), Uganda Bureau of Statistics. Uganda Child Verbal Autopsy Study 2007. Calverton, United States of America: Macro International, Inc.
21024	Ukraine Demographic and Health Survey 2007	Ukraine	Macro International, Inc, State Statistical Committee (Ukraine), Ukrainian Center for Social Reforms (UCSR). Ukraine Demographic and Health Survey 2007. Fairfax, United States of America: ICF International.
408836	Uganda Violence Against Children Survey 2015	Uganda	AfriChild Centre, Centers for Disease Control and Prevention (CDC), ChildFund International (CFI), Makerere University School of Public Health (Uganda), Ministry of Gender, Labour and Social Development (MGLSD) (Uganda), Together for Girls, Transcultural Psychological Organisation (Uganda), UNICEF Uganda, Uganda Bureau of Statistics, United Nations Children's Fund (UNICEF). Uganda Violence Against Children Survey 2015.
13218	Ukraine Reproductive Health Survey 1999	Ukraine	Division of Reproductive Health-Centers for Disease Control and Prevention (CDC) and Kiev International Institute of Sociology. (2001) Ukraine Reproductive Health Survey 1999. Atlanta, United States: Centers for Disease Control and Prevention (CDC).
554627	Uganda National Survey on Violence 2020	Uganda	Uganda Bureau of Statistics. Uganda National Survey on Violence 2020.
126610	United Kingdom - England Adult Psychiatric Morbidity Survey 2006-2007 - UK Data Service	United Kingdom	National Centre for Social Research and University of Leicester, Adult Psychiatric Morbidity Survey, 2007 [computer file]. 3rd Edition. Colchester, Essex: UK Data Archive [distributor], January 2011. SN: 6379, http://dx.doi.org/10.5255/UKDA-SN-6379-1

169749	United Kingdom Gender, Alcohol and Culture: An International Study (GENACIS) 2000	United Kingdom	Aarhus University, Addiction Switzerland Research Institute, Alcohol Research Group, Public Health Institute, Centre for Addiction and Mental Health (Canada), Centre for Alcohol Policy Research, Turning Point Alcohol and Drug Centre (Australia), Kettil Bruun Society for Social and Epidemiological Research on Alcohol, University of North Dakota. United Kingdom Gender, Alcohol and Culture: An International Study (GENACIS) 2000.
490245	United Kingdom - Scottish Health Survey 2019 - Scottish Government	United Kingdom	Scottish Centre for Social Research (ScotCen), University of Glasgow. United Kingdom - Scottish Health Survey 2019 - Scottish Government.
104825	United States Behavioral Risk Factor Surveillance System 2012	United States of America	Centers for Disease Control and Prevention (CDC). United States Behavioral Risk Factor Surveillance System 2012. Atlanta, Georgia: CDC, US Department of Health and Human Services, 2013.
148648	United States Commonwealth Fund Survey of Women's Health 1998	United States of America	Louis Harris and Associates. United States Commonwealth Fund Survey of Women's Health 1998. New York, United States of America: Commonwealth Fund, 1999.
152745	United States Commonwealth Fund Survey of the Health of Adolescent Girls and Boys 1996-1997	United States of America	Louis Harris and Associates. United States Commonwealth Fund Survey of the Health of Adolescent Girls and Boys 1996-1997. New York, United States of America: Commonwealth Fund, 1997.
30018	United States Behavioral Risk Factor Surveillance System 2009	United States of America	Centers for Disease Control and Prevention (CDC). United States Behavioral Risk Factor Surveillance System 2009. Atlanta, Georgia: CDC, US Department of Health and Human Services.
83627	United States Behavioral Risk Factor Surveillance System 2010	United States of America	Centers for Disease Control and Prevention (CDC). United States Behavioral Risk Factor Surveillance System 2010. Atlanta, United States of America: Centers for Disease Control and Prevention (CDC).
83633	United States Behavioral Risk Factor Surveillance System 2011	United States of America	Centers for Disease Control and Prevention (CDC). United States Behavioral Risk Factor Surveillance System 2011. Atlanta, Georgia: CDC, US Department of Health and Human Services.
283356	United States High School Youth Risk Behavior Survey 1991-2015	United States of America	Centers for Disease Control and Prevention (CDC). United States High School Youth Risk Behavior Survey 1991-2015. Atlanta, United States of America: Centers for Disease Control and Prevention (CDC).
169751	United States Gender, Alcohol and Culture: An International Study (GENACIS) 2001	United States of America	Aarhus University, Addiction Switzerland Research Institute, Alcohol Research Group, Public Health Institute, Centre for Addiction and Mental Health (Canada), Centre for Alcohol Policy Research, Turning Point Alcohol and Drug Centre (Australia), Kettil

			Bruun Society for Social and Epidemiological Research on Alcohol, University of North Dakota. United States Gender, Alcohol and Culture: An International Study (GENACIS) 2001.
532948	United States Behavioral Risk Factor Surveillance System 2022	United States of America	Centers for Disease Control and Prevention (CDC). United States Behavioral Risk Factor Surveillance System 2022. Atlanta, United States of America: Centers for Disease Control and Prevention (CDC), 2023.
73721	United States National Comorbidity Survey: Baseline 1990-1992	United States of America	Kessler, Ronald C. National Comorbidity Survey: Baseline (NCS-1), 1990-1992. ICPSR06693-v6. Ann Arbor, MI: Inter-university Consortium for Political and Social Research [distributor], 2008-09-12. http://doi.org/10.3886/ICPSR06693.v6
120195	United States National Longitudinal Study of Adolescent to Adult Health 2007-2009, Wave 4	United States of America	Harris, Kathleen Mullan, and J. Richard Udry. National Longitudinal Study of Adolescent to Adult Health (Add Health), 1994-2008. ICPSR21600-v12. Ann Arbor, MI: Inter-university Consortium for Political and Social Research [distributor], 2013-03-08. doi:10.3886/ICPSR21600.v12
124091	United States National Epidemiologic Survey on Alcohol and Related Conditions 2004-2005	United States of America	National Institute on Alcohol Abuse and Alcoholism (NIAAA), National Institutes of Health (NIH), U.S. Department of Health and Human Services. United States National Epidemiologic Survey on Alcohol and Related Conditions 2004-2005.
150625	United States National Violence Against Women Survey 1995-1996	United States of America	Centers for Disease Control and Prevention (CDC), National Institute of Justice (United States), Schulman, Ronca and Bucuvalas Inc. (SRBI). United States National Violence Against Women Survey 1995-1996.
449434	United States National Youth Risk Behavior Survey 2019	United States of America	Centers for Disease Control and Prevention (CDC). United States National Youth Risk Behavior Survey 2019. Atlanta, United States of America: Centers for Disease Control and Prevention (CDC).
169750	Uruguay Gender, Alcohol and Culture: An International Study (GENACIS) 2004	Uruguay	Aarhus University, Addiction Switzerland Research Institute, Alcohol Research Group, Public Health Institute, Centre for Addiction and Mental Health (Canada), Centre for Alcohol Policy Research, Turning Point Alcohol and Drug Centre (Australia), Kettil Bruun Society for Social and Epidemiological Research on Alcohol, University of North Dakota. Uruguay Gender, Alcohol and Culture: An International Study (GENACIS) 2004.
540927	United States Virgin Islands Youth Risk Behavior Survey 2017	United States of America	Department of Health (United States Virgin Islands), University of the Virgin Islands (United States). United States Virgin Islands Youth Risk Behavior Survey 2017.

126424	Vietnam National Study of Domestic Violence Against Women 2009-2010	Viet Nam	General Statistics Office (Vietnam), World Health Organization (WHO). Vietnam National Study of Domestic Violence Against Women 2009-2010.
355055	Violence against children, later victimisation, and mental health: a cross-sectional study of the general Norwegian population	Norway	Thoresen S, Myhre M, Wentzel-Larsen T, Aakvaag HF, Hjemdal OK. Violence against children, later victimisation, and mental health: a cross-sectional study of the general Norwegian population. Eur J Psychotraumatol. 2015; 6(1): 26259.
128665	Victimization experiences of adolescents in Malaysia	Malaysia	Choo W-Y, Dunne MP, Marret MJ, Fleming M, Wong Y-L. Victimization experiences of adolescents in Malaysia. J Adolesc Health. 2011; 49(6): 627-34.
137206	Violence Against Women: An International Perspective	Australia; Canada; China; Costa Rica; Czechia; Denmark; Greece; Italy; Mozambique; Philippines; Poland; Switzerland	Johnson H, Ollus N, Nevala S. Violence Against Women: An International Perspective. New York City, United States: Springer; 2008.
508124	Viet Nam National Study on Violence Against Women 2018-2019	Viet Nam	General Statistics Office (Vietnam), Ministry of Labour, Invalids and Social Affairs (MOLISA) (Vietnam), United Nations Population Fund (UNFPA). Viet Nam National Study on Violence Against Women 2018-2019.
21117	Zambia Demographic and Health Survey 2007	Zambia	Central Statistical Office (Zambia), Macro International, Inc. Zambia Demographic and Health Survey 2007. Fairfax, United States of America: ICF International.
231766	Zimbabwe National Baseline Survey on Life Experiences of Adolescents 2011	Zimbabwe	Centers for Disease Control and Prevention (CDC), Centre for Collaborative Operational Research and Evaluation (Zimbabwe), United Nations Children's Fund (UNICEF), Zimbabwe National Statistics Agency. Zimbabwe National Baseline Survey on Life Experiences of Adolescents 2011.
408815	Zambia Violence Against Children Survey (VACS) 2014	Zambia	Centers for Disease Control and Prevention (CDC), Central Statistical Office (Zambia), Ministry of Community Development and Social Services (MCDSS) (Zambia), Ministry of Youth Sport and Child Development (Zambia), Save the Children International, Together for Girls, United Nations Children's Fund (UNICEF), University of Zambia. Zambia Violence Against Children Survey (VACS) 2014.
454636	Zimbabwe Young Adult Survey: A Violence Against Children Study, 2017	Zimbabwe	Centers for Disease Control and Prevention (CDC), Elizabeth Glaser Pediatric AIDS Foundation (EGPAF), Ministry of Health and Child Welfare (Zimbabwe), Together for Girls, ZimStat. Zimbabwe Young Adult Survey: A Violence Against Children Study,

			2017. Washington, DC, United States of America: Together for Girls.
21163	Zimbabwe Demographic and Health Survey 2005-2006	Zimbabwe	Central Statistical Office (Zimbabwe), Macro International, Inc. Zimbabwe Demographic and Health Survey 2005-2006. Fairfax, United States of America: ICF International.
21102	Zambia Demographic and Health Survey 2001-2002	Zambia	Central Board of Health (Zambia), Central Statistical Office (Zambia), Macro International, Inc. Zambia Demographic and Health Survey 2001-2002. Fairfax, United States of America: ICF International.
77516	Zambia Demographic and Health Survey 2013-2014	Zambia	Central Statistical Office (Zambia), ICF International, Ministry of Health (Zambia), Tropical Diseases Research Centre, University Teaching Hospital (Zambia), University of Zambia. Zambia Demographic and Health Survey 2013-2014. Fairfax, United States of America: ICF International.
157066	Zimbabwe Demographic and Health Survey 2015	Zimbabwe	ICF International, National Microbiology Reference Laboratory, Harare Central Hospital (NMRL) (Zimbabwe), Zimbabwe National Statistics Agency. Zimbabwe Demographic and Health Survey 2015. Fairfax, United States of America: ICF International, 2016.
55992	Zimbabwe Demographic and Health Survey 2010-2011	Zimbabwe	ICF Macro, Zimbabwe National Statistics Agency. Zimbabwe Demographic and Health Survey 2010-2011. Calverton, United States of America: ICF Macro, 2012.
287630	Zambia Population-Based HIV Impact Assessment 2016	Zambia	Centers for Disease Control and Prevention (CDC), ICAP, Columbia University Mailman School of Public Health, Ministry of Health (Zambia), Statistical Center for HIV/AIDS Research and Prevention (SCHARP), Tropical Diseases Research Centre, University of Zambia, Westat. Zambia Population-Based HIV Impact Assessment 2016. New York, New York: ICAP, Columbia University Mailman School of Public Health.
411301	Zambia Demographic and Health Survey 2018-2019	Zambia	Central Statistical Office (Zambia), ICF International, Ministry of Health (Zambia), University Teaching Hospital (Zambia), University of Zambia. Zambia Demographic and Health Survey 2018-2019. Fairfax, United States of America: ICF International, 2020.
137147	[Factors for sexual abuse during childhood and adolescence in students of Morelos, Mexico]	Mexico	Chavez Ayala R, Rivera-Rivera L, Angeles-Llerenas A, Díaz-Cerón E, Allen-Leigh B, Ponce EL. [Factors for sexual abuse during childhood and adolescence in students of Morelos, Mexico]. Rev Saude Publica. 2009; 43(3): 506-14.
137172	[Prevalence and consequences of child sexual abuse in Spain]	Spain	López F, Carpintero E, Hernández A, Martín MJ, Fuertes A. [Prevalence and consequences of child sexual abuse in Spain]. Child Abuse Negl. 1995; 19(9): 1039-50.

137187	[Prevalence of sexual abuse in students and its relation with drug abuse]	Mexico	Ramos-Lira L, Saldívar-Hernández G, Medina-Mora ME, Rojas-Guiot E, Villatoro-Velázquez J. [Prevalence of sexual abuse in students and its relation with drug abuse]. Salud Publica Mex. 1998; 40(3): 221-33.
493562	Madagascar Demographic and Health Survey 2021	Madagascar	ICF Macro, National Institute of Statistics (Madagascar). Madagascar Demographic and Health Survey 2021. Fairfax, United States of America: ICF International, 2022.

Section 2.1.5: Exposure data coverage

Figure S1: Data coverage for the prevalence of intimate partner violence

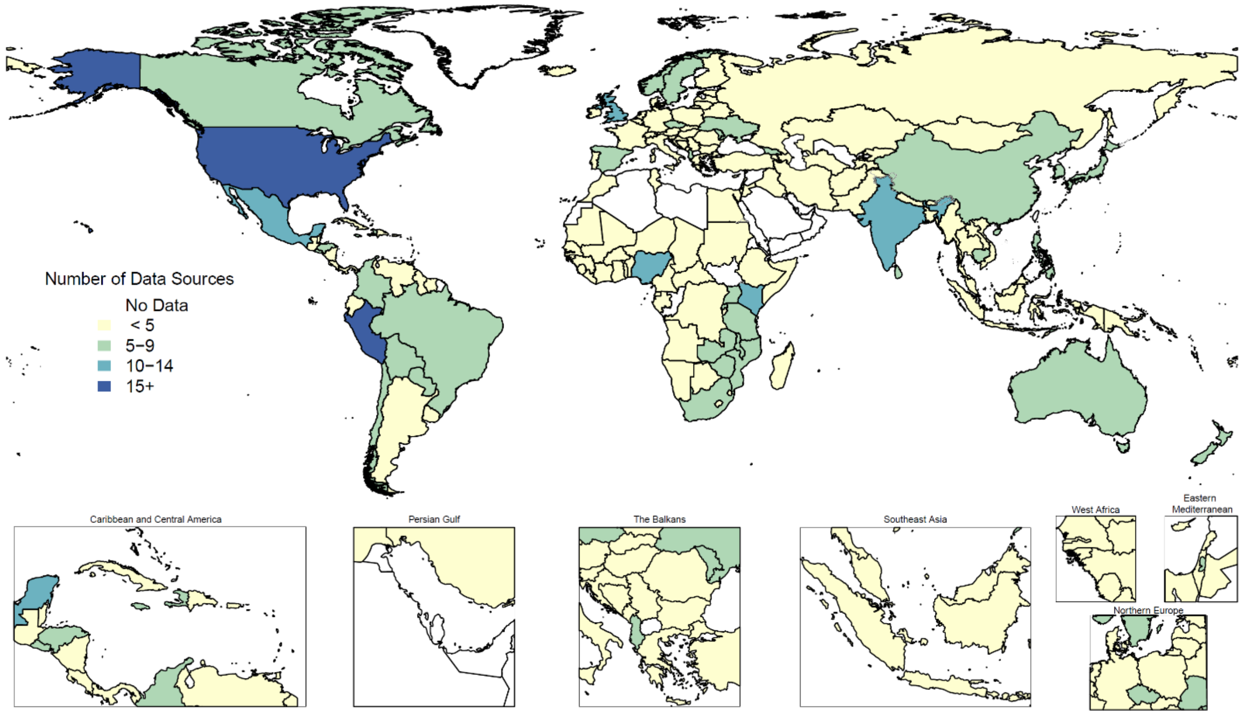

Figure S2: Year of most recent data on the prevalence of intimate partner violence

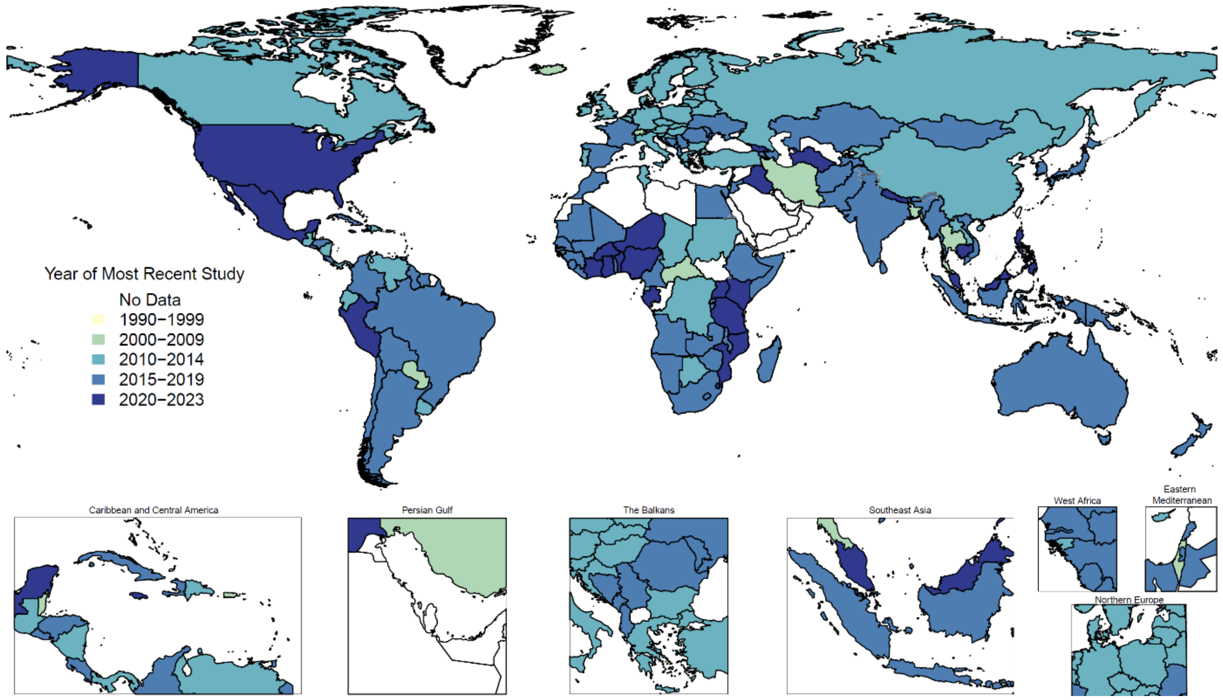

Figure S3: Data coverage for the prevalence of sexual violence against children

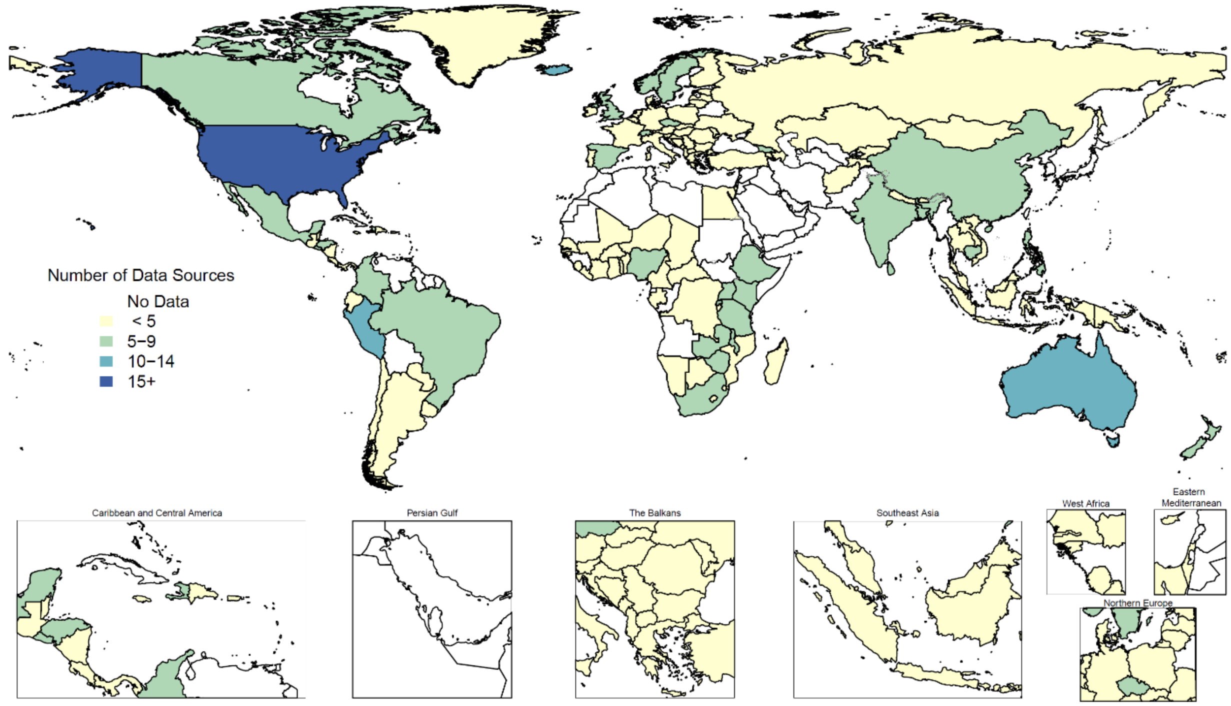

Figure S4: Year of most recent data on the prevalence of sexual violence against children

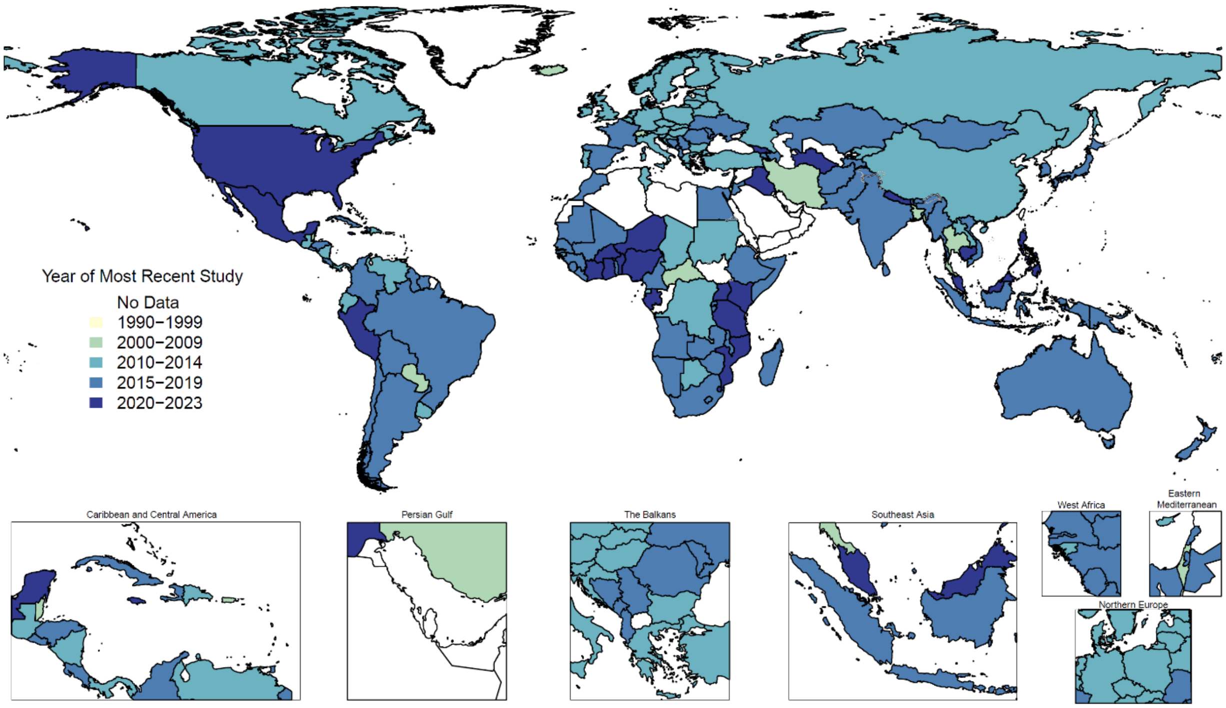

Section 2.2: Intimate partner violence exposure estimation

Model flowchart

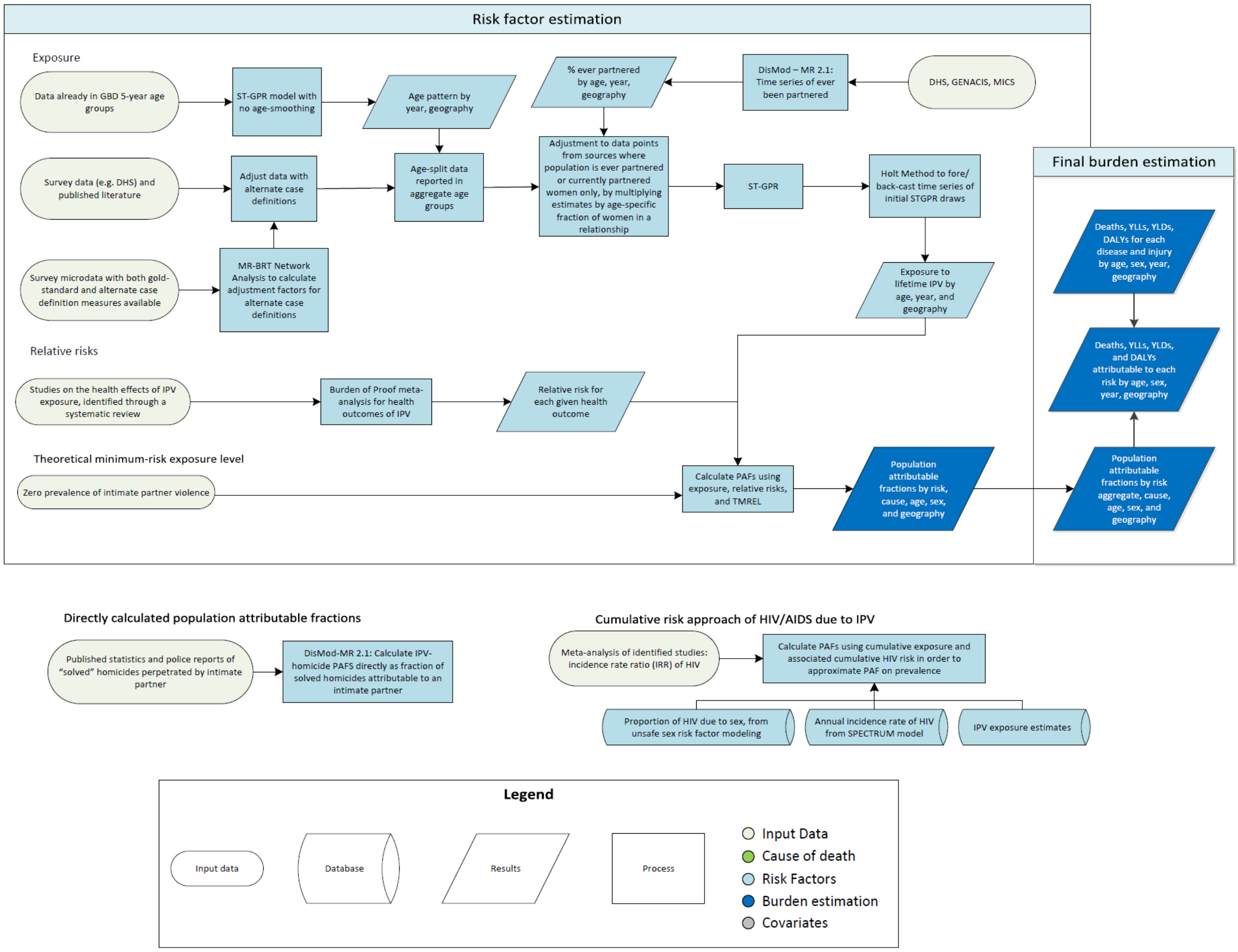

Section 2.2.1: Adjustment for non-standard case definitions

For data that reported IPV using an alternate case definition (i.e., IPV experienced in the past-year), we ran a logit-difference meta-regression with the *Meta-Regression-Bayesian, Regularized, Trimmed (*MR-BRT) tool to estimate correction factors. The MR-BRT tool is described in detail in the GBD 2021 Risk Factors Capstone[4]. To describe the method briefly, we first (1) find matched pairs of observations from the same study sample using different case definitions, (2) transform observations into logit space using the delta method; (3) calculate the difference of alternate case definitions to reference definition and calculate the standard error of the difference; and (4) use these differences as the dependent variable in a mixed effects meta-regression. The model is then used to predict adjustments needed for a non-reference definition and resultant adjustments applied to data for which only non-reference definitions are available.

After examining all available data, we identified seven alternate definitions for which sufficient data were available to inform adjustment factors:

1. Physical or sexual IPV experienced in the past 12 months
2. Physical IPV experienced over the lifetime
3. Physical IPV experienced over the past 12 months
4. Sexual IPV experienced over the lifetime
5. Sexual IPV experienced over the past 12 months
6. Severe physical IPV experienced over the lifetime
7. Severe physical IPV experienced over the past 12 months

We identified 274 studies from which multiple IPV case definitions were available, resulting in 90,714 observations informing our meta-regression. Matched pairs of observations were created within a study only (i.e., we only compared alternate definitions to reference definitions or other alternate definitions from within the same study) and by GBD age group. Our model was a network meta-analysis, meaning that we drew upon both direct comparisons made between our reference case definition and alternate case definitions, as well as indirect comparisons made between two alternate case definitions. For studies with samples representative of subnational locations as well as national locations, we only used national estimates in our model to avoid including multiple data points from the same study in our model and biasing crosswalk calculations towards locations with estimates available from multiple subnational locations. We observed that the difference between 12-month recall and lifetime recall definitions differed by abuse type (i.e., the difference in 12-month and lifetime recall was larger for physical IPV definitions when compared to sexual IPV definitions). For this reason, we treated alternate definitions as mutually exclusive, i.e., we did not assume that the difference in prevalence due to using 12-month as opposed to lifetime recall periods in the case definitions was the same regardless of which definition of violence was used (sexual, physical). Finally, we observed that the difference between IPV case definitions varied by age of respondent (i.e., the difference between 12-month and lifetime recall widens as participants get older) and therefore fit our model including a spline on age. Spline terms offer a method for incorporating non-linear relationships between a continuous variable and the model dependent variable. The model was fit using 10% trimming and two priors. Our priors assumed that (1) past 12-month prevalence would be less than lifetime prevalence of the same definition type; and (2) component definition prevalence would be less than aggregate within the same recall period (i.e., severe physical IPV over the lifetime would be less than any physical and/or sexual IPV over the lifetime).

<u>Results</u>

Betas from a spline are not directly interpretable, therefore we use dose-response plots to show the non-linear effects of our model fits and uncertainty surrounding our model predictions.

Figure S5: Estimated correction factors for alternate definitions of IPV exposure

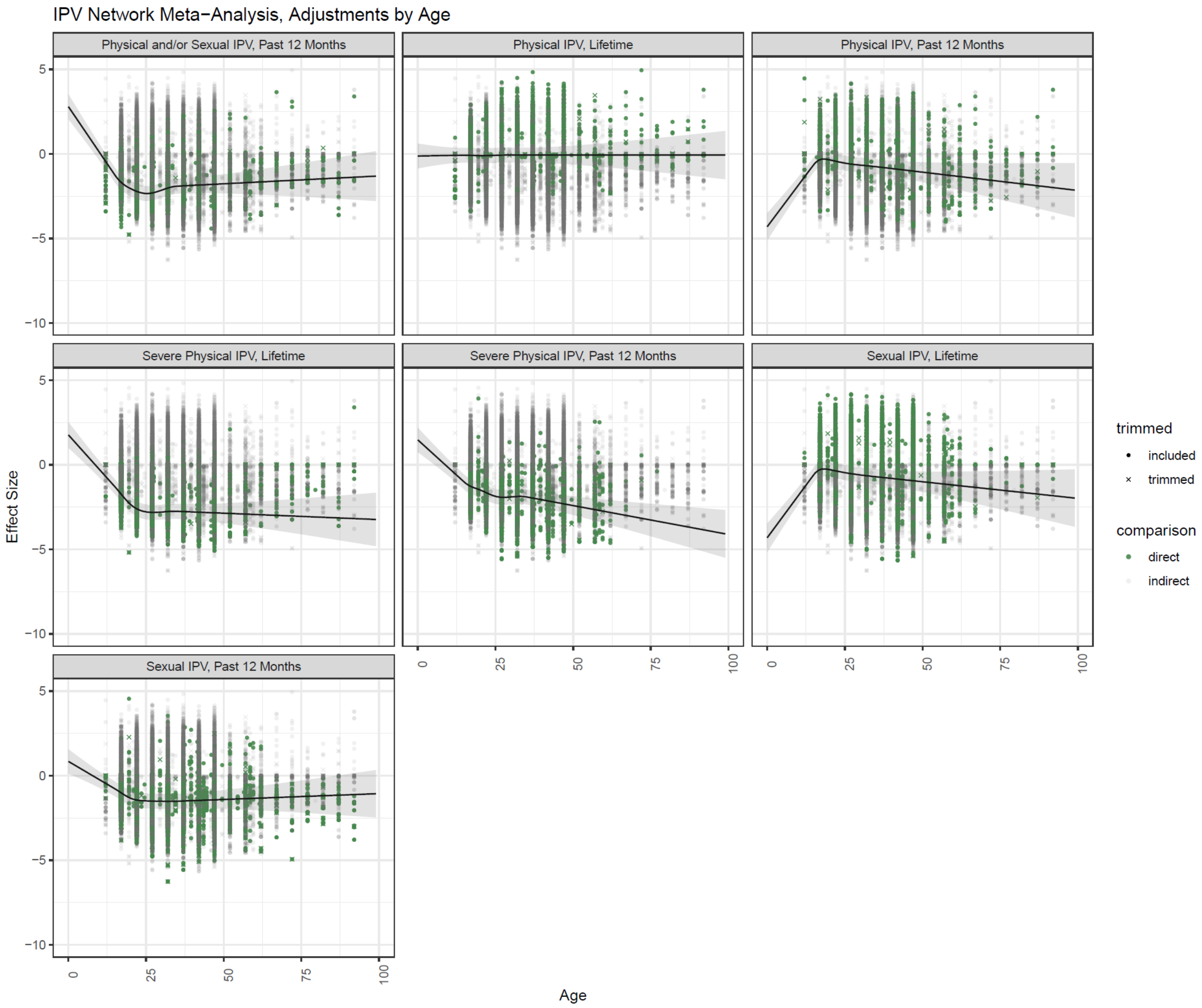

We also include scatter plots below demonstrating the effect of adjustment, when adjustments derived from our network meta-analysis are applied to data using alternate definitions that were used as inputs to the network meta-analysis.

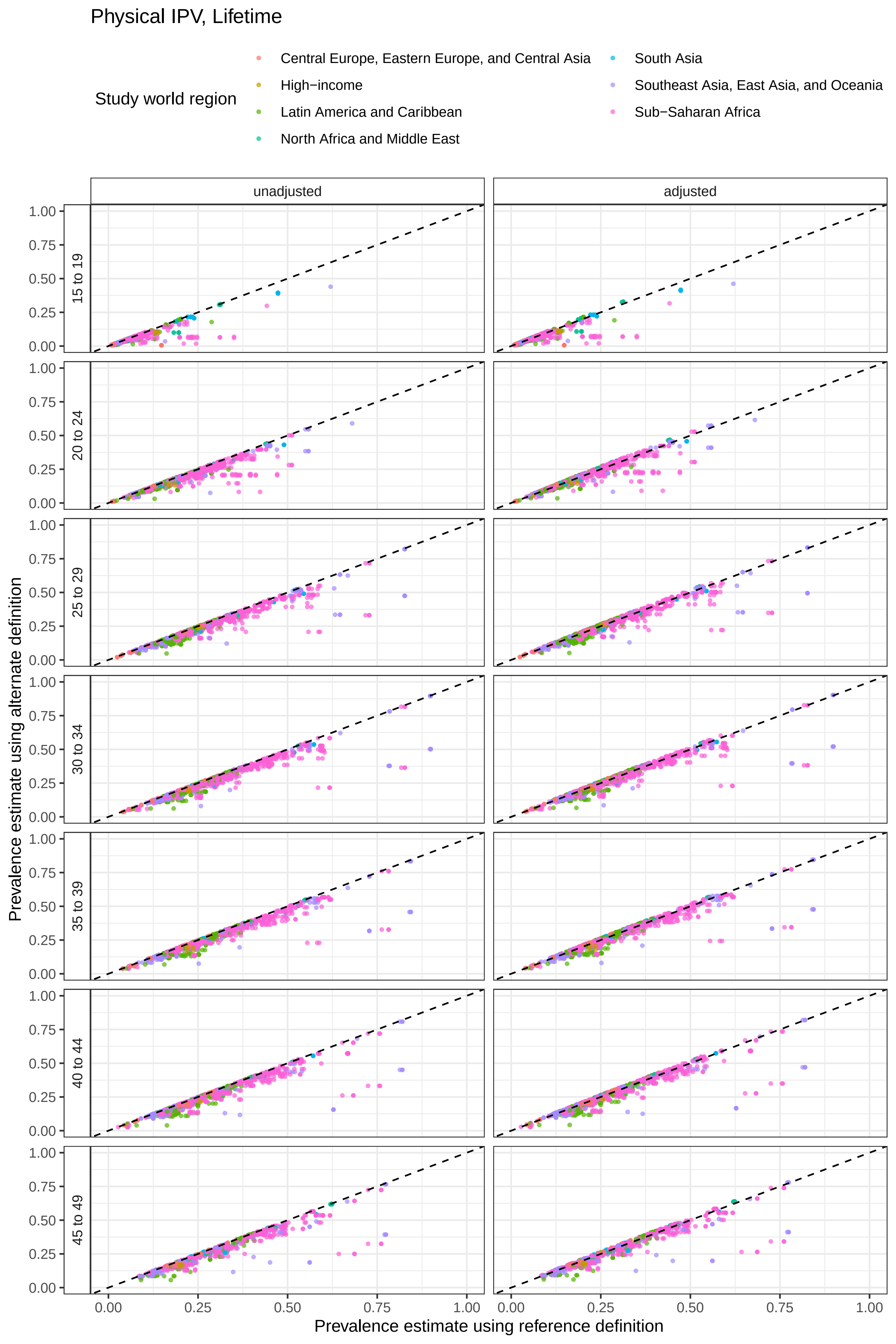
Physical IPV, Lifetime
Study world region
Central Europe, Eastern Europe, and Central Asia
High−income
Latin America and Caribbean
North Africa and Middle East
South Asia
Southeast Asia, East Asia, and Oceania
Sub−Saharan Africa
unadjusted
adjusted
15 to 19
20 to 24
25 to 29
30 to 34
35 to 39
40 to 44
45 to 49
Prevalence estimate using alternate definition
Prevalence estimate using reference definition
0.00
0.25
0.50
0.75
1.00

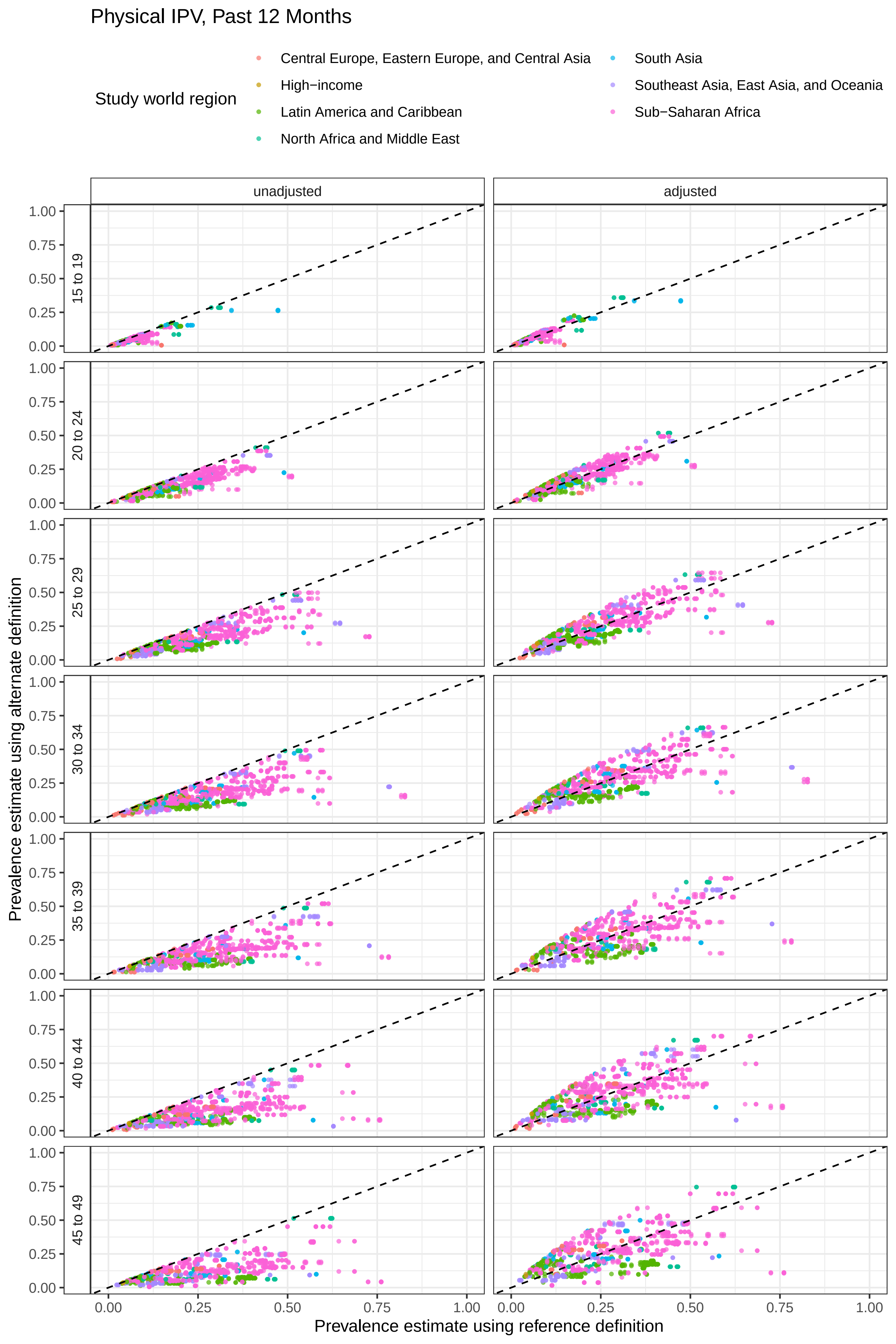

Physical IPV, Past 12 Months
Study world region
Central Europe, Eastern Europe, and Central Asia
High−income
Latin America and Caribbean
North Africa and Middle East
South Asia
Southeast Asia, East Asia, and Oceania
Sub−Saharan Africa
unadjusted
adjusted
15 to 19
20 to 24
25 to 29
30 to 34
35 to 39
40 to 44
45 to 49
0.00
0.25
0.50
0.75
1.00
Prevalence estimate using alternate definition
Prevalence estimate using reference definition

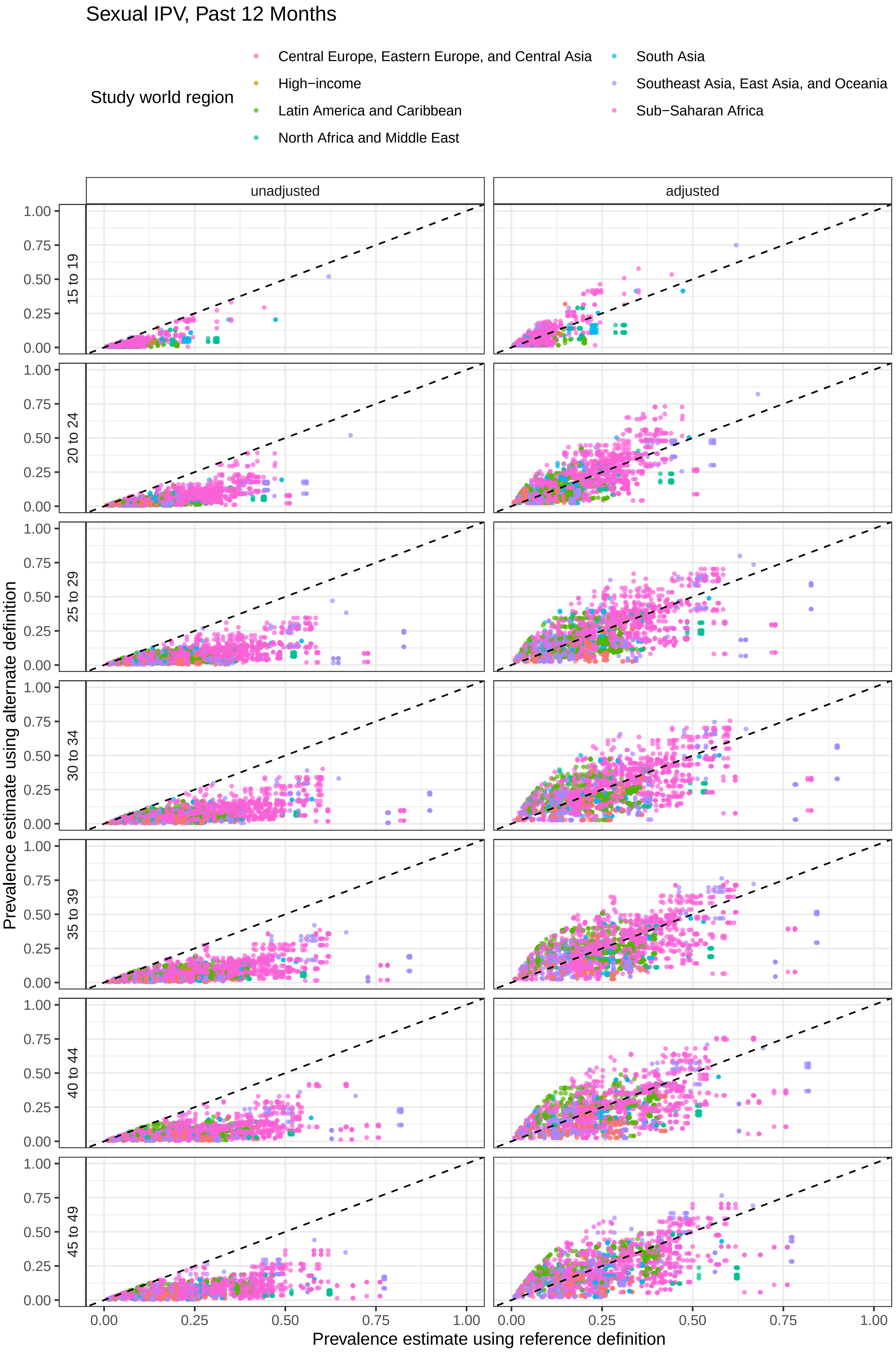

Sexual IPV, Past 12 Months
Study world region
Central Europe, Eastern Europe, and Central Asia
High−income
Latin America and Caribbean
North Africa and Middle East
South Asia
Southeast Asia, East Asia, and Oceania
Sub−Saharan Africa
unadjusted
adjusted
15 to 19
20 to 24
25 to 29
30 to 34
35 to 39
40 to 44
45 to 49
0.00
0.25
0.50
0.75
1.00
Prevalence estimate using alternate definition
Prevalence estimate using reference definition

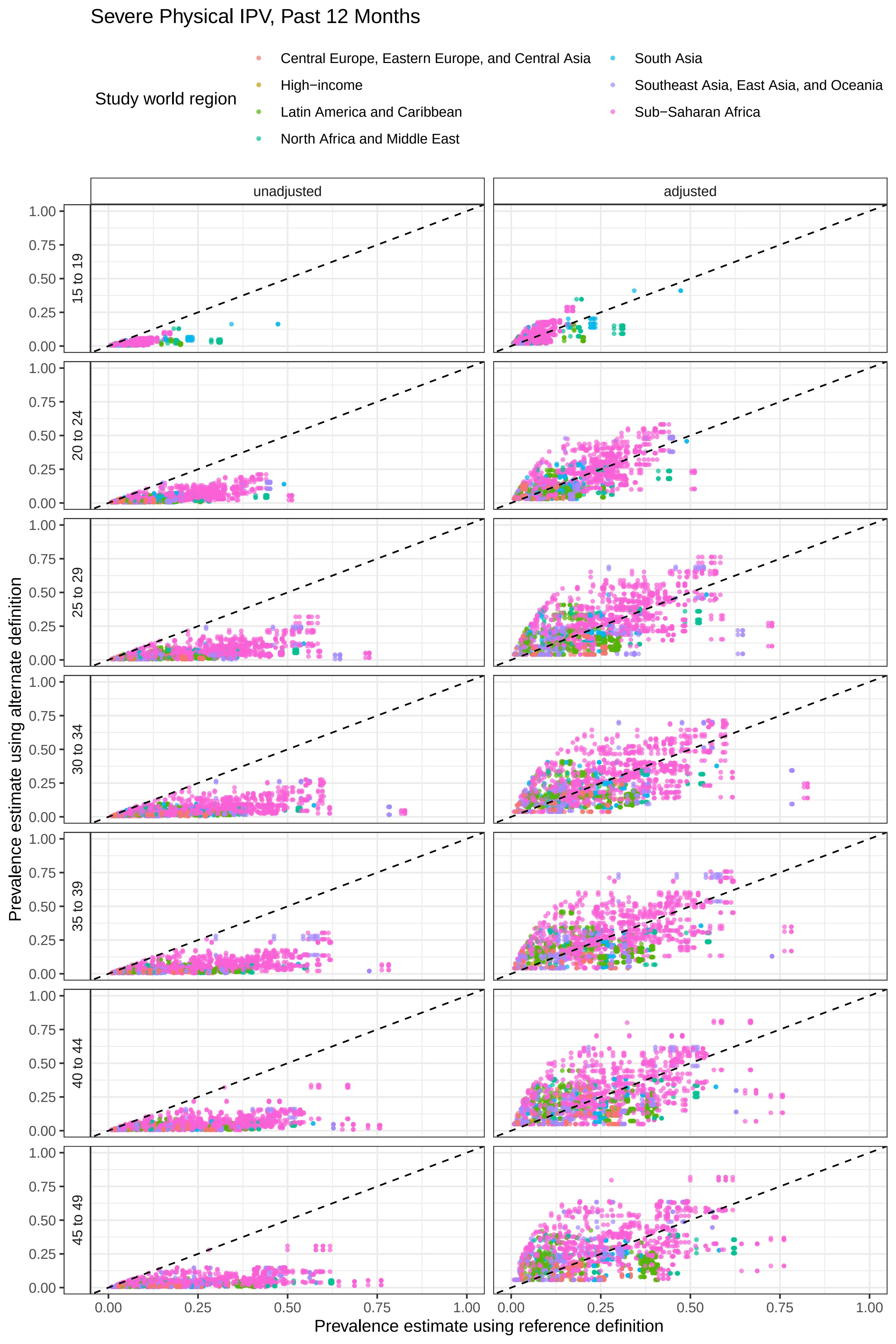
Severe Physical IPV, Past 12 Months
Study world region
Central Europe, Eastern Europe, and Central Asia
High−income
Latin America and Caribbean
North Africa and Middle East
South Asia
Southeast Asia, East Asia, and Oceania
Sub−Saharan Africa
unadjusted
adjusted
15 to 19
20 to 24
25 to 29
30 to 34
35 to 39
40 to 44
45 to 49
0.00
0.25
0.50
0.75
1.00
Prevalence estimate using alternate definition
Prevalence estimate using reference definition

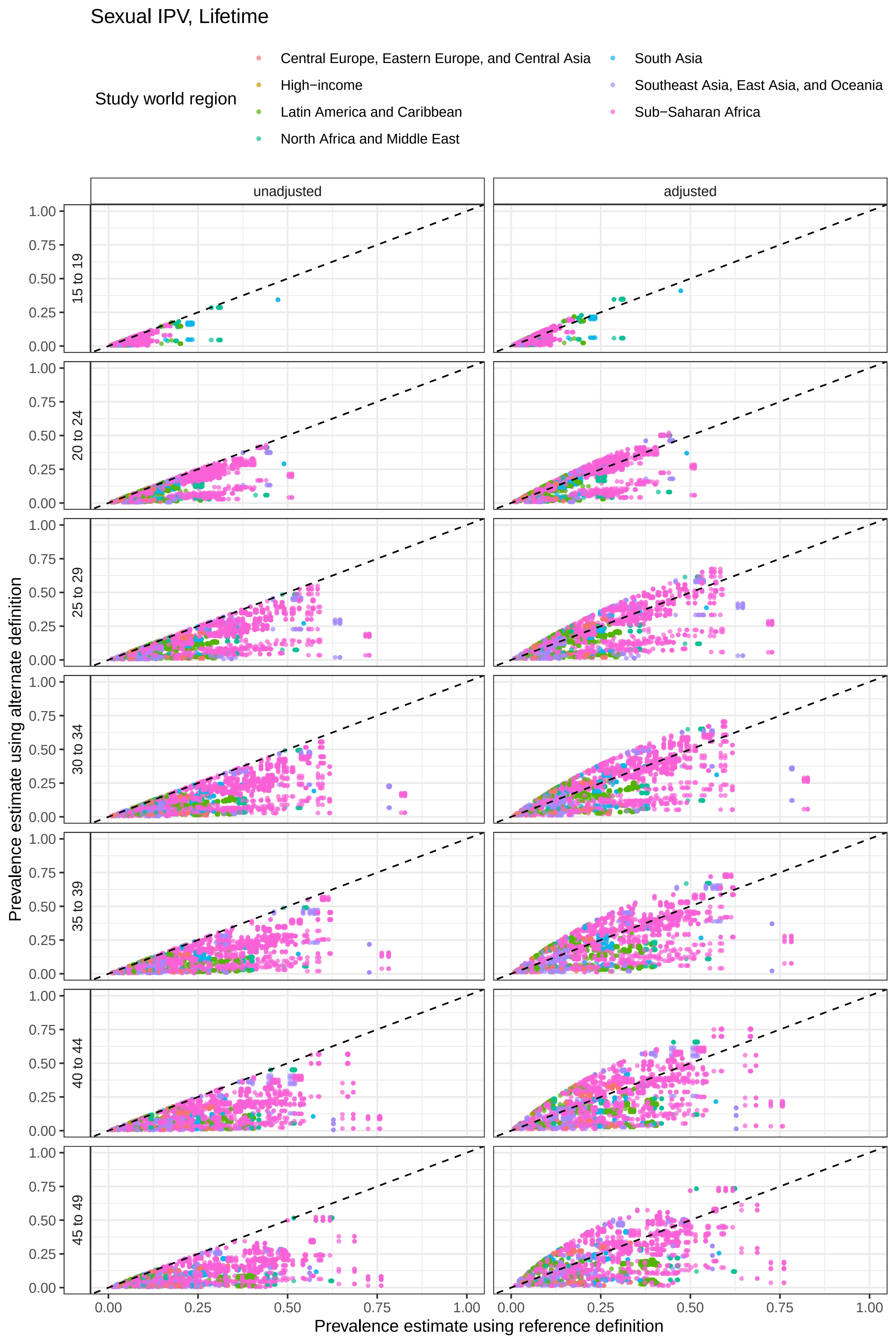

Sexual IPV, Lifetime
Study world region
Central Europe, Eastern Europe, and Central Asia
High−income
Latin America and Caribbean
North Africa and Middle East
South Asia
Southeast Asia, East Asia, and Oceania
Sub−Saharan Africa
unadjusted
adjusted
15 to 19
20 to 24
25 to 29
30 to 34
35 to 39
40 to 44
45 to 49
0.00
0.25
0.50
0.75
1.00
Prevalence estimate using alternate definition
Prevalence estimate using reference definition

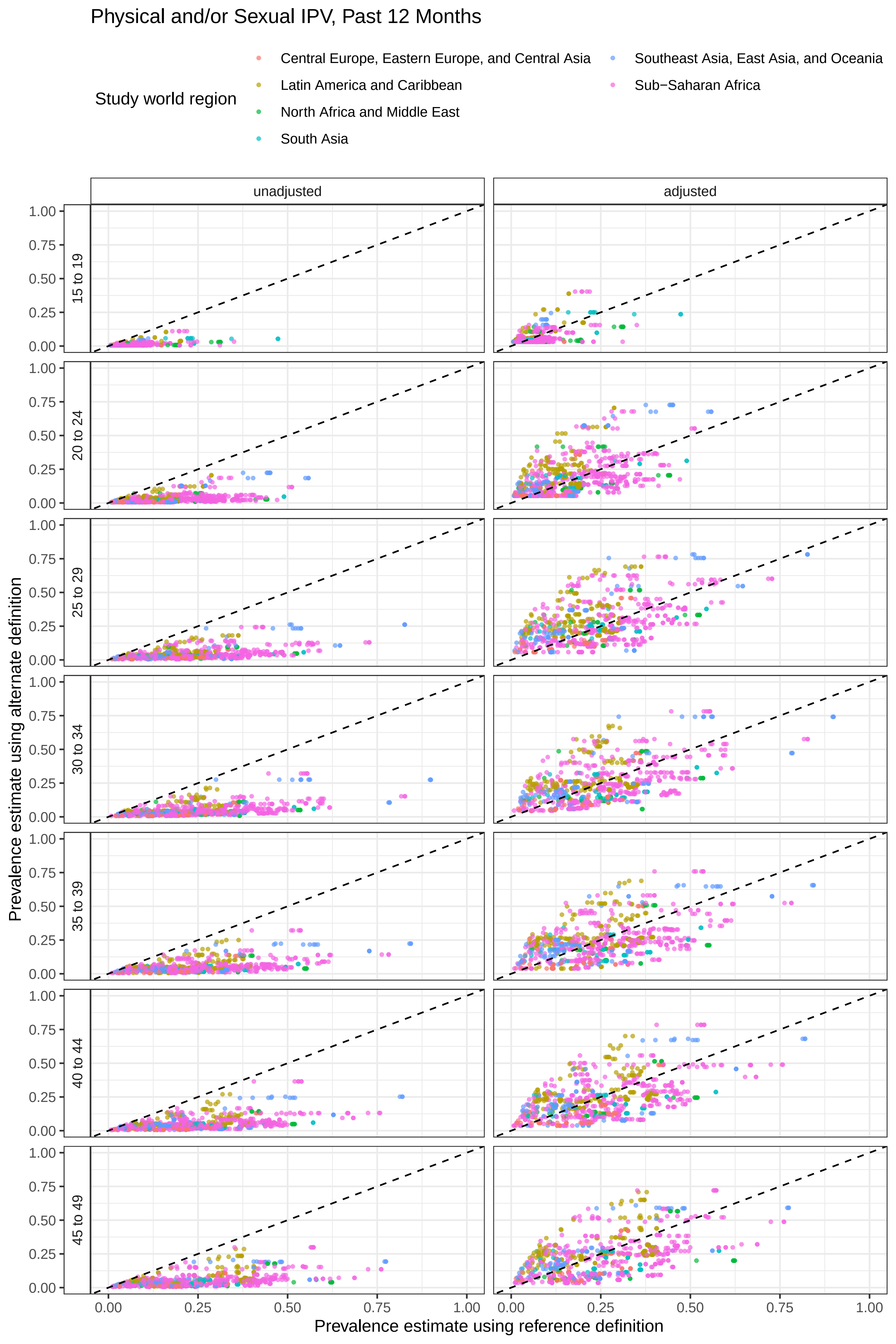

Physical and/or Sexual IPV, Past 12 Months
Study world region
Central Europe, Eastern Europe, and Central Asia
Latin America and Caribbean
North Africa and Middle East
South Asia
Southeast Asia, East Asia, and Oceania
Sub−Saharan Africa
unadjusted
adjusted
15 to 19
20 to 24
25 to 29
30 to 34
35 to 39
40 to 44
45 to 49
Prevalence estimate using alternate definition
Prevalence estimate using reference definition
0.00
0.25
0.50
0.75
1.00

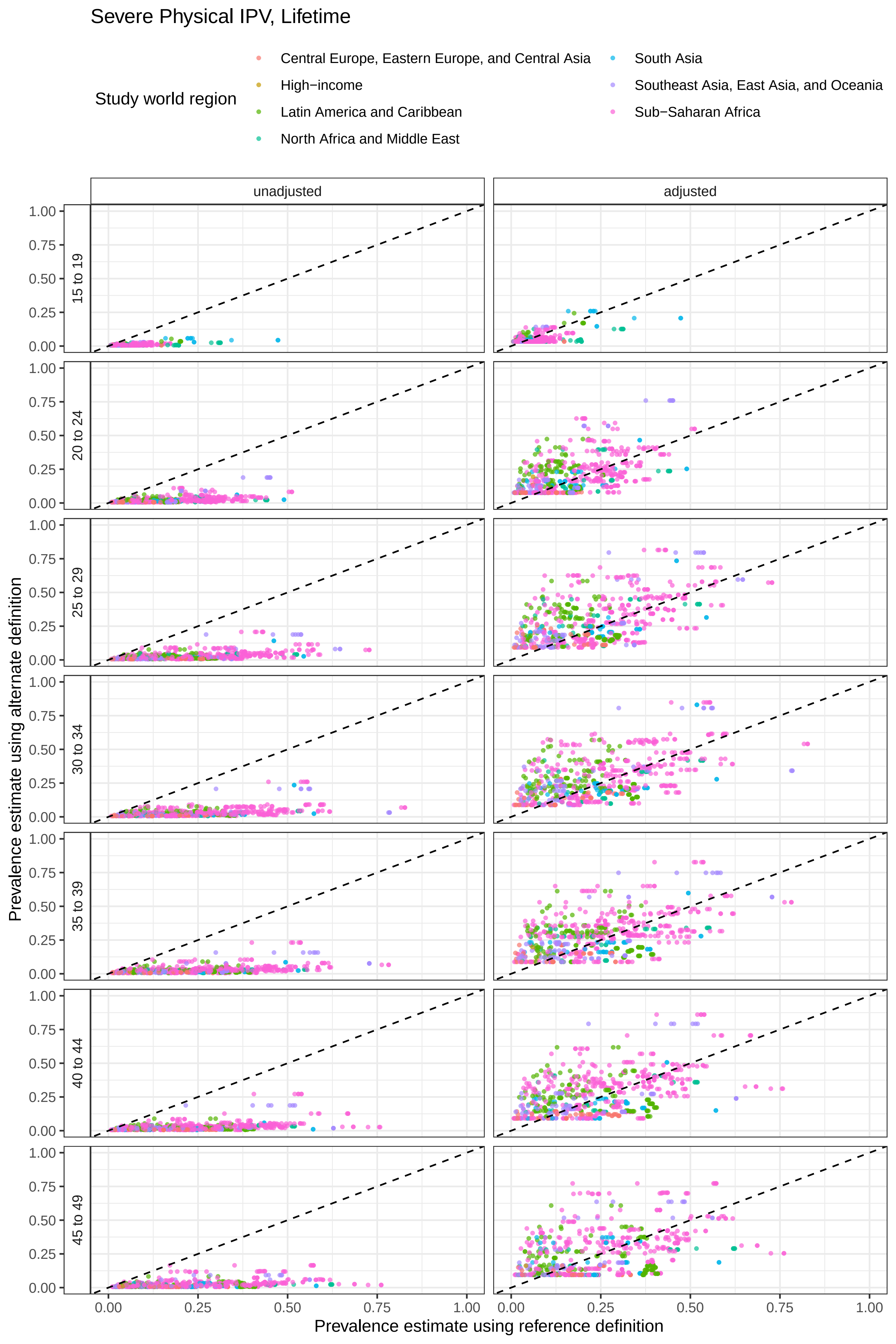

Severe Physical IPV, Lifetime
Study world region
Central Europe, Eastern Europe, and Central Asia
High−income
Latin America and Caribbean
North Africa and Middle East
South Asia
Southeast Asia, East Asia, and Oceania
Sub−Saharan Africa
unadjusted
adjusted
15 to 19
20 to 24
25 to 29
30 to 34
35 to 39
40 to 44
45 to 49
Prevalence estimate using alternate definition
Prevalence estimate using reference definition
0.00
0.25
0.50
0.75
1.00

Section 2.2.2: Age-splitting

We split data reported in broader age groups than the GBD five-year age groups by adapting the method reported in Ng et al[5]. to split aggregate data using a reference age pattern. We divided the data into two sets: (1) a training dataset, containing data that already fell into GBD five-year age groups, and (2) a split dataset, which contained data reported in aggregate age groups broader than GBD five-year bins. We then used spatiotemporal Gaussian process regression (ST-GPR) to estimate geography-time-specific age patterns using the training dataset. Full details on the ST-GPR method are described in detail elsewher[4]. The ST-GPR model used an age-weight parameter value that minimised the effect of any age smoothing within the model. This parameter choice allowed the estimated age pattern to be driven by data rather than enforced by smoothing parameters of the model. Due to data sparsity within the training dataset, estimated geography-age patterns were aggregated to the GBD region level. The age pattern from the GBD region with the most training datapoints (south Asia) was used to adjust data reported in aggregated age groups.

Section 2.2.3: Adjustment for estimates among ever-partnered women only

To correct for studies reporting IPV prevalence out of only ever or currently partnered women, we multiplied estimates from these studies by the age-specific fraction of women who had ever been partnered. We generated ever-partnered estimates using MICS and DHS data in a single parameter DisMod-MR 2.1 model to reflect the most recent data on proportion of women who have ever been partnered. DisMod-MR 2.1 is described in detail in the GBD 2021 Risk Factors Capstone[4]. For studies restricting the perpetrator to spouses or current spouses, due to insufficient data comparing our reference and alternate populations in specific age-location-years, we refrained from calculating under-informed correction factors.

Section 2.2.4: Prevalence modelling

ST-GPR

We used ST-GPR to model lifetime IPV prevalence. Input data were prepared by first adjusting data with alternate case definitions and then splitting data into GBD standard five-year age groups by applying modelled reference age patterns, as described above. Full details on the ST-GPR method are described in detail in another publication[4]. Briefly, the mean function input to GPR is a complete time series of

estimates generated from a mixed effects hierarchical linear model plus weighted residuals smoothed across time, space, and age. The linear model formula for IPV is:

$$logit(p_{g,a,t}) = \beta_0 + \sum_{k=1}^{18} \beta_k I_{A[a]} + \alpha_s + \alpha_r + \alpha_g + \epsilon_{g,a,t}$$

Where, $I_{A[a]}$ is a dummy variable indicating specific age group that the prevalence point captures, and α_s, α_r, and α_g are super-region, region, and geography random intercepts, respectively. Random effects were used in model fitting but not in prediction.

Holt's linear trend method

Data sparsity within the IPV model caused poor model fits over time. Thus, we introduced Holt's linear trend method (extended simple exponential smoothing) to forecast and back-cast draws from the initial ST-GPR model. Holt's linear trend method allows forecasting of data with a linear trend using a weighted average of past observations, with weights decaying exponentially as observations get older[6]. We applied this method to location-age-specific draws from our initial ST-GPR model, with the year range of the ST-GPR draws to be used as the initial time series defined based upon location-age data availability. For location-age combinations with available data spanning more than three years, draws were bounded from the minimum year to the maximum year of location-age-specific data. Otherwise, draws were bounded from the minimum year to the maximum year of super-region-age-specific data. To avoid over-forecasting for longer time periods (i.e., in locations where only very old data were available), we used a damping parameter (phi = 0.9) to enforce a zero-slope linear trend over time. Finally, due to our adjustment to ST-GPR draws we needed to re-enforce consistency between subnational and national means, so we logit-raked subnational draws to fit national means for countries with subnational estimation.

Section 2.3: Sexual violence against children exposure estimation

The details of our SVAC prevalence estimation have been previously published[7]. We additionally describe them here.

Model flowchart

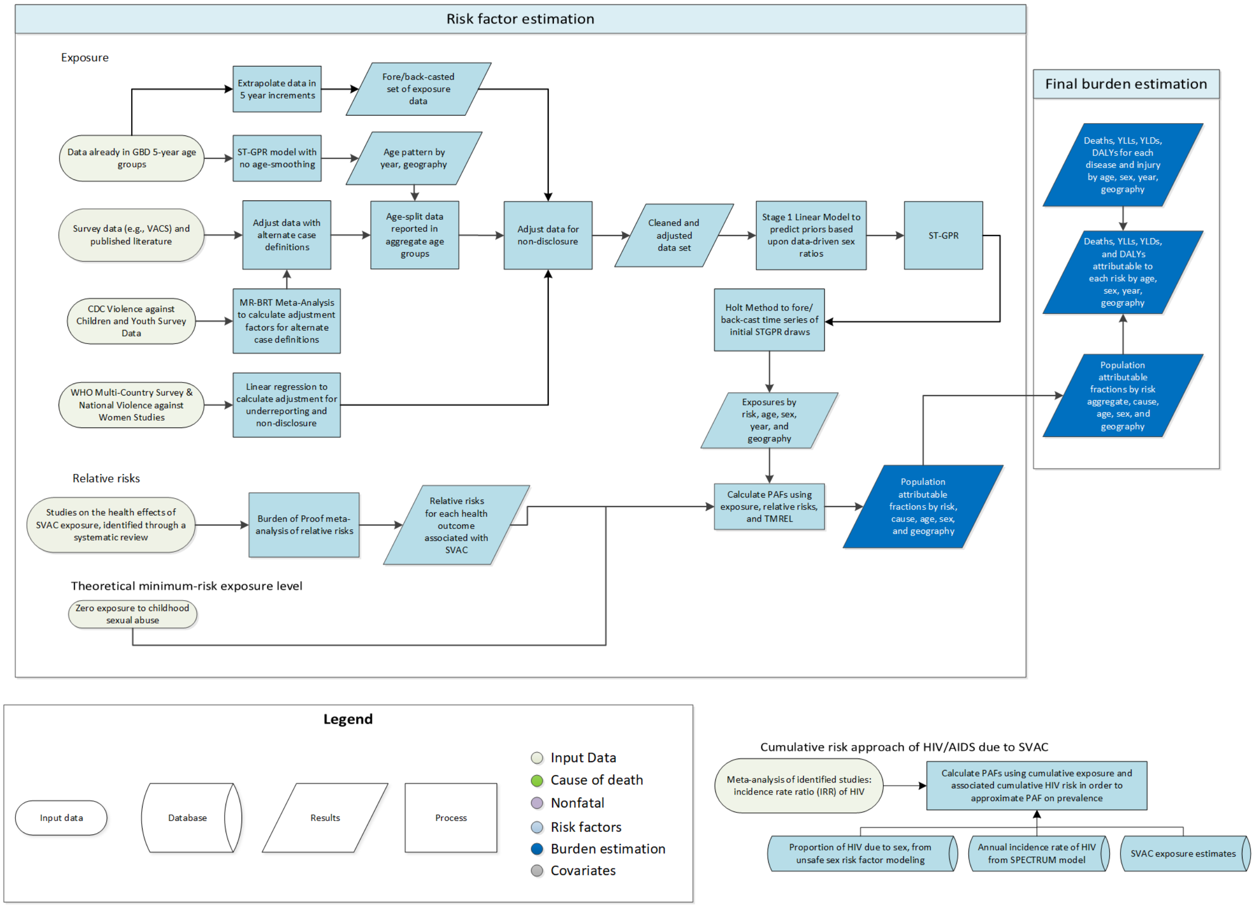

Section 2.3.1: Adjustment for non-standard case definitions

For alternate case definitions of SVAC, we used high-quality data from the CDC Violence Against Children and Youth Surveys[8] (n=15) to run a logit-difference meta-regression with the MR-BRT tool to estimate correction factors. While we originally intended to fit separate models for each sex, we decided to model one set of crosswalk adjustments as we did not have rich data to inform significant differences between the sexes and observed implausible differences in adjustments made to the same source between the sexes when modelling adjustment factors separately. Our models were fit using 10% trimming and two priors, 1) contact-only (reference) definitions should be less than definitions including contact or non-contact cases, and 2) intercourse-only definitions be less than contact-only (reference) definitions. Due to

insufficient data, over-adjustment issues and other limitations, we did not calculate an adjustment factor for data with the restricted perpetrator or sexual debut alternate definitions. Adjustment factors are presented in Table S5.

Table S5: Estimated adjustment factors for alternate definitions of SVAC exposure

**Data input**	**Reference or alternative case definition**	**Gamma**	**Beta coefficient, logit (SD)***	**Adjustment factor****
Contact only SVAC	Ref	0.0134	---	---
Contact or non-contact SVAC	Alt		0.4343 (0.0230)	1.5439
Intercourse-only SVAC	Alt		-0.4263 (0.0144)	0.6529
SVAC before age 15	Alt		-0.4551 (0.0116)	0.6344
SVAC before age 12	Alt		-1.2791 (0.0134)	0.2783

**MR-BRT crosswalk adjustments can be interpreted as the factor the alternative case definition is adjusted by to reflect what it would have been had it been measured using the reference case definition. If the logit beta coefficient is negative, then the alternative is adjusted up to the reference. If the logit beta coefficient is positive, then the alternative is adjusted down to the reference.*

***The adjustment factor column is the exponentiated beta coefficient. For logit beta coefficients, this is the relative odds between the two case definitions.*

Section 2.3.2: Adjustment for non-disclosure by mode of survey delivery

For SVAC, we also accounted for differential reporting, recognizing that some people might not directly disclose experiences of SVAC to an enumerator. To calculate this adjustment factor, we used data from the WHO Multi-country Study on Women's Health and Domestic Violence against Women[9] and 25 national violence against women surveys, all of which measured female SVAC using face-to-face interviews and anonymous self-report cards. Respondents were given two opportunities to disclose if someone had ever touched them sexually or made them do something sexual that they did not want to do during childhood. First, respondents verbally replied to the question so that interviewers could mark the response. Later, at the end of the interview, respondents were given another, more private opportunity to reply to the question using a card with pictorial representations of "yes" and "no." Preliminary results from the WHO Multi-country Study and its national adaptations suggest that the private self-report card was usually able to ascertain more cases of SVAC than the interview alone. A simple linear regression

model was run to estimate the relationship between prevalence estimates derived from each administration method:

$$y_i = mx_i + b$$

where:

- x_i is the prevalence of SVAC derived from the traditional face-to-face interview
- y_i is the prevalence of SVAC derived from private self-report cards
- i is a unique study identifier

We estimated an intercept (b) of 0.058 and a slope (m) of 1.049, and we used these values to predict what prevalence would be in a private, self-report survey given the prevalence from a face-to-face interview. However, our face-to-face input data did not extend beyond 25%, limiting our understanding of reporting patterns in studies already reporting relatively high prevalence estimates. As such, we do not apply the linear shift (intercept) to input values equal to or greater than 25% prevalence. Instead, we only use the modelled slope to relatively increase those values:

$$y_i = \begin{cases} mx_i + b, & x_i < 0.25 \\ mx_i, & x_i \geq 0.25 \end{cases}$$

Section 2.3.3: Age-splitting

We split data reported in age groups broader than the standard GBD five-year age groups by adapting the method reported in Ng et al.[5] to split aggregate data using a reference age pattern. We divided the data into two sets: (1) a training dataset, containing data that already fell into GBD five-year age groups, and (2) a split dataset, which contained data reported in aggregate age groups broader than GBD five-year bins. We then used spatiotemporal Gaussian process regression (ST-GPR) to estimate geography-time-specific age patterns using the training dataset. The ST-GPR model used an age-weight parameter value that minimised the effect of any age smoothing within the model. This parameter choice allowed the estimated age pattern to be driven by data rather than enforced by smoothing parameters of the model. Due to data sparsity within the training dataset, estimated geography-time age patterns were aggregated to the GBD region level. For female SVAC, the age pattern from the GBD world region with the most training datapoints (high-income North America) was used to adjust all non-standard age data. Due to extreme data sparsity within the male SVAC model, even the aggregated regional age-pattern for the GBD region with the most training datapoints (high-income North America) was unrealistically variable

across neighbouring age groups. Therefore, countries within this region were visually examined and the most stable age pattern (Canada) was selected to adjust non-standard age data.

Section 2.3.4: Cohort extrapolation

In addition to the adjustment steps described above, we introduced and applied a cohort extrapolation step to the input data prior to modelling. The case definition of SVAC pertains to experiences occurring in childhood, and adults aged 18 and older have completed the window during which new cases can occur. We therefore extrapolated data reported in the standard 5-year age groups (beginning at age 20) in 5-year increments through time. For example, the prevalence reported by a given survey for 25–29-year-olds in 2015 can also be considered as an estimate of SVAC prevalence for 20–24-year-olds in 2010 and for 30–34-year-olds in 2020. In recognition of demographic changes over time as well as SVAC's association with adverse health outcomes, both of which might influence prevalence, the uncertainty around extrapolated data points were inflated by a factor of 2, which consequently down-weights them in future modelling steps. We did not apply the same assumption or extrapolation to data points representative of 10–19-year-olds, as respondents below the age threshold detailed in the SVAC case definition are still at risk of experiencing violence. Thus, their prevalence is subject to change over time and cannot be subject to the same assumptions described above.

Section 2.3.5: Prevalence modelling

ST-GPR

Separate models were run for males and females. However, in our first stage of ST-GPR modelling, we fit a custom linear regression model that predicted SVAC trends using data from both males and females, allowing the global sex ratio of SVAC exposure to inform the priors of consequent modelling stages in each sex-specific model. The complete time series of first-stage estimates plus weighted residuals was then used as a mean function in a Gaussian process regression.

The stage one linear model formula is as follows:

$$logit(p_{g,a,t}) = \beta_0 + \beta_1 S_{A[a],g,t} + \sum_{k=2}^{20} \beta_k I_{A[a]} + \alpha_s + \alpha_r + \alpha_g + \epsilon_{g,a,t}$$

Where $S_{A[a],g,t}$ is the sex of the prevalence point by specific age group A, geography g, and time t, $I_{A[a]}$ is a dummy variable indicating specific age group A that the prevalence point $p_{g,a,t}$ captures, and α_s, α_r, and

α_g are super-region, region, and geography random intercepts, respectively. Random effects were used in model fitting and prediction.

Holt's linear trend method

Data sparsity within the SVAC models caused poor model fits over time. Thus, we introduced Holt's linear trend method (extended simple exponential smoothing) to fore- and back-cast draws from the initial ST-GPR model. Holt's linear trend method allows forecasting of data with a linear trend using a weighted average of past observations, with weights decaying exponentially as observations get older[6]. We applied this method to location-age-specific draws from our initial ST-GPR model, with the year range of the ST-GPR draws to be used as the initial time series defined based upon location-age data availability. For location-age combinations with available data spanning more than three years, draws were bounded from the minimum year to the maximum year of location-age-specific data. Otherwise, draws were bounded from the minimum year to the maximum year of super-region-age-specific data. For male SVAC, there is one super-region (north Africa and the Middle East) for which we have no data. In this case, we preserved most of the ST-GPR fit by using the time range of 1990–2019 (i.e., forecasting only 2019–2023). To avoid over-forecasting for longer time periods (i.e., in locations where only very old data were available), we used a damping parameter (phi = 0.9) to enforce a zero-slope linear trend over time. Finally, due to our adjustment to ST-GPR draws, we needed to re-enforce consistency between subnational and national means, so we logit-raked subnational draws to fit national means for countries with subnational estimation.

Section 3: Risk outcome pair identification and modelling

Section 3.1: Systematic review of scientific literature

Our original systematic review and health outcomes associated with exposure to IPV and SVAC has been described in detail in Spencer et al[10], and more details on search strings, article screening and data extraction procedures, relative risk estimation methodology can be found in this publication. The original search encompassed evidence published 1 January 1970 and 31 January 2023. In this study, we extended the original search results by including updated searches that encompass newly published evidence from 1 February 2023 to 31 January 2024. Studies newly identified in the search update and which met inclusion criteria for IPV and SVAC were extracted in line with the established systematic review procedures (described in detail in Spencer et al[10]) and incorporated into updated models of relative risk. We briefly describe the search parameters and relative risk estimation methodology here.

Section 3.1.1: Search parameters

The systematic review search strategy was designed to identify case-control, cohort, or case-crossover studies conducted in generalizable participant groups and which reported a relative measure of association between violence exposure and health. Because the review aimed to identify all health outcomes associated with exposure to IPV and/or SVAC, search strings were not restricted to predefined health outcomes. Search strings can be found in Spencer et al[10]. Throughout article screening and selection, GBD health outcome case definitions were used to evaluate acceptable outcomes. We accepted study definitions of IPV and SVAC exposure which aligned with GBD reference definitions and predefined alternative definitions.

Across the original and updated search time period (1 January 1970 to 31 January 2024), a total of 75,331 articles were identified, of which 4,446 met inclusion criteria during title/abstract screening. Of these, 36 studies reporting on health impacts of IPV and 114 studies reporting on health impacts of SVAC were accepted and extracted after full text screening. Primary search results were additionally supported by citation searching other systematic reviews/meta-analyses for additional references (this process is described in detail in Spencer et al[10]).

Section 3.1.2: Screening process

We used the criteria listed below to title and abstract screen the identified articles. Each review step (title/abstract screening, full-text screening, and data extraction) began with consensus building exercises across the review team. After training and consensus-building, the first two-thirds of titles/abstracts were

reviewed by two independent reviewers, with conflicts resolved by project leaders. Upon confirmation of a low rate of total conflicts (<5% of total screened), the remainder of titles/abstracts were single screened. Non-English articles were screened by reviewers with proficiency in the language. Studies which met inclusion criteria during title/abstract screening were full text screened and excluded if found to meet any exclusion criteria. Two independent reviewers full text screened 10% of articles, with conflicts resolved by project leads. Upon confirming a low conflict rate (<5%), the remaining 90% of articles were single screened.

Section 3.1.3: Inclusion criteria

Study design: case–control, cohort, or case-crossover studies.

Participants: Studies conducted in participant groups likely to be generalizable to the population of interest. Exposed groups are defined as any individual who has experienced a form of Gender-based Violence (GBV) and/or Violence against Children (VAC) throughout the lifetime. Comparators will be non-exposed control groups, or study groups without reported exposure to a form of GBV and/or VAC.

Outcomes: Studies reporting an estimate of association (either RR, risk ratio, odds ratio, hazard ratio or similar) or reporting cases and non-cases among those exposed and unexposed. If not provided directly, studies providing enough information to allow an estimate of RR to be calculated will meet inclusion criteria.

Section 3.1.4: Exclusion criteria

Study design: Cross-sectional, ecological, case series or case studies.

Participants: Studies conducted in subgroups identified only by convenience sampling or subgroups identified via a shared characteristic that is likely related to risk of exposure to violence or the reported health outcome (e.g., domestic violence shelter residents).

Exposure measurement: Studies that report only an aggregate measure of exposure combining exposure to a form of violence with other, non-eligible exposures (e.g., reports a composite ACE score only) will be excluded. For these studies, we are unable to disentangle the effect of violence exposure from the effects of other hardships or exposure types, preventing their inclusion in our review.

Does not meet minimum reporting criteria: Studies missing essential data, that is, those that do not report effect sizes and uncertainty information (confidence intervals, sample sizes) or the data needed to impute an effect size with uncertainty information.

Section 3.1.5: PRISMA diagram

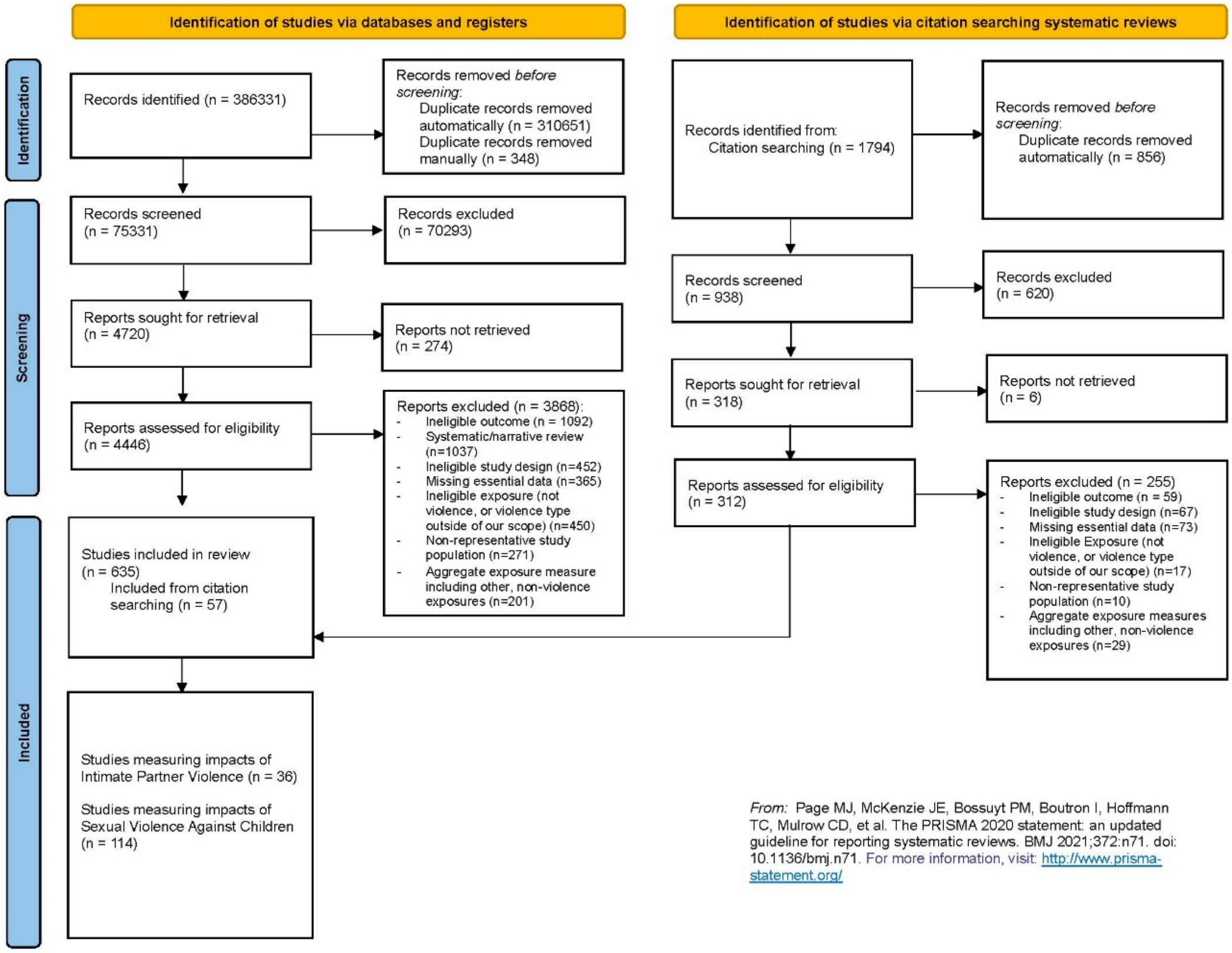

From: Page MJ, McKenzie JE, Bossuyt PM, Boutron I, Hoffmann TC, Mulrow CD, et al. The PRISMA 2020 statement: an updated guideline for reporting systematic reviews. BMJ 2021;372:n71. doi: 10.1136/bmj.n71. For more information, visit: http://www.prisma-statement.org/

Section 3.2: Relative risk estimation

Section 3.2.1: Burden of Proof Risk Factor methodology

We used the Burden of Proof Risk Factor methodology[11] to estimate the risk of health outcomes in association with exposure to IPV and SVAC. This method is the GBD standard for assessing the strength of evidence underlying relationships between risk factors and health outcomes and has been applied to many risk factors, including smoking, red meat consumption, and other forms of violence against children and gender-based violence[12–15]. The BPRF approach employs MR-BRT to estimate relative risks and uncertainty estimates that incorporate between-study heterogeneity. Drawing upon all available input data identified in the updated systematic review, we investigated health outcomes for which at least three studies had been identified for IPV and/or SVAC and performed meta-regression analyses and estimated the risk of the selected health outcomes given exposure to IPV or SVAC. Seven health outcomes (major depressive disorder, maternal abortion and miscarriage, HIV/AIDS, drug use disorders, anxiety disorders, maternal hemorrhage, and self-harm) met the three-study threshold for IPV. Sixteen health outcomes (self-harm, alcohol use disorders, major depressive disorder, asthma, bipolar disorder, diabetes mellitus type 2, HIV/AIDS, drug use disorders, sexually transmitted infections excluding HIV, maternal abortion and miscarriage, anxiety disorders, conduct disorder, schizophrenia, bulimia nervosa, anorexia nervosa, and ischemic heart disease) met the three-study threshold for SVAC.

Following burden of proof methodology, we estimated the burden of proof risk function (BPRF), defined as the fifth quantile estimate of the risk closest to the null estimate and reflecting the most conservative estimate of the harmful association between IPV and SVAC and the selected outcomes that is consistent with all available evidence. This process followed five main analytical steps. First, we estimated a pooled relative comparing the risk of health outcomes to individuals exposed to the given risk factor relative to non-exposed individuals. Second, we evaluated and adjusted for systematic sources of bias within input studies. We extracted risk of bias criteria in accordance with the Grading of Recommendations, Assessment, Development and Evaluations (GRADE) approach[16]. Binary covariates were created to capture each risk of bias criterion, these have been described in further detail in Spencer et al[10]. We tested the potential effect of each covariate using MR-BRT's automated covariate selection process, which uses a Lasso strategy to identify statistically significant covariates and adjusts for these selected covariates within the final relative risk model. Third, we quantified between-study heterogeneity such that the final uncertainty estimate reflects both the posterior uncertainty corresponding to the fixed effect and the 95th quantile of gamma[11]. Fourth, we detected potential publication bias using Egger's regression test. Finally, we estimated the BPRF, defined as the fifth quantile estimate of the risk closest to the null estimate and

reflecting the most conservative estimate of the harmful association between IPV and SVAC and the selected outcomes that is consistent with all available evidence. The BPRF is calculated by combining uncertainty of the mean, estimated between-study heterogeneity (γ), and the 95th quantile of γ obtained from the fisher information matrix.

Risk outcome scores (ROS) are calculated from the BPRF. The risk-outcome score (ROS) summarizes the effect of the risk factor on the health outcome under study and is calculated as the absolute value of the log BRPF divided by two:

$$ROS = \frac{|\log{(BPRF)}|}{2}$$

From the log BPRF, we can also calculate a measure of excess risk:

$$Minimum\ percent\ increase\ in\ risk = (BPRF - 1) * 100\%$$

ROS can be categorized into star rating categories ranging from zero to five (one star, ≤0.0 ROS; two stars, >0.0–0.14 ROS; three stars, >0.14–0.41 ROS; four stars, >0.41–0.62 ROS; and five stars, >0.62 ROS). Risk-outcome pairs receiving a one- to five-star rating are eligible for inclusion in GBD. Greater star ratings signify greater strength of evidence for and magnitude of association between risk and outcome. All one- to five-star-rated risk-outcome pairs resulting from the updated systematic review and BRPF analysis were included in the GBD. Two outcomes evaluated for SVAC (ischemic heart disease and anorexia nervosa) were rated with zero stars and therefore not eligible for inclusion. For outcomes included in previous GBD rounds, relative risks were updated with new results from the above BPRF analyses (Appendix 2, tables S3-S4).

Section 4: Population attributable fraction calculations

Section 4.1: Cumulative risk approach for HIV/AIDS

GBD typically quantifies burden based on deaths and prevalence; the relationship between IPV and SVAC with HIV is better understood using incident risk. To calculate a PAF, we first drew upon estimates of the proportion of HIV transmission due to sexual contact and applied this to the overall HIV incidence, yielding the cumulative incidence of HIV attributable to sexual transmission.

Next, we utilized the standard PAF equation to calculate a PAF for HIV associated with each risk factor ($PAF_{HIV\ incidence}$).

The cumulative incidence of HIV attributable to IPV and SVAC was then determined by applying the respective IPV-HIV and SVAC-HIV PAFs to the calculated cumulative incidence of HIV resulting from sexual transmission.

The ratio of cumulative IPV-attributable HIV incidence to total HIV incidence was used as an approximation of the relevant PAF for HIV prevalence:

$$\frac{Cumulative\ HIV\ incidence\ due\ to\ IPV}{Cumulative\ HIV\ incidence\ overall} = \frac{1 - \prod_{a=o}^{a=n}\left(1 - PAF_{ay} * I_{ay}\right)}{1 - \prod_{a=o}^{a=n}\left(1 - I_{ay}\right)}$$

where:

I = annual incidence rate of HIV

a = age (15-95)

y = year (1990-2023)

$$PAF_{HIV\ incidence} = \frac{[Prevalence\ of\ IPV]_{ay} * (IRR-1)}{[Prevalence\ of\ IPV]_{ay} * (IRR-1)+1}$$

Section 5: Demographics and age-standardisation

For the 204 countries and territories included in analysis, we produced estimates by sex (IPV: females only; SVAC: males and females) and five-year age group from 1990 to 2023.

Additionally, we produced estimates aggregated by age:

- All-age prevalence reflects population-weighted estimates for ages 15 and above.
- Age-standardised prevalence reflects weighting according to the GBD population standard for ages 15 and above.
- Attributable burden is calculated for ages 15 and above in line with the exposure case definition for IPV and because there is insufficient data to estimate exposure to SVAC among children currently under 15 years old.

Age-standardised results for individuals aged 15+ were calculated at the 1000 draw level by multiplying each draw in an age group by a set age weight (Table S6). The age weight for each age group was normalised to the population aged 15+ so that the weights summed to 1 using the world population age standard as defined by the GBD. Additional details on the GBD world population standard can be found in the GBD 2023 Demographics Capstone.

Table S6: GBD standard age weights

Age group name	Age group weight
15 to 19	0.116247
20 to 24	0.109404
25 to 29	0.106462
30 to 34	0.10268
35 to 39	0.095757
40 to 44	0.08621
45 to 49	0.077291
50 to 54	0.068902
55 to 59	0.060946
60 to 64	0.05164
65 to 69	0.041863
70 to 74	0.031768
75 to 79	0.022404
80 to 84	0.015388
85 to 89	0.008478
90 to 94	0.003459
95 plus	0.001101

Section 6: Locations estimated

For the Global Burden of Diseases, Injuries, and Risk Factors Study 2023 (GBD 2023), we produced estimates for 204 countries and territories (Table S7). These countries and territories were nested in 21 aggregate regions and seven aggregate super-regions. Standard regions and super-regions in the GBD are defined based on a combination of epidemiologic patterns and spatial distance.

Table S7: GBD location hierarchy

Location	Super-Region
Albania	Central Europe, Eastern Europe, and Central Asia
Armenia	Central Europe, Eastern Europe, and Central Asia
Azerbaijan	Central Europe, Eastern Europe, and Central Asia
Belarus	Central Europe, Eastern Europe, and Central Asia
Bosnia and Herzegovina	Central Europe, Eastern Europe, and Central Asia
Bulgaria	Central Europe, Eastern Europe, and Central Asia
Croatia	Central Europe, Eastern Europe, and Central Asia
Czechia	Central Europe, Eastern Europe, and Central Asia
Estonia	Central Europe, Eastern Europe, and Central Asia
Georgia	Central Europe, Eastern Europe, and Central Asia
Hungary	Central Europe, Eastern Europe, and Central Asia
Kazakhstan	Central Europe, Eastern Europe, and Central Asia
Kyrgyzstan	Central Europe, Eastern Europe, and Central Asia
Latvia	Central Europe, Eastern Europe, and Central Asia
Lithuania	Central Europe, Eastern Europe, and Central Asia
Mongolia	Central Europe, Eastern Europe, and Central Asia
Montenegro	Central Europe, Eastern Europe, and Central Asia
North Macedonia	Central Europe, Eastern Europe, and Central Asia
Poland	Central Europe, Eastern Europe, and Central Asia
Republic of Moldova	Central Europe, Eastern Europe, and Central Asia
Romania	Central Europe, Eastern Europe, and Central Asia
Russian Federation	Central Europe, Eastern Europe, and Central Asia
Serbia	Central Europe, Eastern Europe, and Central Asia
Slovakia	Central Europe, Eastern Europe, and Central Asia
Slovenia	Central Europe, Eastern Europe, and Central Asia
Tajikistan	Central Europe, Eastern Europe, and Central Asia
Turkmenistan	Central Europe, Eastern Europe, and Central Asia
Ukraine	Central Europe, Eastern Europe, and Central Asia

Uzbekistan	Central Europe, Eastern Europe, and Central Asia
Andorra	High-income
Argentina	High-income
Australia	High-income
Austria	High-income
Belgium	High-income
Brunei Darussalam	High-income
Canada	High-income
Chile	High-income
Cyprus	High-income
Denmark	High-income
Finland	High-income
France	High-income
Germany	High-income
Greece	High-income
Greenland	High-income
Iceland	High-income
Ireland	High-income
Israel	High-income
Italy	High-income
Japan	High-income
Luxembourg	High-income
Malta	High-income
Monaco	High-income
Netherlands	High-income
New Zealand	High-income
Norway	High-income
Portugal	High-income
Republic of Korea	High-income
San Marino	High-income
Singapore	High-income
Spain	High-income
Sweden	High-income
Switzerland	High-income
United Kingdom	High-income
United States of America	High-income
Uruguay	High-income
Antigua and Barbuda	Latin America and Caribbean

Bahamas	Latin America and Caribbean
Barbados	Latin America and Caribbean
Belize	Latin America and Caribbean
Bermuda	Latin America and Caribbean
Bolivia (Plurinational State of)	Latin America and Caribbean
Brazil	Latin America and Caribbean
Colombia	Latin America and Caribbean
Costa Rica	Latin America and Caribbean
Cuba	Latin America and Caribbean
Dominica	Latin America and Caribbean
Dominican Republic	Latin America and Caribbean
Ecuador	Latin America and Caribbean
El Salvador	Latin America and Caribbean
Grenada	Latin America and Caribbean
Guatemala	Latin America and Caribbean
Guyana	Latin America and Caribbean
Haiti	Latin America and Caribbean
Honduras	Latin America and Caribbean
Jamaica	Latin America and Caribbean
Mexico	Latin America and Caribbean
Nicaragua	Latin America and Caribbean
Panama	Latin America and Caribbean
Paraguay	Latin America and Caribbean
Peru	Latin America and Caribbean
Puerto Rico	Latin America and Caribbean
Saint Kitts and Nevis	Latin America and Caribbean
Saint Lucia	Latin America and Caribbean
Saint Vincent and the Grenadines	Latin America and Caribbean
Suriname	Latin America and Caribbean
Trinidad and Tobago	Latin America and Caribbean
United States Virgin Islands	Latin America and Caribbean
Venezuela (Bolivarian Republic of)	Latin America and Caribbean
Afghanistan	North Africa and Middle East
Algeria	North Africa and Middle East
Bahrain	North Africa and Middle East
Egypt	North Africa and Middle East
Iran (Islamic Republic of)	North Africa and Middle East
Iraq	North Africa and Middle East

Jordan	North Africa and Middle East
Kuwait	North Africa and Middle East
Lebanon	North Africa and Middle East
Libya	North Africa and Middle East
Morocco	North Africa and Middle East
Oman	North Africa and Middle East
Palestine	North Africa and Middle East
Qatar	North Africa and Middle East
Saudi Arabia	North Africa and Middle East
Sudan	North Africa and Middle East
Syrian Arab Republic	North Africa and Middle East
Turkiye	North Africa and Middle East
Tunisia	North Africa and Middle East
United Arab Emirates	North Africa and Middle East
Yemen	North Africa and Middle East
Bangladesh	South Asia
Bhutan	South Asia
India	South Asia
Nepal	South Asia
Pakistan	South Asia
American Samoa	Southeast Asia, East Asia, and Oceania
Cambodia	Southeast Asia, East Asia, and Oceania
China	Southeast Asia, East Asia, and Oceania
Cook Islands	Southeast Asia, East Asia, and Oceania
Democratic People's Republic of Korea	Southeast Asia, East Asia, and Oceania
Fiji	Southeast Asia, East Asia, and Oceania
Guam	Southeast Asia, East Asia, and Oceania
Indonesia	Southeast Asia, East Asia, and Oceania
Kiribati	Southeast Asia, East Asia, and Oceania
Lao People's Democratic Republic	Southeast Asia, East Asia, and Oceania
Malaysia	Southeast Asia, East Asia, and Oceania
Maldives	Southeast Asia, East Asia, and Oceania
Marshall Islands	Southeast Asia, East Asia, and Oceania
Mauritius	Southeast Asia, East Asia, and Oceania
Micronesia (Federated States of)	Southeast Asia, East Asia, and Oceania
Myanmar	Southeast Asia, East Asia, and Oceania
Nauru	Southeast Asia, East Asia, and Oceania
Niue	Southeast Asia, East Asia, and Oceania

Northern Mariana Islands	Southeast Asia, East Asia, and Oceania
Palau	Southeast Asia, East Asia, and Oceania
Papua New Guinea	Southeast Asia, East Asia, and Oceania
Philippines	Southeast Asia, East Asia, and Oceania
Samoa	Southeast Asia, East Asia, and Oceania
Seychelles	Southeast Asia, East Asia, and Oceania
Solomon Islands	Southeast Asia, East Asia, and Oceania
Sri Lanka	Southeast Asia, East Asia, and Oceania
Taiwan	Southeast Asia, East Asia, and Oceania
Thailand	Southeast Asia, East Asia, and Oceania
Timor-Leste	Southeast Asia, East Asia, and Oceania
Tokelau	Southeast Asia, East Asia, and Oceania
Tonga	Southeast Asia, East Asia, and Oceania
Tuvalu	Southeast Asia, East Asia, and Oceania
Vanuatu	Southeast Asia, East Asia, and Oceania
Viet Nam	Southeast Asia, East Asia, and Oceania
Angola	Sub-Saharan Africa
Benin	Sub-Saharan Africa
Botswana	Sub-Saharan Africa
Burkina Faso	Sub-Saharan Africa
Burundi	Sub-Saharan Africa
Côte d'Ivoire	Sub-Saharan Africa
Cabo Verde	Sub-Saharan Africa
Cameroon	Sub-Saharan Africa
Central African Republic	Sub-Saharan Africa
Chad	Sub-Saharan Africa
Comoros	Sub-Saharan Africa
Congo	Sub-Saharan Africa
Democratic Republic of the Congo	Sub-Saharan Africa
Djibouti	Sub-Saharan Africa
Equatorial Guinea	Sub-Saharan Africa
Eritrea	Sub-Saharan Africa
Eswatini	Sub-Saharan Africa
Ethiopia	Sub-Saharan Africa
Gabon	Sub-Saharan Africa
Gambia	Sub-Saharan Africa
Ghana	Sub-Saharan Africa
Guinea	Sub-Saharan Africa

Guinea-Bissau	Sub-Saharan Africa
Kenya	Sub-Saharan Africa
Lesotho	Sub-Saharan Africa
Liberia	Sub-Saharan Africa
Madagascar	Sub-Saharan Africa
Malawi	Sub-Saharan Africa
Mali	Sub-Saharan Africa
Mauritania	Sub-Saharan Africa
Mozambique	Sub-Saharan Africa
Namibia	Sub-Saharan Africa
Niger	Sub-Saharan Africa
Nigeria	Sub-Saharan Africa
Rwanda	Sub-Saharan Africa
Sao Tome and Principe	Sub-Saharan Africa
Senegal	Sub-Saharan Africa
Sierra Leone	Sub-Saharan Africa
Somalia	Sub-Saharan Africa
South Africa	Sub-Saharan Africa
South Sudan	Sub-Saharan Africa
Togo	Sub-Saharan Africa
Uganda	Sub-Saharan Africa
United Republic of Tanzania	Sub-Saharan Africa
Zambia	Sub-Saharan Africa
Zimbabwe	Sub-Saharan Africa

Section 7: GBD estimation draws

In GBD 2023, we reduced the number of draws (or computations) per process to 250 to minimize computing power and time. Based on simulation testing, we determined that a change in the number of draws did not impact final mean estimates, nor lead to inappropriately narrow uncertainty estimates.

To help assess the impact of reduced draws, we conducted the following experiment. We first generated 1000 samples from a random variable X~N(30,10^2) to be used as our "true" distribution. For each n depicted in the plot below, we subset n samples with which we calculated the sample standard deviation. We then estimated a 95% uncertainty interval 30±1.96s and computed the percent of the "true" distribution covered by that 95% uncertainty interval. For each n, these steps were conducted 1000 times. The distribution of coverages from those 1000 simulations for each number of draws is shown below in green:

Figure S6: Simulations of draw quantities

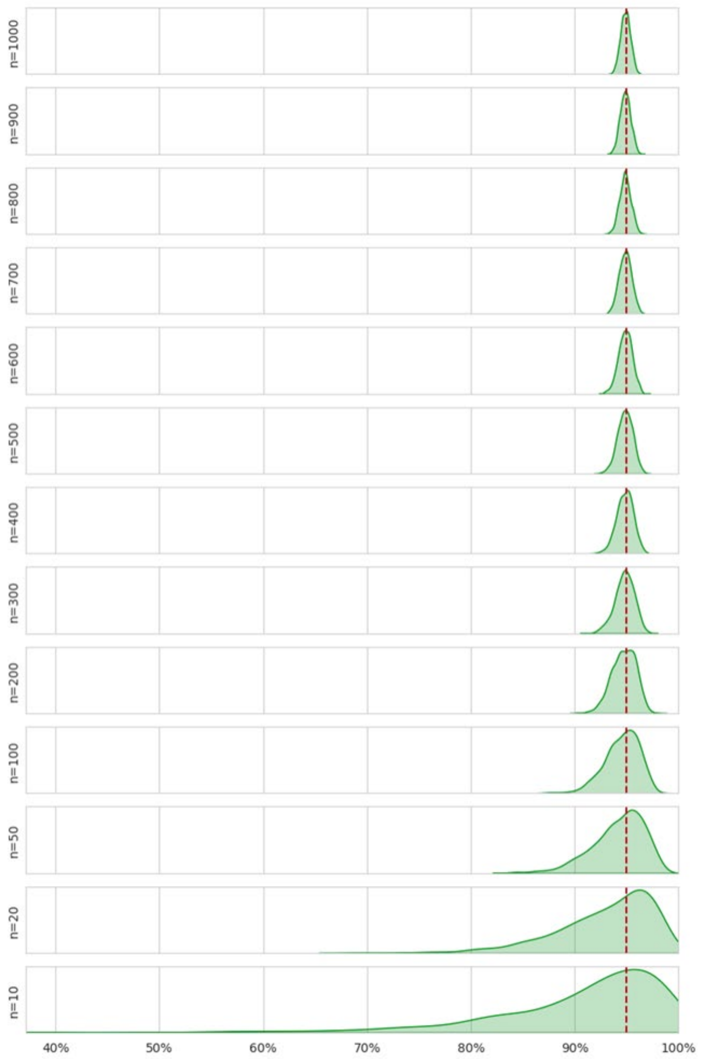

Section 8: Risk factors hierarchy

For the GBD 2023, we produced estimates for 53 risk factors (Table S8). These risk factors were nested in 20 aggregate groupings and three further categorizations: Behavioural risk factors, Environmental/occupational risk factors, and Metabolic risk factors.

Table S8: GBD 2023 risk factor hierarchy

Level 1 Category	Level 2 Risk Factor	Level 3 Risk Factor
Behavioral risks	Child and maternal malnutrition	Suboptimal breastfeeding
Behavioral risks	Child and maternal malnutrition	Child growth failure
Behavioral risks	Child and maternal malnutrition	Low birth weight and short gestation
Behavioral risks	Child and maternal malnutrition	Iron deficiency
Behavioral risks	Child and maternal malnutrition	Vitamin A deficiency
Behavioral risks	Child and maternal malnutrition	Zinc deficiency
Behavioral risks	Tobacco	Smoking
Behavioral risks	Tobacco	Chewing tobacco
Behavioral risks	Tobacco	Second-hand smoke
Behavioral risks	High alcohol use	
Behavioral risks	Drug use	
Behavioral risks	Dietary risks	Diet low in fruits
Behavioral risks	Dietary risks	Diet low in vegetables
Behavioral risks	Dietary risks	Diet low in legumes
Behavioral risks	Dietary risks	Diet low in whole grains
Behavioral risks	Dietary risks	Diet low in nuts and seeds
Behavioral risks	Dietary risks	Diet low in milk
Behavioral risks	Dietary risks	Diet high in red meat
Behavioral risks	Dietary risks	Diet high in processed meat
Behavioral risks	Dietary risks	Diet high in sugar-sweetened beverages
Behavioral risks	Dietary risks	Diet low in fiber
Behavioral risks	Dietary risks	Diet low in calcium
Behavioral risks	Dietary risks	Diet low in seafood omega-3 fatty acids
Behavioral risks	Dietary risks	Diet low in omega-6 polyunsaturated fatty acids
Behavioral risks	Dietary risks	Diet high in trans fatty acids
Behavioral risks	Dietary risks	Diet high in sodium
Behavioral risks	Low physical activity	
Behavioral risks	Intimate partner violence	
Behavioral risks	Unsafe sex	
Behavioral risks	Sexual violence against children and bullying	Sexual violence against children
Behavioral risks	Sexual violence against children and bullying	Bullying victimization
Environmental/occupational risks	Unsafe water, sanitation, and handwashing	Unsafe water source

Environmental/occupational risks	Unsafe water, sanitation, and handwashing	Unsafe sanitation
Environmental/occupational risks	Unsafe water, sanitation, and handwashing	No access to handwashing facility
Environmental/occupational risks	Air pollution	Particulate matter pollution
Environmental/occupational risks	Air pollution	Ambient ozone pollution
Environmental/occupational risks	Air pollution	Ambient nitrogen dioxide pollution
Environmental/occupational risks	Other environmental risks	Residential radon
Environmental/occupational risks	Other environmental risks	Lead exposure
Environmental/occupational risks	Occupational risks	Occupational carcinogens
Environmental/occupational risks	Occupational risks	Occupational asthmagens
Environmental/occupational risks	Occupational risks	Occupational particulate matter, gases, and fumes
Environmental/occupational risks	Occupational risks	Occupational noise
Environmental/occupational risks	Occupational risks	Occupational injuries
Environmental/occupational risks	Occupational risks	Occupational ergonomic factors
Environmental/occupational risks	Non-optimal temperature	High temperature
Environmental/occupational risks	Non-optimal temperature	Low temperature
Metabolic risks	High fasting plasma glucose	
Metabolic risks	High systolic blood pressure	
Metabolic risks	High body-mass index	
Metabolic risks	Low bone mineral density	
Metabolic risks	Kidney dysfunction	
Metabolic risks	High LDL cholesterol	

References

1 Institute for Health Metrics and Evaluation (IHME). Global Health Data Exchange (GHDx). University of Washington, 2019 http://ghdx.healthdata.org/.

2 World Health Organization. Global Database on the Prevalence of Violence Against Women. https://vaw-data.srhr.org/.

3 UN Women. Global Database on Violence Against Women. https://evaw-global-database.unwomen.org/en.

4 GBD 2021 Risk Factors Collaborators. Global burden and strength of evidence for 88 risk factors in 204 countries and 811 subnational locations, 1990-2021: a systematic analysis for the Global Burden of Disease Study 2021. *The Lancet* 2024; **403**: 2162–203.

5 Ng M, Freeman MK, Fleming TD, *et al.* Smoking prevalence and cigarette consumption in 187 countries, 1980-2012. *JAMA* 2014; **311**: 183–92.

6 Hyndman RJ, Athanasopoulos G. Forecasting: principles and practice, 2nd edition. Melbourne: OTexts, 2018 https://otexts.com/fpp2/ (accessed March 1, 2024).

7 Cagney J, Spencer CN, Flor LS, *et al.* Prevalence of sexual violence against children and age at first exposure: a global analysis by location, age and sex (1990-2023). *Lancet Press* 2025.

8 CDC. Violence Against Children and Youth Surveys (VACS). 2021. https://www.cdc.gov/violence-against-children/site.html?CDC_ AAref_Val=https://www.cdc.gov/violenceprevention/ childabuseandneglect/vacs/index.html (accessed March 20, 2024).

9 García-Moreno C, Jansen H, Ellsberg M, Heise L, Watts C. WHO multi-country study on women's health and domestic violence against women: summary report of initial results on prevalence, health outcomes and women's responses. Geneva: World Health Organization, 2005.

10 Spencer CN, Khalil M, Herbert M, *et al.* Health effects associated with exposure to intimate partner violence against women and childhood sexual abuse: a Burden of Proof study. *Nat Med* 2023; **29**: 3243–58.

11 Zheng P, Afshin A, Biryukov S, *et al.* The Burden of Proof studies: assessing the evidence of risk. *Nat Med* 2022; **28**: 2038–44.

12 Dai X, Gil GF, Reitsma MB, *et al.* Health effects associated with smoking: a Burden of Proof study. *Nat Med* 2022; **28**: 2045–55.

13 Lescinsky H, Afshin A, Ashbaugh C, *et al.* Health effects associated with consumption of unprocessed red meat: a Burden of Proof study. *Nat Med* 2022; **28**: 2075–82.

14 Flor LS, Stein C, Gil GF, *et al.* Health effects associated with exposure of children to physical violence, psychological violence and neglect: a Burden of Proof study. *Nat Hum Behav* 2025; 1–20.

15 Stein C, Flor LS, Gil GF, *et al.* The health effects associated with physical, sexual and psychological gender-based violence against men and women: a Burden of Proof study. *Nat Hum Behav* 2025; 1–16.

16 Guyatt GH, Oxman AD, Vist G, *et al.* GRADE guidelines: 4. Rating the quality of evidence—study limitations (risk of bias). *J Clin Epidemiol* 2011; **64**: 407–15.
