# Supplementary material for: Disease burden attributable to intimate partner violence against females and sexual violence against children in 204 countries and territories, 1990–2023: a systematic analysis for the Global Burden of Disease Study 2023
Source: Lancet. 2026 Jan 3;407(10523):31–52. doi: 10.1016/S0140-6736(25)02503-6 (PMC12775558; doi:10.1016/S0140-6736(25)02503-6)

THE LANCET

Supplementary appendix 2

This appendix formed part of the original submission and has been peer reviewed. We post it as supplied by the authors.

Supplement to: GBD 2023 Intimate Partner Violence and Sexual Violence against Children Collaborators. Disease burden attributable to intimate partner violence against females and sexual violence against children in 204 countries and territories, 1990–2023: a systematic analysis for the Global Burden of Disease Study 2023. *Lancet* 2025; published online Dec 9. https://doi.org/10.1016/S0140-6736(25)02503-6.

Supplementary Results

Disease burden attributable to intimate partner violence against females and sexual violence against children in 204 countries and territories, 1990–2023: a systematic analysis from the Global Burden of Disease Study 2023

GBD 2023 Intimate Partner Violence and Sexual Violence against Children collaborators

Contents

Section 1: Burden attributable to IPV and SVAC in 1990 and 2023 3

Table S1: Age-standardised numbers and rates of deaths attributable to IPV and SVAC by location and sex.............. 3

Table S2: Age-standardised numbers and rates of DALYs attributable to IPV and SVAC by location and sex. 23

Section 2: Prevalence of IPV and SVAC 42

Table S3: Global prevalence of IPV and SVAC by sex and age group 42

Figure S1: Age-standardised prevalence of SVAC among males and females 15 years and older in 2023........ 43

Table S4: Age-standardised prevalence of IPV and SVAC among individuals aged 15 years and older, by sex, in 2023 44

Section 3: IPV and SVAC relative risks 52

Table S5: Strength of the evidence for the relationship between IPV and the 7 health outcomes analysed with the Burden of Proof methodology........ 52

Table S6: Strength of the evidence for the relationship between SVAC and the 16 health outcomes analysed with the Burden of Proof methodology........ 54

Section 4: Additional detailed burden results for 2023 57

Table S7: Cause-specific 15+ age-standardized DALY rates attributable to IPV by world region 57

Figure S2: Age-standardized rates of all-cause DALYs attributable to SVAC by country for females and males aged 15 years and older 58

Table S8: Cause-specific 15+ age-standardized DALY rates attributable to SVAC by region and sex 59

Figure S3: Leading 25 Risk Factors, ranked by attributable DALY counts, among females 15+ years old. 62

Figure S4: Leading 25 Risk Factors, ranked by attributable DALY counts, among females 15-49 years old. 63

Figure S5: Leading 25 Risk Factors, ranked by attributable DALY counts, among males 15+ years old. 64

Figure S6: Leading 25 Risk Factors, ranked by attributable DALY counts, among males 15-49 years old. 65

Figure S7: Percentages of all-cause global DALYs due to IPV and SVAC among females and males across 5-year GBD age groups........ 66

Table S9: Counts of female 15+ cause-specific DALYs attributable to IPV by region........ 68

Table S10: Percentages of female 15+ cause-specific DALYs attributable to IPV by region 69

Table S11: Counts of female 15+ cause-specific deaths attributable to IPV by region 70

Table S12: Percentages of female 15+ cause-specific deaths attributable to IPV by region........ 71

Table S13: Cause-specific 15+ age-standardized death rates attributable to IPV by world region........ 72

Table S14: Counts of cause-specific DALYs attributable to SVAC by region and sex........ 73

Table S15: Percentages of cause-specific DALYs attributable to SVAC by region and sex 76

Table S16: Counts of cause-specific deaths attributable to SVAC by region and sex 78

Table S17: Percentages of cause-specific deaths attributable to SVAC by region and sex 80

Table S18: Cause-specific 15+ age-standardized death rates attributable to SVAC by region and sex........ 82

Figure S8: Percentages of cause-specific DALYs attributable to SVAC by world region and sex 84

Figure S9: Global and regional rates of cause-specific deaths attributable to IPV and SVAC in 2023 85

Section 1: Burden attributable to IPV and SVAC in 1990 and 2023

Table S1: Age-standardised numbers and rates of deaths attributable to IPV and SVAC by location and sex.

Risk Factor	Sex	Location	Number of Deaths		Rate of Deaths (per 100,000)	
			1990	2023	1990	2023
IPV	Female	Global	138,000 (-14,000—301,000)	145,000 (9,180—301,000)	7.6 (-0.9—16.7)	4.8 (0.3—9.8)
IPV	Female	Central Europe, Eastern Europe, and Central Asia	6,790 (611—16,500)	4,520 (334—11,800)	3.9 (0.4—9.4)	2.4 (0.2—6.0)
IPV	Female	Central Asia	624 (153—1,490)	570 (-10.7—1,650)	2.8 (0.7—6.7)	1.6 (-0.0—4.7)
IPV	Female	Armenia	14.6 (5.95—34.1)	7.50 (1.65—21.8)	1.2 (0.5—2.8)	0.5 (0.1—1.4)
IPV	Female	Azerbaijan	42.4 (17.6—76.7)	26.9 (6.72—61.6)	1.6 (0.7—3.0)	0.6 (0.2—1.5)
IPV	Female	Georgia	20.1 (5.24—56.9)	7.83 (1.36—25.1)	0.9 (0.2—2.5)	0.5 (0.1—1.4)
IPV	Female	Kazakhstan	242 (58.2—611)	166 (-1.70—404)	4.1 (1.0—10.3)	2.2 (-0.0—5.4)
IPV	Female	Kyrgyzstan	60.2 (7.76—134)	34.7 (0.557—79.9)	4.3 (0.5—9.7)	1.4 (0.0—3.4)
IPV	Female	Mongolia	35.3 (3.71—78.7)	22.8 (-5.01—54.5)	5.8 (0.7—12.7)	1.9 (-0.4—4.7)
IPV	Female	Tajikistan	43.2 (5.60—99.5)	55.0 (-11.4—135)	2.9 (0.4—6.4)	1.6 (-0.3—4.2)
IPV	Female	Turkmenistan	29.7 (6.16—75.5)	41.0 (3.79—113)	2.7 (0.6—6.8)	2.1 (0.2—6.0)
IPV	Female	Uzbekistan	136 (24.4—343)	209 (-10.7—746)	2.3 (0.4—5.9)	1.7 (-0.1—6.2)
IPV	Female	Central Europe	1,670 (-123—4,390)	729 (-65.4—2,080)	3.2 (-0.2—8.3)	1.3 (-0.1—3.5)
IPV	Female	Albania	25.0 (6.24—50.6)	13.8 (0.350—34.7)	2.3 (0.6—4.7)	1.3 (0.0—3.1)
IPV	Female	Bosnia and Herzegovina	44.5 (-2.76—124)	16.3 (-0.495—49.9)	2.6 (-0.2—7.2)	0.9 (0.0—2.7)
IPV	Female	Bulgaria	109 (-42.4—340)	44.2 (-14.8—133)	2.8 (-1.1—8.5)	1.3 (-0.5—3.7)
IPV	Female	Croatia	84.9 (4.82—246)	31.2 (1.66—98.9)	3.8 (0.3—10.8)	1.5 (0.1—4.2)
IPV	Female	Czechia	214 (-28.0—501)	82.0 (3.82—221)	4.8 (-0.6—10.9)	1.6 (0.0—4.1)
IPV	Female	Hungary	307 (-72.2—885)	110 (-24.2—331)	6.5 (-1.5—17.9)	2.2 (-0.4—6.0)
IPV	Female	Montenegro	8.09 (-0.757—21.3)	6.57 (-1.04—18.3)	3.5 (-0.3—9.1)	2.1 (-0.2—5.7)
IPV	Female	North Macedonia	16.4 (-1.97—45.8)	10.3 (-1.08—31.5)	2.2 (-0.3—6.3)	1.2 (-0.1—3.4)
IPV	Female	Poland	327 (-55.4—906)	173 (-28.6—528)	2.1 (-0.3—5.7)	1.0 (-0.2—3.0)
IPV	Female	Romania	265 (48.3—578)	98.3 (5.40—237)	2.9 (0.5—6.1)	1.1 (0.1—2.4)
IPV	Female	Serbia	145 (-8.15—467)	84.4 (-10.6—265)	3.8 (-0.2—11.8)	1.7 (-0.2—5.2)
IPV	Female	Slovakia	59.9 (-1.29—139)	34.3 (-0.989—85.7)	2.9 (-0.1—6.6)	1.3 (-0.0—3.1)
IPV	Female	Slovenia	33.4 (-3.61—107)	14.5 (-1.07—51.3)	3.9 (-0.4—12.3)	1.3 (-0.1—4.0)
IPV	Female	Eastern Europe	4,500 (538—11,000)	3,220 (411—8,170)	4.6 (0.7—10.8)	3.2 (0.5—7.6)
IPV	Female	Belarus	112 (15.5—317)	88.5 (3.39—270)	2.5 (0.4—6.9)	1.9 (0.1—5.6)
IPV	Female	Estonia	27.4 (-3.29—78.1)	10.2 (-0.518—31.6)	3.9 (-0.5—10.6)	1.5 (-0.1—4.2)
IPV	Female	Latvia	61.3 (-12.6—153)	24.4 (-2.94—60.0)	4.9 (-1.0—11.6)	2.5 (-0.4—5.6)
IPV	Female	Lithuania	79.6 (-25.7—200)	41.2 (-7.52—108)	5.0 (-1.6—12.3)	2.8 (-0.5—6.6)
IPV	Female	Republic of Moldova	74.4 (-4.86—177)	35.4 (-6.17—79.1)	4.2 (-0.3—10.0)	1.9 (-0.2—4.2)
IPV	Female	Russian Federation	3,460 (595—8,660)	2,450 (399—6,200)	5.3 (1.1—12.8)	3.4 (0.6—8.2)
IPV	Female	Ukraine	682 (2.92—1,610)	571 (13.6—1,420)	2.9 (0.1—6.5)	2.8 (0.1—6.7)
IPV	Female	High-income	13,500 (-1,580—31,900)	19,400 (1,290—39,300)	3.6 (-0.4—8.3)	4.3 (0.3—8.5)
IPV	Female	Australasia	245 (1.14—551)	378 (-40.5—907)	3.1 (0.0—6.9)	3.0 (-0.3—7.0)
IPV	Female	Australia	207 (4.37—458)	307 (-31.2—739)	3.2 (0.1—6.9)	2.8 (-0.3—6.7)
IPV	Female	New Zealand	37.6 (-4.45—91.8)	71.2 (-9.45—162)	2.8 (-0.3—6.9)	3.6 (-0.5—8.1)
IPV	Female	High-income Asia Pacific	2,540 (-962—7,650)	2,880 (-1,110—8,640)	3.5 (-1.3—10.4)	3.6 (-1.6—10.4)
IPV	Female	Brunei Darussalam	0.885 (-0.0325—2.09)	1.53 (-0.136—4.16)	1.2 (-0.0—3.0)	0.9 (-0.1—2.3)
IPV	Female	Japan	2,040 (-873—6,410)	1,960 (-806—5,890)	3.6 (-1.6—11.0)	3.6 (-1.7—10.5)
IPV	Female	Republic of Korea	481 (-105—1,390)	896 (-307—2,700)	2.9 (-0.6—8.4)	3.9 (-1.4—11.3)

Risk Factor	Sex	Location	Number of Deaths		Rate of Deaths (per 100,000)	
			1990	2023	1990	2023
IPV	Female	Singapore	25.3 (-2.15—98.3)	20.5 (-2.48—74.8)	2.1 (-0.1—8.1)	0.8 (-0.1—3.0)
IPV	Female	High-income North America	5,180 (460—9,600)	11,500 (1,730—21,700)	4.6 (0.4—8.4)	7.9 (1.1—14.7)
IPV	Female	Canada	331 (-12.3—758)	346 (53.9—869)	3.0 (-0.1—6.8)	2.4 (0.4—5.8)
IPV	Female	Greenland	6.07 (-0.274—13.9)	3.50 (-0.379—9.49)	31.0 (-0.6—72.1)	17.2 (-1.4—47.8)
IPV	Female	United States of America	4,850 (470—8,910)	11,200 (1,630—20,800)	4.7 (0.5—8.6)	8.5 (1.1—15.7)
IPV	Female	Southern Latin America	594 (-22.6—1,300)	772 (-43.2—1,660)	3.3 (-0.1—7.2)	2.7 (-0.1—5.8)
IPV	Female	Argentina	359 (3.34—775)	552 (-31.8—1,190)	3.0 (0.0—6.5)	2.9 (-0.2—6.2)
IPV	Female	Chile	199 (-19.9—427)	169 (-4.22—392)	4.2 (-0.5—9.1)	2.1 (-0.0—4.9)
IPV	Female	Uruguay	35.3 (3.71—88.0)	51.1 (-4.12—132)	2.9 (0.3—7.2)	3.6 (-0.3—8.9)
IPV	Female	Western Europe	4,910 (-940—12,900)	3,790 (-444—10,200)	2.9 (-0.5—7.5)	1.9 (-0.2—4.9)
IPV	Female	Andorra	0.415 (-0.223—1.43)	0.472 (-0.145—1.54)	1.9 (-1.0—6.7)	1.2 (-0.4—3.8)
IPV	Female	Austria	123 (-10.6—385)	74.4 (-0.483—219)	3.6 (-0.3—10.6)	1.8 (0.1—4.9)
IPV	Female	Belgium	171 (-21.0—490)	153 (-18.8—444)	4.0 (-0.5—11.1)	2.9 (-0.4—8.0)
IPV	Female	Cyprus	4.63 (1.47—10.4)	5.28 (1.33—12.1)	1.6 (0.5—3.6)	0.9 (0.2—2.0)
IPV	Female	Denmark	148 (-29.8—390)	66.8 (-9.21—162)	6.5 (-1.2—16.5)	2.5 (-0.4—5.6)
IPV	Female	Finland	128 (-20.0—307)	90.1 (-11.8—224)	5.9 (-0.8—13.9)	3.9 (-0.6—9.0)
IPV	Female	France	940 (-135—2,690)	689 (-95.1—2,040)	3.8 (-0.6—10.6)	2.1 (-0.3—5.8)
IPV	Female	Germany	1,410 (-425—3,700)	802 (-221—2,370)	3.7 (-1.1—9.3)	2.1 (-0.6—5.7)
IPV	Female	Greece	34.5 (10.7—81.8)	34.3 (5.36—81.0)	0.8 (0.3—1.8)	0.7 (0.1—1.6)
IPV	Female	Iceland	3.22 (-0.414—8.93)	4.38 (-0.261—10.2)	3.4 (-0.5—9.5)	2.8 (-0.1—6.1)
IPV	Female	Ireland	23.6 (-2.47—69.3)	28.2 (-0.263—76.4)	1.8 (-0.2—5.3)	1.2 (-0.0—3.3)
IPV	Female	Israel	31.6 (1.59—81.0)	36.0 (1.61—90.9)	1.8 (0.1—4.6)	1.0 (0.0—2.4)
IPV	Female	Italy	400 (10.8—1,070)	245 (-8.24—642)	1.6 (0.1—4.1)	0.9 (-0.0—2.3)
IPV	Female	Luxembourg	6.76 (-0.634—18.5)	4.61 (-0.376—12.3)	4.0 (-0.3—10.9)	1.5 (-0.1—3.9)
IPV	Female	Malta	1.92 (0.511—4.58)	1.57 (0.383—3.80)	1.3 (0.3—3.0)	0.7 (0.2—1.5)
IPV	Female	Monaco	0.475 (-0.260—1.74)	0.400 (-0.128—1.49)	3.3 (-1.9—11.4)	2.3 (-0.8—8.0)
IPV	Female	Netherlands	192 (-44.0—492)	182 (-51.3—521)	3.1 (-0.7—7.8)	2.3 (-0.7—6.3)
IPV	Female	Norway	51.2 (-8.65—145)	55.8 (-4.89—131)	3.1 (-0.5—8.5)	2.5 (-0.2—5.8)
IPV	Female	Portugal	138 (6.76—376)	96.3 (-1.33—253)	3.3 (0.2—8.6)	1.8 (0.1—4.1)
IPV	Female	San Marino	0.130 (-0.0363—0.395)	0.146 (-0.0346—0.473)	1.4 (-0.4—4.2)	0.9 (-0.2—2.7)
IPV	Female	Spain	226 (19.7—673)	301 (-40.8—820)	1.4 (0.1—4.0)	1.3 (-0.1—3.4)
IPV	Female	Sweden	170 (-36.5—477)	140 (-36.7—357)	4.7 (-1.0—12.8)	3.2 (-0.9—7.7)
IPV	Female	Switzerland	116 (2.97—332)	64.7 (2.59—186)	3.9 (0.2—11.0)	1.6 (0.1—4.3)
IPV	Female	United Kingdom	590 (-154—1,560)	713 (-49.3—1,510)	2.5 (-0.7—6.4)	2.5 (-0.2—5.2)
IPV	Female	Latin America and Caribbean	4,390 (1,620—7,920)	7,430 (3,020—13,100)	3.3 (1.1—6.0)	3.2 (1.3—5.6)
IPV	Female	Andean Latin America	479 (145—853)	891 (350—1,470)	3.8 (1.1—6.6)	3.6 (1.4—5.9)
IPV	Female	Bolivia (Plurinational State of)	154 (37.3—269)	261 (69.0—479)	7.4 (1.8—13.0)	5.7 (1.5—10.5)
IPV	Female	Ecuador	123 (43.9—232)	315 (123—505)	3.7 (1.3—6.8)	4.6 (1.8—7.3)
IPV	Female	Peru	202 (56.9—379)	314 (139—524)	2.8 (0.8—5.2)	2.3 (1.0—3.9)
IPV	Female	Caribbean	1,170 (402—2,240)	1,500 (778—2,320)	9.2 (3.0—17.8)	8.2 (4.3—13.1)
IPV	Female	Antigua and Barbuda	0.383 (0.219—0.626)	0.638 (0.370—1.06)	1.7 (1.0—2.8)	1.7 (1.0—2.9)
IPV	Female	Bahamas	5.26 (2.91—8.82)	11.8 (6.98—18.1)	5.4 (2.9—9.2)	6.9 (4.1—10.5)
IPV	Female	Barbados	3.69 (1.96—5.80)	4.30 (2.62—6.42)	3.6 (1.9—5.7)	3.8 (2.3—5.4)
IPV	Female	Belize	1.84 (0.887—3.28)	6.63 (3.68—10.6)	3.3 (1.5—6.3)	4.2 (2.3—6.9)
IPV	Female	Bermuda	0.647 (0.147—1.36)	0.442 (0.180—0.856)	2.5 (0.6—5.3)	1.8 (0.9—3.2)
IPV	Female	Cuba	243 (55.7—648)	129 (45.9—323)	5.7 (1.2—15.3)	2.6 (1.1—5.6)
IPV	Female	Dominica	0.531 (0.254—0.866)	0.655 (0.296—1.05)	2.2 (1.0—3.6)	2.5 (1.2—4.0)
IPV	Female	Dominican Republic	111 (48.7—202)	185 (78.7—322)	4.5 (1.8—8.3)	4.4 (1.8—7.7)
IPV	Female	Grenada	0.648 (0.236—1.24)	0.626 (0.308—0.994)	2.0 (0.7—3.9)	1.5 (0.8—2.4)
IPV	Female	Guyana	28.7 (-2.44—59.0)	28.0 (1.33—57.7)	11.6 (-1.9—24.8)	9.1 (0.4—18.7)

Risk Factor	Sex	Location	Number of Deaths		Rate of Deaths (per 100,000)	
			1990	2023	1990	2023
IPV	Female	Haiti	595 (219—1,100)	880 (460—1,450)	29.5 (10.5—55.3)	17.8 (9.2—29.4)
IPV	Female	Jamaica	35.8 (23.6—48.8)	105 (72.8—141)	4.6 (3.1—6.2)	9.2 (6.3—12.2)
IPV	Female	Puerto Rico	62.4 (31.3—98.6)	37.8 (20.7—61.1)	4.5 (2.3—7.1)	3.1 (1.8—4.7)
IPV	Female	Saint Kitts and Nevis	0.388 (0.151—0.751)	0.627 (0.221—1.28)	2.7 (1.0—5.3)	3.0 (1.1—6.0)
IPV	Female	Saint Lucia	1.74 (0.807—2.97)	1.74 (0.990—2.74)	3.8 (1.7—6.6)	2.6 (1.5—3.9)
IPV	Female	Saint Vincent and the Grenadines	1.71 (0.944—2.61)	1.69 (0.999—2.66)	4.7 (2.6—7.4)	4.0 (2.4—6.3)
IPV	Female	Suriname	9.69 (-0.436—22.5)	18.0 (-0.0152—41.4)	7.5 (-0.6—17.8)	8.0 (0.1—18.0)
IPV	Female	Trinidad and Tobago	26.7 (7.51—56.2)	33.6 (12.4—59.2)	6.5 (1.6—14.0)	6.3 (2.5—10.6)
IPV	Female	United States Virgin Islands	2.83 (1.45—4.44)	1.73 (1.00—2.61)	7.2 (3.7—11.3)	6.2 (3.9—9.1)
IPV	Female	Central Latin America	988 (306—1,820)	2,250 (608—4,250)	1.8 (0.5—3.3)	2.2 (0.6—4.2)
IPV	Female	Colombia	214 (46.8—374)	347 (-19.9—762)	1.8 (0.4—3.2)	1.7 (-0.1—3.7)
IPV	Female	Costa Rica	20.8 (7.32—34.6)	50.9 (8.96—93.6)	2.0 (0.7—3.4)	2.5 (0.5—4.6)
IPV	Female	El Salvador	62.4 (4.95—150)	73.5 (14.4—144)	3.6 (0.3—8.2)	2.9 (0.6—5.6)
IPV	Female	Guatemala	81.3 (23.4—155)	119 (26.2—254)	3.5 (0.9—6.6)	1.9 (0.4—4.1)
IPV	Female	Honduras	45.9 (22.8—80.4)	117 (64.6—188)	3.3 (1.6—5.7)	3.0 (1.7—4.7)
IPV	Female	Mexico	431 (155—750)	1,230 (424—2,270)	1.6 (0.5—2.7)	2.4 (0.8—4.4)
IPV	Female	Nicaragua	31.9 (8.40—66.7)	56.5 (-1.22—128)	2.5 (0.6—5.1)	2.2 (-0.0—5.1)
IPV	Female	Panama	10.9 (4.24—22.1)	32.4 (9.92—65.4)	1.3 (0.5—2.6)	2.1 (0.6—4.2)
IPV	Female	Venezuela (Bolivarian Republic of)	89.8 (23.6—205)	226 (70.7—440)	1.4 (0.4—3.3)	2.3 (0.8—4.4)
IPV	Female	Tropical Latin America	1,760 (772—2,990)	2,800 (1,130—5,030)	3.2 (1.3—5.6)	3.2 (1.3—5.7)
IPV	Female	Brazil	1,730 (761—2,930)	2,720 (1,110—4,890)	3.3 (1.4—5.7)	3.2 (1.3—5.7)
IPV	Female	Paraguay	25.9 (10.1—52.3)	78.5 (15.7—164)	2.0 (0.8—4.2)	3.3 (0.7—7.0)
IPV	Female	North Africa and Middle East	2,370 (391—5,130)	4,170 (1,080—8,020)	2.3 (0.4—4.9)	1.9 (0.5—3.7)
IPV	Female	Afghanistan	90.6 (9.61—180)	230 (38.5—418)	3.2 (0.3—6.4)	2.5 (0.4—4.8)
IPV	Female	Algeria	198 (14.2—520)	249 (19.4—639)	2.5 (0.1—6.4)	1.5 (0.1—3.9)
IPV	Female	Bahrain	2.33 (-0.106—5.48)	4.71 (0.869—10.1)	1.8 (-0.0—4.3)	1.1 (0.2—2.3)
IPV	Female	Egypt	154 (-0.492—372)	190 (45.1—366)	0.9 (-0.0—2.2)	0.6 (0.1—1.1)
IPV	Female	Iran (Islamic Republic of)	510 (34.3—1,220)	660 (124—1,490)	2.9 (0.2—6.7)	2.1 (0.4—4.7)
IPV	Female	Iraq	157 (51.2—306)	752 (415—1,250)	3.0 (1.0—5.9)	5.0 (2.9—8.0)
IPV	Female	Jordan	19.4 (8.07—35.0)	37.0 (19.9—59.8)	1.9 (0.7—3.5)	0.9 (0.5—1.3)
IPV	Female	Kuwait	3.71 (1.34—7.14)	11.8 (1.12—26.2)	0.9 (0.4—1.9)	0.9 (0.1—2.0)
IPV	Female	Lebanon	126 (94.1—156)	11.2 (4.04—29.6)	11.4 (8.5—14.2)	0.5 (0.2—1.4)
IPV	Female	Libya	27.2 (5.11—60.0)	48.1 (11.2—114)	2.4 (0.4—5.0)	1.7 (0.4—4.0)
IPV	Female	Morocco	202 (0.162—597)	215 (32.7—495)	2.4 (0.0—6.9)	1.5 (0.2—3.5)
IPV	Female	Oman	1.96 (0.466—4.26)	4.62 (0.712—10.9)	0.5 (0.1—1.0)	0.3 (0.1—0.8)
IPV	Female	Palestine	4.72 (1.29—8.54)	22.9 (11.8—37.2)	0.9 (0.2—1.6)	1.4 (0.7—2.2)
IPV	Female	Qatar	0.823 (-0.317—2.40)	2.93 (-0.222—7.45)	1.1 (-0.3—3.3)	0.4 (0.0—1.2)
IPV	Female	Saudi Arabia	76.0 (-0.925—185)	88.3 (10.2—205)	2.0 (-0.0—4.7)	1.0 (0.1—2.4)
IPV	Female	Sudan	292 (-28.1—770)	804 (88.3—1,810)	4.8 (-0.5—13.0)	6.8 (0.7—15.1)
IPV	Female	Syrian Arab Republic	47.9 (18.6—96.0)	112 (77.6—159)	1.4 (0.5—2.7)	1.7 (1.1—2.4)
IPV	Female	Tunisia	44.8 (7.88—103)	48.6 (9.55—114)	1.7 (0.3—4.0)	1.0 (0.2—2.4)
IPV	Female	Türkiye	319 (53.9—589)	468 (-18.4—981)	1.7 (0.3—3.3)	1.4 (-0.1—3.0)
IPV	Female	United Arab Emirates	6.05 (-0.912—15.2)	16.3 (0.441—39.9)	1.9 (-0.2—4.7)	0.9 (0.1—2.4)
IPV	Female	Yemen	81.0 (7.53—220)	193 (26.9—507)	2.4 (0.3—6.1)	1.7 (0.3—4.5)
IPV	Female	South Asia	27,600 (-5,530—63,000)	34,500 (-13,300—86,200)	7.8 (-1.6—17.6)	5.0 (-1.8—12.5)
IPV	Female	Bangladesh	4,780 (-198—9,700)	1,900 (-446—4,080)	14.5 (0.5—28.3)	3.0 (-0.6—6.2)
IPV	Female	Bhutan	5.29 (0.202—15.4)	6.70 (-1.28—19.8)	3.5 (-0.1—10.4)	2.4 (-0.5—7.4)
IPV	Female	India	21,300 (-5,800—50,000)	30,100 (-12,900—76,000)	7.6 (-2.1—17.8)	5.6 (-2.4—14.4)
IPV	Female	Nepal	606 (-168—1,630)	572 (-195—1,760)	9.1 (-2.6—24.2)	4.7 (-1.6—14.6)

Risk Factor	Sex	Location	Number of Deaths		Rate of Deaths (per 100,000)	
			1990	2023	1990	2023
IPV	Female	Pakistan	933 (115—2,040)	1,950 (45.7—4,470)	2.9 (0.3—6.3)	2.5 (0.1—5.6)
IPV	Female	Southeast Asia, East Asia, and Oceania	53,900 (-19,600—140,000)	18,500 (-3,430—47,700)	9.5 (-3.8—25.6)	1.9 (-0.3—4.8)
IPV	Female	East Asia	48,900 (-20,500—130,000)	12,900 (-4,250—34,700)	11.6 (-5.3—32.0)	1.8 (-0.5—4.7)
IPV	Female	China	48,300 (-20,400—128,000)	12,100 (-4,110—32,800)	11.9 (-5.5—32.8)	1.7 (-0.5—4.6)
IPV	Female	Democratic People's Republic of Korea	351 (-82.5—929)	375 (-73.5—1,060)	4.6 (-1.1—12.5)	3.2 (-0.5—8.8)
IPV	Female	Taiwan	251 (-28.1—583)	446 (-211—1,330)	3.7 (-0.5—8.9)	3.8 (-1.8—10.8)
IPV	Female	Oceania	107 (3.38—209)	406 (96.7—741)	5.2 (0.1—10.3)	8.8 (2.2—15.9)
IPV	Female	American Samoa	0.323 (0.0298—0.676)	0.360 (0.00367—0.844)	2.1 (0.2—4.2)	2.2 (0.0—5.0)
IPV	Female	Cook Islands	0.387 (-0.0473—1.09)	0.169 (-0.0643—0.485)	6.1 (-0.8—16.8)	3.3 (-1.2—9.6)
IPV	Female	Fiji	10.8 (-3.95—25.2)	13.6 (-4.58—30.8)	4.3 (-1.7—10.2)	4.1 (-1.4—9.3)
IPV	Female	Guam	1.77 (-0.777—4.04)	1.71 (-0.364—3.89)	4.1 (-1.7—9.6)	3.0 (-0.7—6.7)
IPV	Female	Kiribati	1.38 (-0.615—3.01)	1.70 (-0.812—3.91)	5.4 (-2.5—11.8)	3.7 (-1.7—8.3)
IPV	Female	Marshall Islands	0.630 (-0.0299—1.54)	0.643 (-0.0381—1.44)	5.5 (-0.1—13.2)	4.6 (-0.3—10.4)
IPV	Female	Micronesia (Federated States of)	0.936 (0.125—2.05)	0.832 (0.0827—1.92)	3.2 (0.4—6.8)	2.3 (0.2—5.3)
IPV	Female	Nauru	0.290 (-0.00851—0.608)	0.307 (0.0295—0.664)	9.3 (-0.3—19.7)	7.3 (0.7—15.9)
IPV	Female	Niue	0.0326 (-0.00739—0.0786)	0.0197 (-0.0000122—0.0484)	4.3 (-1.0—10.1)	3.1 (0.0—7.5)
IPV	Female	Northern Mariana Islands	1.13 (0.174—2.26)	0.562 (0.0155—1.22)	6.8 (0.9—13.9)	3.5 (0.1—7.5)
IPV	Female	Palau	0.417 (0.0898—0.944)	0.358 (0.0654—0.843)	7.6 (1.6—17.2)	5.9 (1.2—14.2)
IPV	Female	Papua New Guinea	69.0 (6.66—134)	346 (90.5—627)	5.4 (0.6—10.2)	10.3 (2.9—18.3)
IPV	Female	Samoa	2.12 (0.121—4.92)	2.71 (0.294—5.96)	4.3 (0.2—9.6)	4.0 (0.4—8.8)
IPV	Female	Solomon Islands	8.29 (-0.667—16.9)	16.8 (-2.30—38.2)	9.0 (-0.7—18.6)	6.0 (-0.9—13.6)
IPV	Female	Tokelau	0.0235 (-0.00397—0.0595)	0.0175 (0.000375—0.0440)	4.5 (-0.8—11.2)	3.0 (0.1—7.5)
IPV	Female	Tonga	1.13 (0.283—2.16)	0.479 (0.00179—1.37)	3.8 (1.0—7.2)	1.3 (0.0—3.7)
IPV	Female	Tuvalu	0.213 (-0.0140—0.480)	0.271 (-0.0172—0.569)	6.3 (-0.4—14.4)	7.1 (-0.4—15.0)
IPV	Female	Vanuatu	1.43 (-1.06—3.96)	3.24 (-2.30—8.63)	3.3 (-2.4—9.2)	3.0 (-2.1—8.1)
IPV	Female	Southeast Asia	4,920 (598—10,400)	5,140 (437—11,800)	3.2 (0.3—6.8)	1.9 (0.2—4.3)
IPV	Female	Cambodia	195 (66.0—361)	147 (26.0—333)	5.7 (1.9—10.6)	2.3 (0.4—5.2)
IPV	Female	Indonesia	1,050 (164—2,410)	1,090 (149—2,440)	1.7 (0.3—3.9)	1.0 (0.1—2.2)
IPV	Female	Lao People's Democratic Republic	86.4 (29.2—175)	70.2 (25.8—149)	6.7 (2.2—13.6)	2.6 (0.9—5.5)
IPV	Female	Malaysia	43.5 (13.1—118)	61.8 (21.6—153)	0.8 (0.2—2.4)	0.5 (0.2—1.2)
IPV	Female	Maldives	1.24 (0.00423—3.25)	0.543 (0.0876—1.26)	2.1 (-0.0—5.6)	0.4 (0.1—0.9)
IPV	Female	Mauritius	11.7 (-0.641—38.5)	6.44 (0.139—17.7)	3.0 (-0.2—9.9)	1.2 (0.0—3.3)
IPV	Female	Myanmar	500 (95.5—959)	670 (84.3—1,660)	3.3 (0.5—6.4)	3.0 (0.4—7.6)
IPV	Female	Philippines	694 (368—1,100)	618 (300—1,160)	3.4 (1.8—5.4)	1.5 (0.7—2.9)
IPV	Female	Seychelles	0.485 (0.126—0.871)	0.822 (0.186—1.59)	2.0 (0.5—3.5)	2.2 (0.5—4.3)
IPV	Female	Sri Lanka	475 (-146—1,220)	207 (-34.6—557)	7.7 (-2.4—20.0)	2.2 (-0.3—5.9)
IPV	Female	Thailand	912 (99.8—1,960)	1,000 (-27.6—2,180)	4.4 (0.5—9.4)	3.2 (-0.1—6.8)
IPV	Female	Timor-Leste	25.5 (0.888—56.7)	28.1 (3.66—56.7)	10.2 (0.4—21.7)	6.1 (0.7—12.5)
IPV	Female	Viet Nam	915 (-337—2,650)	1,230 (-304—3,670)	4.2 (-1.7—12.1)	3.0 (-0.7—8.9)
IPV	Female	Sub-Saharan Africa	29,400 (8,230—54,000)	56,400 (16,100—107,000)	22.0 (5.6—41.4)	17.6 (4.2—33.9)
IPV	Female	Central Sub-Saharan Africa	3,640 (536—7,270)	6,250 (1,220—11,700)	23.9 (2.8—48.3)	15.9 (2.8—30.0)
IPV	Female	Angola	244 (41.3—521)	1,920 (305—4,260)	8.0 (0.9—17.2)	19.8 (2.9—44.7)
IPV	Female	Central African Republic	248 (37.3—520)	538 (137—1,000)	30.9 (4.4—67.9)	32.4 (6.7—62.4)
IPV	Female	Congo	268 (27.4—629)	697 (98.9—1,590)	41.3 (3.9—97.4)	41.9 (4.4—95.0)
IPV	Female	Democratic Republic of the Congo	2,820 (420—5,660)	2,630 (452—4,940)	26.7 (3.3—53.6)	10.1 (1.4—19.4)
IPV	Female	Equatorial Guinea	19.5 (4.17—38.2)	252 (33.6—536)	14.6 (2.2—29.5)	58.4 (7.3—121.1)

Risk Factor	Sex	Location	Number of Deaths		Rate of Deaths (per 100,000)	
			1990	2023	1990	2023
IPV	Female	Gabon	41.4 (4.02—84.0)	211 (34.5—434)	15.3 (1.2—31.5)	36.2 (5.4—75.6)
IPV	Female	Eastern Sub-Saharan Africa	17,200 (4,130—32,500)	23,700 (6,220—44,800)	34.7 (7.8—66.8)	20.9 (4.2—40.3)
IPV	Female	Burundi	606 (53.8—1,710)	335 (15.7—689)	40.4 (3.1—116.4)	10.3 (-0.0—21.0)
IPV	Female	Comoros	5.26 (1.76—12.1)	8.36 (3.40—17.8)	3.8 (1.1—9.2)	3.0 (1.2—6.7)
IPV	Female	Djibouti	11.6 (3.28—23.5)	98.5 (23.5—217)	9.8 (1.9—21.4)	23.8 (5.6—52.3)
IPV	Female	Eritrea	275 (72.3—587)	369 (102—800)	26.6 (6.2—56.4)	17.9 (4.6—38.6)
IPV	Female	Ethiopia	3,460 (1,020—7,040)	3,320 (940—6,250)	25.4 (6.9—53.5)	11.5 (2.7—22.7)
IPV	Female	Kenya	1,070 (294—2,050)	2,910 (867—5,710)	19.2 (4.4—37.4)	20.2 (5.0—40.0)
IPV	Female	Madagascar	522 (-15.8—1,060)	1,020 (-30.7—2,130)	15.2 (-1.7—30.6)	11.8 (-1.1—26.2)
IPV	Female	Malawi	923 (157—1,940)	1,870 (504—3,450)	36.2 (5.1—77.9)	37.3 (9.1—73.1)
IPV	Female	Mozambique	459 (143—857)	3,180 (677—6,220)	11.0 (2.8—21.0)	40.1 (7.5—79.7)
IPV	Female	Rwanda	435 (94.0—960)	500 (74.5—1,090)	21.0 (3.1—49.5)	12.9 (1.5—28.3)
IPV	Female	Somalia	176 (65.6—348)	508 (212—961)	7.9 (2.9—17.3)	8.6 (3.5—17.2)
IPV	Female	South Sudan	229 (58.4—475)	610 (198—1,240)	14.2 (2.6—31.4)	20.9 (5.7—44.1)
IPV	Female	Uganda	4,700 (920—8,800)	3,000 (758—5,580)	117.9 (19.5—221.5)	28.6 (5.1—54.7)
IPV	Female	United Republic of Tanzania	2,610 (544—4,960)	3,300 (748—6,440)	39.2 (6.5—77.7)	20.7 (3.9—41.6)
IPV	Female	Zambia	1,750 (345—3,130)	2,610 (510—4,880)	84.7 (12.8—159.7)	54.9 (9.4—107.7)
IPV	Female	Southern Sub-Saharan Africa	3,090 (1,400—5,170)	9,790 (3,140—18,900)	18.2 (7.7—31.9)	30.2 (9.0—59.0)
IPV	Female	Botswana	78.4 (6.09—161)	392 (83.0—761)	21.2 (1.6—43.7)	49.0 (9.6—95.8)
IPV	Female	Eswatini	85.9 (44.7—134)	439 (105—782)	29.2 (14.4—46.2)	116.1 (25.5—211.7)
IPV	Female	Lesotho	118 (36.2—204)	656 (148—1,200)	22.2 (6.4—39.3)	107.7 (22.6—202.6)
IPV	Female	Namibia	94.6 (45.4—164)	273 (105—511)	19.9 (9.7—34.7)	27.7 (9.8—55.1)
IPV	Female	South Africa	1,510 (846—2,380)	6,120 (2,070—12,000)	11.8 (6.6—18.8)	24.4 (8.1—48.4)
IPV	Female	Zimbabwe	1,200 (301—2,350)	1,910 (557—3,610)	46.2 (9.6—92.4)	42.6 (11.8—82.8)
IPV	Female	Western Sub-Saharan Africa	5,420 (1,840—10,400)	16,700 (5,480—31,300)	10.3 (3.1—20.1)	12.1 (3.5—23.6)
IPV	Female	Benin	87.3 (25.7—174)	253 (81.4—488)	6.4 (1.7—13.3)	6.6 (1.9—13.3)
IPV	Female	Burkina Faso	433 (147—1,050)	347 (148—665)	17.3 (5.2—42.7)	4.9 (1.9—10.3)
IPV	Female	Cabo Verde	5.51 (2.18—11.3)	6.64 (2.02—14.6)	5.5 (2.0—11.4)	3.6 (1.1—8.1)
IPV	Female	Cameroon	436 (75.9—851)	2,500 (514—4,980)	15.7 (2.1—31.3)	30.9 (6.1—63.9)
IPV	Female	Chad	233 (51.3—481)	754 (198—1,460)	13.5 (2.8—28.4)	16.3 (3.5—33.9)
IPV	Female	Côte d'Ivoire	736 (140—1,780)	1,110 (281—2,240)	26.5 (4.2—65.6)	13.4 (3.3—29.4)
IPV	Female	Gambia	10.8 (-0.0430—27.9)	91.1 (10.5—190)	4.3 (-0.1—11.4)	13.8 (1.5—29.5)
IPV	Female	Ghana	414 (144—764)	1,040 (285—2,300)	9.7 (3.0—18.4)	10.4 (2.5—23.7)
IPV	Female	Guinea	231 (24.3—486)	639 (151—1,220)	13.0 (1.2—27.9)	16.1 (3.6—31.8)
IPV	Female	Guinea-Bissau	30.3 (9.85—57.8)	101 (34.9—190)	10.0 (2.9—20.2)	16.7 (4.8—34.2)
IPV	Female	Liberia	122 (7.93—262)	301 (37.4—590)	15.6 (0.3—35.1)	17.0 (1.7—35.1)
IPV	Female	Mali	205 (26.4—464)	719 (160—1,430)	8.4 (0.9—19.2)	11.9 (2.5—24.6)
IPV	Female	Mauritania	39.1 (12.2—90.2)	68.7 (20.0—168)	6.7 (2.0—15.7)	5.3 (1.4—14.0)
IPV	Female	Niger	128 (47.0—278)	392 (135—858)	5.9 (1.9—13.9)	5.7 (1.9—13.0)
IPV	Female	Nigeria	1,940 (878—3,200)	7,410 (2,410—13,100)	7.7 (3.3—12.7)	11.2 (3.5—20.3)
IPV	Female	Sao Tome and Principe	1.30 (0.583—2.47)	3.41 (1.28—6.61)	3.8 (1.6—7.7)	4.9 (1.7—10.0)
IPV	Female	Senegal	86.4 (19.3—177)	233 (58.9—484)	4.1 (0.8—8.7)	4.4 (0.9—9.8)
IPV	Female	Sierra Leone	196 (-57.2—461)	435 (70.7—802)	14.7 (-4.5—35.3)	17.2 (2.2—32.8)
IPV	Female	Togo	78.3 (25.7—157)	293 (81.2—591)	7.6 (2.1—15.8)	11.2 (2.9—23.3)
SVAC	Both	Global	181,000 (69,800—333,000)	290,000 (119,000—537,000)	5.3 (2.0—9.9)	4.7 (1.9—8.6)
SVAC	Both	Central Europe, Eastern Europe, and Central Asia	19,300 (6,440—41,600)	18,500 (6,350—39,500)	5.9 (2.0—12.8)	4.9 (1.7—10.5)
SVAC	Both	Central Asia	1,090 (344—2,500)	1,510 (433—3,480)	2.7 (0.9—6.2)	2.2 (0.6—5.0)
SVAC	Both	Armenia	28.1 (8.82—66.5)	21.5 (7.20—47.3)	1.3 (0.4—3.0)	0.8 (0.3—1.7)
SVAC	Both	Azerbaijan	54.1 (16.5—116)	92.6 (32.4—201)	1.3 (0.4—2.7)	1.1 (0.4—2.4)

Risk Factor	Sex	Location	Number of Deaths		Rate of Deaths (per 100,000)	
			1990	2023	1990	2023
SVAC	Both	Georgia	61.6 (21.0—136)	56.8 (16.9—115)	1.4 (0.5—3.2)	1.6 (0.5—3.3)
SVAC	Both	Kazakhstan	476 (147—1,110)	488 (152—1,110)	4.4 (1.4—10.1)	3.3 (1.0—7.5)
SVAC	Both	Kyrgyzstan	88.5 (28.2—201)	74.3 (24.7—169)	3.5 (1.1—7.9)	1.6 (0.5—3.7)
SVAC	Both	Mongolia	36.7 (12.1—83.2)	79.8 (38.9—159)	3.3 (1.1—7.5)	3.3 (1.6—6.7)
SVAC	Both	Tajikistan	53.4 (16.5—126)	102 (23.7—271)	2.1 (0.6—4.9)	1.9 (0.4—5.1)
SVAC	Both	Turkmenistan	45.8 (14.0—105)	88.4 (25.6—229)	2.4 (0.7—5.4)	2.3 (0.7—6.0)
SVAC	Both	Uzbekistan	245 (70.8—585)	506 (103—1,240)	2.3 (0.7—5.5)	2.2 (0.4—5.3)
SVAC	Both	Central Europe	4,860 (1,570—10,400)	4,050 (1,510—8,260)	4.8 (1.6—10.3)	3.5 (1.3—7.1)
SVAC	Both	Albania	34.9 (13.7—70.0)	31.6 (11.6—63.5)	1.8 (0.7—3.6)	1.4 (0.5—2.7)
SVAC	Both	Bosnia and Herzegovina	187 (53.9—406)	136 (34.9—302)	5.6 (1.6—12.2)	4.0 (1.2—8.6)
SVAC	Both	Bulgaria	276 (75.8—608)	153 (45.8—316)	3.6 (1.0—7.9)	2.1 (0.7—4.3)
SVAC	Both	Croatia	210 (62.3—469)	166 (54.6—365)	4.9 (1.5—11.0)	3.7 (1.4—8.2)
SVAC	Both	Czechia	398 (123—801)	379 (135—744)	4.6 (1.4—9.1)	3.4 (1.3—6.5)
SVAC	Both	Hungary	784 (221—1,770)	395 (133—775)	8.8 (2.5—19.7)	3.9 (1.3—7.5)
SVAC	Both	Montenegro	17.4 (4.27—38.2)	18.7 (4.25—44.8)	3.8 (0.9—8.5)	3.0 (0.7—7.1)
SVAC	Both	North Macedonia	43.2 (11.9—90.7)	54.2 (11.7—125)	3.1 (0.8—6.7)	2.8 (0.7—6.3)
SVAC	Both	Poland	1,560 (515—3,360)	1,690 (668—3,290)	5.1 (1.7—11.1)	4.6 (1.8—8.9)
SVAC	Both	Romania	537 (177—1,130)	389 (135—781)	2.9 (0.9—6.1)	2.1 (0.7—4.2)
SVAC	Both	Serbia	441 (122—967)	334 (89.5—750)	5.9 (1.6—13.0)	3.5 (0.9—7.8)
SVAC	Both	Slovakia	178 (51.7—385)	146 (50.1—311)	4.4 (1.3—9.6)	2.8 (0.9—5.9)
SVAC	Both	Slovenia	119 (34.2—266)	96.5 (38.4—199)	7.3 (2.1—16.3)	4.1 (1.7—8.3)
SVAC	Both	Eastern Europe	13,400 (4,470—29,100)	13,000 (4,370—27,900)	7.3 (2.4—15.9)	6.8 (2.3—14.6)
SVAC	Both	Belarus	432 (144—914)	550 (210—1,160)	5.3 (1.8—11.1)	6.1 (2.3—13.0)
SVAC	Both	Estonia	87.7 (28.7—181)	63.6 (28.8—128)	6.9 (2.3—14.3)	4.9 (2.2—9.6)
SVAC	Both	Latvia	152 (49.2—325)	92.1 (37.9—184)	6.8 (2.2—14.5)	5.0 (2.2—9.9)
SVAC	Both	Lithuania	219 (71.2—463)	150 (57.1—321)	7.5 (2.5—16.0)	5.5 (2.1—11.6)
SVAC	Both	Republic of Moldova	187 (63.4—389)	139 (48.8—294)	5.7 (1.9—11.9)	4.0 (1.4—8.3)
SVAC	Both	Russian Federation	10,200 (3,410—22,500)	9,940 (3,260—21,500)	8.4 (2.8—18.5)	7.4 (2.5—15.9)
SVAC	Both	Ukraine	2,070 (688—4,430)	2,020 (720—4,420)	4.8 (1.6—10.2)	5.5 (2.0—12.0)
SVAC	Both	High-income	34,600 (12,600—63,900)	58,700 (22,700—96,400)	4.6 (1.7—8.5)	6.0 (2.2—9.8)
SVAC	Both	Australasia	776 (275—1,460)	1,320 (492—2,220)	4.9 (1.7—9.2)	4.8 (1.8—8.2)
SVAC	Both	Australia	627 (213—1,210)	1,100 (396—1,860)	4.7 (1.6—9.2)	4.7 (1.8—8.1)
SVAC	Both	New Zealand	149 (56.3—255)	222 (83.7—375)	5.7 (2.1—9.6)	5.3 (2.1—8.8)
SVAC	Both	High-income Asia Pacific	6,400 (1,750—13,400)	8,600 (1,910—18,200)	4.6 (1.3—9.6)	4.9 (1.1—10.2)
SVAC	Both	Brunei Darussalam	4.26 (1.09—8.96)	9.99 (1.87—24.1)	4.3 (0.9—9.7)	3.1 (0.5—7.5)
SVAC	Both	Japan	4,780 (1,160—10,300)	5,370 (1,190—11,300)	4.4 (1.1—9.5)	4.7 (1.0—9.8)
SVAC	Both	Republic of Korea	1,510 (474—3,660)	3,110 (759—6,770)	5.4 (1.7—12.9)	5.9 (1.4—12.7)
SVAC	Both	Singapore	103 (24.7—222)	112 (26.6—235)	4.8 (1.1—10.5)	2.3 (0.6—4.9)
SVAC	Both	High-income North America	11,000 (4,380—17,800)	32,500 (9,960—58,500)	4.9 (2.0—7.9)	10.3 (2.9—19.1)
SVAC	Both	Canada	912 (332—1,610)	1,980 (641—3,590)	4.1 (1.5—7.2)	6.0 (1.8—11.1)
SVAC	Both	Greenland	15.4 (4.20—32.4)	10.2 (3.08—20.3)	35.8 (10.0—74.5)	23.8 (7.3—47.4)
SVAC	Both	United States of America	10,100 (4,090—16,400)	30,500 (9,330—55,000)	5.0 (2.0—8.1)	10.8 (2.9—20.0)
SVAC	Both	Southern Latin America	1,530 (439—3,140)	2,130 (580—4,500)	4.5 (1.3—9.3)	3.7 (1.0—7.8)
SVAC	Both	Argentina	837 (249—1,780)	1,400 (357—3,030)	3.6 (1.1—7.7)	3.7 (0.9—7.9)
SVAC	Both	Chile	609 (166—1,260)	576 (177—1,150)	7.0 (1.9—14.4)	3.5 (1.1—6.8)
SVAC	Both	Uruguay	82.6 (23.3—192)	150 (37.2—337)	3.4 (1.0—7.7)	5.1 (1.3—11.4)
SVAC	Both	Western Europe	14,900 (4,950—30,500)	14,200 (5,310—27,400)	4.4 (1.5—8.9)	3.3 (1.3—6.1)
SVAC	Both	Andorra	1.42 (0.370—3.14)	1.66 (0.474—3.79)	3.1 (0.8—6.9)	2.0 (0.5—4.5)
SVAC	Both	Austria	361 (99.5—835)	322 (118—657)	5.3 (1.5—12.1)	3.4 (1.3—7.0)
SVAC	Both	Belgium	365 (108—768)	404 (151—749)	4.1 (1.2—8.5)	3.6 (1.4—6.5)

Risk Factor	Sex	Location	Number of Deaths		Rate of Deaths (per 100,000)	
			1990	2023	1990	2023
SVAC	Both	Cyprus	12.3 (2.76—27.7)	20.0 (4.97—45.6)	2.1 (0.5—4.8)	1.6 (0.4—3.5)
SVAC	Both	Denmark	364 (119—796)	250 (100—486)	8.0 (2.7—17.4)	4.2 (1.7—8.0)
SVAC	Both	Finland	310 (122—558)	224 (101—370)	7.3 (2.8—13.1)	4.3 (1.8—7.4)
SVAC	Both	France	3,260 (1,090—6,800)	2,850 (1,100—5,440)	6.6 (2.2—13.6)	4.2 (1.6—7.8)
SVAC	Both	Germany	3,930 (1,310—8,470)	3,330 (1,350—6,570)	5.2 (1.8—11.2)	3.9 (1.6—7.6)
SVAC	Both	Greece	101 (27.9—227)	150 (44.0—328)	1.1 (0.3—2.5)	1.4 (0.4—2.9)
SVAC	Both	Iceland	7.79 (2.19—16.9)	12.1 (4.29—24.4)	4.1 (1.2—8.9)	3.6 (1.3—7.1)
SVAC	Both	Ireland	129 (47.9—238)	165 (57.4—288)	4.9 (1.8—8.9)	3.6 (1.2—6.2)
SVAC	Both	Israel	121 (35.7—256)	201 (55.5—445)	3.6 (1.1—7.5)	2.6 (0.8—5.5)
SVAC	Both	Italy	1,350 (358—3,000)	1,070 (270—2,270)	2.5 (0.8—5.4)	1.6 (0.5—3.1)
SVAC	Both	Luxembourg	19.0 (6.09—39.3)	17.0 (7.25—31.8)	5.7 (1.8—11.8)	2.7 (1.1—5.0)
SVAC	Both	Malta	6.11 (1.35—13.9)	8.32 (2.05—18.4)	2.1 (0.5—4.8)	1.4 (0.4—3.0)
SVAC	Both	Monaco	1.12 (0.207—2.58)	1.25 (0.245—3.12)	3.8 (0.7—8.7)	3.3 (0.7—7.8)
SVAC	Both	Netherlands	514 (151—988)	614 (238—1,040)	4.0 (1.2—7.7)	3.5 (1.4—5.8)
SVAC	Both	Norway	203 (73.5—412)	192 (76.6—331)	5.7 (2.1—11.5)	3.8 (1.5—6.7)
SVAC	Both	Portugal	336 (87.3—752)	285 (83.7—625)	3.9 (1.1—8.7)	2.3 (0.8—4.8)
SVAC	Both	San Marino	0.474 (0.0930—1.09)	0.533 (0.105—1.34)	2.6 (0.5—5.9)	1.4 (0.3—3.4)
SVAC	Both	Spain	1,030 (362—1,800)	824 (276—1,520)	3.1 (1.1—5.3)	1.6 (0.6—3.0)
SVAC	Both	Sweden	375 (123—769)	403 (165—742)	4.9 (1.6—10.1)	4.1 (1.6—7.3)
SVAC	Both	Switzerland	400 (124—857)	266 (108—495)	6.6 (2.1—14.0)	2.9 (1.1—5.4)
SVAC	Both	United Kingdom	1,710 (616—3,270)	2,550 (979—4,470)	3.6 (1.3—6.6)	4.4 (1.7—7.5)
SVAC	Both	Latin America and Caribbean	7,050 (2,710—14,000)	16,400 (5,850—32,000)	3.4 (1.2—7.0)	3.5 (1.3—6.9)
SVAC	Both	Andean Latin America	437 (172—872)	1,280 (390—2,790)	2.2 (0.8—4.4)	2.7 (0.8—5.8)
SVAC	Both	Bolivia (Plurinational State of)	96.6 (31.6—201)	272 (79.1—625)	3.0 (1.0—6.4)	3.4 (1.0—7.9)
SVAC	Both	Ecuador	154 (63.6—312)	399 (120—827)	2.9 (1.2—6.0)	3.1 (0.9—6.3)
SVAC	Both	Peru	186 (70.5—365)	610 (193—1,290)	1.6 (0.6—3.2)	2.3 (0.7—4.8)
SVAC	Both	Caribbean	1,400 (439—3,130)	2,060 (649—4,160)	6.4 (2.0—14.1)	5.4 (1.7—10.9)
SVAC	Both	Antigua and Barbuda	1.18 (0.354—2.64)	2.25 (0.735—5.14)	3.2 (1.0—7.2)	2.7 (0.9—6.2)
SVAC	Both	Bahamas	5.17 (1.27—12.3)	10.9 (2.98—24.5)	3.6 (0.9—8.5)	3.3 (0.9—7.3)
SVAC	Both	Barbados	7.09 (1.49—17.7)	9.12 (1.79—21.5)	3.5 (0.8—8.6)	2.8 (0.6—6.4)
SVAC	Both	Belize	2.37 (0.812—4.78)	9.85 (3.05—19.3)	2.9 (0.9—6.0)	3.8 (1.0—7.4)
SVAC	Both	Bermuda	1.39 (0.407—3.13)	1.22 (0.359—2.75)	2.9 (0.8—6.6)	1.8 (0.6—3.9)
SVAC	Both	Cuba	418 (98.0—987)	468 (128—1,030)	5.2 (1.2—12.3)	3.9 (1.1—8.5)
SVAC	Both	Dominica	1.31 (0.311—3.31)	2.06 (0.452—5.11)	2.7 (0.7—6.7)	3.2 (0.7—7.6)
SVAC	Both	Dominican Republic	109 (31.5—244)	331 (85.0—739)	3.0 (0.8—6.6)	4.3 (1.1—9.7)
SVAC	Both	Grenada	2.35 (0.461—5.82)	3.93 (1.03—9.31)	4.3 (0.9—10.6)	3.8 (1.1—8.8)
SVAC	Both	Guyana	35.2 (10.1—80.6)	52.8 (12.6—115)	8.7 (2.3—20.1)	9.2 (2.1—20.0)
SVAC	Both	Haiti	512 (15.8—1,290)	753 (161—1,470)	15.4 (1.2—37.0)	9.7 (2.5—19.6)
SVAC	Both	Jamaica	40.0 (7.35—89.3)	72.0 (18.0—160)	3.1 (0.6—6.9)	3.1 (0.8—6.9)
SVAC	Both	Puerto Rico	136 (45.4—286)	141 (41.0—321)	5.2 (1.7—10.9)	4.4 (1.3—10.1)
SVAC	Both	Saint Kitts and Nevis	1.24 (0.382—2.84)	1.91 (0.635—4.01)	5.0 (1.6—11.2)	4.5 (1.5—9.6)
SVAC	Both	Saint Lucia	3.32 (0.747—8.21)	4.85 (1.22—11.5)	4.7 (1.1—11.6)	3.0 (0.8—6.9)
SVAC	Both	Saint Vincent and the Grenadines	2.50 (0.642—5.91)	3.88 (1.07—8.86)	4.5 (1.1—10.7)	3.8 (1.1—8.5)
SVAC	Both	Suriname	16.1 (4.62—37.5)	37.6 (9.05—82.0)	6.8 (2.0—16.2)	8.2 (2.0—18.0)
SVAC	Both	Trinidad and Tobago	56.1 (12.5—133)	80.9 (14.6—187)	8.2 (1.6—19.9)	6.5 (1.2—14.8)
SVAC	Both	United States Virgin Islands	3.08 (1.09—6.59)	3.66 (1.26—8.16)	4.7 (1.5—10.4)	4.6 (1.6—9.7)
SVAC	Both	Central Latin America	2,830 (1,050—5,840)	6,980 (2,140—14,500)	3.6 (1.3—7.7)	3.6 (1.1—7.4)
SVAC	Both	Colombia	391 (118—833)	1,030 (310—2,190)	2.2 (0.6—4.7)	2.5 (0.8—5.3)
SVAC	Both	Costa Rica	57.9 (21.0—110)	182 (60.6—354)	3.5 (1.2—6.7)	4.3 (1.4—8.4)
SVAC	Both	El Salvador	182 (67.8—394)	219 (85.0—432)	6.4 (2.4—14.9)	4.8 (1.9—9.6)

Risk Factor	Sex	Location	Number of Deaths		Rate of Deaths (per 100,000)	
			1990	2023	1990	2023
SVAC	Both	Guatemala	187 (79.5—378)	486 (189—970)	5.2 (2.2—10.6)	4.9 (1.8—9.8)
SVAC	Both	Honduras	70.3 (30.0—127)	163 (69.2—298)	3.2 (1.3—6.0)	2.6 (1.0—4.8)
SVAC	Both	Mexico	1,550 (564—3,290)	4,020 (954—8,210)	4.1 (1.4—9.1)	4.0 (0.9—8.2)
SVAC	Both	Nicaragua	67.8 (24.5—138)	178 (60.9—350)	3.9 (1.4—8.1)	4.0 (1.3—8.0)
SVAC	Both	Panama	32.3 (10.3—67.6)	95.9 (21.6—214)	2.3 (0.7—4.9)	3.0 (0.7—6.8)
SVAC	Both	Venezuela (Bolivarian Republic of)	292 (83.2—637)	604 (126—1,320)	3.1 (0.8—7.1)	3.0 (0.6—6.6)
SVAC	Both	Tropical Latin America	2,380 (817—5,220)	6,070 (2,390—11,600)	2.8 (0.9—6.3)	3.4 (1.3—6.4)
SVAC	Both	Brazil	2,350 (806—5,120)	5,880 (2,340—11,200)	2.8 (0.9—6.4)	3.4 (1.4—6.4)
SVAC	Both	Paraguay	36.9 (10.6—83.0)	183 (53.8—405)	1.9 (0.5—4.2)	4.1 (1.2—9.3)
SVAC	Both	North Africa and Middle East	3,310 (1,040—6,850)	7,530 (2,060—17,900)	2.0 (0.6—4.4)	1.9 (0.5—4.5)
SVAC	Both	Afghanistan	81.5 (24.3—181)	200 (54.8—492)	1.6 (0.5—3.6)	1.6 (0.4—3.8)
SVAC	Both	Algeria	253 (74.8—569)	564 (148—1,400)	2.0 (0.6—4.2)	1.9 (0.5—4.6)
SVAC	Both	Bahrain	7.78 (1.83—17.6)	25.9 (3.47—66.3)	4.0 (0.8—9.7)	3.4 (0.4—9.2)
SVAC	Both	Egypt	361 (96.3—836)	753 (97.6—1,850)	1.7 (0.4—4.1)	1.6 (0.2—3.9)
SVAC	Both	Iran (Islamic Republic of)	799 (217—1,660)	1,480 (399—3,420)	2.7 (0.8—5.8)	2.3 (0.6—5.5)
SVAC	Both	Iraq	231 (66.4—505)	850 (212—2,100)	2.9 (0.8—6.6)	3.5 (0.9—9.0)
SVAC	Both	Jordan	23.9 (4.71—57.3)	66.7 (8.64—159)	1.9 (0.3—4.9)	1.0 (0.1—2.5)
SVAC	Both	Kuwait	7.86 (2.42—18.1)	38.3 (9.09—89.0)	1.5 (0.4—4.0)	1.4 (0.2—3.5)
SVAC	Both	Lebanon	15.7 (4.19—36.6)	30.0 (7.51—66.4)	0.9 (0.2—2.1)	0.7 (0.2—1.6)
SVAC	Both	Libya	40.2 (12.3—85.8)	101 (26.0—243)	1.9 (0.6—4.0)	1.9 (0.5—4.7)
SVAC	Both	Morocco	276 (84.6—604)	503 (129—1,180)	2.0 (0.6—4.5)	1.9 (0.5—4.5)
SVAC	Both	Oman	10.8 (2.98—24.3)	33.8 (5.92—84.5)	1.8 (0.4—4.4)	1.7 (0.2—4.1)
SVAC	Both	Palestine	9.45 (1.73—23.3)	31.1 (3.14—86.9)	1.4 (0.2—3.5)	1.5 (0.1—4.6)
SVAC	Both	Qatar	4.86 (1.16—10.8)	22.0 (3.88—55.4)	3.3 (0.7—8.2)	1.7 (0.2—4.1)
SVAC	Both	Saudi Arabia	196 (51.6—442)	344 (57.3—844)	2.6 (0.7—5.8)	2.3 (0.3—5.5)
SVAC	Both	Sudan	215 (69.8—466)	604 (149—1,340)	2.3 (0.7—5.0)	2.7 (0.7—6.1)
SVAC	Both	Syrian Arab Republic	72.5 (22.9—161)	114 (30.8—262)	1.6 (0.5—3.8)	1.1 (0.3—2.6)
SVAC	Both	Tunisia	103 (28.2—252)	195 (40.9—458)	2.7 (0.7—6.9)	2.1 (0.4—4.8)
SVAC	Both	Türkiye	472 (123—1,070)	1,180 (244—2,720)	1.6 (0.4—3.8)	1.7 (0.4—4.0)
SVAC	Both	United Arab Emirates	20.8 (5.25—54.5)	77.0 (20.5—182)	3.4 (0.7—8.3)	1.5 (0.3—3.7)
SVAC	Both	Yemen	105 (33.4—238)	308 (79.5—716)	2.1 (0.7—5.0)	1.8 (0.5—4.1)
SVAC	Both	South Asia	38,900 (12,000—73,800)	82,000 (26,200—163,000)	6.6 (2.0—12.7)	6.6 (2.0—13.6)
SVAC	Both	Bangladesh	3,530 (1,290—6,560)	3,840 (1,370—7,000)	6.7 (2.5—13.0)	3.8 (1.3—7.4)
SVAC	Both	Bhutan	10.2 (3.28—19.6)	21.5 (6.04—46.8)	3.9 (1.2—7.8)	4.1 (1.1—8.9)
SVAC	Both	India	32,800 (9,860—64,100)	71,600 (22,300—140,000)	6.9 (2.1—13.4)	7.2 (2.2—14.7)
SVAC	Both	Nepal	640 (185—1,370)	1,280 (331—2,870)	6.3 (1.9—13.7)	6.0 (1.5—13.5)
SVAC	Both	Pakistan	1,960 (603—3,870)	5,260 (1,340—11,100)	3.7 (1.1—7.7)	4.1 (1.0—9.0)
SVAC	Both	Southeast Asia, East Asia, and Oceania	51,200 (17,900—105,000)	37,500 (14,400—71,100)	4.7 (1.5—9.9)	1.9 (0.7—3.6)
SVAC	Both	East Asia	43,400 (14,900—88,600)	21,700 (7,230—41,200)	5.2 (1.7—11.2)	1.5 (0.5—2.9)
SVAC	Both	China	42,600 (14,600—86,400)	20,300 (6,760—38,900)	5.3 (1.8—11.3)	1.5 (0.5—2.8)
SVAC	Both	Democratic People's Republic of Korea	387 (93.5—916)	528 (127—1,200)	2.8 (0.7—6.8)	2.3 (0.6—5.3)
SVAC	Both	Taiwan	416 (120—954)	951 (230—2,170)	3.3 (0.9—7.5)	3.9 (1.0—8.7)
SVAC	Both	Oceania	139 (31.0—309)	358 (93.1—797)	5.4 (1.1—12.6)	5.2 (1.1—11.2)
SVAC	Both	American Samoa	0.859 (0.193—2.06)	1.24 (0.238—3.05)	4.0 (0.9—10.2)	3.6 (0.7—8.9)
SVAC	Both	Cook Islands	0.949 (0.163—2.19)	0.710 (0.122—1.72)	8.8 (1.4—21.0)	6.0 (1.1—14.6)
SVAC	Both	Fiji	34.5 (7.92—79.0)	65.3 (11.8—156)	11.1 (2.4—26.7)	11.2 (2.0—26.5)
SVAC	Both	Guam	4.49 (0.992—10.4)	6.29 (1.54—14.4)	5.2 (1.1—12.5)	5.3 (1.3—12.2)

Risk Factor	Sex	Location	Number of Deaths		Rate of Deaths (per 100,000)	
			1990	2023	1990	2023
SVAC	Both	Kiribati	3.05 (0.870—6.13)	5.14 (1.49—11.3)	7.6 (2.0—16.0)	6.8 (1.8—15.2)
SVAC	Both	Marshall Islands	1.26 (0.273—2.75)	1.51 (0.321—3.62)	6.7 (1.5—15.9)	6.5 (1.3—15.9)
SVAC	Both	Micronesia (Federated States of)	3.84 (0.678—9.18)	5.36 (0.820—13.7)	8.5 (1.4—20.3)	8.6 (1.2—21.7)
SVAC	Both	Nauru	0.553 (0.135—1.18)	0.699 (0.189—1.55)	13.4 (2.9—31.6)	11.8 (2.4—27.0)
SVAC	Both	Niue	0.0983 (0.0182—0.240)	0.0760 (0.0130—0.195)	6.3 (1.2—15.2)	5.2 (1.0—13.2)
SVAC	Both	Northern Mariana Islands	1.84 (0.440—4.24)	2.25 (0.546—5.29)	7.5 (1.7—18.6)	6.4 (1.5—15.1)
SVAC	Both	Palau	0.564 (0.116—1.26)	0.830 (0.176—1.99)	6.2 (1.5—13.8)	5.7 (1.2—13.6)
SVAC	Both	Papua New Guinea	51.2 (9.72—119)	186 (46.2—396)	3.5 (0.6—8.3)	3.9 (1.0—8.7)
SVAC	Both	Samoa	4.15 (0.857—9.55)	5.73 (1.20—13.7)	4.7 (1.0—11.0)	4.4 (0.9—10.7)
SVAC	Both	Solomon Islands	17.2 (3.66—35.9)	50.1 (11.4—102)	12.3 (2.5—26.7)	12.0 (2.2—26.2)
SVAC	Both	Tokelau	0.0544 (0.0102—0.137)	0.0485 (0.00794—0.121)	5.2 (1.0—13.0)	3.9 (0.6—9.6)
SVAC	Both	Tonga	1.54 (0.282—3.85)	2.02 (0.342—4.83)	3.4 (0.6—8.6)	3.2 (0.5—7.8)
SVAC	Both	Tuvalu	0.360 (0.0778—0.856)	0.516 (0.0983—1.32)	6.5 (1.4—15.6)	7.3 (1.4—18.4)
SVAC	Both	Vanuatu	3.66 (0.782—8.60)	9.07 (1.64—21.5)	5.2 (1.1—12.3)	4.9 (0.9—11.8)
SVAC	Both	Southeast Asia	7,680 (2,650—15,100)	15,400 (4,880—30,800)	3.1 (1.1—6.2)	2.9 (0.9—5.8)
SVAC	Both	Cambodia	141 (51.6—274)	314 (108—616)	3.1 (1.1—6.0)	2.7 (0.9—5.3)
SVAC	Both	Indonesia	1,200 (314—2,740)	3,150 (778—6,870)	1.4 (0.4—3.4)	1.6 (0.4—3.5)
SVAC	Both	Lao People's Democratic Republic	81.9 (28.4—174)	162 (54.8—318)	4.0 (1.3—8.5)	3.6 (1.2—7.3)
SVAC	Both	Malaysia	160 (39.3—381)	352 (72.7—845)	1.9 (0.5—4.4)	1.4 (0.3—3.4)
SVAC	Both	Maldives	2.07 (0.544—4.16)	5.00 (1.28—11.5)	2.5 (0.7—5.2)	1.7 (0.4—4.0)
SVAC	Both	Mauritius	57.7 (17.9—127)	82.9 (20.6—198)	9.0 (2.8—20.0)	6.8 (1.8—16.0)
SVAC	Both	Myanmar	1,460 (529—3,370)	2,650 (898—5,060)	7.0 (2.5—16.3)	6.6 (2.3—12.8)
SVAC	Both	Philippines	699 (209—1,460)	1,870 (573—4,190)	2.4 (0.7—5.0)	2.6 (0.8—5.9)
SVAC	Both	Seychelles	1.49 (0.442—3.39)	2.68 (0.736—5.52)	3.4 (1.0—7.6)	3.1 (0.8—6.3)
SVAC	Both	Sri Lanka	1,330 (413—2,660)	1,110 (309—2,290)	12.7 (4.1—26.2)	5.7 (1.6—11.5)
SVAC	Both	Thailand	1,500 (488—3,130)	2,940 (878—5,970)	4.1 (1.3—8.7)	4.8 (1.5—9.5)
SVAC	Both	Timor-Leste	13.9 (4.07—28.1)	35.7 (10.7—70.7)	4.1 (1.1—9.0)	4.5 (1.3—9.1)
SVAC	Both	Viet Nam	1,030 (322—2,270)	2,700 (779—5,810)	2.8 (0.9—6.2)	3.3 (1.0—7.3)
SVAC	Both	Sub-Saharan Africa	26,400 (4,470—56,700)	69,900 (5,740—157,000)	11.7 (2.5—25.3)	12.6 (1.3—27.9)
SVAC	Both	Central Sub-Saharan Africa	2,560 (618—6,030)	6,110 (1,370—13,700)	10.3 (2.5—24.9)	9.1 (2.0—20.5)
SVAC	Both	Angola	310 (93.7—728)	1,880 (252—4,860)	7.1 (2.1—15.7)	11.6 (1.8—28.0)
SVAC	Both	Central African Republic	179 (19.4—463)	385 (32.9—931)	13.8 (2.1—34.8)	13.2 (1.2—31.1)
SVAC	Both	Congo	195 (5.67—570)	534 (-25.2—1,450)	17.2 (1.1—49.7)	16.7 (-0.2—44.5)
SVAC	Both	Democratic Republic of the Congo	1,820 (413—4,350)	2,960 (786—6,640)	10.6 (2.4—25.6)	7.0 (1.8—15.7)
SVAC	Both	Equatorial Guinea	17.6 (5.70—41.1)	187 (-7.78—499)	9.1 (2.9—21.3)	23.1 (-0.2—60.8)
SVAC	Both	Gabon	44.0 (13.9—107)	164 (11.1—424)	9.1 (2.8—22.1)	15.0 (1.1—38.4)
SVAC	Both	Eastern Sub-Saharan Africa	13,700 (177—33,000)	25,800 (2,390—58,300)	16.2 (1.1—37.4)	12.7 (1.4—28.2)
SVAC	Both	Burundi	469 (63.4—1,510)	482 (125—1,020)	17.7 (2.8—56.4)	8.0 (2.1—17.4)
SVAC	Both	Comoros	7.67 (2.13—17.1)	14.3 (3.30—36.1)	3.8 (1.0—8.4)	3.0 (0.7—7.5)
SVAC	Both	Djibouti	10.7 (3.48—23.3)	90.5 (9.66—234)	6.2 (2.0—13.1)	11.2 (1.6—28.0)
SVAC	Both	Eritrea	183 (36.6—470)	372 (90.7—846)	11.2 (2.6—28.0)	10.0 (2.5—22.9)
SVAC	Both	Ethiopia	2,260 (322—5,240)	3,690 (1,180—7,110)	9.8 (2.3—23.1)	7.3 (2.2—14.4)
SVAC	Both	Kenya	1,270 (195—2,760)	4,260 (202—9,510)	13.8 (3.3—29.3)	16.5 (1.1—37.7)
SVAC	Both	Madagascar	442 (134—949)	1,040 (278—2,430)	8.4 (2.6—17.5)	7.7 (2.1—17.6)
SVAC	Both	Malawi	1,170 (-63.4—2,670)	2,340 (-70.6—5,960)	26.6 (-0.3—59.9)	26.9 (-1.2—68.0)
SVAC	Both	Mozambique	326 (88.1—723)	2,800 (-378—8,300)	5.1 (1.5—11.3)	19.2 (-1.8—55.9)
SVAC	Both	Rwanda	536 (184—1,240)	864 (240—1,640)	17.2 (5.9—39.8)	12.9 (3.6—25.0)
SVAC	Both	Somalia	182 (56.2—435)	541 (122—1,220)	5.6 (1.6—13.4)	5.8 (1.3—13.2)

Risk Factor	Sex	Location	Number of Deaths		Rate of Deaths (per 100,000)	
			1990	2023	1990	2023
SVAC	Both	South Sudan	212 (60.3—510)	471 (97.0—1,070)	8.2 (2.4—19.6)	10.0 (2.2—22.8)
SVAC	Both	Uganda	3,540 (-908—10,300)	2,840 (95.6—6,750)	48.8 (-12.0—142.4)	15.5 (0.3—37.2)
SVAC	Both	United Republic of Tanzania	1,980 (11.3—4,980)	3,450 (26.1—8,260)	17.5 (0.8—44.7)	11.7 (0.3—27.6)
SVAC	Both	Zambia	1,120 (-138—3,080)	2,520 (-162—6,270)	31.8 (-2.3—86.5)	27.6 (-1.2—65.0)
SVAC	Both	Southern Sub-Saharan Africa	3,110 (935—6,690)	16,300 (-1,260—42,000)	11.2 (3.3—24.5)	27.4 (-2.1—70.3)
SVAC	Both	Botswana	134 (22.1—285)	545 (-75.3—1,260)	21.9 (4.2—46.4)	37.1 (-5.3—85.3)
SVAC	Both	Eswatini	28.0 (8.95—56.2)	250 (-50.4—617)	8.2 (2.3—17.4)	36.4 (-7.0—90.1)
SVAC	Both	Lesotho	76.5 (22.1—158)	486 (-55.5—1,190)	10.3 (2.9—21.5)	43.1 (-5.3—105.8)
SVAC	Both	Namibia	62.6 (20.8—126)	359 (2.01—890)	9.2 (3.0—18.9)	20.9 (0.1—52.7)
SVAC	Both	South Africa	1,810 (606—3,630)	13,000 (-864—33,400)	8.9 (2.8—18.1)	28.1 (-1.8—71.9)
SVAC	Both	Zimbabwe	992 (-161—2,600)	1,630 (-422—4,670)	21.1 (-2.8—54.5)	20.6 (-5.2—58.7)
SVAC	Both	Western Sub-Saharan Africa	7,020 (1,760—13,900)	21,700 (2,620—46,900)	8.2 (2.3—16.8)	9.6 (1.5—20.6)
SVAC	Both	Benin	102 (32.3—209)	381 (109—822)	5.4 (1.6—11.9)	6.6 (1.8—14.4)
SVAC	Both	Burkina Faso	701 (-76.4—2,020)	486 (112—1,040)	17.2 (-1.5—48.8)	5.1 (1.1—11.1)
SVAC	Both	Cabo Verde	10.1 (3.10—22.8)	21.2 (5.43—43.2)	6.0 (1.9—13.5)	6.1 (1.6—12.5)
SVAC	Both	Cameroon	327 (115—705)	1,920 (174—4,600)	7.5 (2.6—16.5)	13.0 (1.5—30.2)
SVAC	Both	Chad	223 (68.7—541)	729 (136—1,770)	8.6 (2.7—20.5)	10.6 (2.3—24.1)
SVAC	Both	Côte d'Ivoire	1,600 (-281—4,080)	2,860 (142—6,370)	29.5 (-3.3—73.7)	17.9 (1.4—39.3)
SVAC	Both	Gambia	18.6 (6.53—38.9)	130 (0.884—367)	4.8 (1.5—10.0)	11.0 (0.4—29.9)
SVAC	Both	Ghana	462 (150—1,030)	1,720 (391—3,960)	7.2 (2.3—15.6)	10.0 (2.5—22.3)
SVAC	Both	Guinea	165 (51.6—384)	543 (106—1,230)	5.9 (1.8—14.0)	8.7 (1.8—20.1)
SVAC	Both	Guinea-Bissau	30.7 (8.87—67.4)	127 (6.48—311)	7.4 (2.1—16.4)	13.3 (0.8—31.6)
SVAC	Both	Liberia	84.4 (25.4—186)	286 (49.7—659)	7.0 (2.3—15.5)	9.4 (1.8—20.7)
SVAC	Both	Mali	199 (61.6—470)	697 (132—1,600)	5.5 (1.6—12.8)	7.1 (1.5—16.0)
SVAC	Both	Mauritania	58.7 (16.7—128)	156 (33.8—400)	6.5 (1.8—14.0)	7.4 (1.6—18.3)
SVAC	Both	Niger	158 (48.0—337)	548 (142—1,220)	5.5 (1.7—12.2)	5.9 (1.4—13.1)
SVAC	Both	Nigeria	2,500 (846—4,810)	9,760 (409—22,400)	6.2 (2.1—12.9)	9.3 (1.0—20.9)
SVAC	Both	Sao Tome and Principe	1.23 (0.345—2.95)	3.57 (0.464—8.37)	2.4 (0.6—5.6)	3.1 (0.5—7.2)
SVAC	Both	Senegal	160 (54.6—330)	503 (130—1,090)	5.2 (1.7—10.4)	6.0 (1.5—13.0)
SVAC	Both	Sierra Leone	128 (42.7—294)	371 (90.2—795)	6.6 (2.1—15.0)	9.4 (2.4—20.5)
SVAC	Both	Togo	94.8 (27.7—228)	404 (48.6—940)	6.8 (2.0—15.8)	9.3 (1.4—21.0)
SVAC	Female	Global	82,200 (28,700—155,000)	124,000 (45,600—217,000)	4.7 (1.6—8.9)	3.9 (1.5—6.9)
SVAC	Female	Central Europe, Eastern Europe, and Central Asia	4,870 (1,620—10,400)	4,350 (1,520—9,620)	2.7 (0.9—5.7)	2.1 (0.8—4.5)
SVAC	Female	Central Asia	466 (149—1,030)	607 (175—1,390)	2.2 (0.7—4.8)	1.7 (0.5—3.9)
SVAC	Female	Armenia	14.5 (3.91—33.6)	8.64 (2.93—17.6)	1.2 (0.3—2.9)	0.5 (0.2—1.0)
SVAC	Female	Azerbaijan	21.2 (6.38—50.2)	31.9 (9.59—73.0)	0.9 (0.3—2.2)	0.7 (0.2—1.6)
SVAC	Female	Georgia	27.9 (9.32—64.3)	21.3 (4.88—44.4)	1.1 (0.4—2.6)	1.0 (0.3—1.8)
SVAC	Female	Kazakhstan	188 (56.4—418)	151 (47.3—323)	3.2 (1.0—7.1)	1.9 (0.6—4.1)
SVAC	Female	Kyrgyzstan	34.7 (10.7—77.5)	25.5 (7.44—59.7)	2.5 (0.8—5.7)	1.1 (0.3—2.5)
SVAC	Female	Mongolia	22.0 (7.31—47.6)	28.2 (14.8—48.7)	3.9 (1.3—8.5)	2.3 (1.2—4.0)
SVAC	Female	Tajikistan	27.4 (8.35—63.4)	48.7 (10.6—129)	2.0 (0.6—4.6)	1.8 (0.4—4.9)
SVAC	Female	Turkmenistan	21.3 (6.76—47.4)	38.6 (10.3—98.5)	2.1 (0.7—4.7)	2.1 (0.5—5.2)
SVAC	Female	Uzbekistan	108 (32.9—249)	254 (49.8—612)	2.0 (0.6—4.5)	2.2 (0.4—5.3)
SVAC	Female	Central Europe	1,030 (276—2,370)	732 (241—1,400)	1.9 (0.5—4.3)	1.1 (0.4—1.9)
SVAC	Female	Albania	9.86 (2.98—21.8)	8.54 (3.39—15.9)	1.0 (0.3—2.3)	0.7 (0.3—1.2)
SVAC	Female	Bosnia and Herzegovina	37.1 (7.45—96.9)	30.4 (4.37—80.6)	2.2 (0.4—5.8)	1.4 (0.3—3.6)
SVAC	Female	Bulgaria	67.6 (13.5—147)	33.2 (9.00—63.5)	1.6 (0.4—3.4)	0.8 (0.2—1.6)
SVAC	Female	Croatia	47.8 (11.4—125)	34.7 (8.19—70.7)	2.0 (0.5—5.3)	1.2 (0.3—2.3)
SVAC	Female	Czechia	113 (33.8—227)	105 (30.3—201)	2.3 (0.7—4.5)	1.5 (0.5—2.7)

Risk Factor	Sex	Location	Number of Deaths		Rate of Deaths (per 100,000)	
			1990	2023	1990	2023
SVAC	Female	Hungary	192 (45.0—489)	87.3 (27.2—165)	3.8 (0.9—9.5)	1.5 (0.5—2.9)
SVAC	Female	Montenegro	3.09 (0.481—8.23)	3.06 (0.640—6.67)	1.3 (0.2—3.6)	0.9 (0.2—1.9)
SVAC	Female	North Macedonia	12.2 (2.17—31.8)	14.1 (2.09—37.5)	1.7 (0.3—4.5)	1.3 (0.2—3.2)
SVAC	Female	Poland	258 (78.6—557)	237 (97.0—414)	1.6 (0.5—3.3)	1.1 (0.5—2.0)
SVAC	Female	Romania	110 (32.6—266)	49.1 (18.0—90.3)	1.1 (0.3—2.7)	0.5 (0.2—0.9)
SVAC	Female	Serbia	107 (20.6—292)	81.2 (15.1—207)	2.7 (0.5—7.6)	1.5 (0.3—3.7)
SVAC	Female	Slovakia	27.6 (5.94—69.7)	21.6 (7.09—42.2)	1.3 (0.3—3.1)	0.7 (0.2—1.4)
SVAC	Female	Slovenia	27.3 (7.16—67.8)	16.5 (6.44—30.4)	3.1 (0.8—7.5)	1.3 (0.5—2.3)
SVAC	Female	Eastern Europe	3,370 (1,080—7,340)	3,010 (981—6,850)	3.2 (1.0—6.9)	2.7 (0.9—5.9)
SVAC	Female	Belarus	110 (33.1—248)	140 (53.3—288)	2.4 (0.7—5.3)	2.6 (1.0—5.2)
SVAC	Female	Estonia	25.6 (8.28—54.1)	14.2 (5.90—25.8)	3.4 (1.1—7.1)	1.8 (0.8—3.1)
SVAC	Female	Latvia	39.6 (12.5—89.1)	20.4 (7.47—36.2)	3.0 (1.0—6.6)	1.7 (0.7—3.1)
SVAC	Female	Lithuania	49.5 (15.1—108)	28.3 (10.6—50.0)	3.0 (0.9—6.6)	1.7 (0.7—3.1)
SVAC	Female	Republic of Moldova	64.9 (22.5—134)	35.9 (12.7—73.6)	3.6 (1.3—7.5)	1.8 (0.7—3.6)
SVAC	Female	Russian Federation	2,540 (814—5,570)	2,320 (705—5,450)	3.7 (1.2—8.0)	3.0 (0.9—6.4)
SVAC	Female	Ukraine	543 (166—1,230)	450 (136—970)	2.1 (0.6—4.7)	2.2 (0.7—4.5)
SVAC	Female	High-income	12,500 (4,170—23,100)	20,700 (8,230—34,600)	3.1 (1.1—5.5)	4.1 (1.5—7.0)
SVAC	Female	Australasia	265 (94.9—480)	483 (189—788)	3.2 (1.2—5.8)	3.3 (1.3—5.4)
SVAC	Female	Australia	220 (76.2—405)	405 (153—660)	3.2 (1.1—5.9)	3.3 (1.3—5.4)
SVAC	Female	New Zealand	44.9 (17.7—73.6)	77.4 (30.9—130)	3.3 (1.3—5.4)	3.5 (1.4—6.0)
SVAC	Female	High-income Asia Pacific	2,790 (755—5,900)	3,290 (768—6,790)	3.8 (1.0—7.9)	3.7 (0.9—7.6)
SVAC	Female	Brunei Darussalam	1.73 (0.338—4.21)	4.11 (0.635—9.77)	4.1 (0.7—10.7)	2.7 (0.4—6.5)
SVAC	Female	Japan	2,150 (525—4,640)	2,100 (472—4,450)	3.7 (0.9—7.9)	3.6 (0.9—7.3)
SVAC	Female	Republic of Korea	593 (179—1,310)	1,140 (283—2,480)	4.1 (1.2—9.1)	4.5 (1.1—9.5)
SVAC	Female	Singapore	49.7 (11.4—108)	42.4 (9.96—89.9)	4.6 (1.0—10.1)	1.7 (0.4—3.6)
SVAC	Female	High-income North America	3,410 (1,270—5,550)	11,600 (3,400—21,100)	2.9 (1.1—4.6)	7.3 (1.9—13.8)
SVAC	Female	Canada	285 (102—517)	730 (258—1,250)	2.5 (0.9—4.4)	4.5 (1.5—7.9)
SVAC	Female	Greenland	4.50 (1.40—8.68)	3.45 (1.18—6.25)	24.3 (7.6—47.5)	17.2 (6.3—30.8)
SVAC	Female	United States of America	3,120 (1,180—5,080)	10,800 (3,160—19,900)	2.9 (1.1—4.7)	7.6 (2.0—14.2)
SVAC	Female	Southern Latin America	450 (131—941)	592 (147—1,250)	2.5 (0.7—5.2)	1.9 (0.5—3.9)
SVAC	Female	Argentina	256 (66.5—579)	352 (83.5—769)	2.1 (0.6—4.7)	1.7 (0.4—3.7)
SVAC	Female	Chile	166 (50.0—337)	200 (49.4—391)	3.7 (1.1—7.6)	2.3 (0.6—4.2)
SVAC	Female	Uruguay	28.6 (7.27—67.1)	40.2 (9.03—90.9)	2.1 (0.6—4.9)	2.4 (0.6—5.4)
SVAC	Female	Western Europe	5,620 (1,650—11,500)	4,790 (2,070—8,120)	3.0 (0.9—6.0)	2.0 (0.9—3.3)
SVAC	Female	Andorra	0.430 (0.0976—1.00)	0.518 (0.154—1.16)	2.0 (0.5—4.7)	1.2 (0.4—2.6)
SVAC	Female	Austria	108 (25.5—259)	72.8 (25.8—133)	2.8 (0.7—6.6)	1.5 (0.5—2.6)
SVAC	Female	Belgium	159 (45.9—310)	161 (58.7—274)	3.3 (1.0—6.5)	2.8 (1.0—4.7)
SVAC	Female	Cyprus	4.10 (0.578—11.5)	5.88 (1.24—12.6)	1.3 (0.2—3.7)	0.8 (0.2—1.7)
SVAC	Female	Denmark	137 (42.9—299)	79.7 (32.8—145)	5.7 (1.8—12.0)	2.5 (1.0—4.3)
SVAC	Female	Finland	117 (44.5—205)	93.7 (45.5—155)	5.1 (2.0—8.8)	3.4 (1.5—5.7)
SVAC	Female	France	1,270 (412—2,490)	1,040 (432—1,720)	4.7 (1.6—9.3)	2.7 (1.1—4.6)
SVAC	Female	Germany	1,550 (475—3,370)	1,050 (398—1,790)	3.6 (1.1—7.7)	2.3 (0.9—3.9)
SVAC	Female	Greece	29.6 (5.41—74.7)	31.5 (9.72—60.9)	0.6 (0.1—1.4)	0.5 (0.2—1.0)
SVAC	Female	Iceland	3.12 (0.918—6.85)	4.63 (1.65—8.91)	3.2 (1.0—7.1)	2.8 (1.0—5.2)
SVAC	Female	Ireland	39.5 (14.3—71.5)	41.0 (14.1—72.9)	2.9 (1.1—5.1)	1.7 (0.6—3.0)
SVAC	Female	Israel	42.5 (10.3—95.5)	59.0 (11.7—138)	2.3 (0.6—5.2)	1.3 (0.3—3.0)
SVAC	Female	Italy	488 (75.0—1,190)	377 (95.3—761)	1.5 (0.3—3.7)	0.9 (0.3—1.7)
SVAC	Female	Luxembourg	7.17 (2.22—15.1)	6.03 (2.71—10.3)	4.0 (1.3—8.2)	1.8 (0.8—3.2)
SVAC	Female	Malta	2.30 (0.462—5.04)	2.53 (0.446—5.13)	1.4 (0.3—3.1)	0.7 (0.2—1.4)
SVAC	Female	Monaco	0.504 (0.0908—1.28)	0.426 (0.0752—1.05)	3.2 (0.6—7.9)	2.1 (0.4—5.0)

Risk Factor	Sex	Location	Number of Deaths		Rate of Deaths (per 100,000)	
			1990	2023	1990	2023
SVAC	Female	Netherlands	248 (63.6—471)	253 (101—416)	3.6 (1.0—6.6)	2.8 (1.1—4.5)
SVAC	Female	Norway	70.1 (24.0—139)	71.9 (32.6—129)	3.7 (1.3—7.2)	2.8 (1.2—5.0)
SVAC	Female	Portugal	91.1 (17.3—234)	69.6 (16.8—132)	1.9 (0.4—4.8)	0.9 (0.3—1.8)
SVAC	Female	San Marino	0.138 (0.0220—0.337)	0.179 (0.0304—0.431)	1.4 (0.2—3.2)	0.8 (0.2—2.0)
SVAC	Female	Spain	369 (114—682)	218 (68.8—410)	2.0 (0.6—3.5)	0.8 (0.3—1.4)
SVAC	Female	Sweden	143 (41.8—285)	147 (51.8—245)	3.6 (1.1—7.0)	2.9 (1.0—4.8)
SVAC	Female	Switzerland	150 (46.3—316)	95.9 (39.3—165)	4.6 (1.5—9.3)	2.0 (0.8—3.5)
SVAC	Female	United Kingdom	592 (202—1,130)	915 (347—1,580)	2.2 (0.8—4.1)	2.9 (1.1—5.1)
SVAC	Female	Latin America and Caribbean	2,380 (647—4,990)	5,280 (1,440—10,000)	2.3 (0.5—5.0)	2.2 (0.6—4.1)
SVAC	Female	Andean Latin America	161 (48.1—323)	411 (96.8—867)	1.5 (0.4—3.1)	1.7 (0.4—3.5)
SVAC	Female	Bolivia (Plurinational State of)	34.7 (7.82—88.0)	91.8 (19.8—227)	2.0 (0.4—5.1)	2.2 (0.5—5.5)
SVAC	Female	Ecuador	44.7 (13.9—82.3)	100 (23.2—192)	1.5 (0.5—2.9)	1.5 (0.3—2.8)
SVAC	Female	Peru	82.0 (25.0—167)	219 (55.4—478)	1.3 (0.3—2.8)	1.6 (0.4—3.5)
SVAC	Female	Caribbean	632 (132—1,470)	746 (170—1,650)	5.5 (1.2—13.1)	3.8 (0.8—8.1)
SVAC	Female	Antigua and Barbuda	0.514 (0.0967—1.35)	0.864 (0.193—2.12)	2.5 (0.5—6.4)	2.0 (0.5—4.8)
SVAC	Female	Bahamas	2.05 (0.459—5.08)	4.26 (0.775—10.9)	2.8 (0.6—6.7)	2.5 (0.5—6.2)
SVAC	Female	Barbados	3.28 (0.469—9.19)	3.42 (0.349—8.96)	2.8 (0.4—7.5)	1.8 (0.2—4.4)
SVAC	Female	Belize	0.864 (0.194—1.88)	3.13 (0.680—6.64)	2.2 (0.4—5.1)	2.5 (0.5—5.3)
SVAC	Female	Bermuda	0.438 (0.104—1.09)	0.421 (0.0803—1.11)	1.7 (0.4—4.3)	1.0 (0.3—2.4)
SVAC	Female	Cuba	191 (39.4—451)	116 (22.2—264)	4.7 (1.0—11.1)	1.8 (0.4—4.0)
SVAC	Female	Dominica	0.611 (0.0960—1.65)	0.791 (0.119—2.17)	2.2 (0.4—5.7)	2.3 (0.4—6.1)
SVAC	Female	Dominican Republic	42.8 (9.75—98.7)	109 (25.5—271)	2.3 (0.5—5.7)	2.8 (0.6—6.8)
SVAC	Female	Grenada	1.39 (0.190—3.67)	1.91 (0.247—4.58)	4.4 (0.6—11.2)	3.4 (0.5—7.7)
SVAC	Female	Guyana	13.1 (3.51—31.1)	15.7 (2.95—38.3)	6.5 (1.5—16.2)	5.4 (1.0—13.2)
SVAC	Female	Haiti	274 (-48.0—788)	355 (15.2—828)	15.5 (-1.2—44.3)	8.7 (1.1—19.5)
SVAC	Female	Jamaica	21.0 (2.83—53.2)	32.8 (6.07—73.0)	3.0 (0.4—7.6)	2.7 (0.5—5.8)
SVAC	Female	Puerto Rico	29.0 (6.45—72.4)	32.9 (6.73—85.3)	2.0 (0.5—5.0)	1.4 (0.3—3.6)
SVAC	Female	Saint Kitts and Nevis	0.543 (0.144—1.39)	0.653 (0.191—1.51)	3.8 (1.1—9.5)	3.0 (0.8—6.9)
SVAC	Female	Saint Lucia	1.45 (0.276—3.82)	1.57 (0.292—3.70)	3.9 (0.7—10.2)	1.8 (0.3—4.2)
SVAC	Female	Saint Vincent and the Grenadines	1.03 (0.171—2.90)	1.27 (0.144—3.13)	3.4 (0.6—9.5)	2.4 (0.3—5.8)
SVAC	Female	Suriname	5.48 (1.37—12.5)	10.8 (2.34—24.0)	4.7 (1.1—11.0)	4.6 (1.0—10.1)
SVAC	Female	Trinidad and Tobago	21.3 (3.62—54.5)	29.1 (3.35—73.3)	6.5 (1.0—17.0)	4.4 (0.6—10.7)
SVAC	Female	United States Virgin Islands	1.11 (0.288—2.55)	1.04 (0.276—2.46)	3.5 (0.8—8.5)	2.1 (0.7—4.7)
SVAC	Female	Central Latin America	834 (194—1,820)	2,210 (455—4,390)	2.2 (0.5—5.3)	2.1 (0.4—4.3)
SVAC	Female	Colombia	133 (33.5—290)	303 (73.7—658)	1.6 (0.4—3.6)	1.4 (0.4—3.1)
SVAC	Female	Costa Rica	16.8 (5.14—31.7)	55.2 (14.2—96.7)	2.1 (0.6—4.2)	2.5 (0.7—4.4)
SVAC	Female	El Salvador	40.7 (13.0—80.8)	59.9 (16.4—121)	2.5 (0.8—5.2)	2.3 (0.6—4.7)
SVAC	Female	Guatemala	32.9 (12.3—65.2)	128 (31.0—245)	1.8 (0.8—3.7)	2.5 (0.6—5.1)
SVAC	Female	Honduras	18.7 (5.88—35.7)	47.5 (16.9—85.4)	1.7 (0.5—3.7)	1.5 (0.5—2.7)
SVAC	Female	Mexico	478 (103—1,140)	1,340 (209—3,050)	2.7 (0.5—6.7)	2.5 (0.4—5.8)
SVAC	Female	Nicaragua	20.0 (5.60—41.5)	55.5 (15.9—112)	2.2 (0.5—4.9)	2.5 (0.6—5.2)
SVAC	Female	Panama	8.01 (1.85—19.2)	26.6 (4.48—67.9)	1.3 (0.3—3.2)	1.6 (0.3—4.0)
SVAC	Female	Venezuela (Bolivarian Republic of)	85.0 (20.3—199)	193 (27.9—446)	2.0 (0.4—4.9)	1.8 (0.3—4.1)
SVAC	Female	Tropical Latin America	756 (183—1,660)	1,910 (613—3,330)	1.8 (0.4—4.2)	2.0 (0.7—3.5)
SVAC	Female	Brazil	744 (181—1,640)	1,870 (604—3,240)	1.8 (0.4—4.3)	2.0 (0.7—3.5)
SVAC	Female	Paraguay	12.1 (2.60—25.9)	44.5 (10.3—92.3)	1.2 (0.2—2.8)	2.0 (0.4—4.3)
SVAC	Female	North Africa and Middle East	1,110 (232—2,830)	2,260 (467—5,350)	1.4 (0.2—3.9)	1.2 (0.2—2.9)
SVAC	Female	Afghanistan	32.6 (4.68—91.1)	74.7 (15.1—183)	1.4 (0.2—4.0)	1.4 (0.2—3.5)
SVAC	Female	Algeria	93.1 (16.5—243)	175 (37.7—420)	1.5 (0.2—3.9)	1.2 (0.2—2.9)

Risk Factor	Sex	Location	Number of Deaths		Rate of Deaths (per 100,000)	
			1990	2023	1990	2023
SVAC	Female	Bahrain	1.91 (0.238—5.50)	5.06 (0.448—14.9)	2.8 (0.2—8.2)	1.9 (0.1—6.1)
SVAC	Female	Egypt	153 (25.1—439)	315 (35.6—838)	1.6 (0.2—5.0)	1.4 (0.1—4.0)
SVAC	Female	Iran (Islamic Republic of)	256 (50.2—626)	355 (84.4—907)	1.7 (0.3—4.1)	1.2 (0.3—3.1)
SVAC	Female	Iraq	69.1 (10.8—188)	217 (43.6—545)	1.9 (0.3—5.5)	2.0 (0.4—5.3)
SVAC	Female	Jordan	8.91 (0.735—26.8)	20.0 (1.80—55.5)	1.6 (0.1—4.9)	0.7 (0.1—2.0)
SVAC	Female	Kuwait	2.12 (0.427—6.13)	7.90 (0.858—20.5)	1.4 (0.2—4.2)	1.0 (0.1—2.7)
SVAC	Female	Lebanon	5.65 (0.745—16.8)	11.7 (2.15—28.7)	0.6 (0.1—1.9)	0.5 (0.1—1.3)
SVAC	Female	Libya	12.2 (2.22—33.0)	24.9 (5.52—59.8)	1.3 (0.2—3.3)	1.1 (0.2—2.6)
SVAC	Female	Morocco	114 (20.4—279)	160 (36.2—381)	1.6 (0.3—4.0)	1.2 (0.3—2.9)
SVAC	Female	Oman	2.52 (0.334—7.12)	6.19 (0.878—15.7)	1.1 (0.1—3.3)	0.9 (0.1—2.4)
SVAC	Female	Palestine	3.85 (0.202—11.8)	10.3 (0.887—31.9)	1.1 (0.1—3.6)	1.0 (0.1—3.4)
SVAC	Female	Qatar	0.710 (0.0823—2.12)	2.78 (0.296—7.61)	2.2 (0.1—6.7)	1.0 (0.1—3.0)
SVAC	Female	Saudi Arabia	35.8 (5.58—94.8)	62.5 (8.75—168)	1.3 (0.2—3.6)	1.3 (0.1—3.8)
SVAC	Female	Sudan	85.1 (11.2—205)	261 (30.7—653)	1.6 (0.3—4.2)	2.3 (0.3—5.9)
SVAC	Female	Syrian Arab Republic	24.6 (5.15—76.7)	41.8 (9.17—107)	1.1 (0.2—3.7)	0.8 (0.2—2.3)
SVAC	Female	Tunisia	42.6 (5.99—125)	71.8 (12.1—186)	2.3 (0.3—7.0)	1.5 (0.2—3.8)
SVAC	Female	Türkiye	126 (18.4—350)	328 (63.5—891)	0.9 (0.1—2.7)	0.9 (0.2—2.4)
SVAC	Female	United Arab Emirates	3.73 (0.598—10.3)	9.76 (1.99—23.4)	2.1 (0.2—6.8)	1.0 (0.2—2.7)
SVAC	Female	Yemen	34.3 (5.08—88.8)	93.3 (16.1—218)	1.3 (0.2—3.5)	1.1 (0.2—2.7)
SVAC	Female	South Asia	22,800 (6,460—42,100)	45,100 (14,800—82,900)	7.7 (2.1—14.3)	7.3 (2.3—14.0)
SVAC	Female	Bangladesh	1,410 (375—2,800)	955 (295—1,970)	4.8 (1.2—9.8)	2.0 (0.6—4.8)
SVAC	Female	Bhutan	3.63 (0.772—8.20)	6.62 (1.77—15.3)	2.9 (0.6—6.9)	2.7 (0.7—6.2)
SVAC	Female	India	20,600 (5,660—38,200)	42,300 (14,000—77,700)	8.7 (2.3—16.3)	8.5 (2.7—16.0)
SVAC	Female	Nepal	275 (58.9—658)	438 (100—1,080)	5.0 (1.0—11.6)	3.9 (0.9—9.7)
SVAC	Female	Pakistan	543 (96.4—1,410)	1,430 (277—3,110)	2.3 (0.4—6.0)	2.4 (0.4—5.2)
SVAC	Female	Southeast Asia, East Asia, and Oceania	26,000 (7,980—53,200)	12,700 (3,940—24,700)	4.7 (1.4—10.2)	1.2 (0.4—2.4)
SVAC	Female	East Asia	24,000 (7,280—49,000)	8,420 (2,250—17,100)	5.8 (1.7—12.3)	1.1 (0.3—2.2)
SVAC	Female	China	23,600 (7,210—48,300)	7,880 (2,140—15,300)	5.9 (1.7—12.5)	1.1 (0.3—2.0)
SVAC	Female	Democratic People's Republic of Korea	176 (34.1—477)	218 (41.4—550)	2.4 (0.5—6.5)	1.8 (0.3—4.4)
SVAC	Female	Taiwan	168 (42.4—414)	319 (52.6—777)	2.9 (0.7—7.1)	2.4 (0.4—5.9)
SVAC	Female	Oceania	58.2 (9.62—133)	154 (38.1—313)	4.9 (0.7—11.4)	4.8 (0.9—10.3)
SVAC	Female	American Samoa	0.190 (0.0259—0.532)	0.302 (0.0601—0.669)	2.0 (0.3—5.6)	1.7 (0.4—4.0)
SVAC	Female	Cook Islands	0.368 (0.0441—0.946)	0.256 (0.0310—0.616)	7.3 (0.8—19.3)	3.9 (0.5—9.5)
SVAC	Female	Fiji	17.1 (3.28—39.5)	34.8 (6.21—74.1)	11.3 (2.0—27.6)	11.6 (2.1—24.2)
SVAC	Female	Guam	1.32 (0.258—3.32)	0.951 (0.201—2.20)	3.9 (0.7—10.0)	1.5 (0.3—3.4)
SVAC	Female	Kiribati	1.06 (0.231—2.32)	1.81 (0.556—3.42)	5.6 (1.0—13.4)	4.8 (1.3—9.9)
SVAC	Female	Marshall Islands	0.336 (0.0455—0.937)	0.458 (0.0768—1.09)	4.3 (0.5—12.6)	4.3 (0.6—10.2)
SVAC	Female	Micronesia (Federated States of)	1.02 (0.105—2.83)	1.55 (0.180—3.83)	5.2 (0.5—14.8)	5.1 (0.5—12.5)
SVAC	Female	Nauru	0.264 (0.0571—0.598)	0.327 (0.0843—0.673)	14.5 (2.7—37.7)	11.7 (2.3—24.6)
SVAC	Female	Niue	0.0407 (0.00545—0.115)	0.0297 (0.00338—0.0729)	4.5 (0.6—12.3)	3.4 (0.5—8.2)
SVAC	Female	Northern Mariana Islands	0.446 (0.0840—1.18)	0.446 (0.0711—1.04)	5.0 (0.8—13.5)	2.7 (0.4—6.4)
SVAC	Female	Palau	0.253 (0.0395—0.639)	0.301 (0.0617—0.722)	5.5 (0.8—14.6)	4.7 (1.1—10.9)
SVAC	Female	Papua New Guinea	18.9 (2.01—50.7)	70.9 (17.2—146)	2.6 (0.2—7.7)	3.1 (0.6—6.6)
SVAC	Female	Samoa	1.18 (0.174—2.97)	1.90 (0.379—4.35)	2.9 (0.4—7.7)	3.0 (0.6—6.8)
SVAC	Female	Solomon Islands	9.99 (1.89—19.6)	29.3 (6.01—55.9)	16.2 (2.1—34.4)	14.8 (2.2—30.4)
SVAC	Female	Tokelau	0.0264 (0.00331—0.0746)	0.0221 (0.00269—0.0535)	4.8 (0.6—13.5)	3.4 (0.4—8.1)
SVAC	Female	Tonga	0.798 (0.101—2.27)	0.984 (0.140—2.47)	3.5 (0.4—10.0)	2.9 (0.4—7.5)
SVAC	Female	Tuvalu	0.154 (0.0213—0.403)	0.206 (0.0237—0.532)	5.2 (0.7—13.8)	5.6 (0.7—14.4)

Risk Factor	Sex	Location	Number of Deaths		Rate of Deaths (per 100,000)	
			1990	2023	1990	2023
SVAC	Female	Vanuatu	1.15 (0.161—2.81)	3.20 (0.382—7.83)	3.9 (0.5—10.0)	3.7 (0.4—9.3)
SVAC	Female	Southeast Asia	1,940 (496—4,580)	4,140 (1,260—8,640)	1.6 (0.4—3.8)	1.5 (0.5—3.2)
SVAC	Female	Cambodia	48.5 (7.52—103)	89.6 (24.9—190)	1.8 (0.3—4.0)	1.5 (0.4—3.1)
SVAC	Female	Indonesia	483 (89.3—1,410)	1,300 (266—3,030)	1.1 (0.2—3.3)	1.3 (0.3—3.1)
SVAC	Female	Lao People's Democratic Republic	30.8 (2.76—75.7)	47.3 (14.2—97.2)	2.7 (0.4—6.5)	2.1 (0.6—4.4)
SVAC	Female	Malaysia	47.6 (9.39—133)	93.9 (20.7—241)	1.2 (0.2—3.2)	0.8 (0.2—2.1)
SVAC	Female	Maldives	0.671 (0.124—1.61)	1.28 (0.350—3.03)	1.9 (0.3—4.9)	1.2 (0.3—3.2)
SVAC	Female	Mauritius	15.0 (3.06—38.6)	18.6 (2.14—53.3)	4.5 (0.9—11.8)	2.8 (0.4—7.8)
SVAC	Female	Myanmar	256 (51.8—724)	596 (118—1,560)	2.4 (0.5—7.0)	2.9 (0.6—7.5)
SVAC	Female	Philippines	124 (29.6—292)	403 (122—792)	0.9 (0.2—2.3)	1.1 (0.3—2.1)
SVAC	Female	Seychelles	0.269 (0.0604—0.774)	0.484 (0.104—1.25)	1.1 (0.3—3.3)	1.2 (0.2—3.1)
SVAC	Female	Sri Lanka	248 (55.2—559)	268 (71.8—661)	4.7 (1.1—11.2)	2.5 (0.7—5.9)
SVAC	Female	Thailand	403 (101—1,000)	706 (205—1,490)	2.2 (0.5—5.7)	2.0 (0.6—3.8)
SVAC	Female	Timor-Leste	8.17 (0.871—18.4)	21.1 (5.23—40.4)	4.9 (0.9—11.1)	5.4 (1.4—10.8)
SVAC	Female	Viet Nam	269 (49.1—674)	592 (166—1,240)	1.3 (0.2—3.6)	1.4 (0.4—2.9)
SVAC	Female	Sub-Saharan Africa	12,500 (-823—32,000)	33,200 (-2,780—81,300)	10.4 (-0.2—26.2)	11.3 (-0.6—27.7)
SVAC	Female	Central Sub-Saharan Africa	1,120 (-69.0—3,300)	2,560 (-44.8—6,810)	8.2 (-0.3—23.2)	7.2 (0.0—18.9)
SVAC	Female	Angola	82.6 (17.2—199)	930 (-98.6—2,820)	3.6 (0.9—8.7)	10.2 (-0.7—29.7)
SVAC	Female	Central African Republic	75.4 (-11.2—261)	165 (-25.3—457)	10.5 (-1.4—35.1)	10.6 (-1.5—29.1)
SVAC	Female	Congo	99.6 (-17.2—342)	298 (-57.5—882)	16.1 (-2.6—54.1)	18.4 (-3.2—52.7)
SVAC	Female	Democratic Republic of the Congo	843 (-63.7—2,410)	992 (158—2,400)	8.8 (-0.4—24.8)	4.5 (0.8—10.7)
SVAC	Female	Equatorial Guinea	5.55 (0.416—15.0)	94.1 (-19.1—289)	5.0 (0.9—13.6)	22.5 (-4.0—66.3)
SVAC	Female	Gabon	16.1 (1.25—46.0)	84.4 (-17.5—267)	6.4 (0.6—18.1)	14.7 (-2.8—46.3)
SVAC	Female	Eastern Sub-Saharan Africa	6,820 (-773—17,600)	12,700 (-642—30,400)	14.9 (-1.1—38.2)	11.9 (-0.3—29.2)
SVAC	Female	Burundi	258 (-25.0—889)	205 (29.1—512)	17.9 (-1.1—60.7)	6.9 (1.1—16.8)
SVAC	Female	Comoros	2.90 (0.665—7.12)	4.82 (1.01—11.4)	2.8 (0.6—6.8)	2.0 (0.4—4.8)
SVAC	Female	Djibouti	5.18 (1.00—13.9)	50.9 (-3.90—140)	5.9 (1.4—14.5)	13.2 (-0.3—34.6)
SVAC	Female	Eritrea	105 (-15.6—297)	174 (26.3—429)	11.5 (0.4—29.9)	9.6 (1.7—22.9)
SVAC	Female	Ethiopia	1,500 (-89.1—4,340)	2,240 (276—4,900)	12.7 (0.5—36.2)	9.1 (1.4—19.7)
SVAC	Female	Kenya	633 (-8.13—1,520)	2,060 (-176—5,100)	12.7 (0.4—30.4)	15.3 (-1.3—38.4)
SVAC	Female	Madagascar	203 (46.3—501)	475 (96.6—1,100)	7.2 (1.8—16.9)	6.7 (1.5—15.3)
SVAC	Female	Malawi	643 (-122—1,580)	1,200 (-241—3,550)	27.0 (-4.6—66.2)	25.7 (-5.5—74.5)
SVAC	Female	Mozambique	144 (6.01—380)	1,320 (-287—3,920)	4.0 (0.5—10.5)	16.0 (-3.2—46.6)
SVAC	Female	Rwanda	257 (70.3—654)	450 (53.4—936)	14.9 (4.3—35.8)	12.3 (1.6—25.6)
SVAC	Female	Somalia	86.2 (9.45—214)	263 (35.1—680)	5.0 (1.0—12.4)	5.3 (1.0—13.1)
SVAC	Female	South Sudan	95.1 (11.9—247)	233 (20.3—586)	7.4 (1.3—19.5)	9.4 (1.2—23.4)
SVAC	Female	Uganda	1,390 (-386—4,480)	1,050 (-185—2,840)	34.9 (-9.1—111.3)	10.3 (-1.4—28.7)
SVAC	Female	United Republic of Tanzania	812 (-119—2,250)	1,440 (-160—3,420)	13.1 (-1.4—37.3)	9.1 (-0.7—21.4)
SVAC	Female	Zambia	670 (-161—1,930)	1,480 (-276—3,960)	35.0 (-7.2—97.8)	30.0 (-4.8—75.5)
SVAC	Female	Southern Sub-Saharan Africa	1,510 (159—3,210)	8,300 (-1,460—21,500)	10.2 (1.7—22.2)	26.2 (-4.3—67.4)
SVAC	Female	Botswana	59.8 (-1.53—140)	263 (-63.6—663)	17.5 (0.2—39.8)	33.2 (-7.9—82.8)
SVAC	Female	Eswatini	13.1 (4.14—24.3)	161 (-52.2—401)	6.4 (1.7—12.7)	43.9 (-13.9—110.4)
SVAC	Female	Lesotho	39.9 (9.39—95.0)	302 (-66.2—836)	9.0 (2.1—20.7)	50.2 (-11.3—139.7)
SVAC	Female	Namibia	26.9 (5.97—61.3)	193 (-31.2—514)	7.4 (1.6—17.3)	21.4 (-2.4—56.5)
SVAC	Female	South Africa	633 (199—1,360)	6,100 (-901—15,800)	6.1 (1.8—13.4)	24.8 (-3.5—64.1)
SVAC	Female	Zimbabwe	741 (-161—1,960)	1,280 (-386—3,520)	29.4 (-5.7—78.7)	29.7 (-8.6—82.1)
SVAC	Female	Western Sub-Saharan Africa	3,020 (-220—7,300)	9,660 (-758—23,200)	6.7 (0.1—15.7)	7.8 (-0.3—18.1)
SVAC	Female	Benin	32.9 (4.96—83.4)	118 (15.2—292)	3.0 (0.6—7.9)	3.7 (0.6—9.1)

Risk Factor	Sex	Location	Number of Deaths		Rate of Deaths (per 100,000)	
			1990	2023	1990	2023
SVAC	Female	Burkina Faso	399 (-64.4—1,210)	214 (38.7—505)	17.3 (-2.2—51.7)	4.1 (0.8—9.9)
SVAC	Female	Cabo Verde	3.24 (0.618—7.96)	4.97 (1.15—10.7)	3.4 (0.7—8.1)	2.7 (0.6—6.0)
SVAC	Female	Cameroon	88.0 (7.38—238)	644 (-107—1,740)	3.8 (0.5—9.8)	8.1 (-1.1—22.1)
SVAC	Female	Chad	91.5 (-4.33—260)	296 (-13.2—839)	6.2 (0.4—17.1)	7.7 (0.3—20.8)
SVAC	Female	Côte d'Ivoire	765 (-242—2,070)	1,230 (-267—3,100)	28.7 (-8.0—76.7)	16.3 (-3.3—41.1)
SVAC	Female	Gambia	6.23 (1.04—15.0)	57.1 (-6.04—177)	3.1 (0.7—7.5)	9.0 (-0.5—26.0)
SVAC	Female	Ghana	190 (28.1—476)	742 (-15.5—2,130)	5.4 (1.0—13.2)	7.9 (0.0—21.6)
SVAC	Female	Guinea	69.4 (8.65—183)	239 (-0.850—640)	4.6 (0.8—11.6)	6.8 (0.4—17.5)
SVAC	Female	Guinea-Bissau	11.5 (2.18—29.9)	57.5 (-5.79—153)	5.0 (1.0—12.5)	10.9 (-0.7—27.3)
SVAC	Female	Liberia	34.7 (-0.867—96.6)	120 (-4.57—324)	5.5 (0.6—13.9)	7.8 (0.3—20.4)
SVAC	Female	Mali	78.8 (12.9—228)	298 (5.02—768)	4.1 (0.7—11.0)	5.8 (0.3—15.2)
SVAC	Female	Mauritania	24.9 (-0.106—69.1)	66.0 (5.84—206)	5.1 (0.4—13.1)	5.9 (0.8—18.0)
SVAC	Female	Niger	72.8 (1.91—192)	258 (14.4—619)	4.4 (0.7—11.3)	4.8 (0.8—10.5)
SVAC	Female	Nigeria	1,010 (43.5—2,350)	4,800 (-561—11,300)	4.8 (0.4—11.1)	8.1 (-0.6—19.5)
SVAC	Female	Sao Tome and Principe	0.575 (0.0985—1.36)	1.75 (-0.0395—4.45)	2.1 (0.4—5.0)	3.0 (0.1—7.5)
SVAC	Female	Senegal	54.8 (5.11—145)	189 (14.0—456)	3.2 (0.5—8.1)	4.1 (0.5—9.9)
SVAC	Female	Sierra Leone	44.0 (10.1—112)	148 (7.00—358)	4.3 (0.9—11.1)	6.9 (0.8—15.8)
SVAC	Female	Togo	39.4 (3.72—111)	178 (-9.42—496)	4.9 (0.8—13.0)	7.5 (-0.2—20.3)
SVAC	Male	Global	98,700 (39,500—187,000)	167,000 (70,500—322,000)	6.0 (2.4—11.5)	5.5 (2.3—10.6)
SVAC	Male	Central Europe, Eastern Europe, and Central Asia	14,400 (4,770—31,100)	14,200 (4,940—30,600)	9.7 (3.2—20.9)	8.0 (2.8—17.4)
SVAC	Male	Central Asia	624 (189—1,470)	901 (261—2,090)	3.2 (1.0—7.5)	2.7 (0.8—6.3)
SVAC	Male	Armenia	13.7 (3.66—33.0)	12.9 (3.43—29.6)	1.3 (0.3—3.1)	1.1 (0.3—2.4)
SVAC	Male	Azerbaijan	32.9 (10.1—72.0)	60.7 (21.5—120)	1.7 (0.5—3.7)	1.5 (0.5—3.0)
SVAC	Male	Georgia	33.7 (10.7—76.0)	35.5 (10.9—72.6)	1.8 (0.6—4.0)	2.3 (0.7—4.8)
SVAC	Male	Kazakhstan	288 (88.3—707)	337 (103—792)	5.6 (1.7—13.4)	4.9 (1.5—11.5)
SVAC	Male	Kyrgyzstan	53.8 (16.2—128)	48.8 (17.1—110)	4.5 (1.4—10.5)	2.1 (0.7—4.8)
SVAC	Male	Mongolia	14.7 (3.98—40.7)	51.6 (18.4—122)	2.6 (0.7—7.0)	4.4 (1.5—10.5)
SVAC	Male	Tajikistan	26.1 (7.97—64.1)	53.2 (12.1—126)	2.1 (0.7—5.2)	1.9 (0.4—4.8)
SVAC	Male	Turkmenistan	24.5 (7.20—58.2)	49.8 (14.7—127)	2.6 (0.8—6.2)	2.6 (0.7—6.5)
SVAC	Male	Uzbekistan	136 (37.0—321)	252 (54.4—626)	2.7 (0.8—6.2)	2.2 (0.5—5.4)
SVAC	Male	Central Europe	3,830 (1,260—8,070)	3,310 (1,170—6,860)	8.1 (2.6—17.0)	6.1 (2.1—12.5)
SVAC	Male	Albania	25.0 (9.11—51.8)	23.0 (7.17—49.5)	2.6 (1.0—5.3)	2.1 (0.7—4.5)
SVAC	Male	Bosnia and Herzegovina	150 (45.7—316)	106 (31.1—222)	9.2 (2.7—19.3)	6.7 (2.0—13.8)
SVAC	Male	Bulgaria	209 (61.6—484)	120 (31.5—257)	5.8 (1.7—13.4)	3.5 (1.0—7.5)
SVAC	Male	Croatia	162 (50.4—364)	131 (41.5—296)	8.3 (2.6—18.7)	6.5 (2.1—14.7)
SVAC	Male	Czechia	286 (87.8—579)	275 (101—545)	7.2 (2.2—14.7)	5.3 (2.0—10.2)
SVAC	Male	Hungary	592 (174—1,310)	308 (96.9—639)	14.3 (4.2—31.8)	6.5 (2.1—13.5)
SVAC	Male	Montenegro	14.3 (3.51—31.5)	15.7 (3.42—38.2)	6.4 (1.6—14.1)	5.2 (1.2—12.7)
SVAC	Male	North Macedonia	31.0 (9.40—60.2)	40.0 (10.1—89.3)	4.5 (1.4—9.0)	4.4 (1.3—9.2)
SVAC	Male	Poland	1,300 (431—2,810)	1,450 (555—2,880)	9.0 (3.0—19.5)	8.2 (3.1—16.3)
SVAC	Male	Romania	427 (147—913)	339 (103—711)	4.8 (1.6—10.2)	3.8 (1.2—7.9)
SVAC	Male	Serbia	334 (99.8—691)	253 (71.1—565)	9.3 (2.8—19.4)	5.6 (1.6—12.4)
SVAC	Male	Slovakia	151 (45.7—322)	125 (36.6—276)	7.9 (2.4—16.9)	4.9 (1.4—10.9)
SVAC	Male	Slovenia	91.6 (26.9—205)	80.0 (29.2—169)	12.1 (3.5—27.2)	7.0 (2.5—14.9)
SVAC	Male	Eastern Europe	10,000 (3,310—21,700)	9,950 (3,460—21,100)	12.1 (4.0—26.4)	11.4 (3.9—24.2)
SVAC	Male	Belarus	322 (108—665)	410 (153—883)	8.5 (2.8—17.7)	10.2 (3.8—22.1)
SVAC	Male	Estonia	62.1 (20.3—131)	49.3 (19.5—103)	10.9 (3.6—23.2)	8.1 (3.2—17.0)
SVAC	Male	Latvia	112 (36.3—242)	71.8 (25.5—146)	11.2 (3.6—24.3)	8.7 (3.0—17.8)
SVAC	Male	Lithuania	169 (56.0—361)	122 (40.3—275)	12.8 (4.2—27.2)	9.6 (3.2—21.6)

Risk Factor	Sex	Location	Number of Deaths		Rate of Deaths (per 100,000)	
			1990	2023	1990	2023
SVAC	Male	Republic of Moldova	122 (39.9—259)	104 (36.0—219)	8.2 (2.6—17.2)	6.3 (2.2—13.4)
SVAC	Male	Russian Federation	7,680 (2,550—16,800)	7,620 (2,650—15,900)	13.9 (4.6—30.7)	12.3 (4.2—25.9)
SVAC	Male	Ukraine	1,530 (519—3,220)	1,570 (560—3,470)	7.9 (2.7—16.7)	9.2 (3.3—20.4)
SVAC	Male	High-income	22,100 (8,260—41,600)	38,000 (15,100—65,900)	6.3 (2.3—11.8)	7.9 (3.0—13.5)
SVAC	Male	Australasia	511 (175—1,000)	839 (299—1,460)	6.6 (2.2—13.0)	6.3 (2.2—11.2)
SVAC	Male	Australia	407 (137—824)	694 (241—1,230)	6.3 (2.1—12.8)	6.2 (2.1—11.2)
SVAC	Male	New Zealand	105 (37.7—183)	145 (50.1—255)	8.1 (2.9—14.3)	7.0 (2.5—12.3)
SVAC	Male	High-income Asia Pacific	3,610 (987—7,630)	5,310 (1,150—11,400)	5.4 (1.5—11.5)	6.0 (1.3—12.8)
SVAC	Male	Brunei Darussalam	2.53 (0.746—5.39)	5.88 (1.26—15.2)	4.5 (1.2—9.8)	3.3 (0.6—8.7)
SVAC	Male	Japan	2,630 (638—5,880)	3,260 (701—6,940)	5.1 (1.2—11.5)	5.7 (1.2—12.2)
SVAC	Male	Republic of Korea	921 (278—2,270)	1,970 (486—4,330)	7.0 (2.2—17.9)	7.3 (1.8—16.2)
SVAC	Male	Singapore	52.8 (12.8—118)	69.4 (16.0—148)	4.9 (1.2—11.0)	2.9 (0.7—6.3)
SVAC	Male	High-income North America	7,610 (3,200—12,500)	20,900 (6,560—37,300)	7.1 (2.9—11.7)	13.4 (4.0—24.3)
SVAC	Male	Canada	627 (230—1,110)	1,250 (403—2,310)	5.8 (2.1—10.4)	7.6 (2.2—14.5)
SVAC	Male	Greenland	10.9 (2.81—24.2)	6.72 (1.89—14.4)	46.0 (12.0—100.4)	29.9 (8.3—64.8)
SVAC	Male	United States of America	6,970 (2,960—11,400)	19,700 (6,180—35,000)	7.2 (3.0—11.9)	14.1 (4.2—25.4)
SVAC	Male	Southern Latin America	1,080 (327—2,290)	1,540 (427—3,270)	6.7 (2.0—14.2)	5.6 (1.6—12.0)
SVAC	Male	Argentina	581 (186—1,220)	1,050 (272—2,270)	5.4 (1.7—11.4)	5.8 (1.5—12.6)
SVAC	Male	Chile	443 (118—954)	375 (123—764)	10.5 (2.9—22.6)	4.7 (1.5—9.6)
SVAC	Male	Uruguay	54.0 (15.2—126)	110 (28.4—246)	4.7 (1.3—10.9)	8.1 (2.1—18.2)
SVAC	Male	Western Europe	9,290 (3,250—19,000)	9,380 (3,550—19,000)	5.9 (2.1—12.1)	4.5 (1.7—9.3)
SVAC	Male	Andorra	0.989 (0.257—2.31)	1.14 (0.309—2.65)	4.0 (1.0—9.5)	2.6 (0.7—6.3)
SVAC	Male	Austria	253 (74.9—569)	249 (84.1—525)	8.1 (2.4—18.3)	5.5 (1.9—11.8)
SVAC	Male	Belgium	206 (55.5—461)	243 (80.1—537)	5.0 (1.3—11.0)	4.5 (1.4—9.7)
SVAC	Male	Cyprus	8.19 (2.21—19.5)	14.1 (3.42—33.0)	3.0 (0.8—7.1)	2.3 (0.6—5.5)
SVAC	Male	Denmark	227 (72.2—505)	170 (61.5—337)	10.4 (3.3—23.1)	5.9 (2.2—11.6)
SVAC	Male	Finland	193 (66.4—355)	130 (54.1—236)	9.6 (3.3—17.7)	5.2 (2.0—9.7)
SVAC	Male	France	1,990 (679—4,270)	1,810 (589—3,740)	8.6 (3.0—18.5)	5.7 (1.9—11.7)
SVAC	Male	Germany	2,380 (823—5,060)	2,280 (786—4,880)	7.0 (2.4—14.9)	5.5 (1.9—11.8)
SVAC	Male	Greece	71.6 (21.8—157)	118 (32.7—269)	1.7 (0.5—3.7)	2.4 (0.7—5.1)
SVAC	Male	Iceland	4.67 (1.28—10.2)	7.49 (2.50—15.7)	5.0 (1.4—10.9)	4.4 (1.5—9.0)
SVAC	Male	Ireland	89.2 (32.0—169)	124 (43.5—219)	6.9 (2.5—13.2)	5.6 (2.0—9.8)
SVAC	Male	Israel	78.9 (24.0—164)	142 (43.4—308)	4.9 (1.5—10.1)	3.8 (1.2—8.2)
SVAC	Male	Italy	858 (285—1,820)	696 (181—1,550)	3.6 (1.2—7.5)	2.2 (0.6—4.8)
SVAC	Male	Luxembourg	11.8 (3.64—26.0)	11.0 (4.07—24.0)	7.5 (2.3—16.5)	3.5 (1.3—7.7)
SVAC	Male	Malta	3.81 (1.03—8.64)	5.79 (1.62—13.4)	2.8 (0.7—6.3)	2.0 (0.6—4.6)
SVAC	Male	Monaco	0.619 (0.115—1.60)	0.825 (0.164—2.06)	4.5 (0.8—11.9)	4.5 (1.0—11.0)
SVAC	Male	Netherlands	266 (85.5—534)	361 (139—653)	4.4 (1.4—8.8)	4.2 (1.6—7.6)
SVAC	Male	Norway	132 (47.0—273)	120 (45.5—213)	7.6 (2.7—15.7)	4.8 (1.8—8.6)
SVAC	Male	Portugal	245 (69.8—545)	215 (62.0—499)	6.2 (1.8—13.8)	3.9 (1.2—8.9)
SVAC	Male	San Marino	0.336 (0.0695—0.818)	0.354 (0.0723—0.945)	3.9 (0.8—9.3)	2.0 (0.4—5.1)
SVAC	Male	Spain	661 (254—1,160)	606 (196—1,140)	4.3 (1.7—7.6)	2.5 (0.9—4.8)
SVAC	Male	Sweden	232 (78.9—494)	256 (97.6—497)	6.3 (2.1—13.5)	5.2 (1.9—10.2)
SVAC	Male	Switzerland	250 (77.9—565)	170 (51.2—360)	8.6 (2.7—19.5)	3.9 (1.2—8.2)
SVAC	Male	United Kingdom	1,120 (421—2,100)	1,640 (612—3,020)	5.0 (1.9—9.2)	5.9 (2.1—10.7)
SVAC	Male	Latin America and Caribbean	4,670 (1,830—9,510)	11,100 (3,780—22,800)	4.5 (1.7—9.4)	5.0 (1.7—10.4)
SVAC	Male	Andean Latin America	276 (100—554)	870 (264—1,930)	2.8 (1.0—5.9)	3.7 (1.1—8.2)
SVAC	Male	Bolivia (Plurinational State of)	62.0 (21.2—131)	181 (57.8—420)	4.2 (1.4—8.8)	4.7 (1.5—11.1)
SVAC	Male	Ecuador	110 (40.0—231)	299 (83.0—673)	4.3 (1.6—9.2)	4.8 (1.3—10.8)
SVAC	Male	Peru	104 (37.6—207)	391 (115—811)	1.8 (0.7—3.7)	2.9 (0.9—6.1)

Risk Factor	Sex	Location	Number of Deaths		Rate of Deaths (per 100,000)	
			1990	2023	1990	2023
SVAC	Male	Caribbean	769 (261—1,540)	1,310 (448—2,570)	7.3 (2.4—14.6)	7.2 (2.5—14.0)
SVAC	Male	Antigua and Barbuda	0.666 (0.237—1.53)	1.38 (0.537—2.98)	4.0 (1.4—9.1)	3.5 (1.4—7.6)
SVAC	Male	Bahamas	3.12 (0.838—7.28)	6.62 (2.07—13.8)	4.5 (1.2—10.3)	4.2 (1.3—8.9)
SVAC	Male	Barbados	3.81 (1.06—8.59)	5.70 (1.36—12.8)	4.4 (1.2—9.8)	3.9 (1.0—8.6)
SVAC	Male	Belize	1.51 (0.616—2.87)	6.72 (2.22—12.6)	3.6 (1.4—7.0)	5.1 (1.6—9.5)
SVAC	Male	Bermuda	0.951 (0.283—2.02)	0.799 (0.265—1.74)	4.2 (1.2—9.0)	2.6 (0.9—5.6)
SVAC	Male	Cuba	228 (58.3—534)	351 (98.3—759)	5.7 (1.5—13.5)	6.1 (1.7—13.2)
SVAC	Male	Dominica	0.697 (0.208—1.66)	1.27 (0.327—3.03)	3.3 (1.0—7.8)	4.0 (1.1—9.6)
SVAC	Male	Dominican Republic	66.1 (19.4—149)	222 (57.6—494)	3.6 (1.1—8.3)	5.9 (1.4—13.2)
SVAC	Male	Grenada	0.960 (0.303—2.14)	2.03 (0.659—4.57)	3.9 (1.2—8.6)	4.0 (1.3—9.0)
SVAC	Male	Guyana	22.1 (6.21—50.8)	37.2 (9.69—82.2)	11.0 (3.1—25.3)	13.2 (3.4—29.4)
SVAC	Male	Haiti	238 (43.5—501)	398 (131—779)	15.3 (2.8—32.3)	10.9 (3.5—21.3)
SVAC	Male	Jamaica	19.0 (4.14—43.0)	39.2 (10.9—84.3)	3.1 (0.7—7.1)	3.6 (1.0—7.7)
SVAC	Male	Puerto Rico	107 (38.4—226)	109 (34.0—243)	8.8 (3.2—18.5)	7.7 (2.2—17.4)
SVAC	Male	Saint Kitts and Nevis	0.701 (0.238—1.53)	1.26 (0.440—2.53)	6.4 (2.3—14.2)	6.2 (2.1—12.4)
SVAC	Male	Saint Lucia	1.87 (0.547—4.38)	3.28 (0.922—7.61)	5.6 (1.6—13.1)	4.1 (1.1—9.7)
SVAC	Male	Saint Vincent and the Grenadines	1.47 (0.504—3.19)	2.61 (0.823—5.69)	5.5 (1.8—11.9)	5.1 (1.6—10.9)
SVAC	Male	Suriname	10.6 (3.22—25.1)	26.8 (6.94—57.1)	9.1 (2.8—21.7)	12.2 (3.1—26.1)
SVAC	Male	Trinidad and Tobago	34.8 (9.10—79.3)	51.8 (11.4—118)	10.0 (2.3—23.1)	8.6 (1.9—19.6)
SVAC	Male	United States Virgin Islands	1.97 (0.716—4.21)	2.62 (0.932—5.72)	6.0 (2.1—13.1)	7.2 (2.6—15.6)
SVAC	Male	Central Latin America	2,000 (737—4,110)	4,770 (1,440—10,500)	5.0 (1.8—10.5)	5.1 (1.5—11.2)
SVAC	Male	Colombia	257 (85.2—539)	724 (219—1,510)	2.8 (0.9—5.9)	3.7 (1.1—7.7)
SVAC	Male	Costa Rica	41.1 (15.1—80.1)	127 (42.1—256)	4.8 (1.8—9.6)	6.0 (2.0—12.3)
SVAC	Male	El Salvador	142 (49.5—323)	159 (67.7—316)	10.8 (3.7—24.7)	8.3 (3.5—16.6)
SVAC	Male	Guatemala	154 (62.8—327)	358 (143—736)	8.7 (3.5—18.3)	7.6 (3.0—15.6)
SVAC	Male	Honduras	51.6 (21.4—98.9)	115 (42.8—230)	4.8 (2.0—9.4)	3.8 (1.3—7.5)
SVAC	Male	Mexico	1,070 (392—2,260)	2,680 (709—6,120)	5.6 (1.9—12.0)	5.6 (1.5—12.8)
SVAC	Male	Nicaragua	47.8 (15.7—98.9)	123 (43.0—237)	5.8 (2.0—12.4)	5.8 (1.9—11.4)
SVAC	Male	Panama	24.3 (8.21—50.5)	69.4 (17.0—147)	3.3 (1.1—7.1)	4.5 (1.1—9.6)
SVAC	Male	Venezuela (Bolivarian Republic of)	207 (61.6—443)	411 (90.6—884)	4.3 (1.2—9.5)	4.4 (1.0—9.5)
SVAC	Male	Tropical Latin America	1,630 (602—3,570)	4,160 (1,470—8,180)	3.8 (1.3—8.3)	4.9 (1.7—9.6)
SVAC	Male	Brazil	1,600 (593—3,520)	4,020 (1,420—7,900)	3.8 (1.4—8.3)	4.8 (1.7—9.5)
SVAC	Male	Paraguay	24.8 (7.83—59.1)	139 (42.4—306)	2.5 (0.8—5.8)	6.4 (1.9—14.3)
SVAC	Male	North Africa and Middle East	2,200 (684—4,470)	5,270 (1,360—12,200)	2.6 (0.9—5.4)	2.5 (0.7—6.1)
SVAC	Male	Afghanistan	48.9 (15.4—110)	125 (35.4—333)	1.9 (0.6—4.4)	1.8 (0.5—5.0)
SVAC	Male	Algeria	160 (47.8—355)	389 (93.9—938)	2.5 (0.8—5.4)	2.5 (0.6—6.2)
SVAC	Male	Bahrain	5.87 (1.34—13.9)	20.8 (2.84—51.6)	4.9 (1.1—12.0)	4.6 (0.5—12.5)
SVAC	Male	Egypt	208 (64.0—451)	438 (60.3—1,120)	1.8 (0.5—3.8)	1.6 (0.2—4.4)
SVAC	Male	Iran (Islamic Republic of)	543 (145—1,090)	1,130 (299—2,690)	3.7 (1.1—7.6)	3.4 (0.9—8.3)
SVAC	Male	Iraq	162 (48.1—357)	633 (152—1,570)	3.9 (1.2—8.9)	5.0 (1.2—12.9)
SVAC	Male	Jordan	15.0 (3.30—34.3)	46.7 (6.54—114)	2.2 (0.5—5.5)	1.3 (0.2—3.2)
SVAC	Male	Kuwait	5.75 (1.91—12.3)	30.4 (8.34—68.3)	1.6 (0.5—3.6)	1.6 (0.3—3.8)
SVAC	Male	Lebanon	10.0 (2.87—22.9)	18.3 (4.53—42.0)	1.1 (0.3—2.6)	0.9 (0.2—2.1)
SVAC	Male	Libya	27.9 (8.25—59.0)	76.4 (18.9—182)	2.4 (0.8—5.2)	2.8 (0.7—6.6)
SVAC	Male	Morocco	162 (50.8—345)	343 (89.2—841)	2.4 (0.8—5.3)	2.5 (0.7—6.2)
SVAC	Male	Oman	8.30 (2.34—18.5)	27.6 (4.81—70.7)	2.3 (0.7—5.6)	2.4 (0.3—6.3)
SVAC	Male	Palestine	5.60 (1.34—13.9)	20.8 (2.36—56.8)	1.7 (0.4—4.4)	2.1 (0.2—5.9)
SVAC	Male	Qatar	4.15 (1.01—9.78)	19.3 (3.60—47.1)	4.0 (0.9—9.6)	2.1 (0.3—5.1)
SVAC	Male	Saudi Arabia	160 (42.8—355)	282 (47.7—729)	3.5 (1.0—8.0)	2.9 (0.4—6.8)

Risk Factor	Sex	Location	Number of Deaths		Rate of Deaths (per 100,000)	
			1990	2023	1990	2023
SVAC	Male	Sudan	130 (45.3—279)	343 (77.9—806)	2.9 (1.0—6.7)	3.0 (0.7—7.4)
SVAC	Male	Syrian Arab Republic	47.9 (16.7—94.9)	72.1 (20.2—159)	2.0 (0.7—4.5)	1.3 (0.4—3.0)
SVAC	Male	Tunisia	60.7 (19.4—134)	123 (27.3—293)	3.2 (1.0—6.9)	2.7 (0.6—6.4)
SVAC	Male	Türkiye	345 (90.6—863)	849 (191—1,910)	2.3 (0.6—5.6)	2.6 (0.6—5.9)
SVAC	Male	United Arab Emirates	17.1 (4.11—47.1)	67.2 (18.2—157)	4.2 (0.9—11.4)	1.8 (0.4—4.3)
SVAC	Male	Yemen	70.3 (24.1—152)	215 (61.1—488)	3.0 (1.0—7.2)	2.5 (0.7—5.6)
SVAC	Male	South Asia	16,100 (5,340—33,000)	36,800 (10,400—80,000)	5.5 (2.0—11.0)	5.8 (1.6—12.6)
SVAC	Male	Bangladesh	2,120 (774—4,080)	2,890 (995—5,270)	8.2 (3.0—16.0)	5.4 (1.7—10.2)
SVAC	Male	Bhutan	6.54 (2.14—14.7)	14.9 (3.98—33.7)	4.8 (1.5—10.3)	5.5 (1.5—12.3)
SVAC	Male	India	12,200 (3,690—26,800)	29,200 (7,600—64,000)	5.2 (1.7—11.2)	5.9 (1.5—12.8)
SVAC	Male	Nepal	365 (108—735)	841 (220—1,830)	7.7 (2.3—15.6)	8.3 (2.2—18.1)
SVAC	Male	Pakistan	1,410 (445—2,910)	3,830 (1,040—8,350)	4.8 (1.6—9.7)	5.6 (1.5—12.4)
SVAC	Male	Southeast Asia, East Asia, and Oceania	25,300 (9,080—48,900)	24,800 (9,640—48,300)	4.7 (1.7—9.3)	2.6 (1.0—4.9)
SVAC	Male	East Asia	19,400 (6,370—39,600)	13,300 (4,860—25,600)	4.6 (1.5—9.7)	1.9 (0.7—3.6)
SVAC	Male	China	19,000 (6,160—38,600)	12,400 (4,550—24,000)	4.7 (1.5—9.8)	1.8 (0.7—3.6)
SVAC	Male	Democratic People's Republic of Korea	212 (55.7—521)	310 (82.7—755)	3.4 (0.9—8.5)	2.9 (0.8—7.1)
SVAC	Male	Taiwan	248 (79.1—555)	632 (169—1,420)	3.6 (1.1—8.2)	5.3 (1.5—11.8)
SVAC	Male	Oceania	80.7 (19.2—182)	204 (50.0—504)	5.9 (1.5—13.9)	5.6 (1.2—14.5)
SVAC	Male	American Samoa	0.669 (0.159—1.59)	0.939 (0.157—2.55)	6.1 (1.4—15.6)	5.6 (0.9—15.2)
SVAC	Male	Cook Islands	0.581 (0.113—1.40)	0.455 (0.0856—1.17)	10.0 (1.9—23.9)	8.2 (1.6—20.9)
SVAC	Male	Fiji	17.4 (4.50—40.4)	30.5 (4.44—84.0)	10.8 (2.8—25.7)	10.6 (1.5—29.1)
SVAC	Male	Guam	3.17 (0.756—7.17)	5.34 (1.28—12.3)	6.2 (1.4—14.3)	8.9 (2.1—20.6)
SVAC	Male	Kiribati	1.99 (0.585—4.34)	3.33 (0.810—8.00)	9.7 (3.1—21.4)	8.7 (2.1—20.9)
SVAC	Male	Marshall Islands	0.922 (0.217—2.01)	1.05 (0.207—2.78)	9.1 (2.4—21.4)	8.6 (1.6—23.5)
SVAC	Male	Micronesia (Federated States of)	2.82 (0.557—6.87)	3.81 (0.583—9.84)	11.9 (2.3—30.4)	12.4 (1.8—32.3)
SVAC	Male	Nauru	0.289 (0.0738—0.652)	0.372 (0.0956—1.01)	11.8 (2.9—27.6)	11.4 (2.7—30.5)
SVAC	Male	Niue	0.0575 (0.0143—0.134)	0.0464 (0.00918—0.128)	8.0 (2.0—18.7)	7.3 (1.6—19.8)
SVAC	Male	Northern Mariana Islands	1.39 (0.341—3.26)	1.80 (0.456—4.28)	9.5 (2.4—21.3)	9.7 (2.3—23.5)
SVAC	Male	Palau	0.311 (0.0803—0.738)	0.529 (0.104—1.35)	6.9 (1.7—15.7)	6.8 (1.3—17.3)
SVAC	Male	Papua New Guinea	32.3 (7.81—72.5)	115 (26.6—280)	4.3 (1.0—10.5)	4.6 (1.1—11.7)
SVAC	Male	Samoa	2.97 (0.655—6.95)	3.82 (0.677—10.3)	6.4 (1.3—14.6)	5.9 (1.0—16.0)
SVAC	Male	Solomon Islands	7.22 (1.44—17.1)	20.8 (3.76—50.2)	9.3 (1.8—21.8)	9.3 (1.6—23.2)
SVAC	Male	Tokelau	0.0279 (0.00642—0.0700)	0.0264 (0.00519—0.0695)	5.5 (1.3—13.8)	4.3 (0.9—11.4)
SVAC	Male	Tonga	0.747 (0.159—1.70)	1.03 (0.170—2.89)	3.4 (0.7—7.7)	3.6 (0.6—10.2)
SVAC	Male	Tuvalu	0.205 (0.0546—0.459)	0.310 (0.0760—0.772)	8.2 (2.3—17.9)	8.8 (2.1—22.0)
SVAC	Male	Vanuatu	2.51 (0.624—5.94)	5.87 (1.22—14.4)	6.5 (1.7—15.3)	6.2 (1.3—15.2)
SVAC	Male	Southeast Asia	5,740 (2,190—11,000)	11,200 (3,580—23,100)	4.9 (1.9—9.2)	4.3 (1.3—8.9)
SVAC	Male	Cambodia	92.5 (32.9—183)	225 (79.4—457)	4.8 (1.8—9.7)	4.2 (1.4—9.0)
SVAC	Male	Indonesia	719 (223—1,550)	1,850 (504—4,410)	1.8 (0.5—4.1)	1.9 (0.5—4.5)
SVAC	Male	Lao People's Democratic Republic	51.0 (17.8—110)	115 (37.4—236)	5.6 (2.0—11.7)	5.1 (1.6—11.0)
SVAC	Male	Malaysia	112 (30.6—278)	258 (58.5—599)	2.7 (0.7—6.5)	2.0 (0.4—4.7)
SVAC	Male	Maldives	1.40 (0.428—2.91)	3.72 (0.949—8.50)	3.0 (0.9—6.2)	2.0 (0.5—5.0)
SVAC	Male	Mauritius	42.6 (14.6—90.0)	64.3 (18.2—148)	14.1 (5.0—29.5)	11.1 (3.2—25.4)
SVAC	Male	Myanmar	1,200 (446—2,660)	2,050 (750—4,080)	11.9 (4.5—26.4)	11.1 (4.0—22.1)
SVAC	Male	Philippines	575 (180—1,180)	1,470 (358—3,530)	3.9 (1.3—7.9)	4.2 (1.0—10.1)
SVAC	Male	Seychelles	1.22 (0.368—2.83)	2.19 (0.597—4.66)	5.8 (1.8—13.3)	4.6 (1.2—9.8)
SVAC	Male	Sri Lanka	1,080 (347—2,190)	837 (237—1,720)	20.8 (7.1—41.9)	9.3 (2.7—19.1)

Risk Factor	Sex	Location	Number of Deaths		Rate of Deaths (per 100,000)	
			1990	2023	1990	2023
SVAC	Male	Thailand	1,100 (390—2,320)	2,240 (607—4,650)	6.1 (2.1—13.2)	7.9 (2.1—16.1)
SVAC	Male	Timor-Leste	5.73 (1.93—13.4)	14.6 (3.97—33.6)	3.4 (1.2—7.5)	3.6 (1.0—8.7)
SVAC	Male	Viet Nam	759 (250—1,720)	2,100 (597—4,350)	4.7 (1.6—10.4)	5.4 (1.5—11.3)
SVAC	Male	Sub-Saharan Africa	13,900 (4,200—30,200)	36,700 (7,400—75,700)	13.1 (4.1—29.3)	14.1 (2.8—29.9)
SVAC	Male	Central Sub-Saharan Africa	1,440 (543—3,450)	3,540 (1,010—7,750)	12.7 (4.7—30.5)	11.5 (3.3—24.8)
SVAC	Male	Angola	227 (70.4—534)	950 (247—2,050)	10.7 (3.2—25.2)	13.4 (3.4—29.8)
SVAC	Male	Central African Republic	103 (28.8—246)	220 (39.9—534)	17.5 (5.0—42.6)	16.2 (3.1—38.2)
SVAC	Male	Congo	95.5 (24.2—236)	235 (38.7—598)	18.6 (4.9—46.7)	15.3 (3.0—37.9)
SVAC	Male	Democratic Republic of the Congo	977 (358—2,330)	1,970 (523—4,620)	12.5 (4.5—30.1)	9.9 (2.5—23.5)
SVAC	Male	Equatorial Guinea	12.0 (3.84—28.3)	93.1 (9.89—243)	14.4 (4.5—33.3)	24.8 (3.3—62.0)
SVAC	Male	Gabon	27.9 (8.51—61.0)	79.3 (18.4—169)	12.3 (3.7—26.9)	15.3 (3.5—32.2)
SVAC	Male	Eastern Sub-Saharan Africa	6,910 (1,200—15,100)	13,100 (2,860—27,200)	17.5 (3.4—38.7)	13.6 (2.7—28.7)
SVAC	Male	Burundi	211 (45.2—595)	278 (75.1—602)	17.7 (3.9—48.8)	9.1 (2.6—20.3)
SVAC	Male	Comoros	4.77 (1.27—11.2)	9.43 (2.27—24.5)	4.9 (1.3—11.2)	4.0 (0.9—10.6)
SVAC	Male	Djibouti	5.55 (1.68—12.6)	39.7 (9.38—84.3)	6.4 (2.0—14.1)	9.4 (2.2—19.7)
SVAC	Male	Eritrea	77.8 (22.6—172)	198 (55.4—465)	11.0 (3.1—23.6)	10.5 (2.8—24.3)
SVAC	Male	Ethiopia	755 (248—1,670)	1,440 (516—2,740)	7.0 (2.2—16.1)	5.6 (1.9—10.8)
SVAC	Male	Kenya	641 (176—1,400)	2,200 (375—4,630)	15.0 (4.6—33.6)	18.0 (3.3—37.3)
SVAC	Male	Madagascar	240 (72.9—542)	567 (149—1,370)	9.6 (3.0—21.4)	8.7 (2.3—21.8)
SVAC	Male	Malawi	529 (91.3—1,140)	1,140 (151—2,620)	26.3 (4.9—58.6)	28.5 (3.1—62.2)
SVAC	Male	Mozambique	181 (62.1—400)	1,480 (-34.1—4,150)	6.5 (2.2—14.1)	23.1 (-0.0—65.4)
SVAC	Male	Rwanda	279 (92.7—618)	414 (142—808)	20.2 (6.6—45.0)	13.8 (4.3—27.4)
SVAC	Male	Somalia	96.2 (27.6—244)	278 (73.7—647)	6.3 (1.7—15.8)	6.4 (1.7—14.7)
SVAC	Male	South Sudan	117 (35.6—280)	238 (58.3—528)	8.9 (2.6—20.9)	10.6 (2.6—23.9)
SVAC	Male	Uganda	2,150 (-463—6,180)	1,790 (255—3,730)	63.3 (-13.3—183.2)	21.6 (3.1—46.1)
SVAC	Male	United Republic of Tanzania	1,170 (138—2,680)	2,000 (275—4,560)	22.1 (3.1—50.9)	14.6 (1.7—33.3)
SVAC	Male	Zambia	450 (44.6—1,130)	1,040 (123—2,280)	28.3 (3.2—72.9)	24.9 (2.9—55.4)
SVAC	Male	Southern Sub-Saharan Africa	1,590 (581—3,230)	7,990 (102—19,700)	12.3 (4.5—24.7)	28.9 (0.3—71.4)
SVAC	Male	Botswana	74.0 (19.0—150)	282 (-20.5—658)	27.3 (7.0—56.0)	42.3 (-3.5—98.7)
SVAC	Male	Eswatini	14.9 (3.81—33.6)	88.9 (-0.647—223)	10.3 (2.8—22.7)	29.7 (-1.5—74.6)
SVAC	Male	Lesotho	36.6 (11.5—83.5)	184 (5.61—428)	12.2 (3.8—28.0)	35.4 (0.1—85.6)
SVAC	Male	Namibia	35.6 (11.7—75.7)	166 (19.1—386)	11.1 (3.7—24.6)	20.4 (2.1—50.6)
SVAC	Male	South Africa	1,180 (359—2,440)	6,920 (157—17,200)	12.0 (3.8—24.6)	31.8 (0.8—78.8)
SVAC	Male	Zimbabwe	251 (-12.8—728)	351 (-15.5—1,020)	12.2 (-0.5—36.8)	10.2 (-0.4—30.5)
SVAC	Male	Western Sub-Saharan Africa	4,010 (1,560—8,000)	12,000 (2,600—25,300)	9.7 (3.9—20.0)	11.6 (2.7—24.7)
SVAC	Male	Benin	69.4 (22.8—135)	263 (75.1—571)	8.2 (2.7—15.9)	9.8 (2.8—22.6)
SVAC	Male	Burkina Faso	302 (8.86—885)	272 (63.5—645)	17.0 (1.1—48.9)	6.3 (1.5—15.2)
SVAC	Male	Cabo Verde	6.90 (2.01—15.2)	16.2 (4.09—32.8)	9.8 (3.0—21.7)	9.7 (2.4—20.1)
SVAC	Male	Cameroon	239 (88.2—507)	1,280 (223—2,830)	11.5 (4.2—25.0)	18.0 (3.3—39.0)
SVAC	Male	Chad	132 (45.1—274)	433 (114—950)	11.2 (3.8—23.4)	13.2 (3.4—28.3)
SVAC	Male	Côte d'Ivoire	831 (0.746—1,970)	1,630 (318—3,390)	30.1 (2.0—70.0)	19.3 (3.8—38.3)
SVAC	Male	Gambia	12.4 (4.11—26.1)	72.6 (10.0—176)	6.3 (2.1—13.5)	13.0 (2.0—31.8)
SVAC	Male	Ghana	272 (94.0—574)	980 (272—1,930)	9.2 (2.9—19.7)	12.8 (3.3—25.9)
SVAC	Male	Guinea	95.5 (32.4—211)	304 (68.2—650)	7.2 (2.4—16.1)	10.6 (2.3—23.1)
SVAC	Male	Guinea-Bissau	19.3 (6.99—39.8)	69.5 (11.3—160)	9.9 (3.5—20.4)	16.1 (2.8—36.6)
SVAC	Male	Liberia	49.6 (18.5—102)	166 (41.8—360)	8.5 (3.1—18.0)	10.9 (2.7—23.1)
SVAC	Male	Mali	120 (40.1—261)	399 (100—849)	6.9 (2.2—15.3)	8.2 (2.1—17.3)
SVAC	Male	Mauritania	33.8 (11.3—69.7)	90.1 (21.3—208)	8.0 (2.5—16.6)	9.0 (2.1—20.8)
SVAC	Male	Niger	85.3 (28.3—187)	290 (76.8—666)	6.4 (2.0—14.1)	6.9 (1.8—16.4)

Risk Factor	Sex	Location	Number of Deaths		Rate of Deaths (per 100,000)	
			1990	2023	1990	2023
SVAC	Male	Nigeria	1,490 (636—2,830)	4,960 (839—11,000)	7.5 (3.1—14.7)	10.8 (2.2—23.6)
SVAC	Male	Sao Tome and Principe	0.656 (0.214—1.60)	1.82 (0.433—4.27)	2.7 (0.8—6.6)	3.2 (0.8—7.3)
SVAC	Male	Senegal	105 (32.7—218)	314 (86.1—701)	7.2 (2.2—15.2)	7.9 (2.2—17.9)
SVAC	Male	Sierra Leone	84.1 (28.0—186)	223 (62.8—474)	9.0 (2.9—19.8)	11.9 (3.2—25.6)
SVAC	Male	Togo	55.4 (19.4—112)	225 (46.3—469)	8.9 (3.1—18.8)	11.7 (2.5—25.3)

Table S2: Age-standardised numbers and rates of DALYs attributable to IPV and SVAC by location and sex.

Risk Factor	Sex	Location	Number of DALYs (thousands)		Rate of DALYs (per 100,000)	
			1990	2023	1990	2023
IPV	Female	Global	12,800 (3,900—23,900)	18,500 (8,740—30,000)	686.5 (209.7—1273.4)	624.4 (294.7—1015.6)
IPV	Female	Central Europe, Eastern Europe, and Central Asia	612 (255—1,200)	568 (279—974)	369.7 (156.0—713.6)	321.3 (158.0—542.1)
IPV	Female	Central Asia	59.9 (28.0—116)	90.1 (44.4—165)	255.7 (119.3—488.9)	247.0 (120.2—462.4)
IPV	Female	Armenia	2.05 (0.907—4.17)	2.15 (0.809—4.67)	164.3 (71.7—333.6)	164.1 (62.4—347.5)
IPV	Female	Azerbaijan	6.56 (3.34—11.3)	7.53 (3.47—13.7)	242.6 (121.8—423.6)	175.3 (82.8—323.8)
IPV	Female	Georgia	2.83 (1.24—5.58)	1.90 (0.869—3.51)	128.9 (57.3—247.3)	135.8 (61.4—254.9)
IPV	Female	Kazakhstan	17.9 (7.97—36.5)	19.9 (8.62—35.4)	293.0 (130.8—600.7)	266.5 (114.0—482.4)
IPV	Female	Kyrgyzstan	5.52 (2.23—10.7)	7.66 (3.91—13.4)	384.2 (154.0—735.1)	296.9 (150.5—520.4)
IPV	Female	Mongolia	3.39 (1.32—6.19)	4.99 (2.29—8.41)	522.3 (210.1—922.8)	399.2 (182.3—674.3)
IPV	Female	Tajikistan	4.76 (1.94—9.06)	12.1 (5.66—20.9)	301.7 (127.5—555.6)	343.7 (160.6—594.9)
IPV	Female	Turkmenistan	2.93 (1.12—6.70)	5.26 (2.18—9.79)	248.9 (99.1—557.3)	271.2 (112.1—507.3)
IPV	Female	Uzbekistan	14.0 (5.61—30.1)	28.7 (8.20—75.7)	221.0 (86.5—466.1)	222.0 (62.8—592.3)
IPV	Female	Central Europe	167 (57.7—322)	130 (45.5—237)	338.4 (119.2—649.4)	269.2 (95.9—486.1)
IPV	Female	Albania	3.02 (1.43—5.71)	3.08 (1.01—6.42)	270.3 (122.6—517.5)	309.4 (103.8—637.2)
IPV	Female	Bosnia and Herzegovina	5.27 (1.90—10.3)	3.07 (1.05—6.01)	300.7 (109.1—594.6)	209.8 (74.6—413.0)
IPV	Female	Bulgaria	11.0 (2.24—24.2)	8.56 (2.79—16.9)	315.3 (65.9—691.8)	300.4 (99.0—561.5)
IPV	Female	Croatia	7.02 (2.42—15.3)	4.51 (1.54—8.82)	346.4 (126.4—735.6)	279.4 (96.9—538.9)
IPV	Female	Czechia	22.9 (8.93—37.6)	15.0 (6.56—26.8)	567.2 (226.2—911.1)	348.7 (152.7—623.5)
IPV	Female	Hungary	22.0 (4.88—48.6)	14.7 (5.21—26.6)	515.2 (124.5—1116.4)	357.8 (130.3—607.9)
IPV	Female	Montenegro	0.853 (0.249—1.69)	1.08 (0.289—2.29)	363.9 (107.2—725.3)	392.1 (106.0—832.2)
IPV	Female	North Macedonia	2.05 (0.693—4.30)	2.30 (0.549—5.49)	275.8 (93.2—577.5)	305.9 (74.8—728.4)
IPV	Female	Poland	37.6 (11.2—77.1)	31.7 (9.77—65.7)	245.8 (74.6—503.6)	194.0 (61.8—396.9)
IPV	Female	Romania	29.7 (12.0—53.5)	22.9 (7.82—44.9)	332.9 (136.9—598.9)	291.4 (98.9—567.4)
IPV	Female	Serbia	12.5 (3.76—26.9)	11.8 (3.57—24.3)	334.9 (103.6—723.8)	293.5 (92.3—607.0)
IPV	Female	Slovakia	7.12 (3.12—12.7)	7.48 (2.75—13.6)	354.8 (156.2—626.9)	335.2 (120.3—604.8)
IPV	Female	Slovenia	2.87 (0.852—6.70)	2.34 (0.718—4.56)	358.7 (108.4—823.5)	276.7 (88.9—544.0)
IPV	Female	Eastern Europe	386 (166—746)	347 (180—605)	413.8 (181.8—783.5)	376.6 (193.8—633.3)
IPV	Female	Belarus	10.7 (3.91—23.6)	10.1 (3.36—21.8)	253.5 (93.1—552.6)	239.6 (78.6—507.8)
IPV	Female	Estonia	2.35 (0.739—4.99)	1.59 (0.689—2.92)	367.0 (116.9—768.9)	297.0 (128.1—550.5)
IPV	Female	Latvia	5.42 (1.74—10.6)	3.28 (1.42—5.59)	476.6 (155.8—919.6)	417.6 (183.0—702.1)
IPV	Female	Lithuania	7.47 (2.23—13.9)	5.23 (2.41—8.95)	501.4 (148.8—921.1)	436.8 (198.4—770.8)
IPV	Female	Republic of Moldova	6.75 (2.84—12.2)	7.10 (2.73—12.1)	385.9 (162.9—698.0)	425.8 (167.0—717.7)
IPV	Female	Russian Federation	272 (116—564)	245 (124—423)	438.0 (193.9—891.3)	372.5 (191.2—617.3)
IPV	Female	Ukraine	80.9 (37.5—145)	75.1 (36.4—129)	371.7 (174.7—650.9)	412.1 (200.5—728.4)
IPV	Female	High-income	1,870 (744—3,150)	3,000 (1,270—5,200)	526.1 (212.2—873.6)	734.8 (316.2—1262.6)
IPV	Female	Australasia	36.0 (16.5—59.2)	87.8 (29.4—165)	459.8 (211.3—758.1)	743.8 (246.3—1404.2)
IPV	Female	Australia	28.3 (13.3—46.0)	72.7 (23.4—138)	434.4 (203.0—706.5)	732.5 (231.8—1399.4)
IPV	Female	New Zealand	7.69 (2.64—14.7)	15.2 (4.64—28.2)	583.9 (200.6—1117.6)	803.1 (248.5—1471.3)
IPV	Female	High-income Asia Pacific	227 (58.3—486)	261 (64.5—515)	329.8 (86.4—705.5)	382.1 (88.2—752.2)
IPV	Female	Brunei Darussalam	0.183 (0.0578—0.405)	0.442 (0.123—0.967)	215.8 (67.2—479.5)	246.4 (68.6—543.2)
IPV	Female	Japan	164 (35.3—347)	174 (44.8—337)	325.7 (72.1—674.2)	383.9 (89.8—737.0)
IPV	Female	Republic of Korea	60.0 (19.2—121)	84.7 (19.2—176)	344.1 (112.1—695.0)	411.5 (88.8—842.5)
IPV	Female	Singapore	2.02 (0.498—5.93)	2.37 (0.826—5.17)	156.9 (40.0—456.0)	102.7 (36.5—231.1)
IPV	Female	High-income North America	856 (375—1,390)	1,610 (669—2,770)	774.6 (342.1—1255.8)	1158.4 (489.5—1959.7)
IPV	Female	Canada	51.9 (20.9—89.6)	46.9 (15.8—96.2)	471.0 (191.3—817.6)	333.0 (111.8—688.9)
IPV	Female	Greenland	0.426 (0.0759—0.879)	0.299 (0.0593—0.690)	2065.7 (410.0—4321.6)	1497.0 (295.0—3550.0)
IPV	Female	United States of America	804 (354—1,300)	1,560 (648—2,680)	807.7 (359.0—1304.8)	1253.6 (530.4—2084.4)

Risk Factor	Sex	Location	Number of DALYs (thousands)		Rate of DALYs (per 100,000)	
			1990	2023	1990	2023
IPV	Female	Southern Latin America	110 (40.8—193)	206 (82.5—365)	615.9 (226.9—1082.6)	738.9 (294.8—1306.5)
IPV	Female	Argentina	67.9 (23.7—127)	148 (58.7—259)	580.9 (202.2—1087.7)	786.6 (310.8—1366.6)
IPV	Female	Chile	36.7 (14.9—61.1)	48.9 (18.7—90.1)	736.4 (295.4—1237.1)	633.0 (243.4—1155.6)
IPV	Female	Uruguay	5.13 (1.78—10.5)	8.61 (2.88—16.6)	446.5 (156.6—907.8)	653.8 (221.2—1276.9)
IPV	Female	Western Europe	646 (230—1,210)	837 (316—1,470)	422.8 (153.8—783.6)	504.8 (190.4—897.9)
IPV	Female	Andorra	0.0719 (0.0183—0.164)	0.150 (0.0372—0.340)	329.5 (85.1—758.6)	425.6 (104.4—994.9)
IPV	Female	Austria	10.1 (3.05—22.9)	10.4 (3.49—20.2)	319.0 (97.8—709.6)	300.1 (106.3—577.4)
IPV	Female	Belgium	16.0 (4.93—34.8)	20.8 (7.89—37.9)	404.9 (126.3—864.4)	467.0 (177.3—862.2)
IPV	Female	Cyprus	0.816 (0.291—1.72)	1.88 (0.467—3.98)	282.1 (101.2—593.1)	325.2 (79.5—677.9)
IPV	Female	Denmark	11.2 (3.09—21.9)	12.0 (4.68—21.0)	539.6 (154.5—1021.9)	545.8 (206.5—958.6)
IPV	Female	Finland	10.0 (2.76—18.3)	9.98 (4.03—16.9)	497.8 (144.9—901.5)	501.9 (202.7—831.3)
IPV	Female	France	98.7 (28.8—212)	124 (44.2—237)	431.3 (127.9—911.3)	464.5 (167.7—874.0)
IPV	Female	Germany	174 (51.7—319)	134 (54.2—251)	532.5 (165.2—959.5)	438.6 (167.3—854.3)
IPV	Female	Greece	9.71 (3.23—21.7)	17.5 (6.34—34.8)	235.0 (78.6—517.5)	427.7 (153.6—827.9)
IPV	Female	Iceland	0.459 (0.151—0.892)	0.891 (0.363—1.55)	490.3 (162.5—952.2)	622.3 (252.3—1088.5)
IPV	Female	Ireland	3.43 (1.21—6.78)	6.60 (2.22—13.6)	269.3 (94.7—528.7)	321.4 (107.2—659.3)
IPV	Female	Israel	4.51 (1.90—7.87)	10.7 (3.66—22.3)	260.7 (109.6—453.0)	305.7 (104.5—631.5)
IPV	Female	Italy	70.7 (24.9—145)	76.6 (20.5—160)	311.3 (110.4—637.8)	358.4 (90.2—739.5)
IPV	Female	Luxembourg	0.698 (0.225—1.46)	1.09 (0.465—1.86)	444.7 (147.7—925.3)	410.6 (173.0—707.5)
IPV	Female	Malta	0.426 (0.152—0.847)	0.751 (0.171—1.74)	299.7 (105.1—595.9)	354.2 (77.9—836.9)
IPV	Female	Monaco	0.0498 (0.00965—0.116)	0.0662 (0.0162—0.154)	394.6 (77.4—910.4)	481.2 (115.5—1075.9)
IPV	Female	Netherlands	33.2 (13.9—58.1)	52.0 (20.3—94.6)	553.3 (227.6—963.1)	787.5 (302.6—1428.0)
IPV	Female	Norway	7.02 (2.03—14.3)	11.2 (4.01—20.3)	435.9 (127.5—881.2)	566.0 (203.7—1005.4)
IPV	Female	Portugal	19.4 (6.56—39.7)	23.5 (7.86—45.1)	486.8 (166.8—982.6)	554.7 (170.4—1020.5)
IPV	Female	San Marino	0.0256 (0.00783—0.0595)	0.0554 (0.0136—0.130)	303.0 (93.3—695.8)	418.2 (98.5—976.8)
IPV	Female	Spain	35.2 (14.4—69.7)	85.7 (31.8—160)	235.5 (97.6—464.8)	478.9 (167.1—879.9)
IPV	Female	Sweden	15.1 (4.59—32.0)	24.0 (11.0—40.8)	455.2 (141.8—949.9)	637.6 (278.1—1097.4)
IPV	Female	Switzerland	10.9 (4.27—23.7)	12.0 (4.35—25.7)	390.5 (157.4—835.2)	346.7 (119.2—747.0)
IPV	Female	United Kingdom	115 (42.2—207)	201 (81.1—354)	510.6 (189.9—909.5)	765.8 (314.8—1313.5)
IPV	Female	Latin America and Caribbean	687 (364—1,100)	1,390 (553—2,380)	519.1 (259.7—854.5)	592.8 (238.3—1018.6)
IPV	Female	Andean Latin America	83.2 (37.6—141)	194 (79.6—341)	681.1 (296.2—1158.3)	779.7 (320.0—1367.3)
IPV	Female	Bolivia (Plurinational State of)	19.5 (8.87—31.2)	46.7 (17.6—76.8)	965.0 (432.5—1531.5)	1025.5 (387.2—1693.9)
IPV	Female	Ecuador	18.7 (9.16—31.5)	61.2 (27.3—103)	576.6 (261.6—981.9)	890.8 (396.9—1493.5)
IPV	Female	Peru	45.1 (17.8—79.2)	86.4 (31.9—161)	649.3 (248.3—1163.1)	635.1 (235.5—1184.1)
IPV	Female	Caribbean	101 (51.0—173)	155 (92.3—224)	786.9 (387.9—1359.9)	850.7 (512.2—1223.1)
IPV	Female	Antigua and Barbuda	0.0832 (0.0313—0.162)	0.196 (0.0603—0.400)	373.4 (134.0—738.9)	516.5 (164.0—1042.4)
IPV	Female	Bahamas	0.571 (0.306—0.998)	1.44 (0.712—2.57)	582.8 (292.8—1047.2)	835.4 (418.9—1483.7)
IPV	Female	Barbados	0.494 (0.246—0.902)	0.750 (0.306—1.45)	495.6 (244.7—901.5)	630.9 (285.9—1180.1)
IPV	Female	Belize	0.218 (0.116—0.397)	0.916 (0.431—1.75)	397.5 (197.3—738.4)	580.3 (270.2—1111.9)
IPV	Female	Bermuda	0.112 (0.0456—0.196)	0.137 (0.0483—0.289)	436.8 (177.4—764.5)	565.7 (211.3—1151.5)
IPV	Female	Cuba	21.6 (8.97—47.2)	14.9 (6.69—29.1)	501.4 (205.1—1118.2)	333.8 (164.3—642.6)
IPV	Female	Dominica	0.0958 (0.0366—0.187)	0.143 (0.0504—0.286)	398.6 (152.1—769.3)	553.3 (196.2—1099.8)
IPV	Female	Dominican Republic	15.0 (7.70—25.8)	28.9 (13.4—47.6)	601.6 (304.1—1044.9)	679.3 (317.8—1122.4)
IPV	Female	Grenada	0.126 (0.0498—0.240)	0.219 (0.0870—0.426)	405.2 (155.3—769.2)	515.1 (211.5—991.3)
IPV	Female	Guyana	2.59 (0.770—4.53)	3.41 (1.49—5.63)	981.6 (264.4—1708.1)	1096.5 (481.0—1807.5)
IPV	Female	Haiti	41.3 (19.0—71.1)	72.8 (44.2—110)	1979.8 (905.7—3416.5)	1445.3 (864.1—2161.7)
IPV	Female	Jamaica	3.55 (2.08—5.68)	9.68 (5.96—14.0)	449.0 (260.0—713.5)	847.1 (522.6—1229.5)
IPV	Female	Puerto Rico	7.65 (4.03—12.2)	8.88 (3.72—16.3)	555.7 (292.6—882.7)	688.5 (294.6—1268.7)
IPV	Female	Saint Kitts and Nevis	0.0594 (0.0256—0.115)	0.116 (0.0414—0.231)	429.3 (181.4—830.4)	563.6 (210.9—1120.8)
IPV	Female	Saint Lucia	0.229 (0.112—0.414)	0.411 (0.151—0.810)	506.0 (240.8—932.0)	573.0 (218.1—1121.2)

Risk Factor	Sex	Location	Number of DALYs (thousands)		Rate of DALYs (per 100,000)	
			1990	2023	1990	2023
IPV	Female	Saint Vincent and the Grenadines	0.199 (0.104—0.341)	0.268 (0.114—0.510)	561.7 (287.6—977.4)	628.4 (275.6—1176.6)
IPV	Female	Suriname	0.916 (0.313—1.78)	1.95 (0.706—3.33)	691.0 (216.4—1347.2)	864.8 (314.2—1482.9)
IPV	Female	Trinidad and Tobago	2.62 (1.24—4.62)	4.07 (2.23—6.48)	624.5 (288.3—1105.5)	753.5 (417.8—1184.0)
IPV	Female	United States Virgin Islands	0.282 (0.155—0.446)	0.237 (0.112—0.393)	707.0 (388.6—1124.8)	807.4 (394.5—1297.0)
IPV	Female	Central Latin America	201 (106—319)	511 (207—912)	377.3 (191.1—603.7)	499.2 (203.7—891.5)
IPV	Female	Colombia	49.3 (21.0—80.3)	107 (37.8—201)	437.3 (179.2—721.0)	510.5 (180.4—963.4)
IPV	Female	Costa Rica	4.95 (2.61—8.51)	13.2 (5.27—23.4)	491.7 (249.9—848.7)	633.7 (250.6—1116.1)
IPV	Female	El Salvador	7.69 (2.92—15.3)	12.6 (4.88—22.1)	457.1 (187.5—841.5)	486.4 (190.2—851.3)
IPV	Female	Guatemala	10.0 (4.49—19.3)	29.2 (12.0—51.4)	433.1 (190.7—853.5)	480.5 (195.2—846.2)
IPV	Female	Honduras	5.17 (2.99—8.57)	20.2 (9.38—33.9)	378.5 (223.6—653.0)	509.2 (235.0—853.2)
IPV	Female	Mexico	99.9 (51.3—155)	264 (110—473)	363.9 (182.1—567.6)	504.2 (211.0—906.8)
IPV	Female	Nicaragua	4.78 (2.40—8.19)	13.3 (4.94—23.8)	412.2 (196.3—698.2)	513.6 (195.3—919.7)
IPV	Female	Panama	1.98 (0.891—3.71)	6.43 (3.04—11.2)	242.1 (103.6—460.5)	413.2 (195.2—724.6)
IPV	Female	Venezuela (Bolivarian Republic of)	17.3 (6.25—33.2)	45.7 (20.4—81.1)	277.9 (97.7—537.5)	451.0 (207.9—803.2)
IPV	Female	Tropical Latin America	302 (151—498)	527 (181—1,020)	562.6 (277.8—953.0)	594.1 (224.2—1129.0)
IPV	Female	Brazil	297 (149—490)	513 (177—1,000)	567.3 (280.2—958.6)	594.1 (222.9—1135.9)
IPV	Female	Paraguay	4.47 (2.05—7.86)	14.2 (5.80—26.1)	360.6 (156.8—656.0)	595.1 (242.7—1095.2)
IPV	Female	North Africa and Middle East	502 (232—943)	1,230 (590—2,010)	491.8 (228.4—906.0)	549.7 (262.4—909.3)
IPV	Female	Afghanistan	17.5 (8.51—29.9)	73.4 (36.3—112)	595.9 (295.0—992.6)	729.8 (370.4—1134.1)
IPV	Female	Algeria	32.8 (12.4—64.9)	68.4 (26.3—133)	423.4 (162.6—830.7)	414.2 (157.8—808.7)
IPV	Female	Bahrain	0.596 (0.197—1.25)	1.91 (0.661—3.76)	394.9 (129.4—829.4)	408.1 (140.5—817.8)
IPV	Female	Egypt	65.8 (25.5—124)	171 (66.0—311)	388.3 (149.2—732.0)	472.7 (181.8—855.2)
IPV	Female	Iran (Islamic Republic of)	98.8 (34.9—209)	231 (71.5—466)	589.2 (209.9—1245.9)	668.3 (210.4—1352.5)
IPV	Female	Iraq	24.1 (12.7—45.2)	75.7 (47.0—115)	452.6 (235.7—842.9)	496.9 (312.1—744.5)
IPV	Female	Jordan	3.81 (1.89—6.51)	15.4 (7.49—26.4)	359.4 (187.6—606.0)	346.5 (172.1—586.6)
IPV	Female	Kuwait	1.60 (0.603—3.18)	5.57 (1.97—10.9)	310.0 (116.4—620.5)	380.1 (133.7—756.8)
IPV	Female	Lebanon	10.2 (7.05—14.5)	5.39 (1.95—12.2)	903.8 (626.9—1290.0)	244.3 (88.4—547.7)
IPV	Female	Libya	4.92 (2.12—9.85)	13.9 (5.20—26.6)	421.9 (175.2—838.5)	487.8 (181.0—943.6)
IPV	Female	Morocco	30.0 (10.5—71.3)	55.3 (26.3—93.8)	355.3 (125.2—821.9)	389.4 (185.5—659.5)
IPV	Female	Oman	1.30 (0.388—2.81)	5.14 (1.57—10.5)	300.8 (88.4—640.1)	357.1 (106.1—743.6)
IPV	Female	Palestine	2.86 (1.14—5.25)	8.98 (3.92—15.2)	511.3 (203.8—922.1)	511.5 (224.1—860.2)
IPV	Female	Qatar	0.288 (0.105—0.581)	2.59 (0.885—4.97)	292.1 (102.7—602.6)	315.2 (105.9—631.0)
IPV	Female	Saudi Arabia	16.2 (6.54—32.9)	43.5 (14.0—89.5)	398.9 (157.1—795.7)	453.6 (144.4—946.9)
IPV	Female	Sudan	41.7 (13.5—82.9)	111 (54.9—184)	688.7 (229.3—1327.5)	900.0 (443.8—1496.6)
IPV	Female	Syrian Arab Republic	15.9 (5.57—33.8)	41.1 (14.8—82.9)	459.3 (154.3—953.2)	613.5 (218.8—1238.5)
IPV	Female	Tunisia	9.24 (3.78—19.8)	18.3 (5.74—36.2)	341.3 (137.8—727.9)	382.5 (121.0—756.2)
IPV	Female	Türkiye	108 (54.2—188)	218 (109—365)	574.2 (278.1—982.5)	650.3 (325.3—1097.4)
IPV	Female	United Arab Emirates	1.56 (0.565—3.28)	9.17 (3.17—17.8)	367.5 (129.4—772.3)	390.5 (134.1—772.0)
IPV	Female	Yemen	14.6 (5.20—30.3)	50.6 (19.7—97.7)	416.3 (148.4—847.3)	454.9 (176.7—871.3)
IPV	Female	South Asia	2,490 (434—4,790)	4,720 (1,580—8,300)	700.7 (146.5—1367.1)	674.3 (231.0—1200.2)
IPV	Female	Bangladesh	414 (85.5—745)	456 (216—780)	1234.5 (286.1—2116.0)	685.6 (331.5—1167.1)
IPV	Female	Bhutan	0.508 (0.181—1.09)	0.893 (0.340—1.67)	312.3 (104.7—668.6)	312.4 (116.9—595.6)
IPV	Female	India	1,890 (197—3,830)	3,800 (1,090—6,940)	671.8 (91.2—1352.6)	696.7 (208.0—1266.9)
IPV	Female	Nepal	51.4 (0.174—120)	69.5 (14.7—137)	778.1 (27.8—1754.1)	571.4 (131.4—1132.7)
IPV	Female	Pakistan	127 (62.3—215)	395 (214—639)	410.0 (203.7—684.9)	519.8 (274.8—867.4)
IPV	Female	Southeast Asia, East Asia, and Oceania	4,520 (582—8,740)	3,160 (1,570—5,050)	743.5 (88.1—1440.7)	354.7 (178.7—561.4)
IPV	Female	East Asia	4,050 (381—7,930)	2,430 (1,160—3,970)	903.5 (69.6—1772.7)	399.4 (192.6—649.8)
IPV	Female	China	3,980 (351—7,840)	2,320 (1,100—3,860)	919.9 (63.7—1814.6)	395.4 (191.0—652.1)

Risk Factor	Sex	Location	Number of DALYs (thousands)		Rate of DALYs (per 100,000)	
			1990	2023	1990	2023
IPV	Female	Democratic People's Republic of Korea	38.6 (15.2—70.5)	57.0 (23.9—103)	491.2 (189.9—902.0)	510.0 (219.5—925.1)
IPV	Female	Taiwan	31.1 (14.2—55.0)	47.4 (14.9—91.9)	415.9 (185.8—738.7)	483.0 (159.1—905.1)
IPV	Female	Oceania	12.2 (5.28—19.8)	43.9 (23.2—65.7)	581.5 (254.6—946.9)	908.5 (480.3—1375.3)
IPV	Female	American Samoa	0.0582 (0.0280—0.101)	0.0854 (0.0345—0.148)	363.6 (171.3—627.0)	501.5 (203.2—866.5)
IPV	Female	Cook Islands	0.0359 (0.00707—0.0792)	0.0259 (0.00900—0.0515)	554.4 (114.3—1179.9)	485.7 (157.7—990.7)
IPV	Female	Fiji	1.35 (0.345—2.41)	2.14 (0.797—3.80)	518.0 (146.3—915.8)	642.6 (237.8—1137.4)
IPV	Female	Guam	0.218 (0.0626—0.400)	0.314 (0.123—0.554)	462.5 (135.3—850.2)	542.1 (214.6—952.6)
IPV	Female	Kiribati	0.174 (0.0382—0.299)	0.377 (0.160—0.619)	689.9 (169.9—1168.0)	825.6 (369.5—1334.6)
IPV	Female	Marshall Islands	0.0687 (0.0246—0.130)	0.0920 (0.0354—0.161)	556.7 (204.6—1009.6)	646.3 (245.4—1129.8)
IPV	Female	Micronesia (Federated States of)	0.122 (0.0558—0.228)	0.165 (0.0737—0.289)	397.3 (184.4—728.3)	446.8 (198.0—776.3)
IPV	Female	Nauru	0.0248 (0.00600—0.0478)	0.0343 (0.0138—0.0608)	766.1 (208.0—1461.1)	807.0 (322.4—1429.4)
IPV	Female	Niue	0.00364 (0.00116—0.00688)	0.00348 (0.00139—0.00618)	495.6 (155.2—927.6)	557.6 (231.2—993.0)
IPV	Female	Northern Mariana Islands	0.112 (0.0445—0.203)	0.0980 (0.0405—0.169)	617.1 (248.9—1090.7)	569.4 (233.0—1001.8)
IPV	Female	Palau	0.0365 (0.0153—0.0759)	0.0404 (0.0175—0.0765)	643.5 (277.8—1310.4)	651.4 (281.0—1239.3)
IPV	Female	Papua New Guinea	7.88 (3.75—12.7)	35.3 (19.3—52.6)	601.3 (296.2—959.9)	988.3 (538.6—1472.7)
IPV	Female	Samoa	0.230 (0.0875—0.448)	0.376 (0.160—0.640)	460.1 (176.0—842.3)	554.7 (237.8—936.9)
IPV	Female	Solomon Islands	0.756 (0.206—1.34)	2.21 (0.776—3.61)	781.1 (224.8—1357.0)	774.4 (281.4—1261.8)
IPV	Female	Tokelau	0.00264 (0.000912—0.00501)	0.00321 (0.00138—0.00566)	512.1 (177.5—956.3)	551.1 (238.0—975.8)
IPV	Female	Tonga	0.139 (0.0709—0.229)	0.130 (0.0559—0.248)	460.9 (239.2—764.0)	351.5 (152.6—674.0)
IPV	Female	Tuvalu	0.0209 (0.00740—0.0383)	0.0313 (0.0110—0.0520)	606.1 (215.8—1113.2)	826.6 (308.6—1365.7)
IPV	Female	Vanuatu	0.211 (0.0526—0.385)	0.650 (0.227—1.05)	464.2 (126.0—855.5)	589.7 (211.7—970.4)
IPV	Female	Southeast Asia	462 (192—851)	683 (310—1,210)	285.5 (118.7—522.5)	246.9 (112.6—441.1)
IPV	Female	Cambodia	15.2 (7.14—26.6)	18.8 (8.23—31.9)	439.7 (200.8—768.3)	291.6 (128.4—501.2)
IPV	Female	Indonesia	124 (51.3—264)	231 (96.8—435)	195.3 (78.1—413.9)	206.3 (87.1—384.8)
IPV	Female	Lao People's Democratic Republic	6.27 (2.67—12.1)	7.62 (3.69—14.0)	471.6 (206.7—895.6)	274.5 (133.9—504.6)
IPV	Female	Malaysia	5.20 (2.73—10.2)	10.6 (5.73—18.5)	89.5 (45.9—181.5)	82.7 (45.0—144.5)
IPV	Female	Maldives	0.129 (0.0447—0.253)	0.262 (0.106—0.476)	215.1 (77.2—434.6)	152.0 (63.2—272.8)
IPV	Female	Mauritius	1.07 (0.284—2.76)	1.06 (0.484—2.03)	260.9 (70.4—668.7)	203.3 (93.8—388.6)
IPV	Female	Myanmar	51.3 (22.2—90.0)	76.6 (32.9—149)	351.1 (156.5—609.9)	345.7 (148.1—676.1)
IPV	Female	Philippines	63.0 (36.1—99.0)	84.4 (46.0—142)	296.9 (173.6—458.6)	205.5 (111.8—346.2)
IPV	Female	Seychelles	0.0674 (0.0330—0.109)	0.134 (0.0589—0.219)	268.4 (132.2—430.0)	373.4 (170.9—602.3)
IPV	Female	Sri Lanka	35.7 (-0.543—80.8)	23.8 (8.36—43.8)	555.5 (-7.0—1244.6)	262.4 (91.3—481.0)
IPV	Female	Thailand	77.5 (28.7—133)	94.5 (36.1—158)	356.6 (133.3—613.9)	334.0 (133.4—574.0)
IPV	Female	Timor-Leste	1.85 (0.394—3.67)	2.66 (1.07—4.45)	730.8 (176.4—1376.4)	566.7 (230.4—939.4)
IPV	Female	Viet Nam	80.1 (12.3—177)	131 (43.2—245)	356.4 (56.0—759.9)	322.9 (107.9—621.9)
IPV	Female	Sub-Saharan Africa	2,080 (795—3,550)	4,490 (2,080—7,410)	1448.0 (541.2—2526.2)	1252.5 (540.8—2160.8)
IPV	Female	Central Sub-Saharan Africa	285 (90.5—513)	609 (289—968)	1769.2 (541.1—3218.7)	1424.3 (635.5—2332.2)
IPV	Female	Angola	27.0 (10.6—54.4)	164 (67.4—312)	884.2 (331.1—1793.0)	1565.5 (611.5—2996.3)
IPV	Female	Central African Republic	17.6 (5.02—33.9)	40.2 (17.1—67.5)	2057.2 (559.5—3999.0)	2231.8 (860.8—3861.9)
IPV	Female	Congo	17.8 (4.03—37.4)	47.4 (14.6—95.3)	2566.3 (536.9—5509.1)	2573.8 (777.4—5145.2)
IPV	Female	Democratic Republic of the Congo	217 (67.6—402)	327 (179—504)	1949.8 (592.2—3644.0)	1165.6 (628.8—1802.1)
IPV	Female	Equatorial Guinea	1.74 (0.607—3.10)	16.8 (4.24—33.2)	1279.8 (446.1—2266.8)	3619.0 (892.8—6968.4)
IPV	Female	Gabon	3.63 (1.51—6.36)	13.7 (4.40—24.2)	1270.3 (489.0—2230.0)	2202.9 (686.9—3947.1)
IPV	Female	Eastern Sub-Saharan Africa	1,150 (368—2,020)	1,820 (832—3,060)	2133.4 (625.7—3817.9)	1405.6 (555.2—2458.8)
IPV	Female	Burundi	39.3 (7.56—101)	35.8 (14.1—59.2)	2452.1 (448.4—6361.4)	936.1 (373.6—1649.2)
IPV	Female	Comoros	0.463 (0.217—0.864)	0.885 (0.439—1.57)	320.4 (146.0—607.7)	304.0 (151.6—548.8)
IPV	Female	Djibouti	0.976 (0.380—1.90)	6.61 (2.34—12.9)	796.5 (310.3—1562.4)	1490.1 (526.0—2889.3)
IPV	Female	Eritrea	19.3 (6.82—40.3)	28.6 (12.8—55.2)	1807.9 (599.3—3766.5)	1305.7 (580.1—2578.9)
IPV	Female	Ethiopia	245 (101—486)	312 (178—480)	1685.3 (649.9—3292.4)	923.4 (494.7—1494.1)

Risk Factor	Sex	Location	Number of DALYs (thousands)		Rate of DALYs (per 100,000)	
			1990	2023	1990	2023
IPV	Female	Kenya	77.6 (32.2—136)	216 (96.6—368)	1261.5 (488.5—2240.1)	1332.6 (551.5—2303.8)
IPV	Female	Madagascar	42.0 (8.81—76.9)	108 (42.4—171)	1171.9 (230.9—2126.3)	1137.6 (429.6—1875.1)
IPV	Female	Malawi	59.7 (15.4—118)	125 (54.9—204)	2153.9 (501.2—4222.4)	2163.4 (859.9—3676.2)
IPV	Female	Mozambique	41.8 (20.9—69.2)	213 (65.1—388)	980.1 (472.7—1647.9)	2363.4 (665.0—4247.4)
IPV	Female	Rwanda	30.8 (11.4—59.3)	41.2 (17.2—73.2)	1409.6 (457.3—2848.2)	947.3 (366.6—1695.2)
IPV	Female	Somalia	14.7 (6.66—27.9)	41.9 (19.9—72.3)	645.1 (289.9—1271.7)	679.0 (327.3—1174.0)
IPV	Female	South Sudan	18.3 (6.67—35.6)	48.6 (23.2—88.5)	1081.1 (385.7—2122.7)	1566.5 (738.0—2864.4)
IPV	Female	Uganda	279 (66.8—512)	228 (90.9—373)	6368.8 (1310.9—11798.3)	1875.4 (683.7—3183.5)
IPV	Female	United Republic of Tanzania	166 (49.9—294)	247 (110—392)	2299.0 (619.6—4207.2)	1352.3 (544.7—2189.7)
IPV	Female	Zambia	111 (28.5—189)	168 (54.5—285)	4926.3 (1136.4—8664.2)	3072.9 (908.3—5327.0)
IPV	Female	Southern Sub-Saharan Africa	226 (129—348)	622 (270—1,070)	1268.9 (698.7—2009.5)	1858.9 (791.0—3240.7)
IPV	Female	Botswana	6.00 (1.38—11.1)	22.7 (7.43—40.5)	1486.0 (346.2—2738.0)	2665.0 (858.2—4682.3)
IPV	Female	Eswatini	6.48 (3.90—9.42)	25.8 (9.15—42.8)	2182.7 (1329.2—3185.6)	6472.8 (2117.4—10847.1)
IPV	Female	Lesotho	8.72 (4.03—13.7)	36.9 (11.1—61.1)	1600.2 (703.0—2552.9)	5778.7 (1624.2—9841.3)
IPV	Female	Namibia	6.51 (3.33—10.9)	17.2 (8.49—29.2)	1332.9 (701.5—2215.9)	1659.9 (776.8—2895.6)
IPV	Female	South Africa	118 (73.7—169)	400 (177—712)	894.6 (559.4—1302.1)	1563.8 (687.6—2801.3)
IPV	Female	Zimbabwe	80.2 (26.6—149)	119 (49.8—207)	2794.4 (891.3—5287.5)	2461.9 (982.0—4336.8)
IPV	Female	Western Sub-Saharan Africa	419 (194—741)	1,430 (682—2,280)	745.5 (338.4—1340.2)	925.0 (415.2—1541.8)
IPV	Female	Benin	8.30 (3.84—14.2)	27.5 (13.5—46.1)	580.6 (253.0—1014.3)	651.4 (309.5—1106.6)
IPV	Female	Burkina Faso	27.9 (11.5—64.2)	41.6 (21.6—69.2)	1039.5 (420.4—2412.7)	552.2 (275.1—922.3)
IPV	Female	Cabo Verde	0.485 (0.249—0.913)	0.780 (0.373—1.35)	456.7 (229.5—851.0)	422.8 (200.9—733.3)
IPV	Female	Cameroon	34.6 (13.3—60.7)	176 (65.2—312)	1148.4 (412.8—2018.4)	1895.7 (643.2—3497.4)
IPV	Female	Chad	18.6 (7.04—35.6)	70.7 (33.8—122)	1038.4 (382.9—1985.2)	1384.1 (638.9—2387.6)
IPV	Female	Côte d'Ivoire	47.1 (13.1—109)	96.5 (44.9—172)	1511.9 (361.7—3474.8)	987.6 (424.9—1816.1)
IPV	Female	Gambia	1.12 (0.339—2.42)	7.92 (3.15—13.6)	416.7 (129.3—879.7)	1068.3 (400.2—1838.6)
IPV	Female	Ghana	33.5 (14.8—56.4)	86.1 (43.7—154)	733.8 (313.9—1255.3)	791.3 (386.4—1453.9)
IPV	Female	Guinea	18.7 (6.07—37.0)	60.1 (25.9—95.3)	1019.7 (328.3—2000.7)	1390.8 (582.8—2231.9)
IPV	Female	Guinea-Bissau	2.39 (1.17—4.48)	7.71 (3.81—12.8)	762.3 (348.7—1466.4)	1157.7 (530.6—2022.5)
IPV	Female	Liberia	9.65 (2.86—18.8)	26.8 (10.8—46.8)	1160.6 (297.6—2265.7)	1383.3 (522.9—2386.2)
IPV	Female	Mali	18.1 (7.02—36.0)	75.1 (35.5—121)	703.2 (270.3—1415.6)	1107.7 (518.1—1825.9)
IPV	Female	Mauritania	2.97 (1.16—6.87)	6.22 (3.01—11.7)	480.6 (179.9—1125.5)	435.6 (201.6—867.1)
IPV	Female	Niger	10.3 (4.30—23.6)	36.7 (16.4—70.4)	452.7 (182.6—1062.1)	497.5 (215.0—980.6)
IPV	Female	Nigeria	153 (78.8—245)	617 (293—992)	571.2 (288.9—916.7)	830.6 (375.7—1376.2)
IPV	Female	Sao Tome and Principe	0.160 (0.0804—0.276)	0.475 (0.224—0.789)	457.4 (231.8—809.2)	629.0 (316.7—1055.4)
IPV	Female	Senegal	8.74 (3.96—16.0)	27.8 (13.9—47.1)	395.0 (172.4—734.6)	482.7 (242.3—811.6)
IPV	Female	Sierra Leone	15.7 (0.935—31.5)	39.9 (17.1—63.2)	1165.5 (73.4—2304.5)	1449.0 (605.8—2317.2)
IPV	Female	Togo	7.25 (3.29—12.6)	27.9 (13.5—47.3)	655.7 (292.1—1155.9)	975.5 (450.6—1661.1)
SVAC	Both	Global	19,400 (9,890—33,000)	32,200 (16,400—52,500)	532.3 (270.7—903.0)	533.0 (273.4—868.0)
SVAC	Both	Central Europe, Eastern Europe, and Central Asia	1,680 (677—3,130)	1,650 (752—3,240)	527.5 (212.1—979.1)	479.3 (215.3—947.0)
SVAC	Both	Central Asia	141 (53.9—278)	214 (80.9—447)	317.6 (120.9—626.0)	298.5 (112.1—627.6)
SVAC	Both	Armenia	6.53 (2.61—12.2)	6.28 (2.64—10.8)	271.4 (108.8—507.5)	255.6 (106.7—432.7)
SVAC	Both	Azerbaijan	12.1 (4.28—24.9)	20.6 (7.74—38.0)	249.3 (87.1—512.5)	243.7 (91.9—448.0)
SVAC	Both	Georgia	11.4 (4.59—21.4)	8.42 (4.03—13.6)	271.7 (109.2—507.8)	293.2 (145.2—474.6)
SVAC	Both	Kazakhstan	45.7 (17.3—91.4)	52.4 (18.9—113)	392.1 (148.4—783.0)	358.5 (128.3—774.6)
SVAC	Both	Kyrgyzstan	9.94 (3.66—20.4)	13.3 (4.75—28.2)	360.5 (133.8—744.3)	268.0 (95.4—566.3)
SVAC	Both	Mongolia	4.36 (1.62—8.67)	8.90 (4.46—15.2)	345.2 (130.6—688.5)	364.5 (181.4—629.0)
SVAC	Both	Tajikistan	8.43 (3.08—17.4)	18.2 (6.36—39.7)	282.5 (103.2—582.2)	270.4 (94.4—595.0)
SVAC	Both	Turkmenistan	6.49 (2.38—13.3)	12.4 (4.40—27.2)	295.0 (108.6—597.2)	312.3 (110.5—687.4)
SVAC	Both	Uzbekistan	36.0 (13.3—74.1)	73.2 (25.4—161)	296.4 (108.7—607.9)	292.9 (101.1—651.8)

Risk Factor	Sex	Location	Number of DALYs (thousands)		Rate of DALYs (per 100,000)	
			1990	2023	1990	2023
SVAC	Both	Central Europe	443 (192—792)	391 (207—658)	455.9 (198.0—810.3)	399.1 (211.6—656.8)
SVAC	Both	Albania	6.58 (2.77—11.6)	5.78 (2.64—9.85)	293.5 (124.6—523.1)	290.7 (130.6—495.6)
SVAC	Both	Bosnia and Herzegovina	19.4 (8.59—35.2)	13.3 (5.67—25.3)	558.8 (246.3—1014.4)	481.0 (219.1—887.2)
SVAC	Both	Bulgaria	25.7 (10.7—47.2)	18.1 (9.16—29.2)	368.9 (156.0—678.0)	314.1 (159.0—502.8)
SVAC	Both	Croatia	17.3 (6.79—32.9)	13.0 (6.52—22.1)	431.2 (169.9—819.4)	387.9 (196.0—635.7)
SVAC	Both	Czechia	36.6 (18.9—58.4)	39.3 (19.8—59.6)	456.2 (237.7—728.0)	430.6 (215.8—672.9)
SVAC	Both	Hungary	53.0 (21.8—100)	34.2 (18.4—54.0)	634.3 (260.7—1194.7)	411.0 (221.3—653.3)
SVAC	Both	Montenegro	1.80 (0.674—3.39)	1.85 (0.818—3.39)	385.6 (144.0—726.4)	342.9 (151.7—629.3)
SVAC	Both	North Macedonia	5.68 (2.12—10.6)	6.15 (2.75—11.8)	384.2 (143.1—715.3)	390.6 (179.9—700.3)
SVAC	Both	Poland	146 (61.5—267)	150 (78.9—252)	489.4 (205.1—891.8)	455.6 (240.6—769.8)
SVAC	Both	Romania	60.7 (23.1—116)	47.0 (24.8—75.3)	337.7 (128.9—642.5)	294.9 (157.2—470.1)
SVAC	Both	Serbia	37.2 (14.2—70.6)	32.2 (13.1—65.8)	501.4 (191.7—953.1)	405.6 (171.5—788.7)
SVAC	Both	Slovakia	16.9 (6.60—31.3)	16.3 (8.40—26.4)	425.6 (166.6—785.3)	357.2 (184.6—592.5)
SVAC	Both	Slovenia	9.27 (3.77—17.4)	8.35 (4.52—13.7)	588.8 (238.7—1104.9)	457.0 (243.4—752.7)
SVAC	Both	Eastern Europe	1,100 (429—2,090)	1,050 (421—2,150)	621.7 (242.2—1176.3)	605.9 (242.7—1268.9)
SVAC	Both	Belarus	40.3 (15.5—77.0)	42.1 (17.0—82.7)	502.9 (192.3—959.0)	524.1 (208.8—1041.3)
SVAC	Both	Estonia	7.13 (2.93—13.1)	5.80 (3.00—9.85)	588.2 (239.6—1084.4)	536.1 (267.9—954.6)
SVAC	Both	Latvia	12.1 (4.91—22.6)	7.52 (4.05—12.3)	568.0 (228.6—1057.3)	481.9 (259.5—786.6)
SVAC	Both	Lithuania	17.1 (6.82—32.1)	12.1 (6.45—20.5)	603.6 (240.0—1129.4)	518.4 (274.3—888.6)
SVAC	Both	Republic of Moldova	17.9 (7.28—33.1)	15.0 (6.91—28.8)	548.0 (223.5—1014.8)	479.8 (229.0—912.6)
SVAC	Both	Russian Federation	798 (312—1,530)	779 (308—1,620)	675.5 (262.8—1291.7)	628.6 (248.8—1335.3)
SVAC	Both	Ukraine	207 (81.7—392)	185 (75.0—377)	501.0 (197.6—946.3)	563.6 (228.3—1162.9)
SVAC	Both	High-income	4,410 (2,310—7,170)	6,760 (3,150—10,900)	621.3 (323.9—1005.0)	794.3 (358.7—1311.8)
SVAC	Both	Australasia	117 (58.5—187)	218 (104—359)	751.5 (374.8—1198.3)	897.2 (427.4—1493.4)
SVAC	Both	Australia	91.5 (44.9—150)	182 (85.3—301)	703.8 (344.3—1155.0)	886.5 (416.1—1492.7)
SVAC	Both	New Zealand	25.7 (13.6—39.5)	36.9 (18.1—60.4)	983.6 (518.3—1509.7)	952.2 (468.2—1557.8)
SVAC	Both	High-income Asia Pacific	692 (294—1,230)	840 (327—1,600)	504.2 (212.8—897.2)	572.4 (228.0—1080.4)
SVAC	Both	Brunei Darussalam	0.742 (0.292—1.39)	1.65 (0.657—3.20)	453.2 (183.6—832.1)	441.7 (174.4—855.7)
SVAC	Both	Japan	491 (212—877)	545 (207—1,040)	484.6 (208.6—861.4)	567.0 (218.6—1062.3)
SVAC	Both	Republic of Korea	188 (73.3—345)	272 (112—529)	563.5 (220.6—1034.1)	603.5 (248.6—1165.2)
SVAC	Both	Singapore	12.3 (5.10—21.8)	20.4 (7.61—39.3)	499.6 (202.2—892.0)	440.9 (176.9—827.4)
SVAC	Both	High-income North America	1,620 (858—2,430)	3,370 (1,200—5,940)	744.9 (394.7—1111.6)	1176.7 (405.7—2095.9)
SVAC	Both	Canada	124 (62.6—195)	219 (91.4—373)	573.6 (289.3—900.5)	743.6 (316.7—1255.1)
SVAC	Both	Greenland	1.09 (0.380—2.15)	0.748 (0.317—1.41)	2395.9 (839.5—4692.6)	1811.7 (783.9—3378.0)
SVAC	Both	United States of America	1,500 (790—2,260)	3,150 (1,100—5,540)	763.4 (403.6—1146.8)	1226.2 (415.1—2189.7)
SVAC	Both	Southern Latin America	215 (90.6—396)	337 (154—652)	617.3 (261.5—1139.3)	615.9 (281.4—1184.9)
SVAC	Both	Argentina	126 (50.4—232)	215 (87.0—434)	545.2 (218.3—1004.4)	584.6 (238.4—1176.4)
SVAC	Both	Chile	78.2 (33.9—140)	106 (50.5—190)	824.8 (359.8—1470.6)	694.5 (332.6—1234.2)
SVAC	Both	Uruguay	11.0 (4.18—21.3)	15.8 (6.13—33.0)	479.2 (182.3—925.3)	597.2 (233.0—1238.0)
SVAC	Both	Western Europe	1,760 (837—3,130)	1,990 (1,050—3,290)	573.8 (271.0—1013.4)	574.1 (299.8—962.1)
SVAC	Both	Andorra	0.228 (0.0861—0.417)	0.367 (0.135—0.730)	497.7 (187.9—911.7)	489.3 (182.2—963.2)
SVAC	Both	Austria	32.2 (12.9—59.2)	32.5 (17.1—53.9)	513.3 (205.1—940.2)	448.3 (227.5—768.5)
SVAC	Both	Belgium	38.3 (18.5—65.0)	47.1 (25.6—73.7)	475.9 (226.3—811.6)	509.8 (283.2—792.3)
SVAC	Both	Cyprus	2.09 (0.736—4.23)	4.13 (1.80—7.39)	362.4 (127.9—730.5)	370.7 (158.7—663.9)
SVAC	Both	Denmark	30.1 (12.1—58.1)	27.9 (13.8—49.4)	724.5 (294.9—1383.2)	601.0 (308.3—1049.1)
SVAC	Both	Finland	28.0 (15.1—42.9)	24.6 (12.4—38.9)	699.7 (372.7—1052.5)	571.7 (282.3—925.7)
SVAC	Both	France	327 (140—593)	350 (186—578)	717.2 (305.9—1294.6)	642.6 (332.4—1040.3)
SVAC	Both	Germany	397 (160—741)	364 (197—577)	598.8 (240.9—1140.7)	544.7 (299.1—878.5)
SVAC	Both	Greece	32.3 (11.9—62.6)	36.5 (15.7—66.3)	394.0 (144.1—765.9)	441.9 (183.8—804.8)
SVAC	Both	Iceland	1.06 (0.432—1.98)	1.80 (0.849—3.13)	560.6 (228.8—1043.3)	591.3 (278.9—1013.4)

Risk Factor	Sex	Location	Number of DALYs (thousands)		Rate of DALYs (per 100,000)	
			1990	2023	1990	2023
SVAC	Both	Ireland	17.6 (8.77—29.4)	27.0 (11.7—43.9)	677.5 (336.8—1133.2)	663.1 (286.9—1094.3)
SVAC	Both	Israel	18.1 (7.63—32.5)	37.3 (17.1—66.7)	524.6 (221.1—940.4)	526.5 (240.8—936.4)
SVAC	Both	Italy	184 (70.5—365)	202 (92.7—346)	396.0 (151.0—782.6)	428.7 (183.9—747.0)
SVAC	Both	Luxembourg	2.01 (0.833—3.59)	2.83 (1.41—4.58)	643.0 (266.2—1157.4)	525.6 (262.7—860.2)
SVAC	Both	Malta	1.16 (0.484—1.99)	2.03 (0.805—3.69)	405.9 (167.6—701.8)	440.0 (160.5—821.9)
SVAC	Both	Monaco	0.134 (0.0528—0.252)	0.165 (0.0636—0.343)	540.5 (220.0—1005.7)	568.6 (224.9—1165.1)
SVAC	Both	Netherlands	83.3 (42.3—137)	105 (51.5—168)	684.9 (353.9—1132.6)	739.1 (359.6—1196.4)
SVAC	Both	Norway	22.6 (9.51—40.5)	27.4 (13.3—46.0)	680.9 (286.0—1228.1)	635.1 (292.9—1095.7)
SVAC	Both	Portugal	40.3 (15.0—80.7)	39.7 (19.3—71.4)	509.3 (189.7—1016.9)	460.6 (213.7—817.1)
SVAC	Both	San Marino	0.0806 (0.0310—0.148)	0.129 (0.0472—0.260)	474.9 (182.7—872.3)	462.0 (172.6—907.9)
SVAC	Both	Spain	151 (78.1—240)	140 (62.4—238)	494.8 (253.4—786.6)	361.7 (162.5—628.9)
SVAC	Both	Sweden	36.0 (17.7—62.3)	50.0 (25.6—77.8)	532.8 (267.1—919.6)	611.0 (313.8—942.2)
SVAC	Both	Switzerland	40.8 (17.9—76.1)	40.3 (19.8—65.2)	731.1 (320.7—1356.2)	563.9 (264.8—920.8)
SVAC	Both	United Kingdom	278 (136—450)	428 (192—756)	621.4 (305.6—1005.9)	820.4 (361.0—1456.6)
SVAC	Both	Latin America and Caribbean	1,230 (546—2,140)	2,580 (1,370—4,210)	502.0 (221.9—890.2)	563.4 (300.8—922.5)
SVAC	Both	Andean Latin America	110 (43.3—197)	240 (115—429)	467.5 (184.7—839.6)	489.7 (234.7—875.5)
SVAC	Both	Bolivia (Plurinational State of)	16.9 (5.99—33.0)	41.9 (15.8—90.5)	453.1 (160.1—866.5)	480.1 (181.6—1038.6)
SVAC	Both	Ecuador	26.5 (12.0—45.4)	62.7 (29.7—111)	420.5 (193.5—725.0)	475.2 (224.4—839.3)
SVAC	Both	Peru	67.0 (25.2—119)	135 (59.2—246)	493.2 (183.9—883.1)	500.9 (219.2—911.9)
SVAC	Both	Caribbean	152 (63.3—304)	236 (111—445)	638.5 (266.8—1275.9)	644.0 (308.1—1215.2)
SVAC	Both	Antigua and Barbuda	0.180 (0.0657—0.356)	0.365 (0.141—0.759)	444.0 (162.5—876.1)	480.5 (183.9—1000.4)
SVAC	Both	Bahamas	0.832 (0.303—1.67)	1.71 (0.643—3.58)	478.5 (182.1—972.2)	507.1 (189.6—1062.6)
SVAC	Both	Barbados	0.870 (0.328—1.68)	1.21 (0.459—2.63)	459.4 (174.0—889.3)	469.7 (183.1—991.8)
SVAC	Both	Belize	0.389 (0.190—0.661)	1.36 (0.641—2.31)	380.9 (184.9—681.2)	463.2 (218.1—792.9)
SVAC	Both	Bermuda	0.221 (0.0844—0.425)	0.236 (0.0879—0.493)	453.4 (173.1—874.1)	454.2 (171.9—936.5)
SVAC	Both	Cuba	48.2 (18.1—92.1)	49.6 (19.0—103)	576.6 (215.2—1101.1)	512.1 (195.4—1058.2)
SVAC	Both	Dominica	0.204 (0.0743—0.399)	0.272 (0.105—0.573)	423.7 (156.0—823.2)	482.3 (191.2—1007.4)
SVAC	Both	Dominican Republic	20.3 (7.50—39.6)	45.2 (17.4—94.0)	457.4 (170.7—881.8)	547.9 (210.8—1143.1)
SVAC	Both	Grenada	0.309 (0.122—0.568)	0.503 (0.239—0.903)	514.9 (207.5—938.7)	549.2 (262.2—968.7)
SVAC	Both	Guyana	3.24 (1.26—6.42)	4.54 (1.77—9.35)	676.9 (258.4—1374.8)	755.6 (295.1—1551.3)
SVAC	Both	Haiti	43.0 (12.7—90.4)	84.1 (42.5—145)	1174.3 (360.4—2473.7)	941.3 (458.6—1629.8)
SVAC	Both	Jamaica	6.67 (2.95—11.8)	11.8 (5.81—19.0)	449.2 (200.6—811.3)	525.0 (257.6—841.8)
SVAC	Both	Puerto Rico	14.5 (5.73—28.8)	15.4 (5.32—32.9)	553.0 (219.1—1104.6)	585.0 (197.9—1227.0)
SVAC	Both	Saint Kitts and Nevis	0.137 (0.0528—0.268)	0.224 (0.0880—0.468)	531.9 (205.6—1036.6)	556.5 (217.0—1162.3)
SVAC	Both	Saint Lucia	0.417 (0.158—0.805)	0.734 (0.278—1.58)	513.9 (193.3—997.9)	490.2 (186.5—1041.3)
SVAC	Both	Saint Vincent and the Grenadines	0.319 (0.124—0.613)	0.475 (0.188—1.02)	492.1 (193.3—955.6)	513.3 (200.8—1081.7)
SVAC	Both	Suriname	1.59 (0.602—3.20)	3.27 (1.29—6.50)	614.3 (229.5—1244.2)	732.4 (286.0—1452.2)
SVAC	Both	Trinidad and Tobago	5.01 (1.92—9.92)	7.04 (2.70—14.6)	639.9 (239.3—1303.3)	607.8 (240.0—1266.6)
SVAC	Both	United States Virgin Islands	0.399 (0.162—0.755)	0.403 (0.164—0.823)	531.0 (212.9—1001.5)	615.6 (256.1—1229.1)
SVAC	Both	Central Latin America	477 (214—850)	1,050 (512—1,690)	487.3 (213.0—875.1)	529.0 (257.6—852.5)
SVAC	Both	Colombia	96.0 (37.5—177)	201 (89.3—372)	451.6 (175.8—832.6)	493.8 (221.1—907.5)
SVAC	Both	Costa Rica	12.4 (6.12—21.1)	30.5 (16.7—45.6)	644.8 (321.3—1121.8)	730.4 (405.6—1090.1)
SVAC	Both	El Salvador	19.0 (9.02—35.4)	24.9 (12.7—41.8)	608.0 (277.8—1181.3)	534.9 (273.5—907.6)
SVAC	Both	Guatemala	23.7 (11.3—42.2)	61.8 (32.4—106)	555.0 (262.4—1015.5)	551.9 (293.2—960.1)
SVAC	Both	Honduras	13.0 (6.22—22.0)	36.2 (19.5—56.7)	519.0 (240.9—883.0)	503.0 (268.7—803.6)
SVAC	Both	Mexico	241 (111—426)	554 (280—906)	489.1 (217.6—872.6)	545.4 (274.7—890.4)
SVAC	Both	Nicaragua	10.7 (5.04—17.8)	25.8 (12.8—42.6)	513.0 (233.6—873.6)	526.1 (259.8—874.9)
SVAC	Both	Panama	6.35 (2.51—11.8)	14.4 (5.98—29.7)	406.4 (160.4—768.5)	464.2 (192.1—956.0)
SVAC	Both	Venezuela (Bolivarian Republic of)	54.6 (22.0—102)	98.8 (38.9—200)	465.3 (189.5—877.9)	497.5 (197.2—1008.0)
SVAC	Both	Tropical Latin America	495 (201—894)	1,050 (531—1,730)	492.7 (202.7—886.2)	610.2 (307.4—1001.2)

Risk Factor	Sex	Location	Number of DALYs (thousands)		Rate of DALYs (per 100,000)	
			1990	2023	1990	2023
SVAC	Both	Brazil	486 (198—879)	1,030 (519—1,700)	495.8 (204.4—892.7)	614.1 (308.3—1010.3)
SVAC	Both	Paraguay	8.66 (3.44—16.0)	22.7 (11.1—41.3)	362.9 (139.7—683.3)	486.8 (238.5—886.9)
SVAC	Both	North Africa and Middle East	752 (252—1,520)	1,820 (633—3,760)	368.0 (125.2—739.6)	394.9 (137.9—832.0)
SVAC	Both	Afghanistan	18.0 (5.41—38.9)	67.4 (21.6—147)	311.3 (94.8—665.2)	340.5 (109.1—725.2)
SVAC	Both	Algeria	54.1 (17.7—111)	123 (40.4—261)	357.9 (114.8—723.3)	381.5 (124.9—820.8)
SVAC	Both	Bahrain	1.47 (0.510—2.98)	5.51 (1.97—11.5)	432.2 (149.2—898.9)	433.9 (152.3—914.2)
SVAC	Both	Egypt	113 (35.7—226)	268 (92.6—548)	342.0 (107.6—679.3)	371.8 (129.1—790.5)
SVAC	Both	Iran (Islamic Republic of)	157 (46.4—336)	336 (96.4—716)	469.9 (140.4—970.0)	487.7 (143.0—1047.5)
SVAC	Both	Iraq	40.1 (13.1—81.1)	131 (42.5—288)	388.3 (126.1—788.1)	439.9 (144.8—972.4)
SVAC	Both	Jordan	7.00 (2.21—14.3)	32.2 (10.2—65.9)	336.7 (109.8—690.8)	336.9 (109.7—694.1)
SVAC	Both	Kuwait	3.90 (1.35—7.87)	14.7 (5.12—30.6)	338.3 (116.8—679.4)	371.2 (128.1—796.6)
SVAC	Both	Lebanon	7.24 (2.16—15.4)	16.2 (4.77—34.8)	345.1 (101.8—733.9)	374.7 (110.1—807.1)
SVAC	Both	Libya	9.13 (2.99—18.8)	23.5 (7.56—50.8)	364.3 (118.5—742.6)	409.4 (131.8—900.4)
SVAC	Both	Morocco	56.7 (19.1—115)	106 (33.7—234)	354.0 (118.9—720.2)	374.2 (118.5—823.7)
SVAC	Both	Oman	3.69 (1.22—7.53)	13.1 (4.32—27.3)	320.4 (109.4—650.1)	338.8 (113.3—737.1)
SVAC	Both	Palestine	3.60 (1.04—8.04)	12.2 (3.64—26.0)	334.7 (98.3—745.9)	359.2 (114.2—785.5)
SVAC	Both	Qatar	1.19 (0.432—2.27)	9.46 (3.29—19.2)	378.4 (139.1—711.2)	352.4 (120.5—745.1)
SVAC	Both	Saudi Arabia	38.5 (13.7—73.8)	106 (38.2—223)	399.5 (144.7—768.2)	420.7 (148.3—935.4)
SVAC	Both	Sudan	41.2 (14.1—81.2)	103 (36.4—223)	360.1 (123.0—719.2)	407.6 (146.2—895.6)
SVAC	Both	Syrian Arab Republic	25.9 (8.00—57.8)	53.3 (17.0—119)	376.9 (116.4—816.6)	406.6 (130.0—918.6)
SVAC	Both	Tunisia	19.5 (6.12—40.7)	36.9 (12.0—80.9)	374.5 (116.3—789.6)	392.1 (125.6—858.5)
SVAC	Both	Türkiye	123 (47.9—228)	250 (113—446)	322.5 (127.2—601.3)	373.2 (172.8—660.6)
SVAC	Both	United Arab Emirates	5.08 (1.77—10.2)	35.2 (12.1—71.5)	402.1 (147.4—772.2)	391.3 (131.3—819.8)
SVAC	Both	Yemen	22.6 (7.67—48.0)	77.2 (25.2—165)	337.8 (112.5—729.0)	357.2 (116.9—761.7)
SVAC	Both	South Asia	4,060 (1,700—7,230)	8,360 (4,260—13,000)	604.6 (256.8—1065.2)	609.9 (311.3—982.1)
SVAC	Both	Bangladesh	395 (201—645)	577 (269—959)	651.1 (321.9—1073.4)	475.0 (220.9—792.5)
SVAC	Both	Bhutan	1.43 (0.640—2.57)	2.54 (1.14—4.75)	423.8 (188.3—758.9)	441.3 (196.8—826.3)
SVAC	Both	India	3,330 (1,360—5,940)	6,930 (3,500—10,500)	619.5 (248.9—1100.0)	642.5 (327.2—1004.6)
SVAC	Both	Nepal	61.1 (21.9—124)	122 (47.9—246)	536.4 (193.2—1078.3)	538.4 (212.0—1090.3)
SVAC	Both	Pakistan	275 (100—534)	725 (296—1,470)	452.6 (164.1—872.8)	492.6 (199.7—996.2)
SVAC	Both	Southeast Asia, East Asia, and Oceania	5,100 (2,560—8,590)	5,140 (2,550—8,270)	411.9 (203.2—703.7)	286.1 (142.3—452.3)
SVAC	Both	East Asia	4,240 (2,100—7,040)	3,460 (1,590—5,680)	454.0 (222.5—767.4)	281.1 (130.3—457.3)
SVAC	Both	China	4,140 (2,050—6,820)	3,310 (1,530—5,490)	458.6 (226.1—770.9)	278.1 (130.2—452.1)
SVAC	Both	Democratic People's Republic of Korea	48.3 (17.7—98.6)	71.0 (26.2—153)	319.0 (116.9—655.2)	322.3 (120.0—704.3)
SVAC	Both	Taiwan	46.7 (18.3—91.7)	81.1 (29.1—169)	313.7 (122.1—617.5)	399.3 (146.5—833.6)
SVAC	Both	Oceania	14.2 (6.05—25.6)	37.0 (18.1—64.6)	393.3 (152.9—740.6)	416.2 (198.9—745.9)
SVAC	Both	American Samoa	0.0966 (0.0364—0.193)	0.129 (0.0535—0.259)	341.3 (124.1—706.2)	358.0 (148.3—718.1)
SVAC	Both	Cook Islands	0.0733 (0.0251—0.149)	0.0567 (0.0213—0.119)	591.5 (196.8—1213.4)	514.9 (196.1—1078.9)
SVAC	Both	Fiji	2.65 (1.04—5.04)	4.29 (1.72—8.67)	615.1 (223.9—1235.7)	652.7 (257.1—1356.1)
SVAC	Both	Guam	0.467 (0.174—0.893)	0.626 (0.231—1.30)	469.7 (173.3—907.2)	530.2 (195.4—1104.9)
SVAC	Both	Kiribati	0.291 (0.123—0.535)	0.546 (0.258—0.917)	632.6 (261.4—1165.9)	638.7 (290.4—1100.5)
SVAC	Both	Marshall Islands	0.113 (0.0424—0.221)	0.140 (0.0507—0.298)	484.2 (176.5—990.9)	502.7 (178.1—1088.2)
SVAC	Both	Micronesia (Federated States of)	0.302 (0.103—0.599)	0.421 (0.152—0.913)	544.1 (179.8—1145.7)	583.8 (206.7—1288.4)
SVAC	Both	Nauru	0.0459 (0.0185—0.0851)	0.0616 (0.0291—0.117)	820.5 (298.3—1597.4)	807.8 (349.8—1578.1)
SVAC	Both	Niue	0.00762 (0.00270—0.0155)	0.00640 (0.00236—0.0144)	506.4 (180.7—1028.8)	491.5 (185.6—1057.1)
SVAC	Both	Northern Mariana Islands	0.182 (0.0693—0.376)	0.204 (0.0787—0.409)	546.3 (204.3—1169.2)	550.7 (212.7—1115.9)
SVAC	Both	Palau	0.0526 (0.0210—0.101)	0.0743 (0.0287—0.157)	486.1 (191.5—932.7)	507.8 (199.6—1054.5)
SVAC	Both	Papua New Guinea	6.63 (2.82—12.0)	22.7 (10.6—40.6)	300.7 (123.3—562.4)	348.7 (163.5—645.4)

Risk Factor	Sex	Location	Number of DALYs (thousands)		Rate of DALYs (per 100,000)	
			1990	2023	1990	2023
SVAC	Both	Samoa	0.398 (0.155—0.797)	0.561 (0.226—1.07)	399.2 (154.3—808.1)	415.7 (168.2—799.5)
SVAC	Both	Solomon Islands	1.40 (0.592—2.49)	4.32 (1.99—6.81)	789.6 (309.8—1456.5)	825.2 (357.2—1401.7)
SVAC	Both	Tokelau	0.00456 (0.00163—0.00926)	0.00516 (0.00177—0.0108)	444.8 (161.3—906.4)	428.6 (146.3—889.5)
SVAC	Both	Tonga	0.196 (0.0753—0.392)	0.260 (0.119—0.467)	356.3 (134.9—735.7)	384.4 (174.9—678.7)
SVAC	Both	Tuvalu	0.0298 (0.0109—0.0601)	0.0411 (0.0151—0.0909)	487.6 (177.7—987.2)	543.1 (198.3—1197.9)
SVAC	Both	Vanuatu	0.369 (0.140—0.719)	0.964 (0.365—1.98)	438.0 (163.8—861.9)	459.5 (174.3—954.1)
SVAC	Both	Southeast Asia	844 (360—1,590)	1,640 (759—3,040)	288.4 (121.5—545.5)	297.3 (137.2—554.2)
SVAC	Both	Cambodia	16.3 (8.03—28.3)	38.5 (19.6—60.1)	293.8 (141.1—513.3)	310.9 (158.8—496.7)
SVAC	Both	Indonesia	221 (74.7—470)	489 (162—1,020)	193.7 (66.0—419.2)	217.8 (72.3—458.3)
SVAC	Both	Lao People's Democratic Republic	8.79 (4.05—16.7)	20.5 (10.2—32.8)	373.0 (171.3—711.9)	387.9 (191.6—635.5)
SVAC	Both	Malaysia	27.5 (9.90—51.7)	72.5 (28.3—144)	252.5 (90.9—479.5)	270.2 (106.1—541.2)
SVAC	Both	Maldives	0.331 (0.135—0.612)	1.26 (0.522—2.22)	292.8 (116.1—552.8)	284.5 (116.8—505.6)
SVAC	Both	Mauritius	4.25 (1.64—8.65)	5.76 (2.26—11.8)	571.9 (222.5—1177.3)	515.5 (203.6—1023.3)
SVAC	Both	Myanmar	113 (45.1—242)	205 (85.8—393)	459.1 (184.6—1003.2)	490.4 (205.4—945.1)
SVAC	Both	Philippines	97.2 (38.6—183)	232 (111—386)	258.1 (102.9—483.5)	286.0 (135.1—477.1)
SVAC	Both	Seychelles	0.153 (0.0589—0.298)	0.291 (0.109—0.598)	318.0 (124.2—633.8)	340.2 (127.5—704.3)
SVAC	Both	Sri Lanka	89.7 (37.1—169)	78.7 (32.8—139)	750.9 (313.6—1429.8)	437.3 (184.3—755.4)
SVAC	Both	Thailand	152 (68.5—294)	235 (116—396)	368.8 (163.3—710.0)	426.7 (215.8—701.7)
SVAC	Both	Timor-Leste	1.82 (0.800—3.50)	4.09 (2.22—6.47)	404.9 (179.2—785.6)	452.7 (240.1—739.3)
SVAC	Both	Viet Nam	111 (48.4—211)	256 (117—480)	267.8 (115.1—517.3)	321.3 (146.2—610.4)
SVAC	Both	Sub-Saharan Africa	2,200 (791—4,410)	5,870 (2,250—10,800)	841.1 (311.7—1722.1)	883.1 (314.1—1643.2)
SVAC	Both	Central Sub-Saharan Africa	226 (90.3—483)	590 (223—1,210)	776.0 (313.3—1679.8)	733.8 (278.5—1536.6)
SVAC	Both	Angola	35.4 (14.1—67.6)	170 (55.4—359)	643.8 (258.2—1213.7)	889.7 (289.6—1864.4)
SVAC	Both	Central African Republic	13.4 (3.80—31.2)	30.4 (9.18—70.6)	904.9 (259.1—2109.1)	904.9 (273.5—2074.5)
SVAC	Both	Congo	14.3 (3.82—34.1)	39.8 (9.32—87.0)	1114.6 (289.0—2673.1)	1089.9 (253.5—2395.5)
SVAC	Both	Democratic Republic of the Congo	158 (63.1—338)	324 (128—684)	784.2 (310.0—1677.4)	619.0 (251.3—1285.7)
SVAC	Both	Equatorial Guinea	1.57 (0.684—2.92)	13.5 (1.72—31.9)	702.6 (302.5—1288.7)	1427.9 (160.4—3482.5)
SVAC	Both	Gabon	3.91 (1.56—8.43)	11.9 (3.26—25.5)	709.9 (279.3—1533.3)	991.4 (267.4—2127.0)
SVAC	Both	Eastern Sub-Saharan Africa	1,010 (254—2,080)	2,100 (835—3,800)	1030.2 (275.1—2149.2)	849.8 (300.2—1577.4)
SVAC	Both	Burundi	33.1 (7.56—92.4)	46.9 (19.3—94.8)	1115.3 (258.9—3134.3)	610.7 (249.8—1242.9)
SVAC	Both	Comoros	0.938 (0.356—1.78)	1.99 (0.744—4.33)	383.5 (145.4—725.7)	363.2 (137.7—789.5)
SVAC	Both	Djibouti	1.14 (0.502—2.19)	6.79 (2.14—14.0)	510.2 (220.3—1003.5)	739.0 (229.8—1514.3)
SVAC	Both	Eritrea	14.9 (4.52—35.9)	30.6 (12.4—61.7)	793.0 (285.1—1860.8)	711.5 (296.0—1431.6)
SVAC	Both	Ethiopia	201 (73.0—395)	381 (193—637)	747.2 (278.5—1525.6)	589.6 (293.0—994.4)
SVAC	Both	Kenya	105 (35.9—207)	318 (112—579)	932.9 (322.7—1808.6)	1035.1 (315.0—1922.6)
SVAC	Both	Madagascar	38.7 (16.5—74.7)	103 (42.1—210)	608.1 (252.1—1181.0)	587.1 (236.4—1189.2)
SVAC	Both	Malawi	78.5 (9.81—165)	158 (29.9—345)	1549.1 (206.7—3319.9)	1490.8 (184.3—3332.3)
SVAC	Both	Mozambique	35.7 (13.5—71.5)	199 (14.4—503)	491.5 (183.3—997.4)	1168.4 (68.4—2962.8)
SVAC	Both	Rwanda	40.1 (18.2—79.4)	72.0 (37.6—115)	1108.2 (508.1—2223.6)	890.4 (443.5—1423.3)
SVAC	Both	Somalia	20.5 (8.64—40.0)	57.6 (23.3—118)	497.7 (213.6—968.3)	511.6 (210.0—1070.8)
SVAC	Both	South Sudan	19.3 (8.00—40.7)	41.6 (14.1—82.9)	617.0 (253.8—1307.6)	742.0 (255.8—1462.8)
SVAC	Both	Uganda	209 (-26.8—561)	220 (71.2—415)	2585.0 (-375.7—7037.4)	966.7 (233.1—1905.8)
SVAC	Both	United Republic of Tanzania	137 (34.0—297)	288 (115—508)	1051.5 (256.8—2360.7)	808.1 (296.6—1436.9)
SVAC	Both	Zambia	74.5 (3.05—189)	175 (19.4—377)	1841.5 (93.2—4726.9)	1607.1 (143.7—3548.3)
SVAC	Both	Southern Sub-Saharan Africa	281 (127—513)	1,040 (81.9—2,380)	888.0 (400.3—1648.2)	1647.2 (120.6—3760.8)
SVAC	Both	Botswana	9.99 (3.09—19.4)	31.9 (1.52—64.7)	1430.0 (464.6—2774.2)	1977.7 (63.3—4033.9)
SVAC	Both	Eswatini	2.71 (1.25—4.85)	14.4 (-0.420—32.7)	638.9 (287.3—1181.7)	1944.6 (-105.8—4457.8)
SVAC	Both	Lesotho	6.05 (2.54—11.6)	27.7 (-0.367—65.5)	728.4 (309.8—1419.3)	2283.6 (-63.4—5329.2)
SVAC	Both	Namibia	5.46 (2.26—10.2)	24.0 (4.44—53.1)	691.5 (286.6—1331.9)	1267.5 (214.4—2847.8)
SVAC	Both	South Africa	184 (84.1—329)	837 (72.0—1,890)	787.8 (357.7—1405.9)	1716.8 (145.5—3881.9)

Risk Factor	Sex	Location	Number of DALYs (thousands)		Rate of DALYs (per 100,000)	
			1990	2023	1990	2023
SVAC	Both	Zimbabwe	72.5 (0.352—164)	105 (5.42—244)	1341.3 (9.1—3109.8)	1198.5 (26.2—2836.6)
SVAC	Both	Western Sub-Saharan Africa	684 (302—1,300)	2,140 (913—3,830)	667.9 (300.3—1270.9)	760.2 (305.2—1365.9)
SVAC	Both	Benin	11.7 (4.77—21.9)	43.5 (18.0—85.9)	501.0 (212.8—923.0)	578.6 (248.2—1152.5)
SVAC	Both	Burkina Faso	47.4 (5.19—111)	64.3 (28.3—126)	1043.9 (102.3—2487.3)	505.2 (210.7—1007.6)
SVAC	Both	Cabo Verde	1.04 (0.447—1.96)	2.13 (0.924—4.13)	560.4 (247.1—1054.4)	581.3 (252.1—1126.4)
SVAC	Both	Cameroon	29.4 (13.1—55.0)	148 (52.8—274)	552.4 (242.1—1063.8)	809.3 (261.2—1542.4)
SVAC	Both	Chad	20.8 (8.61—40.1)	72.0 (28.0—150)	683.0 (289.1—1295.3)	802.8 (314.1—1642.3)
SVAC	Both	Côte d'Ivoire	111 (9.61—242)	244 (93.9—434)	1744.3 (143.3—3814.6)	1226.9 (413.5—2173.8)
SVAC	Both	Gambia	2.46 (1.05—4.42)	11.4 (3.48—25.2)	485.7 (207.0—863.4)	805.7 (245.8—1792.8)
SVAC	Both	Ghana	48.0 (21.2—95.9)	155 (60.3—301)	597.2 (269.5—1185.6)	751.2 (291.6—1453.4)
SVAC	Both	Guinea	17.2 (7.15—33.2)	54.3 (20.7—117)	534.9 (225.1—1020.9)	712.8 (279.0—1503.3)
SVAC	Both	Guinea-Bissau	2.98 (1.34—5.85)	10.3 (3.26—20.4)	585.5 (267.7—1155.3)	886.3 (256.9—1785.0)
SVAC	Both	Liberia	8.70 (3.81—17.7)	27.1 (10.4—52.2)	602.2 (262.6—1200.9)	740.4 (279.3—1408.2)
SVAC	Both	Mali	22.4 (9.33—42.2)	76.0 (30.7—150)	508.9 (217.4—978.7)	622.3 (246.8—1240.9)
SVAC	Both	Mauritania	5.87 (2.49—11.4)	15.2 (6.08—30.8)	535.9 (228.0—1044.0)	590.4 (234.7—1191.5)
SVAC	Both	Niger	18.3 (7.51—35.4)	67.0 (30.3—128)	474.6 (196.9—927.1)	527.5 (229.6—1062.7)
SVAC	Both	Nigeria	293 (147—503)	1,010 (381—1,750)	592.8 (290.2—1036.1)	773.7 (269.9—1325.6)
SVAC	Both	Sao Tome and Principe	0.207 (0.0787—0.408)	0.558 (0.223—1.01)	329.8 (124.3—653.6)	381.2 (158.6—688.5)
SVAC	Both	Senegal	19.3 (8.12—36.6)	59.5 (25.0—122)	499.6 (210.4—930.6)	559.9 (234.8—1136.8)
SVAC	Both	Sierra Leone	12.9 (5.44—22.9)	35.6 (14.2—69.6)	566.3 (240.3—991.7)	723.3 (285.7—1445.3)
SVAC	Both	Togo	11.3 (4.78—23.5)	39.5 (15.0—80.2)	617.3 (270.5—1286.6)	765.4 (290.2—1539.4)
SVAC	Female	Global	9,920 (4,940—16,700)	16,400 (8,580—26,200)	540.7 (269.9—911.9)	545.4 (284.1—866.5)
SVAC	Female	Central Europe, Eastern Europe, and Central Asia	637 (244—1,200)	640 (311—1,210)	379.9 (145.8—723.0)	359.0 (174.0—686.7)
SVAC	Female	Central Asia	75.9 (28.6—144)	116 (44.9—233)	328.7 (125.7—630.3)	320.8 (122.7—646.1)
SVAC	Female	Armenia	3.90 (1.53—7.22)	3.81 (1.39—6.40)	312.1 (122.2—578.7)	288.9 (100.3—494.9)
SVAC	Female	Azerbaijan	6.30 (2.03—13.3)	10.2 (3.66—19.4)	244.4 (79.4—516.8)	236.8 (85.1—447.8)
SVAC	Female	Georgia	6.91 (2.73—12.9)	4.93 (2.40—8.03)	307.6 (120.4—564.2)	338.1 (148.9—545.9)
SVAC	Female	Kazakhstan	22.4 (8.18—43.8)	24.7 (8.75—52.4)	369.4 (135.1—721.8)	328.3 (115.9—698.7)
SVAC	Female	Kyrgyzstan	5.07 (1.86—10.1)	7.31 (2.49—15.3)	351.7 (130.0—694.9)	286.3 (97.5—600.9)
SVAC	Female	Mongolia	2.92 (1.09—5.59)	4.98 (2.33—8.02)	462.4 (170.8—877.1)	400.3 (189.7—644.0)
SVAC	Female	Tajikistan	4.93 (1.72—9.71)	10.7 (3.71—22.8)	320.7 (112.1—636.6)	317.0 (108.7—688.0)
SVAC	Female	Turkmenistan	3.63 (1.29—7.12)	6.69 (2.37—14.6)	318.2 (112.8—639.2)	346.4 (122.5—757.4)
SVAC	Female	Uzbekistan	19.9 (7.08—39.1)	43.2 (14.7—95.7)	316.5 (112.1—624.5)	343.7 (115.6—765.5)
SVAC	Female	Central Europe	156 (61.9—286)	139 (66.2—224)	314.1 (125.0—573.8)	278.9 (129.8—449.7)
SVAC	Female	Albania	2.54 (0.848—5.32)	2.28 (0.872—3.85)	234.8 (75.5—496.8)	224.4 (83.9—378.5)
SVAC	Female	Bosnia and Herzegovina	6.52 (2.26—12.5)	4.97 (1.83—10.5)	374.3 (130.3—719.4)	353.1 (130.2—704.5)
SVAC	Female	Bulgaria	9.70 (4.33—19.1)	7.17 (3.11—12.3)	273.5 (123.3—530.4)	248.6 (98.8—436.3)
SVAC	Female	Croatia	5.94 (1.95—12.6)	4.35 (1.71—8.04)	290.1 (96.9—616.8)	257.5 (92.0—477.2)
SVAC	Female	Czechia	15.8 (8.06—27.2)	17.6 (8.18—29.0)	384.6 (197.0—663.7)	378.0 (168.4—644.6)
SVAC	Female	Hungary	18.1 (6.72—34.9)	13.2 (5.80—22.5)	414.8 (152.6—810.2)	313.3 (128.0—530.6)
SVAC	Female	Montenegro	0.561 (0.166—1.26)	0.571 (0.247—0.985)	238.9 (70.8—537.3)	210.6 (89.7—363.8)
SVAC	Female	North Macedonia	2.26 (0.754—4.56)	2.39 (0.989—4.95)	304.8 (101.6—612.5)	300.7 (121.0—569.2)
SVAC	Female	Poland	48.6 (19.8—86.7)	47.2 (21.5—78.2)	316.1 (128.0—563.0)	278.8 (125.3—444.8)
SVAC	Female	Romania	21.2 (6.91—45.6)	15.3 (5.41—29.0)	232.2 (74.6—506.6)	193.4 (65.2—369.5)
SVAC	Female	Serbia	13.7 (4.69—27.9)	12.7 (4.51—26.7)	365.3 (125.7—730.1)	327.2 (119.3—668.1)
SVAC	Female	Slovakia	5.56 (1.94—11.3)	5.81 (2.23—10.2)	272.0 (94.8—553.6)	253.8 (95.1—434.6)
SVAC	Female	Slovenia	3.29 (1.16—6.65)	2.98 (1.23—5.19)	405.8 (144.4—821.5)	342.7 (135.9—577.1)
SVAC	Female	Eastern Europe	404 (150—772)	385 (150—808)	428.3 (158.1—816.7)	418.0 (161.6—866.5)
SVAC	Female	Belarus	15.8 (5.55—30.3)	15.9 (6.04—32.4)	366.7 (128.5—706.1)	371.6 (136.8—750.1)

Risk Factor	Sex	Location	Number of DALYs (thousands)		Rate of DALYs (per 100,000)	
			1990	2023	1990	2023
SVAC	Female	Estonia	2.89 (1.14—5.33)	2.21 (0.963—3.67)	442.2 (177.7—819.1)	399.2 (163.2—654.1)
SVAC	Female	Latvia	4.51 (1.68—8.73)	2.75 (1.20—4.59)	391.4 (148.1—752.0)	332.7 (138.7—558.2)
SVAC	Female	Lithuania	5.82 (2.12—11.3)	4.02 (1.71—7.11)	387.2 (141.0—752.6)	328.5 (138.2—577.7)
SVAC	Female	Republic of Moldova	8.54 (3.34—15.7)	6.97 (3.02—13.1)	491.3 (193.8—903.5)	433.0 (190.6—791.2)
SVAC	Female	Russian Federation	280 (102—543)	278 (104—592)	445.8 (162.8—866.5)	421.6 (157.0—892.7)
SVAC	Female	Ukraine	87.1 (32.0—172)	74.7 (27.5—159)	390.2 (143.1—777.2)	424.5 (156.7—890.3)
SVAC	Female	High-income	2,300 (1,100—3,780)	3,510 (1,490—5,830)	636.3 (304.9—1040.8)	825.5 (333.6—1414.4)
SVAC	Female	Australasia	61.1 (29.7—96.0)	119 (47.7—205)	780.7 (378.7—1228.5)	975.9 (393.1—1703.7)
SVAC	Female	Australia	47.8 (23.5—76.0)	100 (40.4—170)	734.1 (361.3—1167.8)	973.0 (391.1—1674.9)
SVAC	Female	New Zealand	13.3 (5.98—21.4)	19.2 (7.40—33.5)	1006.7 (454.5—1629.1)	990.7 (379.0—1733.2)
SVAC	Female	High-income Asia Pacific	370 (157—672)	437 (177—828)	532.2 (222.4—966.9)	606.4 (251.2—1116.9)
SVAC	Female	Brunei Darussalam	0.387 (0.148—0.720)	0.874 (0.342—1.69)	511.4 (204.7—948.9)	500.8 (193.8—972.0)
SVAC	Female	Japan	268 (118—483)	292 (116—548)	520.7 (224.2—938.4)	605.6 (246.9—1102.9)
SVAC	Female	Republic of Korea	94.8 (38.8—182)	134 (53.6—258)	560.4 (229.8—1072.3)	627.3 (254.8—1196.1)
SVAC	Female	Singapore	6.70 (2.86—11.7)	10.8 (4.06—20.4)	544.8 (229.4—950.6)	473.9 (194.3—893.9)
SVAC	Female	High-income North America	846 (362—1,380)	1,720 (567—3,070)	763.9 (328.7—1247.0)	1197.0 (388.4—2169.0)
SVAC	Female	Canada	65.9 (27.7—115)	121 (47.7—205)	604.0 (258.4—1052.4)	825.3 (321.9—1402.9)
SVAC	Female	Greenland	0.379 (0.142—0.707)	0.326 (0.158—0.574)	1914.0 (723.7—3573.9)	1660.4 (804.9—2788.9)
SVAC	Female	United States of America	780 (337—1,280)	1,600 (507—2,900)	781.3 (334.0—1281.9)	1240.0 (385.3—2267.6)
SVAC	Female	Southern Latin America	112 (44.0—207)	180 (70.7—344)	626.2 (245.7—1155.2)	639.2 (251.7—1221.4)
SVAC	Female	Argentina	63.4 (23.2—126)	105 (39.8—215)	535.2 (194.5—1052.6)	554.6 (208.8—1130.7)
SVAC	Female	Chile	42.9 (18.5—77.9)	66.4 (26.4—121)	872.3 (376.3—1599.8)	865.4 (365.2—1569.4)
SVAC	Female	Uruguay	6.05 (2.11—12.2)	7.75 (2.85—15.9)	511.6 (178.4—1023.1)	562.4 (205.9—1151.3)
SVAC	Female	Western Europe	908 (384—1,570)	1,060 (473—1,730)	578.8 (245.0—1011.5)	612.4 (260.3—1019.3)
SVAC	Female	Andorra	0.112 (0.0385—0.216)	0.194 (0.0682—0.382)	527.6 (182.3—1020.2)	548.6 (194.7—1090.6)
SVAC	Female	Austria	12.8 (4.90—24.2)	12.9 (5.43—22.2)	395.4 (148.7—746.6)	366.8 (149.3—628.3)
SVAC	Female	Belgium	20.9 (10.2—35.8)	26.8 (12.0—44.1)	515.2 (252.0—882.8)	588.5 (264.9—974.1)
SVAC	Female	Cyprus	1.01 (0.316—2.13)	1.97 (0.676—3.65)	352.0 (110.0—742.2)	353.5 (119.0—680.2)
SVAC	Female	Denmark	13.8 (5.63—25.6)	13.6 (5.81—23.7)	661.0 (272.7—1205.6)	597.1 (263.6—1035.0)
SVAC	Female	Finland	14.3 (8.34—21.6)	13.4 (6.52—20.9)	702.1 (403.7—1049.0)	617.7 (281.3—1003.2)
SVAC	Female	France	176 (74.1—316)	199 (86.4—339)	757.0 (319.9—1339.9)	719.1 (297.3—1240.1)
SVAC	Female	Germany	205 (79.7—386)	180 (84.0—302)	609.2 (232.3—1137.3)	550.8 (247.6—908.6)
SVAC	Female	Greece	16.2 (5.24—34.2)	17.0 (5.44—31.5)	389.1 (125.4—810.3)	408.3 (126.0—802.6)
SVAC	Female	Iceland	0.610 (0.213—1.12)	1.05 (0.466—1.81)	649.9 (228.6—1185.1)	725.9 (320.9—1232.1)
SVAC	Female	Ireland	8.35 (3.89—14.1)	11.8 (4.36—20.5)	642.4 (301.3—1074.3)	579.6 (205.6—1010.5)
SVAC	Female	Israel	8.91 (3.51—16.9)	18.7 (7.67—34.2)	504.3 (199.1—956.8)	523.9 (215.4—958.9)
SVAC	Female	Italy	91.8 (31.4—177)	117 (46.2—201)	381.6 (127.1—727.2)	496.7 (182.2—895.4)
SVAC	Female	Luxembourg	1.00 (0.431—1.79)	1.53 (0.625—2.58)	637.3 (270.3—1128.6)	584.4 (245.2—976.7)
SVAC	Female	Malta	0.610 (0.246—1.20)	0.997 (0.350—1.86)	419.0 (167.8—829.7)	467.0 (150.1—916.2)
SVAC	Female	Monaco	0.0760 (0.0302—0.145)	0.0895 (0.0350—0.177)	592.3 (231.4—1132.5)	614.6 (249.7—1182.3)
SVAC	Female	Netherlands	50.7 (24.7—85.7)	65.0 (28.3—109)	833.7 (409.5—1421.4)	929.2 (386.0—1571.8)
SVAC	Female	Norway	11.7 (4.52—21.4)	15.2 (5.96—25.9)	704.7 (273.7—1269.8)	726.8 (275.3—1273.2)
SVAC	Female	Portugal	17.3 (5.57—37.8)	17.3 (6.35—32.6)	418.5 (133.5—922.9)	394.1 (134.3—731.6)
SVAC	Female	San Marino	0.0426 (0.0142—0.0833)	0.0739 (0.0262—0.149)	495.4 (168.8—968.3)	526.6 (179.9—1048.4)
SVAC	Female	Spain	77.3 (40.2—126)	64.0 (27.9—114)	497.6 (250.2—813.3)	330.4 (135.9—608.8)
SVAC	Female	Sweden	18.3 (9.24—31.4)	26.7 (13.1—42.3)	547.7 (273.9—927.3)	676.8 (297.7—1081.3)
SVAC	Female	Switzerland	20.1 (9.25—36.1)	22.1 (8.06—39.2)	718.6 (331.9—1265.3)	627.7 (205.0—1157.1)
SVAC	Female	United Kingdom	140 (62.1—236)	232 (92.1—435)	611.7 (272.7—1019.8)	877.3 (337.3—1640.9)
SVAC	Female	Latin America and Caribbean	642 (272—1,140)	1,370 (596—2,270)	506.6 (205.4—921.6)	582.4 (254.0—965.3)
SVAC	Female	Andean Latin America	63.0 (23.6—113)	128 (51.8—233)	514.5 (191.9—940.8)	516.7 (208.3—937.5)

Risk Factor	Sex	Location	Number of DALYs (thousands)		Rate of DALYs (per 100,000)	
			1990	2023	1990	2023
SVAC	Female	Bolivia (Plurinational State of)	8.62 (2.69—18.4)	21.1 (7.01—47.6)	440.2 (137.0—927.7)	473.0 (158.0—1065.7)
SVAC	Female	Ecuador	12.5 (5.76—21.8)	29.8 (13.7—50.8)	369.9 (172.2—634.5)	433.3 (200.0—742.3)
SVAC	Female	Peru	42.0 (16.0—77.0)	77.4 (28.6—140)	601.6 (220.5—1107.7)	573.9 (213.0—1034.5)
SVAC	Female	Caribbean	80.2 (31.4—163)	119 (50.9—237)	652.8 (257.1—1348.6)	637.1 (275.3—1260.4)
SVAC	Female	Antigua and Barbuda	0.0993 (0.0343—0.198)	0.197 (0.0725—0.418)	458.7 (157.5—906.3)	508.6 (184.1—1077.9)
SVAC	Female	Bahamas	0.444 (0.151—0.896)	0.935 (0.341—2.05)	488.6 (169.8—988.3)	533.0 (192.7—1170.2)
SVAC	Female	Barbados	0.473 (0.163—0.944)	0.659 (0.228—1.45)	470.1 (161.3—919.5)	490.9 (176.3—1050.9)
SVAC	Female	Belize	0.188 (0.0813—0.332)	0.652 (0.285—1.19)	370.7 (159.8—674.6)	432.6 (189.9—776.7)
SVAC	Female	Bermuda	0.115 (0.0400—0.237)	0.130 (0.0441—0.275)	457.2 (160.5—936.6)	492.3 (168.4—1025.6)
SVAC	Female	Cuba	26.2 (9.39—53.0)	23.5 (8.17—49.2)	625.5 (222.9—1262.8)	495.8 (171.5—1029.9)
SVAC	Female	Dominica	0.110 (0.0377—0.221)	0.138 (0.0497—0.311)	444.1 (149.4—883.8)	503.0 (183.5—1107.9)
SVAC	Female	Dominican Republic	11.3 (3.76—23.2)	23.5 (8.55—49.5)	489.9 (166.7—1006.7)	562.1 (203.6—1188.1)
SVAC	Female	Grenada	0.187 (0.0730—0.358)	0.282 (0.139—0.461)	595.1 (239.7—1138.5)	628.2 (301.3—1041.1)
SVAC	Female	Guyana	1.52 (0.575—2.95)	1.96 (0.741—4.40)	625.9 (234.9—1262.0)	640.8 (242.4—1433.4)
SVAC	Female	Haiti	23.8 (4.31—55.4)	44.2 (19.8—81.6)	1226.5 (265.1—2732.0)	944.7 (422.1—1758.4)
SVAC	Female	Jamaica	3.60 (1.56—6.27)	6.33 (2.99—10.1)	463.7 (194.6—815.7)	551.4 (261.9—885.4)
SVAC	Female	Puerto Rico	5.65 (1.82—11.7)	6.73 (2.24—14.8)	409.6 (132.0—846.1)	468.8 (153.3—1010.9)
SVAC	Female	Saint Kitts and Nevis	0.0721 (0.0264—0.142)	0.114 (0.0431—0.247)	527.5 (192.5—1027.6)	550.0 (204.8—1200.1)
SVAC	Female	Saint Lucia	0.224 (0.0798—0.442)	0.372 (0.133—0.806)	525.4 (185.6—1037.4)	495.3 (180.0—1047.7)
SVAC	Female	Saint Vincent and the Grenadines	0.162 (0.0570—0.322)	0.230 (0.0845—0.515)	487.7 (171.2—972.3)	509.9 (185.9—1114.1)
SVAC	Female	Suriname	0.731 (0.269—1.45)	1.43 (0.526—3.13)	565.5 (202.8—1121.5)	624.7 (228.3—1355.8)
SVAC	Female	Trinidad and Tobago	2.35 (0.879—4.66)	3.34 (1.20—7.60)	604.1 (219.8—1214.2)	576.1 (206.6—1289.0)
SVAC	Female	United States Virgin Islands	0.205 (0.0740—0.395)	0.192 (0.0740—0.394)	522.9 (189.5—996.2)	564.2 (215.7—1099.5)
SVAC	Female	Central Latin America	239 (105—438)	539 (250—878)	469.4 (202.6—868.6)	525.4 (243.7—855.5)
SVAC	Female	Colombia	50.9 (17.9—100)	105 (43.5—200)	467.2 (164.8—912.2)	504.9 (210.8—962.3)
SVAC	Female	Costa Rica	6.87 (3.16—11.2)	16.5 (7.11—27.3)	706.4 (330.3—1175.5)	788.0 (336.8—1306.1)
SVAC	Female	El Salvador	7.49 (3.97—11.8)	11.8 (6.15—19.3)	438.0 (220.9—740.7)	459.8 (239.6—750.6)
SVAC	Female	Guatemala	8.58 (4.20—14.5)	25.6 (12.8—40.6)	377.8 (175.0—664.0)	431.7 (219.9—684.8)
SVAC	Female	Honduras	6.38 (3.05—10.3)	18.8 (8.30—31.3)	493.6 (225.1—799.6)	495.8 (224.9—807.4)
SVAC	Female	Mexico	123 (55.0—220)	291 (101—558)	476.9 (217.5—870.7)	553.1 (191.0—1058.8)
SVAC	Female	Nicaragua	5.31 (2.54—8.80)	12.9 (5.81—22.3)	477.6 (210.6—826.3)	513.2 (234.0—880.3)
SVAC	Female	Panama	2.90 (1.01—5.78)	6.74 (2.54—14.6)	377.9 (131.5—756.8)	426.3 (160.5—928.0)
SVAC	Female	Venezuela (Bolivarian Republic of)	26.9 (9.68—54.3)	50.8 (18.7—105)	451.6 (163.3—911.8)	484.5 (179.0—1005.3)
SVAC	Female	Tropical Latin America	260 (99.7—472)	583 (233—985)	507.3 (192.4—935.7)	659.4 (261.7—1126.3)
SVAC	Female	Brazil	256 (97.9—465)	574 (228—971)	511.7 (193.6—942.4)	667.6 (264.1—1142.4)
SVAC	Female	Paraguay	3.90 (1.44—7.48)	8.94 (4.18—15.6)	322.5 (119.6—624.6)	379.2 (180.6—660.0)
SVAC	Female	North Africa and Middle East	358 (97.7—810)	817 (260—1,740)	352.1 (95.6—778.6)	371.7 (119.9—799.1)
SVAC	Female	Afghanistan	9.21 (2.30—22.2)	32.2 (8.80—73.9)	298.6 (73.7—707.5)	324.8 (91.7—758.1)
SVAC	Female	Algeria	25.3 (6.28—58.0)	54.8 (17.1—124)	331.9 (85.8—752.2)	341.4 (107.7—773.6)
SVAC	Female	Bahrain	0.514 (0.129—1.18)	1.69 (0.514—3.92)	382.7 (101.9—904.3)	381.7 (120.5—906.1)
SVAC	Female	Egypt	58.8 (15.5—132)	135 (42.4—297)	360.3 (96.3—799.3)	388.0 (123.6—855.2)
SVAC	Female	Iran (Islamic Republic of)	73.3 (18.1—183)	146 (35.7—341)	437.0 (107.8—1045.5)	431.9 (108.9—1010.6)
SVAC	Female	Iraq	18.0 (4.38—43.2)	55.1 (15.7—126)	352.9 (89.1—840.2)	380.9 (111.2—887.2)
SVAC	Female	Jordan	3.21 (0.802—7.53)	14.3 (3.93—32.5)	321.9 (81.5—760.6)	326.7 (90.9—740.7)
SVAC	Female	Kuwait	1.51 (0.382—3.31)	4.83 (1.41—11.0)	321.2 (82.3—725.5)	345.1 (105.1—797.8)
SVAC	Female	Lebanon	3.82 (0.896—9.22)	8.03 (2.10—18.8)	351.1 (84.3—830.9)	369.0 (97.1—858.2)
SVAC	Female	Libya	3.95 (0.956—9.22)	9.95 (2.89—22.7)	336.1 (83.0—765.3)	359.7 (105.1—834.9)
SVAC	Female	Morocco	27.9 (6.81—61.0)	48.5 (15.1—109)	337.9 (85.3—740.9)	343.9 (107.6—771.5)
SVAC	Female	Oman	1.26 (0.315—3.05)	4.27 (1.11—9.32)	303.1 (77.6—729.2)	316.3 (87.7—710.8)
SVAC	Female	Palestine	1.80 (0.418—4.39)	5.79 (1.51—13.1)	323.4 (79.3—797.9)	338.6 (90.7—795.3)

Risk Factor	Sex	Location	Number of DALYs (thousands)		Rate of DALYs (per 100,000)	
			1990	2023	1990	2023
SVAC	Female	Qatar	0.294 (0.0785—0.625)	2.44 (0.730—5.34)	337.8 (92.6—745.1)	328.9 (106.1—720.1)
SVAC	Female	Saudi Arabia	13.7 (3.41—31.5)	35.1 (10.2—80.0)	346.1 (90.2—770.7)	390.8 (118.8—903.5)
SVAC	Female	Sudan	20.5 (5.12—44.3)	49.1 (16.1—108)	340.6 (88.6—729.6)	393.1 (129.6—870.7)
SVAC	Female	Syrian Arab Republic	12.6 (2.89—31.2)	26.8 (7.40—63.9)	363.0 (87.0—862.4)	392.9 (109.2—946.5)
SVAC	Female	Tunisia	9.40 (2.32—21.7)	17.2 (4.73—39.5)	357.0 (89.6—832.3)	363.0 (101.0—833.9)
SVAC	Female	Türkiye	60.8 (22.5—120)	122 (51.0—217)	313.4 (117.0—626.2)	370.9 (157.3—633.3)
SVAC	Female	United Arab Emirates	1.40 (0.356—3.10)	7.87 (2.30—17.5)	365.9 (97.4—815.1)	359.2 (114.8—812.1)
SVAC	Female	Yemen	10.5 (2.57—23.6)	34.9 (10.9—78.4)	304.9 (76.4—694.9)	319.8 (97.8—715.1)
SVAC	Female	South Asia	2,390 (1,020—4,150)	4,770 (2,410—7,290)	724.0 (301.8—1261.4)	703.5 (354.3—1080.7)
SVAC	Female	Bangladesh	156 (74.3—260)	169 (67.2—296)	488.3 (237.1—809.0)	272.9 (108.2—485.7)
SVAC	Female	Bhutan	0.507 (0.186—1.00)	0.860 (0.351—1.57)	319.2 (114.3—628.0)	313.7 (125.7—584.4)
SVAC	Female	India	2,100 (872—3,590)	4,270 (2,190—6,400)	798.3 (326.1—1384.6)	801.5 (410.4—1203.1)
SVAC	Female	Nepal	27.5 (8.37—60.9)	49.4 (17.1—111)	456.9 (141.4—1005.5)	417.2 (144.7—938.7)
SVAC	Female	Pakistan	105 (30.8—219)	282 (95.4—631)	364.6 (106.6—764.2)	394.1 (133.4—900.0)
SVAC	Female	Southeast Asia, East Asia, and Oceania	2,450 (1,160—4,160)	2,200 (1,020—3,750)	403.1 (192.0—690.0)	246.8 (115.3—411.9)
SVAC	Female	East Asia	2,140 (999—3,710)	1,590 (691—2,670)	474.8 (224.3—821.9)	260.8 (110.9—447.5)
SVAC	Female	China	2,100 (979—3,650)	1,520 (641—2,560)	481.1 (227.5—834.4)	258.7 (110.1—439.4)
SVAC	Female	Democratic People's Republic of Korea	23.0 (7.83—48.5)	33.6 (11.5—76.9)	293.8 (100.1—622.3)	295.0 (100.9—679.2)
SVAC	Female	Taiwan	20.7 (7.49—42.5)	34.0 (11.3—74.1)	291.4 (104.4—598.7)	334.3 (112.7—725.9)
SVAC	Female	Oceania	6.25 (2.85—10.9)	16.8 (7.90—26.5)	358.8 (147.4—681.8)	389.0 (177.5—639.7)
SVAC	Female	American Samoa	0.0303 (0.00981—0.0631)	0.0417 (0.0174—0.0743)	217.2 (67.2—469.3)	234.5 (97.3—418.0)
SVAC	Female	Cook Islands	0.0301 (0.0103—0.0596)	0.0232 (0.00782—0.0461)	508.3 (165.3—1057.4)	402.9 (140.4—790.6)
SVAC	Female	Fiji	1.36 (0.562—2.37)	2.28 (0.887—4.19)	638.8 (228.4—1219.1)	692.6 (271.3—1269.9)
SVAC	Female	Guam	0.168 (0.0618—0.335)	0.204 (0.0678—0.446)	384.7 (140.6—783.6)	341.1 (114.1—744.0)
SVAC	Female	Kiribati	0.130 (0.0574—0.249)	0.260 (0.128—0.391)	571.5 (233.6—1096.6)	598.7 (306.4—913.8)
SVAC	Female	Marshall Islands	0.0361 (0.0129—0.0743)	0.0514 (0.0176—0.0999)	336.8 (111.5—701.7)	381.3 (129.6—763.5)
SVAC	Female	Micronesia (Federated States of)	0.0961 (0.0315—0.196)	0.151 (0.0512—0.304)	370.2 (116.5—819.8)	422.0 (138.4—849.5)
SVAC	Female	Nauru	0.0233 (0.00992—0.0431)	0.0318 (0.0141—0.0538)	887.1 (341.4—1766.6)	848.2 (359.0—1484.1)
SVAC	Female	Niue	0.00313 (0.00105—0.00681)	0.00276 (0.000931—0.00583)	406.7 (140.1—872.6)	399.6 (133.9—851.7)
SVAC	Female	Northern Mariana Islands	0.0604 (0.0218—0.123)	0.0656 (0.0223—0.138)	412.8 (143.2—889.6)	368.1 (124.1—771.1)
SVAC	Female	Palau	0.0253 (0.0102—0.0537)	0.0333 (0.0129—0.0640)	477.9 (186.1—1020.0)	519.7 (204.2—1000.2)
SVAC	Female	Papua New Guinea	2.66 (1.10—5.22)	9.47 (4.48—15.7)	245.7 (98.5—518.7)	297.4 (132.6—514.6)
SVAC	Female	Samoa	0.131 (0.0506—0.271)	0.214 (0.0829—0.358)	281.4 (103.5—616.3)	318.2 (125.9—528.3)
SVAC	Female	Solomon Islands	0.853 (0.374—1.41)	2.67 (1.30—4.18)	1024.0 (406.9—1793.7)	1045.5 (471.2—1623.0)
SVAC	Female	Tokelau	0.00214 (0.000711—0.00472)	0.00239 (0.000784—0.00509)	408.4 (136.7—895.0)	396.6 (129.4—840.7)
SVAC	Female	Tonga	0.0976 (0.0343—0.197)	0.133 (0.0584—0.222)	349.0 (119.2—716.1)	366.4 (167.3—630.6)
SVAC	Female	Tuvalu	0.0138 (0.00469—0.0294)	0.0176 (0.00606—0.0391)	418.4 (139.8—904.4)	468.1 (161.2—1038.7)
SVAC	Female	Vanuatu	0.147 (0.0527—0.301)	0.415 (0.148—0.889)	366.8 (123.6—759.0)	397.3 (140.1—866.2)
SVAC	Female	Southeast Asia	299 (114—597)	599 (245—1,120)	198.7 (74.0—402.6)	216.7 (88.7—405.9)
SVAC	Female	Cambodia	6.30 (2.70—11.5)	13.5 (6.04—22.5)	199.5 (82.6—381.5)	209.6 (95.1—349.8)
SVAC	Female	Indonesia	106 (32.4—239)	230 (73.6—530)	179.9 (56.4—406.8)	206.8 (66.5—478.5)
SVAC	Female	Lao People's Democratic Republic	3.75 (1.43—7.66)	7.79 (3.52—12.5)	296.7 (116.9—586.2)	297.1 (132.8—467.2)
SVAC	Female	Malaysia	11.5 (3.74—23.9)	30.0 (10.8—62.8)	210.2 (70.8—435.1)	236.9 (87.8—493.2)
SVAC	Female	Maldives	0.146 (0.0576—0.279)	0.442 (0.167—0.740)	269.3 (104.3—519.3)	270.5 (111.8—440.3)
SVAC	Female	Mauritius	1.38 (0.466—2.98)	1.80 (0.591—4.00)	362.3 (121.2—800.8)	318.9 (106.4—722.0)
SVAC	Female	Myanmar	29.4 (9.86—67.4)	61.5 (19.6—142)	227.4 (76.0—525.0)	279.4 (89.5—646.3)
SVAC	Female	Philippines	28.7 (9.63—61.5)	73.3 (26.3—135)	151.9 (51.0—334.0)	181.4 (67.5—333.7)
SVAC	Female	Seychelles	0.0463 (0.0137—0.106)	0.0834 (0.0279—0.198)	190.8 (57.4—443.4)	223.6 (74.9—522.9)

Risk Factor	Sex	Location	Number of DALYs (thousands)		Rate of DALYs (per 100,000)	
			1990	2023	1990	2023
SVAC	Female	Sri Lanka	21.8 (7.75—44.8)	25.3 (10.6—46.5)	359.8 (131.0—735.1)	266.5 (113.7—473.4)
SVAC	Female	Thailand	53.4 (23.3—104)	79.9 (34.2—132)	258.6 (112.2—509.8)	268.1 (111.4—455.3)
SVAC	Female	Timor-Leste	1.05 (0.461—1.94)	2.42 (1.24—3.59)	479.4 (214.5—918.9)	547.1 (285.7—821.8)
SVAC	Female	Viet Nam	35.6 (12.7—73.1)	72.5 (32.6—127)	160.2 (54.1—333.2)	184.0 (82.1—322.2)
SVAC	Female	Sub-Saharan Africa	1,150 (326—2,350)	3,090 (951—5,970)	836.2 (242.1—1765.3)	884.8 (260.4—1722.7)
SVAC	Female	Central Sub-Saharan Africa	114 (32.5—270)	292 (91.1—647)	743.1 (224.1—1738.7)	709.5 (221.4—1574.5)
SVAC	Female	Angola	15.3 (5.39—31.0)	92.2 (23.0—207)	547.5 (199.6—1112.1)	896.5 (227.9—1999.8)
SVAC	Female	Central African Republic	6.19 (1.05—16.0)	14.3 (2.62—33.8)	784.3 (132.9—2035.7)	821.6 (137.2—1900.1)
SVAC	Female	Congo	7.77 (0.907—20.2)	22.6 (3.29—53.2)	1138.1 (128.6—2929.3)	1235.8 (163.4—2881.1)
SVAC	Female	Democratic Republic of the Congo	82.0 (21.1—197)	149 (57.5—304)	770.3 (212.7—1813.3)	565.5 (216.8—1149.5)
SVAC	Female	Equatorial Guinea	0.714 (0.259—1.53)	6.89 (0.212—18.8)	570.1 (212.9—1209.0)	1498.1 (60.4—3946.2)
SVAC	Female	Gabon	1.81 (0.640—4.21)	6.56 (1.19—16.1)	642.5 (228.3—1489.7)	1046.8 (176.0—2578.1)
SVAC	Female	Eastern Sub-Saharan Africa	549 (94.3—1,220)	1,130 (362—2,180)	1047.8 (217.0—2270.1)	877.4 (260.2—1733.6)
SVAC	Female	Burundi	19.0 (1.95—54.9)	23.5 (9.02—49.8)	1196.4 (151.9—3466.1)	619.3 (247.7—1286.0)
SVAC	Female	Comoros	0.447 (0.158—0.928)	0.893 (0.310—1.89)	354.4 (129.5—730.9)	328.3 (113.5—711.1)
SVAC	Female	Djibouti	0.596 (0.227—1.25)	3.78 (0.799—9.08)	545.6 (215.7—1130.9)	876.1 (197.2—2073.7)
SVAC	Female	Eritrea	8.85 (1.37—21.1)	15.2 (5.54—31.2)	879.0 (177.7—1976.9)	734.6 (273.2—1511.9)
SVAC	Female	Ethiopia	141 (36.3—280)	243 (96.7—471)	1015.0 (296.5—2129.6)	762.2 (295.2—1472.0)
SVAC	Female	Kenya	57.2 (15.3—117)	169 (43.1—330)	957.6 (268.6—1886.0)	1064.9 (239.9—2114.9)
SVAC	Female	Madagascar	20.5 (7.89—43.1)	54.6 (21.1—115)	616.0 (250.8—1305.5)	600.2 (232.3—1231.0)
SVAC	Female	Malawi	44.8 (0.270—95.5)	85.0 (11.3—205)	1661.3 (36.0—3544.2)	1521.9 (119.6—3744.5)
SVAC	Female	Mozambique	18.4 (6.11—36.2)	99.6 (1.42—260)	455.9 (152.4—940.9)	1057.5 (-3.4—2664.5)
SVAC	Female	Rwanda	20.9 (9.00—43.5)	40.1 (18.3—65.9)	1056.4 (464.6—2211.5)	923.5 (414.7—1538.4)
SVAC	Female	Somalia	10.9 (4.55—21.2)	30.9 (11.9—65.1)	530.7 (232.9—1029.8)	546.6 (215.2—1148.6)
SVAC	Female	South Sudan	9.88 (3.39—21.4)	22.8 (7.48—45.8)	647.1 (238.3—1344.6)	780.9 (257.6—1546.6)
SVAC	Female	Uganda	89.3 (-13.0—267)	96.1 (24.8—195)	1994.0 (-296.3—5948.8)	774.7 (176.2—1635.1)
SVAC	Female	United Republic of Tanzania	60.6 (8.01—137)	137 (46.4—247)	852.3 (114.4—2000.7)	717.0 (219.4—1335.9)
SVAC	Female	Zambia	46.2 (-2.91—123)	105 (0.561—255)	2110.0 (-93.3—5615.8)	1812.1 (-6.2—4308.3)
SVAC	Female	Southern Sub-Saharan Africa	151 (60.4—277)	555 (24.1—1,220)	900.3 (359.2—1732.5)	1680.6 (72.5—3670.2)
SVAC	Female	Botswana	4.97 (1.02—10.1)	16.2 (-0.0191—34.1)	1275.3 (265.3—2526.3)	1907.7 (-16.2—4009.5)
SVAC	Female	Eswatini	1.64 (0.761—2.93)	9.54 (-1.40—21.9)	648.0 (283.3—1191.7)	2470.2 (-365.0—5703.1)
SVAC	Female	Lesotho	3.51 (1.36—6.68)	17.3 (-2.13—43.2)	714.5 (281.0—1372.8)	2734.7 (-339.9—6847.9)
SVAC	Female	Namibia	2.65 (1.02—5.14)	13.1 (1.01—29.3)	637.5 (245.0—1224.3)	1335.7 (96.0—3058.7)
SVAC	Female	South Africa	82.4 (34.9—152)	417 (24.7—894)	679.5 (288.9—1247.5)	1642.5 (102.3—3495.4)
SVAC	Female	Zimbabwe	55.9 (-1.85—130)	82.0 (-2.62—186)	1925.5 (-50.1—4438.9)	1748.6 (-104.3—4066.6)
SVAC	Female	Western Sub-Saharan Africa	335 (117—656)	1,120 (419—2,130)	635.7 (226.9—1258.1)	734.4 (244.5—1430.1)
SVAC	Female	Benin	5.39 (1.83—11.4)	19.5 (7.12—38.0)	411.2 (145.8—877.0)	482.4 (176.8—979.5)
SVAC	Female	Burkina Faso	29.2 (1.86—73.0)	38.8 (15.3—73.1)	1143.4 (66.3—2883.1)	556.1 (225.3—1077.1)
SVAC	Female	Cabo Verde	0.475 (0.176—0.943)	0.848 (0.319—1.77)	455.1 (171.6—889.7)	464.5 (175.0—969.4)
SVAC	Female	Cameroon	10.5 (3.47—24.8)	57.1 (12.9—121)	365.9 (123.8—869.8)	594.7 (127.8—1254.4)
SVAC	Female	Chad	10.5 (3.61—24.6)	36.2 (9.86—84.9)	625.5 (228.3—1366.3)	747.8 (216.2—1745.3)
SVAC	Female	Côte d'Ivoire	56.6 (-1.04—132)	120 (32.6—225)	1802.8 (-40.9—4271.7)	1269.5 (291.6—2406.9)
SVAC	Female	Gambia	1.12 (0.423—2.26)	5.71 (1.50—13.7)	433.2 (159.3—848.9)	765.4 (175.5—1788.7)
SVAC	Female	Ghana	23.6 (8.78—50.0)	77.8 (22.5—163)	552.7 (210.0—1154.4)	719.4 (209.8—1498.3)
SVAC	Female	Guinea	8.69 (3.18—19.0)	28.4 (8.38—66.1)	501.9 (188.0—1105.6)	675.6 (205.9—1544.0)
SVAC	Female	Guinea-Bissau	1.42 (0.584—2.86)	5.20 (1.42—11.2)	508.7 (209.8—1064.0)	825.9 (222.6—1731.1)
SVAC	Female	Liberia	4.21 (1.36—9.14)	13.1 (3.92—29.0)	564.6 (196.8—1219.3)	708.4 (217.3—1554.0)
SVAC	Female	Mali	10.9 (4.22—22.2)	38.1 (12.2—81.4)	468.0 (180.2—969.1)	600.9 (190.3—1241.3)
SVAC	Female	Mauritania	2.86 (0.935—6.42)	7.56 (2.43—16.6)	496.4 (173.0—1076.2)	554.5 (179.0—1190.4)
SVAC	Female	Niger	9.75 (3.68—20.5)	37.1 (14.1—75.2)	475.8 (189.5—1005.1)	541.6 (221.7—1109.9)

Risk Factor	Sex	Location	Number of DALYs (thousands)		Rate of DALYs (per 100,000)	
			1990	2023	1990	2023
SVAC	Female	Nigeria	139 (58.0—258)	563 (196—999)	564.8 (231.5—1048.9)	771.4 (244.5—1377.0)
SVAC	Female	Sao Tome and Principe	0.114 (0.0417—0.223)	0.311 (0.117—0.562)	349.4 (125.4—691.0)	422.0 (160.6—749.5)
SVAC	Female	Senegal	9.10 (3.27—18.9)	28.9 (10.3—61.4)	438.2 (161.5—909.3)	519.7 (186.6—1098.8)
SVAC	Female	Sierra Leone	5.95 (2.12—12.7)	17.2 (5.57—35.7)	489.9 (177.8—1063.3)	663.3 (216.6—1339.4)
SVAC	Female	Togo	5.77 (1.99—12.5)	20.4 (6.00—42.5)	570.5 (210.4—1229.3)	735.6 (216.1—1523.1)
SVAC	Male	Global	9,520 (4,730—16,200)	15,800 (8,170—26,700)	524.0 (259.0—892.7)	520.7 (270.1—882.8)
SVAC	Male	Central Europe, Eastern Europe, and Central Asia	1,050 (415—2,000)	1,010 (422—2,020)	686.4 (271.5—1312.2)	605.5 (250.3—1217.7)
SVAC	Male	Central Asia	65.1 (25.0—134)	97.4 (36.7—205)	304.4 (116.7—626.3)	275.5 (103.3—585.0)
SVAC	Male	Armenia	2.63 (0.896—5.54)	2.47 (0.818—5.21)	225.9 (77.4—474.3)	217.9 (71.4—462.6)
SVAC	Male	Azerbaijan	5.85 (2.18—11.8)	10.4 (4.38—18.7)	254.9 (97.1—506.3)	250.9 (104.6—446.6)
SVAC	Male	Georgia	4.53 (1.62—9.23)	3.49 (1.62—5.90)	230.7 (82.6—469.5)	249.5 (111.8—428.2)
SVAC	Male	Kazakhstan	23.3 (8.81—51.3)	27.8 (10.1—59.8)	414.0 (156.1—905.5)	389.7 (141.3—844.3)
SVAC	Male	Kyrgyzstan	4.88 (1.73—10.5)	6.03 (2.16—13.1)	369.4 (132.1—797.4)	247.9 (89.1—538.8)
SVAC	Male	Mongolia	1.44 (0.473—3.54)	3.92 (1.41—8.36)	225.3 (74.0—546.7)	324.3 (114.9—699.9)
SVAC	Male	Tajikistan	3.50 (1.25—7.42)	7.58 (2.77—16.8)	242.8 (86.8—519.5)	224.2 (83.2—498.2)
SVAC	Male	Turkmenistan	2.85 (1.05—6.07)	5.72 (2.02—12.8)	269.1 (97.9—575.4)	278.2 (98.2—622.7)
SVAC	Male	Uzbekistan	16.2 (5.93—35.0)	30.0 (10.8—62.9)	273.9 (100.5—587.8)	241.1 (86.0—506.8)
SVAC	Male	Central Europe	287 (120—537)	253 (110—477)	603.2 (252.8—1127.4)	519.9 (228.3—970.1)
SVAC	Male	Albania	4.03 (1.58—7.11)	3.51 (1.46—6.59)	350.4 (137.2—627.6)	358.5 (151.7—653.1)
SVAC	Male	Bosnia and Herzegovina	12.9 (5.90—23.8)	8.31 (3.77—15.2)	745.1 (342.1—1373.8)	612.7 (286.3—1076.3)
SVAC	Male	Bulgaria	16.0 (6.15—32.4)	10.9 (4.18—20.9)	467.8 (182.9—935.9)	379.6 (144.8—722.1)
SVAC	Male	Croatia	11.3 (4.53—21.7)	8.69 (3.59—17.1)	582.7 (232.8—1118.4)	523.3 (217.7—1035.0)
SVAC	Male	Czechia	20.8 (9.65—35.2)	21.7 (9.59—34.9)	532.5 (251.7—900.6)	481.8 (212.7—770.1)
SVAC	Male	Hungary	34.8 (13.7—69.1)	21.0 (8.67—40.3)	869.3 (342.4—1714.6)	514.3 (213.4—992.4)
SVAC	Male	Montenegro	1.24 (0.474—2.36)	1.28 (0.478—2.58)	533.3 (203.1—1010.3)	475.3 (183.5—946.2)
SVAC	Male	North Macedonia	3.42 (1.36—5.97)	3.75 (1.79—6.57)	464.7 (184.2—811.3)	480.7 (238.2—810.9)
SVAC	Male	Poland	97.4 (40.1—188)	103 (44.7—198)	669.7 (276.1—1299.3)	634.6 (273.0—1226.0)
SVAC	Male	Romania	39.5 (16.5—75.1)	31.6 (12.9—60.4)	447.2 (186.9—851.1)	397.5 (165.7—745.0)
SVAC	Male	Serbia	23.5 (9.46—45.4)	19.4 (8.15—37.6)	640.6 (257.6—1242.2)	483.3 (208.5—927.2)
SVAC	Male	Slovakia	11.3 (4.51—21.8)	10.5 (4.27—19.5)	587.1 (233.1—1127.1)	461.2 (189.1—876.2)
SVAC	Male	Slovenia	5.98 (2.31—11.7)	5.36 (2.20—10.4)	781.4 (301.6—1522.2)	565.5 (228.6—1123.0)
SVAC	Male	Eastern Europe	695 (273—1,350)	662 (272—1,330)	831.7 (325.2—1609.3)	805.6 (324.2—1635.0)
SVAC	Male	Belarus	24.6 (9.77—47.5)	26.1 (11.0—52.2)	647.9 (257.0—1249.7)	692.0 (294.4—1396.3)
SVAC	Male	Estonia	4.24 (1.68—8.20)	3.58 (1.45—7.35)	745.6 (295.1—1443.2)	672.7 (260.7—1367.6)
SVAC	Male	Latvia	7.61 (2.93—14.9)	4.77 (1.88—9.46)	762.3 (293.6—1492.1)	638.1 (245.5—1264.4)
SVAC	Male	Lithuania	11.3 (4.41—22.6)	8.08 (3.22—16.5)	842.5 (329.7—1687.1)	713.2 (282.0—1453.7)
SVAC	Male	Republic of Moldova	9.39 (3.79—18.2)	8.01 (3.56—15.3)	610.8 (246.1—1187.9)	528.9 (239.8—988.9)
SVAC	Male	Russian Federation	518 (202—1,020)	500 (203—1,010)	924.4 (359.0—1808.6)	849.8 (336.8—1736.8)
SVAC	Male	Ukraine	120 (48.0—231)	111 (46.7—218)	623.0 (249.0—1201.4)	709.8 (294.5—1418.0)
SVAC	Male	High-income	2,120 (1,120—3,370)	3,240 (1,540—5,290)	605.6 (319.7—963.0)	763.9 (358.4—1267.6)
SVAC	Male	Australasia	56.2 (27.5—94.0)	99.1 (47.9—159)	721.5 (352.3—1208.8)	818.9 (399.0—1327.1)
SVAC	Male	Australia	43.7 (20.1—75.9)	81.5 (39.7—131)	672.6 (308.6—1167.8)	800.5 (386.0—1298.2)
SVAC	Male	New Zealand	12.4 (6.77—19.1)	17.6 (8.85—28.6)	960.1 (521.7—1475.9)	913.3 (451.5—1465.2)
SVAC	Male	High-income Asia Pacific	322 (131—593)	402 (149—766)	474.6 (192.4—869.8)	539.7 (204.5—1013.0)
SVAC	Male	Brunei Darussalam	0.355 (0.139—0.664)	0.777 (0.295—1.51)	402.7 (157.2—762.2)	387.6 (144.9—779.2)
SVAC	Male	Japan	223 (86.4—417)	253 (90.6—480)	446.7 (175.5—829.4)	529.1 (193.0—991.4)
SVAC	Male	Republic of Korea	93.3 (35.6—191)	138 (54.7—271)	568.4 (218.4—1156.6)	582.6 (231.8—1141.0)
SVAC	Male	Singapore	5.60 (2.12—10.4)	9.67 (3.48—18.8)	453.7 (169.1—849.5)	410.9 (156.8—773.2)
SVAC	Male	High-income North America	777 (417—1,150)	1,650 (601—2,910)	724.6 (389.0—1061.8)	1156.7 (403.9—2060.6)

Risk Factor	Sex	Location	Number of DALYs (thousands)		Rate of DALYs (per 100,000)	
			1990	2023	1990	2023
SVAC	Male	Canada	58.6 (32.3—91.3)	98.9 (39.7—178)	543.8 (300.1—850.9)	664.9 (260.6—1222.2)
SVAC	Male	Greenland	0.715 (0.231—1.52)	0.422 (0.147—0.858)	2810.7 (911.1—5919.4)	1954.6 (689.7—4044.6)
SVAC	Male	United States of America	718 (389—1,050)	1,550 (553—2,700)	744.0 (404.1—1093.0)	1212.2 (422.0—2181.2)
SVAC	Male	Southern Latin America	103 (44.6—180)	158 (66.7—298)	609.6 (264.9—1070.2)	591.8 (249.3—1122.8)
SVAC	Male	Argentina	62.3 (26.7—109)	110 (45.6—217)	557.7 (239.0—975.3)	616.7 (256.8—1217.9)
SVAC	Male	Chile	35.3 (15.5—68.3)	39.9 (16.3—70.3)	774.3 (341.4—1491.3)	523.7 (212.5—926.0)
SVAC	Male	Uruguay	4.95 (2.00—9.61)	8.02 (3.22—16.9)	445.7 (180.2—864.1)	634.2 (254.0—1334.8)
SVAC	Male	Western Europe	857 (402—1,500)	932 (451—1,660)	568.3 (267.8—997.6)	536.9 (260.3—948.7)
SVAC	Male	Andorra	0.116 (0.0453—0.217)	0.173 (0.0679—0.357)	471.0 (181.6—880.7)	436.0 (167.4—906.5)
SVAC	Male	Austria	19.4 (7.61—37.3)	19.6 (7.68—38.4)	635.2 (249.3—1212.0)	530.5 (205.3—1049.8)
SVAC	Male	Belgium	17.4 (7.29—32.5)	20.3 (7.73—36.6)	436.9 (179.3—812.5)	432.1 (164.5—764.0)
SVAC	Male	Cyprus	1.08 (0.389—1.98)	2.16 (0.783—4.48)	373.5 (135.3—684.6)	389.7 (137.5—799.1)
SVAC	Male	Denmark	16.3 (6.19—32.9)	14.3 (6.00—27.7)	786.3 (301.5—1573.9)	605.8 (267.4—1159.2)
SVAC	Male	Finland	13.7 (6.81—22.1)	11.2 (4.94—18.9)	693.1 (339.9—1105.9)	525.8 (228.1—908.6)
SVAC	Male	France	151 (61.2—287)	151 (59.1—296)	676.4 (273.8—1275.4)	565.1 (218.5—1114.8)
SVAC	Male	Germany	192 (76.8—366)	183 (74.3—372)	587.6 (236.9—1116.3)	540.1 (216.1—1085.0)
SVAC	Male	Greece	16.1 (6.09—30.0)	19.6 (7.53—38.1)	398.8 (153.0—746.6)	476.4 (181.4—943.8)
SVAC	Male	Iceland	0.450 (0.177—0.871)	0.745 (0.336—1.35)	472.2 (186.9—917.1)	467.2 (216.7—828.9)
SVAC	Male	Ireland	9.21 (4.57—14.8)	15.3 (7.20—25.2)	713.7 (356.0—1145.3)	750.8 (352.6—1231.2)
SVAC	Male	Israel	9.15 (4.09—15.6)	18.7 (8.66—33.2)	545.2 (241.6—931.9)	529.2 (246.7—940.5)
SVAC	Male	Italy	92.3 (37.3—172)	85.4 (31.3—173)	409.8 (162.7—765.7)	362.4 (137.5—734.9)
SVAC	Male	Luxembourg	1.00 (0.401—1.87)	1.30 (0.525—2.58)	647.4 (259.8—1210.2)	469.3 (189.3—925.7)
SVAC	Male	Malta	0.547 (0.209—1.03)	1.03 (0.346—2.10)	391.9 (151.8—736.2)	416.7 (138.0—850.6)
SVAC	Male	Monaco	0.0584 (0.0221—0.109)	0.0757 (0.0301—0.158)	486.7 (186.3—900.3)	524.0 (206.4—1085.8)
SVAC	Male	Netherlands	32.6 (15.0—54.8)	40.3 (17.9—68.6)	536.5 (245.3—907.9)	551.6 (243.0—966.0)
SVAC	Male	Norway	10.9 (4.78—19.6)	12.2 (5.13—21.5)	657.8 (285.8—1183.8)	547.3 (227.8—993.0)
SVAC	Male	Portugal	23.1 (9.18—44.6)	22.3 (8.82—45.9)	606.7 (241.5—1171.3)	532.2 (210.6—1091.7)
SVAC	Male	San Marino	0.0380 (0.0144—0.0707)	0.0553 (0.0210—0.115)	454.0 (173.3—848.1)	398.5 (154.4—800.7)
SVAC	Male	Spain	73.6 (35.6—116)	75.7 (35.8—134)	492.6 (237.3—772.4)	393.6 (184.3—671.9)
SVAC	Male	Sweden	17.6 (8.26—32.8)	23.3 (11.1—40.3)	519.0 (245.0—963.8)	549.4 (261.1—968.6)
SVAC	Male	Switzerland	20.8 (8.02—41.2)	18.2 (6.28—36.9)	741.5 (290.5—1472.2)	502.3 (174.6—1032.3)
SVAC	Male	United Kingdom	138 (65.7—221)	195 (93.4—338)	630.0 (299.5—1011.9)	764.3 (355.9—1324.7)
SVAC	Male	Latin America and Caribbean	592 (249—1,090)	1,210 (545—2,240)	497.1 (208.7—910.9)	544.3 (246.3—1009.1)
SVAC	Male	Andean Latin America	47.3 (18.5—87.8)	112 (46.4—226)	418.6 (163.5—766.9)	462.4 (191.3—939.5)
SVAC	Male	Bolivia (Plurinational State of)	8.27 (3.20—15.5)	20.9 (8.43—43.8)	467.3 (180.9—881.3)	489.3 (198.4—1016.7)
SVAC	Male	Ecuador	14.0 (5.68—26.0)	32.9 (13.5—66.1)	471.9 (194.9—890.7)	520.9 (214.1—1046.9)
SVAC	Male	Peru	25.1 (9.79—46.5)	58.0 (24.2—114)	381.2 (144.7—699.1)	428.3 (179.1—843.7)
SVAC	Male	Caribbean	71.6 (31.5—134)	117 (58.5—213)	624.0 (274.8—1175.3)	652.3 (327.9—1187.1)
SVAC	Male	Antigua and Barbuda	0.0810 (0.0316—0.160)	0.168 (0.0669—0.333)	427.8 (170.4—848.2)	452.1 (178.5—902.2)
SVAC	Male	Bahamas	0.388 (0.161—0.774)	0.776 (0.320—1.60)	467.0 (190.4—935.9)	479.7 (197.3—985.9)
SVAC	Male	Barbados	0.397 (0.161—0.782)	0.556 (0.223—1.16)	446.9 (180.7—879.5)	448.0 (175.9—935.5)
SVAC	Male	Belize	0.201 (0.106—0.337)	0.705 (0.350—1.11)	390.0 (203.5—662.7)	496.7 (246.6—780.5)
SVAC	Male	Bermuda	0.107 (0.0432—0.212)	0.106 (0.0427—0.217)	449.8 (182.3—895.0)	415.0 (166.9—843.0)
SVAC	Male	Cuba	21.9 (8.70—42.7)	26.1 (10.3—52.4)	528.6 (209.0—1039.0)	531.1 (209.5—1063.7)
SVAC	Male	Dominica	0.0933 (0.0367—0.184)	0.134 (0.0542—0.269)	402.1 (158.6—803.2)	462.2 (188.8—935.1)
SVAC	Male	Dominican Republic	9.02 (3.53—17.2)	21.6 (9.15—42.7)	422.8 (165.1—816.5)	533.4 (225.6—1058.8)
SVAC	Male	Grenada	0.123 (0.0480—0.233)	0.221 (0.0870—0.453)	423.9 (165.8—803.1)	473.7 (186.3—966.1)
SVAC	Male	Guyana	1.72 (0.667—3.48)	2.58 (0.990—5.12)	729.2 (282.2—1490.4)	874.5 (334.6—1742.4)
SVAC	Male	Haiti	19.2 (8.04—36.8)	39.9 (21.6—65.3)	1114.9 (463.6—2156.8)	940.4 (507.4—1594.4)
SVAC	Male	Jamaica	3.07 (1.38—5.67)	5.51 (2.45—9.52)	433.6 (190.2—814.6)	497.5 (222.3—859.8)

Risk Factor	Sex	Location	Number of DALYs (thousands)		Rate of DALYs (per 100,000)	
			1990	2023	1990	2023
SVAC	Male	Puerto Rico	8.80 (3.70—17.2)	8.67 (3.09—18.1)	713.4 (300.5—1394.9)	717.4 (243.4—1509.5)
SVAC	Male	Saint Kitts and Nevis	0.0645 (0.0264—0.127)	0.110 (0.0460—0.217)	538.3 (220.4—1084.8)	563.6 (235.2—1127.2)
SVAC	Male	Saint Lucia	0.194 (0.0763—0.376)	0.363 (0.144—0.755)	500.3 (197.1—981.5)	484.9 (190.3—1005.1)
SVAC	Male	Saint Vincent and the Grenadines	0.157 (0.0646—0.304)	0.245 (0.107—0.477)	494.5 (204.4—959.6)	516.8 (226.0—1007.1)
SVAC	Male	Suriname	0.859 (0.324—1.85)	1.85 (0.716—3.59)	663.9 (249.2—1444.1)	846.8 (327.1—1654.2)
SVAC	Male	Trinidad and Tobago	2.66 (1.02—5.38)	3.69 (1.43—7.76)	674.9 (249.4—1384.8)	638.9 (248.4—1315.1)
SVAC	Male	United States Virgin Islands	0.194 (0.0785—0.366)	0.210 (0.0859—0.425)	539.8 (219.2—1028.5)	668.9 (279.1—1328.4)
SVAC	Male	Central Latin America	238 (106—443)	509 (222—957)	506.3 (221.4—976.2)	533.7 (232.2—1004.6)
SVAC	Male	Colombia	45.1 (19.1—83.1)	96.4 (44.3—174)	435.1 (183.6—805.9)	483.4 (222.2—872.5)
SVAC	Male	Costa Rica	5.56 (2.65—9.65)	14.1 (6.05—26.0)	581.6 (271.8—1022.6)	671.9 (289.3—1243.7)
SVAC	Male	El Salvador	11.5 (4.47—23.8)	13.1 (6.32—23.4)	799.0 (312.4—1685.8)	642.6 (304.9—1164.6)
SVAC	Male	Guatemala	15.1 (6.34—29.9)	36.2 (15.1—68.1)	741.6 (312.6—1482.8)	688.8 (285.4—1290.8)
SVAC	Male	Honduras	6.62 (2.90—11.8)	17.4 (8.98—28.7)	546.1 (243.9—983.6)	512.3 (256.4—891.4)
SVAC	Male	Mexico	118 (54.4—221)	264 (118—503)	502.3 (222.3—977.8)	538.2 (240.4—1028.7)
SVAC	Male	Nicaragua	5.35 (2.28—10.3)	12.8 (5.69—23.4)	552.3 (235.5—1070.2)	541.9 (233.9—988.3)
SVAC	Male	Panama	3.45 (1.44—6.62)	7.70 (3.48—15.3)	434.4 (182.1—843.6)	502.7 (227.0—999.5)
SVAC	Male	Venezuela (Bolivarian Republic of)	27.7 (11.7—51.9)	48.0 (18.8—92.6)	479.3 (199.2—915.8)	512.3 (200.1—984.8)
SVAC	Male	Tropical Latin America	234 (93.3—435)	468 (207—817)	477.1 (189.4—895.3)	559.6 (249.2—974.7)
SVAC	Male	Brazil	230 (91.9—425)	454 (201—797)	479.0 (190.7—897.6)	558.9 (249.7—977.2)
SVAC	Male	Paraguay	4.76 (1.75—9.11)	13.8 (5.50—27.4)	403.0 (149.3—769.8)	597.3 (236.2—1194.6)
SVAC	Male	North Africa and Middle East	394 (143—732)	1,000 (367—2,110)	383.1 (138.6—701.4)	416.3 (151.7—879.8)
SVAC	Male	Afghanistan	8.78 (3.28—16.7)	35.3 (12.8—73.6)	325.0 (116.7—614.9)	357.2 (126.9—771.7)
SVAC	Male	Algeria	28.7 (10.4—54.9)	68.4 (23.5—146)	384.2 (139.8—727.7)	421.2 (147.3—904.2)
SVAC	Male	Bahrain	0.951 (0.367—1.77)	3.82 (1.43—7.88)	465.8 (180.4—895.0)	468.0 (169.0—983.9)
SVAC	Male	Egypt	54.7 (19.3—104)	133 (46.8—274)	324.6 (114.9—623.3)	356.6 (125.8—743.9)
SVAC	Male	Iran (Islamic Republic of)	83.5 (26.1—162)	190 (64.7—408)	500.9 (152.6—969.8)	541.8 (182.1—1172.4)
SVAC	Male	Iraq	22.1 (8.34—41.3)	76.0 (27.9—158)	422.5 (165.8—779.2)	495.6 (186.7—1023.9)
SVAC	Male	Jordan	3.78 (1.34—7.11)	17.9 (6.25—36.6)	350.5 (123.6—651.8)	345.7 (120.9—708.1)
SVAC	Male	Kuwait	2.40 (0.876—4.71)	9.90 (3.57—19.8)	347.4 (134.0—665.1)	385.9 (135.6—792.2)
SVAC	Male	Lebanon	3.43 (1.23—6.38)	8.19 (2.88—16.7)	338.3 (121.5—633.6)	383.3 (133.9—778.0)
SVAC	Male	Libya	5.18 (1.86—9.56)	13.5 (4.89—28.5)	387.0 (137.7—720.8)	456.2 (164.4—972.6)
SVAC	Male	Morocco	28.8 (10.7—53.2)	57.9 (20.5—123)	370.3 (139.1—684.6)	404.1 (143.3—859.4)
SVAC	Male	Oman	2.43 (0.867—4.68)	8.82 (3.12—17.8)	333.7 (121.3—625.9)	356.0 (130.5—755.4)
SVAC	Male	Palestine	1.80 (0.590—3.44)	6.41 (2.21—13.0)	349.2 (118.2—657.6)	380.3 (135.8—797.4)
SVAC	Male	Qatar	0.898 (0.330—1.67)	7.02 (2.48—13.8)	399.0 (150.9—745.2)	365.9 (125.0—741.1)
SVAC	Male	Saudi Arabia	24.8 (9.58—46.1)	70.9 (26.8—147)	434.4 (166.6—804.9)	438.1 (165.7—953.7)
SVAC	Male	Sudan	20.7 (8.09—38.9)	53.6 (18.9—112)	378.4 (146.8—727.9)	421.9 (149.1—899.1)
SVAC	Male	Syrian Arab Republic	13.3 (4.74—26.5)	26.5 (9.42—55.5)	390.0 (138.9—743.3)	419.6 (149.7—873.7)
SVAC	Male	Tunisia	10.1 (3.89—18.7)	19.7 (7.18—41.9)	392.4 (152.8—717.5)	422.9 (152.3—897.4)
SVAC	Male	Türkiye	61.8 (22.4—114)	127 (46.7—258)	332.6 (121.8—599.6)	377.0 (138.8—767.9)
SVAC	Male	United Arab Emirates	3.67 (1.32—7.12)	27.4 (9.60—54.2)	419.5 (162.7—783.8)	406.3 (148.0—837.4)
SVAC	Male	Yemen	12.1 (4.49—23.4)	42.3 (15.3—87.9)	371.4 (140.6—715.2)	395.3 (144.6—846.7)
SVAC	Male	South Asia	1,670 (671—3,290)	3,590 (1,680—6,370)	493.4 (196.7—963.5)	517.5 (242.2—941.8)
SVAC	Male	Bangladesh	239 (111—406)	408 (194—664)	789.1 (367.9—1335.6)	679.0 (317.4—1110.7)
SVAC	Male	Bhutan	0.926 (0.362—1.74)	1.68 (0.716—3.13)	517.1 (197.9—933.7)	558.3 (236.2—1055.1)
SVAC	Male	India	1,230 (482—2,500)	2,660 (1,180—4,760)	453.0 (176.2—918.3)	486.7 (216.1—882.8)
SVAC	Male	Nepal	33.6 (13.4—64.3)	72.3 (29.0—137)	614.4 (242.7—1170.9)	671.8 (269.6—1281.4)
SVAC	Male	Pakistan	170 (67.0—323)	443 (181—811)	530.3 (211.4—975.1)	584.7 (235.6—1079.6)
SVAC	Male	Southeast Asia, East Asia, and Oceania	2,650 (1,300—4,470)	2,930 (1,430—4,610)	420.3 (203.1—721.3)	323.3 (159.3—496.1)

Risk Factor	Sex	Location	Number of DALYs (thousands)		Rate of DALYs (per 100,000)	
			1990	2023	1990	2023
SVAC	Male	East Asia	2,090 (1,010—3,440)	1,870 (908—3,080)	433.8 (215.3—723.6)	299.6 (143.3—488.9)
SVAC	Male	China	2,040 (987—3,350)	1,790 (842—2,970)	436.8 (216.4—727.2)	295.8 (141.3—493.7)
SVAC	Male	Democratic People's Republic of Korea	25.3 (9.63—49.3)	37.5 (14.0—72.4)	344.4 (131.6—667.0)	348.6 (132.8—679.1)
SVAC	Male	Taiwan	26.0 (10.7—51.0)	47.0 (18.0—93.2)	335.3 (137.2—659.6)	464.4 (180.1—915.4)
SVAC	Male	Oceania	7.93 (3.29—15.1)	20.2 (8.39—39.0)	425.5 (170.5—834.7)	442.7 (176.4—869.5)
SVAC	Male	American Samoa	0.0663 (0.0258—0.135)	0.0874 (0.0311—0.196)	460.1 (178.0—981.8)	479.7 (171.0—1083.9)
SVAC	Male	Cook Islands	0.0432 (0.0139—0.0905)	0.0334 (0.0126—0.0732)	668.5 (216.2—1397.0)	635.6 (243.6—1391.3)
SVAC	Male	Fiji	1.29 (0.478—2.66)	2.01 (0.693—4.62)	590.9 (216.5—1286.0)	610.4 (209.8—1419.0)
SVAC	Male	Guam	0.299 (0.110—0.596)	0.423 (0.159—0.881)	538.2 (195.6—1078.7)	708.1 (265.3—1478.9)
SVAC	Male	Kiribati	0.160 (0.0611—0.305)	0.286 (0.101—0.610)	694.2 (266.4—1315.8)	673.0 (242.8—1456.9)
SVAC	Male	Marshall Islands	0.0769 (0.0285—0.154)	0.0884 (0.0318—0.195)	625.6 (232.1—1256.4)	620.6 (219.8—1383.0)
SVAC	Male	Micronesia (Federated States of)	0.206 (0.0698—0.409)	0.270 (0.0957—0.610)	714.7 (236.0—1462.7)	745.0 (258.1—1712.2)
SVAC	Male	Nauru	0.0226 (0.00837—0.0446)	0.0298 (0.0112—0.0635)	747.3 (273.4—1451.6)	751.7 (277.1—1666.7)
SVAC	Male	Niue	0.00450 (0.00164—0.00885)	0.00364 (0.00131—0.00793)	601.7 (221.0—1197.6)	589.9 (218.1—1273.4)
SVAC	Male	Northern Mariana Islands	0.121 (0.0450—0.247)	0.138 (0.0523—0.297)	664.9 (244.7—1392.9)	715.5 (272.4—1550.9)
SVAC	Male	Palau	0.0273 (0.0105—0.0538)	0.0409 (0.0155—0.0898)	495.5 (190.4—971.9)	501.8 (192.5—1112.0)
SVAC	Male	Papua New Guinea	3.97 (1.58—7.46)	13.3 (5.49—25.7)	352.2 (141.3—671.4)	396.6 (159.1—772.6)
SVAC	Male	Samoa	0.267 (0.104—0.521)	0.347 (0.123—0.776)	507.3 (194.0—1023.0)	514.7 (181.7—1152.9)
SVAC	Male	Solomon Islands	0.543 (0.198—1.10)	1.65 (0.603—3.49)	587.7 (209.5—1231.1)	611.9 (221.4—1315.2)
SVAC	Male	Tokelau	0.00241 (0.000928—0.00474)	0.00276 (0.000994—0.00604)	481.8 (184.0—954.8)	458.2 (164.6—996.7)
SVAC	Male	Tonga	0.0986 (0.0396—0.198)	0.128 (0.0476—0.271)	364.6 (146.2—750.1)	406.1 (151.4—848.7)
SVAC	Male	Tuvalu	0.0160 (0.00629—0.0308)	0.0235 (0.00882—0.0512)	572.3 (222.8—1109.5)	612.0 (231.8—1330.4)
SVAC	Male	Vanuatu	0.221 (0.0880—0.419)	0.549 (0.203—1.16)	507.8 (201.0—986.2)	523.1 (191.9—1095.6)
SVAC	Male	Southeast Asia	545 (236—1,020)	1,040 (478—1,920)	383.6 (166.7—726.6)	379.0 (173.0—700.0)
SVAC	Male	Cambodia	10.0 (4.87—16.9)	25.0 (11.4—41.7)	414.3 (196.3—710.0)	422.6 (194.2—720.9)
SVAC	Male	Indonesia	116 (38.7—239)	259 (87.7—492)	208.3 (73.6—448.7)	228.9 (76.9—446.9)
SVAC	Male	Lao People's Democratic Republic	5.04 (2.28—8.76)	12.7 (5.91—21.8)	456.4 (201.3—796.0)	478.5 (222.3—846.8)
SVAC	Male	Malaysia	16.1 (5.71—31.0)	42.5 (16.5—81.7)	295.2 (107.0—569.9)	300.2 (117.0—581.7)
SVAC	Male	Maldives	0.185 (0.0713—0.355)	0.822 (0.296—1.59)	314.2 (120.3—608.7)	292.8 (108.1—559.2)
SVAC	Male	Mauritius	2.87 (1.20—5.69)	3.96 (1.59—8.14)	792.4 (328.8—1600.5)	713.1 (293.1—1429.2)
SVAC	Male	Myanmar	83.2 (33.6—172)	143 (60.4—261)	705.5 (285.5—1469.9)	727.9 (305.2—1329.2)
SVAC	Male	Philippines	68.5 (27.9—130)	159 (66.8—295)	366.2 (150.7—691.8)	391.1 (163.1—729.3)
SVAC	Male	Seychelles	0.106 (0.0441—0.210)	0.207 (0.0837—0.410)	445.1 (183.7—893.4)	429.2 (173.9—845.8)
SVAC	Male	Sri Lanka	67.9 (27.7—127)	53.5 (22.0—100)	1143.3 (463.6—2139.7)	624.8 (257.0—1170.8)
SVAC	Male	Thailand	98.4 (41.7—198)	155 (66.8—297)	483.9 (206.1—965.0)	598.2 (260.2—1159.2)
SVAC	Male	Timor-Leste	0.772 (0.309—1.42)	1.67 (0.707—3.22)	334.2 (136.8—638.4)	361.6 (155.0—699.9)
SVAC	Male	Viet Nam	75.7 (31.6—143)	183 (81.5—347)	393.3 (166.4—744.4)	460.7 (204.5—878.0)
SVAC	Male	Sub-Saharan Africa	1,050 (464—2,130)	2,780 (1,160—4,860)	844.3 (368.1—1737.0)	882.1 (349.0—1577.1)
SVAC	Male	Central Sub-Saharan Africa	113 (48.4—224)	297 (124—582)	814.2 (349.8—1606.9)	764.1 (309.1—1503.1)
SVAC	Male	Angola	20.1 (7.91—38.2)	77.9 (32.9—155)	739.0 (299.0—1375.3)	886.7 (363.0—1786.9)
SVAC	Male	Central African Republic	7.17 (2.61—15.3)	16.1 (5.76—33.4)	1038.1 (370.1—2268.2)	995.1 (344.0—2094.3)
SVAC	Male	Congo	6.57 (2.58—14.3)	17.1 (5.65—36.9)	1096.5 (426.6—2405.4)	949.3 (320.0—2044.7)
SVAC	Male	Democratic Republic of the Congo	75.9 (32.8—154)	174 (69.4—355)	802.0 (340.6—1626.0)	677.4 (268.6—1384.3)
SVAC	Male	Equatorial Guinea	0.857 (0.340—1.77)	6.57 (1.65—14.8)	866.4 (343.4—1781.3)	1399.8 (317.8—3177.6)
SVAC	Male	Gabon	2.10 (0.879—4.14)	5.30 (2.11—10.5)	783.7 (328.8—1564.0)	929.0 (363.6—1845.5)
SVAC	Male	Eastern Sub-Saharan Africa	462 (164—941)	971 (435—1,670)	1009.5 (343.9—2089.7)	820.6 (334.5—1459.9)
SVAC	Male	Burundi	14.1 (4.10—35.4)	23.4 (9.35—45.4)	1027.0 (298.5—2619.9)	602.9 (238.0—1179.4)
SVAC	Male	Comoros	0.491 (0.194—0.903)	1.10 (0.426—2.29)	414.5 (166.5—786.1)	398.4 (155.6—829.5)
SVAC	Male	Djibouti	0.544 (0.226—1.05)	3.01 (1.15—5.93)	477.8 (199.7—923.7)	618.1 (241.7—1227.7)

Risk Factor	Sex	Location	Number of DALYs (thousands)		Rate of DALYs (per 100,000)	
			1990	2023	1990	2023
SVAC	Male	Eritrea	6.03 (2.36—11.7)	15.4 (5.91—30.0)	703.5 (284.1—1335.5)	689.3 (270.8—1351.9)
SVAC	Male	Ethiopia	60.4 (31.2—109)	137 (71.0—213)	475.5 (239.1—878.4)	418.4 (222.1—667.3)
SVAC	Male	Kenya	48.2 (19.1—97.0)	149 (59.8—261)	906.4 (356.4—1854.6)	1006.2 (381.7—1802.1)
SVAC	Male	Madagascar	18.2 (7.17—35.8)	48.3 (19.5—99.5)	599.9 (242.1—1174.0)	573.1 (221.6—1184.6)
SVAC	Male	Malawi	33.7 (9.25—72.9)	72.6 (24.9—136)	1431.9 (376.5—3136.1)	1461.6 (364.6—2849.7)
SVAC	Male	Mozambique	17.4 (7.24—32.3)	99.3 (13.4—255)	532.0 (219.1—996.5)	1297.2 (165.1—3415.3)
SVAC	Male	Rwanda	19.2 (9.31—38.8)	31.9 (14.1—52.8)	1176.3 (576.5—2405.8)	856.3 (380.3—1439.7)
SVAC	Male	Somalia	9.56 (3.69—18.3)	26.7 (10.6—54.1)	468.7 (177.1—907.2)	478.6 (191.8—980.1)
SVAC	Male	South Sudan	9.44 (3.82—18.6)	18.7 (7.01—37.1)	588.2 (245.2—1193.6)	699.6 (262.7—1387.2)
SVAC	Male	Uganda	120 (-12.6—319)	124 (44.1—225)	3193.0 (-422.9—8688.5)	1188.4 (329.7—2236.2)
SVAC	Male	United Republic of Tanzania	76.0 (23.7—161)	150 (67.4—257)	1267.1 (370.8—2773.8)	904.9 (374.1—1639.4)
SVAC	Male	Zambia	28.3 (6.82—64.3)	69.2 (19.7—135)	1550.2 (348.4—3754.2)	1379.4 (322.5—2750.4)
SVAC	Male	Southern Sub-Saharan Africa	130 (60.9—237)	485 (73.2—1,100)	873.9 (410.7—1589.0)	1614.9 (217.7—3623.8)
SVAC	Male	Botswana	5.02 (1.80—9.64)	15.6 (1.76—32.7)	1613.8 (582.3—2966.6)	2077.0 (171.7—4351.5)
SVAC	Male	Eswatini	1.07 (0.415—2.19)	4.89 (0.571—10.7)	625.6 (235.6—1279.4)	1436.2 (80.3—3263.5)
SVAC	Male	Lesotho	2.54 (1.02—5.25)	10.4 (1.39—24.6)	750.8 (290.8—1571.3)	1804.1 (175.5—4169.2)
SVAC	Male	Namibia	2.80 (1.25—5.33)	10.9 (3.22—23.9)	749.8 (331.9—1431.2)	1193.2 (338.6—2631.2)
SVAC	Male	South Africa	102 (44.5—197)	420 (66.0—961)	903.6 (391.4—1735.6)	1801.1 (261.1—4099.0)
SVAC	Male	Zimbabwe	16.7 (2.30—41.9)	23.3 (5.53—54.8)	712.0 (81.2—1875.4)	575.4 (126.6—1358.8)
SVAC	Male	Western Sub-Saharan Africa	349 (170—623)	1,020 (472—1,730)	696.4 (341.4—1282.3)	788.5 (347.6—1321.2)
SVAC	Male	Benin	6.32 (2.85—11.1)	24.0 (10.3—47.6)	604.2 (285.5—1058.1)	683.7 (302.5—1308.5)
SVAC	Male	Burkina Faso	18.2 (4.17—46.6)	25.5 (10.6—53.3)	920.5 (193.4—2451.2)	447.4 (185.5—936.1)
SVAC	Male	Cabo Verde	0.561 (0.246—1.03)	1.28 (0.582—2.37)	707.0 (314.9—1296.4)	699.3 (313.1—1298.1)
SVAC	Male	Cameroon	18.9 (8.77—35.3)	90.8 (35.2—166)	752.5 (356.7—1430.1)	1027.2 (364.5—1945.0)
SVAC	Male	Chad	10.3 (4.53—18.6)	35.8 (15.4—64.9)	746.7 (327.2—1336.5)	848.6 (367.6—1601.9)
SVAC	Male	Côte d'Ivoire	54.1 (9.39—116)	124 (51.2—214)	1681.7 (288.1—3591.7)	1190.3 (475.4—2088.2)
SVAC	Male	Gambia	1.33 (0.593—2.37)	5.66 (1.91—11.5)	532.8 (239.6—946.0)	844.6 (271.5—1744.6)
SVAC	Male	Ghana	24.4 (11.2—44.7)	76.9 (35.9—141)	645.3 (301.3—1197.4)	791.8 (359.9—1434.0)
SVAC	Male	Guinea	8.47 (3.66—15.1)	25.9 (10.7—51.7)	569.0 (251.5—1015.4)	750.0 (310.6—1475.0)
SVAC	Male	Guinea-Bissau	1.56 (0.712—2.89)	5.10 (1.99—9.61)	669.9 (307.3—1265.5)	954.8 (322.1—1840.1)
SVAC	Male	Liberia	4.49 (2.01—8.04)	14.1 (5.76—25.9)	635.5 (290.8—1142.4)	768.2 (313.0—1434.6)
SVAC	Male	Mali	11.5 (5.12—20.7)	37.9 (17.2—71.5)	551.2 (252.5—999.9)	639.5 (282.5—1208.8)
SVAC	Male	Mauritania	3.01 (1.35—5.54)	7.65 (3.20—13.8)	578.3 (253.6—1076.3)	627.7 (255.0—1168.4)
SVAC	Male	Niger	8.53 (3.64—15.7)	30.0 (13.0—57.2)	469.2 (196.1—865.8)	509.6 (215.3—949.7)
SVAC	Male	Nigeria	154 (80.5—249)	450 (186—748)	613.3 (322.1—1003.7)	778.7 (311.6—1286.0)
SVAC	Male	Sao Tome and Principe	0.0929 (0.0371—0.181)	0.247 (0.107—0.430)	308.7 (123.9—596.4)	340.5 (147.4—586.4)
SVAC	Male	Senegal	10.2 (4.48—18.2)	30.6 (13.1—59.8)	561.7 (251.3—1016.0)	603.2 (258.4—1198.0)
SVAC	Male	Sierra Leone	6.98 (3.03—13.0)	18.4 (8.26—34.6)	643.2 (281.3—1195.9)	778.8 (339.9—1478.9)
SVAC	Male	Togo	5.54 (2.47—10.0)	19.1 (7.82—36.8)	672.3 (310.1—1229.5)	804.0 (334.0—1565.7)

Section 2: Prevalence of IPV and SVAC

Table S3: Global prevalence of IPV and SVAC by sex and age group

Age group	Intimate Partner Violence	Sexual Violence against Children	
	Females	Females	Males
15 to 19	6.78% (3.60 - 14.11)	18.76% (12.45 - 29.55)	13.78% (8.65 - 23.20)
20 to 24	15.91% (12.13 - 22.10)	18.61% (16.44 - 23.36)	13.06% (7.78 - 21.74)
25 to 29	23.85% (19.64 - 30.23)	19.58% (17.21 - 24.68)	13.42% (8.21 - 22.22)
30 to 34	27.47% (22.45 - 34.21)	19.98% (16.96 - 25.59)	16.10% (11.27 - 23.90)
35 to 39	28.12% (22.84 - 35.63)	21.68% (18.50 - 27.49)	16.17% (11.07 - 24.58)
40 to 44	27.93% (22.86 - 35.15)	21.65% (19.33 - 25.34)	18.67% (11.24 - 29.24)
45 to 49	27.51% (21.53 - 35.88)	19.83% (17.57 - 23.29)	17.32% (12.48 - 25.26)
50 to 54	24.95% (18.70 - 32.12)	18.92% (13.56 - 27.94)	15.93% (10.01 - 25.39)
55 to 59	21.98% (15.15 - 30.97)	17.72% (12.76 - 25.62)	14.80% (9.35 - 23.28)
60 to 64	15.84% (10.63 - 23.60)	18.11% (12.76 - 27.22)	14.29% (9.10 - 22.97)
65 to 69	11.72% (5.06 - 27.35)	16.59% (10.75 - 26.36)	13.22% (6.93 - 24.27)
70 to 74	10.57% (4.43 - 24.96)	14.92% (8.46 - 26.11)	11.00% (5.86 - 20.24)
75 to 79	8.17% (3.21 - 21.29)	14.26% (7.10 - 26.24)	9.96% (4.98 - 18.61)
80 to 84	5.57% (1.99 - 15.56)	10.62% (5.24 - 20.46)	7.39% (3.44 - 14.96)
85 to 89	5.45% (1.32 - 16.05)	9.05% (3.99 - 19.03)	6.69% (3.14 - 13.60)
90 to 94	5.37% (1.35 - 15.48)	7.93% (3.89 - 16.62)	6.00% (3.18 - 12.04)
95 plus	5.32% (1.35 - 14.86)	8.03% (4.22 - 16.39)	6.14% (3.47 - 11.93)

Figure S1: Age-standardised prevalence of SVAC among males and females 15 years and older in 2023

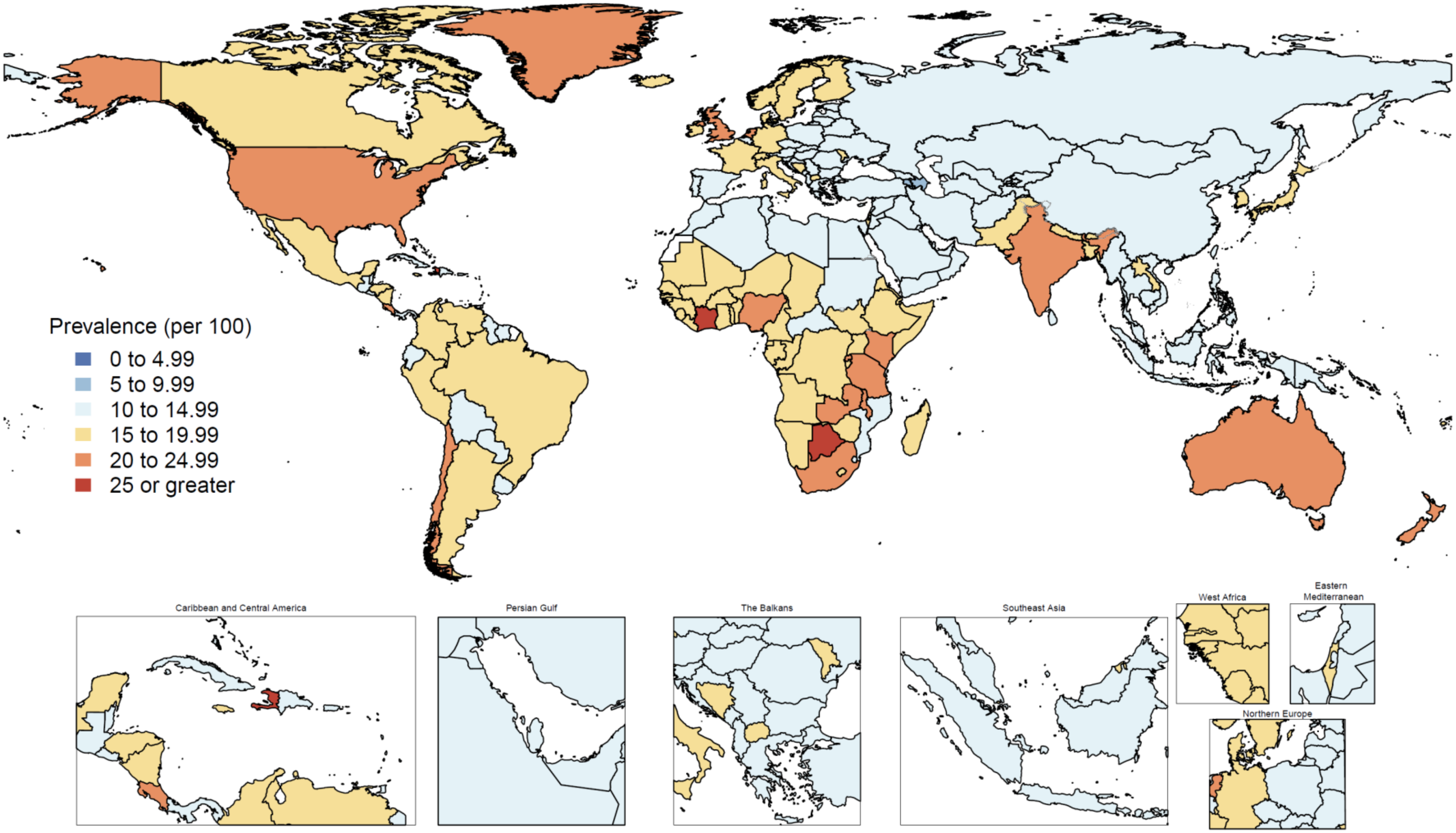

Table S4: Age-standardised prevalence of IPV and SVAC among individuals aged 15 years and older, by sex, in 2023

Location name	Intimate Partner Violence	Sexual Violence against Children		
	Females, 15+	Females, 15+	Males, 15+	Both sexes, 15+
**Global**	**20.16% (17.19–23.96)**	**18.91% (15.64–25.67)**	**14.67% (9.46–23.43)**	**16.79% (12.72–24.43)**
**Central Europe, Eastern Europe, and Central Asia**	**12.48% (10.05–16.79)**	**13.67% (7.65–25.82)**	**12.07% (5.31–24.1)**	**12.88% (6.51–24.92)**
Albania	14.27% (8.57–22.11)	9.13% (6.84–11.81)	16.92% (8.93–30.87)	12.98% (9.55–19.17)
Armenia	5.9% (3.87–8.94)	11.66% (7.66–19.05)	7% (2.68–15.31)	9.48% (6.65–13.4)
Azerbaijan	8.83% (5.91–13)	9.74% (4.86–17.54)	8.39% (4.65–14.3)	9.07% (4.8–15.85)
Belarus	9.73% (3.32–21.95)	15.35% (5.44–33.38)	12.95% (5.24–26.59)	14.2% (5.34–30.15)
Bosnia and Herzegovina	7.38% (5.55–10.45)	14.9% (7.09–29.69)	20.85% (11.49–36.49)	17.85% (9.27–33.06)
Bulgaria	16.7% (12.57–21.93)	10.24% (6.41–16.03)	13.51% (5.8–26.99)	11.87% (9.26–17.22)
Croatia	9.57% (7.03–15.09)	8.97% (4.81–15.42)	13.27% (6.61–25.27)	11.09% (8.62–15.34)
Czechia	11.8% (8.57–15.94)	14.31% (10.6–19.54)	13.85% (8.18–21.19)	14.1% (11.34–17.72)
Estonia	14.3% (8.93–21.89)	16.75% (11.6–25.55)	12.95% (5.24–26.59)	14.86% (11.69–20.19)
Georgia	5.49% (3.96–7.56)	15.18% (11.86–19.36)	6.95% (4.18–11.4)	11.07% (8.41–14.66)
Hungary	14.86% (11.96–20.58)	11.43% (7.74–18.05)	14.58% (6–29.43)	12.99% (9.83–19.39)
Kazakhstan	12.72% (9.69–16.38)	13.74% (4.79–30.48)	7.2% (2.84–15.55)	10.58% (3.84–23.29)
Kyrgyzstan	18.5% (13.32–26.27)	13.74% (4.79–30.48)	7% (2.68–15.31)	10.48% (3.77–23.14)
Latvia	22.6% (16.87–30.06)	13.62% (9.33–21.22)	12.95% (5.24–26.59)	13.29% (10.34–18.8)
Lithuania	19.94% (12.45–30.14)	12.44% (8.46–19.52)	12.95% (5.24–26.59)	12.68% (9.77–18.28)
Mongolia	25.36% (21.52–31)	15.66% (10.02–23.5)	4.17% (1.62–9.12)	10.16% (6.42–15.39)
Montenegro	15.02% (6.77–29.13)	7.01% (5.12–9.45)	14.58% (6–29.43)	10.81% (6.32–18.7)
North Macedonia	14.62% (3.39–37.66)	12.5% (8.22–20.71)	19.02% (13.07–29.1)	15.77% (10.66–24.83)
Poland	9.44% (4.54–20.37)	10.37% (6.95–16.77)	14.58% (6–29.43)	12.47% (8.45–19.33)
Republic of Moldova	22.77% (19.84–26.29)	22.21% (11.86–39.38)	13.48% (6.84–24.95)	17.95% (9.36–32.53)
Romania	15.74% (10.51–22.86)	7.71% (4.1–13.25)	13.65% (6.85–25.87)	10.66% (8.12–15.38)
Russian Federation	12.17% (8.11–18.94)	15.35% (5.42–33.38)	12.95% (5.24–26.59)	14.2% (5.33–30.13)
Serbia	10.39% (5.41–18.16)	12.97% (5.94–26.63)	15.13% (7.74–28.18)	14.07% (6.93–27.43)

Slovakia	16.8% (11.18–25.3)	10.34% (6.96–16.41)	14.58% (6–29.43)	12.46% (9.26–19.06)
Slovenia	9.48% (6.95–14.88)	12.34% (8.39–19.39)	14.58% (6–29.43)	13.5% (10.2–20.23)
Tajikistan	19.5% (14.5–25.95)	13.74% (4.79–30.48)	7% (2.68–15.31)	10.35% (3.73–22.85)
Turkmenistan	10.37% (6.13–16.64)	13.74% (4.79–30.48)	7% (2.68–15.31)	10.31% (3.72–22.76)
Ukraine	13.02% (8.39–18.91)	15.35% (5.44–33.38)	12.95% (5.24–26.59)	14.19% (5.34–30.12)
Uzbekistan	9.85% (2.31–26.88)	13.74% (4.79–30.48)	7% (2.68–15.31)	10.43% (3.76–23.02)
**High-income**	**18.67% (16.21–23.29)**	**23.57% (21.67–27.09)**	**14.77% (10.47–22.6)**	**19.17% (16.45–24.81)**
Andorra	14.24% (3.61–35.98)	20.26% (7.58–41.64)	13.58% (5.53–27.69)	16.74% (6.5–34.29)
Argentina	25.06% (18.52–32.28)	18.33% (7.07–37.53)	16.69% (7.19–32.54)	17.52% (7.15–35.05)
Australia	17.15% (12.75–23.51)	27.34% (22.21–33.87)	16.21% (11.77–21.9)	21.84% (18.05–26.35)
Austria	9.16% (6–15.69)	12.08% (8.3–18.66)	13.58% (5.53–27.69)	12.83% (9.77–18.97)
Belgium	13.38% (10.01–17.51)	19.48% (15.17–25.02)	9.59% (4.15–17.58)	14.52% (11.9–17.85)
Brunei Darussalam	15.16% (3.38–39.5)	23.53% (9.09–46.56)	14.89% (6.14–29.94)	18.95% (7.53–37.78)
Canada	6.52% (2.83–13.45)	24.18% (17.47–32.32)	12.01% (9.26–16.04)	18.09% (14.62–22.79)
Chile	16.79% (13.63–22.04)	31.74% (15.12–54)	14.2% (6.35–25.18)	23.02% (12.03–38.99)
Cyprus	9.24% (5.04–17.57)	10.98% (7.53–16.91)	13.58% (5.53–27.69)	12.26% (9.27–18.34)
Denmark	21.36% (13.22–33.82)	20.85% (13.74–29.65)	15.8% (7.25–30.54)	18.3% (11.87–28.18)
Finland	20.6% (15.24–26.88)	20.9% (13.65–31.06)	10.58% (6.23–17.39)	15.69% (11.58–22.19)
France	13.02% (9.37–17.58)	25.72% (18.37–36.54)	13.58% (5.53–27.69)	19.75% (16.14–24.84)
Germany	16.05% (8.94–27.32)	19.75% (13.67–29.48)	13.58% (5.53–27.69)	16.63% (13.23–22.04)
Greece	12.78% (9.15–17.37)	12.55% (6.87–21.01)	15.13% (6.49–29.68)	13.83% (11.06–18.65)
Greenland	19.47% (5.32–45.38)	27.72% (12.68–50.86)	16.05% (6.43–33.08)	21.58% (9.21–41.25)
Iceland	18.5% (12.35–30.34)	22.48% (14.77–34.85)	10.55% (6.69–17.18)	16.26% (10.64–25.55)
Ireland	8.93% (4.85–14.89)	17.93% (11.59–27.92)	20.47% (13.9–29.42)	19.17% (14.54–27.13)
Israel	11.56% (4.59–24.67)	21.22% (10.63–39.42)	18.55% (10.08–32.87)	19.89% (10.34–36.17)
Italy	13.11% (7.89–22.72)	21.38% (13.44–32.46)	13.58% (5.53–27.69)	17.47% (13.66–22.35)
Japan	15.07% (8.94–24.5)	23.53% (9.09–46.56)	14.89% (6.14–29.94)	19.18% (7.6–38.2)
Luxembourg	14.06% (10.98–19.93)	21.16% (14.66–31.44)	13.58% (5.53–27.69)	17.31% (13.82–22.7)
Malta	9.93% (6.64–14.82)	16.17% (11.34–22.36)	13.58% (5.53–27.69)	14.77% (11.5–20.94)
Monaco	14.24% (3.61–35.98)	20.26% (7.58–41.64)	13.58% (5.53–27.69)	16.95% (6.57–34.73)

Netherlands	17.38% (11.42–25.26)	29.11% (22.13–37.17)	13.73% (7.6–22.34)	21.38% (18.92–24.32)
New Zealand	21.74% (15.54–29.29)	28.69% (19.87–39.79)	21.17% (14.57–30.47)	24.93% (18.85–33.5)
Norway	16.96% (12.3–23.18)	24.8% (17.7–35.19)	14.18% (7.62–23.47)	19.39% (16.99–22.6)
Portugal	12.98% (9.52–19.72)	10.05% (6.88–15.51)	13.58% (5.53–27.69)	11.76% (8.79–17.91)
Republic of Korea	12.64% (7.67–20.11)	23.53% (9.09–46.56)	14.89% (6.14–29.94)	19.1% (7.57–38.06)
San Marino	14.24% (3.61–35.98)	20.26% (7.58–41.64)	13.58% (5.53–27.69)	16.92% (6.56–34.67)
Singapore	4.47% (2.65–8.84)	23.53% (9.09–46.56)	14.89% (6.14–29.94)	19.13% (7.59–38.11)
Spain	16.1% (13.53–19.03)	10.57% (7.81–14.36)	12.25% (7.42–19.07)	11.4% (7.9–15.81)
Sweden	21.28% (13.39–31.77)	22.62% (15.72–31.16)	13.4% (8.59–20.91)	17.89% (14.25–22.25)
Switzerland	7.99% (3.38–18.18)	19.72% (10.37–33.87)	13.38% (4.44–28.55)	16.52% (12.71–21.53)
United Kingdom	18.11% (12.79–27.5)	24.22% (15.18–35.27)	16.2% (9.74–25.96)	20.28% (12.71–30.41)
United States of America	26.45% (22.91–30.27)	27.43% (22.78–32.62)	15.76% (13.69–18.45)	21.59% (19.86–23.8)
Uruguay	17.43% (7.5–32.1)	16.96% (6.42–35.31)	12.3% (5.1–24.95)	14.71% (5.8–30.32)
**Latin America and Caribbean**	**14.74% (13.04–17.38)**	**17.64% (13.73–23.17)**	**13.89% (7.23–24.84)**	**15.81% (11.9–21.88)**
Antigua and Barbuda	18.4% (4.93–43.67)	15.79% (5.64–34.18)	12.78% (5.17–26.23)	14.32% (5.41–30.3)
Bahamas	18.4% (4.93–43.67)	15.79% (5.64–34.18)	12.78% (5.17–26.23)	14.35% (5.41–30.39)
Barbados	18.4% (4.93–43.67)	15.79% (5.64–34.18)	12.78% (5.17–26.23)	14.34% (5.41–30.35)
Belize	13.78% (3.75–31.94)	11.97% (7.16–18.72)	11.46% (7.3–17.33)	11.72% (7.32–18.08)
Bermuda	20.45% (7.06–39.65)	15.79% (5.64–34.18)	12.78% (5.17–26.23)	14.31% (5.41–30.28)
Bolivia (Plurinational State of)	29.53% (26–32.97)	14.56% (5.12–31.94)	14.71% (6.06–29.65)	14.63% (5.58–30.81)
Brazil	10.47% (7.15–17.71)	18.05% (12.38–27.15)	12.85% (6.75–21.77)	15.51% (12.88–18.89)
Colombia	22.54% (13.24–35.63)	19.99% (9.65–37.08)	15.5% (7.93–28.28)	17.77% (8.8–32.84)
Costa Rica	25.16% (13.77–41.59)	30.46% (17.24–48.99)	18.82% (8.31–35.8)	24.64% (20.4–29.88)
Cuba	6.51% (2.84–12.88)	15.79% (5.64–34.18)	12.78% (5.17–26.23)	14.28% (5.4–30.19)
Dominica	18.4% (4.93–43.67)	15.79% (5.64–34.18)	12.78% (5.17–26.23)	14.25% (5.4–30.1)
Dominican Republic	15.82% (10.29–22.79)	15.79% (5.64–34.18)	12.78% (5.17–26.23)	14.31% (5.41–30.27)
Ecuador	27.08% (17.23–39.02)	13.76% (10.53–17.83)	14.71% (6.06–29.65)	14.22% (9.43–22.55)
El Salvador	16.56% (11.07–25.3)	14.31% (11.34–18.32)	11.28% (5.96–20.12)	12.85% (8.93–18.98)
Grenada	18.53% (10.45–30.33)	20.06% (14.23–27.19)	12.78% (5.17–26.23)	16.32% (11.37–24.39)
Guatemala	15.43% (10.53–21.84)	11.58% (9.21–14.79)	14.47% (5.94–29.21)	12.93% (8.06–21.43)

Guyana	31.78% (19.63–47.05)	15.79% (5.64–34.18)	12.78% (5.17–26.23)	14.31% (5.41–30.29)
Haiti	16.77% (13.05–21.56)	24.39% (15.05–39.75)	25.82% (16.71–39.52)	25.07% (15.84–39.65)
Honduras	14.9% (10.68–20.12)	17.97% (13.82–23.29)	15.94% (9.92–26.38)	17.01% (13.06–23.07)
Jamaica	16.02% (10.06–24.58)	17.9% (13.85–23.23)	17.48% (9.77–29.15)	17.7% (13.27–24.05)
Mexico	14.8% (11.7–18.63)	17.34% (7.19–34.51)	13.42% (7.27–25.28)	15.43% (10.9–22.33)
Nicaragua	17.81% (11.6–24.93)	16.83% (11.74–23.24)	13.05% (5.78–24.82)	14.99% (10.1–22.29)
Panama	14.82% (10.77–21.33)	14.97% (5.48–32.2)	14.47% (5.94–29.21)	14.73% (5.73–30.75)
Paraguay	14.1% (8.64–23.48)	8.93% (7.03–11.82)	12.99% (5.26–26.64)	10.94% (6.5–19.26)
Peru	16.96% (12.2–24.48)	20.27% (14.03–28.88)	14.87% (6.55–28.84)	17.57% (12.11–25.66)
Puerto Rico	19.59% (11.83–31.23)	12.62% (4.39–28.17)	12.78% (5.17–26.23)	12.7% (4.79–27.21)
Saint Kitts and Nevis	18.4% (4.93–43.67)	15.79% (5.64–34.18)	12.78% (5.17–26.23)	14.35% (5.41–30.38)
Saint Lucia	18.4% (4.93–43.67)	15.79% (5.64–34.18)	12.78% (5.17–26.23)	14.29% (5.4–30.21)
Saint Vincent and the Grenadines	18.4% (4.93–43.67)	15.79% (5.64–34.18)	12.78% (5.17–26.23)	14.25% (5.4–30.12)
Suriname	19.81% (10.99–32.43)	15.79% (5.64–34.18)	12.78% (5.17–26.23)	14.32% (5.41–30.31)
Trinidad and Tobago	18.64% (9.8–31.78)	15.79% (5.64–34.18)	12.78% (5.17–26.23)	14.28% (5.4–30.2)
United States Virgin Islands	23.2% (14.64–37.59)	18.35% (7.81–36.53)	13.01% (5.66–25.92)	15.74% (6.74–31.32)
Venezuela (Bolivarian Republic of)	13.76% (9.21–20.59)	16.62% (5.96–35.63)	14.47% (5.94–29.21)	15.6% (5.95–32.61)
**North Africa and Middle East**	**20.54% (15.17–32.35)**	**12.28% (4.64–27.83)**	**14.72% (5.94–30.5)**	**13.56% (5.24–28.81)**
Afghanistan	32.03% (27.23–37.66)	11.95% (3.56–29.2)	14.72% (5.94–30.5)	13.3% (4.7–29.48)
Algeria	18.43% (4.95–43.73)	11.95% (3.56–29.2)	14.72% (5.94–30.5)	13.34% (4.73–29.52)
Bahrain	18.43% (4.95–43.73)	11.95% (3.56–29.2)	14.72% (5.94–30.5)	13.73% (5.08–29.61)
Egypt	21.09% (15.36–28.73)	13.71% (4.69–31.23)	14.72% (5.94–30.5)	14.24% (5.26–30.59)
Iran (Islamic Republic of)	18.43% (4.94–43.72)	11.95% (3.56–29.2)	14.72% (5.94–30.5)	13.35% (4.74–29.51)
Iraq	6.65% (5.17–9.64)	11.95% (3.56–29.2)	14.72% (5.94–30.5)	13.37% (4.76–29.52)
Jordan	11.94% (8.44–17.4)	11.95% (3.56–29.2)	14.72% (5.94–30.5)	13.46% (4.84–29.63)
Kuwait	18.43% (4.95–43.73)	11.95% (3.56–29.2)	14.72% (5.94–30.5)	13.7% (5.06–29.59)
Lebanon	6.05% (1.65–15.29)	11.95% (3.56–29.2)	14.72% (5.94–30.5)	13.29% (4.7–29.45)
Libya	18.43% (4.95–43.73)	11.95% (3.56–29.2)	14.72% (5.94–30.5)	13.38% (4.77–29.53)
Morocco	14.11% (7.83–22.85)	11.95% (3.56–29.2)	14.72% (5.94–30.5)	13.35% (4.74–29.52)

Oman	18.43% (4.95–43.73)	11.95% (3.56–29.2)	14.72% (5.94–30.5)	13.7% (5.06–29.59)
Palestine	20.64% (10.67–35)	11.95% (3.56–29.2)	14.72% (5.94–30.5)	13.35% (4.74–29.51)
Qatar	18.43% (4.95–43.73)	11.95% (3.56–29.2)	14.72% (5.94–30.5)	13.88% (5.2–29.65)
Saudi Arabia	18.43% (4.95–43.73)	11.95% (3.56–29.2)	14.72% (5.94–30.5)	13.73% (5.08–29.62)
Sudan	32.1% (15.7–52.85)	11.95% (3.56–29.2)	14.72% (5.94–30.5)	13.37% (4.75–29.54)
Syrian Arab Republic	18.43% (4.95–43.73)	11.95% (3.56–29.2)	14.72% (5.94–30.5)	13.28% (4.67–29.54)
Türkiye	29.62% (20.74–40.24)	12.68% (8.61–19.36)	14.72% (5.94–30.5)	13.68% (7.88–24.2)
Tunisia	13.17% (3.87–28.21)	11.95% (3.56–29.2)	14.72% (5.94–30.5)	13.31% (4.71–29.51)
United Arab Emirates	18.43% (4.95–43.73)	11.95% (3.56–29.2)	14.72% (5.94–30.5)	13.97% (5.23–29.8)
Yemen	18.43% (4.95–43.73)	11.95% (3.56–29.2)	14.72% (5.94–30.5)	13.32% (4.72–29.51)
**South Asia**	**23.58% (17.65–30.09)**	**26.52% (21.21–33.97)**	**15.21% (7.99–27.28)**	**20.8% (15.79–29.68)**
Bangladesh	34.56% (23.72–47.86)	9.29% (6.65–13.66)	27.19% (16.87–41.33)	18.18% (11.8–27.66)
Bhutan	12.02% (9.86–14.49)	11.49% (6–19.58)	19.13% (7.35–39.1)	15.47% (7.42–29.28)
India	23.05% (15.83–31.18)	30.55% (24.85–37.03)	13.22% (6.85–24.15)	21.76% (17.47–29.03)
Nepal	20.08% (14.96–28.21)	14.42% (5.07–31.76)	19.13% (7.35–39.1)	16.68% (6.3–34.85)
Pakistan	18.7% (14.27–24.96)	14.42% (5.07–31.76)	19.13% (7.35–39.1)	16.87% (6.42–35.15)
**Southeast Asia, East Asia, and Oceania**	**19.42% (15.01–25.1)**	**12.15% (9.12–16.68)**	**13.4% (10.22–17.54)**	**12.8% (9.92–16.83)**
American Samoa	29.77% (9.33–60.4)	7.97% (4.95–12.49)	13.27% (5.35–27.27)	10.65% (5.82–18.63)
Cambodia	11.72% (8.01–17.46)	8.59% (6.78–10.97)	18.54% (9.71–31.1)	13.33% (9.98–18.96)
China	21.8% (15.07–30.99)	12.95% (9.58–17.44)	13.21% (8.79–19.8)	13.1% (9.89–18.14)
Cook Islands	21.13% (7.6–41.32)	12.99% (6.01–24.17)	13.27% (5.35–27.27)	13.13% (5.9–25.24)
Democratic People's Republic of Korea	23.58% (5.25–55.6)	13.88% (4.85–30.72)	13.48% (5.45–27.61)	13.7% (5.13–29.23)
Fiji	38.7% (17.39–61.69)	20.46% (14.2–28.45)	13.27% (5.35–27.27)	16.84% (10.76–26.52)
Guam	29.77% (9.33–60.4)	15.36% (5.46–33.4)	13.27% (5.35–27.27)	14.28% (5.38–30.2)
Indonesia	12.88% (6.05–24.18)	10.97% (3.71–25.19)	10.97% (5.02–20.02)	10.96% (4.49–22.53)
Kiribati	53.92% (29.34–78.31)	25.36% (18.59–35.8)	13.27% (5.35–27.27)	19.58% (14.83–26.39)
Lao People's Democratic Republic	11.8% (7.17–21.09)	15.39% (10.4–21.95)	21.62% (10.67–38)	18.51% (11.43–28.93)
Malaysia	3.13% (1.64–5.91)	11.51% (5.48–23.91)	13.85% (5.72–28.24)	12.74% (5.72–25.99)

Maldives	11.53% (8.08–16.91)	17.35% (12.01–26.95)	15.71% (6.49–31.49)	16.31% (10.96–26.29)
Marshall Islands	31.37% (14.3–52.93)	11.81% (5.21–22.06)	13.27% (5.35–27.27)	12.55% (5.56–24.28)
Mauritius	7.75% (4.11–16.3)	10.97% (3.71–25.17)	15.71% (6.49–31.49)	13.33% (5.08–28.29)
Micronesia (Federated States of)	21.68% (9.12–39.64)	14.12% (6.08–27.34)	13.27% (5.35–27.27)	13.68% (5.87–26.49)
Myanmar	14.94% (7.38–28.41)	10.97% (3.71–25.17)	15.71% (6.49–31.49)	13.19% (5–28.09)
Nauru	31.22% (13.07–54.79)	29.18% (19.39–42.03)	13.27% (5.35–27.27)	21.34% (12.82–34.45)
Niue	29.77% (9.33–60.4)	15.36% (5.46–33.4)	13.27% (5.35–27.27)	14.35% (5.39–30.37)
Northern Mariana Islands	29.77% (9.33–60.4)	15.36% (5.46–33.4)	13.27% (5.35–27.27)	14.26% (5.39–30.13)
Palau	16.74% (5.89–33.6)	17.22% (7.74–31.91)	13.27% (5.35–27.27)	15.05% (6.58–29.15)
Papua New Guinea	40.49% (25.86–58.95)	10.41% (7.16–16.42)	13.13% (6.1–23.97)	11.79% (7.13–19.73)
Philippines	8.63% (6.7–11.49)	8.11% (5.18–13.32)	15.71% (6.49–31.49)	11.91% (8.14–19.02)
Samoa	24.19% (11.89–38.96)	10.76% (6.02–19.17)	13.27% (5.35–27.27)	12.01% (7.06–19.57)
Seychelles	26.88% (15.8–44.93)	10.97% (3.71–25.17)	15.71% (6.49–31.49)	13.62% (5.25–28.69)
Solomon Islands	35.29% (25.69–49.26)	42% (33.37–52.83)	13.27% (5.35–27.27)	27.35% (22.3–33.8)
Sri Lanka	14.76% (12.31–17.87)	11.24% (6.48–18.84)	18.91% (8.94–33.86)	14.92% (8.51–24.67)
Taiwan	23.58% (5.25–55.6)	13.88% (4.85–30.72)	13.48% (5.45–27.61)	13.69% (5.14–29.18)
Thailand	20.32% (13.75–32.66)	13.32% (7.78–23.22)	15.71% (6.49–31.49)	14.46% (8.91–22.57)
Timor-Leste	24.5% (18.29–31.67)	29.32% (23.67–35.97)	15.71% (6.49–31.49)	22.39% (16.97–31.24)
Tokelau	29.77% (9.33–60.4)	15.36% (5.46–33.4)	13.27% (5.35–27.27)	14.28% (5.38–30.2)
Tonga	14.04% (6.91–26.19)	13.31% (9.38–19.47)	13.27% (5.35–27.27)	13.29% (8.84–20.67)
Tuvalu	36.36% (16.43–62.07)	15.36% (5.46–33.4)	13.27% (5.35–27.27)	14.28% (5.38–30.19)
Vanuatu	35.88% (26.74–44.7)	15.36% (5.46–33.4)	13.27% (5.35–27.27)	14.32% (5.39–30.3)
Viet Nam	18.45% (14.44–24.8)	7.24% (4.8–11.04)	15.71% (6.49–31.49)	11.41% (5.76–20.7)
**Sub-Saharan Africa**	**22.61% (20.17–28.37)**	**21.83% (10.9–38.95)**	**18.51% (9.83–32.06)**	**20.23% (10.48–35.64)**
Angola	23.87% (10.2–43.3)	18.08% (6.59–38.12)	15.61% (6.47–31.17)	16.92% (6.54–34.88)
Benin	14.38% (9.55–22.21)	16.01% (6.58–32.93)	20.42% (8.87–38.84)	18.13% (7.74–35.77)
Botswana	26.51% (16.6–40.51)	28.52% (13.93–45.9)	26.12% (13.29–41.27)	27.28% (13.56–43.6)
Burkina Faso	12.83% (9–19.17)	21.48% (9.6–41.38)	12.72% (5.33–25.72)	17.35% (7.63–34.27)
Burundi	31.98% (26.64–38.49)	20.67% (7.78–42.29)	13.45% (5.48–27.46)	16.99% (6.6–34.73)
Côte d'Ivoire	16.01% (11.16–23.41)	33.39% (17.28–56.04)	28.62% (14.78–48.22)	30.87% (15.97–52.02)

Cabo Verde	10.69% (5.55–19.72)	19.05% (7.05–39.7)	20.42% (8.87–38.84)	19.72% (7.96–39.24)
Cameroon	27.06% (20.69–34.9)	10.36% (5.82–17.15)	20.42% (8.87–38.84)	15.34% (7.72–26.96)
Central African Republic	23.64% (16.13–34.63)	12.86% (4.55–28.55)	15.61% (6.47–31.17)	14.19% (5.5–29.83)
Chad	23.47% (17.61–30.19)	19.05% (7.05–39.7)	20.42% (8.87–38.84)	19.75% (7.96–39.3)
Comoros	6.03% (2.41–13.08)	13.19% (4.79–28.95)	13.45% (5.48–27.46)	13.33% (5.15–28.23)
Congo	30.04% (9.64–61.08)	18.08% (6.59–38.12)	15.61% (6.47–31.17)	16.85% (6.53–34.68)
Democratic Republic of the Congo	33.62% (25.88–41.84)	18.08% (6.59–38.12)	15.61% (6.47–31.17)	16.85% (6.53–34.66)
Djibouti	27.71% (8.76–57)	20.67% (7.78–42.29)	13.45% (5.48–27.46)	16.78% (6.54–34.3)
Equatorial Guinea	34.01% (13.4–58.36)	18.08% (6.59–38.12)	15.61% (6.47–31.17)	16.82% (6.53–34.61)
Eritrea	27.71% (8.76–57)	20.67% (7.78–42.29)	13.45% (5.48–27.46)	17% (6.61–34.77)
Eswatini	39.26% (25.92–53.79)	17.1% (10.27–26.72)	9.54% (4.67–16.61)	13.4% (7.94–21.09)
Ethiopia	21.63% (17–30.16)	23.75% (9.98–45.63)	8.75% (6.95–11.19)	16.24% (8.99–27.71)
Gabon	25.46% (15.46–38.36)	18.08% (6.59–38.12)	15.61% (6.47–31.17)	16.89% (6.54–34.79)
Gambia	21.83% (17.97–29.37)	19.05% (7.05–39.7)	20.42% (8.87–38.84)	19.73% (7.94–39.29)
Ghana	13.71% (8.4–22.53)	19.05% (7.05–39.7)	20.42% (8.87–38.84)	19.71% (7.93–39.26)
Guinea	32.94% (18.95–49.83)	19.05% (7.05–39.7)	20.42% (8.87–38.84)	19.71% (7.91–39.31)
Guinea-Bissau	15.41% (10.55–22.87)	19.05% (7.05–39.7)	20.42% (8.87–38.84)	19.7% (7.91–39.28)
Kenya	28.6% (20.81–39.75)	27.65% (14.97–45.41)	20.4% (10.87–34.02)	24.1% (12.81–39.82)
Lesotho	30.76% (19.24–45.33)	22.26% (10.54–40.88)	11.9% (5.29–23.38)	17.33% (7.96–32.56)
Liberia	28.1% (22.22–35.17)	19.05% (7.05–39.7)	20.42% (8.87–38.84)	19.76% (7.99–39.27)
Madagascar	44.56% (33.72–57.62)	20.67% (7.78–42.29)	13.45% (5.48–27.46)	17.15% (6.66–35.07)
Malawi	26.32% (18.18–37.91)	27.45% (13.62–47.38)	19.44% (9.59–34.59)	23.66% (11.65–41.42)
Mali	29.11% (15.67–46.04)	19.05% (7.05–39.7)	20.42% (8.87–38.84)	19.75% (7.97–39.3)
Mauritania	8.84% (3.37–18.98)	19.05% (7.05–39.7)	20.42% (8.87–38.84)	19.72% (7.93–39.29)
Mozambique	22.25% (16.83–29.78)	14.3% (6.4–27.22)	14.68% (6.81–28.15)	14.48% (6.63–27.73)
Namibia	12.71% (9.13–18.63)	21.14% (8.91–41.4)	14.95% (6.79–28.9)	18.18% (7.89–35.44)
Niger	8.39% (2.61–20.56)	19.64% (8.24–38.72)	17.36% (7.65–33.29)	18.53% (8.01–36.13)
Nigeria	16.39% (11.65–23.27)	23.79% (13.08–38)	24.26% (13.65–38.85)	23.99% (13.33–38.52)
Rwanda	24.07% (18.56–32.75)	30.12% (23.59–39.19)	17.92% (8.36–31.01)	24.33% (20.56–29.45)
Sao Tome and Principe	20.91% (14.74–30.22)	15.46% (9.33–24.15)	12.1% (6.72–19.84)	13.78% (8.09–21.71)

Senegal	12.81% (10.13–18.04)	19.05% (7.05–39.7)	20.42% (8.87–38.84)	19.71% (7.93–39.27)
Sierra Leone	34.79% (26.69–44.87)	19.05% (7.05–39.7)	20.42% (8.87–38.84)	19.75% (7.96–39.29)
Somalia	10.56% (3.72–22.89)	20.67% (7.78–42.29)	13.45% (5.48–27.46)	17.07% (6.63–34.92)
South Africa	13.24% (10.06–17.74)	24.89% (12.04–45.16)	19.94% (8.79–37.86)	22.52% (10.33–41.77)
South Sudan	27.71% (8.76–57)	20.67% (7.78–42.29)	13.45% (5.48–27.46)	17.14% (6.65–35.03)
Togo	16.95% (13.17–21.95)	19.05% (7.05–39.7)	20.42% (8.87–38.84)	19.68% (7.9–39.27)
Uganda	35.82% (30.3–40.86)	17.87% (8.08–33)	19.8% (10.46–33.44)	18.77% (9.21–33.07)
United Republic of Tanzania	28.43% (22.01–36.96)	19.81% (13.98–28.43)	20.85% (14–31.51)	20.32% (14.05–29.93)
Zambia	31.93% (27.63–36.81)	26.38% (12.88–46.23)	16.15% (8.55–28.03)	21.53% (10.84–37.15)
Zimbabwe	25% (19.5–31.29)	25.5% (16.24–39.76)	7.72% (3.44–15.36)	17.28% (10.29–28.39)

Section 3: IPV and SVAC relative risks

Table S5: Strength of the evidence for the relationship between IPV and the 7 health outcomes analysed with the Burden of Proof methodology

Health outcome	RR (95% UI without heterogeneity)	RR (95% UI with heterogeneity)	BPRF	ROS	Star rating	Pub. bias	No. of studies	Selected bias covariates	Included in GBD 2023
Major depressive disorder	1.91 (1.69, 2.17)	1.91 (1.35, 2.72)	1.42	0.18	★★★	No	12	Representativeness	Yes
Maternal abortion and miscarriage	1.93 (1.55, 2.41)	1.93 (1.15, 3.26)	1.25	0.11	★★	No	9	Non-lifetime recall, risk of reverse causation	Yes
HIV/AIDS	1.58 (1.36, 1.84)	1.58 (1.06, 2.34)	1.13	0.06	★★	No	6	None	Yes
Drug use disorders	1.94 (1.44, 2.62)	1.94 (0.91, 4.12)	1.03	0.02	★★	No	5	Females only included in the effect size, non-lifetime recall	Yes
Anxiety disorders	2.49 (1.72, 3.61)	2.49 (0.8, 7.82)	0.96	-0.02	★	No	5	Non-lifetime recall, females only included in the effect size, risk of selection bias	Yes
Maternal hemorrhage	1.24 (1.06, 1.46)	1.24 (0.86, 1.81)	0.91	-0.05	★	No	3	None	Yes
Self-harm	2.99 (1.36, 6.57)	2.99 (0.29, 30.25)	0.43	-0.42	★	No	4	None	Yes

The reported mean relative risk (RR) reflects the likelihood that a woman who has experienced Intimate Partner Violence (IPV) will develop the health outcome listed in the corresponding row, compared to someone who has not been exposed to this form of violence. The 95% uncertainty interval (UI) for the mean RR without heterogeneity refers to the 95% UI estimated without fully incorporating between-study heterogeneity, while the 95% UI for the mean RR with heterogeneity refers to the 95% UI estimated fully incorporating between-study heterogeneity and the uncertainty around quantified between-study heterogeneity. Outcomes for which the uncertainty without heterogeneity does not cross the null value of 1 are considered for inclusion in the Global Burden of Disease (GBD) study as attributable to IPV. Mean RR and uncertainty values incorporating heterogeneity were used in the calculation of Population Attributable Fractions (PAFs). As the conservative estimate of excess risk consistent with existing evidence, the Burden of Proof Risk Function (BPRF) corresponds to the fifth quantile of RR estimates incorporating between-study heterogeneity that is closest to the null value of 1. The percentage of excess risk is derived as $(BPRF - 1) \times 100$ and

represents the percentage increase or decrease in RR based on a conservative interpretation of the evidence. The Risk-Outcome Score (ROS) is derived as the signed natural log(BPRF)/2. It is determined for risk–outcome pairs found to be statistically significant when estimating a conventional RR and 95% UI (without heterogeneity), and it reflects both the strength of the association and the robustness of the evidence. Risk–outcome pairs not meeting the criteria for ROS calculation are not assigned a star rating. The ROS is translated into a star rating ranging from 1 (weak evidence) to 5 (strong and consistent evidence of a strong association). The risk of publication bias is flagged based on the results of Egger's regression and should inform the interpretation of the results. Statistically significant bias covariates, flagged as reflecting systematic sources of bias, were adjusted for in the final models. Pub., Publication

Table S6: Strength of the evidence for the relationship between SVAC and the 16 health outcomes analysed with the Burden of Proof methodology

Health outcome	RR (95% UI without heterogeneity)	RR (95% UI with heterogeneity)	BPRF	ROS	Star rating	Pub. bias	No. of studies	Selected bias covariates	Included in GBD 2023
Self-harm	2.46 (2.11, 2.86)	2.46 (1.36, 4.44)	1.49	0.20	★★★	No	22	Risk of reverse causation, unadjusted effect size, unadjusted for sex, effect size for both sexes combined	Yes
Alcohol use disorders	1.74 (1.56, 1.94)	1.74 (1.34, 2.25)	1.40	0.17	★★★	No	12	Unadjusted effect size	Yes
Major depressive disorder	1.68 (1.51, 1.87)	1.68 (1.01, 2.8)	1.10	0.05	★★	No	33	Unadjusted for sex, exposure defined including ages above 15, effect size for both sexes combined, subpopulation included	Yes
Asthma	1.25 (1.16, 1.35)	1.25 (1.06, 1.47)	1.09	0.04	★★	No	5	None	Yes
Bipolar disorder	3.43 (2.03, 5.8)	3.43 (0.83, 14.16)	1.05	0.02	★★	No	3	None	Yes
Diabetes mellitus type 2	1.16 (1.1, 1.23)	1.16 (0.99, 1.36)	1.02	0.01	★★	No	11	Exposure defined including ages above 15, males included in the effect size, effect size for both sexes combined	Yes
HIV/AIDS	1.34 (1.12, 1.61)	1.34 (0.87, 2.07)	0.93	-0.04	★	No	7	None	Yes
Drug use disorders	1.87 (1.57, 2.22)	1.87 (0.81, 4.29)	0.93	-0.04	★	No	21	Risk of reverse causation, exposure ascertained from an administrative source, unadjusted for age, unadjusted for age/sex and at least one other confounding variable, males included in the effect size, subpopulation included	Yes
Sexually transmitted	1.28 (1.04, 1.57)	1.28 (0.79, 2.08)	0.85	-0.08	★	No	4	None	Yes

Health outcome	RR (95% UI without heterogeneity)	RR (95% UI with heterogeneity)	BPRF	ROS	Star rating	Pub. bias	No. of studies	Selected bias covariates	Included in GBD 2023
infections, excluding HIV									
Maternal abortion and miscarriage	1.35 (1.11, 1.66)	1.35 (0.75, 2.44)	0.83	-0.09	★	No	6	None	Yes
Anxiety disorders	1.85 (1.5, 2.28)	1.85 (0.72, 4.76)	0.84	-0.09	★	No	17	Females only included in the effect size, risk of selection bias, unadjusted effect size, unadjusted for age, exposure defined including ages above 15, unadjusted for age/sex and at least one other confounding variable, males included in the effect size, outcome is defined as PTSD	Yes
Conduct disorder	3.42 (1.64, 7.14)	3.42 (0.45, 25.7)	0.63	-0.23	★	No	3	None	Yes
Schizophrenia	2.89 (1.64, 5.11)	2.89 (0.38, 21.8)	0.53	-0.32	★	No	7	Unadjusted effect size, unadjusted for age	Yes
Bulimia nervosa	2.95 (1.45, 5.97)	2.95 (0.37, 23.6)	0.51	-0.33	★	No	5	None	Yes
Ischemic Heart Disease	1.32 (0.86-2.04)	1.32 (0.39-4.47)	0.48	N/A	N/A	No	3	None	No
Anorexia	2.07 (0.95-4.51)	2.07 (0.22-19.76)	0.31	N/A	N/A	No	4	None	No

The reported mean relative risk (RR) reflects the likelihood that a woman who has experienced Intimate Partner Violence (IPV) will develop the health outcome listed in the corresponding row, compared to someone who has not been exposed to this form of violence. The 95% uncertainty interval (UI) for the mean RR without heterogeneity refers to the 95% UI estimated without fully incorporating between-study heterogeneity, while the 95% UI for the mean RR with heterogeneity refers to the 95% UI estimated fully incorporating between-study heterogeneity and the uncertainty around quantified between-study heterogeneity. Outcomes for which the uncertainty without heterogeneity does not cross the null value of 1 are considered for inclusion in the Global Burden of Disease (GBD) study as attributable to IPV. Mean RR and uncertainty values incorporating heterogeneity were used in the calculation of Population Attributable Fractions (PAFs). As the conservative estimate of excess risk consistent with existing evidence, the Burden of Proof Risk Function (BPRF) corresponds to the fifth quantile of RR estimates incorporating between-study heterogeneity that is closest to the null value of 1. The percentage of excess risk is derived as (BPRF − 1) × 100 and

represents the percentage increase or decrease in RR based on a conservative interpretation of the evidence. The Risk-Outcome Score (ROS) is derived as the signed natural log(BPRF)/2. It is determined for risk–outcome pairs found to be statistically significant when estimating a conventional RR and 95% UI (without heterogeneity), and it reflects both the strength of the association and the robustness of the evidence. Risk–outcome pairs not meeting the criteria for ROS calculation are not assigned a star rating. The ROS is translated into a star rating ranging from 1 (weak evidence) to 5 (strong and consistent evidence of a strong association). The risk of publication bias is flagged based on the results of Egger's regression and should inform the interpretation of the results. Statistically significant bias covariates, flagged as reflecting systematic sources of bias, were adjusted for in the final models. N/A, not available; Pub., Publication

Section 4: Additional detailed burden results for 2023

Table S7: Cause-specific 15+ age-standardized DALY rates attributable to IPV by world region

Cause Name	IPV DALY rates per 100,000 females by region							
	Global	Central Europe, Eastern Europe, and Central Asia	High-income	Latin America and Caribbean	North Africa and Middle East	South Asia	Southeast Asia, East Asia, and Oceania	Sub-Saharan Africa
Anxiety disorders	181.1 (-41.8–484.4)	95.5 (-18.4–276.0)	253.2 (-62.8–645.3)	272.8 (-54.4–732.2)	194.1 (-40.6–507.8)	209.7 (-42.6–528.1)	107.4 (-25.0–284.8)	185.7 (-48.3–474.5)
Drug use disorders	39.3 (-2.8–89.1)	23.6 (-1.4–57.6)	179.0 (-14.1–406.0)	16.2 (-0.8–39.4)	29.0 (-2.2–70.2)	18.2 (-1.1–42.7)	16.3 (-1.1–38.5)	15.4 (-1.0–38.8)
Major depressive disorder	132.0 (56.9–230.7)	79.6 (32.2–150.5)	146.9 (60.8–250.9)	109.8 (45.1–196.2)	188.7 (66.3–342.1)	157.9 (68.9–277.4)	90.7 (38.7–160.3)	163.4 (62.1–287.6)
Self-harm	100.8 (-71.0–286.6)	66.6 (-28.7–226.0)	105.1 (-67.0–286.9)	51.9 (-22.4–154.6)	35.8 (-19.8–112.2)	197.5 (-146.6–563.7)	55.5 (-30.1–158.9)	80.9 (-53.3–247.5)
HIV/AIDS	67.7 (10.8–133.8)	13.0 (1.7–26.0)	4.1 (0.6–7.9)	19.2 (2.7–35.9)	12.2 (2.0–27.2)	14.1 (2.2–28.6)	10.6 (1.7–22.0)	552.1 (89.7–1087.4)
Interpersonal violence	89.9 (58.5–127.9)	42.5 (29.1–56.2)	45.8 (31.4–65.7)	118.3 (79.5–166.1)	85.2 (59.4–114.3)	66.8 (41.1–100.2)	71.5 (48.2–103.2)	192.5 (115.1–295.5)
Maternal abortion and miscarriage	7.2 (1.6–16.0)	0.4 (0.1–0.9)	0.5 (0.1–1.0)	2.7 (0.6–6.0)	1.8 (0.4–4.2)	3.5 (0.7–7.4)	0.8 (0.1–1.7)	37.1 (8.2–83.2)
Maternal hemorrhage	6.4 (-3.9–17.6)	0.2 (-0.1–0.6)	0.2 (-0.1–0.5)	1.9 (-1.2–5.6)	2.9 (-2.0–9.1)	6.7 (-4.1–18.5)	1.9 (-1.2–5.8)	25.5 (-16.0–71.9)

Figure S2: Age-standardized rates of all-cause DALYs attributable to SVAC by country for females and males aged 15 years and older

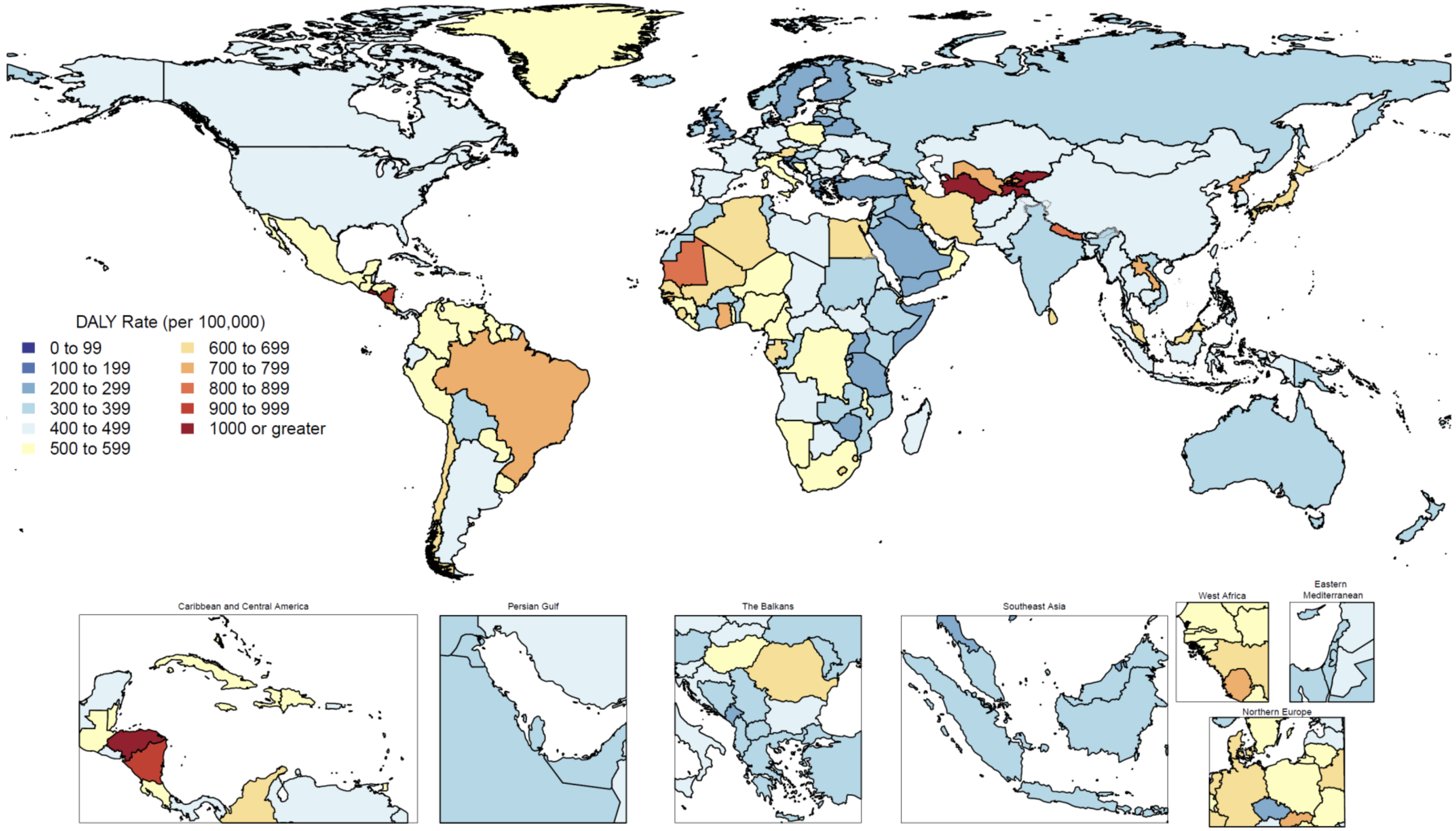

Table S8: Cause-specific 15+ age-standardized DALY rates attributable to SVAC by region and sex

Cause name	Sex	SVAC DALY rates per 100,000 by region							
		Global	Central Europe, Eastern Europe, and Central Asia	High-income	Latin America and Caribbean	North Africa and Middle East	South Asia	Southeast Asia, East Asia, and Oceania	Sub-Saharan Africa
HIV/AIDS	Female	47.1 (-17.6–131.7)	10.9 (-3.3–37.3)	2.7 (-1.0–6.6)	15.0 (-5.3–37.7)	3.7 (-1.3–12.9)	11.0 (-4.1–28.2)	5.0 (-1.8–13.9)	368.7 (-135.0–1047.0)
	Male	35.6 (-11.7–103.0)	11.7 (-3.4–41.1)	4.6 (-1.4–12.3)	23.4 (-7.0–69.2)	2.2 (-0.7–7.0)	6.2 (-1.9–18.7)	10.4 (-3.6–32.0)	289.8 (-96.2–843.6)
	Both sexes	41.3 (-14.6–117.0)	11.3 (-3.4–39.2)	3.6 (-1.2–9.4)	19.1 (-6.4–53.7)	2.9 (-1.0–10.1)	8.6 (-2.8–23.2)	7.8 (-2.8–23.0)	330.7 (-115.9–935.5)
Sexually transmitted infections excluding HIV	Female	2.0 (-1.4–6.5)	1.1 (-0.6–4.4)	1.6 (-1.1–5.5)	2.2 (-1.7–7.7)	0.7 (-0.5–3.3)	2.6 (-2.3–9.2)	0.7 (-0.4–2.3)	5.1 (-3.4–19.0)
	Male	0.6 (-0.4–2.2)	0.3 (-0.2–1.1)	0.3 (-0.2–0.9)	0.8 (-0.4–2.7)	0.6 (-0.3–2.1)	0.4 (-0.3–1.7)	0.5 (-0.3–1.7)	2.2 (-1.3–8.5)
	Both sexes	1.3 (-0.9–4.4)	0.7 (-0.4–2.7)	0.9 (-0.6–3.2)	1.5 (-1.1–4.9)	0.6 (-0.4–2.7)	1.5 (-1.2–5.1)	0.6 (-0.4–1.9)	3.7 (-2.4–14.5)
Maternal abortion and miscarriage	Female	3.4 (-2.3–12.6)	0.2 (-0.1–0.7)	0.2 (-0.2–0.7)	1.5 (-1.1–4.6)	0.6 (-0.3–2.3)	1.4 (-1.0–4.2)	0.3 (-0.2–1.2)	17.0 (-10.5–67.7)
Asthma	Female	16.3 (4.0–36.1)	6.3 (1.4–16.2)	22.7 (5.6–44.6)	14.4 (3.4–29.0)	10.2 (1.7–26.9)	33.7 (6.5–90.4)	7.1 (1.5–16.2)	24.2 (4.5–63.0)
	Male	10.7 (2.4–25.3)	4.4 (0.9–12.1)	10.4 (2.6–21.9)	7.4 (1.6–17.4)	9.1 (1.7–23.9)	18.0 (3.0–48.8)	7.6 (1.8–16.9)	21.1 (4.6–56.7)
	Both sexes	13.5 (3.3–30.0)	5.4 (1.2–14.2)	16.6 (4.2–32.5)	11.0 (2.6–23.5)	9.6 (1.7–25.3)	25.9 (5.0–63.7)	7.3 (1.6–16.0)	22.7 (4.6–57.0)
Diabetes mellitus type 2	Female	33.2 (0.0–79.6)	16.0 (0.0–42.3)	21.9 (-0.0–47.9)	43.2 (-0.1–98.3)	32.5 (0.1–85.2)	78.1 (0.1–196.4)	15.7 (0.0–36.2)	44.5 (0.1–119.4)
	Male	33.1 (0.0–82.4)	18.9 (0.0–52.6)	22.1 (0.0–50.2)	43.2 (0.1–113.0)	46.4 (0.1–121.5)	54.2 (0.1–139.9)	21.7 (0.0–50.8)	55.1 (0.1–145.7)
	Both sexes	33.2 (0.0–81.2)	17.3 (0.0–46.9)	22.0 (0.0–48.3)	43.2 (0.0–99.8)	39.7 (0.1–104.7)	66.4 (0.1–172.2)	18.8 (0.0–43.3)	49.5 (0.1–129.7)
Schizophrenia	Female	66.3 (-34.1–202.9)	47.1 (-18.2–154.7)	81.4 (-50.8–232.9)	66.1 (-35.2–204.3)	56.5 (-19.0–186.2)	80.6 (-55.5–222.2)	52.9 (-19.6–174.0)	72.4 (-34.5–211.0)
	Male	69.2 (-27.7–225.2)	49.6 (-17.5–170.8)	76.1 (-30.9–246.5)	64.8 (-23.2–215.7)	71.7 (-25.9–226.1)	77.0 (-30.1–242.2)	66.3 (-28.2–220.0)	67.6 (-29.1–204.7)
	Both sexes	67.8 (-31.3–214.2)	48.3 (-17.7–162.3)	78.9 (-39.4–238.9)	65.5 (-27.9–211.1)	64.5 (-22.3–205.8)	78.8 (-41.9–234.1)	59.7 (-23.4–197.5)	70.1 (-32.0–207.8)

Major depressive disorder	Female	85.2 (9.1–192.3)	57.7 (6.1–153.1)	121.0 (12.8–263.2)	85.6 (8.4–189.0)	79.7 (7.9–231.0)	111.8 (11.7–247.0)	45.9 (4.4–111.2)	102.8 (11.8–242.5)
	Male	46.0 (4.8–109.8)	30.8 (3.2–84.2)	48.2 (5.0–116.2)	35.7 (3.8–90.3)	56.0 (6.1–149.6)	54.0 (5.4–131.0)	34.4 (3.3–80.2)	65.5 (7.3–154.3)
	Both sexes	65.5 (6.9–156.0)	44.7 (4.7–120.8)	84.5 (8.8–189.2)	61.3 (6.2–137.0)	67.3 (7.1–187.5)	82.6 (8.7–189.8)	40.1 (3.8–92.8)	84.8 (9.6–199.5)
Bipolar disorder	Female	39.4 (-1.2–97.9)	38.1 (-0.8–109.2)	65.4 (-2.2–148.8)	74.6 (-2.1–181.9)	47.6 (-1.2–142.5)	33.8 (-1.1–76.4)	17.8 (-0.4–49.1)	46.2 (-1.4–115.5)
	Male	33.1 (-0.9–89.3)	33.3 (-0.7–98.2)	44.8 (-1.1–117.1)	56.9 (-1.5–157.1)	51.2 (-1.2–143.5)	28.9 (-0.8–77.6)	17.9 (-0.4–46.5)	40.9 (-1.2–105.1)
	Both sexes	36.2 (-1.0–95.6)	35.7 (-0.8–104.3)	55.1 (-1.7–135.4)	66.0 (-1.8–167.8)	49.5 (-1.3–141.7)	31.3 (-1.0–78.3)	17.9 (-0.4–47.8)	43.7 (-1.3–110.5)
Anxiety disorders	Female	92.4 (-41.1–292.7)	53.1 (-19.3–180.8)	156.7 (-76.4–467.4)	157.2 (-69.5–473.9)	75.9 (-28.7–285.5)	104.5 (-50.6–304.4)	43.6 (-16.6–141.1)	92.9 (-34.8–310.0)
	Male	45.4 (-15.8–159.3)	26.4 (-8.6–99.5)	61.2 (-21.7–203.6)	64.2 (-21.1–238.8)	58.9 (-20.5–214.7)	41.8 (-14.8–145.3)	29.3 (-12.2–93.8)	61.3 (-21.6–213.0)
	Both sexes	68.8 (-28.2–220.5)	40.1 (-14.0–141.5)	108.8 (-50.1–333.4)	111.9 (-49.6–358.5)	66.9 (-24.4–251.8)	72.8 (-35.6–227.0)	36.4 (-14.4–116.5)	77.6 (-28.4–265.0)
Bulimia nervosa	Female	18.7 (-10.3–59.8)	16.3 (-6.0–58.9)	38.9 (-23.8–118.1)	34.3 (-16.7–110.6)	19.7 (-6.6–71.0)	18.5 (-11.3–57.0)	9.5 (-3.3–32.4)	10.8 (-5.6–34.7)
	Male	8.0 (-3.1–28.2)	7.4 (-2.6–27.5)	13.6 (-5.3–47.9)	11.3 (-4.4–40.3)	10.8 (-3.7–37.6)	7.1 (-2.7–25.6)	6.1 (-2.4–21.3)	5.0 (-2.3–16.8)
	Both sexes	13.3 (-6.7–43.8)	11.8 (-4.3–42.8)	26.1 (-14.7–82.6)	22.9 (-12.2–75.7)	15.0 (-4.8–53.8)	12.7 (-7.5–40.8)	7.8 (-3.0–26.6)	8.0 (-4.0–26.0)
Conduct disorder	Female	5.0 (-1.7–14.2)	4.8 (-1.2–15.6)	6.2 (-2.0–17.3)	6.2 (-1.9–18.3)	3.0 (-0.6–9.7)	4.9 (-1.9–13.5)	3.1 (-0.8–10.3)	6.7 (-2.2–18.4)
	Male	9.2 (-2.5–27.8)	8.2 (-1.9–26.5)	9.5 (-2.6–29.2)	10.8 (-2.7–30.8)	8.0 (-2.1–24.3)	8.6 (-1.9–27.2)	7.6 (-1.7–23.5)	11.7 (-3.6–33.2)
	Both sexes	7.1 (-2.1–21.3)	6.6 (-1.6–21.2)	7.9 (-2.4–23.5)	8.5 (-2.2–24.4)	5.5 (-1.4–17.8)	6.8 (-1.9–20.5)	5.5 (-1.2–16.9)	9.2 (-3.0–26.1)
Alcohol use disorders	Female	14.0 (6.7–23.0)	29.3 (11.6–58.1)	37.2 (16.7–59.5)	17.2 (6.8–30.1)	2.6 (0.9–6.0)	10.2 (4.8–17.4)	4.6 (1.9–8.7)	15.9 (5.9–31.0)
	Male	46.1 (19.3–83.6)	105.9 (33.6–221.8)	63.9 (28.7–112.3)	66.8 (24.2–128.9)	7.4 (2.4–15.9)	40.7 (16.6–76.1)	39.0 (17.5–64.5)	39.1 (14.2–75.3)
	Both sexes	30.1 (13.0–53.9)	65.8 (22.2–136.8)	50.5 (24.2–83.3)	41.0 (17.4–75.7)	5.1 (1.7–11.1)	25.7 (11.5–46.6)	22.1 (9.9–37.6)	27.1 (10.1–52.4)
Drug use disorders	Female	35.5 (-11.5–88.7)	25.0 (-5.9–78.4)	170.9 (-62.4–435.1)	18.8 (-5.2–51.4)	18.3 (-5.0–63.3)	17.6 (-5.0–44.6)	10.6 (-2.6–30.2)	13.5 (-3.6–39.3)
	Male	44.9 (-10.4–122.7)	67.8 (-15.3–206.7)	204.7 (-50.5–540.6)	21.7 (-5.9–63.1)	33.9 (-7.4–106.0)	14.8 (-4.4–45.5)	18.1 (-4.2–50.0)	15.0 (-3.7–43.9)
	Both sexes	40.2 (-10.9–106.5)	46.4 (-10.6–141.6)	188.0 (-56.7–501.2)	20.2 (-4.9–54.4)	26.5 (-6.2–85.8)	16.2 (-5.0–41.8)	14.5 (-3.4–39.7)	14.2 (-3.6–41.1)
Self-harm	Female	87.1 (28.2–151.6)	53.1 (11.2–115.9)	98.6 (33.1–167.4)	46.1 (14.1–82.9)	20.9 (3.8–48.5)	194.7 (63.1–345.0)	30.0 (8.4–57.6)	64.1 (15.5–139.0)

	Male	138.8 (38.0–284.4)	240.8 (47.4–550.8)	204.4 (56.6–393.9)	137.3 (34.3–283.5)	60.2 (12.0–146.2)	165.6 (39.4–340.0)	64.4 (19.3–128.8)	207.7 (48.9–475.6)
	Both sexes	113.0 (33.8–214.1)	145.1 (29.0–327.6)	151.5 (46.7–285.3)	90.5 (26.7–179.0)	41.5 (8.3–99.2)	179.9 (57.8–342.7)	47.6 (14.3–92.0)	133.0 (34.5–294.6)

Figure S3: Leading 25 Risk Factors, ranked by attributable DALY counts, among females 15+ years old.

World Region

	Global	Central Europe, Eastern Europe, and Central Asia	High−income	Latin America and Caribbean	North Africa and Middle East	South Asia	Southeast Asia, East Asia, and Oceania	Sub−Saharan Africa
High systolic blood pressure	1	1	3	3	1	2	1	2
High fasting plasma glucose	2	3	2	2	3	3	3	5
Particulate matter pollution	3	5	9	5	4	1	2	3
High body−mass index	4	2	1	1	2	4	4	4
Kidney dysfunction	5	7	5	4	5	6	5	8
High LDL cholesterol	6	4	8	7	6	7	6	12
Unsafe sex	7	17	23	8	22	14	14	1
Lead exposure	8	6	14	9	7	8	7	16
Smoking	9	8	4	6	13	15	10	21
Secondhand smoke	10	12	19	15	8	10	8	18
Iron deficiency	11	23	25	12	9	5	18	6
Diet low in fruits	12	14	20	14	16	9	11	11
Intimate partner violence	13	28	11	10	11	12	16	7
Sexual violence against children	14	25	10	11	15	11	20	9
Low bone mineral density	15	21	7	18	26	21	13	24
Diet low in whole grains	16	13	15	19	17	18	12	30
Diet high in sodium	17	16	24	23	27	26	9	23
Diet low in vegetables	18	29	22	13	20	16	24	13
Low temperature	19	11	13	26	12	30	15	32
Low physical activity	20	18	18	17	10	24	17	25
Drug use	21	19	6	25	24	33	27	20
High alcohol use	22	9	12	16	36	36	29	19
Diet low in nuts and seeds	23	10	31	20	14	19	25	34
Unsafe water source	24	45	46	40	42	13	36	10
Occupational injuries	25	32	29	28	35	28	19	17

Rank: 10, 20, 30, 40

Figure S4: Leading 25 Risk Factors, ranked by attributable DALY counts, among females 15-49 years old.

World Region

	Global	Central Europe, Eastern Europe, and Central Asia	High−income	Latin America and Caribbean	North Africa and Middle East	South Asia	Southeast Asia, East Asia, and Oceania	Sub−Saharan Africa
Unsafe sex	1	4	10	4	12	7	7	1
Iron deficiency	2	7	9	6	4	1	9	2
High body−mass index	3	2	4	1	1	6	1	6
Intimate partner violence	4	9	3	3	5	2	5	3
Sexual violence against children	5	8	2	5	8	3	12	5
High fasting plasma glucose	6	12	7	2	2	5	3	11
High systolic blood pressure	7	6	12	8	3	8	2	7
Particulate matter pollution	8	14	23	10	6	4	4	4
Kidney dysfunction	9	13	13	7	9	10	6	8
Drug use	10	5	1	13	10	16	15	16
Occupational injuries	11	15	11	15	27	13	8	9
High LDL cholesterol	12	11	16	12	7	11	10	17
Diet low in fruits	13	17	22	17	19	12	13	14
High alcohol use	14	1	6	9	33	26	21	18
Secondhand smoke	15	16	17	19	11	17	11	19
Smoking	16	3	5	11	15	29	22	27
Low birth weight and short gestation	17	21	15	16	18	9	23	24
Unsafe water source	18	42	45	37	36	14	31	10
Occupational ergonomic factors	19	10	8	14	22	23	16	20
Lead exposure	20	20	29	21	13	15	17	21
Unsafe sanitation	21	43	48	42	41	18	36	12
Diet low in whole grains	22	18	19	23	21	20	14	30
Diet low in vegetables	23	33	26	20	24	22	24	15
Bullying victimization	24	29	14	18	14	30	29	22
Diet low in nuts and seeds	25	19	36	25	20	19	26	33

Rank: 10, 20, 30, 40

Figure S5: Leading 25 Risk Factors, ranked by attributable DALY counts, among males 15+ years old.

World Region

Risk factor	Global	Central Europe, Eastern Europe, and Central Asia	High−income	Latin America and Caribbean	North Africa and Middle East	South Asia	Southeast Asia, East Asia, and Oceania	Sub−Saharan Africa
Smoking	1	2	1	6	2	3	1	9
High systolic blood pressure	2	1	2	3	1	2	2	2
Particulate matter pollution	3	8	9	7	5	1	3	3
High fasting plasma glucose	4	6	4	1	4	4	4	4
High body−mass index	5	5	3	2	3	10	9	7
High LDL cholesterol	6	3	8	8	6	7	5	12
Kidney dysfunction	7	9	7	5	7	9	8	8
High alcohol use	8	4	5	4	28	11	10	6
Lead exposure	9	7	10	9	8	6	7	14
Occupational injuries	10	20	21	10	9	5	12	5
Diet low in fruits	11	13	16	17	12	8	11	11
Diet high in sodium	12	10	19	12	24	16	6	23
Drug use	13	12	6	13	17	27	17	18
Secondhand smoke	14	17	20	16	10	12	14	21
Diet low in whole grains	15	14	12	19	14	14	13	30
Unsafe sex	16	32	32	18	38	36	27	1
Sexual violence against children	17	21	11	11	18	18	19	10
Diet low in nuts and seeds	18	11	27	15	11	13	21	28
Diet low in vegetables	19	24	22	14	21	15	22	13
Low temperature	20	16	17	31	16	31	15	36
Low bone mineral density	21	25	15	21	26	30	16	22
Diet low in legumes	22	15	24	28	22	32	20	31
Diet low in omega−6 polyunsaturated fatty acids	23	18	30	34	19	21	26	25
High temperature	24	36	41	25	13	17	29	20
Diet low in seafood omega−3 fatty acids	25	23	29	23	15	24	34	26

Rank: 10, 20, 30, 40

Figure S6: Leading 25 Risk Factors, ranked by attributable DALY counts, among males 15-49 years old.

World Region

Risk factor	Global	Central Europe, Eastern Europe, and Central Asia	High−income	Latin America and Caribbean	North Africa and Middle East	South Asia	Southeast Asia, East Asia, and Oceania	Sub−Saharan Africa
Occupational injuries	1	7	6	4	5	1	4	2
Smoking	2	2	5	7	2	4	1	10
High alcohol use	3	1	2	1	25	6	3	3
High systolic blood pressure	4	4	8	5	4	3	2	6
High body−mass index	5	6	3	2	1	8	6	7
High fasting plasma glucose	6	9	7	3	3	5	7	9
Particulate matter pollution	7	10	17	12	7	2	5	4
Drug use	8	3	1	10	9	16	10	14
High LDL cholesterol	9	5	9	9	6	7	8	18
Unsafe sex	10	16	20	11	31	26	16	1
Sexual violence against children	11	8	4	8	10	10	12	5
Kidney dysfunction	12	12	11	6	8	13	9	8
Diet low in fruits	13	13	13	14	14	9	11	15
Lead exposure	14	14	24	13	11	11	14	21
Diet low in whole grains	15	15	15	21	16	18	13	31
Low birth weight and short gestation	16	31	16	22	23	12	27	25
Diet low in nuts and seeds	17	11	28	15	12	15	19	29
Iron deficiency	18	24	25	26	22	14	25	16
High temperature	19	34	35	16	13	17	29	19
Diet low in vegetables	20	28	22	18	26	20	23	17
Secondhand smoke	21	22	18	20	17	24	18	22
Diet high in sodium	22	19	26	29	33	28	15	34
Unsafe water source	23	43	46	38	38	19	31	11
Occupational ergonomic factors	24	18	10	19	19	32	22	24
Diet low in fiber	25	29	29	32	35	25	17	37

Figure S7: Percentages of all-cause global DALYs due to IPV and SVAC among females and males across 5-year GBD age groups

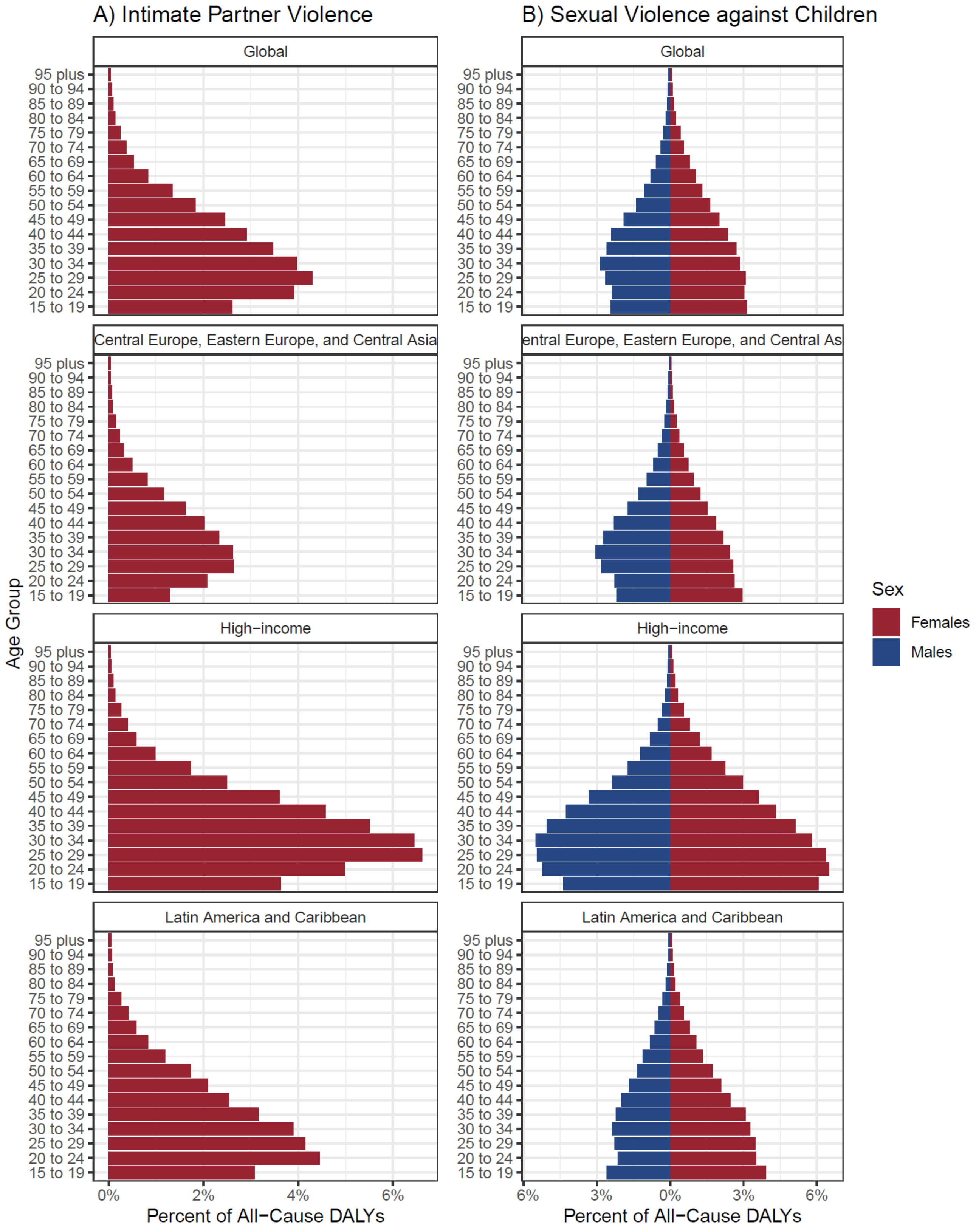

Figure S7: Percentages of all-cause global DALYs due to IPV and SVAC among females and males across 5-year GBD age groups (cont.)

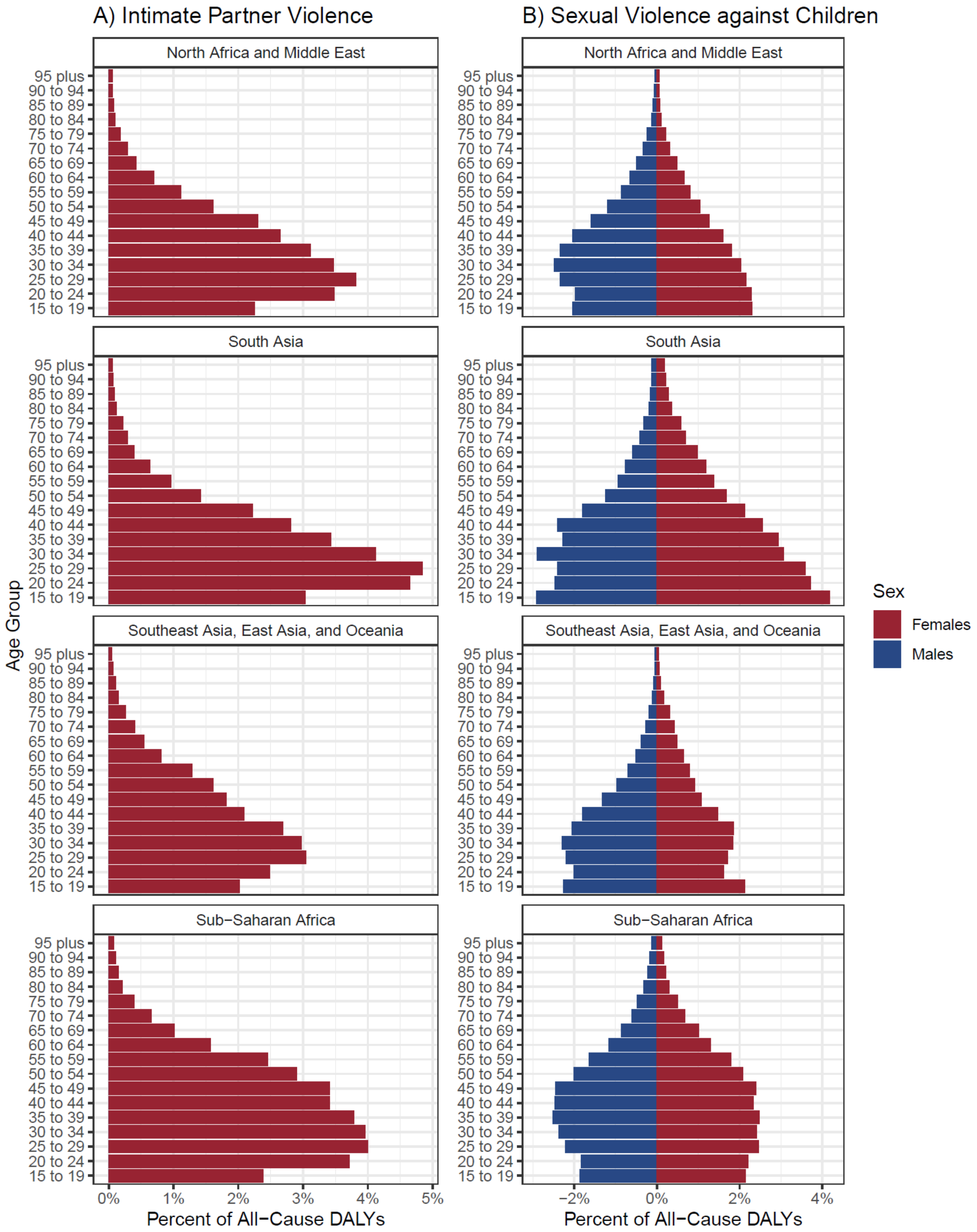

Table S9: Counts of female 15+ cause-specific DALYs attributable to IPV by region

Cause name	Number (in millions) of cause-specific DALYs attributable to IPV by region							
	Global	Central Europe, Eastern Europe, and Central Asia	High-income	Latin America and Caribbean	North Africa and Middle East	South Asia	Southeast Asia, East Asia, and Oceania	Sub-Saharan Africa
HIV/AIDS	2.05 (0.328–4.04)	0.0227 (0.00302–0.0448)	0.0194 (0.00298–0.0374)	0.0467 (0.00649–0.0869)	0.0278 (0.00462–0.0623)	0.0953 (0.0151–0.196)	0.096 (0.0151–0.196)	1.74 (0.283–3.44)
Maternal hemorrhage	0.182 (-0.111–0.499)	0.000298 (-0.000184–0.000821)	0.000603 (-0.000356–0.00165)	0.00428 (-0.00264–0.0128)	0.00667 (-0.00468–0.0212)	0.0494 (-0.0301–0.136)	0.0138 (-0.00853–0.0413)	0.106 (-0.0673–0.298)
Maternal abortion and miscarriage	0.203 (0.0455–0.452)	0.000547 (0.000118–0.00122)	0.00167 (0.00038–0.0035)	0.00602 (0.00129–0.0136)	0.00424 (0.000922–0.00961)	0.0256 (0.00531–0.0544)	0.00566 (0.00105–0.0125)	0.159 (0.0347–0.36)
Major depressive disorder	3.96 (1.71–6.92)	0.146 (0.058–0.285)	0.615 (0.252–1.05)	0.262 (0.108–0.468)	0.42 (0.15–0.761)	1.09 (0.476–1.91)	0.827 (0.356–1.48)	0.594 (0.229–1.03)
Anxiety disorders	5.43 (-1.25–14.6)	0.173 (-0.0328–0.49)	1.05 (-0.249–2.71)	0.647 (-0.128–1.74)	0.43 (-0.0914–1.13)	1.44 (-0.296–3.64)	0.986 (-0.23–2.6)	0.698 (-0.185–1.8)
Drug use disorders	1.16 (-0.0835–2.62)	0.0377 (-0.00222–0.0905)	0.697 (-0.054–1.58)	0.0374 (-0.00189–0.0906)	0.0657 (-0.00498–0.159)	0.127 (-0.00749–0.299)	0.135 (-0.00926–0.313)	0.0597 (-0.00368–0.152)
Self-harm	2.97 (-2.08–8.4)	0.115 (-0.0505–0.39)	0.438 (-0.272–1.21)	0.119 (-0.0519–0.354)	0.0805 (-0.0449–0.252)	1.41 (-1.07–4.02)	0.507 (-0.295–1.46)	0.294 (-0.194–0.894)
Interpersonal violence	2.6 (1.71–3.67)	0.0721 (0.0504–0.0957)	0.176 (0.124–0.238)	0.265 (0.178–0.368)	0.191 (0.133–0.258)	0.473 (0.288–0.722)	0.586 (0.396–0.816)	0.836 (0.493–1.31)

Table S10: Percentages of female 15+ cause-specific DALYs attributable to IPV by region

Cause name	Percentages of cause-specific DALYs attributable to IPV by region							
	**Global**	**Central Europe, Eastern Europe, and Central Asia**	**High-income**	**Latin America and Caribbean**	**North Africa and Middle East**	**South Asia**	**Southeast Asia, East Asia, and Oceania**	**Sub-Saharan Africa**
HIV/AIDS	10.1% (1.5–19.1)	4.9% (0.7–9.9)	10.3% (1.5–19.6)	6.8% (0.9–13.1)	11.3% (1.8–24.0)	6.6% (1.0–12.8)	8.4% (1.2–16.4)	10.7% (1.6–20.3)
Maternal hemorrhage	5.7% (-3.1–15.0)	3.9% (-1.9–10.7)	6.3% (-3.6–16.6)	4.5% (-2.4–12.3)	6.3% (-3.8–19.0)	6.7% (-3.9–17.8)	4.5% (-2.4–13.4)	5.6% (-3.2–15.2)
Drug use disorders	18.0% (-1.2–38.3)	11.2% (-0.6–27.5)	19.7% (-1.4–41.9)	12.2% (-0.6–29.7)	16.4% (-1.2–37.4)	18.3% (-1.1–41.7)	16.1% (-1.0–37.2)	18.1% (-1.1–38.8)
Major depressive disorder	14.7% (7.0–23.7)	9.6% (4.1–16.4)	13.5% (6.1–21.7)	11.2% (5.2–18.6)	15.3% (6.6–26.1)	16.9% (7.7–27.7)	14.9% (7.3–24.6)	15.9% (7.5–25.4)
Anxiety disorders	20.2% (-3.3–49.9)	14.4% (-2.0–37.3)	19.0% (-3.2–47.2)	17.0% (-2.3–43.8)	20.0% (-3.6–50.7)	23.4% (-3.5–54.3)	21.2% (-4.3–50.0)	20.7% (-3.8–49.5)
Self-harm	27.9% (-18.6–78.0)	21.8% (-9.3–72.5)	27.2% (-16.6–75.3)	23.6% (-10.7–70.9)	26.3% (-15.1–80.7)	28.9% (-20.3–79.2)	28.4% (-17.2–80.5)	29.2% (-19.8–81.2)
Interpersonal violence	41.0% (28.5–53.3)	22.6% (16.7–29.4)	37.4% (27.2–49.9)	25.8% (18.2–34.9)	67.6% (49.8–84.8)	38.8% (25.6–54.4)	44.5% (32.2–58.1)	49.1% (32.1–65.4)
Abortion and miscarriage	16.3% (4.0–29.9)	11.6% (2.5–24.7)	17.5% (4.4–33.3)	12.7% (2.9–25.3)	16.7% (3.8–32.0)	21.5% (5.0–41.3)	12.6% (2.7–23.9)	16.0% (3.9–29.0)

Table S11: Counts of female 15+ cause-specific deaths attributable to IPV by region

Cause name	Number (in thousands) of cause-specific deaths attributable to IPV by region							
	**Global**	**Central Europe, Eastern Europe, and Central Asia**	**High-income**	**Latin America and Caribbean**	**North Africa and Middle East**	**South Asia**	**Southeast Asia, East Asia, and Oceania**	**Sub-Saharan Africa**
HIV/AIDS	40.7 (6.4–80.6)	0.416 (0.0534–0.822)	0.307 (0.0459–0.584)	0.956 (0.13–1.8)	0.558 (0.0926–1.26)	1.95 (0.306–4)	2.07 (0.322–4.24)	34.5 (5.5–68.2)
Maternal hemorrhage	3.07 (-1.86–8.47)	0.00361 (-0.00219–0.0111)	0.00696 (-0.00448–0.0205)	0.0733 (-0.0454–0.221)	0.116 (-0.0818–0.365)	0.835 (-0.513–2.27)	0.232 (-0.145–0.705)	1.8 (-1.13–5.1)
Maternal abortion and miscarriage	3.3 (0.746–7.31)	0.0025 (0.000499–0.00539)	0.0101 (0.00213–0.0222)	0.0811 (0.0183–0.187)	0.0743 (0.0166–0.169)	0.428 (0.0899–0.92)	0.0946 (0.0176–0.212)	2.61 (0.575–5.9)
Drug use disorders	8.94 (-0.68–19.8)	0.269 (-0.015–0.657)	6.76 (-0.52–14.5)	0.0995 (-0.00559–0.25)	0.232 (-0.0125–0.628)	0.804 (-0.0609–2.06)	0.421 (-0.0339–1.05)	0.354 (-0.0252–0.941)
Self-harm	60.6 (-39.9–173)	2.7 (-1.15–9.71)	9.99 (-5.56–29.3)	2.26 (-0.98–6.71)	1.41 (-0.779–4.46)	25.4 (-18.5–73.4)	13.2 (-6.69–39.3)	5.72 (-3.7–17.8)
Interpersonal violence	28.2 (18.4–40.5)	1.13 (0.826–1.49)	2.28 (1.66–3.05)	3.96 (2.79–5.36)	1.79 (1.2–2.45)	5.07 (2.8–8.41)	2.47 (1.68–3.53)	11.5 (6.5–18.4)

Table S12: Percentages of female 15+ cause-specific deaths attributable to IPV by region

Cause name	Percentages of cause-specific deaths attributable to IPV by region							
	**Global**	**Central Europe, Eastern Europe, and Central Asia**	**High-income**	**Latin America and Caribbean**	**North Africa and Middle East**	**South Asia**	**Southeast Asia, East Asia, and Oceania**	**Sub-Saharan Africa**
HIV/AIDS	10.7% (1.6–20.4)	5.1% (0.7–10.1)	10.8% (1.6–20.4)	7.2% (1.0–14.0)	11.8% (1.8–24.9)	7.0% (1.0–13.6)	9.1% (1.3–17.5)	11.4% (1.7–21.8)
Maternal hemorrhage	5.9% (-3.2–15.5)	4.2% (-2.1–11.8)	7.1% (-4.0–18.6)	4.7% (-2.5–12.7)	6.6% (-4.0–19.7)	6.9% (-4.1–18.3)	4.6% (-2.5–13.7)	5.8% (-3.3–15.7)
Drug use disorders	18.2% (-1.2–39.2)	10.7% (-0.6–25.9)	19.4% (-1.3–41.3)	11.4% (-0.6–27.1)	13.8% (-0.8–33.5)	17.3% (-1.2–39.4)	14.7% (-1.0–34.2)	19.8% (-1.2–42.8)
Self-harm	26.8% (-17.3–77.9)	20.2% (-8.3–71.6)	24.9% (-14.2–73.0)	23.3% (-10.5–71.3)	26.5% (-15.2–81.2)	28.8% (-20.6–80.1)	26.6% (-14.8–79.8)	28.8% (-18.9–81.5)
Interpersonal violence	37.9% (26.8–48.9)	20.8% (15.6–27.4)	35.6% (25.7–46.8)	25.0% (17.6–33.2)	66.9% (49.9–83.4)	36.7% (25.2–49.0)	42.8% (31.0–55.1)	47.4% (32.0–62.3)
Maternal abortion and miscarriage	17.0% (4.2–31.1)	13.5% (3.1–27.8)	20.9% (4.8–39.2)	13.6% (3.2–26.7)	17.7% (4.1–33.8)	22.1% (5.3–42.1)	12.8% (2.8–24.4)	16.7% (4.1–30.0)

Table S13: Cause-specific 15+ age-standardized death rates attributable to IPV by world region

Cause Name	IPV death rates per 100,000 females by region							
	**Global**	**Central Europe, Eastern Europe, and Central Asia**	**High-income**	**Latin America and Caribbean**	**North Africa and Middle East**	**South Asia**	**Southeast Asia, East Asia, and Oceania**	**Sub-Saharan Africa**
HIV/AIDS	1.3 (0.2–2.6)	0.2 (0.0–0.5)	0.1 (0.0–0.1)	0.4 (0.1–0.7)	0.2 (0.0–0.6)	0.3 (0.0–0.6)	0.2 (0.0–0.4)	11.8 (1.9–23.3)
Maternal hemorrhage	0.1 (-0.1–0.3)	0.0 (-0.0–0.0)	0.0 (-0.0–0.0)	0.0 (-0.0–0.1)	0.0 (-0.0–0.2)	0.1 (-0.1–0.3)	0.0 (-0.0–0.1)	0.4 (-0.3–1.3)
Drug use disorders	0.3 (-0.0–0.6)	0.1 (-0.0–0.4)	1.6 (-0.1–3.3)	0.0 (-0.0–0.1)	0.1 (-0.0–0.3)	0.1 (-0.0–0.3)	0.0 (-0.0–0.1)	0.1 (-0.0–0.3)
Self-harm	2.0 (-1.3–5.6)	1.4 (-0.6–4.8)	2.1 (-1.3–5.9)	1.0 (-0.4–2.9)	0.6 (-0.3–2.0)	3.7 (-2.7–10.6)	1.3 (-0.7–3.9)	1.8 (-1.2–5.6)
Interpersonal violence	1.0 (0.6–1.4)	0.6 (0.4–0.8)	0.6 (0.4–0.8)	1.7 (1.2–2.4)	0.8 (0.5–1.1)	0.7 (0.4–1.2)	0.3 (0.2–0.4)	2.8 (1.7–4.5)
Maternal abortion and miscarriage	0.1 (0.0–0.3)	0.0 (0.0–0.0)	0.0 (0.0–0.0)	0.0 (0.0–0.1)	0.0 (0.0–0.1)	0.1 (0.0–0.1)	0.0 (0.0–0.0)	0.6 (0.1–1.4)

Table S14: Counts of cause-specific DALYs attributable to SVAC by region and sex

Cause name	Sex	Number (in millions) of cause-specific DALYs attributable to SVAC by region							
		Global	Central Europe, Eastern Europe, and Central Asia	High-income	Latin America and Caribbean	North Africa and Middle East	South Asia	Southeast Asia, East Asia, and Oceania	Sub-Saharan Africa
HIV/AIDS	Female	1.41 (-0.524–3.95)	0.0183 (-0.00569–0.0621)	0.0126 (-0.00458–0.0307)	0.0359 (-0.0128–0.0903)	0.00856 (-0.00296–0.0295)	0.0746 (-0.0284–0.192)	0.043 (-0.0152–0.117)	1.22 (-0.452–3.42)
	Male	1.09 (-0.357–3.15)	0.0199 (-0.00585–0.0695)	0.0221 (-0.00682–0.0584)	0.0526 (-0.0155–0.155)	0.00565 (-0.00191–0.0181)	0.0434 (-0.0133–0.132)	0.0936 (-0.0335–0.286)	0.853 (-0.283–2.5)
	Both sexes	2.5 (-0.881–7.08)	0.0383 (-0.0115–0.132)	0.0348 (-0.0114–0.0888)	0.0885 (-0.0297–0.249)	0.0142 (-0.00508–0.0494)	0.118 (-0.0392–0.315)	0.137 (-0.0496–0.403)	2.07 (-0.734–5.85)
Sexually transmitted infections excluding HIV	Female	0.0583 (-0.041–0.193)	0.00195 (-0.00111–0.00765)	0.00663 (-0.00474–0.0229)	0.00523 (-0.00407–0.0183)	0.00165 (-0.00108–0.00753)	0.0176 (-0.0156–0.0627)	0.0058 (-0.00367–0.02)	0.0194 (-0.0131–0.0725)
	Male	0.0186 (-0.0111–0.0653)	0.000495 (-0.000274–0.00182)	0.00112 (-0.0007–0.00394)	0.00166 (-0.00091–0.00589)	0.00138 (-0.000799–0.00526)	0.00277 (-0.00199–0.0109)	0.00397 (-0.0028–0.0143)	0.00719 (-0.00401–0.0273)
	Both sexes	0.0769 (-0.0513–0.263)	0.00245 (-0.00136–0.00938)	0.00775 (-0.00542–0.0268)	0.00689 (-0.00502–0.0229)	0.00303 (-0.00189–0.0128)	0.0204 (-0.0173–0.0707)	0.00977 (-0.00661–0.0324)	0.0266 (-0.0175–0.103)
Maternal abortion and miscarriage	Female	0.0944 (-0.064–0.354)	0.000247 (-0.000157–0.000952)	0.000768 (-0.000566–0.00221)	0.00337 (-0.00236–0.0102)	0.00127 (-0.000723–0.00527)	0.0101 (-0.00726–0.0306)	0.0021 (-0.00125–0.00821)	0.0766 (-0.0472–0.305)
Asthma	Female	0.517 (0.127–1.15)	0.0131 (0.00293–0.0325)	0.104 (0.0262–0.201)	0.0337 (0.00801–0.0677)	0.0209 (0.00352–0.0556)	0.203 (0.0409–0.523)	0.0673 (0.0138–0.155)	0.0748 (0.0143–0.187)
	Male	0.326 (0.0743–0.777)	0.00772 (0.00158–0.0206)	0.0457 (0.0115–0.0951)	0.0163 (0.00356–0.0382)	0.0195 (0.00377–0.0503)	0.109 (0.0182–0.289)	0.0725 (0.0168–0.164)	0.0559 (0.0127–0.139)
	Both sexes	0.843 (0.207–1.86)	0.0209 (0.00465–0.0531)	0.15 (0.0381–0.297)	0.05 (0.0118–0.106)	0.0404 (0.00753–0.107)	0.311 (0.0625–0.751)	0.14 (0.031–0.299)	0.131 (0.0276–0.316)
Diabetes mellitus type 2	Female	1.11 (0.000805–2.7)	0.0436 (0.0000897–0.116)	0.157 (0.0000188–0.343)	0.11 (-0.000179–0.251)	0.063 (0.000164–0.166)	0.461 (0.000525–1.15)	0.173 (-0.00000477–0.397)	0.107 (0.000131–0.284)
	Male	1.04 (0.00136–2.6)	0.0383 (0.0000977–0.107)	0.14 (0.0000809–0.32)	0.097 (0.000216–0.253)	0.0973 (0.000287–0.251)	0.322 (0.000464–0.819)	0.228 (0.000013–0.538)	0.119 (0.000202–0.317)

	Both sexes	2.16 (0.00217–5.29)	0.0819 (0.000187–0.222)	0.297 (0.0000997–0.664)	0.207 (0.0000375–0.478)	0.16 (0.000512–0.418)	0.783 (0.000989–2)	0.401 (0.00000825–0.936)	0.225 (0.000333–0.583)
Schizophrenia	Female	2.03 (-1.04–6.23)	0.0873 (-0.0336–0.291)	0.385 (-0.233–1.1)	0.16 (-0.085–0.496)	0.125 (-0.0424–0.412)	0.542 (-0.368–1.48)	0.489 (-0.18–1.64)	0.244 (-0.119–0.691)
	Male	2.12 (-0.846–6.89)	0.0855 (-0.0305–0.293)	0.345 (-0.139–1.13)	0.146 (-0.0522–0.485)	0.178 (-0.0648–0.551)	0.533 (-0.21–1.68)	0.622 (-0.263–2.07)	0.212 (-0.0927–0.633)
	Both sexes	4.15 (-1.92–13.1)	0.173 (-0.0639–0.588)	0.73 (-0.361–2.23)	0.306 (-0.13–0.986)	0.303 (-0.105–0.953)	1.07 (-0.579–3.2)	1.11 (-0.443–3.76)	0.456 (-0.212–1.32)
Major depressive disorder	Female	2.55 (0.27–5.81)	0.105 (0.011–0.275)	0.513 (0.0526–1.14)	0.201 (0.0196–0.444)	0.177 (0.0176–0.51)	0.766 (0.0798–1.68)	0.403 (0.0374–0.97)	0.385 (0.0433–0.925)
	Male	1.39 (0.144–3.29)	0.0506 (0.00531–0.137)	0.205 (0.0207–0.498)	0.0789 (0.00846–0.2)	0.136 (0.0147–0.366)	0.379 (0.0384–0.928)	0.309 (0.0288–0.724)	0.227 (0.0248–0.545)
	Both sexes	3.93 (0.415–9.34)	0.155 (0.0163–0.41)	0.718 (0.0734–1.66)	0.279 (0.0281–0.625)	0.312 (0.0329–0.863)	1.15 (0.12–2.64)	0.712 (0.0665–1.66)	0.612 (0.0681–1.47)
Bipolar disorder	Female	1.19 (-0.0347–2.96)	0.0685 (-0.00142–0.194)	0.283 (-0.00898–0.648)	0.176 (-0.00477–0.431)	0.106 (-0.00277–0.315)	0.229 (-0.0079–0.515)	0.158 (-0.00331–0.432)	0.167 (-0.00545–0.411)
	Male	1 (-0.026–2.7)	0.0555 (-0.00114–0.162)	0.192 (-0.00478–0.502)	0.126 (-0.00332–0.349)	0.125 (-0.00303–0.349)	0.201 (-0.00574–0.539)	0.163 (-0.00373–0.427)	0.137 (-0.00418–0.351)
	Both sexes	2.19 (-0.0607–5.79)	0.124 (-0.00256–0.356)	0.475 (-0.0138–1.16)	0.302 (-0.00817–0.775)	0.231 (-0.00596–0.66)	0.43 (-0.0136–1.07)	0.32 (-0.00704–0.858)	0.304 (-0.00963–0.764)
Anxiety disorders	Female	2.76 (-1.24–8.84)	0.093 (-0.0344–0.324)	0.666 (-0.326–2.07)	0.368 (-0.164–1.1)	0.168 (-0.0632–0.63)	0.713 (-0.343–2.09)	0.385 (-0.15–1.27)	0.371 (-0.137–1.22)
	Male	1.36 (-0.478–4.76)	0.0421 (-0.0141–0.162)	0.261 (-0.0959–0.866)	0.142 (-0.0466–0.525)	0.142 (-0.0492–0.523)	0.293 (-0.104–1.02)	0.262 (-0.109–0.856)	0.222 (-0.0778–0.767)
	Both sexes	4.13 (-1.71–13.3)	0.135 (-0.0485–0.482)	0.927 (-0.43–2.96)	0.51 (-0.227–1.64)	0.31 (-0.112–1.17)	1.01 (-0.501–3.14)	0.647 (-0.26–2.14)	0.593 (-0.213–1.97)
Bulimia nervosa	Female	0.532 (-0.295–1.7)	0.0226 (-0.00833–0.0785)	0.136 (-0.0834–0.407)	0.0764 (-0.0376–0.247)	0.0451 (-0.0151–0.163)	0.135 (-0.0817–0.416)	0.0681 (-0.0246–0.229)	0.0483 (-0.0253–0.157)
	Male	0.233 (-0.0913–0.827)	0.0105 (-0.00364–0.0379)	0.0485 (-0.0191–0.168)	0.0244 (-0.00954–0.0869)	0.0277 (-0.00943–0.0961)	0.0539 (-0.0203–0.194)	0.0468 (-0.0195–0.158)	0.0212 (-0.00981–0.0708)

	Both sexes	0.765 (-0.387–2.52)	0.0332 (-0.012–0.115)	0.185 (-0.104–0.575)	0.101 (-0.0532–0.332)	0.0728 (-0.0236–0.263)	0.189 (-0.11–0.608)	0.115 (-0.0461–0.391)	0.0694 (-0.0349–0.228)
Conduct disorder	Female	0.134 (-0.0448–0.383)	0.00498 (-0.00122–0.0161)	0.0166 (-0.00551–0.0465)	0.0124 (-0.00372–0.0362)	0.00678 (-0.0013–0.0223)	0.0368 (-0.0139–0.101)	0.0179 (-0.00463–0.0597)	0.0386 (-0.0127–0.106)
	Male	0.26 (-0.0721–0.79)	0.00894 (-0.00212–0.0288)	0.027 (-0.00735–0.0829)	0.0219 (-0.00555–0.0629)	0.0192 (-0.00497–0.0585)	0.0687 (-0.0152–0.217)	0.0487 (-0.0108–0.151)	0.066 (-0.0203–0.187)
	Both sexes	0.394 (-0.119–1.18)	0.0139 (-0.00342–0.0449)	0.0435 (-0.0132–0.13)	0.0343 (-0.00871–0.0979)	0.026 (-0.00636–0.0836)	0.105 (-0.0288–0.317)	0.0666 (-0.0152–0.206)	0.105 (-0.0337–0.297)
Alcohol use disorders	Female	0.425 (0.202–0.698)	0.0552 (0.0214–0.109)	0.161 (0.0757–0.258)	0.0404 (0.0163–0.0709)	0.00577 (0.00197–0.0135)	0.0685 (0.0328–0.115)	0.0402 (0.0164–0.0755)	0.0535 (0.0201–0.104)
	Male	1.42 (0.591–2.56)	0.194 (0.0615–0.401)	0.293 (0.131–0.507)	0.15 (0.0542–0.29)	0.0185 (0.006–0.04)	0.282 (0.117–0.526)	0.361 (0.161–0.594)	0.118 (0.0429–0.224)
	Both sexes	1.84 (0.791–3.3)	0.249 (0.0831–0.519)	0.454 (0.214–0.747)	0.191 (0.0811–0.351)	0.0242 (0.00802–0.0526)	0.351 (0.158–0.639)	0.402 (0.179–0.674)	0.172 (0.0649–0.334)
Drug use disorders	Female	1.04 (-0.34–2.62)	0.0385 (-0.00894–0.121)	0.661 (-0.242–1.67)	0.0428 (-0.0118–0.117)	0.0412 (-0.0113–0.143)	0.122 (-0.0344–0.31)	0.0847 (-0.0202–0.238)	0.0531 (-0.0143–0.154)
	Male	1.34 (-0.311–3.66)	0.103 (-0.0232–0.315)	0.799 (-0.2–2.11)	0.0474 (-0.013–0.138)	0.0855 (-0.0186–0.267)	0.105 (-0.0318–0.323)	0.148 (-0.0343–0.405)	0.0535 (-0.0134–0.157)
	Both sexes	2.39 (-0.65–6.3)	0.142 (-0.0321–0.432)	1.46 (-0.442–3.89)	0.0902 (-0.0218–0.243)	0.127 (-0.0299–0.41)	0.227 (-0.07–0.579)	0.233 (-0.0545–0.627)	0.107 (-0.0268–0.305)
Self-harm	Female	2.55 (0.819–4.44)	0.0885 (0.0188–0.191)	0.411 (0.138–0.697)	0.104 (0.0318–0.188)	0.0471 (0.00868–0.109)	1.39 (0.453–2.46)	0.268 (0.0755–0.513)	0.238 (0.0601–0.508)
	Male	4.16 (1.14–8.51)	0.394 (0.0778–0.903)	0.863 (0.24–1.66)	0.302 (0.0751–0.623)	0.149 (0.0297–0.361)	1.2 (0.287–2.46)	0.573 (0.171–1.15)	0.684 (0.164–1.55)
	Both sexes	6.71 (2–12.7)	0.483 (0.0965–1.09)	1.27 (0.392–2.4)	0.406 (0.119–0.805)	0.196 (0.0394–0.468)	2.59 (0.834–4.94)	0.84 (0.25–1.65)	0.922 (0.241–2.02)

Table S15: Percentages of cause-specific DALYs attributable to SVAC by region and sex

Cause name	Sex	Percentages of cause-specific DALYs attributable to SVAC by region							
		Global	Central Europe, Eastern Europe, and Central Asia	High-income	Latin America and Caribbean	North Africa and Middle East	South Asia	Southeast Asia, East Asia, and Oceania	Sub-Saharan Africa
HIV/AIDS	Female	6.9% (-2.4–18.9)	4.0% (-1.2–13.6)	6.7% (-2.4–17.0)	5.2% (-1.8–13.2)	3.5% (-1.1–12.4)	5.2% (-1.8–13.7)	3.8% (-1.2–10.3)	7.5% (-2.6–20.4)
	Male	5.5% (-1.8–15.6)	2.2% (-0.6–7.2)	4.5% (-1.5–11.9)	4.3% (-1.3–12.3)	2.6% (-0.7–8.1)	2.9% (-0.9–8.5)	3.8% (-1.3–10.2)	6.5% (-2.1–18.4)
	Both sexes	6.2% (-2.1–17.1)	2.8% (-0.8–9.2)	5.1% (-1.8–13.4)	4.6% (-1.5–13.0)	3.1% (-0.9–10.2)	4.0% (-1.3–11.3)	3.8% (-1.2–10.0)	7.1% (-2.4–19.5)
Sexually transmitted infections excluding HIV	Female	6.0% (-4.3–20.4)	4.3% (-2.7–17.4)	6.7% (-5.0–20.5)	5.3% (-3.6–16.6)	3.9% (-2.6–17.0)	7.5% (-6.0–21.5)	3.8% (-2.4–12.5)	6.4% (-4.5–23.9)
	Male	4.7% (-3.2–17.3)	3.3% (-2.0–14.5)	4.5% (-2.9–15.7)	4.3% (-2.8–17.2)	4.5% (-2.8–19.4)	5.2% (-3.8–17.3)	4.2% (-2.9–13.7)	5.1% (-3.4–19.1)
	Both sexes	5.6% (-3.9–19.8)	4.1% (-2.5–16.8)	6.3% (-4.6–19.6)	5.0% (-3.5–15.5)	4.2% (-2.6–18.2)	7.1% (-5.6–21.4)	4.0% (-2.6–12.8)	6.0% (-4.2–22.7)
Abortion and miscarriage	Female	7.5% (-4.9–27.4)	5.4% (-3.0–20.6)	8.2% (-5.7–25.1)	7.2% (-4.6–21.7)	4.9% (-3.0–21.9)	8.4% (-6.2–23.6)	4.6% (-2.7–17.3)	7.6% (-4.9–28.6)
Asthma	Female	4.5% (1.2–9.8)	3.1% (0.7–8.3)	5.4% (1.5–9.8)	4.2% (1.1–8.0)	2.8% (0.5–7.7)	5.8% (1.5–13.1)	2.6% (0.6–6.1)	5.1% (1.2–13.0)
	Male	3.5% (0.8–8.8)	2.8% (0.6–7.8)	3.6% (0.9–7.5)	3.4% (0.8–8.5)	3.5% (0.7–9.7)	3.6% (0.8–8.5)	3.1% (0.8–7.3)	4.2% (1.0–10.5)
	Both sexes	4.1% (1.1–9.3)	3.0% (0.7–8.1)	4.7% (1.3–8.9)	3.9% (1.0–7.7)	3.1% (0.6–8.6)	4.8% (1.3–11.0)	2.9% (0.7–6.6)	4.6% (1.1–11.8)
Diabetes mellitus type 2	Female	2.7% (0.0–6.5)	1.8% (0.0–4.7)	3.2% (-0.0–7.0)	2.3% (-0.0–5.2)	1.7% (0.0–4.9)	4.0% (0.0–9.6)	1.7% (-0.0–4.0)	3.2% (0.0–8.4)
	Male	2.2% (0.0–5.5)	1.8% (0.0–4.9)	2.2% (-0.0–5.0)	2.0% (0.0–5.4)	2.3% (0.0–6.0)	2.3% (0.0–5.8)	2.0% (-0.0–4.8)	2.8% (0.0–7.2)
	Both sexes	2.5% (0.0–6.0)	1.8% (0.0–4.8)	2.6% (-0.0–5.9)	2.2% (-0.0–5.1)	2.0% (0.0–5.4)	3.1% (0.0–7.6)	1.9% (-0.0–4.4)	3.0% (0.0–7.8)
Schizophrenia	Female	26.0% (-12.6–75.2)	22.1% (-7.6–69.1)	29.6% (-16.5–80.4)	25.5% (-12.3–75.4)	21.4% (-7.1–70.2)	30.9% (-17.9–80.8)	21.1% (-7.3–70.1)	29.0% (-12.4–80.4)
	Male	23.8% (-8.4–73.3)	21.2% (-6.7–68.9)	24.2% (-9.4–73.7)	22.9% (-7.6–73.0)	24.1% (-8.5–75.1)	24.7% (-9.1–75.9)	22.7% (-7.9–70.4)	26.7% (-10.7–77.3)
	Both sexes	24.8% (-10.7–74.2)	21.7% (-7.0–69.7)	26.8% (-12.2–76.8)	24.2% (-9.6–74.2)	22.9% (-8.1–73.5)	27.5% (-13.8–77.7)	21.9% (-7.5–70.1)	27.9% (-11.5–79.2)
Major depressive disorder	Female	9.4% (1.2–19.8)	6.8% (0.8–16.7)	11.1% (1.4–21.6)	8.5% (1.0–17.6)	6.4% (0.8–17.2)	11.7% (1.4–22.2)	7.2% (0.8–15.3)	10.2% (1.4–23.6)
	Male	7.8% (1.0–18.1)	6.0% (0.7–15.5)	7.6% (1.0–17.3)	6.9% (0.9–16.6)	7.4% (0.9–19.0)	8.0% (1.0–18.9)	7.8% (0.9–16.2)	8.6% (1.2–20.2)

	Both sexes	8.7% (1.1–19.5)	6.5% (0.8–16.3)	9.8% (1.2–19.5)	8.0% (1.0–16.5)	6.8% (0.8–17.8)	10.1% (1.3–21.5)	7.4% (0.9–16.0)	9.5% (1.3–22.2)
Bipolar disorder	Female	30.6% (-0.9–68.2)	25.5% (-0.6–64.1)	34.8% (-1.2–71.8)	30.1% (-0.9–66.3)	24.2% (-0.6–62.8)	36.8% (-1.4–75.3)	23.8% (-0.5–58.4)	34.1% (-1.1–74.5)
	Male	27.3% (-0.7–66.1)	24.1% (-0.5–62.9)	27.2% (-0.7–65.6)	26.0% (-0.7–63.8)	27.5% (-0.6–69.0)	27.9% (-0.9–67.3)	26.1% (-0.6–63.7)	30.9% (-0.9–70.9)
	Both sexes	29.0% (-0.8–67.3)	24.8% (-0.6–63.1)	31.3% (-1.0–69.6)	28.3% (-0.8–66.0)	25.8% (-0.6–66.7)	32.0% (-1.1–70.5)	24.9% (-0.6–60.9)	32.6% (-1.0–72.9)
Anxiety disorders	Female	10.2% (-3.9–29.5)	7.7% (-2.5–25.9)	11.9% (-5.1–32.2)	9.6% (-3.7–28.4)	7.7% (-3.1–31.7)	11.5% (-4.9–30.7)	8.2% (-2.9–24.2)	10.9% (-4.6–34.2)
	Male	8.3% (-3.1–26.9)	7.3% (-2.4–26.9)	8.3% (-2.9–26.1)	7.4% (-2.9–25.2)	8.5% (-3.1–32.2)	7.6% (-3.0–26.4)	8.8% (-3.0–26.6)	9.2% (-3.6–29.8)
	Both sexes	9.5% (-3.6–28.1)	7.5% (-2.4–26.2)	10.6% (-4.2–30.2)	8.9% (-3.2–26.1)	8.1% (-3.2–32.1)	10.0% (-4.4–28.6)	8.4% (-2.9–24.6)	10.2% (-4.2–32.3)
Bulimia nervosa	Female	28.9% (-14.1–78.7)	24.5% (-8.4–76.2)	31.7% (-17.3–80.4)	28.4% (-13.1–77.0)	23.7% (-8.6–78.6)	32.9% (-20.9–82.7)	23.1% (-8.2–70.0)	31.2% (-15.9–85.7)
	Male	25.3% (-9.6–77.6)	23.2% (-7.2–78.7)	25.6% (-9.6–78.1)	25.1% (-8.9–78.3)	25.8% (-9.1–82.3)	24.9% (-9.4–76.5)	24.6% (-8.5–71.8)	28.6% (-12.2–82.7)
	Both sexes	27.7% (-12.5–78.4)	24.1% (-7.9–77.0)	29.9% (-14.7–80.0)	27.5% (-12.3–75.5)	24.5% (-8.2–79.9)	30.2% (-16.4–81.1)	23.7% (-8.0–70.5)	30.4% (-14.7–84.7)
Conduct disorder	Female	31.1% (-8.8–80.0)	25.9% (-5.3–76.9)	34.1% (-11.1–82.1)	31.0% (-8.8–78.9)	25.1% (-4.9–75.4)	34.3% (-11.0–84.3)	24.8% (-5.3–69.4)	33.1% (-9.6–83.5)
	Male	26.8% (-6.1–76.3)	22.8% (-4.4–72.2)	26.0% (-5.8–75.1)	27.4% (-6.4–75.8)	26.4% (-5.7–78.3)	25.7% (-5.6–76.2)	25.3% (-5.5–70.8)	30.3% (-7.9–80.5)
	Both sexes	28.1% (-6.9–77.5)	23.8% (-4.7–73.9)	28.6% (-7.3–77.3)	28.6% (-7.3–76.8)	26.0% (-5.7–76.7)	28.1% (-7.2–78.6)	25.1% (-5.4–70.4)	31.3% (-8.4–81.6)
Alcohol use disorders	Female	12.7% (6.2–19.8)	9.2% (3.8–17.9)	15.3% (7.0–23.5)	11.5% (5.0–18.7)	8.4% (2.8–17.8)	17.2% (8.4–27.1)	8.3% (3.4–14.2)	13.7% (5.3–25.3)
	Male	9.7% (4.2–17.0)	8.5% (2.8–17.3)	10.1% (4.5–16.6)	8.9% (3.4–16.6)	9.9% (3.2–20.5)	10.2% (4.5–19.0)	9.8% (4.6–15.6)	11.0% (4.4–20.2)
	Both sexes	10.3% (4.6–17.9)	8.6% (2.9–17.5)	11.5% (5.6–18.3)	9.4% (4.0–17.1)	9.5% (3.0–19.6)	11.1% (5.2–19.0)	9.6% (4.4–15.3)	11.7% (4.7–21.7)
Drug use disorders	Female	16.2% (-5.0–40.0)	11.4% (-2.6–36.7)	18.7% (-6.1–44.6)	14.0% (-3.7–36.8)	10.3% (-2.5–37.5)	17.7% (-4.6–43.1)	10.2% (-2.3–28.4)	16.2% (-4.5–45.7)
	Male	12.1% (-2.8–33.5)	10.5% (-2.4–34.9)	12.6% (-3.1–32.6)	11.1% (-3.0–32.4)	11.8% (-2.7–39.1)	12.1% (-3.5–35.8)	11.2% (-2.5–30.5)	13.4% (-3.6–39.3)
	Both sexes	13.6% (-3.6–35.6)	10.7% (-2.4–35.5)	14.8% (-4.3–37.4)	12.3% (-2.9–33.3)	11.3% (-2.8–38.5)	14.6% (-4.3–38.3)	10.8% (-2.4–29.0)	14.7% (-4.0–42.3)
Self-harm	Female	23.9% (7.7–41.6)	16.7% (3.6–35.6)	25.4% (8.5–43.9)	20.5% (6.2–36.8)	15.4% (3.0–34.3)	28.5% (9.7–49.0)	15.0% (4.4–28.2)	23.6% (6.5–46.1)
	Male	17.5% (4.6–35.5)	15.8% (3.3–35.5)	18.0% (5.1–35.2)	16.7% (4.1–34.3)	17.8% (3.4–39.0)	17.1% (4.6–33.7)	16.9% (5.2–31.8)	20.2% (5.0–40.4)
	Both sexes	19.5% (5.6–37.5)	15.9% (3.4–35.5)	19.9% (6.1–37.6)	17.6% (5.0–35.0)	17.1% (3.3–37.6)	21.8% (6.4–38.6)	16.2% (4.9–31.1)	21.0% (5.3–41.6)

Table S16: Counts of cause-specific deaths attributable to SVAC by region and sex

Cause name	Sex	Number (in thousands) of cause-specific deaths attributable to SVAC by region							
		**Global**	**Central Europe, Eastern Europe, and Central Asia**	**High-income**	**Latin America and Caribbean**	**North Africa and Middle East**	**South Asia**	**Southeast Asia, East Asia, and Oceania**	**Sub-Saharan Africa**
HIV/AIDS	Female	26.6 (-9.86–74.4)	0.326 (-0.102–1.07)	0.191 (-0.069–0.479)	0.689 (-0.25–1.75)	0.164 (-0.0602–0.555)	1.51 (-0.566–4.05)	0.866 (-0.309–2.33)	22.9 (-8.54–64.6)
	Male	22 (-7.24–64.1)	0.353 (-0.105–1.21)	0.358 (-0.118–0.95)	1.03 (-0.297–3)	0.111 (-0.0382–0.35)	0.874 (-0.285–2.53)	1.9 (-0.691–5.67)	17.3 (-5.82–51.2)
	Both sexes	48.6 (-17.1–137)	0.68 (-0.206–2.27)	0.549 (-0.189–1.43)	1.72 (-0.579–4.8)	0.276 (-0.101–0.951)	2.38 (-0.76–6.45)	2.77 (-1.02–8.13)	40.2 (-14.4–113)
Sexually transmitted infections excluding HIV	Female	0.438 (-0.343–1.69)	0.014 (-0.00836–0.0611)	0.0296 (-0.0198–0.0981)	0.0298 (-0.021–0.0979)	0.00256 (-0.00127–0.0124)	0.192 (-0.168–0.719)	0.0178 (-0.0101–0.0736)	0.152 (-0.106–0.719)
	Male	0.123 (-0.0791–0.505)	0.00136 (-0.000747–0.00595)	0.00414 (-0.00277–0.0138)	0.00859 (-0.00558–0.034)	0.00268 (-0.0014–0.0124)	0.0324 (-0.0244–0.138)	0.00878 (-0.00646–0.0352)	0.0646 (-0.0363–0.317)
	Both sexes	0.56 (-0.433–2.41)	0.0154 (-0.00913–0.067)	0.0337 (-0.0232–0.111)	0.0384 (-0.0277–0.127)	0.00524 (-0.00286–0.0241)	0.224 (-0.187–0.903)	0.0266 (-0.0147–0.112)	0.217 (-0.136–0.954)
Maternal abortion and miscarriage	Female	1.49 (-0.986–5.61)	0.00105 (-0.000601–0.00405)	0.00396 (-0.00254–0.0133)	0.0434 (-0.0289–0.139)	0.0212 (-0.0121–0.089)	0.166 (-0.121–0.505)	0.0346 (-0.0208–0.135)	1.22 (-0.746–4.85)
Asthma	Female	9.42 (1.54–26.9)	0.0943 (0.0191–0.273)	0.4 (0.0942–0.942)	0.136 (0.0315–0.306)	0.246 (0.0308–0.797)	6.06 (0.833–19.2)	1.2 (0.19–3.61)	1.28 (0.2–3.65)
	Male	5.99 (1.01–15.7)	0.0641 (0.0123–0.195)	0.145 (0.0336–0.316)	0.0918 (0.0174–0.246)	0.297 (0.0483–0.859)	3 (0.431–8.86)	1.27 (0.213–3.14)	1.12 (0.199–3.32)
	Both sexes	15.4 (2.87–39.4)	0.158 (0.0318–0.481)	0.545 (0.128–1.25)	0.228 (0.0523–0.534)	0.543 (0.0861–1.68)	9.06 (1.5–25.8)	2.47 (0.453–6.54)	2.41 (0.424–6.44)
Diabetes mellitus type 2	Female	22.9 (0.00909–58)	0.898 (0.00151–2.61)	2.38 (0.000263–5.31)	2.18 (-0.0027–4.92)	0.857 (0.00105–2.46)	11.2 (0.00546–30.9)	3.13 (0.0000568–8.23)	2.27 (0.00182–6.31)
	Male	19.3 (0.0242–51.3)	0.641 (0.00111–1.79)	2.2 (0.000459–4.77)	2.06 (0.00399–5.56)	1.2 (0.00242–3.45)	6.78 (0.00851–19.2)	3.83 (0.00374–10.5)	2.6 (0.00397–7.75)
	Both sexes	42.2 (0.0333–109)	1.54 (0.00262–4.4)	4.58 (0.000722–9.81)	4.24 (0.00129–10.5)	2.06 (0.00488–5.66)	18 (0.014–47.5)	6.95 (0.0038–17.6)	4.87 (0.00579–13.3)

Alcohol use disorders	Female	3.44 (1.69–5.62)	0.67 (0.246–1.39)	1.64 (0.724–2.52)	0.157 (0.0716–0.254)	0.0134 (0.00333–0.0336)	0.303 (0.132–0.529)	0.106 (0.0454–0.205)	0.546 (0.18–1.19)
	Male	13.8 (5.57–25.3)	2.88 (0.906–5.77)	3.66 (1.68–5.75)	1.49 (0.556–2.86)	0.0769 (0.0212–0.168)	1.56 (0.518–3.23)	2.97 (1.21–5.36)	1.14 (0.371–2.45)
	Both sexes	17.2 (7.17–30.8)	3.55 (1.16–7.11)	5.3 (2.54–8.32)	1.65 (0.64–3.08)	0.0904 (0.0245–0.198)	1.86 (0.677–3.67)	3.07 (1.26–5.53)	1.68 (0.582–3.41)
Drug use disorders	Female	8.24 (-2.71–20.7)	0.278 (-0.0549–0.917)	6.43 (-2.14–16.6)	0.114 (-0.0295–0.297)	0.156 (-0.0365–0.599)	0.71 (-0.199–2.03)	0.263 (-0.0554–0.832)	0.291 (-0.0682–1.04)
	Male	15.4 (-3.68–41)	1.25 (-0.263–3.77)	10.6 (-2.75–28.4)	0.335 (-0.0809–1.03)	0.788 (-0.157–2.41)	0.916 (-0.249–2.47)	1.01 (-0.238–2.6)	0.461 (-0.0976–1.34)
	Both sexes	23.6 (-6.73–61.3)	1.52 (-0.317–4.68)	17 (-5.19–43.2)	0.448 (-0.115–1.31)	0.944 (-0.181–2.99)	1.63 (-0.448–4.25)	1.27 (-0.312–3.37)	0.751 (-0.181–2.31)
Self-harm	Female	51.1 (16.2–90.5)	2.07 (0.424–4.55)	9.64 (3.24–16.5)	1.93 (0.585–3.52)	0.794 (0.147–1.88)	25 (7.98–44.3)	7.11 (1.97–14.1)	4.57 (1.1–9.96)
	Male	90.2 (24.2–189)	8.98 (1.76–20.7)	21 (5.78–40.4)	6.09 (1.47–12.6)	2.79 (0.555–6.8)	23.7 (5.5–48.4)	13.8 (4.19–29.2)	13.9 (3.26–32.1)
	Both sexes	141 (40.6–273)	11.1 (2.2–25.2)	30.6 (9.22–57.7)	8.02 (2.25–16.1)	3.59 (0.716–8.54)	48.7 (15.5–93.6)	20.9 (6.06–42.4)	18.5 (4.77–41.2)

Table S17: Percentages of cause-specific deaths attributable to SVAC by region and sex

Cause name	Sex	Percentages of cause-specific deaths attributable to SVAC by region							
		Global	Central Europe, Eastern Europe, and Central Asia	High-income	Latin America and Caribbean	North Africa and Middle East	South Asia	Southeast Asia, East Asia, and Oceania	Sub-Saharan Africa
HIV/AIDS	Female	7.0% (-2.4–19.0)	4.0% (-1.2–13.3)	6.7% (-2.4–17.3)	5.2% (-1.8–13.3)	3.5% (-1.1–12.4)	5.4% (-1.8–14.2)	3.8% (-1.2–10.1)	7.6% (-2.5–20.6)
	Male	5.6% (-1.8–15.6)	2.2% (-0.6–7.0)	4.5% (-1.5–12.0)	4.3% (-1.3–12.1)	2.6% (-0.7–7.9)	3.0% (-0.9–8.7)	3.7% (-1.2–10.0)	6.6% (-2.1–18.4)
	Both sexes	6.3% (-2.1–17.2)	2.8% (-0.8–9.0)	5.1% (-1.8–13.3)	4.6% (-1.5–13.0)	3.1% (-1.0–10.1)	4.2% (-1.4–11.8)	3.7% (-1.2–9.9)	7.2% (-2.4–19.5)
Sexually transmitted infections excluding HIV	Female	5.8% (-4.1–21.1)	4.1% (-2.4–17.3)	4.6% (-3.1–17.1)	4.4% (-3.2–14.3)	2.7% (-1.7–12.5)	7.2% (-5.5–23.7)	3.1% (-1.9–11.3)	6.1% (-4.2–23.6)
	Male	4.4% (-3.0–16.8)	3.2% (-1.9–13.8)	3.8% (-2.4–13.9)	3.6% (-2.2–15.1)	3.4% (-2.0–15.1)	5.6% (-4.4–19.4)	3.4% (-2.1–13.4)	4.3% (-2.8–16.8)
	Both sexes	5.4% (-3.8–20.0)	4.0% (-2.4–16.8)	4.5% (-3.0–16.6)	4.2% (-3.0–14.6)	3.0% (-1.8–14.0)	6.9% (-5.1–22.6)	3.2% (-2.1–12.0)	5.4% (-3.7–21.6)
Maternal abortion and miscarriage	Female	7.5% (-5.0–27.7)	5.5% (-3.1–22.2)	8.1% (-5.4–27.8)	7.3% (-4.7–22.7)	4.9% (-3.0–21.9)	8.5% (-6.3–23.9)	4.7% (-2.7–17.4)	7.6% (-4.9–28.9)
Asthma	Female	4.0% (1.0–10.0)	2.3% (0.5–7.0)	3.6% (1.0–8.3)	3.0% (0.7–6.9)	2.1% (0.3–5.9)	5.2% (1.3–13.1)	2.1% (0.4–5.7)	4.4% (0.9–11.8)
	Male	3.1% (0.6–8.1)	1.9% (0.4–5.6)	2.8% (0.7–6.6)	2.9% (0.6–7.7)	2.8% (0.6–8.1)	3.2% (0.6–7.9)	2.7% (0.6–7.4)	3.6% (0.8–9.6)
	Both sexes	3.6% (0.8–9.0)	2.1% (0.5–6.4)	3.4% (0.9–7.8)	3.0% (0.7–7.3)	2.4% (0.4–7.0)	4.3% (1.0–10.6)	2.3% (0.5–6.4)	4.0% (0.9–10.7)
Diabetes mellitus type 2	Female	2.4% (0.0–6.4)	1.5% (0.0–4.2)	2.3% (-0.0–5.5)	1.8% (-0.0–4.3)	1.4% (0.0–4.0)	3.6% (0.0–9.5)	1.5% (0.0–3.9)	2.8% (0.0–7.6)
	Male	1.9% (0.0–4.9)	1.6% (0.0–4.4)	1.9% (-0.0–4.2)	1.8% (0.0–4.8)	1.9% (0.0–5.1)	1.9% (0.0–5.1)	1.7% (0.0–4.5)	2.5% (0.0–6.6)
	Both sexes	2.2% (0.0–5.6)	1.5% (0.0–4.3)	2.1% (-0.0–4.8)	1.8% (0.0–4.4)	1.6% (0.0–4.5)	2.7% (0.0–7.1)	1.6% (0.0–4.2)	2.6% (0.0–7.1)
Alcohol use disorders	Female	12.6% (6.1–20.5)	9.1% (3.4–18.1)	15.0% (6.9–23.2)	10.1% (4.6–16.2)	7.5% (2.1–17.1)	18.4% (8.7–28.8)	8.0% (3.4–13.0)	13.1% (4.8–24.9)
	Male	9.5% (4.0–17.3)	8.6% (2.8–17.7)	9.9% (4.5–16.0)	8.5% (3.2–16.1)	9.7% (3.1–20.3)	9.8% (4.1–18.6)	9.8% (4.4–16.9)	11.8% (4.7–21.6)
	Both sexes	10.0% (4.3–17.5)	8.7% (2.9–17.9)	11.1% (5.4–17.8)	8.6% (3.3–15.9)	9.3% (2.9–19.6)	10.6% (4.7–18.9)	9.8% (4.4–16.8)	12.2% (4.7–22.6)
Drug use disorders	Female	16.8% (-5.1–41.1)	11.0% (-2.5–35.8)	18.5% (-6.1–44.4)	13.1% (-3.4–34.8)	9.3% (-2.2–35.3)	15.4% (-3.9–38.8)	9.1% (-2.0–27.0)	16.4% (-4.5–47.6)

	Male	12.5% (-3.0–33.6)	10.7% (-2.4–35.5)	12.8% (-3.2–33.1)	10.9% (-2.8–33.0)	11.7% (-2.7–38.8)	14.4% (-4.6–41.8)	11.1% (-2.4–30.5)	13.0% (-3.4–38.4)
	Both sexes	13.7% (-3.7–35.4)	10.7% (-2.4–35.6)	14.5% (-4.1–37.0)	11.4% (-2.8–33.1)	11.2% (-2.7–38.1)	14.8% (-4.2–40.3)	10.6% (-2.3–29.5)	14.2% (-3.8–41.2)
Self-harm	Female	22.6% (7.2–40.1)	15.5% (3.3–33.7)	23.9% (7.8–42.5)	19.8% (6.1–35.1)	15.0% (2.9–33.7)	28.4% (9.6–48.6)	14.3% (4.2–28.2)	22.9% (6.1–45.5)
	Male	17.0% (4.4–34.9)	15.4% (3.2–34.7)	17.3% (4.8–34.2)	16.3% (4.0–34.1)	17.6% (3.4–38.8)	17.1% (4.6–34.0)	15.6% (4.6–30.4)	19.8% (4.8–39.8)
	Both sexes	18.7% (5.3–36.5)	15.4% (3.2–34.5)	19.0% (5.7–36.5)	17.0% (4.6–34.2)	17.0% (3.2–37.4)	21.6% (6.2–38.9)	15.1% (4.5–29.7)	20.5% (5.1–41.0)

Table S18: Cause-specific 15+ age-standardized death rates attributable to SVAC by region and sex

Cause name	Sex	SVAC death rates per 100,000 by region							
		Global	Central Europe, Eastern Europe, and Central Asia	High-income	Latin America and Caribbean	North Africa and Middle East	South Asia	Southeast Asia, East Asia, and Oceania	Sub-Saharan Africa
HIV/AIDS	Female	0.9 (-0.3–2.4)	0.2 (-0.1–0.6)	0.0 (-0.0–0.1)	0.3 (-0.1–0.7)	0.1 (-0.0–0.2)	0.2 (-0.1–0.6)	0.1 (-0.0–0.3)	7.5 (-2.8–21.3)
	Male	0.7 (-0.2–2.1)	0.2 (-0.1–0.7)	0.1 (-0.0–0.2)	0.5 (-0.1–1.3)	0.0 (-0.0–0.1)	0.1 (-0.0–0.4)	0.2 (-0.1–0.6)	6.4 (-2.2–18.7)
	Both sexes	0.8 (-0.3–2.2)	0.2 (-0.1–0.7)	0.1 (-0.0–0.1)	0.4 (-0.1–1.0)	0.1 (-0.0–0.2)	0.2 (-0.1–0.5)	0.1 (-0.1–0.4)	7.0 (-2.5–19.9)
Sexually transmitted infections excluding HIV	Female	0.0 (-0.0–0.1)	0.0 (-0.0–0.0)	0.0 (-0.0–0.0)	0.0 (-0.0–0.0)	0.0 (-0.0–0.0)	0.0 (-0.0–0.1)	0.0 (-0.0–0.0)	0.0 (-0.0–0.2)
	Male	0.0 (-0.0–0.0)	0.0 (-0.0–0.0)	0.0 (-0.0–0.0)	0.0 (-0.0–0.0)	0.0 (-0.0–0.0)	0.0 (-0.0–0.0)	0.0 (-0.0–0.0)	0.0 (-0.0–0.1)
	Both sexes	0.0 (-0.0–0.0)	0.0 (-0.0–0.0)	0.0 (-0.0–0.0)	0.0 (-0.0–0.0)	0.0 (-0.0–0.0)	0.0 (-0.0–0.1)	0.0 (-0.0–0.0)	0.0 (-0.0–0.2)
Maternal abortion and miscarriage	Female	0.1 (-0.0–0.2)	0.0 (-0.0–0.0)	0.0 (-0.0–0.0)	0.0 (-0.0–0.1)	0.0 (-0.0–0.0)	0.0 (-0.0–0.1)	0.0 (-0.0–0.0)	0.3 (-0.2–1.1)
Asthma	Female	0.3 (0.0–0.8)	0.0 (0.0–0.1)	0.1 (0.0–0.1)	0.1 (0.0–0.1)	0.2 (0.0–0.5)	1.1 (0.1–3.6)	0.1 (0.0–0.3)	0.6 (0.1–1.7)
	Male	0.2 (0.0–0.5)	0.0 (0.0–0.1)	0.0 (0.0–0.0)	0.0 (0.0–0.1)	0.2 (0.0–0.6)	0.6 (0.1–1.7)	0.1 (0.0–0.3)	0.6 (0.1–1.8)
	Both sexes	0.2 (0.0–0.6)	0.0 (0.0–0.1)	0.0 (0.0–0.1)	0.0 (0.0–0.1)	0.2 (0.0–0.5)	0.9 (0.1–2.6)	0.1 (0.0–0.3)	0.6 (0.1–1.7)
Diabetes mellitus type 2	Female	0.7 (0.0–1.6)	0.3 (0.0–0.9)	0.2 (-0.0–0.5)	0.8 (-0.0–1.9)	0.5 (0.0–1.6)	2.1 (0.0–5.8)	0.3 (-0.0–0.7)	1.1 (0.0–3.2)
	Male	0.6 (0.0–1.7)	0.3 (0.0–0.9)	0.3 (0.0–0.7)	0.9 (0.0–2.6)	0.7 (0.0–2.1)	1.3 (0.0–3.7)	0.4 (0.0–1.0)	1.5 (0.0–4.4)
	Both sexes	0.6 (0.0–1.7)	0.3 (0.0–0.9)	0.3 (-0.0–0.6)	0.9 (0.0–2.2)	0.6 (0.0–1.8)	1.7 (0.0–4.6)	0.3 (0.0–0.8)	1.3 (0.0–3.6)
Alcohol use disorders	Female	0.1 (0.1–0.2)	0.3 (0.1–0.6)	0.3 (0.1–0.4)	0.1 (0.0–0.1)	0.0 (0.0–0.0)	0.0 (0.0–0.1)	0.0 (0.0–0.0)	0.2 (0.1–0.4)
	Male	0.4 (0.2–0.8)	1.5 (0.5–3.0)	0.7 (0.3–1.0)	0.7 (0.2–1.3)	0.0 (0.0–0.1)	0.2 (0.1–0.5)	0.3 (0.1–0.5)	0.5 (0.2–1.0)
	Both sexes	0.3 (0.1–0.5)	0.9 (0.3–1.8)	0.5 (0.2–0.8)	0.3 (0.1–0.6)	0.0 (0.0–0.0)	0.1 (0.1–0.3)	0.2 (0.1–0.3)	0.3 (0.1–0.7)
Drug use disorders	Female	0.3 (-0.1–0.7)	0.2 (-0.0–0.5)	1.5 (-0.5–3.8)	0.0 (-0.0–0.1)	0.1 (-0.0–0.3)	0.1 (-0.0–0.3)	0.0 (-0.0–0.1)	0.1 (-0.0–0.3)

	Male	0.5 (-0.1–1.3)	0.8 (-0.2–2.3)	2.5 (-0.6–6.6)	0.2 (-0.0–0.5)	0.3 (-0.1–1.0)	0.1 (-0.0–0.4)	0.1 (-0.0–0.3)	0.2 (-0.0–0.4)
	Both sexes	0.4 (-0.1–1.0)	0.5 (-0.1–1.4)	2.0 (-0.6–5.0)	0.1 (-0.0–0.3)	0.2 (-0.0–0.7)	0.1 (-0.0–0.3)	0.1 (-0.0–0.2)	0.1 (-0.0–0.4)
Self-harm	Female	1.7 (0.5–3.0)	1.1 (0.2–2.4)	2.0 (0.7–3.4)	0.8 (0.3–1.5)	0.4 (0.1–0.9)	3.6 (1.2–6.5)	0.7 (0.2–1.4)	1.4 (0.3–3.2)
	Male	3.0 (0.8–6.3)	5.2 (1.0–12.0)	4.4 (1.2–8.4)	2.8 (0.7–5.7)	1.2 (0.2–2.9)	3.4 (0.8–7.0)	1.5 (0.4–3.1)	5.0 (1.1–11.8)
	Both sexes	2.3 (0.7–4.5)	3.0 (0.6–6.9)	3.2 (1.0–6.0)	1.8 (0.5–3.5)	0.8 (0.2–1.9)	3.5 (1.1–6.8)	1.1 (0.3–2.2)	3.1 (0.8–7.1)

Figure S8: Percentages of cause-specific DALYs attributable to SVAC by world region and sex

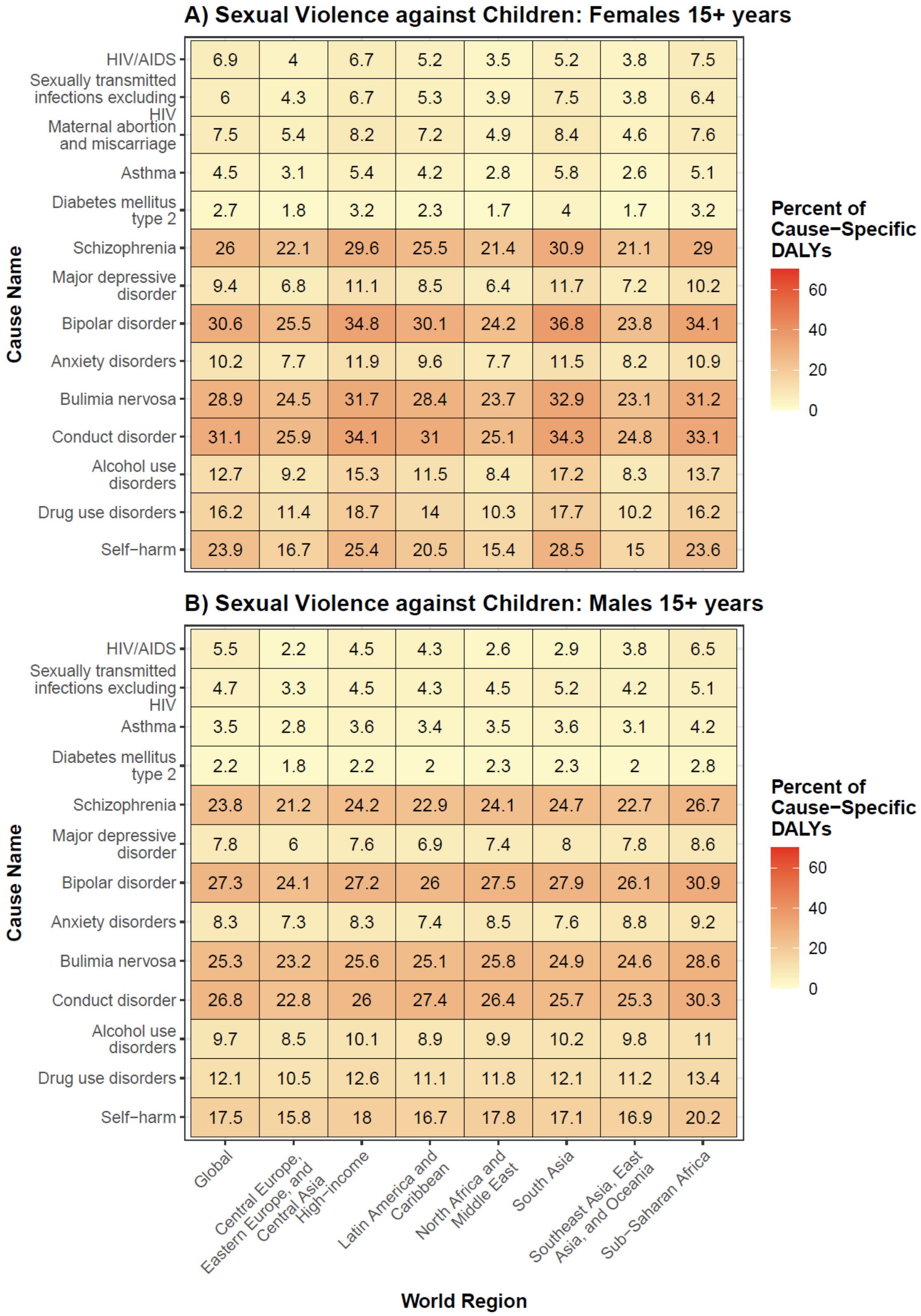

Figure S9: Global and regional rates of cause-specific deaths attributable to IPV and SVAC in 2023

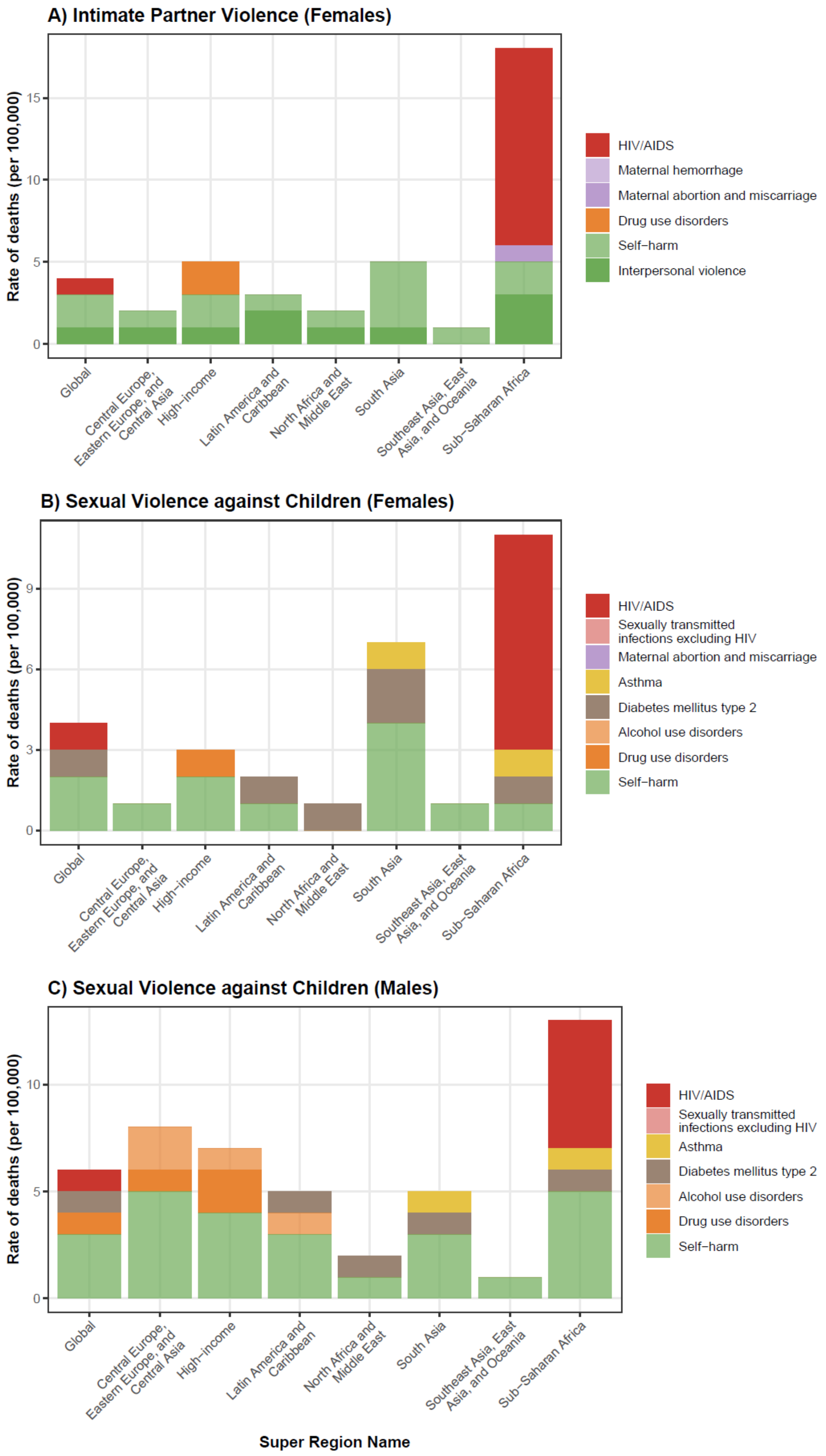

Supplement: Supplementary appendix 2 [file mmc2.pdf]
