# Supplementary material for: Disease burden attributable to intimate partner violence against females and sexual violence against children in 204 countries and territories, 1990–2023: a systematic analysis for the Global Burden of Disease Study 2023
Source: Lancet. 2026 Jan 3;407(10523):31–52. doi: 10.1016/S0140-6736(25)02503-6 (PMC12775558; doi:10.1016/S0140-6736(25)02503-6)
Supplement: Supplementary appendix 3 [file mmc3.pdf]

THE LANCET

Supplementary appendix 3

This appendix formed part of the original submission and has been peer reviewed. We post it as supplied by the authors.

Supplement to: GBD 2023 Intimate Partner Violence and Sexual Violence against Children Collaborators. Disease burden attributable to intimate partner violence against females and sexual violence against children in 204 countries and territories, 1990–2023: a systematic analysis for the Global Burden of Disease Study 2023. *Lancet* 2025; published online Dec 9. https://doi.org/10.1016/S0140-6736(25)02503-6.

Appendix 3: Authorship appendix to "Disease burden attributable to intimate partner violence against females and sexual violence against children in 204 countries and territories, 1990-2023: a systematic analysis for the Global Burden of Disease Study 2023"

This appendix provides further authorship detail for "Disease burden attributable to intimate partner violence against females and sexual violence against children in 204 countries and territories, 1990-2023: a systematic analysis for the Global Burden of Disease Study 2023"

Table of Contents

GBD 2023 Intimate Partner Violence and Sexual Violence against Children Collaborators 2

Affiliations 4

Authors' Contributions.. 17

Managing the overall research enterprise... 17

Writing the first draft of the manuscript 17

Primary responsibility for applying analytical methods to produce estimates 17

Primary responsibility for seeking, cataloguing, extracting, or cleaning data; designing or coding figures and tables.. 17

Providing data or critical feedback on data sources... 17

Developing methods or computational machinery 19

Providing critical feedback on methods or results 19

Drafting the work or revising it critically for important intellectual content 21

Managing the estimation or publications process... 23

GBD 2023 Intimate Partner Violence and Sexual Violence against Children Collaborators

Luisa S Flor, Cory N Spencer, Jack Cagney, Gabriela Fernanda Gil, Hasan Aalruz, Samar Abd ElHafeez, Siddig Ibrahim Abdelwahab, Meriem Abdoun, Mesfin Abebe, Yonas Abebe, Armita Abedi, Roberto Ariel Abeldaño Zuñiga, Alemwork Abie, Olumide Abiodun, Richard Gyan Aboagye, Lucas Guimarães Abreu, Rana Kamal Abu Farha, Bilyaminu Abubakar, Sawsan Abuhammad, Meshack Achore, Lisa C Adams, Babatope Oluwadamilare Adebiyi, Kamoru Ademola Adedokun, Oluwatobi E Adegbile, Nurudeen A Adegoke, Olumide Thomas Adeleke, Makinde Adebayo Adeniyi, Miracle Ayomikun Adesina, Habeeb Omoponle Adewuyi, Qorinah Estiningtyas Sakilah Adnani, Leticia Akua Adzigbli, Aanuoluwapo Adeyimika Afolabi, Rotimi Felix Afolabi, Muhammad Sohail Afzal, Saira Afzal, Williams Agyemang-Duah, Bright Opoku Ahinkorah, Aqeel Ahmad, Danish Ahmad, Muayyad M Ahmad, Asma Ahmed, Ayman Ahmed, Haroon Ahmed, Mehrunnisha Sharif Ahmed, Oli Ahmed, Elizabeth Oluwatoyin Akin-Odanye, Wole Akosile, Idorenyin Ubon Akpabio, Zufishan Alam, Rasmieh Mustafa Al-Amer, Amani N Alansari, Turki M Alanzi, Shereen M Aleidi, Melaku Birhanu Alemu, Fadwa Naji Alhalaiqa, Montaha Al-Iede, Hamid Alinejad Rokny, Wesam Taher Almagharbeh, Md Al-Mamun, Joseph Uy Almazan, Mohmmad Minwer Alnaeem, Intima Alrimawi, Najim Z Alshahrani, Mohammad Sharif Ibrahim Alyahya, Tarek Tawfik Amin, Saeed Amini, Sohrab Amiri, Hubert Amu, Jimoh Amzat, David B Anderson, Boluwatife Stephen Anuoluwa, Saeid Anvari, Anayochukwu Edward Anyasodor, Aleksandr Y Aravkin, Jorge Arias de la Torre, Benedetta Armocida, Alejandra Arrieta, Deepavalli Arumuganainar, Tahira Ashraf, Bilal Aslam, Yuni Asri, Seyyed Shamsadin Athari, Prince Atorkey, Sachin R Atre, Abadi Hailay Atsbaha, Julie Alaere Atta, Madhu Sudhan Atteraya, Ahmed Y Azzam, Sheeba B, Khlood K Baghlaf, Atif Amin Baig, Wondu Feyisa Balcha, Jose Balmori-de-la-Miyar, Soham Bandyopadhyay, Manish Barik, Suzanne Lyn Barker-Collo, MD Abu Bashar, Shahid Bashir, Azadeh Bashiri, Mohammad-Mahdi Bastan, Narasimha M Beeraka, Melesse Belayneh, Gokce Belge Bilgin, Michelle L Bell, Abiye Assefa Berihun, Amiel Nazer C Bermudez, Arushee Bhatnagar, Ashmin Hari Bhattarai, Mohammad Shahangir Biswas, Espen Bjertness, Obasanjo Afolabi Bolarinwa, Paria Bolourinejad, Sri Harsha Boppana, Alejandro Botero Carvajal, Souad Bouaoud, Traolach Brugha, Danilo Buonsenso, Richard A Burns, Yasser Bustanji, Andrea Carugno, Andre F Carvalho, Felix Carvalho, Joao Mauricio Castaldelli-Maia, Sonia Cerrai, Joht Singh Chandan, Miyuru Chandradasa, Periklis Charalampous, Vijay Kumar Chattu, Anis Ahmad Chaudhary, Sirshendu Chaudhuri, Haiyan Chen, Patrick R Ching, Hitesh Chopra, Chidozie Williams Chukwu, Isaac Sunday Chukwu, Sunghyun Chung, Patricia Cullen, Alanna Gomes da Silva, Berihun Assefa Dachew, Omid Dadras, Xiaochen Dai, Koustuv Dalal, Rakhi Dandona, Lucio D'Anna, Samuel Demissie Darcho, Harsha Dayal, Erin M DeGraw, Endalkachew Dellie, Keshab Deuba, Syed Masudur Rahman Dewan, Diana Dias da Silva, Daniel Diaz, Sushil Dohare, Robert Kokou Dowou, Emeka W Dumbili, Jennifer Dunne, Ejemai Eboreime, Cynthia Edeh, Hisham Atan Edinur, Michael Ekholuenetale, Doaa Abdel Wahab El Morsi, Gilbert Eshun, Adeniyi Francis Fagbamigbe, Qiping Fan, Alireza Farahani, Andre Faro, Abidemi Omolara Fasanmi, Alireza Feizkhah, Nuno Ferreira, Richard Charles Franklin, Deborah Ann Fry, Xiang Gao, Miglas Welay Gebregergis, Mesfin Gebrehiwot, Yohannes Fikadu Geda, Miesa Gelchu, Genanew K Getahun, Shakiba Ghasemi Assl, Ehsan Gholami, Nasim Gholizadeh, Elena Ghotbi, Jaleed Ahmed Gilani, Alem Abera Girmay, Mahaveer Golechha, Pouya Goleij, Michal Grivna, Shi-Yang Guan, Damitha Asanga Gunawardane, Sapna Gupta, Pritam Halder, Hassen Mosa Halil, Asif Hanif, Nasrin Hanifi, Habtamu Endashaw Hareru, Josep Maria Haro, Eka Mishbahatul Marah Has, Ahmed I Hasaballah, Hamidreza Hasani, Simon I Hay, Molly E Herbert, Marjan Hesari, Mbuzeleni Hlongwa, Mazeda Hossain, Md Mahbub Hossain, Md Sabbir Hossain, Mohammad Bellal Hossain, Chengxi Hu, Junjie Huang, Yongsong Huang, Pulwasha Maria Iftikhar,

Meesha Iqbal, Lalu Muhammad Irham, Teresa R Iskander, Md Shahinul Islam, Sheikh Mohammed Shariful Islam, Roxana Jabbarinejad, Belayneh Hamdela Jena, Ravi Prakash Jha, Nitin Joseph, Charity Ehimwenma Joshua, Jiseung Kang, Kehinde Kazeem Kanmodi, Mehrdad Karajizadeh, Jafar Karami, Faizan Zaffar Kashoo, Inn Kynn Khaing, Himanshu Khajuria, Mariam Khalil, Iqra Hamid Khan, Ramsha Mushtaq Khan, Shaghayegh Khanmohammadi, Sameer Uttamaro Khasbage, Khalid A Kheirallah, Samira Khoshvaght, Mahmood Khosrowjerdi, Jagdish Khubchandani, Jinho Kim, Kwanghyun Kim, Felicia Marie Knaul, Ann Kristin Skrindo Knudsen, Elizabeth Koomson-Yalley, Irene Akwo Kretchy, Kewal Krishan, Barthelemy Kuate Defo, Mohammed Kuddus, Ilari Kuitunen, Mukhtar Kulimbet, Dewesh Kumar, G Anil Kumar, Kamal Kumar, Manasi Kumar, Rakesh Kumar, Vijay Kumar, Abigail Kusi Amponsah, Asep Kusnali, John Paul Kuwornu, Frank Kyei-Arthur, Pallavi L C, Lucie Laflamme, Chandrakant Lahariya, Timo Lajunen, Berthold Langguth, Anne-Marie Laslett, Zohra S Lassi, Areeba Latif, Saheed Akinmayowa Lawal, Aliyu Lawan, Seung Won Lee, Cheru Tesema Leshargie, Jie Li, Zhihui Li, Jue Liu, Xuefeng Liu, Erand Llanaj, Arianna Maever Loreche, Kevin Sheng-Kai Ma, Zheng Feei Ma, Farzan Madadizadeh, Aurea Marilia Madureira-Carvalho, Nozad Hussein Mahmood, Abdelrahman M Makram, Trisha Mallick, Deborah Carvalho Malta, Lokesh Manjani, Joemer C Maravilla, Sammer Marzouk, Clara N Matei, Yasith Mathangasinghe, Khurshid A Mattoo, Pallab K Maulik, Ikechukwu Innocent Mbachu, Susan A McLaughlin, Steven M McPhail, Hadush Negash Meles, Walter Mendoza, Ritesh G Menezes, Endalkachew Worku Mengesha, Tomislav Mestrovic, Sachith Mettananda, Sandrine Donfack D Mewoabi, Andrea Michelerio, Ted R Miller, Giuseppe Minervini, Mojgan Mirghafourvand, Chaitanya Mittal, Mona Gamal Mohamed, Nouh Saad Mohamed, Khabab Abbasher Hussien Mohamed Ahmed, Sakineh Mohammad-Alizadeh-Charandabi, Abdollah Mohammadian-Hafshejani, Shafiu Mohammed, Ali H Mokdad, Hossein Molavi Vardanjani, Lorenzo Monasta, Yousef Moradi, Rafael Silveira Moreira, Rohith Motappa, Kimia Mozahheb Yousefi, Sumaira Mubarik, Oscar J Mujica, Erin C Mullany, Christopher J L Murray, Woojae Myung, Karikalan Nagarajan, Shumaila Nargus, Abdulqadir J Nashwan, Mahmoud Nassar, Samidi Nirasha Kumari Navaratna, Biswa Prakash Nayak, Shalini Ganesh Nayak, Athare Nazri-Panjaki, Anthony Wainaina Ndungu, Finiki Nearchou, Amanuel Tebabal Nega, Ionut Negoi, Cao Duy Nguyen, Cuong Tat Nguyen, Huong Lan Thi Nguyen, Long Nguyen, Syed Toukir Ahmed Noor, Mamoona Noreen, Amir Norouzy, Chisom Adaobi Nri-Ezedi, Chijindu N Nwakama, Felix Kwasi Nyande, Erin M O'Connell, Osaretin Christabel Okonji, John Olayemi Okunlola, Oluwaseyi Isaiah Olabisi, Comfort Z Olorunsaiye, Sandersan Onie, Obinna E Onwujekwe, Atakan Orscelik, Esteban Ortiz-Prado, Augustus Osborne, Uchechukwu Levi Osuagwu, Amel Ouyahia, Mahesh P A, Jagadish Rao Padubidri, Raul Felipe Palma-Alvarez, Ioannis Pantazopoulos, Romil R Parikh, Arpit Parmar, Ava Pashaei, Maja Pasovic, Jay Patel, Sangram Kishor Patel, Shankargouda Patil, Shrikant Pawar, Shubhadarshini Pawar, Prince Peprah, Ramesh Poluru, Naeimeh Pourtaheri, Jalandhar Pradhan, Elton Junio Sady Prates, Shuby Puthussery, Jagadeesh Puvvula, Ibrahim Qattea, Xiang Qi, Zhipeng Qi, Yanan Qiao, Kabir Ayobami Raheem, Vafa Rahimi-Movaghar, Md Mosfequr Rahman, Mosiur Rahman, Muhammad Aziz Rahman, Ivano Raimondo, Sathish Rajaa, Pushp Lata Rajpoot, Mahmoud Mohammed Ramadan, Sheena Ramazanu, Chhabi Lal Ranabhat, Sowmya J Rao, Devarajan Rathish, Santosh Kumar Rauniyar, Mohsen Rezaeian, Taeho Gregory Rhee, Jefferson Antonio Buendia Rodriguez, Leonardo Roever, Luca Ronfani, Moustaq Karim Khan Rony, Allen Guy Patrick Ross, Hanieh Rouzbahani, Shiva Rouzbahani, Priyanka Roy, Sharmistha Roy, Robert Rudolf, Cameron John Sabet, Mohd Saeed, Umar Saeed, Rajesh Sagar, Dominic Sagoe, Pragyan Monalisa Sahoo, S Mohammad Sajadi, Dauda Salihu, Sonia Sameen, Abdallah M Samy, Damian F Santomauro, Milena M Santric-Milicevic, Tanmay Sarkar, Gargi Sachin Sarode, Sachin C Sarode, Maheswar Satpathy, Monika Sawhney, Siddharthan Selvaraj, Yashendra Sethi, Muhammad Shahab,

Samiah Shahid, Moyad Jamal Shahwan, Masood Ali Shaikh, Nafhat Shaikh, Anas Shamsi, Alfiya Shamsutdinova, Dan Shan, Mohd Shanawaz, Mohammed Shannawaz, Vishal Sharma, Mahabalesh Shetty, Premalatha K Shetty, Wenming Shi, Md Monir Hossain Shimul, Rahman Shiri, Aminu Shittu, Ivy Shiue, Seyed Afshin Shorofi, Emmanuel Edwar Siddig, Gustavo Correia Basto da Silva, Akanksha Singh, Baljinder Singh, Marco Aurelio Sousa, Muhammad Haroon Stanikzai, Caroline Stein, Dan J Stein, Brendon Stubbs, Vetriselvan Subramaniyan, Mahwish Suhaib, Mark J M Sullman, Jing Sun, Chandan Kumar Swain, Sree Sudha T Y, Rafael Tabarés-Seisdedos, Seyyed Mohammad Tabatabaei, Celine Tabche, Mircea Tampa, Minale Tareke, Sarvenaz Taridashti, Anika Tasnim, Mohamad-Hani Temsah, Azimeraw Arega Tesfu, Rekha Thapar, Muthu Thiruvengadam, Wei Tian, Marcos Roberto Tovani-Palone, Tam Quoc Minh Tran, Thang Huu Tran, Samuel Joseph Tromans, Claudia Truppa, Alexander C Tsai, Evangelia Eirini Tsermpini, Aisha Twalibu, Bhaskaran Unnikrishnan, Zahir Vally, Nadia Machado Vasconcelos, Aliscia Vieira, David Villarreal-Zegarra, Manish Vinayak, Theo Vos, Yasir Waheed, Megha Walia, Qingzhi Wang, Wei Wang, Yuan-Pang Wang, Nuwan Darshana Wickramasinghe, Martin Wiredu Agyekum, Wanqing Xie, Wanqing Xu, Saba Yahoo (Syed), Guangcan Yan, Yuqi Yang, Pengpeng Ye, Renjulal Yesodharan, Siyan Yi, Yazachew Engida Yismaw, Dong Keon Yon, Naohiro Yonemoto, Chuanhua Yu, Umar Yunusa, Siddhesh Zadey, Giulia Zamagni, Hussaini Zandam, Mohammed G M Zeariya, Alemu Birara Zemariam, Beijian Zhang, Haijun Zhang, Abzal Zhumagaliuly, Mikhail Zinchuk, Mohamed Ali Zoromba, Sa'ed H Zyoud, Emmanuela Gakidou,

Affiliations

Institute for Health Metrics and Evaluation (L S Flor PhD, C N Spencer BA, J Cagney MSc, G F Gil MPH, A Y Aravkin PhD, A Arrieta PhD, X Dai PhD, Prof R Dandona PhD, E M DeGraw MPH, Prof S I Hay FMedSci, M E Herbert MSc, S A McLaughlin PhD, T Mestrovic PhD, Prof A H Mokdad PhD, E C Mullany MBA, Prof C J L Murray DPhil, E M O'Connell BA, M Pasovic MEd, D F Santomauro PhD, C Stein PhD, Prof T Vos PhD, Prof E Gakidou PhD), Department of Health Metrics Sciences, School of Medicine (L S Flor PhD, A Y Aravkin PhD, X Dai PhD, Prof R Dandona PhD, Prof S I Hay FMedSci, Prof A H Mokdad PhD, Prof C J L Murray DPhil, C Stein PhD, A Twailbu MS, Prof T Vos PhD, Prof E Gakidou PhD), Department of Applied Mathematics (A Y Aravkin PhD), Department of Global Health (M Khalil BA), University of Washington, Seattle, WA, USA; Department of Nursing (H Aalruz PhD), Al Zaytoonah University of Jordan, Amman, Jordan; Department of Epidemiology (S Abd ElHafeez DrPH), Alexandria University, Alexandria, Egypt; Health Research Centre (Prof S I Abdelwahab PhD), Department of Public Health (S Dohare MD, P Rajpoot PhD), Department of Prosthetic Dental Sciences (K A Mattoo MD), College of Nursing and Health Sciences (M Shanawaz MD), Jazan University, Jazan, Saudi Arabia; Department of Medicine (Prof M Abdoun PhD), Department of Health (Prof M Abdoun PhD), University of Setif Algeria, Sétif, Algeria; Department of Midwifery (M Abebe MSc, Y Abebe MSc), School of Public Health (H Hareru MSc), Dilla University, Dilla, Ethiopia; Department of Emergency Medicine (A Abedi MD), Department of Immunology (S Athari PhD), Department of Critical Care and Emergency Nursing (N Hanifi PhD), Zanjan University of Medical Sciences, Zanjan, Iran; Postgraduate Department (Prof R Abeldaño Zuñiga PhD), University of Sierra Sur, Miahuatlan de Porfirio Diaz, Mexico; Yhteiskuntadatatieteen keskus (Centre for Social Data Science) (Prof R Abeldaño Zuñiga PhD), Department of Psychology (Prof T Lajunen PhD), University of Helsinki, Helsinki, Finland; Department of Midwifery (A Abie MSc, W F Balcha MSc, A T Nega MSc, A A Tesfu MSc), Department of Public Health (M Belayneh PhD), Department of Reproductive Health and Population Studies (E W Mengesha MPH), Department of Psychiatry (M Tareke MSc), Department of Pharmacology (Y E Yismaw MSc), Bahir Dar University, Bahir Dar, Ethiopia; Department of Community Medicine (Prof O Abiodun MPH), Babcock University, Ilishan-Remo, Nigeria; Department of

Family and Community Health (R G Aboagye MPH), Department of Epidemiology and Biostatistics (L A Adzigbli BSc, R K Dowou MPhil), Department of Population and Behavioural Sciences (H Amu PhD), Department of Nursing (F K Nyande PhD), University of Health and Allied Sciences, Ho, Ghana; School of Population Health (R G Aboagye MPH), The Graduate School of Biomedical Engineering (Prof H Alinejad Rokny PhD), School of Medicine (Prof P K Maulik PhD), The George Institute for Global Health (P Ye PhD), University of New South Wales, Sydney, NSW, Australia; Department of Pediatric Dentistry (Prof L Abreu PhD), School of Nursing (A da Silva PhD), Department of Maternal-Child Nursing and Public Health (Prof D C Malta PhD, E J S Prates BS), Faculty of Dentistry (Prof G C B D Silva PhD), Universidade Federal de Minas Gerais (M A Sousa PhD), Department of Preventive and Social Medicine (N M Vasconcelos MSc), Federal University of Minas Gerais, Belo Horizonte, Brazil; Clinical Pharmacy and Therapeutics Department (Prof R K Abu Farha PhD), Applied Science Private University, Amman, Jordan; Department of Pharmacology and Toxicology (B Abubakar PhD), Department of Sociology (Prof J Amzat PhD), Department of Veterinary Public Health and Preventive Medicine (A Shittu MSc), Usmanu Danfodiyo University, Sokoto, Sokoto, Nigeria; Nigerian Institute of Medical Research, Lagos, Nigeria (B Abubakar PhD); Department of Nursing (Prof S Abuhammad PhD), College of Pharmacy (S M Aleidi PhD), Department of Basic Biomedical Sciences (Prof Y Bustanji PhD), Department of Clinical Sciences (Prof M M Ramadan PhD), University of Sharjah, Sharjah, United Arab Emirates; Maternal and Child Health Nursing (Prof S Abuhammad PhD), Faculty of Medicine (Prof M S I Alyahya PhD), Department of Public Health (Prof K A Kheirallah PhD), Jordan University of Science and Technology, Irbid, Jordan; Department of Population Health (M Achore PhD), Hofstra University, Hempstead, NY, USA; Department of Diagnostic and Interventional Radiology (L C Adams PhD), Technical University of Munich, Munich, Germany; Stanford University, Palo Alto, CA, USA (L C Adams PhD); Department of Pediatrics (B O Adebiyi PhD), University of Calgary, Calgary, AB, Canada; School of Public Health (B O Adebiyi PhD), University of the Western Cape, Bellville, South Africa; Department of Immunology (K A Adedokun MSc), Roswell Park Comprehensive Cancer Center, Buffalo, NY, USA; Graduate Program Division (K A Adedokun MSc), University at Buffalo, Buffalo, NY, USA; Department of Pediatrics (O E Adegbile MD), East Tennessee State University, Johnson City, TN, USA; Center for Cardiovascular Risk Research (O E Adegbile MD), Center for Cardiovascular Risk Research, Johnson City, TN, USA; Translational Research Team (N A Adegoke PhD), Melanoma Institute Australia (N A Adegoke PhD), Faculty of Medicine and Health (D B Anderson PhD), Sydney Musculoskeletal Health (D B Anderson PhD), The University of Sydney, Sydney, NSW, Australia; Department of Family Medicine (O T Adeleke MD), College of Health Sciences (O I Olabisi PhD), Bowen University, Iwo, Nigeria; Department of Family Medicine (O T Adeleke MD), Bowen University Teaching Hospital, Ogbomoso, Nigeria; Department of Community Medicine and Primary Care (M A Adeniyi FMCPH), Federal Medical Center Abeokuta, Abeokuta, Nigeria; Slum and Rural Health Initiative Research Academy (M A Adesina BPT), Slum and Rural Health Initiative, Ibadan, Nigeria; Department of Physiotherapy (M A Adesina BPT), Department of Educational Counselling and Developmental Psychology (H O Adewuyi PhD), Department of Epidemiology and Medical Statistics (R F Afolabi PhD, A F Fagbamigbe PhD), College of Medicine (O I Olabisi PhD), University of Ibadan, Ibadan, Nigeria; Department of Educational Psychology (H O Adewuyi PhD), Department of Sociology (Prof J Amzat PhD), Department of Education Leadership and Management (J O Okunlola PhD), University of Johannesburg, Johannesburg, South Africa; Department of Public Health (Q Adnani PhD), Universitas Padjadjaran (Padjadjaran University), Bandung, Indonesia; Technical Services Directorate (A A Afolabi MPH), MSI Nigeria Reproductive Choices, Abuja, Nigeria; Department of Life Sciences (M S Afzal PhD), University of Management and Technology, Lahore, Pakistan (A Latif BS); Department of Community

Medicine (Prof S Afzal PhD), King Edward Memorial Hospital, Lahore, Pakistan; Department of Public Health (Prof S Afzal PhD), Public Health Institute, Lahore, Pakistan; Department of Public Health Sciences (W Agyemang-Duah PhD), Queen's University, Kingston, ON, Canada; School of Public Health (B O Ahinkorah MPhil), University of Technology Sydney, Sydney, NSW, Australia; College of Medicine (A Ahmad PhD), Shaqra University, Shaqra, Saudi Arabia; School of Medicine and Psychology (D Ahmad PhD), National Centre for Epidemiology and Population Health (R A Burns PhD), Australian National University, Canberra, ACT, Australia; Health Research Institute (D Ahmad PhD), University of Canberra, Canberra, NSW, Australia; School of Nursing (Prof M M Ahmad PhD), University of Jordan, Amman, Jordan; Institute of Molecular Biology and Biotechnology (A Ahmed PhD, S Shahid PhD), University Institute of Food Science and Technology (S Bashir PhD), University Institute of Public Health (I H Khan PhD, S Nargus PhD), Research Centre for Health Sciences (RCHS) (S Shahid PhD), The University of Lahore, Lahore, Pakistan; Institute of Endemic Diseases (A Ahmed PhD), Faculty of Medicine (K Mohamed Ahmed MD), Unit of Basic Medical Sciences (E E Siddig MD), University of Khartoum, Khartoum, Sudan; Pan-African One Health Institute (PAOHI), Kigali, Rwanda (A Ahmed PhD); Department of Biosciences (H Ahmed PhD), COMSATS Institute of Information Technology, Islamabad, Pakistan; College of Nursing (M S Ahmed MSc), Majmaah University, Al Majmaah, Saudi Arabia; Department of Psychology (O Ahmed MSc), University of Chittagong, Chattogram, Bangladesh; Clinical Psychology Department (E O Akin-Odanye PhD), University College Hospital, Ibadan, Ibadan, Nigeria; Faculty of Health and Behavioural Sciences (W Akosile PhD), School of Public Health (J C Maravilla PhD, D F Santomauro PhD), The University of Queensland, Brisbane, QLD, Australia; Department of Psychiatry and Mental Health (I U Akpabio MMed), SAMRC Unit on Risk and Resilience in Mental Disorders (Prof D J Stein FRCPC), University of Cape Town, Cape Town, South Africa; Western Cape Department of Health, Cape Town, South Africa (I U Akpabio MMed); School of Health and Environmental Studies (Z Alam PhD), Hamdan Bin Mohammed Smart University, Dubai, United Arab Emirates; School of Nursing (R M Al-Amer PhD), Yarmouk University, Irbid, Jordan; School of Nursing and Midwifery (R M Al-Amer PhD), Western Sydney University, Sydney, NSW, Australia; Department of Pediatric Surgery (A Alansari MD), Nursing & Midwifery Research Department (NMRD) (A J Nashwan PhD), Hamad Medical Corporation, Doha, Qatar; Department of Health Information Management and Technology (Prof T M Alanzi PhD), Department of Pathology - Forensic Medicine Division (Prof R G Menezes MD), Imam Abdulrahman Bin Faisal University, Dammam, Saudi Arabia; School of Pharmacy (S M Aleidi PhD, Prof Y Bustanji PhD), The School of Medicine (M Al-Iede MD), The University of Jordan, Amman, Jordan; School of Population Health (M B Alemu MSc, J Dunne PhD), School of Public Health (B A Dachew PhD), National Drug Research Institute (A Laslett PhD), Curtin University, Perth, WA, Australia; Department of Health Systems and Policy (M B Alemu MSc, E Dellie MPH), Department of Epidemiology (B A Dachew PhD), University of Gondar, Gondar, Ethiopia; College of Nursing (Prof F N Alhalaiqa PhD), Qatar University, Doha, Qatar; Faculty of Nursing (W T Almagharbeh PhD), University of Tabuk, Tabuk, Saudi Arabia; BRAC Institute of Governance and Development (BIGD) (M Al-Mamun MS), BRAC University, Dhaka, Bangladesh; Department of Public and Community Health (M Al-Mamun MS), Frontier University Garowe (FUG), Puntland, Somalia; Department of Medicine (J U Almazan PhD), Nazarbayev University, Astana, Kazakhstan; Department of Clinical Nursing (M M Alnaeem PhD), Al-Zaytoonah University of Jordan, Amman, Jordan; Department of Nursing (I Alrimawi PhD), Department of Medicine (C J Sabet MA), Georgetown University, Washington, DC, USA; Department of Family and Community Medicine (N Z Alshahrani MD), University of Jeddah, Jeddah, Saudi Arabia; Public Health and Community Medicine Department (Prof T T Amin MD), Cairo University, Cairo, Egypt; Department of Health and Management Sciences (S Amini PhD), Laboratory

Science Department (J Karami PhD), Khomein University of Medical Sciences, Khomein, Iran; Spiritual Health Research Center (S Amiri PhD), Baqiyatallah University of Medical Sciences, Tehran, Iran; Department of Environmental and Occupational Health (B S Anuoluwa MPH), University of Medical Sciences, Ondo, Ondo, Nigeria; Regenerative Medicine, Organ Procurement and Transplantation Multi-disciplinary Center (S Anvari MD), Department of Social Medicine and Epidemiology (A Feizkhah MD), Guilan University of Medical Sciences, Rasht, Iran; Rural Health Research Institute (A E Anyasodor PhD, Prof J Sun PhD), Charles Sturt University, Orange, NSW, Australia; Care in Long Term Conditions Research Division (J Arias de la Torre PhD), Department of Psychological Medicine (B Stubbs PhD), King's College London, London, UK; CIBER Epidemiology and Public Health (CIBERESP), Madrid, Spain (J Arias de la Torre PhD); Department of Cardiovascular, Endocrine-Metabolic Diseases and Aging (B Armocida MD), Istituto Superiore di Sanità (ISS), Rome, Italy; Department of Periodontics (D Arumuganainar PhD), Saveetha Dental College and Hospitals (G Minervini PhD, M Tovani-Palone PhD), Saveetha University, Chennai, India; Pioneer Journal of Biostatistics and Medical Research (PJBMR), Pakistan, Pakistan (T Ashraf PhD); Department of Pediatrics (B Aslam MD), Lahore General Hospital, Lahore, Pakistan; Nursing Department (Y Asri PhD), Faculty of Health Science (Y Asri PhD), Institute of Technology and Health Science RS dr Soepraoen, Malang, Indonesia; School of Medicine and Public Health (P Atorkey PhD), University of Newcastle, Newcastle, NSW, Australia; Discipline of Psychological Science (P Atorkey PhD), ACAP University College, Sydney, NSW, Australia; Hospital and Research Centre (S R Atre PhD), Dr. D. Y. Patil Vidyapeeth Pune (Deemed to be University), Pune, India; Center for Clinical Global Health Education (S R Atre PhD), School of Public Health (A A Berihun MPH), Department of Anesthesia and Critical Care Medicine (S Boppana MD), School of Medicine (C Edeh MD, C N Nwakama MD), Department of International Health (H Zhang PhD), Johns Hopkins University, Baltimore, MD, USA; College of Medicine and Health Sciences (A H Atsbaha MPH), Adigrat University, Adigart, Ethiopia; Management Policy and Community Health (J A Atta MPH, M Iqbal MPH), University of Texas, Houston, TX, USA; Department of Social Welfare (M S Atteraya PhD), Keimyung University, Daegu, South Korea; ASIDE Healthcare, Lewes, DE, USA (A Y Azzam MD); Faculty of Medicine (A Y Azzam MD), The Orthopaedic Department (A M Makram MD), October 6 University, 6th of October City, Egypt; School of Public Health (S B MPH), JSS Academy of Higher Education and Research, Mysuru, India; Pediatric Dentistry Department (K K Baghlaf PhD), King Abdulaziz University, Jeddah, Saudi Arabia; International Medical School (A A Baig PhD), Management and Science University, Alam, Malaysia; Anahuac Business School (J Balmori-de-la-Miyar PhD), Universidad Anahuac Mexico, Mexico City, Mexico; Nuffield Department of Surgical Sciences (S Bandyopadhyay MPH), University of Oxford, Oxford, UK; Department of Neurosurgery (S Bandyopadhyay MPH), University of Southampton, Southampton, UK; Global Health Research Department (M Barik MPH), IMPACT Global Consulting, New Delhi, India; Foundation for Reproductive Health Services India, New Delhi, India (M Barik MPH); School of Psychology (Prof S L Barker-Collo PhD), University of Auckland, Auckland, New Zealand; Department of Community and Family Medicine (M Bashar MD), All India Institute of Medical Sciences, Gorakhpur, India; Health Information Management (A Bashiri PhD), Trauma Research Center (M Karajizadeh PhD), Department of Biostatistics (Prof H Molavi Vardanjani PhD), Shiraz University of Medical Sciences, Shiraz, Iran; Non-communicable Diseases Research Center (M Bastan MD), Department of Psychiatry (R Jabbarinejad MD), Department of Immunology (J Karami PhD), School of Medicine (S Khanmohammadi MD), Sina Trauma and Surgery Research Center (Prof V Rahimi-Movaghar MD), Tehran University of Medical Sciences, Tehran, Iran; School of Medicine (M Bastan MD), Department of Medicine (A Farahani MD), Department of Ophthalmology (H Hasani MD), Antimicrobial Resistance Research Center (K Mozahheb

Yousefi MD), Hazrat-e Rasool General Hospital (K Mozahheb Yousefi MD), Iran University of Medical Sciences, Tehran, Iran; Department of Human Anatomy and Histology (Prof N Beeraka PhD), I.M. Sechenov First Moscow State Medical University, Moscow, Russia; Department of Public Health (M Belayneh PhD), Institute for Social and Health Sciences (Prof L Laflamme PhD), University of South Africa, Pretoria, South Africa; Department of Radiology (G Belge Bilgin MD), Mayo Clinic, Rochester, MN, USA; School of the Environment (Prof M L Bell PhD), Department of Genetics (S Pawar PhD), Department of Psychiatry (T Rhee PhD), Yale University, New Haven, CT, USA; School of Health Policy and Management (Prof M L Bell PhD), School of Health and Environmental Science (Prof J Kang PhD), Department of Health Policy and Management (Prof J Kim PhD), College of International Studies (R Rudolf PhD), Korea University, Seoul, South Korea; Department of Epidemiology and Biostatistics (A C Bermudez PhD), National Institutes of Health (A Loreche MS), University of the Philippines Manila, Manila, Philippines; Family Medicine Department (A Bhatnagar MD), Texas Tech University, El Paso, TX, USA; Program Management Department (A H Bhattarai MPH), Emotional Well-Being Institute Canada, Burnaby, BC, Canada; Department of Biochemistry and Biotechnology (M Biswas PhD), University of Science and Technology Chittagong, Chittagong, Bangladesh; Department of Community Medicine and Global Health (Prof E Bjertness PhD), University of Oslo, Oslo, Norway; Department of Global Healthcare Management (O A Bolarinwa PhD), York St John University, London, UK; Department of Demography and Population Studies (O A Bolarinwa PhD), University of the Witwatersrand, Johannesburg, South Africa; Isfahan University of Medical Sciences, Isfahan, Iran (P Bolourinejad MD); Facultad de Salud (Faculty of Health) (Prof A Botero Carvajal PhD), Universidad Santiago de Cali, Cali, Colombia; Department of Medicine (Prof S Bouaoud DrPH), Faculty of Medicine (Prof A Ouyahia PhD), University Ferhat Abbas of Setif, Setif, Algeria; Department of Epidemiology and Preventive Medicine (Prof S Bouaoud DrPH), University Hospital Saadna Abdenour, Setif, Algeria; Department of Health Sciences (Prof T Brugha MD), Division of Public Health and Epidemiology (S J Tromans PhD), University of Leicester, Leicester, UK; Department of Woman and Child Health and Public Health (D Buonsenso MD), Fondazione Policlinico Universitario A. Gemelli IRCCS (Agostino Gemelli University Polyclinic IRCCS), Rome, Italy; Global Health Research Institute (D Buonsenso MD), Università Cattolica del Sacro Cuore (Catholic University of Sacred Heart), Rome, Italy; Department of Medicine and Surgery (A Carugno PhD), University of Insubria, Varese, Italy; IMPInstitute for Mental and Physical Health and Clinical Translation (IMPACT) (A F Carvalho MD), Deakin University, Geelong, VIC, Australia; Research Unit on Applied Molecular Biosciences (UCIBIO) (Prof F Carvalho PhD), UCIBIO Applied Molecular Biosciences Unit (Prof D Dias da Silva PhD), University of Porto, Porto, Portugal; Department of Psychiatry (Prof J Castaldelli-Maia PhD, Y Wang PhD), University of São Paulo, São Paulo, Brazil; Institute of Clinical Physiology (S Cerrai MSc), Italian National Council of Research, Pisa, Italy; Department of Applied Health Sciences (Prof J S Chandan PhD), University of Birmingham, Birmingham, UK; Department of Psychiatry (Prof M Chandradasa MD), Department of Paediatrics (Prof S Mettananda DPhil), University of Kelaniya, Ragama, Sri Lanka; University Psychiatry Unit (Prof M Chandradasa MD), University Paediatrics Unit (Prof S Mettananda DPhil), Colombo North Teaching Hospital, Ragama, Sri Lanka; Department of Public Health (P Charalampous PhD), Erasmus University Medical Center, Rotterdam, Netherlands; Department of Epidemiology and Biostatistics (V Chattu PhD), Semey Medical University (SMU), Semey, Kazakhstan; Department of Community Medicine (V Chattu PhD), Datta Meghe Institute of Medical Sciences, Sawangi, India; Department of Biology (A A Chaudhary PhD), Imam Mohammad Ibn Saud Islamic University, Riyadh, Saudi Arabia; Department of Public Health (S Chaudhuri MD), Indian Institute of Public Health, Hyderabad, India; Clinical Project Management Office (H Chen MPH), National Clinical Research Center for Infectious Diseases, Shenzhen,

Shenzhen, China; Division of Infectious Diseases (P R Ching MD), Virginia Commonwealth University, Richmond, VA, USA; Centre for Research Impact & Outcome (H Chopra PhD, S Khoshvaght MSc), Chitkara University, Rajpura, India; Department of Mathematical Sciences (C Chukwu PhD), Georgia Southern University, Statesboro, GA, USA; Department of Paediatric Surgery (I S Chukwu BMedSc), Federal Medical Centre, Umuahia, Nigeria; Department of Health Behavior (S Chung MPH), Texas A&M University, College Station, TX, USA; School of Population Health (P Cullen PhD), University of New South Wales, Kensington, NSW, Australia; Global Women's Health Program (P Cullen PhD), The George Institute for Global Health, Newtown, NSW, Australia; Department of Health (O Dadras PhD), Northern Territory Government, Darwin, SA, Australia; Institute for Health Sciences (Prof K Dalal PhD), Mid Sweden University, Sundsvall, Sweden; Public Health Foundation of India, Gurugram, India (Prof R Dandona PhD, G Kumar PhD); Department of Brain Sciences (L D'Anna PhD), School of Public Health (A M Makram MD), Department of Primary Care and Public Health (C Tabche MSc), Imperial College London, London, UK; Department of Public Health (S D Darcho MPH), Haramaya University, Harar, Ethiopia; Department of Planning Monitoring and Evaluation (H Dayal PhD), The Presidency Office, Pretoria, South Africa; Pan-African Collective for Evidence, Johannesburg, South Africa (H Dayal PhD); Public Health and Environment Research Centre (PERC) in Nepal, Lalitpur, Nepal (K Deuba DrPH); Global Health Epidemiology Research Group (K Deuba DrPH), Department of Psychosocial Science (Prof D Sagoe PhD), University of Bergen, Bergen, Norway; Department of Pharmacy (S Dewan PhD), United International University, Dhaka, Bangladesh; Pharmacology Division (S Dewan PhD), Center for Life Sciences Research Bangladesh, Dhaka, Bangladesh; Escola Superior de Saúde (Higher School of Health) (Prof D Dias da Silva PhD), Instituto Politécnico do Porto (Polytechnic Institute of Porto), Porto, Portugal; Public Health Intelligence Unit (Prof D Diaz PhD), National Institute of Public Health, Cuernavaca, Mexico; School of Sociology (E W Dumbili PhD), School of Psychology (F Nearchou PhD), University College Dublin, Dublin, Ireland; Department of Psychiatry (E Eboreime PhD, E Tsermpini PhD), Dalhousie University, Halifax, NS, Canada; Department of Psychiatry (E Eboreime PhD), Faculty of Nursing (U Yunusa PhD), University of Alberta, Edmonton, AB, Canada; School of Health Sciences (H A Edinur PhD), Universiti Sains Malaysia (University of Science Malaysia), Kubang Kerian, Malaysia; Faculty of Science and Health (M Ekholuenetale PhD), University of Portsmouth, Hampshire, UK; Department of Forensic Medicine and Clinical Toxicology (Prof D A W El Morsi MD), Department of Cardiology (Prof M M Ramadan PhD), Faculty of Nursing (M Zoromba PhD), Mansoura University, Mansoura, Egypt; Department of Medical Education (Prof D A W El Morsi MD), Delta University for Science and Technology, Mansoura, Egypt; Wassa Amenfi East Municipal Health Directorate (G Eshun BSc), Ghana Health Service, Wassa Akropong, Ghana; Research Centre for Healthcare and Community (A F Fagbamigbe PhD), Coventry University, Coventry, UK; Department of Public Health Sciences (Q Fan DrPH), Clemson University, Clemson, SC, USA; Department of Psychology (A Faro PhD), Federal University of Sergipe, São Cristóvão, Brazil; Satcher Health Leadership Institute (A O Fasanmi PhD), Morehouse School of Medicine, Atlanta, GA, USA; School of Medicine (A O Fasanmi PhD), Emory University, Atlanta, GA, USA; Department of Social Sciences (Prof N Ferreira PhD, Prof M J M Sullman PhD), Department of Life and Health Sciences (Prof M J M Sullman PhD), University of Nicosia, Nicosia, Cyprus; College of Medicine, Dentistry and Public Health (Prof R C Franklin PhD), James Cook University, Townsville, QLD, Australia; Childlight – Global Child Safety Institute (D A Fry PhD), University of Edinburgh, Edinburgh, UK; Department of Biostatistics (Prof X Gao PhD), Key Lab of Environment and Health (Prof X Gao PhD), School of Public Health (Q Wang PhD, Prof W Wang PhD), Xuzhou Medical University, Xuzhou, China; Department of Midwifery (M W Gebregergis MSc), Department of Medical

Laboratory Sciences (H N Meles MSc), Adigrat University, Adigrat, Ethiopia; Environmental Pollution Monitoring and Study Desk (M Gebrehiwot DSc), Ethiopian Environmental Protection Authority, Addis Ababa, Ethiopia; Department of Midwifery (Y F Geda MSc), Wolkite University, Wolkite, Ethiopia; School of Public Health (M Gelchu MPH), Bule Hora University, Bule Hora, Ethiopia; Department of Public Health (G K Getahun MPH), Menelik II Medical and Health Science College, Addis Ababa, Ethiopia; Department of Global Health Sciences (S Ghasemi Assl MD), Department of Neurosurgery (A Orscelik MD), University of California San Francisco, San Francisco, CA, USA; Department of Electrical and Computer Engineering (E Gholami PhD), University of California Davis, Davis, CA, USA; Department of Dermatology (N Gholizadeh MD), Department of Medical-Surgical Nursing (S Shorofi PhD), Mazandaran University of Medical Sciences, Sari, Iran; Obstetrics and Gynecology Department (E Ghotbi MD), Shahid Beheshti University of Medical Sciences, Tehran, Iran; Department of Pediatrics (Z S Lassi PhD), Department of Community Health Sciences (S Sameen MSc), Aga Khan University, Karachi, Pakistan (J A Gilani MD); Department of Nursing (A A Girmay MSc), Aksum University, Aksum, Ethiopia; Department of Health Systems and Policy Research (Prof M Golechha PhD), Indian Institute of Public Health, Gandhinagar, India; Department of Genetics (P Goleij MSc), Sana Institute of Higher Education, Sari, Iran; Universal Scientific Education and Research Network (USERN) (P Goleij MSc), Kermanshah University of Medical Sciences, Kermanshah, Iran; Institute of Public Health (Prof M Grivna PhD), United Arab Emirates University, Al Ain, United Arab Emirates; Department of Public Health and Preventive Medicine (Prof M Grivna PhD), Charles University, Prague, Czech Republic; Department of Epidemiology and Biostatistics (S Guan MD), Anhui Medical University, Hefei, China; Department of Community Medicine (Prof D A Gunawardane MD, Prof S N K Navaratna MD), University of Peradeniya, Kandy, Sri Lanka; Department of Toxicology (S Gupta PhD), Shriram Institute for Industrial Research, Delhi, India; Department of Community Medicine (P Halder MD), Post Graduate Institute of Medical Education and Research, Chandigarh, India; Centre for Community Medicine (P Halder MD), Department of Psychiatry (Prof R Sagar MD), All India Institute of Medical Sciences, New Delhi, India; Department of Midwifery (H M Halil MSc), College of Medicne and Health Science (H M Halil MSc), Werabe University, Werabe, Ethiopia; Sakarya University, Sakarya, Turkiye (A Hanif PhD); Research Unit (J M Haro MD), Parc Sanitari Sant Joan de Deu, Barcelona, Spain; Department of Mental Health (J M Haro MD), Carlos III Health Institute (Prof R Tabarés-Seisdedos PhD), Biomedical Research Networking Center for Mental Health Network (CiberSAM), Madrid, Spain; Department of Advanced Nursing (E M M Has PhD), Universitas Airlangga (Airlangga University), Surabaya, Indonesia; School of Nursing and Midwivery (E M M Has PhD), La Trobe University, Bundoora, VIC, Australia; Department of Zoology and Entomology (A I Hasaballah PhD, M G M Zeariya PhD), Al-Azhar University, Cairo, Egypt; Department of Pediatrics (M Hesari MD), Jacobi Medical Center, New York, NY, USA; School of Nursing and Public Health Medicine (M Hlongwa PhD), University of KwaZulu-Natal, Durban, South Africa; Eastern Africa Centre and Institute for Health & Allied Professionals (Prof M Hossain PhD), Nottingham Trent University, Nottingham, UK; Department of Decision and Information Sciences (M Hossain DrPH), University of Houston, Houston, TX, USA; Public Health Research Group (M Hossain DrPH), Nature Study Society of Bangladesh, Khulna, Bangladesh; Department of Statistics (M Hossain MSc, S Noor MS), Shahjalal University of Science and Technology, Sylhet, Bangladesh; Department of Population Sciences (Prof M B Hossain PhD), University of Dhaka, Dhaka, Bangladesh; Department of Psychological and Cognitive Sciences (C Hu PhD), Tsinghua Vanke School of Public Health (Z Li PhD), Tsinghua University, Beijing, China; Faculty of Medicine (J Huang MD), The Chinese University of Hong Kong, Hong Kong, China; Advanced Institute of Convergence Knowledge Informatics (Y Huang PhD), Graduate School of Engineering (Y Huang PhD), Tohoku

University, Sendai, Japan; Health Policy and Management Department (P M Iftikhar MD), City University of New York, New York, NY, USA; Faculty of Pharmacy (L M Irham PhD), Universitas Ahmad Dahlan, Yogyakarta, Indonesia; Independent Researcher, Cairo, Egypt (T R Iskander BSc); Faculty of Engineering and Technology (M Islam PhD), Eastern University, Dhaka, Bangladesh; Journal of Biosciences and Public Health (JBPH) (M Islam PhD), 4-Green Research Society, Dhaka, Bangladesh; Department of Nutrition (Prof S Islam PhD), Texas Tech University, Lubbock, TX, USA; Department of Physical Medicine & Rehabilitation (R Jabbarinejad MD), Medical Scientist Training Program (S Marzouk MA), Northwestern University, Chicago, IL, USA; Department of Epidemiology (B H Jena PhD), Wachemo University, Hossana, Ethiopia; Department of Community Medicine (R P Jha MSc), Dr. Baba Saheb Ambedkar Medical College & Hospital, Delhi, India; Department of Community Medicine (R P Jha MSc), Banaras Hindu University, Varanasi, India; Department of Community Medicine (N Joseph MD, R Motappa MD, R Thapar MD), Department of Forensic Medicine and Toxicology (Prof J Padubidri MD), Manipal Academy of Higher Education, Mangalore, India; Department of Economics (C E Joshua BSc), National Open University, Benin City, Nigeria; Department of Anesthesia, Critical Care and Pain Medicine (Prof J Kang PhD), Department of Psychiatry (A C Tsai MD), Massachusetts General Hospital, Boston, MA, USA; Office of the Executive Director (Prof K K Kanmodi MPH), Cephas Health Research Initiative Inc, Ibadan, Nigeria; College of Health Sciences (Prof K K Kanmodi MPH), Caleb Univeristy, Imota, Nigeria; Department of Physical Therapy and Health Rehabilitation (Prof F Z Kashoo PhD), Majmaah University, Majmaah, Saudi Arabia; Department of Public Health and Health Policy (I Khaing PhD), Hiroshima University, Hiroshima, Japan; Amity Institute of Forensic Sciences (H Khajuria PhD, B P Nayak PhD), Amity Institute of Public Health and Hospital Administration (M Shannawaz PhD, A Singh PhD), Amity University, Noida, India; Department of Community and Preventive Medicine (R Khan MD), King Edward Medical University, Lahore, Pakistan; Department of Epidemiology (S Khanmohammadi MD), Non-Communicable Diseases Research Center (NCDRC), Tehran, Iran; Department of Pharmacology (S U Khasbage MD), All India Institute of Medical Sciences, Raipur, India; Institute of Research and Development (S Khoshvaght MSc), Institute for Global Health Innovations (C Nguyen MD), Duy Tan University, Da Nang, Vietnam; Research Department (M Khosrowjerdi PhD), University of Inland Norway, Elverum, Norway; Department of Public Health (J Khubchandani PhD, S Roy MD), New Mexico State University, Las Cruces, NM, USA; School of Medicine (Prof K Kim PhD), Creighton University, Omaha, NE, USA; Department of Medicine (F M Knaul PhD), University of California Los Angeles, Los Angeles, CA, USA; Escuela de Medicina y Ciencias de la Salud (F M Knaul PhD), Tecnológico de Monterrey, Mexico City, Mexico; Centre for Disease Burden (A S Knudsen PhD), Norwegian Institute of Public Health, Bergen, Norway; Department of Sociology and Social Work (E Koomson-Yalley PhD), Department of Public Health Nursing (A Kusi Amponsah PhD), Kwame Nkrumah University of Science and Technology, Kumasi, Ghana; School of Pharmacy (Prof I A Kretchy PhD), University of Ghana, Legon, Ghana; Department of Anthropology (Prof K Krishan PhD), Institute of Forensic Science & Criminology (V Sharma PhD), Panjab University, Chandigarh, India; Department of Demography (Prof B Kuate Defo PhD), Department of Social and Preventive Medicine (Prof B Kuate Defo PhD), University of Montreal, Montreal, QC, Canada; Department of Biochemistry (Prof M Kuddus PhD), Department of Health Management (R Kumar PhD), Department of Biology (Prof M Saeed PhD), Department of Public Health (M G M Zeariya PhD), University of Hail, Hail, Saudi Arabia; Department of Pediatrics (I Kuitunen PhD), Kuopio University Hospital, Kuopio, Finland; Institute of Clinical Medicine (I Kuitunen PhD), University of Eastern Finland, Kuopio, Finland; Research and Publication Activity Division (M Kulimbet MSc), Science Department (A Shamsutdinova MD), Atchabarov Scientific-Research Institute of Fundamental and Applied Medicine (A Zhumagaliuly MD), Kazakh

National Medical University, Almaty, Kazakhstan; Center of Medicine and Public Health (M Kulimbet MSc), Asfendiyarov Kazakh National Medical University, Almaty, Kazakhstan; Department of Community Medicine (D Kumar MD), Rajendra Institute of Medical Sciences, Ranchi, India; Department of Mathematics (Prof K Kumar PhD), Amity University Haryana, Gurugram, India; Institute for Excellence in Health Equity (M Kumar PhD), Rory Meyers College of Nursing (X Qi PhD), New York University, New York, NY, USA; Department of Psychiatry (M Kumar PhD), Department of Management Science and Project Planning (A W Ndungu PhD), University of Nairobi, Nairobi, Kenya; Department of Economics (V Kumar PhD), Manipal University, Jaipur, India; National Research and Innovation Agency (BRIN), Jakarta, Indonesia (A Kusnali MA); Australian Centre for Health Services Innovation (J Kuwornu PhD, Prof S M McPhail PhD), Queensland University of Technology, Kelvin Grove, QLD, Australia; Department of Environment and Public Health (F Kyei-Arthur PhD), University of Environment and Sustainable Development, Somanya, Ghana; Kasturba Medical College, Manipal (P L C MD), Manipal College of Nursing (S Nayak PhD), Manipal College of Dental Sciences, Mangalore (Prof P K Shetty MDS), Kasturba Medical College, Mangalore (Prof B Unnikrishnan MD), Manipal Academy of Higher Education, Manipal, India; Department of Global Public Health (Prof L Laflamme PhD), Karolinska Institute, Stockholm, Sweden; Division of Evidence Synthesis (C Lahariya MD), Foundation for People-centric Health Systems, New Delhi, India; Division of Lifestyle Medicine (C Lahariya MD), Centre for Health: The Specialty Practice, New Delhi, India; Department of Psychology (Prof T Lajunen PhD), Norwegian University of Science and Technology, Trondheim, Norway; Department of Psychiatry and Psychotherapy (B Langguth MD), University of Regensburg, Regensburg, Germany; Centre for Alcohol Policy Research (A Laslett PhD), La Trobe University, Melbourne, VIC, Australia; Robinson Research Institute (Z S Lassi PhD), University of Adelaide, Adelaide, SA, Australia; Health Systems, Administration and Management (S A Lawal PhD), Babcock University, Sagamu, Nigeria; Health Services Management Programme (S A Lawal PhD), Plasma University, Mogadishu, Somalia; School of Physical Therapy (A Lawan PhD), The University of Western Ontario, London, ON, Canada; Department of Precision Medicine (Prof S Lee MD), Sungkyunkwan University, Suwon-si, South Korea; Charles Sturt University (C T Leshargie PhD), Haramaya University, Sydney, NSW, Australia; Debre Markos University, Ethiopia (C T Leshargie PhD); Global Health Research Center (Prof J Li PhD), Guangdong Academy of Medical Sciences and General Hospital, Guangzhou, China; Department of Global Health and Population (Z Li PhD), Department of Global Health and Social Medicine (S Onie PhD), Harvard Medical School (A C Tsai MD), Department of Social and Behavioral Sciences (W Xu MPH), Harvard University, Boston, MA, USA; Department of Epidemiology and Biostatistics (Prof J Liu PhD), School of Public Health (H Zhang PhD), Peking University, Beijing, China; Lerner Research Institute (Prof X Liu PhD), Cleveland Clinic, Cleveland, OH, USA; Department of Quantitative Health Science (Prof X Liu PhD), Case Western Reserve University, Cleveland, OH, USA; Department of Molecular Epidemiology (E Llanaj PhD), German Institute of Human Nutrition Potsdam-Rehbrücke, Potsdam, Germany; German Center for Diabetes Research (DZD), München-Neuherberg, Germany (E Llanaj PhD); Ateneo School of Government (A Loreche MS), Ateneo De Manila University, Quezon City, Philippines; Center for Global Health (K Ma DDS), Department of Biostatistics, Epidemiology, and Informatics (J Puvvula PhD), University of Pennsylvania, Philadelphia, PA, USA; Centre for Public Health and Wellbeing (Z Ma PhD), University of the West of England, Bristol, UK; Department of Biostatistics and Epidemiology (F Madadizadeh PhD), Yazd University of Medical Sciences, Yazd, Iran; Associate Laboratory i4HB (A M Madureira-Carvalho PhD), University Institute of Health Sciences - CESPU, Gandra, Portugal; UCIBIO Research Unit on Applied Molecular Biosciences (A M Madureira-Carvalho PhD), University Institute of Health Sciences, Gandra, Portugal; Research Center (N

H Mahmood PhD), Cihan University-Sulaimaniya, Sulaymaniyah, Iraq; Maternal and Child Health Division (T Mallick MSc), Maternal and Child Health Division (MCHD) (S Noor MS), International Centre for Diarrhoeal Disease Research, Bangladesh, Dhaka, Bangladesh; Internal Medicine Department (L Manjani MD), MedStar Health, Washington, DC, USA; Far Eastern University, Manila, Philippines (J C Maravilla PhD); Department of Dermatology (C N Matei PhD, M Tampa PhD), Department of General Surgery (I Negoi PhD), Carol Davila University of Medicine and Pharmacy, Bucharest, Romania; Board of Directors (C N Matei PhD), Association of Resident Physicians, Bucharest, Romania; Department of Anatomy and Developmental Biology (Y Mathangasinghe PhD), Monash University, Clayton, VIC, Australia; Department of Anatomy, Genetics and Biomedical Informatics (Y Mathangasinghe PhD), Postgraduate Institute of Medicine (Prof S N K Navaratna MD), University of Colombo, Colombo, Sri Lanka; Research Division (Prof P K Maulik PhD), The George Institute for Global Health, New Delhi, India; Department of Obstetrics and Gynaecology (Prof I I Mbachu FWACS), Department of Paediatrics (C A Nri-Ezedi PhD), Nnamdi Azikiwe University, Awka, Nigeria; Digital Health and Informatics Directorate (Prof S M McPhail PhD), Queensland Health, Brisbane, QLD, Australia; Dirección General de Investigación, Desarrollo e Innovación (DGIDI) (W Mendoza MD), Universidad Científica del Sur (University of the South), Lima, Peru; University Centre Varazdin (T Mestrovic PhD), University North, Varazdin, Croatia; Faculty of Sciences (S D D Mewoabi PhD), University of Buea, Cameroon, Buea, Cameroon; Dermatology Unit (A Michelerio PhD), Fondazione IRCCS Policlinico San Matteo, Pavia, Italy; College of Human Medicine (T R Miller PhD), Michigan State University, Flint, MI, USA; Multidisciplinary Department of Medical-Surgical and Dental Specialties (G Minervini PhD), University of Campania Luigi Vanvitelli, Naples, Italy; Faculty of Nursing and Midwifery (Prof M Mirghafourvand PhD), Social Determinants of Health Research Center (Prof S Mohammad-Alizadeh-Charandabi PhD), Midwifery Department (Prof S Mohammad-Alizadeh-Charandabi PhD), Tabriz University of Medical Sciences, Tabriz, Iran; Department of Forensic Medicine and Toxicology (C Mittal MD), All India Institute of Medical Sciences, Patna, India; RAK College of Nursing (M Mohamed PhD), RAK Medical and Health Sciences University, Ras Alkhima, United Arab Emirates; Nursing College (M Mohamed PhD), Sohag University, Sohag, Egypt; Molecular Biology Unit (N S Mohamed MSc), Bio-Statistical and Molecular Biology Department (N S Mohamed MSc), Sirius Training and Research Centre, Khartoum, Sudan; Modeling in Health Research Center (A Mohammadian-Hafshejani PhD), Shahrekord University of Medical Sciences, Shahrekord, Iran; Health Systems and Policy Research Unit (Prof S Mohammed PhD), Ahmadu Bello University, Zaria, Nigeria; Heidelberg Institute of Global Health (HIGH) (Prof S Mohammed PhD), Heidelberg University, Heidelberg, Germany; Clinical Epidemiology and Public Health Research Unit (L Monasta DSc, L Ronfani PhD, G Zamagni MSc), Burlo Garofolo Institute for Maternal and Child Health, Trieste, Italy; Department of Epidemiology and Biostatistics (Y Moradi PhD), Kurdistan University of Medical Sciences, Sanandaj, Iran; Department of Public Health (Prof R S Moreira PhD), Oswaldo Cruz Foundation, Recife, Brazil; Department of Public Health (Prof R S Moreira PhD), Federal University of Pernambuco, Recife, Brazil; Unit of Pharmacotherapy, Epidemiology and Economics (Prof S Mubarik PhD), University of Groningen (Rijksuniversiteit Groningen), Groningen, Netherlands; Department of Epidemiology and Biostatistics (Prof S Mubarik PhD, Prof C Yu PhD), Wuhan University, Wuhan, China; Department of Evidence and Intelligence for Action in Health (O J Mujica MD), Pan American Health Organization, Washington, DC, USA; Department of Psychiatry (W Myung PhD), Seoul National University, Seoul, South Korea; Department of Neuropsychiatry (W Myung PhD), Seoul National University Bundang Hospital, Seongnam, South Korea; ICMR-National Institute for Research in Tuberculosis (K Nagarajan PhD), Indian Council of Medical Research, Chennai, India; Division of Endocrinology and Diabetes (M Nassar PhD),

University of Vermont, South Burlington, VT, USA; Department of Health Promotion (A Nazri-Panjaki MSc), Zahedan University of Medical Sciences, Zahedan, Iran; Department of General Surgery (I Negoi PhD), Emergency University Hospital Bucharest, Bucharest, Romania; Institute for Global Health Innovations (C T Nguyen MPH, H L T Nguyen MPH), Duy Tan University, Hanoi, Vietnam; Faculty of Public Health (L Nguyen PhD), International Institute for Training and Research (INSTAR) (L Nguyen PhD), VNU University of Medicine and Pharmacy, Hanoi, Vietnam; Department of Microbiology and Molecular Genetics (M Noreen PhD), The Women University Multan, Multan, Pakistan; Bioprocess Engineering Department (Prof A Norouzy PhD), National Institute of Genetic Engineering and Biotechnology, Tehran, Iran; School of Pharmacy (O C Okonji MSc), University of the Western Cape, Cape Town, South Africa; Department of Public Health (C Olorunsaiye PhD), Arcadia University, Glenside, PA, USA; Wellspring Research (S Onie PhD), Wellspring Center Indonesia, Jakarta, Indonesia; Department of Pharmacology and Therapeutics (Prof O E Onwujekwe PhD), University of Nigeria Nsukka, Enugu, Nigeria; One Health Global Research Group (Prof E Ortiz-Prado PhD), Universidad de las Americas (University of the Americas), Quito, Ecuador; Department of Biological Sciences (A Osborne MSc), Njala University, Freetown, Sierra Leone; School of Medicine (U L Osuagwu PhD), Western Sydney University, Bathurst, NSW, Australia; Department of Optometry and Vision Science (U L Osuagwu PhD), University of KwaZulu-Natal, KwaZulu-Natal, South Africa; Division of Infectious Diseases (Prof A Ouyahia PhD), University Hospital of Setif, Setif, Algeria; Department of Respiratory Medicine (Prof M P A DNB), Jagadguru Sri Shivarathreeswara University, Mysore, India; Department of Mental Health (R Palma-Alvarez PhD), Hospital Universitari Vall d'Hebron (CIBERSAM), Barcelona, Spain; Biomedical Network Research Centre on Mental Health (CIBERSAM), Barcelona, Spain (R Palma-Alvarez PhD); Department of Emergency Medicine (Prof I Pantazopoulos PhD), University of Thessaly, Larissa, Greece; Department of Emergency Medicine (Prof I Pantazopoulos PhD), University of Bern, Bern, Switzerland; Division of Health Policy and Management (R R Parikh MD), University of Minnesota, Minneapolis, MN, USA; Department of Psychiatry (A Parmar DM), All India Institute of Medical Sciences, Bhubaneswar, India; School of Nursing (A Pashaei MSc), University of British Columbia, Vancouver, BC, Canada; Faculty of Medicine and Health (J Patel MChD), University of Leeds, Leeds, UK; Department of Research and Training (S K Patel PhD), Population Council Institute, New Delhi, India; College of Dental Medicine (Prof S Patil PhD), Roseman University of Health Sciences, South Jordan, UT, USA; Department of Interventional Cardiology (S Pawar MD), Cedars Sinai Medical Center, Los Angeles, CA, USA; Australian Institute of Health Innovation (P Peprah MSc), Macquarie University, Sydney, NSW, Australia; Department of Data Management and Analysis (R Poluru PhD), The INCLEN Trust International, New Delhi, India; Non-communicable Diseases Research Center (N Pourtaheri PhD), Bam University of Medical Sciences, Bam, Iran; Department of Humanities and Social Sciences (Prof J Pradhan PhD), National Institute of Technology Rourkela, Rourkela, India; Maternal and Child Health Research Centre (S Puthussery PhD), University of Bedfordshire, Luton, UK; Department of Neonatology (I Qattea MD), Case Western Reserve University, Akron, OH, USA; School of Public Health (Prof Z Qi PhD), Xuzhou Medical University (徐州医科大学公共卫生学院), Xuzhou, China; Department of Epidemiology (Y Qiao MD), Shandong University, Jinan, China; Faculty of Veterinary Medicine (Prof K A Raheem PhD), University of Ilorin, Ilorin, Nigeria; Department of Population Science and Human Resource Development (Prof M Rahman PhD, Prof M Rahman DrPH), University of Rajshahi, Rajshahi, Bangladesh; Institute of Health and Wellbeing (Prof M Rahman PhD), Federation University Australia, Berwick, VIC, Australia; Department of Medical, Surgical and Experimental Sciences (I Raimondo MD), University of Sassari, Sassari, Italy; Gynecology and Breast Care Center (I Raimondo MD), Mater Olbia Hospital, Olbia, Italy;

Department of Community Medicine (S Rajaa MD), Employees' State Insurance Model Hospital, Chennai, India; School of Nursing & Health Sciences (S Ramazanu PhD), Hong Kong Metropolitan University, Hong Kong, China; Saw Swee Hock School of Public Health (S Ramazanu PhD, Prof S Yi PhD), National University of Singapore, Singapore, Singapore; Department of Research (C L Ranabhat PhD), Eastern Scientific LLC, Richmond, KY, USA; Planetary Health Research Centre (PHRC), Kathmandu, Nepal (C L Ranabhat PhD); Department of Oral Pathology, Microbiology and Forensic Odontology (S J Rao MDS), Sharavathi Dental College and Hospital, Shimogga, India; Department of Family Medicine (Prof D Rathish PhD), Department of Community Medicine (N Wickramasinghe MD), Rajarata University of Sri Lanka, Anuradhapura, Sri Lanka; Department of Global Health Policy (S K Rauniyar PhD), University of Tokyo, Tokyo, Japan; Department of Epidemiology and Biostatistics (Prof M Rezaeian PhD), Rafsanjan University of Medical Sciences, Rafsanjan, Iran; Department of Public Health Sciences (T Rhee PhD), University of Connecticut, Farmington, CT, USA; Department of Pharmacology and Toxicology (Prof J A B Rodriguez PhD), University of Antioquia, Medellin, Colombia; Warwick Medical School (Prof J A B Rodriguez PhD), University of Warwick, Coventry, UK; Department of Clinical Research (Prof L Roever PhD), University of Sao Paulo, Ribeirão Preto, Brazil; Gilbert and Rose-Marie Chagoury School of Medicine (Prof L Roever PhD), Lebanese American University, Beirut, Lebanon; Miyan Research Institute (M Rony MPH), International University of Business Agriculture and Technology, Dhaka, Bangladesh; College of Medicine (Prof A G P Ross MD), Center for Medical and Bio-Allied Health Sciences Research (Prof M J Shahwan PhD, A Shamsi PhD), Ajman University, Ajman, United Arab Emirates; Isfahan University of Medical Sciences (H Rouzbahani MD), Islamic Azad University, Isfahan, Iran; Department of Ophthalmology (S Rouzbahani MD), University of Miami, Miami, FL, USA; Department of Labour (P Roy PhD), Government of West Bengal, Kolkata, India; Faculty of Education (R Rudolf PhD), University of Canterbury, Christchurch, New Zealand; Széchenyi István University, Győr, Hungary (Prof U Saeed PhD); Operational Research Center in Healthcare (Prof U Saeed PhD), Near East University, Cyprus, Turkiye; Department of Analytical and Applied Economics (P Sahoo MA, C Swain MPhil), UGC Centre of Advanced Study in Psychology (Prof M Satpathy PhD), Utkal University, Bhubaneswar, India; College of Pharmacy (Prof S Sajadi PhD), Al-Hadba University, Mosul, Iraq; Department of Psychiatric and Mental Health, and Community Health (D Salihu PhD), Qassim University, Buraydah, Saudi Arabia; Department of Entomology (A M Samy PhD), Medical Ain Shams Research Institute (MASRI) (A M Samy PhD), Ain Shams University, Cairo, Egypt; Queensland Centre for Mental Health Research, Wacol, QLD, Australia (D F Santomauro PhD); Faculty of Medicine (Prof M M Santric-Milicevic PhD), School of Public Health and Health Management (Prof M M Santric-Milicevic PhD), University of Belgrade, Belgrade, Serbia; Department of Food Processing Technology (T Sarkar PhD), West Bengal State Council of Technical Education, Malda, India; Department of Oral Pathology and Microbiology (Prof G S Sarode PhD, Prof S C Sarode PhD), Dr. D. Y. Patil Dental College & Hospital (Prof S Selvaraj PhD), Dr. D. Y. Patil Vidyapeeth, Pune (Deemed to be University), Pune, India; Udyam-Global Association for Sustainable Development, Bhubaneswar, India (Prof M Satpathy PhD); Department of Public Health Sciences (M Sawhney PhD), University of North Carolina at Charlotte, Charlotte, NC, USA; Faculty of Dentistry (Prof S Selvaraj PhD), University of Puthisastra, Phnom Penh, Cambodia; Department of Medicine (Y Sethi MD), Swami Vivekanand Subharti University, Meerut, India; Dongguan Key Laboratory of Computer-Aided Drug Design (M Shahab PhD), Guangdong Medical University, Dongguan, China; State Key Laboratories of Chemical Resources Engineering (M Shahab PhD), Beijing University Of Chemical Technology, Beijing, China; Independent Consultant, Karachi, Pakistan (M A Shaikh MD); Department of Medicine (N Shaikh MBBS), Liaquat University Of Medical and Health Sciences, Jamshoro, Pakistan; Centre For

Interdisciplinary Research In Basic Sciences (CIRBSc) (A Shamsi PhD), Jamia Millia Islamia, New Delhi, India; Lancaster University, Lancaster, UK (D Shan PhD); Columbia University, New York, NY, USA (D Shan PhD); Alva's Insitute of Medical Sciences & Research Centre (Prof M Shetty MD), Rajiv Gandhi University of Health Sciences, Moodubidire, India; School of Public Health (W Shi PhD), University of Hong Kong, Hong Kong, China; Department of Public Health (M Shimul MPH), Daffodil International University, Dhaka, Bangladesh; Finnish Institute of Occupational Health, Helsinki, Finland (R Shiri PhD); Oulu Business School (I Shiue PhD), Martti Ahtisaari Institute (I Shiue PhD), University of Oulu, Oulu, Finland; Department of Nursing and Health Sciences (S Shorofi PhD), Flinders University, Adelaide, SA, Australia; Department of Medical Microbiology and Infectious Diseases (E E Siddig MD), Erasmus University, Rotterdam, Netherlands; Department of Biochemistry (B Singh PhD), Central University of Punjab, Bathinda, India; Department of Public Health (M Stanikzai MPH), Kandahar University, Kandahar, Afghanistan; Department of Sport (B Stubbs PhD), University of Vienna, Vienna, Austria; Department of Medical Sciences (Prof V Subramaniyan PhD), Sunway University, Subang Jaya, Malaysia; Hospital Administration (M Suhaib MHA), Sanjay Gandhi Postgraduate Institute of Medical Sciences, Lucknow, India; Hospital Administration (M Suhaib MHA), King George's Medical University, Lucknow, India; Institute of Integrated Intelligence and Systems (Prof J Sun PhD), Griffith University, Brisbane, QLD, Australia; Department of Pharmacology (S T Y MD), All India Institute of Medical Sciences, Deoghar, India; Department of Medicine (Prof R Tabarés-Seisdedos PhD), University of Valencia, Valencia, Spain; Department of Medical Informatics (S Tabatabaei PhD), Applied Biomedical Research Center (S Tabatabaei PhD), Mashhad University of Medical Sciences, Mashhad, Iran; Department of Dermato-Venereology (M Tampa PhD), Dr. Victor Babes Clinical Hospital of Infectious Diseases and Tropical Diseases, Bucharest, Romania; Department of Psychology (S Taridashti MA), Montclair State University, Montclair, NJ, USA; Department of Public Health and Informatics (A Tasnim MPH), Bangladesh Medical University, Dhaka, Bangladesh; Pediatric Intensive Care Unit (Prof M Temsah MD), King Saud University, Riyadh, Saudi Arabia; College of Medicine (Prof M Temsah MD), Alfaisal University, Riyadh, Saudi Arabia; Department of Applied Bioscience (Prof M Thiruvengadam PhD), Konkuk University, Seoul, South Korea; School of Public Health (W Tian PhD, G Yan MD), Harbin Medical University, Harbin, China; John T. Milliken Department of Medicine (T Q M Tran MSc), Washington University in St. Louis, Saint Louis, MO, USA; Department of Internal Medicine (T H Tran MD), University of Medicine and Pharmacy at Ho Chi Minh City, Ho Chi Minh City, Vietnam; Department of Business Analytics (T H Tran MD), University of Massachusetts Dartmouth, Dartmouth, MA, USA; Adult Learning Disability Service (S J Tromans PhD), Leicestershire Partnership National Health Service Trust, Leicester, UK; CRIMEDIM Center for Research and Training in Global Health, Humanitarian Aid and Disaster Medicine (C Truppa MD), University of Eastern Piedmont, Novara, Italy; Department of Primary Care (C Truppa MD), Geneva University Hospital, Geneva, Switzerland; Department of Psychology (Z Vally PhD), Zayed University, Abu Dhabi, United Arab Emirates; Department of Exact and Applied Social Sciences (A Vieira MD), Federal University of Health Science of Porto Alegre, Porto Alegre, Brazil; Digital Health Research Center (D Villarreal-Zegarra MPH), Instituto Peruano de Orientación Psicológica, Lima, Peru; Department of Biomedical Informatics (D Villarreal-Zegarra MPH), University of Utah, Salt Lake City, UT, USA; Department of Cardiology (M Vinayak MD), Icahn School of Medicine at Mount Sinai, New York, NY, USA; NUST School of Health Sciences (Prof Y Waheed PhD), National University of Science and Technology (NUST), Islamabad, Pakistan; Széchenyi István University, Gyor, Hungary (Prof Y Waheed PhD); Department of Forensic Science (M Walia MPhil), Shree Guru Gobind Singh Tricentenary University, Gurugram, India; Legon Centre for Education Research and Policy (M Wiredu Agyekum PhD), University of Ghana, Accra,

Ghana; Department of Intelligent Medical Engineering (Prof W Xie DrPH), Anhui Medical University, Anhui, China; Department of Surgery (Prof W Xie DrPH), The First Affiliated Hospital of Anhui Medical University, Hefei, Anhui, China; Department of Nutrition (W Xu MPH), Tufts University, Boston, MA, USA; Department of Community Medicine (S Yahoo (Syed) MD), Apollo Institute of Medical Sciences and Research, Hyderabad, India; Shanghai Institute of Infectious Disease and Biosecurity (Y Yang MPH), Fudan University, Shanghai, China; National Center for Chronic and Noncommunicable Disease Control and Prevention (P Ye PhD), Chinese Center for Disease Control and Prevention, Beijing, China; Manipal College of Nursing (R Yesodharan MPhil), Manipal Academy of Higher Education, Udupi, India; KHANA Center for Population Health Research, Phnom Penh, Cambodia (Prof S Yi PhD); Pharmacy Department (Y E Yismaw MSc), Alkan Health Science, Business and Technology College, Bahir Dar, Ethiopia; Department of Pediatrics (Prof D Yon MD), Kyung Hee University, Seoul, South Korea; Department of Biostatistics (Prof N Yonemoto PhD), University of Toyama, Toyama, Japan; Department of Public Health (Prof N Yonemoto PhD), Juntendo University, Tokyo, Japan; Department of Nursing Science (U Yunusa PhD), Bayero University, Kano, Nigeria; Association for Socially Applicable Research (ASAR), Pune, India (S Zadey MS); Department of Emergency Medicine (S Zadey MS), Global Emergency Medicine Innovation and Implementation (GEMINI) Research Center, Durham, NC, USA; The Heller School for Social Policy and Management (H Zandam PhD), Brandeis University, Waltham, MA, USA; Department of Pediatrics and Child Health Nursing (A Zemariam MSc), Woldia University, Woldia, Ethiopia; Department of Cardiology (B Zhang PhD), Zhongshan Hospital, Shanghai, China; Department of Suicide Research and Prevention (M Zinchuk PhD), Moscow Research and Clinical Center for Neuropsychiatry, Moscow, Russia; College of Nursing (M Zoromba PhD), Prince Sattam bin Abdulaziz University, Al-Kharj, Saudi Arabia; Department of Clinical and Community Pharmacy (Prof S H Zyoud PhD), An-Najah National University, Nablus, Palestine; Clinical Research Centre (Prof S H Zyoud PhD), An-Najah National University Hospital, Nablus, Palestine.

Authors' Contributions

Managing the overall research enterprise

Luisa S Flor, Erin C Mullany, and Emmanuela Gakidou.

Writing the first draft of the manuscript

Luisa S Flor and Cory N Spencer.

Primary responsibility for applying analytical methods to produce estimates

Jack Cagney, Luisa S Flor, and Cory N Spencer.

Primary responsibility for seeking, cataloguing, extracting, or cleaning data; designing or coding figures and tables

Jack Cagney, Gabriela Fernanda Gil, Molly E Herbert, and Cory N Spencer.

Providing data or critical feedback on data sources

Samar Abd ElHafeez, Siddig Ibrahim Abdelwahab, Armita Abedi, Roberto Ariel Abeldaño Zuñiga, Richard Gyan Aboagye, Lucas Guimarães Abreu, Bilyaminu Abubakar, Sawsan Abuhammad, Meshack Achore, Kamoru Ademola Adedokun, Oluwatobi E Adegbile, Olumide Thomas Adeleke, Habeeb Omoponle Adewuyi, Qorinah Estiningtyas Sakilah Adnani, Leticia Akua Adzigbli, Muhammad Sohail Afzal, Saira

Afzal, Williams Agyemang-Duah, Bright Opoku Ahinkorah, Muayyad M Ahmad, Asma Ahmed, Ayman Ahmed, Haroon Ahmed, Mehrunnisha Sharif Ahmed, Oli Ahmed, Wole Akosile, Rasmieh Mustafa Al-Amer, Turki M Alanzi, Montaha Al-Iede, Hamid Alinejad Rokny, Joseph Uy Almazan, Intima Alrimawi, Najim Z Alshahrani, Saeed Amini, Hubert Amu, Saeid Anvari, Jorge Arias de la Torre, Benedetta Armocida, Alejandra Arrieta, Deepavalli Arumuganainar, Bilal Aslam, Yuni Asri, Seyyed Shamsadin Athari, Prince Atorkey, Madhu Sudhan Atteraya, Ahmed Y Azzam, Sheeba B, Khlood K Baghlaf, Atif Amin Baig, Manish Barik, Shahid Bashir, Mohammad-Mahdi Bastan, Narasimha M Beeraka, Melesse Belayneh, Michelle L Bell, Arushee Bhatnagar, Espen Bjertness, Obasanjo Afolabi Bolarinwa, Sri Harsha Boppana, Souad Bouaoud, Traolach Brugha, Danilo Buonsenso, Jack Cagney, Joao Mauricio Castaldelli-Maia, Joht Singh Chandan, Periklis Charalampous, Vijay Kumar Chattu, Sirshendu Chaudhuri, Hitesh Chopra, Chidozie Williams Chukwu, Sunghyun Chung, Xiaochen Dai, Rakhi Dandona, Lucio D'Anna, Samuel Demissie Darcho, Keshab Deuba, Diana Dias da Silva, Robert Kokou Dowou, Emeka W Dumbili, Jennifer Dunne, Cynthia Edeh, Michael Ekholuenetale, Doaa Abdel Wahab El Morsi, Gilbert Eshun, Adeniyi Francis Fagbamigbe, Qiping Fan, Alireza Farahani, Alireza Feizkhah, Luisa S Flor, Richard Charles Franklin, Deborah Ann Fry, Xiang Gao, Miesa Gelchu, Shakiba Ghasemi Assl, Ehsan Gholami, Elena Ghotbi, Gabriela Fernanda Gil, Jaleed Ahmed Gilani, Alem Abera Girmay, Mahaveer Golechha, Pouya Goleij, Shi-Yang Guan, Damitha Asanga Gunawardane, Sapna Gupta, Pritam Halder, Hassen Mosa Halil, Nasrin Hanifi, Habtamu Endashaw Hareru, Josep Maria Haro, Eka Mishbahatul Marah Has, Simon I Hay, Mazeda Hossain, Md Mahbub Hossain, Mohammad Bellal Hossain, Yongsong Huang, Meesha Iqbal, Lalu Muhammad Irham, Teresa R Iskander, Md. Shahinul Islam, Sheikh Mohammed Shariful Islam, Belayneh Hamdela Jena, Charity Ehimwenma Joshua, Jiseung Kang, Faizan Zaffar Kashoo, Himanshu Khajuria, Iqra Hamid Khan, Ramsha Mushtaq Khan, Sameer Uttamaro Khasbage, Khalid A Kheirallah, Samira Khoshvaght, Jagdish Khubchandani, Jinho Kim, Felicia Marie Knaul, Ann Kristin Skrindo Knudsen, Irene Akwo Kretchy, Kewal Krishan, Barthelemy Kuate Defo, Dewesh Kumar, G Anil Kumar, Kamal Kumar, Manasi Kumar, Vijay Kumar, Asep Kusnali, Pallavi L C, Chandrakant Lahariya, Areeba Latif, Saheed Akinmayowa Lawal, Seung Won Lee, Jue Liu, Xuefeng Liu, Erand Llanaj, Kevin Sheng-Kai Ma, Zheng Feei Ma, Aurea Marilia Madureira-Carvalho, Nozad Hussein Mahmood, Deborah Carvalho Malta, Lokesh Manjani, Joemer C Maravilla, Sammer Marzouk, Clara N Matei, Ikechukwu Innocent Mbachu, Steven M McPhail, Walter Mendoza, Ritesh G Menezes, Andrea Michelerio, Ted R Miller, Chaitanya Mittal, Mona Gamal Mohamed, Nouh Saad Mohamed, Khabab Abbasher Hussien Mohamed Ahmed, Abdollah Mohammadian-Hafshejani, Shafiu Mohammed, Ali H Mokdad, Lorenzo Monasta, Yousef Moradi, Rohith Motappa, Sumaira Mubarik, Oscar J Mujica, Christopher J L Murray, Shumaila Nargus, Mahmoud Nassar, Biswa Prakash Nayak, Ionut Negoi, Cao Duy Nguyen, Cuong Tat Nguyen, Huong Lan Thi Nguyen, Long Nguyen, Mamoona Noreen, Chijindu N Nwakama, John Olayemi Okunlola, Obinna E Onwujekwe, Atakan Orscelik, Uchechukwu Levi Osuagwu, Amel Ouyahia, Mahesh P A, Jagadish Rao Padubidri, Romil R Parikh, Maja Pasovic, Sangram Kishor Patel, Shankargouda Patil, Shrikant Pawar, Shubhadarshini Pawar, Prince Peprah, Ramesh Poluru, Naeimeh Pourtaheri, Jalandhar Pradhan, Elton Junio Sady Prates, Shuby Puthussery, Jagadeesh Puvvula, Xiang Qi, Zhipeng Qi, Vafa Rahimi-Movaghar, Sathish Rajaa, Mahmoud Mohammed Ramadan, Sheena Ramazanu, Chhabi Lal Ranabhat, Sowmya J Rao, Santosh Kumar Rauniyar, Taeho Gregory Rhee, Jefferson Antonio Buendia Rodriguez, Leonardo Roever, Luca Ronfani, Moustaq Karim Khan Rony, Hanieh Rouzbahani, Shiva Rouzbahani, Priyanka Roy, Sharmistha Roy, Robert Rudolf, Cameron John Sabet, Mohd Saeed, Umar Saeed, Rajesh Sagar, Dominic Sagoe, Pragyan Monalisa Sahoo, S Mohammad Sajadi, Sonia Sameen, Abdallah M Samy, Milena M Santric-Milicevic, Tanmay Sarkar, Maheswar Satpathy, Monika Sawhney, Siddharthan Selvaraj, Yashendra Sethi, Muhammad

Shahab, Samiah Shahid, Masood Ali Shaikh, Nafhat Shaikh, Anas Shamsi, Alfiya Shamsutdinova, Dan Shan, Mohammed Shannawaz, Vishal Sharma, Mahabalesh Shetty, Premalatha K Shetty, Wenming Shi, Md Monir Hossain Shimul, Aminu Shittu, Ivy Shiue, Gustavo Correia Basto da Silva, Baljinder Singh, Marco Aurelio Sousa, Cory N Spencer, Muhammad Haroon Stanikzai, Caroline Stein, Brendon Stubbs, Vetriselvan Subramaniyan, Mahwish Suhaib, Chandan Kumar Swain, Sree Sudha T Y, Rafael Tabarés-Seisdedos, Seyyed Mohammad Tabatabaei, Celine Tabche, Mircea Tampa, Sarvenaz Taridashti, Anika Tasnim, Marcos Roberto Tovani-Palone, Tam Quoc Minh Tran, Alexander C Tsai, Bhaskaran Unnikrishnan, Zahir Vally, Aliscia Vieira, David Villarreal-Zegarra, Yasir Waheed, Megha Walia, Wanqing Xu, Pengpeng Ye, Siyan Yi, Naohiro Yonemoto, Chuanhua Yu, Siddhesh Zadey, Hussaini Zandam, Mohammed G M Zeariya, Alemu Birara Zemariam, Beijian Zhang, Abzal Zhumagaliuly, Mohamed Ali Zoromba, and Sa'ed H Zyoud.

Developing methods or computational machinery

Aleksandr Y Aravkin, Jack Cagney, Xiaochen Dai, Simon I Hay, Ali H Mokdad, Christopher J L Murray, Maja Pasovic, Cory N Spencer, and Theo Vos.

Providing critical feedback on methods or results

Samar Abd ElHafeez, Siddig Ibrahim Abdelwahab, Meriem Abdoun, Mesfin Abebe, Yonas Abebe, Armita Abedi, Roberto Ariel Abeldaño Zuñiga, Alemwork Abie, Richard Gyan Aboagye, Lucas Guimarães Abreu, Rana Kamal Abu Farha, Bilyaminu Abubakar, Sawsan Abuhammad, Meshack Achore, Lisa C Adams, Babatope Oluwadamilare Adebiyi, Kamoru Ademola Adedokun, Oluwatobi E Adegbile, Nurudeen A Adegoke, Olumide Thomas Adeleke, Miracle Ayomikun Adesina, Habeeb Omoponle Adewuyi, Qorinah Estiningtyas Sakilah Adnani, Leticia Akua Adzigbli, Aanuoluwapo Adeyimika Afolabi, Rotimi Felix Afolabi, Muhammad Sohail Afzal, Saira Afzal, Williams Agyemang-Duah, Bright Opoku Ahinkorah, Aqeel Ahmad, Danish Ahmad, Muayyad M Ahmad, Asma Ahmed, Ayman Ahmed, Haroon Ahmed, Mehrunnisha Sharif Ahmed, Oli Ahmed, Wole Akosile, Zufishan Alam, Rasmieh Mustafa Al-Amer, Amani N. Alansari, Turki M Alanzi, Shereen M Aleidi, Melaku Birhanu Alemu, Montaha Al-Iede, Hamid Alinejad Rokny, Wesam Taher Almagharbeh, Md. Al-Mamun, Joseph Uy Almazan, Intima Alrimawi, Najim Z Alshahrani, Mohammad Sharif Ibrahim Alyahya, Tarek Tawfik Amin, Saeed Amini, Sohrab Amiri, Hubert Amu, Jimoh Amzat, David B Anderson, Boluwatife Stephen Anuoluwa, Saeid Anvari, Anayochukwu Edward Anyasodor, Jorge Arias de la Torre, Benedetta Armocida, Alejandra Arrieta, Deepavalli Arumuganainar, Tahira Ashraf, Bilal Aslam, Yuni Asri, Seyyed Shamsadin Athari, Prince Atorkey, Sachin R Atre, Abadi Hailay Atsbaha, Julie Alaere Atta, Madhu Sudhan Atteraya, Ahmed Y Azzam, Sheeba B, Khlood K Baghlaf, Atif Amin Baig, Wondu Feyisa Balcha, Jose Balmori-de-la-Miyar, Manish Barik, MD Abu Bashar, Shahid Bashir, Mohammad-Mahdi Bastan, Narasimha M Beeraka, Melesse Belayneh, Gokce Belge Bilgin, Michelle L Bell, Abiye Assefa Berihun, Amiel Nazer C Bermudez, Arushee Bhatnagar, Ashmin Hari Bhattarai, Mohammad Shahangir Biswas, Espen Bjertness, Obasanjo Afolabi Bolarinwa, Paria Bolourinejad, Sri Harsha Boppana, Alejandro Botero Carvajal, Souad Bouaoud, Traolach Brugha, Danilo Buonsenso, Richard A Burns, Yasser Bustanji, Jack Cagney, Andre F Carvalho, Joao Mauricio Castaldelli-Maia, Sonia Cerrai, Joht Singh Chandan, Periklis Charalampous, Vijay Kumar Chattu, Sirshendu Chaudhuri, Haiyan Chen, Hitesh Chopra, Chidozie Williams Chukwu, Isaac Sunday Chukwu, Sunghyun Chung, Patricia Cullen, Alanna Gomes da Silva, Berihun Assefa Dachew, Omid Dadras, Xiaochen Dai, Koustuv Dalal, Rakhi Dandona, Lucio D'Anna, Samuel Demissie Darcho, Harsha Dayal, Endalkachew Dellie, Keshab Deuba, Syed Masudur Rahman Dewan, Diana Dias da Silva, Daniel Diaz, Robert Kokou Dowou, Emeka W Dumbili, Jennifer Dunne, Ejemai Eboreime, Cynthia Edeh, Hisham Atan Edinur, Michael Ekholuenetale,

Doaa Abdel Wahab El Morsi, Gilbert Eshun, Adeniyi Francis Fagbamigbe, Qiping Fan, Alireza Farahani, Andre Faro, Alireza Feizkhah, Luisa S Flor, Richard Charles Franklin, Deborah Ann Fry, Miglas Welay Gebregergis, Mesfin Gebrehiwot, Yohannes Fikadu Geda, Miesa Gelchu, Genanew K Getahun, Shakiba Ghasemi Assl, Ehsan Gholami, Elena Ghotbi, Gabriela Fernanda Gil, Jaleed Ahmed Gilani, Alem Abera Girmay, Mahaveer Golechha, Michal Grivna, Shi-Yang Guan, Damitha Asanga Gunawardane, Sapna Gupta, Pritam Halder, Hassen Mosa Halil, Asif Hanif, Nasrin Hanifi, Habtamu Endashaw Hareru, Eka Mishbahatul Marah Has, Ahmed I Hasaballah, Hamidreza Hasani, Simon I Hay, Marjan Hesari, Mbuzeleni Hlongwa, Mazeda Hossain, Md Mahbub Hossain, Md Sabbir Hossain, Mohammad Bellal Hossain, Chengxi Hu, Yongsong Huang, Meesha Iqbal, Lalu Muhammad Irham, Teresa R Iskander, Md. Shahinul Islam, Sheikh Mohammed Shariful Islam, Roxana Jabbarinejad, Belayneh Hamdela Jena, Ravi Prakash Jha, Nitin Joseph, Charity Ehimwenma Joshua, Jiseung Kang, Kehinde Kazeem Kanmodi, Mehrdad Karajizadeh, Jafar Karami, Faizan Zaffar Kashoo, Inn Kynn Khaing, Himanshu Khajuria, Iqra Hamid Khan, Ramsha Mushtaq Khan, Shaghayegh Khanmohammadi, Sameer Uttamaro Khasbage, Khalid A Kheirallah, Samira Khoshvaght, Mahmood Khosrowjerdi, Jagdish Khubchandani, Jinho Kim, Kwanghyun Kim, Felicia Marie Knaul, Ann Kristin Skrindo Knudsen, Elizabeth Koomson-Yalley, Irene Akwo Kretchy, Kewal Krishan, Barthelemy Kuate Defo, Mohammed Kuddus, Ilari Kuitunen, Dewesh Kumar, G Anil Kumar, Kamal Kumar, Manasi Kumar, Vijay Kumar, Abigail Kusi Amponsah, Asep Kusnali, John Paul Kuwornu, Frank Kyei-Arthur, Pallavi L C, Chandrakant Lahariya, Timo Lajunen, Anne-Marie Laslett, Zohra S Lassi, Areeba Latif, Saheed Akinmayowa Lawal, Aliyu Lawan, Seung Won Lee, Cheru Tesema Leshargie, Zhihui Li, Jue Liu, Xuefeng Liu, Erand Llanaj, Arianna Maever Loreche, Kevin Sheng-Kai Ma, Zheng Feei Ma, Farzan Madadizadeh, Aurea Marilia Madureira-Carvalho, Nozad Hussein Mahmood, Abdelrahman M Makram, Trisha Mallick, Deborah Carvalho Malta, Lokesh Manjani, Joemer C Maravilla, Sammer Marzouk, Clara N Matei, Yasith Mathangasinghe, Khurshid A Mattoo, Ikechukwu Innocent Mbachu, Steven M McPhail, Hadush Negash Meles, Walter Mendoza, Ritesh G Menezes, Endalkachew Worku Mengesha, Tomislav Mestrovic, Sachith Mettananda, Sandrine Donfack D Mewoabi, Andrea Michelerio, Ted R Miller, Giuseppe Minervini, Chaitanya Mittal, Mona Gamal Mohamed, Nouh Saad Mohamed, Khabab Abbasher Hussien Mohamed Ahmed, Sakineh Mohammad-Alizadeh-Charandabi, Abdollah Mohammadian-Hafshejani, Shafiu Mohammed, Ali H Mokdad, Hossein Molavi Vardanjani, Yousef Moradi, Rafael Silveira Moreira, Rohith Motappa, Kimia Mozahheb Yousefi, Sumaira Mubarik, Oscar J Mujica, Christopher J L Murray, Woojae Myung, Karikalan Nagarajan, Shumaila Nargus, Abdulqadir J Nashwan, Mahmoud Nassar, Samidi Nirasha Kumari Navaratna, Biswa Prakash Nayak, Shalini Ganesh Nayak, Athare Nazri-Panjaki, Anthony Wainaina Ndungu, Finiki Nearchou, Amanuel Tebabal Nega, Ionut Negoi, Cao Duy Nguyen, Cuong Tat Nguyen, Huong Lan Thi Nguyen, Long Nguyen, Syed Toukir Ahmed Noor, Mamoona Noreen, Chisom Adaobi Nri-Ezedi, Chijindu N Nwakama, Felix Kwasi Nyande, Osaretin Christabel Okonji, John Olayemi Okunlola, Oluwaseyi Isaiah Olabisi, Comfort Z. Olorunsaiye, Sandersan Onie, Obinna E Onwujekwe, Atakan Orscelik, Esteban Ortiz-Prado, Uchechukwu Levi Osuagwu, Amel Ouyahia, Mahesh P A, Jagadish Rao Padubidri, Ioannis Pantazopoulos, Romil R Parikh, Arpit Parmar, Maja Pasovic, Sangram Kishor Patel, Shankargouda Patil, Shrikant Pawar, Shubhadarshini Pawar, Prince Peprah, Ramesh Poluru, Naeimeh Pourtaheri, Jalandhar Pradhan, Elton Junio Sady Prates, Shuby Puthussery, Jagadeesh Puvvula, Ibrahim Qattea, Xiang Qi, Zhipeng Qi, Vafa Rahimi-Movaghar, Md. Mosfequr Rahman, Mosiur Rahman, Muhammad Aziz Rahman, Sathish Rajaa, Pushp Lata Rajpoot, Mahmoud Mohammed Ramadan, Chhabi Lal Ranabhat, Sowmya J Rao, Devarajan Rathish, Santosh Kumar Rauniyar, Mohsen Rezaeian, Taeho Gregory Rhee, Jefferson Antonio Buendia Rodriguez, Leonardo Roever, Moustaq Karim Khan Rony, Hanieh Rouzbahani, Shiva Rouzbahani, Priyanka Roy,

Sharmistha Roy, Robert Rudolf, Cameron John Sabet, Mohd Saeed, Umar Saeed, Rajesh Sagar, Dominic Sagoe, Pragyan Monalisa Sahoo, S Mohammad Sajadi, Sonia Sameen, Abdallah M Samy, Damian F Santomauro, Milena M Santric-Milicevic, Tanmay Sarkar, Gargi Sachin Sarode, Sachin C Sarode, Maheswar Satpathy, Monika Sawhney, Yashendra Sethi, Muhammad Shahab, Samiah Shahid, Masood Ali Shaikh, Nafhat Shaikh, Anas Shamsi, Dan Shan, Mohd Shanawaz, Mohammed Shannawaz, Vishal Sharma, Mahabalesh Shetty, Wenming Shi, Md Monir Hossain Shimul, Rahman Shiri, Aminu Shittu, Ivy Shiue, Seyed Afshin Shorofi, Emmanuel Edwar Siddig, Gustavo Correia Basto da Silva, Baljinder Singh, Marco Aurelio Sousa, Cory N Spencer, Muhammad Haroon Stanikzai, Caroline Stein, Brendon Stubbs, Vetriselvan Subramaniyan, Mahwish Suhaib, Mark J M Sullman, Jing Sun, Chandan Kumar Swain, Sree Sudha T Y, Rafael Tabarés-Seisdedos, Seyyed Mohammad Tabatabaei, Celine Tabche, Mircea Tampa, Minale Tareke, Sarvenaz Taridashti, Anika Tasnim, Mohamad-Hani Temsah, Azimeraw Arega Tesfu, Rekha Thapar, Muthu Thiruvengadam, Wei Tian, Marcos Roberto Tovani-Palone, Tam Quoc Minh Tran, Samuel Joseph Tromans, Claudia Truppa, Alexander C Tsai, Bhaskaran Unnikrishnan, Zahir Vally, Nadia Machado Vasconcelos, Aliscia Vieira, David Villarreal-Zegarra, Manish Vinayak, Theo Vos, Yasir Waheed, Megha Walia, Qingzhi Wang, Wei Wang, Yuan-Pang Wang, Nuwan Darshana Wickramasinghe, Martin Wiredu Agyekum, Wanqing Xie, Wanqing Xu, Saba Yahoo (Syed), Guangcan Yan, Yuqi Yang, Pengpeng Ye, Renjulal Yesodharan, Siyan Yi, Yazachew Engida Yismaw, Dong Keon Yon, Naohiro Yonemoto, Chuanhua Yu, Umar Yunusa, Siddhesh Zadey, Giulia Zamagni, Hussaini Zandam, Mohammed G M Zeariya, Alemu Birara Zemariam, Beijian Zhang, Haijun Zhang, Mikhail Zinchuk, Mohamed Ali Zoromba, and Sa'ed H Zyoud.

Drafting the work or revising it critically for important intellectual content

Hasan Aalruz, Samar Abd ElHafeez, Siddig Ibrahim Abdelwahab, Armita Abedi, Roberto Ariel Abeldaño Zuñiga, Olumide Abiodun, Lucas Guimarães Abreu, Rana Kamal Abu Farha, Bilyaminu Abubakar, Sawsan Abuhammad, Babatope Oluwadamilare Adebiyi, Kamoru Ademola Adedokun, Oluwatobi E Adegbile, Nurudeen A Adegoke, Olumide Thomas Adeleke, Makinde Adebayo Adeniyi, Habeeb Omoponle Adewuyi, Qorinah Estiningtyas Sakilah Adnani, Aanuoluwapo Adeyimika Afolabi, Rotimi Felix Afolabi, Muhammad Sohail Afzal, Saira Afzal, Bright Opoku Ahinkorah, Danish Ahmad, Muayyad M Ahmad, Asma Ahmed, Ayman Ahmed, Haroon Ahmed, Elizabeth Oluwatoyin Akin-Odanye, Wole Akosile, Idorenyin Ubon Akpabio, Zufishan Alam, Rasmieh Mustafa Al-Amer, Amani N. Alansari, Fadwa Naji Alhalaiqa, Montaha Al-Iede, Hamid Alinejad Rokny, Wesam Taher Almagharbeh, Md. Al-Mamun, Mohmmad Minwer Alnaeem, Intima Alrimawi, Najim Z Alshahrani, Mohammad Sharif Ibrahim Alyahya, Tarek Tawfik Amin, Saeed Amini, Sohrab Amiri, Hubert Amu, Jimoh Amzat, David B Anderson, Boluwatife Stephen Anuoluwa, Saeid Anvari, Anayochukwu Edward Anyasodor, Jorge Arias de la Torre, Benedetta Armocida, Deepavalli Arumuganainar, Bilal Aslam, Yuni Asri, Seyyed Shamsadin Athari, Prince Atorkey, Abadi Hailay Atsbaha, Ahmed Y Azzam, Atif Amin Baig, Jose Balmori-de-la-Miyar, Soham Bandyopadhyay, Manish Barik, Suzanne Lyn Barker-Collo, MD Abu Bashar, Shahid Bashir, Azadeh Bashiri, Mohammad-Mahdi Bastan, Melesse Belayneh, Michelle L Bell, Mohammad Shahangir Biswas, Espen Bjertness, Obasanjo Afolabi Bolarinwa, Paria Bolourinejad, Sri Harsha Boppana, Alejandro Botero Carvajal, Souad Bouaoud, Traolach Brugha, Danilo Buonsenso, Yasser Bustanji, Jack Cagney, Andrea Carugno, Andre F Carvalho, Felix Carvalho, Joao Mauricio Castaldelli-Maia, Sonia Cerrai, Joht Singh Chandan, Miyuru Chandradasa, Vijay Kumar Chattu, Anis Ahmad Chaudhary, Sirshendu Chaudhuri, Patrick R Ching, Hitesh Chopra, Isaac Sunday Chukwu, Sunghyun Chung, Patricia Cullen, Alanna Gomes da Silva, Koustuv Dalal, Lucio D'Anna, Samuel Demissie Darcho, Harsha Dayal, Erin M DeGraw, Syed Masudur Rahman Dewan, Diana Dias da Silva, Daniel Diaz, Sushil Dohare, Emeka W Dumbili, Jennifer Dunne, Cynthia Edeh, Michael

Ekholuenetale, Doaa Abdel Wahab El Morsi, Adeniyi Francis Fagbamigbe, Alireza Farahani, Andre Faro, Abidemi Omolara Fasanmi, Nuno Ferreira, Luisa S Flor, Deborah Ann Fry, Xiang Gao, Miglas Welay Gebregergis, Yohannes Fikadu Geda, Shakiba Ghasemi Assl, Ehsan Gholami, Nasim Gholizadeh, Elena Ghotbi, Gabriela Fernanda Gil, Jaleed Ahmed Gilani, Alem Abera Girmay, Michal Grivna, Shi-Yang Guan, Damitha Asanga Gunawardane, Sapna Gupta, Hassen Mosa Halil, Nasrin Hanifi, Habtamu Endashaw Hareru, Josep Maria Haro, Eka Mishbahatul Marah Has, Ahmed I Hasaballah, Simon I Hay, Mazeda Hossain, Md Mahbub Hossain, Md Sabbir Hossain, Mohammad Bellal Hossain, Chengxi Hu, Junjie Huang, Yongsong Huang, Pulwasha Maria Iftikhar, Lalu Muhammad Irham, Teresa R Iskander, Sheikh Mohammed Shariful Islam, Belayneh Hamdela Jena, Ravi Prakash Jha, Nitin Joseph, Charity Ehimwenma Joshua, Jiseung Kang, Kehinde Kazeem Kanmodi, Faizan Zaffar Kashoo, Himanshu Khajuria, Mariam Khalil, Iqra Hamid Khan, Ramsha Mushtaq Khan, Shaghayegh Khanmohammadi, Sameer Uttamaro Khasbage, Khalid A Kheirallah, Samira Khoshvaght, Mahmood Khosrowjerdi, Jagdish Khubchandani, Kwanghyun Kim, Felicia Marie Knaul, Ann Kristin Skrindo Knudsen, Elizabeth Koomson-Yalley, Irene Akwo Kretchy, Kewal Krishan, Barthelemy Kuate Defo, Mohammed Kuddus, Ilari Kuitunen, Mukhtar Kulimbet, Dewesh Kumar, Kamal Kumar, Rakesh Kumar, Abigail Kusi Amponsah, Asep Kusnali, John Paul Kuwornu, Frank Kyei-Arthur, Pallavi L C, Lucie Laflamme, Chandrakant Lahariya, Timo Lajunen, Berthold Langguth, Anne-Marie Laslett, Areeba Latif, Saheed Akinmayowa Lawal, Aliyu Lawan, Cheru Tesema Leshargie, Jie Li, Zhihui Li, Jue Liu, Erand Llanaj, Kevin Sheng-Kai Ma, Zheng Feei Ma, Farzan Madadizadeh, Aurea Marilia Madureira-Carvalho, Nozad Hussein Mahmood, Abdelrahman M Makram, Trisha Mallick, Deborah Carvalho Malta, Sammer Marzouk, Clara N Matei, Yasith Mathangasinghe, Khurshid A Mattoo, Pallab K Maulik, Ikechukwu Innocent Mbachu, Susan A McLaughlin, Steven M McPhail, Hadush Negash Meles, Walter Mendoza, Ritesh G Menezes, Tomislav Mestrovic, Sachith Mettananda, Sandrine Donfack D Mewoabi, Andrea Michelerio, Ted R Miller, Mojgan Mirghafourvand, Chaitanya Mittal, Mona Gamal Mohamed, Nouh Saad Mohamed, Khabab Abbasher Hussien Mohamed Ahmed, Sakineh Mohammad-Alizadeh-Charandabi, Abdollah Mohammadian-Hafshejani, Shafiu Mohammed, Ali H Mokdad, Lorenzo Monasta, Yousef Moradi, Rafael Silveira Moreira, Kimia Mozahheb Yousefi, Oscar J Mujica, Karikalan Nagarajan, Shumaila Nargus, Abdulqadir J Nashwan, Mahmoud Nassar, Samidi Nirasha Kumari Navaratna, Biswa Prakash Nayak, Ionut Negoi, Cao Duy Nguyen, Cuong Tat Nguyen, Huong Lan Thi Nguyen, Long Nguyen, Mamoona Noreen, Amir Norouzy, Chisom Adaobi Nri-Ezedi, Chijindu N Nwakama, Osaretin Christabel Okonji, John Olayemi Okunlola, Sandersan Onie, Obinna E Onwujekwe, Atakan Orscelik, Esteban Ortiz-Prado, Augustus Osborne, Uchechukwu Levi Osuagwu, Amel Ouyahia, Mahesh P A, Jagadish Rao Padubidri, Raul Felipe Palma-Alvarez, Ioannis Pantazopoulos, Romil R Parikh, Ava Pashaei, Maja Pasovic, Jay Patel, Shankargouda Patil, Shrikant Pawar, Shubhadarshini Pawar, Jalandhar Pradhan, Elton Junio Sady Prates, Shuby Puthussery, Jagadeesh Puvvula, Ibrahim Qattea, Xiang Qi, Yanan Qiao, Kabir Ayobami Raheem, Vafa Rahimi-Movaghar, Md. Mosfequr Rahman, Ivano Raimondo, Sathish Rajaa, Mahmoud Mohammed Ramadan, Chhabi Lal Ranabhat, Sowmya J Rao, Devarajan Rathish, Santosh Kumar Rauniyar, Jefferson Antonio Buendia Rodriguez, Leonardo Roever, Luca Ronfani, Moustaq Karim Khan Rony, Allen Guy Patrick Ross, Hanieh Rouzbahani, Shiva Rouzbahani, Priyanka Roy, Sharmistha Roy, Robert Rudolf, Cameron John Sabet, Umar Saeed, Rajesh Sagar, Dominic Sagoe, Pragyan Monalisa Sahoo, Dauda Salihu, Abdallah M Samy, Damian F Santomauro, Milena M Santric-Milicevic, Gargi Sachin Sarode, Sachin C Sarode, Maheswar Satpathy, Siddharthan Selvaraj, Yashendra Sethi, Muhammad Shahab, Samiah Shahid, Moyad Jamal Shahwan, Anas Shamsi, Alfiya Shamsutdinova, Dan Shan, Mohd Shanawaz, Mohammed Shannawaz, Vishal Sharma, Mahabalesh Shetty, Premalatha K Shetty, Md Monir Hossain Shimul, Aminu Shittu, Seyed

Afshin Shorofi, Emmanuel Edwar Siddig, Gustavo Correia Basto da Silva, Akanksha Singh, Marco Aurelio Sousa, Cory N Spencer, Muhammad Haroon Stanikzai, Caroline Stein, Dan J Stein, Brendon Stubbs, Vetriselvan Subramaniyan, Mahwish Suhaib, Mark J M Sullman, Sree Sudha T Y, Rafael Tabarés-Seisdedos, Celine Tabche, Mircea Tampa, Minale Tareke, Anika Tasnim, Mohamad-Hani Temsah, Azimeraw Arega Tesfu, Marcos Roberto Tovani-Palone, Tam Quoc Minh Tran, Thang Huu Tran, Samuel Joseph Tromans, Claudia Truppa, Alexander C Tsai, Evangelia Eirini Tsermpini, Aisha Twalibu, Bhaskaran Unnikrishnan, Zahir Vally, Nadia Machado Vasconcelos, Aliscia Vieira, David Villarreal-Zegarra, Manish Vinayak, Theo Vos, Yasir Waheed, Qingzhi Wang, Wei Wang, Yuan-Pang Wang, Nuwan Darshana Wickramasinghe, Martin Wiredu Agyekum, Wanqing Xu, Saba Yahoo (Syed), Yuqi Yang, Pengpeng Ye, Renjulal Yesodharan, Dong Keon Yon, Naohiro Yonemoto, Umar Yunusa, Mohammed G M Zeariya, Alemu Birara Zemariam, Beijian Zhang, Haijun Zhang, Mikhail Zinchuk, Mohamed Ali Zoromba, and Sa'ed H Zyoud.

Managing the estimation or publications process

Jack Cagney, Luisa S Flor, Simon I Hay, Ali H Mokdad, Christopher J L Murray, Erin M O'Connell, Maja Pasovic, and Cory N Spencer.
